# A directed enolization strategy enables by-product-free construction of contiguous stereocentres en route to complex amino acids

**DOI:** 10.1038/s41557-024-01473-5

**Published:** 2024-04-02

**Authors:** Fenglin Hong, Timothy P. Aldhous, Paul D. Kemmitt, John F. Bower

**Affiliations:** 1 Department of Chemistry, University of Liverpool, Liverpool, UK; 2 School of Chemistry, University of Bristol, Bristol, UK; 3 Medicinal Chemistry, Oncology, IMED Biotech Unit, AstraZeneca, Cambridge, UK

**Keywords:** Synthetic chemistry methodology, Synthetic chemistry methodology, Stereochemistry

## Abstract

Homochiral α-amino acids are widely used in pharmaceutical design as key subunits in chiral catalyst synthesis or as building blocks in synthetic biology. Many synthetic methods have been developed to access rare or unnatural variants by controlling the installation of the α-stereocentre. By contrast, and despite their importance, α-amino acids possessing β-stereocentres are much harder to synthesize. Here we demonstrate an iridium-catalysed protocol that allows the direct upconversion of simple alkenes and glycine derivatives to give β-substituted α-amino acids with exceptional levels of regio- and stereocontrol. Our method exploits the native directing ability of a glycine-derived N–H unit to facilitate Ir-catalysed enolization of the adjacent carbonyl. The resulting stereodefined enolate cross-couples with a styrene or α-olefin to install two contiguous stereocentres. The process offers very high levels of regio- and stereocontrol and occurs with complete atom economy. In broader terms, our reaction design offers a unique directing-group-controlled strategy for the direct stereocontrolled α-alkylation of carbonyl compounds, and provides a powerful approach for the synthesis of challenging contiguous stereocentres.

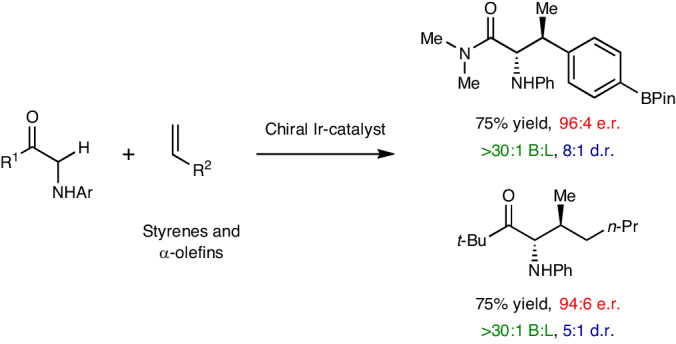

## Abstract (web summary)

α-Amino acids possessing β-stereocentres are difficult to synthesize. Now, an iridium-catalysed protocol allows the direct upconversion of simple alkenes and glycine derivatives to give β-substituted α-amino acids with exceptional levels of regio- and stereocontrol. The reaction design is based on exploiting the native directing ability of a glycine-derived N–H unit to facilitate enolization of the adjacent carbonyl.

## Main

**Fig. 1:**
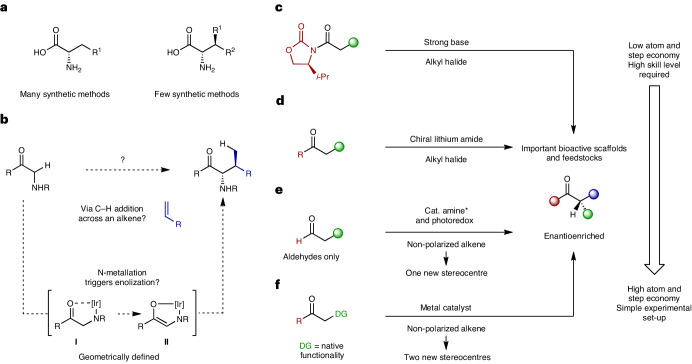
A directed enolization strategy for the hydroalkylative coupling of glycine derivatives and alkenes to give synthetically challenging β-substituted α-amino acids. **a**, β-stereogenic α-amino acids are relatively difficult to access. **b**, This work outlines an N-directed enolization method that enables the stereocontrolled and branch selective C–H addition of glycine-based units across alkenes. **c**,**d**, Conventional stereoselective carbonyl α-alkylation methods require pre-functionalization of one or both reactions partners^17–19^. **e**, Catalyst-controlled α-C–H additions across mono-substituted alkenes can be achieved in a linear selective manner^24^. The asterisk indicates chiral. **f**, This study provides a directing-group-controlled framework for achieving stereocontrolled, branch selective additions of α-C–H bonds across alkenes.

Amino acids are arguably the most valuable homochiral building blocks available to synthetic chemists. This has stimulated the development of a variety of methods to access rare or unnatural variants, focusing predominantly on control of the α-stereocentre^1^. Exemplar catalytic asymmetric methods include Strecker reactions^2^, phase-transfer-catalysed alkylations of glycine imines^3^, alkene hydrogenations^4^, cross-couplings^5^ and conjugate additions^6,7^. Although highly effective, these approaches are not generally suitable for accessing amino acids possessing β-stereocentres (Fig. 1a). Substitution at this position has important ramifications for the three-dimensional structure of a derived peptide^8,9^, for example, or the physiochemical properties of a downstream product. As testament to this, a variety of biosynthetic processes are known that allow the β-functionalization of canonical amino acids^10^. A handful of catalytic asymmetric methods have emerged that allow the synthesis of certain β-stereogenic α-amino acids. These include biocatalytic dynamic kinetic resolutions^11^, diastereoselective C–H arylations^12^, asymmetric hydrogenations^13^ and stereoretentive cross-couplings^14^. These important approaches each have their own limitations and are non-trivial, requiring, for example, a preassembled framework and/or pre-installed homochirality and/or pre-functionalized reaction partners.

We questioned whether an alternative and more convergent approach could be achieved by the direct and stereocontrolled C–H alkylation of a glycine-based precursor (Fig. 1b). In particular, we targeted a process where the new C–C bond and the two stereocentres are established in a single operation. In essence, this requires the invention of a catalytic method that allows the enantio- and diastereoselective direct (that is, stoichiometric base-free) intermolecular α-alkylation of carbonyl compounds. For systems that lack additional strong acidifying groups^3^, this area has proven to be exceptionally challenging^15,16^, such that auxiliary-based approaches are still dominant in target-directed synthesis (Fig. 1c)^17,18^. Asymmetric ketone α-alkylation can be achieved from lithium enolates using catalytic quantities of a chiral amine ligand (Fig. 1d)^19^. Other catalytic enantioselective methods have emerged, but these are not usually direct, relying either on the pre-formation of an enolate or enolate equivalent^20–22^ or the pre-installation of sacrificial functionality^23^. In a key advance, a tricatalytic system was developed that promotes the direct linear selective α-alkylation of aldehydes (Fig. 1e)^24^. This process is also important because it harnesses readily available non-activated alkenes as alkylating agents for enantioselective α-functionalization reactions^25^. Also developed is an alternative Ir-catalysed C–H activation-based branch-selective process that offers promising levels of stereocontrol^26^. Although elegant, these methods are not applicable to the issue at hand, because they are reliant on a condensation event to generate an enamine.

In this Article we outline an alternative approach that is predicated on using the glycine-based N–H unit as a directing group (**I**) to trigger metal-catalysed ‘soft’ enolization en route to geometrically defined homochiral enolates of type **II** (Fig. 1b)^27–29^. At the outset, this proposition was considered tentative because of the low acidity of **I**. Nevertheless, based on our earlier studies involving N-directed C–C bond activation^30^, we were drawn to diphosphine-modified cationic Ir(I) systems as mild Lewis acids for the proposed enolization process (**I** to **II**). A synergistic benefit of these systems resides in the observation that they can also promote the non-enantioselective (and mechanistically unclear) branch-selective addition of highly activated 1,3-dicarbonyls across alkenes^31–33^. Accordingly, our reaction design required the metal catalyst to activate both a relatively non-acidic pronucleophile and a non-polarized proelectrophile. As outlined in the following, the realization of this approach (1) addresses the immediate issue of accessing β-substituted α-amino acids, (2) offers a unique directing-group-based approach to the direct stereocontrolled α-alkylation of low-acidity carbonyl compounds (Fig. 1f) and (3) provides a broader cross-coupling framework for the by-product-free and stereocontrolled installation of contiguous stereocentres, which is a formidable issue^34^. Very recently, we reported enantioselective decarboxylative Takeuchi-type processes that use highly acidic and directing 2-aza-aryl acetates as the pronucleophile^35^. Compared to this, the work described herein represents a major advance because it allows the α-alkylation of much less acidic C–H bonds, and the installation of contiguous stereocentres. Additionally, the directing mode has potentially wider generality and the processes are mechanistically distinct.

## Results and discussion

As part of our early studies towards the envisaged process, we explored the potential C–H addition of amide-based systems **1a**–**f** across styrene **2a** (600 mol%). Because the nature of the N-substituent was deemed to be a critical factor, a range of options were evaluated using Ir(cod)_2_BARF (5 mol%) (cod = 1,5-cyclooctadiene; BARF = tetrakis(3,5-bis(trifluoromethyl)phenyl)borate) and (*R*)-BINAP (**L1**) (5 mol%) in toluene at 130 °C (Table 1). Carbamate (**1a**), sulfonamide (**1b**), amide (**1c**), *N*-benzhydryl (**1d**) and free amine (**1e**) systems were all ineffective. In contrast, aniline derivative **1f** did lead to α-alkylation product **3fa** in 86% yield, >30:1 branched:linear selectivity, 9:1 d.r. and 96:4 e.r. (entry 1). To improve on this remarkable preliminary result, other chiral diphosphine ligands were assayed, and this revealed that replacing **L1** with (*R*)-SEGPHOS (**L5**) was beneficial (entries 2–9). Further studies established that 1,4-dioxane is an effective solvent, offering marginal improvements to enantioselectivity (entries 10–14). The precise nature of the precatalyst is important: counterions that are more strongly coordinating than BARF are less effective (entries 15–18), whereas use of an analogous Rh-complex was not successful (entry 19). Using the combination of Ir(cod)_2_BARF and **L5**, we optimized the loading of styrene, leading to the conditions outlined in entry 21, which use just two equivalents. The reaction temperature can be lowered to 110 °C (entry 23), although subsequent scope studies were conducted at 130 °C. In all entries, branched-to-linear selectivities exceeded 30:1.

**Table 1:**
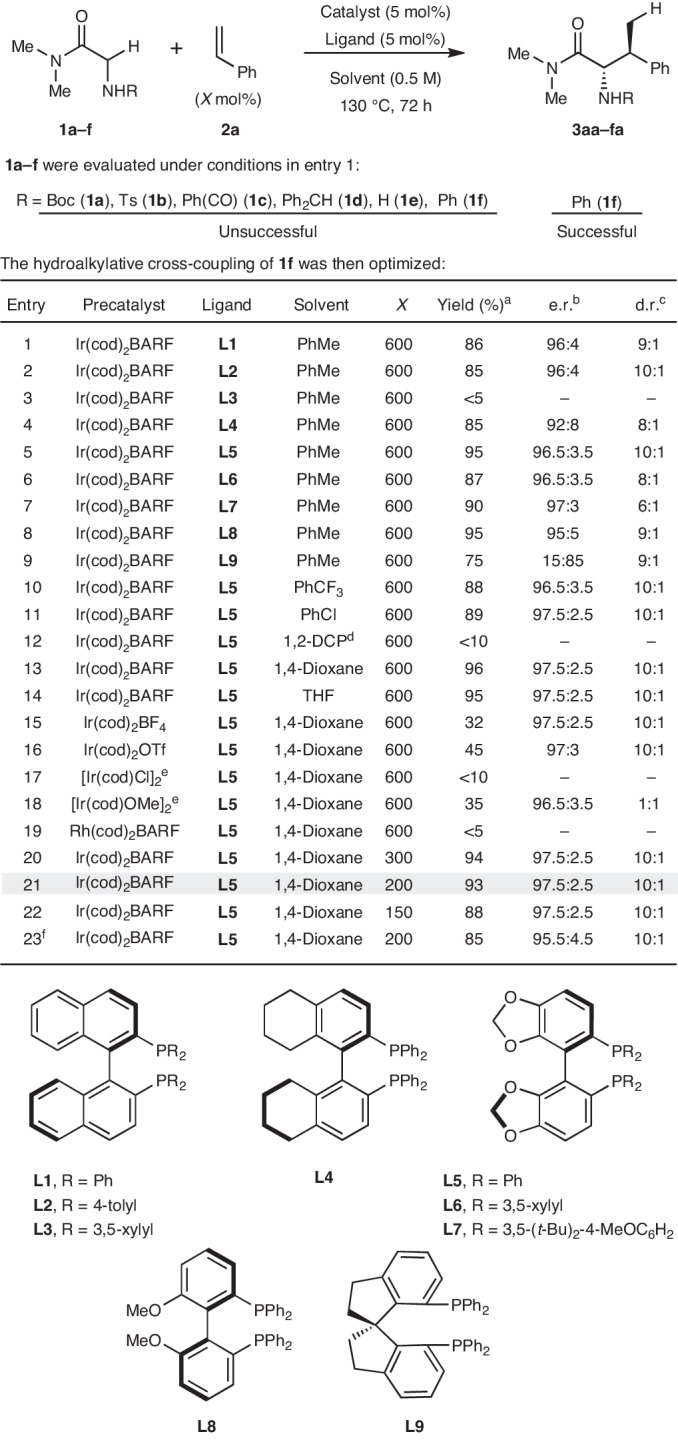
Reaction discovery and the optimization of hydroalkylative cross-coupling

^a^Measured by ^1^H NMR using 1,3,5-trimethoxybenzene as the internal standard.

^b^Determined by chiral supercritical fluid chromatography analysis.

^c^Determined by ^1^H NMR analysis of the reaction mixture.

^d^1,2-DCP = 1,2-dichloropropane.

^e^2.5 mol% of the precatalyst was used.

^f^The reaction was performed at 110 °C.

We have explored the scope of the process, and found that it is effective for the coupling of a range of secondary and tertiary amide-based systems (**1f**–**l**) with styrene **2a** (Table 2). Primary amides also participate with high enantioselectivity, but in more modest yield (Supplementary Fig. 1). Notably, ketone-based systems also participate; for example, using (*R*)-3,5-(*t*-Bu)_2_-8*H*-BINAP as the ligand (not depicted), phenyl ketone-based adduct **3ma** was accessed in good yield, high diastereoselectivity and with promising enantioselectvity. Using **L7** as the ligand, methyl-ketone-based system **3na** was generated in 90:10 e.r.; here, C–C bond formation occurred at the more hindered side of the ketone, demonstrating a further benefit of the directing-group-controlled approach. The method even tolerates very hindered ketones, such that *t*-butyl system **3oa** could be accessed in high yield. The scope of the directing *N*-aryl unit has been investigated, and this revealed that a broad range of systems are viable. Of particular note is the success of *N*-4-hydroxylphenyl (**3pa** and **3wa**) and *N*-4-methoxyphenyl systems (**3qa**), as the aryl units of these products can be removed easily (vide infra). The method also offers very wide scope with respect to the *R*^2^ unit of the styrene coupling partner. A variety of electron-rich and electron-poor systems participated smoothly, including those possessing sterically demanding *ortho*-substitution (for example, **3fl**). Heteroaryl- and ferrocenyl-substituted systems **2f** and **2g** cross-coupled with high degrees of efficiency. Notably, α-olefins can also participate smoothly, although these less reactive proelectrophiles require more readily enolizable ketone-based systems. **L6** offered optimal efficiencies for *t*-butyl ketone **1o**, delivering targets **3op**–**3os** in 71:29 to 96:4 e.r. and 2:1 to 16:1 d.r. The tolerance of the protocol to a wide range of alkene coupling partners bodes well for further development and applications. To highlight the potential of the method for complex molecule synthesis, we prepared indomethacin-derived styrene **2t**. Exposure of this to **1f** under optimized conditions delivered target **3ft** in 63% yield, 98:2 e.r., >30:1 branched-to-linear selectivity and 10:1 d.r. Although the protocol tolerates very sensitive functionality (for example, the –BPin unit of **3fh**), certain limitations (for example, alkenes attached to basic heteroarenes, 1,1- and 1,2-disubstituted alkenes, *ortho-*substituted –NHAr units) have been identified, and these are summarized in Supplementary Fig. 2.

**Table 2:**
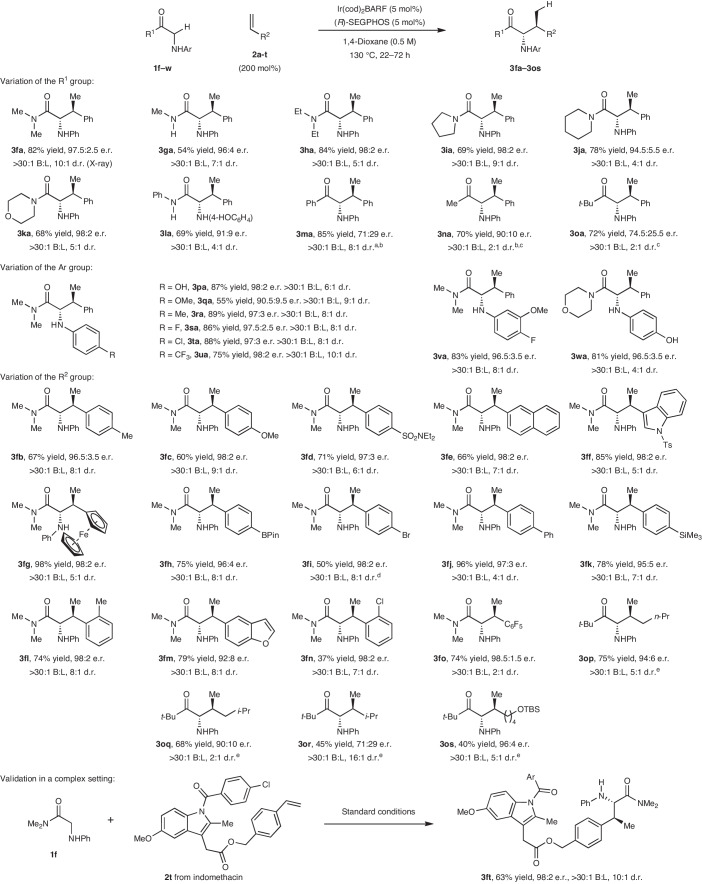
Scope of the hydroalkylation process

^a^(*R*)-3,5-(*t*-Bu)_2_-8*H*-BINAP was used as the ligand.

^b^The reaction temperature was 90 °C.

^c^(*R*)-DTBM-SEGPHOS (**L7**) was used as the ligand.

^d^The reaction temperature was 140 °C, and amide starting material was recovered in 35% yield.

^e^Alkene (1,000 mol%), (*R*)-DM-SEGPHOS (**L6**) (10 mol%) and [Ir(cod)_2_]BARF (10 mol%) were used at 110 °C in mesitylene.

**Fig. 2:**
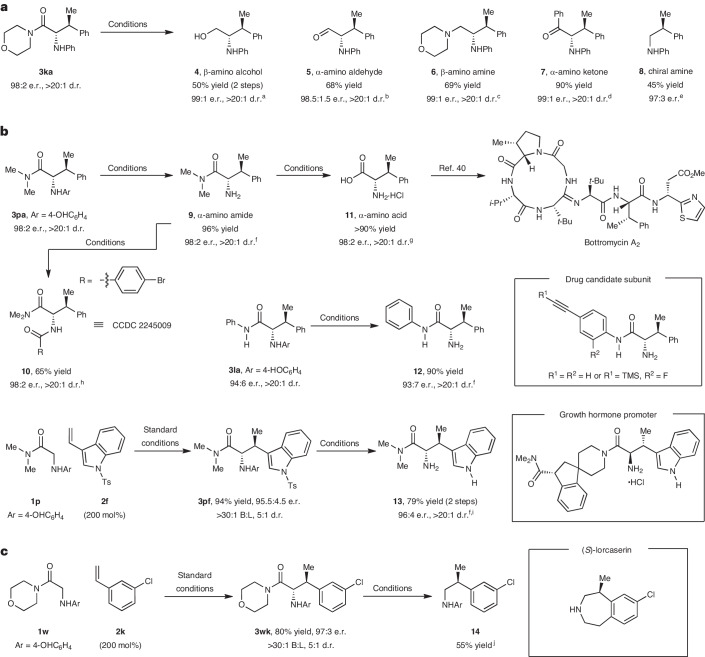
Utility of the alkylation process. **a**, Derivatizations of morpholine amide product **3ka**. **b**, Applications to the synthesis of biologically active frameworks. **c**, Application to the synthesis of a lorcaserin subunit. ^a^LiAlH_4_ (200 mol%), THF, 0 °C, 20 min, then NaBH_4_ (400 mol%), MeOH, 0 °C to r.t., 1 h. ^b^LiAlH_4_ (200 mol%), THF, 0 °C, 20 min. ^c^BH_3_•THF (500 mol%), THF, 90 °C, 4 h. ^d^PhLi (200 mol%), THF, 0 °C, 15 min. ^e^H_2_SO_4_/AcOH/H_2_O, 120 °C, 72 h. ^f^[Bis(trifluoroacetoxy)iodo]benzene (120 mol%), MeCN/H_2_O, 0 °C, 1 h. ^g^HCl/AcOH, 130 °C, 48 h. ^h^4-Bromobenzoyl chloride (100 mol%), Et_3_N (200 mol%), CH_2_Cl_2_, 0 °C to r.t., 12 h. ^i^NaH (200 mol%), *N*,*N*-dimethylacetamide, 60 °C, 3 h. ^j^HCl/AcOH, 150 °C, 72 h. B:L, branched:linear.

The initial alkylation products are readily derivatized to a range of potentially valuable amino-containing building blocks. For example, reductive manipulations of morpholino-amide **3ka** provided selective access to the corresponding amino alcohol **4**, aldehyde **5** and amine **6** (Fig. 2a). Weinreb-amide-like ketone syntheses are also possible, as demonstrated by the formation of **7** (ref. ^36^). Hydrolytic decarboxylation provided chiral amine **8** in 97:3 e.r.; decarboxylations of this type are unusual, and further studies are being undertaken to rationalize the facility of this process^37^. Systems possessing 4-hydroxyphenyl units on nitrogen can easily be deprotected to the free amine under oxidative conditions. For example, treatment of **3pa** with [bis(trifluoroacetoxy)iodo]benzene provided amino-amide **9** in 96% yield (Fig. 2b). Derivatization of this to its corresponding *p*-bromophenyl amide **10** allowed determination of the relative and absolute configuration by single-crystal X-ray diffraction, and the stereochemistry of other catalysis products was assigned on this basis. Hydrolysis of the amide of **9** provided the corresponding amino acid **11**, which is a critical subunit in a range of biologically relevant targets, including peptide β-turn mimics^38^, endomorphin analogues^39^ and the natural product bottromycin A_2_ (depicted)^40^. Compared to previous syntheses, this method for accessing **11** is notable for both its brevity and the level of stereocontrol. N-deprotection of **3la** provided **12**, which maps onto the subunit of mitogen-activated protein kinase kinase inhibitor candidates^41^. Similarly, we were able to access the subunit of a growth hormone promoter candidate by devising a two-step conversion of **1p** to **13** (ref. ^42^). The ability to access chiral amines by decarboxylation of the initial catalysis products was exploited in the conversion of **1w** to **14** via **3wk**, offering access to the key subunit of the weight-loss drug (*S*)-lorcaserin (Fig. 2c)^43^.

**Fig. 3:**
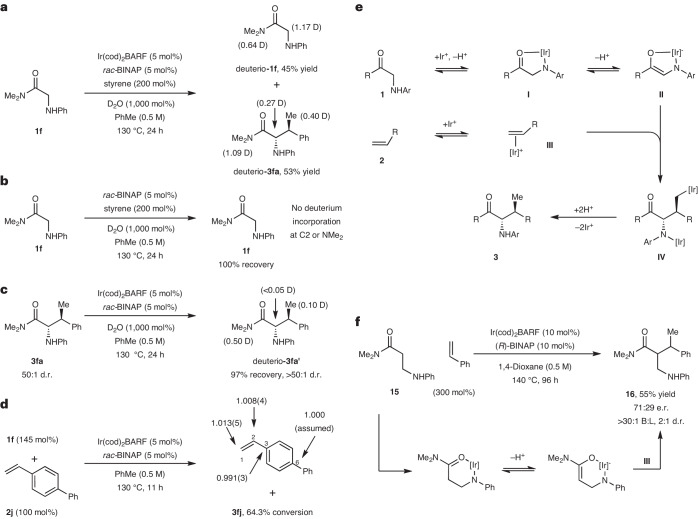
Mechanistic analysis leads to further scope. **a**, A deuterium exchange experiment reveals deuterium incorporation at the α-C–H of both the starting material and the product. **b**, A deuterium exchange experiment indicates that enolization does not occur in the absence of the Ir catalyst. **c**, A deuterium exchange experiment indicates that the product does not enolize under the reaction conditions. **d**, ^13^C KIEs indicate that both carbon centres of the alkene are involved in the turnover-limiting step. **e**, A possible mechanistic pathway. **f**, Homologated pronucleophiles also participate. Further control experiments and mechanistic discussion are provided in Supplementary Figs. 3–5. B:L, branched:linear.

Control experiments confirmed that C–C bond formation requires the Ir complex, the carbonyl unit and an NHAr unit (Supplementary Fig. 3). Exposure of **1f** and **2a** to optimized conditions but in the presence of D_2_O resulted in deuterium incorporation at C2 of both the product *deuterio*-**3fa** and the starting material *deuterio*-**1f** at partial conversion (Fig. 3a). Incorporation was also observed in the *N*-methyl groups, probably via reversible amide-directed C–H activation of this position (Supplementary Fig. 4)^44^. In the absence of the Ir complex, no deuterium incorporation was observed at C2 (Fig. 3b). Resubjection of diastereomerically pure product **3fa** to the reaction conditions in the presence of D_2_O resulted in no epimerization and no deuterium incorporation at C2 (Fig. 3c), although incorporation was observed at the methyl groups (*deuterio*-**3fa′**), presumably as a result of reversible amide-directed C–H activation. Collectively, these results indicate that the envisaged Ir-catalysed enolization of **1f** is feasible, whereas the product is resistant to this process, presumably because of the A(1,3)-strain that would arise in the resulting enolate. Efforts to isolate and characterize a chelate related to **I** or **II** have so far been unsuccessful, with NMR experiments indicating that this is not a resting state for catalysis. Using alkene **2j** as the limiting reagent, natural-abundance ^13^C kinetic isotope effects (KIEs) were determined according to the Singleton method (Fig. 3d)^45,46^. Appreciable KIEs were observed at C1 (1.013) and C2 (1.008). Based on this, we currently favour a mechanism involving turnover-limiting carbometallation from an Ir–*π* complex. Accordingly, our working mechanistic framework is outlined in Fig. 3e. To initiate the process, N–H metallation of **1** provides **I**. Related metallations have been demonstrated within the context of C–C-bond activation processes using cationic Rh(I) complexes^30^. In the current scenario, the N-metallated unit of **I** functions as a Lewis acid to trigger enolization and provide **II**. At this stage, the Ir centre may or may not be deprotonated, with the former option depicted in Fig. 3e. Deprotonation could be facilitated by the –NHAr unit of another molecule of **1**; note that exogenous bases (for example, Et_3_N, K_2_CO_3_) are detrimental to reaction efficiency. Our observations suggest the carbonyl unit must either be strongly coordinating (for example, an amide) to enhance access to **I**, or relatively acidifying (for example, a ketone) to facilitate enolization (cf. **I** to **II**). Thus, amide or ketone-based systems are effective, whereas, at the current level of development, ester analogues are not suitable. To facilitate carbometallation, the alkene component is then activated by *π*-coordination to either **II** or another Ir centre (**III**). To distinguish these options, graphical kinetic analysis was undertaken, revealing the order in catalyst to be ~2 (Supplementary Fig. 5)^47^. Accordingly, we favour a bimetallic pathway leading to **IV**, which then undergoes protodemetallation to release the product. Although not depicted, one or other of the Ir centres of **IV** may be coordinated to the carbonyl unit. The primary factor that governs enantioselectivity is probably the chiral information embodied within Ir-enolate **II**, because this is proximal to both reaction partners in the C–C-bond-forming step. The structural features of substrate **1** and *π*-coordinated alkene complex **III** are both likely to have a substantial influence on the diastereoselectivity of the process. The high branch selectivity during the conversion of **II** to **IV** may reflect electronic effects and/or a preference for the Ir centre of **III** to move to the less hindered end of the alkene.

## Outlook and conclusions

The mechanistic analysis outlined in Fig. 3e raises many interesting possibilities for future development. For example, an intriguing option is to investigate whether the pronucleophile and the proelectrophile can be activated using two different metal complexes. This would raise interesting possibilities for effecting stereocontrol. Perhaps more simply, the current catalyst system might be suitable for a wider range of directing modes. To probe this, we investigated the α-alkylation of amide **15**, where the directing group is moved one methylene unit further from the carbonyl unit (Fig. 3f). Remarkably, using styrene as the coupling partner, we were able to generate target **16** in 55% yield and with promising levels of selectivity^48^. Clearly, further refinement is required, but the result is important because it shows that the directed enolization approach has wider applicability. More broadly, our study is important because enantioselective C(*sp*^3^)–H additions to alkenes are achieved by exploiting ‘native’ directing functionality. This has a clear parallel to Murai’s seminal *ortho*-directed alkene hydroarylations^49^, a report that ignited the field of metal-catalysed C(*sp*^2^)–H functionalization, and has led to an emerging family of by-product-free enantioselective cross-couplings^50^. Our laboratory is now focused on developing a complementary set of C(*sp*^3^)–H-based cross-couplings by harnessing the design principles outlined here.

## Online content

Any methods, additional references, Nature Portfolio reporting summaries, source data, extended data, supplementary information, acknowledgements, peer review information; details of author contributions and competing interests; and statements of data and code availability are available at 10.1038/s41557-024-01473-5.

### Supplementary information

Supplementary Information Supplementary Figs. 1–5, experimental procedures, analytical data, spectra.

Supplementary Data 1 Crystallographic data for compound **3fa**; CCDC reference 2246104.

Supplementary Data 2 Crystallographic data for compound **10**; CCDC reference 2245009.

## Data Availability

The Supplementary Information contains experimental procedures and data that support this study, including Supplementary Figs. 1–5 and analytical data listings and NMR spectra. Crystallographic data for the structures reported in this Article have been deposited at the Cambridge Crystallographic Data Centre under deposition nos. CCDC 2245009 (**10**) and 2246104 (**3fa**). Copies of the data can be obtained free of charge via https://www.ccdc.cam.ac.uk/structures/.
