# Supplementary material for: A directed enolization strategy enables by-product-free construction of contiguous stereocentres en route to complex amino acids
Source: Nat Chem. 2024 Apr 2;16(7):1125–32. doi: 10.1038/s41557-024-01473-5 (PMC11230901; doi:10.1038/s41557-024-01473-5)
Supplement: Supplementary file 1 — Supplementary Figs. 1–5, experimental procedures, analytical data, spectra. [file 41557_2024_1473_MOESM1_ESM.pdf]

nature chemistry

Article

https://doi.org/10.1038/s41557-024-01473-5

A directed enolization strategy enables by-product-free construction of contiguous stereocentres en route to complex amino acids

In the format provided by the authors and unedited

Table of Contents

General experimental details 2
Experimental procedures and data 3
Optimization of reaction conditions 3
Substrate synthesis 4
Reaction scope 15
Supplementary Figure 1 67
Supplementary Figure 2 75
Applications and derivatizations 75
Evaluation of other directing groups 91
Mechanistic studies 92
Supplementary Figure 3: Control experiments 95
Supplementary Figure 4: Deuterium exchange and labelling experiments 98
^{13}C-KIE determination experiment 101
Supplementary Figure 5: Kinetic experiments 105
X-Ray crystallography data 108
References 109
NMR Spectra 112

General experimental details

All reagents requiring purification were purified using standard laboratory techniques according to methods published by Armarego, and Perrin (Pergamon Press, 1966). Catalytic reactions were carried out in Young-type re-sealable tubes. Styrene and other commercially available alkenes (liquid) were quickly distilled using a Hickman distilling head before use. All other commercially available alkenes (solid) were used as received without any further purification. Iridium catalysts were synthesized according to previously reported procedures[1,2]. Anhydrous THF, toluene, and CH_2Cl_2 were obtained by either passed through drying columns supplied by Anhydrous Engineering Ltd or purchased from commercial sources (Acros or Aldrich). Anhydrous 1,4-dioxane, DMF, DMA, DMSO and CH_3CN were purchased as anhydrous grade and stored over activated 4Å molecular sieves prior to use. All reactions were performed using dry solvents unless stated otherwise. Triethylamine (TEA) was distilled over CaH_2 and stored over activated 4Å molecular sieves under nitrogen. Flash column chromatography (FCC) was performed using silica gel (Aldrich 40-63 µm, 230-400 mesh). Thin layer chromatography was performed using aluminium backed 60 F_{254} silica plates. Visualisation was achieved by UV fluorescence or a basic $KMnO_4$ solution and heat. Proton nuclear magnetic resonance spectra (NMR) were recorded at 400 MHz or 500 MHz as stated. ^{13}C NMR spectra were recorded at 125 MHz as stated. Chemical shifts (δ) are given in parts per million (ppm). Peaks are described as singlets (s), doublets (d), triplets (t), quartets (q), septets (sept), multiplets (m) and broad (br.). Coupling constants (*J*) are quoted to the nearest 0.5 Hz. When compounds were isolated as a mixture of diastereoisomers, they are referred to as a (major) and b (minor). *In situ* yields were determined by employing 1,3,5-trimethoxybenzene as the internal standard. High resolution mass spectra were determined by the University of Liverpool mass spectrometry service, given to four decimal places. Mass spectra were recorded on Agilent 7200 Accurate Mass QTOF GC/MS (under condition of chemical ionization-CI) and Agilent 6540 UHD Accurate Mass Q-TOF LC/MS (under condition of electrospray ionization-ESI). Infrared spectra were recorded on a Perkin Elmer Spectrum Two FTIR spectrometer as thin films or solids compressed on a diamond plate. Melting points were determined using Reichert melting point apparatus and are uncorrected. Optical rotations were measured using an ADP440$^+$ polarimeter at the concentration and temperature stated. Enantiomeric excesses were determined using an Agilent 1290 Infinity chiral SFC as stated for each compound.

Experimental procedures and data

Optimization of reaction conditions

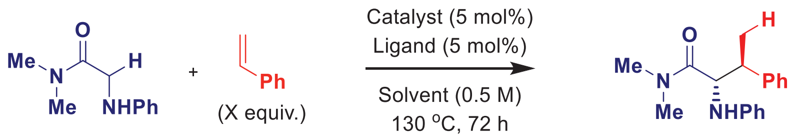

entry	ligand	catalyst	X	solvent	yield[a]	e.r.[b]	d.r.[c]
1	**L1**	$Ir(cod)_2BARF$	6	toluene	86%	96:4	9:1
2	**L2**	$Ir(cod)_2BARF$	6	toluene	85%	96:4	10:1
3	**L3**	$Ir(cod)_2BARF$	6	toluene	<5%	-	-
4	**L4**	$Ir(cod)_2BARF$	6	toluene	95%	96.5:3.5	10:1
5	**L5**	$Ir(cod)_2BARF$	6	toluene	87%	96.5:3.5	8:1
6	**L6**	$Ir(cod)_2BARF$	6	toluene	90%	97:3	6:1
7	**L7**	$Ir(cod)_2BARF$	6	toluene	85%	92:8	8:1
8	**L8**	$Ir(cod)_2BARF$	6	toluene	95%	95:5	9:1
9	**L9**	$Ir(cod)_2BARF$	6	toluene	75%	15:85	9:1
10	**L4**	$Ir(cod)_2BARF$	6	$PhCF_3$	88%	96.5:3.5	10:1
11	**L4**	$Ir(cod)_2BARF$	6	PhCl	89%	97.5:2.5	10:1
12	**L4**	$Ir(cod)_2BARF$	6	1,2-DCP	<10%	-	-
13	**L4**	$Ir(cod)_2BARF$	6	1,4-dioxane	96%	97.5:2.5	10:1
14	**L4**	$Ir(cod)_2BARF$	6	THF	95%	97.5:2.5	10:1
15	**L4**	$Ir(cod)_2BF_4$	6	1,4-dioxane	32%	97.5:2.5	10:1
16	**L4**	$Ir(cod)_2OTf$	6	1,4-dioxane	45%	97:3	10:1
17[d]	**L4**	$[Ir(cod)Cl]_2$	6	1,4-dioxane	<10%	-	-
18[d]	**L4**	$[Ir(cod)OMe]_2$	6	1,4-dioxane	35%	96.5:3.5	1:1
19	**L4**	$Rh(cod)_2BARF$	6	1,4-dioxane	<5%	-	-
20	**L4**	$Ir(cod)_2BARF$	3	1,4-dioxane	94%	97.5:2.5	10:1
**21**	**L4**	**$Ir(cod)_2BARF$**	**2**	**1,4-dioxane**	**93%**	**97.5:2.5**	**10:1**
22	**L4**	$Ir(cod)_2BARF$	1.5	1,4-dioxane	88%	97.5:2.5	10:1
23[e]	**L4**	$Ir(cod)_2BARF$	2	1,4-dioxane	85%	95.5:4.5	10:1

Reaction conditions: **1a** (0.1 mmol), catalyst (0.005 mmol), **L** (0.005 mmol), styrene (0.15, 0.2, 0.3 or 0.6 mmol), solvent (0.2 mL), 130 °C, 72 h, in Schlenk tubes. [a]Measured by ^1H NMR using 1,3,5-trimethoxybenzene as the internal standard. [b]Determined by chiral SFC analysis. [c]Determined by ^1H NMR analysis of the reaction mixture. [d]Catalyst (0.0025 mmol). [e]The reaction was performed at 110 °C.

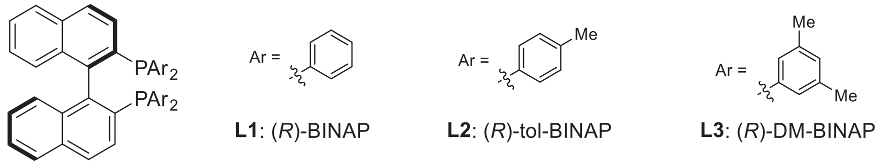

L1: (*R*)-BINAP **L2**: (*R*)-tol-BINAP **L3**: (*R*)-DM-BINAP

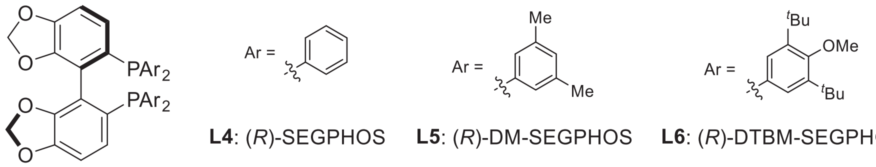

L4: (*R*)-SEGPHOS **L5**: (*R*)-DM-SEGPHOS **L6**: (*R*)-DTBM-SEGPHOS

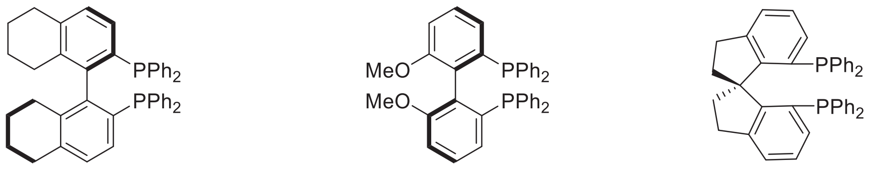

L7: (*R*)-H8-BINAP **L8**: (*R*)-MeO-BIPHEP **L9**: (*R*)-SDP

Substrate synthesis

Amides **1a**, **1c**, **1e** and **1x** were purchased and used as received. Amide **1b** and **1d** were synthesized according to the modified procedures.[3,4]

***N,N*-Dimethyl-2-((4-methylphenyl)sulfonamido)acetamide (1b):**

Amide **1b** was prepared according to the modified procedures.[3]

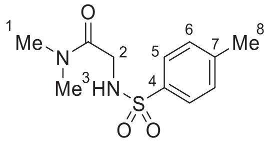

An oven-dried round bottom flask was charged with 2-amino-*N,N*-dimethylacetamide (102 mg, 1.00 mmol, 100 mol%) and DCM (5.0 mL) at r.t. TEA (0.15 mL, 1.10 mmol, 110 mol%) and 4-methylbenzenesulfonyl chloride (209 mg, 1.10 mmol, 110 mol%) were then added to the stirring solution at r.t., respectively. After the addition was complete, the reaction mixture was stirred at r.t. for 12 hours and the progress of the reaction was monitored by TLC. Upon completion, the mixture was concentrated *in vacuo*. The residue was purified by column chromatography (hexane/EtOAc, 60:40) afforded the title compound (200 mg, 78%) as a colorless solid. **m.p.** = 143 – 145 °C (hexane/EtOAc); **IR (thin film)** ν_{max}/cm^{-1}: 3207 (br), 2926 (s), 1650 (s), 1379 (s), 1331 (s), 1160 (s), 1093 (s), 665 (s); **^{1}H NMR** (500 MHz, $CDCl_3$) δ 7.77 (d, *J* = 8.0 Hz, 2H, H^5), 7.32 (d, *J* = 8.0 Hz, 2H, H^6), 5.71 (br s, 1H, H^3), 3.74 (s, 2H, H^2), 2.92 (s, 3H, H^1), 2.89 (s, 3H, $H^{1'}$), 2.43 (s, 3H, H^8); **^{13}C NMR** (126 MHz, $CDCl_3$) δ 166.6 (C=O), 143.6 (C^4), 136.1 (C^7), 129.7 (C^6), 127.2 (C^5), 43.6 (C^2), 35.81 (C^1), 35.75 ($C^{1'}$), 21.5 (C^8); **HRMS** (ESI): calculated for $C_{11}H_{16}N_2O_3SNa$ $[M+Na]^+$ requires *m/z* 279.0774, found *m/z* 279.0779.

2-(Benzylamino)-*N,N*-dimethylacetamide (1d):

Amide **1d** was prepared according to the modified procedures.[4]

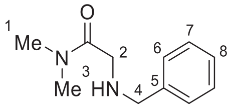

An oven-dried round bottom flask was charged with 2-bromo-*N,N*-dimethylacetamide (165 mg, 1.00 mmol, 100 mol%) and anhydrous THF (3.0 mL). Phenylmethanamine (0.22 mL, 2.00 mmol, 200 mol%) was added dropwise over 5 minutes at 0 °C. TEA (0.28 mL, 2.00 mmol, 200 mol%) in anhydrous THF (2.0 mL) was then added dropwise over 5 minutes at 0 °C. After the addition was complete, the reaction mixture was stirred for 12 hours at r.t. and the progress of the reaction was monitored by TLC. Upon completion, the mixture was concentrated *in vacuo*.

The residue was purified by column chromatography (EtOAc/MeOH, 95:5) afforded the title compound (73.0 mg, 38%) as a pale-yellow oil. **IR (thin film)** ν_{max}/cm^{-1}: 2926 (br), 1650 (s), 1496 (s), 1454 (s), 1400 (s), 1122 (s), 740 (s); **^{1}H NMR** (500 MHz, $CDCl_3$) δ 7.36 – 7.23 (m, 5H, $H^6 + H^7 + H^8$), 3.81 (s, 2H, H^4), 3.39 (s, 2H, H^2), 2.96 (s, 3H, H^1), 2.89 (s, 3H, $H^{1'}$), 2.44 (br s, 1H, H^3); **^{13}C NMR** (126 MHz, $CDCl_3$) δ 171.0 (C=O), 140.0 (C^5), 128.4 (C^7), 128.2 (C^6), 127.0 (C^8), 53.6 (C^4), 49.5 (C^2), 36.0 (C^1), 35.5 ($C^{1'}$); **HRMS**: a stable ion was not found in ESI and CI.

General procedure A for the synthesis of amides 1f-w and **1y:**

Amides **1f-w** and **1y** were synthesized according to a modified procedure.[5]

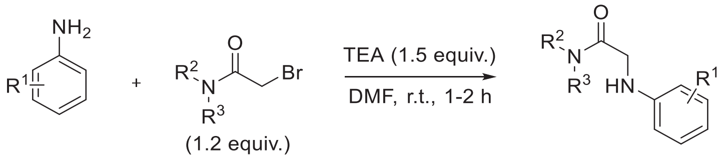

An oven-dried round bottom flask was charged with aniline (1.00 mmol, 100 mol%), TEA (0.21 mL, 1.50 mmol, 150 mol%) and anhydrous DMF (5.0 mL). α-Bromoamide (1.20 mmol, 120 mol%) in anhydrous DMF (5.0 mL) was then added dropwise over 5 minutes at room temperature (r.t.). After the addition was complete, the reaction mixture was stirred for 1-2 hour(s) at r.t. and the progress of the reaction was monitored by TLC. Upon completion, the reaction mixture was transferred to a separatory funnel, and brine (approx. 20.0 mL) was added. The aqueous phase was extracted with EtOAc (approx. 3 × 5.0 mL). The combined organic phases were dried over anhydrous $MgSO_4$, filtered and concentrated *in vacuo*. The residue was purified by FCC under the conditions noted.

***N,N*-Dimethyl-2-(phenylamino)acetamide (1f):**

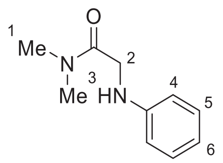

General procedure A: Purification by column chromatography (hexane/EtOAc, 50:50) afforded the title compound (116 mg, 65%) as a colorless solid. **^{1}H NMR** (500 MHz, $CDCl_3$) δ 7.21 – 7.18 (m, 2H, H^5), 6.73 – 6.71 (m, 1H, H^6), 6.63 (d, J = 7.5 Hz, 2H, H^4), 4.91 (br s, 1H, H^3), 3.85 (s, 2H, H^2), 3.03 – 3.02 (m, 6H, H^1).

The spectroscopic properties were consistent with the data available in the literature.[6]

N-Methyl-2-(phenylamino)acetamide (1g):

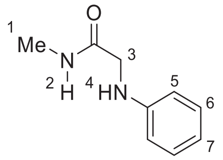

General procedure A: The reaction mixture was stirred for 19 hours at 70 °C using the corresponding α-chloride amide (129 mg, 1.20 mmol, 120 mol%). Purification by column chromatography (hexane/EtOAc, 35:65) afforded the title compound (83.7 mg, 51%) as a pale-yellow solid. **^{1}H NMR** (500 MHz, $CDCl_3$) δ 7.23 – 7.20 (m, 2H, H^6), 6.83 – 6.80 (m, 1H, H^7), 6.75 (br s, 1H, H^2), 6.61 (d, J = 8.5 Hz, 2H, H^5), 4.26 (br s, 1H, H^4), 3.79 (s, 2H, H^3), 2.82 (d, J = 5.0 Hz, 3H, H^1).

The spectroscopic properties were consistent with the data available in the literature.[7]

N,N-Diethyl-2-(phenylamino)acetamide (1h):

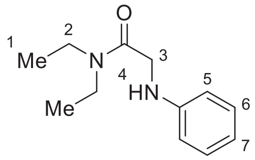

General procedure A: Purification by column chromatography (hexane/EtOAc, 50:50) afforded the title compound (144 mg, 70%) as a colorless solid. **^{1}H NMR** (500 MHz, $CDCl_3$) δ 7.22 – 7.19 (m, 2H, H^6), 6.76 – 6.73 (m, 1H, H^7), 6.68 – 6.66 (m, 2H, H^5), 5.30 (br s, 1H, H^4), 3.88 (s, 2H, H^3), 3.46 (q, J = 7.0 Hz, 2H, H^2), 3.32 (q, J = 7.0 Hz, 2H, $H^{2'}$), 1.24 (t, J = 7.0 Hz, 3H, H^1), 1.17 (t, J = 7.0 Hz, 3H, $H^{1'}$).

The spectroscopic properties were consistent with the data available in the literature.[8]

2-(Phenylamino)-1-(pyrrolidin-1-yl)ethan-1-one (1i):

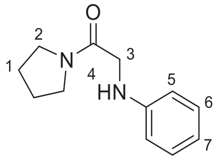

General procedure A: Purification by column chromatography (hexane/EtOAc, 65:35) afforded the title compound (120 mg, 59%) as a pale-yellow solid. **^{1}H NMR** (500 MHz, $CDCl_3$) δ 7.20 – 7.17 (m, 2H, H^6), 6.73 – 6.70 (m, 1H, H^7), 6.63 (d, J = 7.5 Hz, 2H, H^5), 4.63 (br s, 1H, H^4), 3.79 (s, 2H, H^3), 3.54 (t, J = 7.0 Hz, 2H, H^2), 3.41 (t, J = 7.0 Hz, 2H, $H^{2'}$), 2.03 – 1.98 (m, 2H, H^1), 1.91 – 1.86 (m, 2H, $H^{1'}$).

The spectroscopic properties were consistent with the data available in the literature.[6]

2-(Phenylamino)-1-(piperidin-1-yl)ethan-1-one (1j):

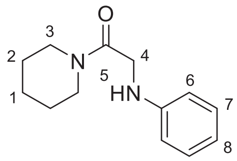

General procedure A: Purification by column chromatography (hexane/EtOAc, 65:35) afforded the title compound (80.7 mg, 37%) as a pale-yellow solid. **^{1}H NMR** (500 MHz, $CDCl_3$) δ 7.21 – 7.17 (m, 2H, H^7), 6.73 – 6.70 (m, 1H, H^8), 6.63 (d, *J* = 7.5 Hz, 2H, H^6), 4.97 (br s, 1H, H^5), 3.86 (s, 2H, H^4), 3.62 (t, *J* = 5.5 Hz, 2H, H^3), 3.37 (t, *J* = 5.5 Hz, 2H, $H^{3'}$), 1.71 – 1.66 (m, 2H, H^2), 1.63 – 1.56 (m, 4H, H^1 + $H^{2'}$).

The spectroscopic properties were consistent with the data available in the literature.[6]

1-Morpholino-2-(phenylamino)ethan-1-one (1k):

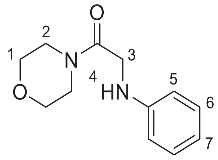

General procedure A: Purification by column chromatography (hexane/EtOAc, 50:50) afforded the title compound (123 mg, 56%) as a pale-yellow solid. **^{1}H NMR** (500 MHz, $CDCl_3$) δ 7.21 – 7.18 (m, 2H, H^6), 6.75 – 6.72 (m, 1H, H^7), 6.63 (d, *J* = 8.0 Hz, 2H, H^5), 4.82 (br s, 1H, H^4), 3.86 (s, 2H, H^3), 3.71 – 3.67 (m, 6H, H^1 + H^2), 3.45 – 3.43 (m, 2H, $H^{2'}$).

The spectroscopic properties were consistent with the data available in the literature.[6]

2-((4-Hydroxyphenyl)amino)-*N*-phenylacetamide (1l):

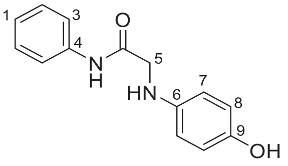

General procedure A: Purification by column chromatography (hexane/EtOAc, 35:65) afforded the title compound (148 mg, 61%) as a colorless solid. **m.p.** = 174 – 176 °C (hexane/EtOAc); **IR (thin film)** ν_{max}/cm^{-1}: 3312 (br), 1660 (s), 1598 (s), 1513 (s), 1243 (s); **^{1}H NMR** (500 MHz, CD_3OD-d^4) δ 7.55 (d, *J* = 7.5 Hz, 2H, H^3), 7.33 – 7.30 (m, 2H, H^2), 7.13 – 7.10 (m, 1H, H^1), 6.68 (d, *J* = 9.0 Hz, 2H, H^8), 6.59 (d, *J* = 9.0 Hz, 2H, H^7), 3.83 (s, 2H, H^5);

^{13}**C NMR** (126 MHz, CD_3OD-d^4) δ 171.2 (C=O), 149.7 (C^9), 141.1 (C^6), 137.8 (C^4), 128.4 (C^2), 124.1 (C^1), 120.0 (C^3), 115.6 (C^8), 114.4 (C^7), 49.4 (C^5); **HRMS** (ESI): calculated for $C_{14}H_{14}N_2O_2Na$ $[M+Na]^+$ requires *m/z* 265.0947, found *m/z* 265.0954.

2-((4-Hydroxyphenyl)amino)-*N,N*-dimethylacetamide (1p):

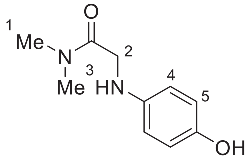

General procedure A: Purification by column chromatography (hexane/EtOAc, 50:50) afforded the title compound (118 mg, 61%) as a pale-yellow solid. ^{1}**H NMR** (500 MHz, $CDCl_3$) δ 6.72 (d, J = 9.0 Hz, 2H, H^5), 6.56 (d, J = 9.0 Hz, 2H, H^4), 4.52 (br s, 1H, H^3), 3.83 (s, 2H, H^2), 3.03 (s, 6H, H^1).

The spectroscopic properties were consistent with the data available in the literature.[9]

2-((4-Methoxyphenyl)amino)-*N,N*-dimethylacetamide (1q):

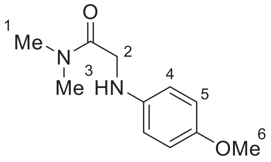

General procedure A: Purification by column chromatography (hexane/EtOAc, 50:50) afforded the title compound (146 mg, 70%) as a pale-yellow solid. ^{1}**H NMR** (500 MHz, $CDCl_3$) δ 6.79 (d, J = 9.0 Hz, 2H, H^5), 6.60 (d, J = 9.0 Hz, 2H, H^4), 4.58 (br s, 1H, H^3), 3.82 (s, 2H, H^2), 3.74 (s, 3H, H^6), 3.01 (s, 6H, H^1).

The spectroscopic properties were consistent with the data available in the literature.[10]

***N,N*-Dimethyl-2-(*p*-tolylamino)acetamide (1r):**

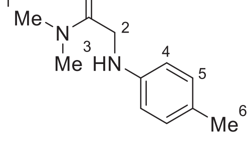

General procedure A: Purification by column chromatography (hexane/EtOAc, 50:50) afforded the title compound (131 mg, 68%) as a colorless solid. ^{1}**H NMR** (500 MHz, $CDCl_3$) δ 7.00 (d, J = 8.0 Hz, 2H, H^5), 6.57 (d, J = 8.0 Hz, 2H, H^4), 4.57 (br s, 1H, H^3), 3.84 (s, 2H, H^2), 3.02 (s, 6H, H^1), 2.24 (s, 3H, H^6).

The spectroscopic properties were consistent with the data available in the literature.[11]

2-((4-Fluorophenyl)amino)-*N,N*-dimethylacetamide (1s):

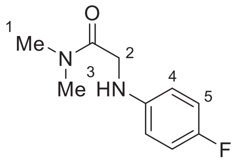

General procedure A: Purification by column chromatography (hexane/EtOAc, 50:50) afforded the title compound (141 mg, 72%) as a colorless solid. **^{1}H NMR** (500 MHz, $CDCl_3$) δ 6.91 – 6.87 (m, 2H, H^5), 6.57 – 6.54 (m, 2H, H^4), 4.72 (br s, 1H, H^3), 3.81 (s, 2H, H^2), 3.02 (s, 6H, H^1).

The spectroscopic properties were consistent with the data available in the literature.[11]

2-((4-Chlorophenyl)amino)-*N,N*-dimethylacetamide (1t):

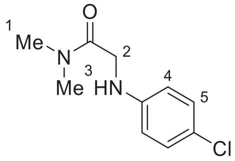

General procedure A: Purification by column chromatography (hexane/EtOAc, 50:50) afforded the title compound (160 mg, 75%) as a colorless solid. **^{1}H NMR** (500 MHz, $CDCl_3$) δ 7.12 (d, J = 8.5 Hz, 2H, H^5), 6.54 (d, J = 8.5 Hz, 2H, H^4), 4.90 (br s, 1H, H^3), 3.81 (s, 2H, H^2), 3.02 (s, 6H, H^1).

The spectroscopic properties were consistent with the data available in the literature.[11]

***N,N*-Dimethyl-2-((4-(trifluoromethyl)phenyl)amino)acetamide (1u):**

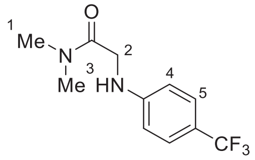

General procedure A: Purification by column chromatography (hexane/EtOAc, 50:50) afforded the title compound (143 mg, 58%) as a colorless solid. **^{1}H NMR** (500 MHz, $CDCl_3$) δ 7.42 (d, J = 8.5 Hz, 2H, H^5), 6.62 (d, J = 8.5 Hz, 2H, H^4), 5.29 (br s, 1H, H^3), 3.87 (s, 2H, H^2), 3.05 (s, 6H, H^1).

The spectroscopic properties were consistent with the data available in the literature.[11]

2-((4-Fluoro-3-methoxyphenyl)amino)-*N,N*-dimethylacetamide (1v):

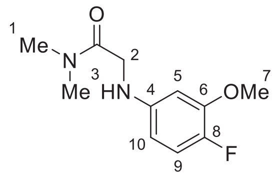

General procedure A: Purification by column chromatography (hexane/EtOAc, 50:50) afforded the title compound (129 mg, 57%) as a colorless solid. **m.p.** = 105 – 107 °C (hexane/EtOAc); **IR (thin film)** ν_{max}/cm^{-1}: 3384 (br), 2933 (s), 1652 (s), 1520 (s), 1225 (s); **^{1}H NMR** (500 MHz, $CDCl_3$) δ 6.90 – 6.86 (m, 1H, H^9), 6.28 – 6.26 (m, 1H, H^{10}), 6.06 – 6.03 (m, 1H, H^5), 4.77 (br s, 1H, H^3), 3.84 (s, 3H, H^7), 3.80 (s, 2H, H^2), 3.01 (s, 6H, H^1); **^{13}C NMR** (126 MHz, $CDCl_3$) δ 168.8 (C=O), 148.0 (d, *J* = 11.5 Hz, C^6), 145.7 (d, *J* = 234.5 Hz, C^8), 144.4 (C^4), 116.1 (d, *J* = 19.0 Hz, C^9), 103.2 (d, *J* = 6.5 Hz, C^{10}), 99.6 (C^5), 56.1 (C^7), 45.6 (C^2), 35.7 (C^1), 35.6 ($C^{1'}$); ^{19}F NMR (471 MHz, $CDCl_3$) δ -149.8; **HRMS** (ESI): calculated for $C_{11}H_{15}FN_2O_2Na$ $[M+Na]^+$ requires *m/z* 249.1010, found *m/z* 249.1012.

2-((4-Hydroxyphenyl)amino)-1-morpholinoethan-1-one (1w):

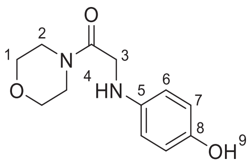

General procedure A: Purification by column chromatography (hexane/EtOAc, 50:50) afforded the title compound (135 mg, 57%) as a pale-yellow solid. **m.p.** = 178 – 180 °C (hexane/EtOAc); **IR (thin film)** ν_{max}/cm^{-1}: 3352 (br), 2860 (s), 1636 (s), 1517 (s), 1440 (s), 1114 (s); **^{1}H NMR** (500 MHz, DMSO-d^6) δ 8.43 (s, 1H, H^9), 6.56 – 6.50 (m, 4H, $H^6 + H^7$), 4.93 (t, *J* = 5.5 Hz, 1H, H^4), 3.80 (d, *J* = 5.0 Hz, 2H, H^3), 3.60 – 3.55 (m, 4H, H^1), 3.49 – 3.45 (m, 4H, H^2); **^{13}C NMR** (126 MHz, DMSO-d^6) δ 168.4 (C=O), 148.6 (C^8), 141.1 (C^5), 115.6 (C^7), 113.8 (C^6), 66.1 (C^1), 66.0 ($C^{1'}$), 45.6 (C^3), 44.5 (C^2), 41.7 ($C^{2'}$); **HRMS** (ESI): calculated for $C_{12}H_{17}N_2O_3$ $[M+H]^+$ requires *m/z* 237.1234, found *m/z* 237.1231.

***N,N*-Dimethyl-2-(*o*-tolylamino)acetamide (1y):**

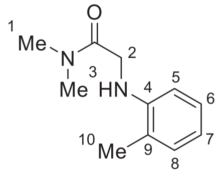

General procedure A: Purification by column chromatography (hexane/EtOAc, 70:30) afforded the title compound (67.2 mg, 35%) as a colorless solid. **m.p.** = 91 – 93 °C (hexane/EtOAc); **IR (thin film)** ν_{max}/cm^{-1}: 3400 (br), 2929 (s), 1654 (s), 1512 (s), 1398 (s), 1133 (s), (746); **^{1}H NMR** (500 MHz, $CDCl_3$) δ 7.17 – 7.14 (m, 1H, H^{6}), 7.11 (d, *J* = 6.5 Hz, 1H, H^{8}), 6.73 – 6.70 (m, 1H, H^{7}), 6.56 (d, *J* = 7.5 Hz, 1H, H^{5}), 3.93 (s, 2H, H^{2}), 3.08 (s, 6H, H^{1}), 2.27 (s, 3H, H^{10}); **^{13}C NMR** (126 MHz, $CDCl_3$) δ 169.1 (C=O), 145.2 (C^{4}), 130.2 (C^{8}), 127.0 (C^{6}), 122.8 (C^{9}), 117.4 (C^{7}), 110.0 (C^{5}), 45.4 (C^{2}), 35.8 (C^{1}), 35.7 ($C^{1'}$), 17.4 (C^{10}); **HRMS** (ESI): calculated for $C_{11}H_{17}N_2O$ $[M+Na]^+$ requires *m/z* 193.1335, found *m/z* 193.1338.

***N,N*-Dimethyl-3-(phenylamino)propanamide (15):**

Amide **15** was prepared by reported procedure.[12]

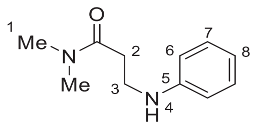

An oven-dried round bottom flask was charged with aniline (93.1 mg, 1.00 mmol, 100 mol%), *N,N*-dimethylacrylamide (129 mg, 1.30 mmol, 130 mol%) and imidazolium chloride (31.2 mg, 0.30 mmol, 30 mol%) at r.t. After the addition was complete, the reaction mixture was stirred at 120 °C for 4 hours and the progress of the reaction was monitored by TLC. Upon completion, the reaction was cooled to r.t. and then was transferred to a separatory funnel and water (approx. 10.0 mL) was added. The aqueous phase was extracted with EtOAc (approx. 3 × 5.0 mL). The combined organic phases were dried over anhydrous $MgSO_4$, filtered and concentrated *in vacuo*. The residue was purified by column chromatography (hexane/EtOAc, 50:50) afforded the title compound (96.1 mg, 50%) as a colorless solid. **^{1}H NMR** (500 MHz, $CDCl_3$) δ 7.18 – 7.15 (m, 2H, H^{7}), 6.71 – 6.68 (m, 1H, H^{8}), 6.63 (d, *J* = 7.5 Hz, 2H, H^{6}), 4.34 (br s, 1H, H^{4}), 3.49 (t, *J* = 6.0 Hz, 2H, H^{3}), 2.96 – 2.95 (m, 6H, H^{1}), 2.59 (t, *J* = 6.0 Hz, 2H, H^{2}).

The spectroscopic properties were consistent with the data available in the literature.[12]

1-Phenyl-2-(phenylamino)ethan-1-one (1m):

Ketone **1m** was prepared by the reported procedure.[13]

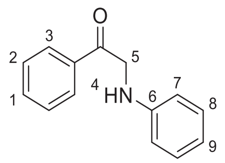

An oven-dried round bottom flask was charged with aniline (93.1 mg, 1.00 mmol, 100 mol%), $NaHCO_3$ (84.0 mg, 1.00 mmol, 100 mol%) and EtOH (5.0 mL) at r.t. 2-Bromo-1-phenylethan-1-one (198 mg, 1.00 mmol, 100 mol%) in EtOH (2.0 mL) was then added dropwise to the stirring solution over 3 minutes at r.t. After the addition was complete, the reaction mixture was stirred for 12 hours at r.t. and the progress of the reaction was monitored by TLC. Upon completion, the crude product could be obtained as a yellow precipitate, which was recrystallized from EtOH affording the title compound (158 mg, 75%) as a pale-yellow solid. **^{1}H NMR** (500 MHz, $CDCl_3$) δ 8.04 (d, J = 7.0 Hz, 2H, H^3), 7.65 – 7.62 (m, 1H, H^1), 7.54 – 7.51 (m, 2H, H^2), 7.26 – 7.22 (m, 2H, H^8), 6.78 – 6.72 (m, 3H, H^7 + H^9), 5.03 (br s, 1H, H^4), 4.63 (s, 2H, H^5).

The spectroscopic properties were consistent with the data available in the literature.[13]

General procedure B for the synthesis of ketones 1n and **1o:**

Ketones **1n** and **1o** were synthesized according to a modified procedure.[14]

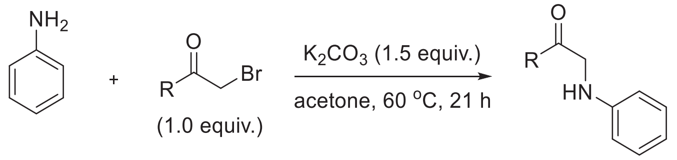

An oven-dried round bottom flask was charged with aniline (93.1 mg, 1.00 mmol, 100 mol%), K_2CO_3 (207 mg, 1.50 mmol, 150 mol%) and acetone (5.0 mL) and was stirred at 60 °C. After 1 hour, bromide ketone (1.00 mmol, 100 mol%) was then added dropwise to the stirring solution over 3 minutes at r.t. After the addition was complete, the reaction mixture was stirred for 21 hours at 60 °C and the progress of the reaction was monitored by TLC. Upon completion, the mixture was filtered and the filtrate was concentrated *in vacuo*. The residue was purified by FCC under the conditions noted.

1-(Phenylamino)propan-2-one (1n):

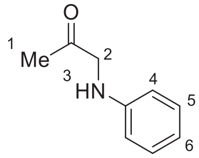

General procedure B: Purification by column chromatography (hexane/EtOAc, 85:15) afforded the title compound (89.4 mg, 60%) as a brown solid. **^{1}H NMR** (500 MHz, $CDCl_3$) 7.22 – 7.19 (m, 2H, H^5), 6.76 – 6.73 (m, 1H, H^6), 6.60 (d, J = 7.5 Hz, 2H, H^4), 4.57 (br s, 1H, H^3), 4.00 (d, J = 4.5 Hz, 2H, H^2), 2.25 (s, 3H, H^1).

The spectroscopic properties were consistent with the data available in the literature.[14]

3,3-Dimethyl-1-(phenylamino)butan-2-one (1o):

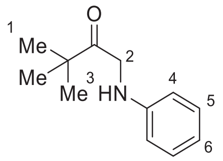

General procedure B: Purification by column chromatography (hexane/EtOAc, 90:10) afforded the title compound (155 mg, 81%) as a colorless solid. **^{1}H NMR** (500 MHz, $CDCl_3$) 7.22 – 7.19 (m, 2H, H^5), 6.75 – 6.72 (m, 1H, H^6), 6.64 (d, *J* = 7.5 Hz, 2H, H^4), 4.76 (br s, 1H, H^3), 4.12 (s, 2H, H^2), 1.24 (s, 9H, H^1).

The spectroscopic properties were consistent with the data available in the literature.[15]

Alkenes:

Styrene **2a**, simple substituted styrenes **2b-c**, **2e**, **2h-l, 2n-o** and **2u-x**, vinyl ferrocene **2g** and α-olefins **2p-r** were purchased and used as received.

Alkenes *N,N*-diethyl-4-vinylbenzenesulfonamide **2d**, 1-tosyl-3-vinyl-1*H*-indole **2f**, 5-vinylbenzofuran **2m,** *tert*-butyl(hex-5-en-1-yloxy)dimethylsilane **2s** and 4-vinylbenzyl 2-(1-(4-chlorobenzoyl)-5-methoxy-2-methyl-1*H*-indol-3-yl)acetate **2t** were prepared by reported procedures.[16-20]

***N,N*-Diethyl-4-vinylbenzenesulfonamide (2d):**

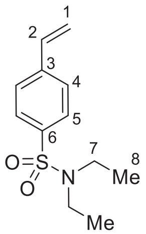

^{1}H NMR (500 MHz, $CDCl_3$) 7.77 (d, *J* = 8.5 Hz, 2H, H^5), 7.52 (d, *J* = 8.5 Hz, 2H, H^4), 6.75 (dd, *J* = 17.5, 11.0 Hz, 1H, H^2), 5.88 (d, *J* = 17.5 Hz, 1H, H^1), 5.42 (d, J = 11.0 Hz, 1H, $H^{1'}$), 3.25 (q, *J* = 7.0 Hz, 4H, H^7), 1.14 (t, *J* = 7.0 Hz, 6H, H^8).

The spectroscopic properties were consistent with the data available in the literature.[16]

1-Tosyl-3-vinyl-1*H*-indole (2f):

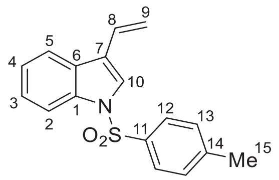

^{1}H NMR (500 MHz, $CDCl_3$) 7.99 (d, J = 8.0 Hz, 1H, H^{5}), 7.78 – 7.73 (m, 3H, $H^{2} + H^{12}$), 7.61 (s, 1H, H^{10}), 7.35 – 7.32 (m, 1H, ArH), 7.29 – 7.25 (m, 1H, ArH), 7.22 (d, J = 8.0 Hz, 2H, H^{13}), 6.77 (dd, J = 18.0, 11.5 Hz, 1H, H^{8}), 5.80 (d, J = 18.0 Hz, 1H, H^{9}), 5.35 (d, J = 11.5 Hz, 1H, $H^{9'}$), 2.33 (s, 3H, H^{15}).

The spectroscopic properties were consistent with the data available in the literature.[17]

5-Vinylbenzofuran (2m):

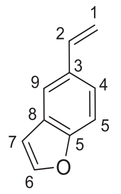

^{1}H NMR (500 MHz, $CDCl_3$) 7.65 – 7.64 (m, 2H, ArH), 7.50 – 7.48 (m, 1H, ArH), 7.44 – 7.42 (m, 1H, ArH), 6.85 (dd, J = 17.5, 11.0 Hz, 1H, H^{2}), 6.78 (d, J = 2.5 Hz, 1H, H^{7}), 5.77 (d, J = 17.5 Hz, 1H, H^{1}), 5.26 (d, J = 11.0 Hz, 1H, $H^{1'}$).

The spectroscopic properties were consistent with the data available in the literature.[18]

***Tert*-butyl(hex-5-en-1-yloxy)dimethylsilane (2s):**

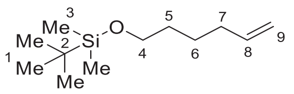

^{1}H NMR (500 MHz, $CDCl_3$) 5.85 – 5.77 (m, 1H, H^{8}), 5.02 – 4.98 (m, 1H, H^{9}), 4.95 – 4.93 (m, 1H, H^{9}), 3.61 (t, J = 6.5 Hz, 2H, H^{4}), 2.09 – 2.04 (m, 2H, H^{7}), 1.56 – 1.40 (m, 4H, H^{5} + H^{6}), 0.89 (s, 9H, H^{1}), 0.05 (s, 6H, H^{3}).

The spectroscopic properties were consistent with the data available in the literature.[19]

4-Vinylbenzyl 2-(1-(4-chlorobenzoyl)-5-methoxy-2-methyl-1*H*-indol-3-yl)acetate (2t):

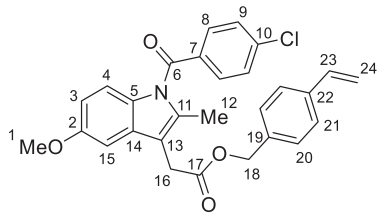

^{1}H NMR (500 MHz, $CDCl_3$) 7.64 (d, J = 8.5 Hz, 2H, H^8), 7.45 (d, J = 8.5 Hz, 2H, H^9), 7.37 (d, J = 8.0 Hz, 2H, ArH), 7.27 – 7.25 (m, 2H, ArH), 6.92 – 6.88 (m, 2H, ArH), 6.74 – 6.66 (m, 2H, ArH + H^{23}), 5.76 (d, J = 17.5 Hz, 1H, H^{24}), 5.27 (d, J = 11.0 Hz, 1H, H^{24}), 5.12 (s, 2H, H^{18}), 3.76 (s, 3H, H^1), 3.71 (s, 2H, H^{16}), 2.36 (s, 3H, H^{12}).

The spectroscopic properties were consistent with the data available in the literature.[20]

Reaction scope

General procedure C for the asymmetric α-alkylation of glycine derivatives:

A Schlenk tube was charged with substrate (0.10 mmol, 100 mol%), $[Ir(cod)_2]$BARF (6.36 mg, 5.00 μmol, 5 mol%), (*R*)-SEGPHOS (3.05 mg, 5.00 μmol, 5 mol%) and styrene derivative (if non-volatile, 200 mol%). The Schlenk tube was evacuated and refilled with N_2 (three cycles), then the alkene partner (if volatile, 200-1000 mol%) was added followed by anhydrous 1,4-dioxane (0.2 mL, 0.5 M). The tube was sealed and heated at 130 °C for 72 hours. After cooling to r.t., the solvent was removed under reduced pressure and the crude reaction mixture was purified by FCC. The racemic products were also obtained using the above procedure (*rac*-BINAP was used in place of (*R*)-SEGPHOS) and purified by FCC.

(*2S,3S*)-*N,N*-Dimethyl-3-phenyl-2-(phenylamino)butanamide (3fa):

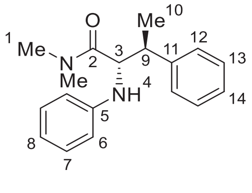

General procedure C: The reaction was carried out with substrate **1f** (17.8 mg, 0.10 mmol, 100 mol%) and styrene (23.0 μL, 0.20 mmol, 200 mol%). Purification of the residue by FCC (hexane/EtOAc 70:30) afforded the title compound (23.1 mg, 82%, >30:1 B:L, d.r. = 18:1 *a:b*, e.r. = 97.5:2.5) as a pale-yellow solid. ^{1}H NMR analysis of the crude material gave >30:1 B:L and d.r. = 10:1. **m.p.** = 103 – 105 °C (hexane/EtOAc); $[\alpha]_D^{25}$ = -7.8 (c = 1.0, $CHCl_3$); **IR (thin**

film) ν_{max}/cm^{-1}: 3335 (br), 3027 (s), 2931 (s), 1638 (s), 1602 (s), 1495 (s), 699 (s); **^{1}H NMR** (500 MHz, $CDCl_3$) Data for the major diastereomer *a*: δ 7.35 – 7.25 (m, 5H, H^{12} + H^{13} + H^{14}), 7.20 – 7.16 (m, 2H, H^{7}), 6.75 – 6.72 (m, 1H, H^{8}), 6.66 (d, *J* = 7.5 Hz, 2H, H^{6}), 5.32 – 4.13 (m, 2H, H^{3} + H^{4}), 3.39 – 3.34 (m, 1H, H^{9}), 2.92 (s, 3H, H^{1}), 2.66 (s, 3H, H$^{1'}$), 1.40 (d, *J* = 7.0 Hz, 3H, H^{10}). Characteristic signals for the minor diastereomer *b*: 2.76 (s, 3H, H^{1}), 2.64 (s, 3H, H$^{1'}$), 1.49 (d, *J* = 7.0 Hz, 3H, H^{10}); **^{13}C NMR** (126 MHz, $CDCl_3$) Data for the major diastereomer *a* only: δ 171.5 (C^{2}), 146.9 (C^{5}), 142.5 (C^{11}), 129.3 (C^{7}), 128.4 (C^{13}), 127.9 (C^{12}), 126.9 (C^{14}), 117.9 (C^{8}), 113.9 (C^{6}), 58.1 (C^{3}), 42.3 (C^{9}), 36.8 (C^{1}), 35.6 (C$^{1'}$), 15.7 (C^{10}); **HRMS** (ESI): calculated for $C_{18}H_{22}N_2ONa$ $[M+Na]^+$ requires *m/z* 305.1624, found *m/z* 305.1627; **Chiral SFC:** DAICEL CHIRALCEL OD-H column (25 cm), CO_2:*i*-PrOH 90:10, 2.0 mL/min, 145 bar, 40 °C. Retention times: 6.7 mins (major), 7.2 mins (minor), e.r. = 97.5:2.5. To facilitate analysis by SFC, the major diastereoisomer was separated from the minor diastereoisomer by FCC.

SFC analysis of the major diastereomer of the racemate, prepared using rac-BINAP:

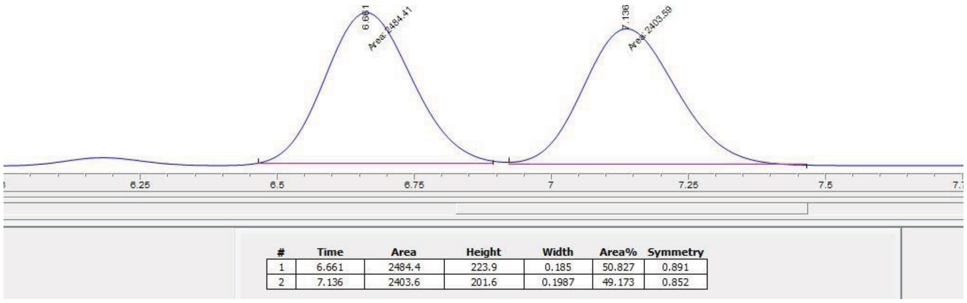

#	Time	Area	Height	Width	Area%	Symmetry
1	6.661	2484.4	223.9	0.185	50.827	0.891
2	7.136	2403.6	201.6	0.1987	49.173	0.852

SFC analysis of the major diastereomer of enantioenriched material, prepared using (R)-SEGPHOS:

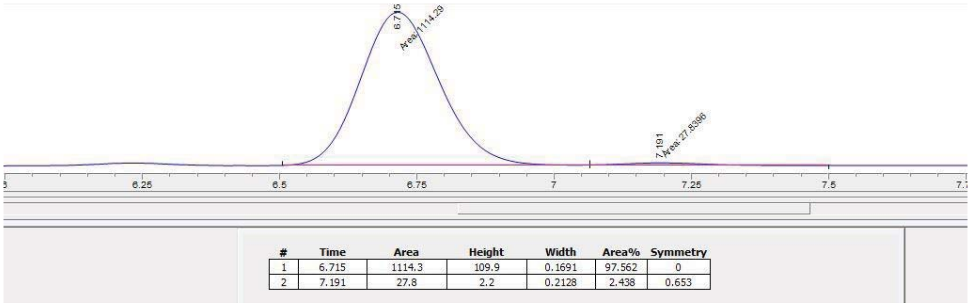

#	Time	Area	Height	Width	Area%	Symmetry
1	6.715	1114.3	109.9	0.1691	97.562	0
2	7.191	27.8	2.2	0.2128	2.438	0.653

The structure of this compound was determined by single crystal X-ray diffraction of crystals grown from EtOAc/hexane. See CCDC **2246104**.

(*2S,3S*)-*N*-Methyl-3-phenyl-2-(phenylamino)butanamide (3ga):

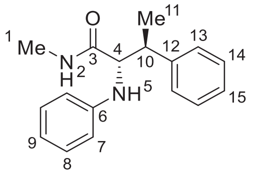

General procedure C: The reaction was carried out with substrate **1g** (16.4 mg, 0.10 mmol, 100 mol%) and styrene (23.0 µL, 0.20 mmol, 200 mol%). Purification of the residue by FCC (hexane/EtOAc 60:40) afforded the title compound (14.5 mg, 54%, >30:1 B:L, d.r. = 18:1 *a*:*b*, e.r. = 96:4) as a pale-yellow oil. ^{1}H NMR analysis of the crude material gave >30:1 B:L and d.r. = 7:1. $[\boldsymbol{\alpha}]_{\boldsymbol{D}}^{\mathbf{25}}$ = +50.3 (c = 1.0, $CHCl_3$); **IR (thin film)** ν_{max}/cm^{-1}: 3308 (br), 2928 (s), 1650 (s), 1603 (s), 1504 (s), 1316 (s), 700 (s); **^{1}H NMR** (500 MHz, $CDCl_3$) Data for the major diastereomer *a:* δ 7.39 – 7.26 (m, 5H, H^{13} + H^{14} + H^{15}), 7.20 – 7.17 (m, 2H, H^8), 6.82 – 6.79 (m, 1H, H^9), 6.57 (d, *J* = 8.0 Hz, 2H, H^7), 6.53 (br s, 1H, H^2), 4.14 – 3.72 (m, 2H, H^4 + H^5), 3.57 – 3.51 (m, 1H, H^{10}), 2.76 (d, *J* = 4.5 Hz, 3H, H^1), 1.46 (d, *J* = 7.0 Hz, 3H, H^{11}). Characteristic signals for the minor diastereomer *b*: 2.80 (d, *J* = 4.5 Hz, 2H, H^1), 1.43 (d, *J* = 7.0 Hz, 2H, H^{11}); **^{13}C NMR** (126 MHz, $CDCl_3$) Data for the major diastereomer *a* only: δ 173.2 (C^2), 146.8 (C^6), 141.2 (C^{12}), 129.4 (C^8), 128.9 (C^{14}), 127.6 (C^{13}), 127.3 (C^{15}), 119.1 (C^9), 113.7 (C^7), 64.6 (C^4), 42.1 (C^{10}), 26.0 (C^1), 18.4 (C^{11}); **HRMS** (ESI): calculated for $C_{17}H_{20}N_2ONa$ $[M+Na]^+$ requires *m/z* 291.1468, found *m/z* 291.1482; **Chiral SFC:** DAICEL CHIRALCEL OD-H column (25 cm), CO_2:*i*-PrOH 90:10, 2.0 mL/min, 140 bar, 40 °C. Retention times: 12.3 mins (major), 13.6 mins (minor), e.r. = 96:4. To facilitate analysis by SFC, the major diastereoisomer was separated from the minor diastereoisomer by FCC.

SFC analysis of the major diastereomer of the racemate, prepared using rac-BINAP:

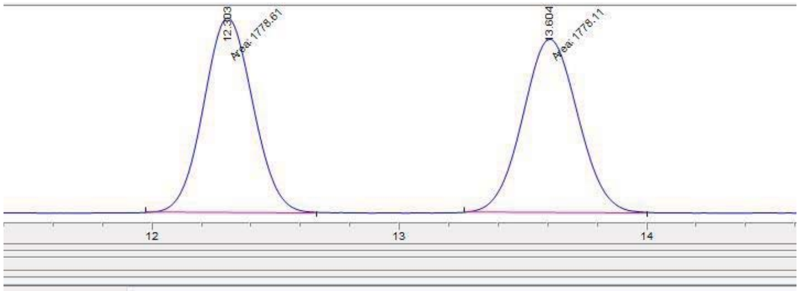

#	Time	Area	Height	Width	Area%	Symmetry
1	12.303	1778.6	127.2	0.233	50.007	0.925
2	13.604	1778.1	113.3	0.2615	49.993	0.939

SFC analysis of the major diastereomer of enantioenriched material, prepared using (R)-SEGPHOS:

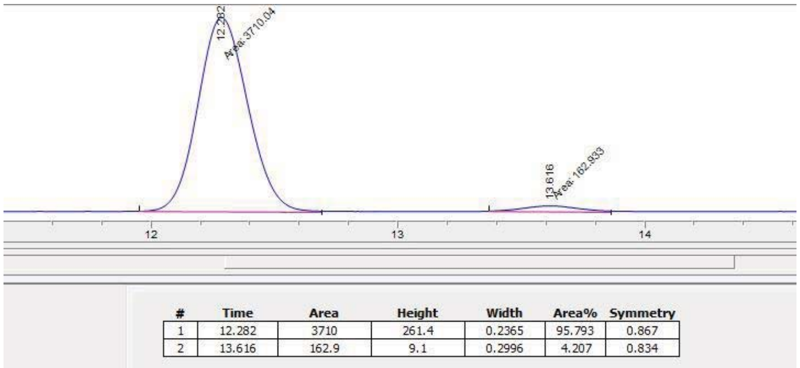

#	Time	Area	Height	Width	Area%	Symmetry
1	12.282	3710	261.4	0.2365	95.793	0.867
2	13.616	162.9	9.1	0.2996	4.207	0.834

(*2S,3S*)-*N,N*-Diethyl-3-phenyl-2-(phenylamino)butanamide (3ha):

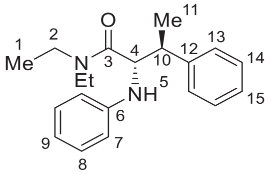

General procedure C: The reaction was carried out with substrate **1h** (20.6 mg, 0.10 mmol, 100 mol%) and styrene (23.0 μL, 0.20 mmol, 200 mol%). Purification of the residue by FCC (hexane/EtOAc 65:35) afforded the title compound (26.1 mg, 84%, >30:1 B:L, d.r. >20:1 *a:b*, e.r. = 98:2) as a colorless solid. ^{1}H NMR analysis of the crude material gave >30:1 B:L and d.r. = 5:1. **m.p.** = 60 – 62 °C (hexane/EtOAc); $[\boldsymbol{\alpha}]_{\boldsymbol{D}}^{\mathbf{25}}$ = -42.2 (c = 1.0, $CHCl_3$); **IR (thin film)** ν_{max}/cm^{-1}: 3334 (br), 2973 (s), 2933 (s), 1631 (s), 1601 (s), 1496 (s), 750 (s), 699 (s); **^{1}H NMR** (500 MHz, $CDCl_3$) Data for the major diastereomer *a* only: δ 7.33 – 7.23 (m, 5H, H^{13} + H^{14} + H^{15}), 7.17 – 7.14 (m, 2H, H^8), 6.72 – 6.69 (m, 1H, H^9), 6.63 (d, *J* = 7.5 Hz, 2H, H^7), 4.60 – 4.32 (m, 2H, H^4 + H^5), 3.68 – 3.61 (m, 1H, H^2), 3.36 – 3.30 (m, 1H, H^{10}), 3.12 – 3.03 (m, 2H, $H^{2'}$), 2.94 – 2.86 (m, 1H, H^2), 1.41 (d, *J* = 7.0 Hz, 3H, H^{11}), 1.12 (t, *J* = 7.0 Hz, 3H, H^1), 1.02 (t, *J* = 7.0 Hz, 3H, $H^{1'}$); **^{13}C NMR** (126 MHz, $CDCl_3$) Data for the major diastereomer *a* only: δ 170.7 (C^3), 147.1 (C^6), 142.6 (C^{12}), 129.2 (C^8), 128.3 (C^{14}), 128.1 (C^{13}), 126.9 (C^{15}), 117.8 (C^9), 114.0 (C^7), 58.5 (C^4), 42.6 (C^{10}), 41.4 (C^2), 40.3 ($C^{2'}$), 16.1 (C^{11}), 14.3 (C^1), 12.8 ($C^{1'}$); **HRMS** (ESl): calculated for $C_{20}H_{26}N_2ONa$ $[M+Na]^+$ requires *m/z* 333.1937, found *m/z* 333.1948; **Chiral SFC:** DAICEL CHIRALCEL IE column (25 cm), CO_2:*i*-PrOH 90:10, 2., 165 bar, 40 °C. Retention times: 17.6 mins (minor), 21.2 mins (major), e.r. = 98:2. To facilitate analysis by SFC, the major diastereoisomer was separated from the minor diastereoisomer by FCC.

SFC analysis of the major diastereomer of the racemate, prepared using rac-BINAP:

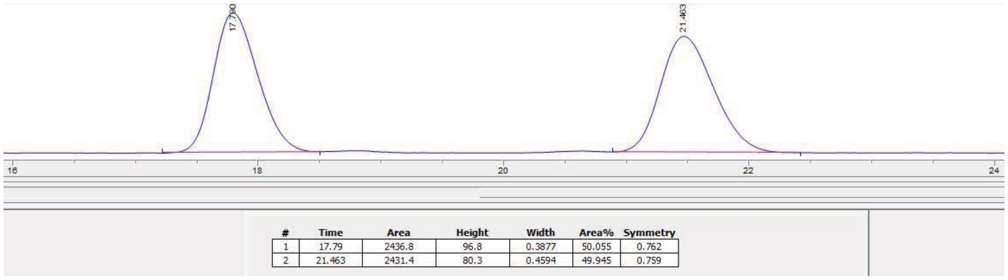

#	Time	Area	Height	Width	Area%	Symmetry
1	17.79	2436.8	96.8	0.3877	50.055	0.762
2	21.463	2431.4	80.3	0.4594	49.945	0.759

SFC analysis of the major diastereomer of enantioenriched material, prepared using (R)-SEGPHS:

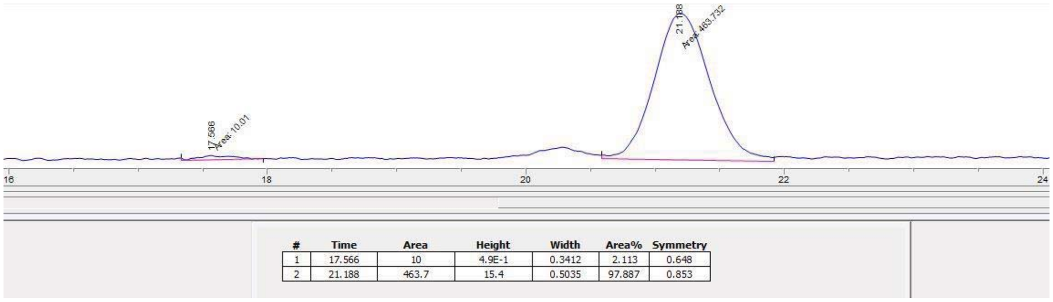

#	Time	Area	Height	Width	Area%	Symmetry
1	17.566	10	4.9E-1	0.3412	2.113	0.648
2	21.188	463.7	15.4	0.5035	97.887	0.853

(*2S,3S*)-3-Phenyl-2-(phenylamino)-1-(pyrrolidin-1-yl)butan-1-one (3ia):

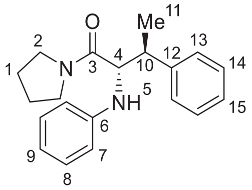

General procedure C: The reaction was carried out with substrate **1i** (20.4 mg, 0.10 mmol, 100 mol%) and styrene (23.0 μL, 0.20 mmol, 200 mol%). Purification of the residue by FCC (hexane/EtOAc 60:40) afforded the title compound (21.3 mg, 69%, >30:1 B:L, d.r. >20:1 *a:b*, e.r. = 98:2) as a colorless solid. ^{1}H NMR analysis of the crude material gave >30:1 B:L and d.r. = 9:1. **m.p.** = 115 – 117 °C (hexane/EtOAc); $[\boldsymbol{\alpha}]_{\boldsymbol{D}}^{\mathbf{25}}$ = -34.3 (c = 1.0 $CHCl_3$); **IR (thin film)** ν_{max}/cm^{-1}: 3335 (br), 3006 (s), 2989 (s), 1631 (s), 1602 (s), 1261 (s), 766 (s); **^{1}H NMR** (500 MHz, $CDCl_3$) Data for the major diastereomer *a* only: δ 7.32 – 7.21 (m, 5H, H^{13} + H^{14} + H^{15}), 7.17 – 7.14 (m, 2H, H^8), 6.73 – 6.70 (m, 1H, H^9), 6.65 (d, *J* = 8.0 Hz, 2H, H^7), 4.78 (br s, 1H, H^5), 4.36 (d, *J* = 6.0 Hz, 1H, H^4), 3.49 – 3.44 (m, 1H, H^2), 3.41 – 3.34 (m, 2H, H^2 + H^{10}), 3.25 – 3.20 (m, 1H, $H^{2'}$), 2.59 – 2.55 (m, 1H, $H^{2'}$), 1.77 – 1.56 (m, 4H, H^1), 1.39 (d, *J* = 7.0 Hz, 3H, H^{11}); **^{13}C NMR** (126 MHz, $CDCl_3$) Data for the major diastereomer *a* only: δ 169.7 (C^3), 146.6 (C^6), 142.7 (C^{12}), 129.3 (C^8), 128.4 (C^{14}), 127.9 (C^{13}), 126.9 (C^{15}), 118.0 (C^9), 113.9 (C^7), 60.4

(C^4), 46.3 (C^2), 45.8 ($C^{2'}$), 42.0 (C^{10}), 25.6 (C^1), 23.9 ($C^{1'}$), 15.4 (C^{11}); **HRMS** (ESI): calculated for $C_{20}H_{24}N_2ONa$ $[M+Na]^+$ requires *m/z* 331.1781, found *m/z* 331.1788; **Chiral SFC:** YMC Chiral ART Cellulose-SB column (25 cm), CO_2:*i*-PrOH 95:5, 2.0 mL/min, 140 bar, 40 °C. Retention times: 25.3 mins (minor), 28.2 mins (major), e.r. = 98:2. To facilitate analysis by SFC, the major diastereoisomer was separated from the minor diastereoisomer by FCC.

SFC analysis of the major diastereomer of the racemate, prepared using rac-BINAP:

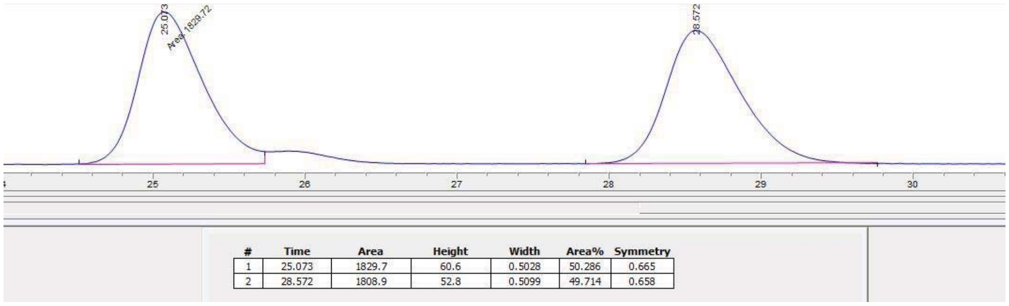

#	Time	Area	Height	Width	Area%	Symmetry
1	25.073	1829.7	60.6	0.5028	50.286	0.665
2	28.572	1808.9	52.8	0.5099	49.714	0.658

SFC analysis of the major diastereomer of enantioenriched material, prepared using (R)-SEGPHOS:

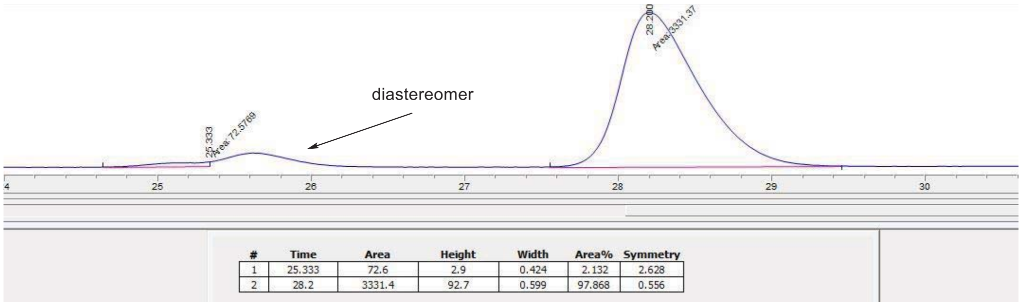

#	Time	Area	Height	Width	Area%	Symmetry
1	25.333	72.6	2.9	0.424	2.132	2.628
2	28.2	3331.4	92.7	0.599	97.868	0.556

(*2S*,*3S*)-3-Phenyl-2-(phenylamino)-1-(piperidin-1-yl)butan-1-one (3ja):

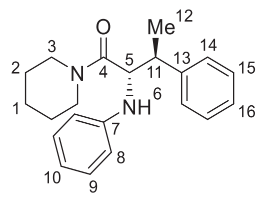

General procedure C: The reaction was carried out with substrate **1j** (21.8 mg, 0.10 mmol, 100 mol%) and styrene (23.0 µL, 0.20 mmol, 200 mol%). Purification of the residue by FCC (hexane/EtOAc 60:40) afforded the title compound (25.1 mg, 78%, >30:1 B:L, d.r. >20:1 *a:b*, e.r. = 94.5:5.5) as a pale-yellow oil. ^{1}H NMR analysis of the crude material gave >30:1 B:L and d.r. = 4:1. $[\alpha]_D^{25}$ = -3.2 (c = 1.0 $CHCl_3$); **IR (thin film)** ν_{max}/cm^{-1}: 3336 (br), 2935 (s), 2855 (s), 1627 (s), 1601 (s), 1495 (s), 1249 (s), 793 (s); **^{1}H NMR** (500 MHz, $CDCl_3$) Data for the

major diastereomer *a* only: δ 7.35 – 7.17 (m, 5H, H^{14} + H^{15} + H^{16}), 7.19 – 7.15 (m, 2H, H^{9}), 6.74 – 6.71 (m, 1H, H^{10}), 6.65 (d, J = 7.5 Hz, 2H, H^{8}), 4.74 – 4.39 (m, 2H, H^{5} + H^{6}), 3.70 – 3.66 (m, 1H, H^{3}), 3.45 – 3.29 (m, 3H, H^{3} + H^{11}), 3.12 – 3.07 (m, 1H, H^{3}), 1.65 – 1.46 (m, 5H, H^{1}), 1.41 (d, J = 7.0 Hz, 3H, H^{12}), 1.38 – 1.32 (m, 1H, H^{1}); **^{13}C NMR** (126 MHz, $CDCl_3$) Data for the major diastereomer *a* only: δ 170.0 (C^{4}), 147.2 (C^{7}), 142.4 (C^{13}), 129.2 (C^{9}), 128.4 (C^{15}), 128.0 (C^{14}), 126.8 (C^{16}), 117.9 (C^{10}), 113.9 (C^{8}), 58.1 (C^{5}), 46.5 (C^{3}), 43.1 ($C^{3'}$), 42.4 (C^{11}), 26.2 (C^{2}), 25.5 ($C^{2'}$), 24.4 (C^{1}), 16.7 (C^{12}); **HRMS** (ESI): calculated for $C_{21}H_{26}N_2ONa$ $[M+Na]^+$ requires *m/z* 345.1937, found *m/z* 345.1951; **Chiral SFC:** DAICEL CHIRALCEL OD-H column (25 cm), CO_2:*i*-PrOH 90:10, 2.0 mL/min, 140 bar, 40 °C. Retention times: 6.5 mins (minor), 8.2 mins (major), e.r. = 94.5:5.5. To facilitate analysis by SFC, the major diastereoisomer was separated from the minor diastereoisomer by FCC.

SFC analysis of the major diastereomer of the racemate, prepared using rac-BINAP:

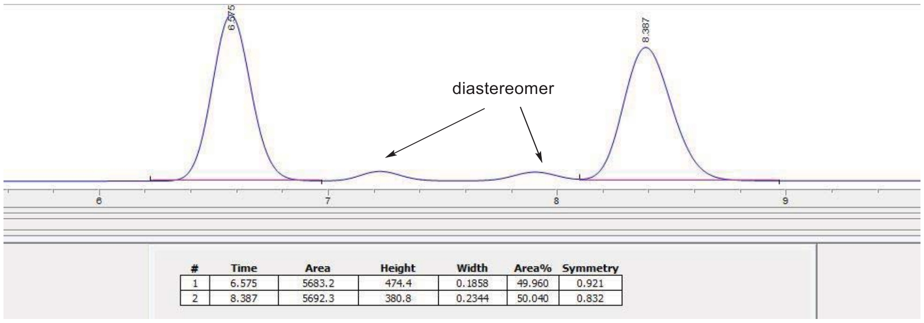

#	Time	Area	Height	Width	Area%	Symmetry
1	6.575	5683.2	474.4	0.1858	49.960	0.921
2	8.387	5692.3	380.8	0.2344	50.040	0.832

SFC analysis of the major diastereomer of enantioenriched material, prepared using (R)-SEGPHOS:

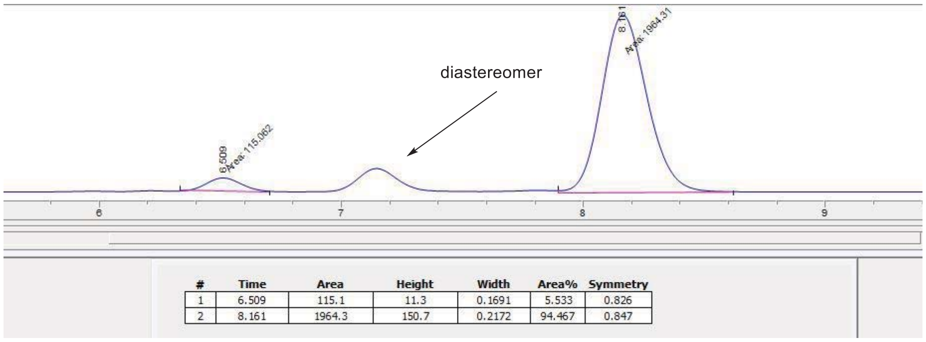

#	Time	Area	Height	Width	Area%	Symmetry
1	6.509	115.1	11.3	0.1691	5.533	0.826
2	8.161	1964.3	150.7	0.2172	94.467	0.847

(*2S,3S*)-1-Morpholino-3-phenyl-2-(phenylamino)butan-1-one (3ka):

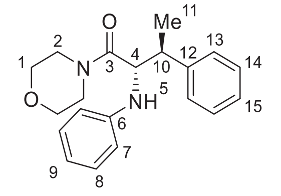

General procedure C: The reaction was carried out with substrate **1k** (22.0 mg, 0.10 mmol, 100 mol%) and styrene (23.0 µL, 0.20 mmol, 200 mol%). Purification of the residue by FCC (hexane/EtOAc 60:40) afforded the title compound (22.0 mg, 68%, >30:1 B:L, d.r. >20:1 *a:b*, e.r. = 98:2) as a pale-yellow oil. ^{1}H NMR analysis of the crude material gave >30:1 B:L and d.r. = 5:1. $[\boldsymbol{\alpha}]_{\boldsymbol{D}}^{\mathbf{25}}$ = -15.9 (c = 1.0 $CHCl_3$); **IR (thin film)** ν_{max}/cm^{-1}: 3344 (br), 2922 (s), 2854 (s), 1635 (s), 1601 (s), 1495 (s), 1423 (s), 1113 (s); **^{1}H NMR** (500 MHz, $CDCl_3$) Data for the major diastereomer *a* only: δ 7.35 – 7.24 (m, 5H, H^{13} + H^{14} + H^{15}), 7.18 – 7.15 (m, 2H, H^8), 6.74 – 6.71 (m, 1H, H^9), 6.64 (d, *J* = 7.5 Hz, 2H, H^7), 4.56 – 4.47 (m, 2H, H^4 + H^5), 3.63 – 3.50 (m, 4H, H^1), 3.43 – 3.26 (m, 3H, H^2 + H^{10}), 3.22 – 3.18 (m, 1H, $H^{2'}$), 3.00 – 2.95 (m, 1H, $H^{2'}$), 1.39 (d, *J* = 7.0 Hz, 3H, H^{11}); **^{13}C NMR** (126 MHz, $CDCl_3$) Data for the major diastereomer *a* only: δ 170.2 (C^3), 146.7 (C^6), 142.2 (C^{12}), 129.4 (C^8), 128.6 (C^{14}), 128.0 (C^{13}), 127.1 (C^{15}), 118.3 (C^9), 113.9 (C^7), 66.7 (C^1), 66.1 ($C^{1'}$), 57.9 (C^4), 46.0 (C^2), 42.3 ($C^{2'}$), 42.0 (C^{10}), 15.7 (C^{11}); **HRMS** (ESI): calculated for $C_{20}H_{24}N_2O_2Na$ $[M+Na]^+$ requires *m/z* 347.1730, found *m/z* 347.1742; **Chiral SFC:** DAICEL CHIRALCEL OD-H column (25 cm), CO_2:*i*-PrOH 90:10, 2.0 mL/min, 140 bar, 40 °C. Retention times: 8.7 mins (minor), 10.9 mins (major), e.r. = 98:2. To facilitate analysis by SFC, the major diastereoisomer was separated from the minor diastereoisomer by FCC.

SFC analysis of the major diastereomer of the racemate, prepared using rac-BINAP:

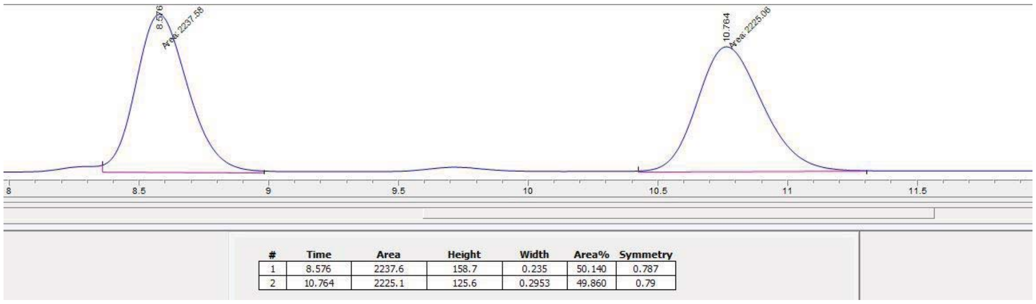

#	Time	Area	Height	Width	Area%	Symmetry
1	8.576	2237.6	158.7	0.235	50.140	0.787
2	10.764	2225.1	125.6	0.2953	49.860	0.79

SFC analysis of the major diastereomer of enantioenriched material, prepared using (R)-SEGPHOS:

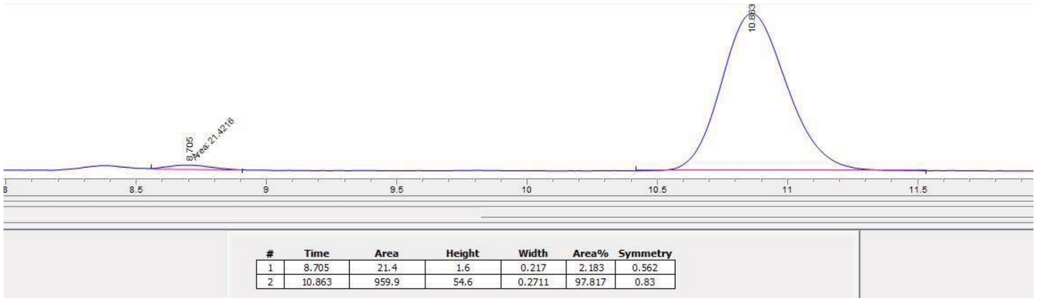

#	Time	Area	Height	Width	Area%	Symmetry
1	8.705	21.4	1.6	0.217	2.183	0.562
2	10.863	959.9	54.6	0.2711	97.817	0.83

(*2S,3S*)-2-((4-Hydroxyphenyl)amino)-*N*,3-diphenylbutanamide (3la):

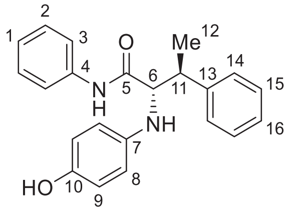

General procedure C: The reaction was carried out with substrate **1l** (24.2 mg, 0.10 mmol, 100 mol%) and styrene (23.0 µL, 0.20 mmol, 200 mol%). Purification of the residue by FCC (hexane/EtOAc 65:35) afforded the title compound (23.9 mg, 69%, >30:1 B:L, d.r. >20:1 *a:b*, e.r. = 91:9) as a pale-yellow oil. ^{1}H NMR analysis of the crude material gave >30:1 B:L and d.r. = 4:1. $[\boldsymbol{\alpha}]_{\boldsymbol{D}}^{\mathbf{25}}$ = -27.4 (c = 1.0 $CHCl_3$); **IR (thin film)** ν_{max}/cm^{-1}: 3320 (br), 2928 (s), 1669 (s), 1599 (s), 1512 (s), 1444 (s), 1238 (s), 756 (s), 702 (s); **^{1}H NMR** (500 MHz, CD_3OD-*d*^{4}) Data for the major diastereomer *a* only: δ 7.47 (d, *J* = 8.0 Hz, 2H, H^3), 7.32 – 7.18 (m, 7H, H^2 + H^{14} + H^{15} + H^{16}), 7.11 – 7.08 (m, 1H, H^1), 6.58 (d, *J* = 8.5 Hz, 2H, H^9), 6.49 (d, *J* = 8.5 Hz, 2H, H^8), 3.97 (d, *J* = 8.0 Hz, 1H, H^6), 3.27 – 3.21 (m, 1H, H^{11}), 1.39 (d, *J* = 7.0 Hz, 3H, H^{12}); **^{13}C NMR** (126 MHz, CD_3OD-*d*^{4}) Data for the major diastereomer *a* only: δ 173.5 (C^5), 149.7 (C^{10}), 142.8 (C^7), 140.5 (C^{13}), 137.7 (C^4), 128.4 (C^2), 128.3 (C^{15}), 127.4 (C^{14}), 126.6 (C^{16}), 124.2 (C^1), 120.3 (C^3), 115.5 (C^9), 115.2 (C^8), 66.0 (C^6), 43.1 (C^{11}), 18.0 (C^{12}); **HRMS** (ESI): calculated for $C_{22}H_{22}N_2O_2Na$ $[M+Na]^+$ requires *m/z* 369.1573, found *m/z* 369.1583; **Chiral SFC:** DAICEL CHIRALCEL IE column (25 cm), CO_2:*i*-PrOH (with 0.5% TEA) 70:30, 2.0 mL/min, 190 bar, 40 °C. Retention times: 4.9 mins (major), 5.8 mins (minor), e.r. = 91:9. To facilitate analysis by SFC, the major diastereoisomer was separated from the minor diastereoisomer by FCC.

SFC analysis of the major diastereomer of the racemate, prepared using rac-BINAP:

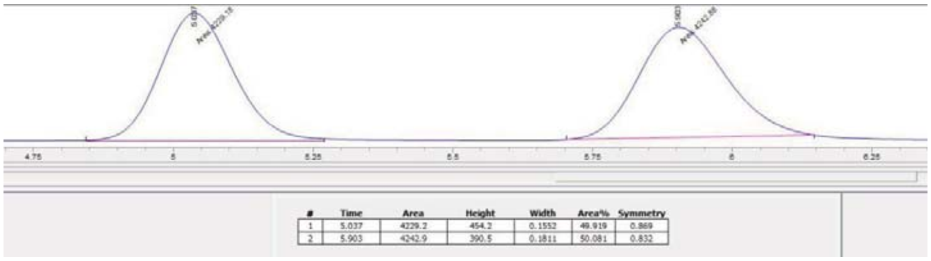

#	Time	Area	Height	Width	Area%	Symmetry
1	5.037	4229.2	454.2	0.1552	49.919	0.869
2	5.903	4242.9	390.5	0.1811	50.081	0.832

SFC analysis of the major diastereomer of enantioenriched material, prepared using (R)-SEGPHOS:

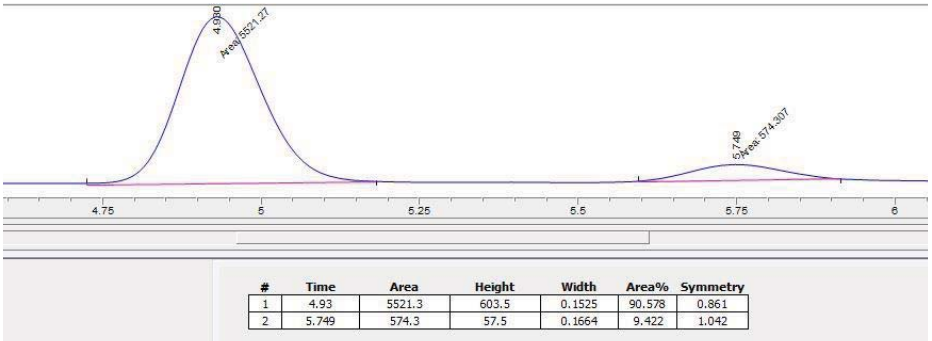

#	Time	Area	Height	Width	Area%	Symmetry
1	4.93	5521.3	603.5	0.1525	90.578	0.861
2	5.749	574.3	57.5	0.1664	9.422	1.042

(*2S,3S*)-1,3-Diphenyl-2-(phenylamino)butan-1-one (3ma):

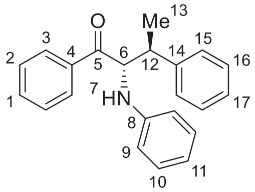

General procedure C: The reaction was carried out with substrate **1m** (21.1 mg, 0.10 mmol, 100 mol%), styrene (23.0 µL, 0.20 mmol, 200 mol%) and (*R*)-3,5-(*t*-Bu)$_2$-H$_8$-BINAP (5.40 mg, 5.00 µmol, 5 mol%) at 90 ºC. Purification of the residue by FCC (hexane/EtOAc 95:5) afforded the title compound (26.8 mg, 85%, >30:1 B:L, d.r. >20:1 *a:b*, e.r. = 71:29) as a pale-yellow oil. ^{1}H NMR analysis of the crude material gave >30:1 B:L and d.r. = 8:1. $[\boldsymbol{\alpha}]_{\boldsymbol{D}}^{\mathbf{25}}$ = +28.6 (c = 1.0 CHCl$_3$); **IR (thin film)** ν_{max}/cm^{-1}: 3379 (br), 3027 (s), 1682 (s), 1601 (s), 1506 (s), 748 (s), 693 (s); **^{1}H NMR** (500 MHz, CD$_2$Cl$_2$) Data for the major diastereomer *a* only: δ 7.93 (d, *J* = 7.5 Hz, 2H, H^3), 7.61 – 7.58 (m, 1H, H^1), 7.49 – 7.46 (m, 2H, H^2), 7.26 – 7.18 (m, 3H, H^{16} + H^{17}), 7.12 – 7.08 (m, 4H, H^{10} + H^{15}), 6.68 – 6.65 (m, 3H, H^9 + H^{11}), 5.19 (d, *J* = 5.0 Hz, 1H, H^6), 4.37 (br s, 1H, H^7), 3.45 – 3.40 (m, 1H, H^{12}), 1.44 (d, *J* = 7.0 Hz, 3H, H^{13}); **^{13}C NMR** (126 MHz, CD$_2$Cl$_2$) Data for the major diastereomer *a* only: δ 200.4 (C^5), 147.8 (C^8), 141.4 (C^{14}), 136.3 (C^4), 133.5 (C^1), 129.3 (C^{10}), 128.9 (C^3), 128.4 (C^{16}), 128.3 (C^2), 128.2 (C^{15}), 127.0 (C^{17}), 118.2 (C^{11}), 114.0 (C^9), 63.5 (C^6), 42.9 (C^{12}), 18.2 (C^{13}); **HRMS** (ESl): calculated for

$C_{22}H_{21}NONa$ $[M+Na]^+$ requires *m/z* 338.1515, found *m/z* 338.1524; **Chiral SFC:** DAICEL CHIRALCEL IE column (25 cm), CO_2:*i*-PrOH 95:5, 1.0 mL/min, 140 bar, 40 °C. Retention times: 42.8 mins (minor), 53.1 mins (major), e.r. = 71:29. To facilitate analysis by SFC, the major diastereoisomer was separated from the minor diastereoisomer by FCC.

SFC analysis of the racemates, prepared using rac-BINAP:

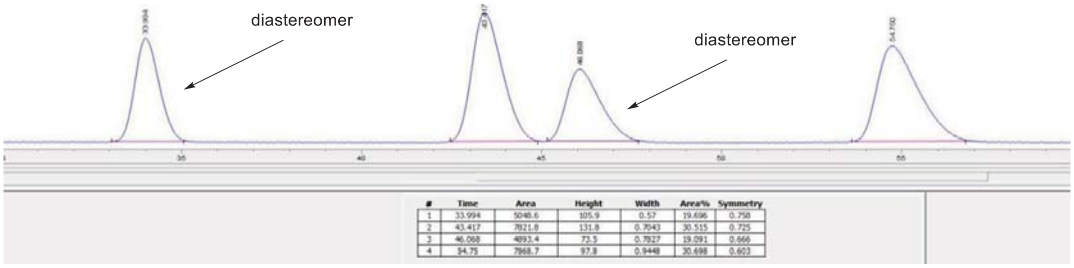

#	Time	Area	Height	Width	Area%	Symmetry
1	33.994	5048.6	105.9	0.57	19.696	0.758
2	43.417	7821.8	131.8	0.7043	30.515	0.725
3	46.068	4893.4	73.5	0.7827	19.091	0.666
4	54.75	7868.7	97.8	0.9448	30.698	0.603

SFC analysis of the major diastereomer of enantioenriched material, prepared using (R)-3,5-(t-Bu)$_2$-H$_8$-BINAP:

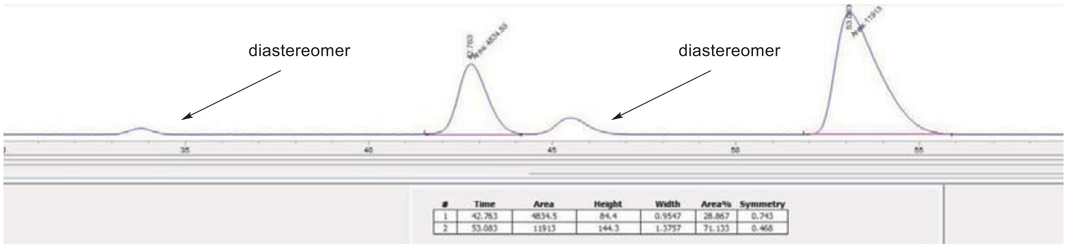

#	Time	Area	Height	Width	Area%	Symmetry
1	42.763	4834.5	84.4	0.9547	28.867	0.743
2	53.083	11913	144.3	1.3757	71.133	0.468

(*3S,4S*)-4-Phenyl-3-(phenylamino)pentan-2-one (3na):

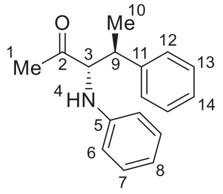

General procedure C: The reaction was carried out with substrate **1n** (14.9 mg, 0.10 mmol, 100 mol%), styrene (23.0 µL, 0.20 mmol, 200 mol%) and (*R*)-DTBM-SEGPHOS (5.90 mg, 5.00 µmol, 5 mol%) and was run for 22 hours at 90 ºC. Purification of the residue by FCC (hexane/EtOAc 85:15) afforded the title compound (17.7 mg, 70%, >30:1 B:L, d.r. = 9:1 *a:b*, e.r. = 90:10) as a pale-yellow oil. ^{1}H NMR analysis of the crude material gave >30:1 B:L and d.r. = 2:1. $[\boldsymbol{\alpha}]_D^{25}$ = -1.5 (c = 1.0 $CHCl_3$); **IR (thin film)** ν_{max}/cm^{-1}: 3378 (br), 2934 (s), 1709 (s), 1603 (s), 1506 (s), 751 (s), 701 (s); **^{1}H NMR** (500 MHz, $CDCl_3$) Data for the major diastereomer *a*: δ 7.39 – 7.27 (m, 5H, $H^{12} + H^{13} + H^{14}$), 7.17 – 7.14 (m, 2H, H^7), 6.75 – 6.72 (m, 1H, H^8), 6.54 (d, *J* = 7.5 Hz, 2H, H^6), 4.19 – 3.89 (m, 2H, $H^3 + H^4$), 3.30 – 3.24 (m, 1H,

H[9]), 2.06 (s, 3H, H[1]), 1.37 (d, J = 7.0 Hz, 3H, H[10]). Characteristic signals for the minor diastereomer *b*: 6.47 (d, J = 7.5 Hz, 2H, H[6]), 1.99 (s, 3H, H[1]), 1.42 (d, J = 7.0 Hz, 3H, H[10]); **^{13}C NMR** (126 MHz, $CDCl_3$) Data for the major diastereomer *a* only: δ 211.3 (C[2]), 146.9 (C[5]), 141.5 (C[11]), 129.4 (C[7]), 128.9 (C[13]), 127.6 (C[12]), 127.4 (C[14]), 118.3 (C[8]), 113.2 (C[6]), 69.2 (C[3]), 41.5 (C[9]), 26.7 (C[1]), 18.2 (C[10]); **HRMS** (ESI): calculated for $C_{17}H_{19}NONa$ $[M+Na]^+$ requires *m/z* 276.1359, found *m/z* 276.1361; **Chiral SFC:** DAICEL CHIRALCEL IE column (25 cm), CO_2:*i*-PrOH 95:5, 1.0 mL/min, 140 bar, 40 °C. Retention times: 12.9 mins (major), 14.2 mins (minor), e.r. = 90:10. To facilitate analysis by SFC, the major diastereoisomer was separated from the minor diastereoisomer by FCC.

SFC analysis of the major diastereomer of the racemate, prepared using rac-BINAP:

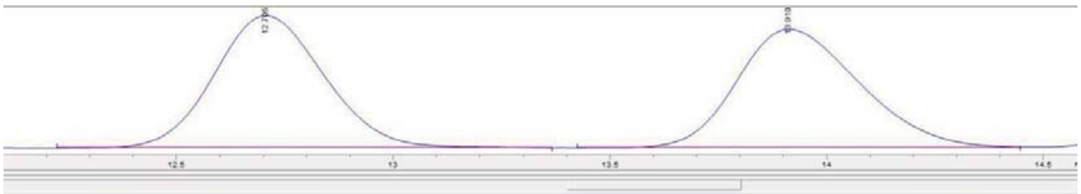

#	Time	Area	Height	Width	Area%	Symmetry
1	12.705	4168.4	235.4	0.2746	50.138	0.858
2	13.91	4145.5	210.7	0.3075	49.862	0.716

SFC analysis of the major diastereomer of enantioenriched material, prepared using (R)-DTBM-SEGPHOS:

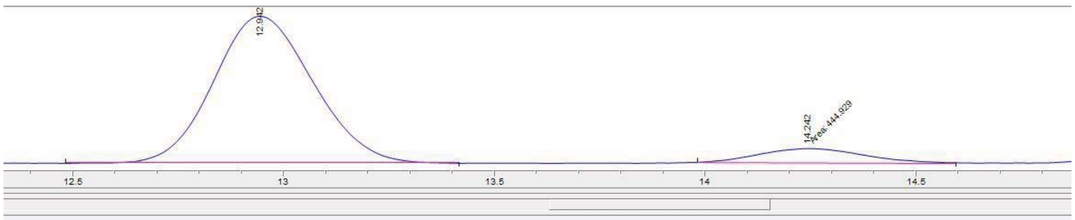

#	Time	Area	Height	Width	Area%	Symmetry
1	12.942	4073.8	247	0.2544	90.154	0.859
2	14.242	444.9	24.8	0.2984	9.846	0.842

(*4S,5S*)-2,2-Dimethyl-5-phenyl-4-(phenylamino)hexan-3-one (3oa):

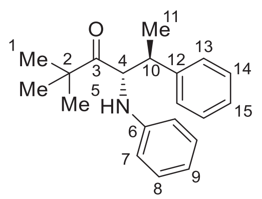

General procedure C: The reaction was carried out with substrate **1o** (19.1 mg, 0.10 mmol, 100 mol%), styrene (23.0 μL, 0.20 mmol, 200 mol%) and (*R*)-DTBM-SEGPHOS (5.90 mg, 5.00 μmol, 5 mol%). Purification of the residue by FCC (hexane/EtOAc 90:10) afforded the

title compound (21.3 mg, 72%, >30:1 B:L, d.r. >20:1 *a*:*b*, e.r. = 74.5:25.5) as a pale-yellow oil. ^{1}H NMR analysis of the crude material gave >30:1 B:L and d.r. = 2:1. $[\boldsymbol{\alpha}]_{\boldsymbol{D}}^{\boldsymbol{25}}$ = +51.5 (c = 1.0 $CHCl_3$); **IR (thin film)** ν_{max}/cm^{-1}: 3338 (br), 2974 (s), 1698 (s), 1601 (s), 1513 (s), 1314 (s), 1060 (s), 699 (s); **^{1}H NMR** (500 MHz, $CDCl_3$) Data for the major diastereomer *a* only: δ 7.34 – 7.21 (m, 5H, H^{13} + H^{14} + H^{15}), 7.17 – 7.14 (m, 2H, H^8), 6.73 – 6.70 (m, 1H, H^9), 6.59 (d, *J* = 8.0 Hz, 2H, H^7), 4.59 (d, *J* = 8.0 Hz, 1H, H^4), 4.08 (br s, 1H, H^5), 3.31 – 3.25 (m, 1H, H^{10}), 1.39 (d, *J* = 7.0 Hz, 3H, H^{11}), 0.87 (s, 9H, H^1); **^{13}C NMR** (126 MHz, $CDCl_3$) Data for the major diastereomer *a* only: δ 215.5 (C^3), 147.3 (C^6), 143.3 (C^{12}), 129.3 (C^8), 128.5 (C^{14}), 128.2 (C^{13}), 126.8 (C^{15}), 118.0 (C^9), 113.6 (C^7), 62.3 (C^4), 43.4 (C^2), 42.7 (C^{10}), 25.9 (C^1), 16.3 (C^{11}); **HRMS** (ESI): calculated for $C_{20}H_{26}NO$ $[M+H]^+$ requires *m/z* 296.2009, found *m/z* 296.2016; **Chiral SFC:** DAICEL CHIRALCEL IE column (25 cm), CO_2:*i*-PrOH 95:5, 1.0 mL/min, 140 bar, 40 °C. Retention times: 9.8 mins (major), 10.8 mins (minor), e.r. = 74.5:25.5. To facilitate analysis by SFC, the major diastereoisomer was separated from the minor diastereoisomer by FCC.

SFC analysis of the major diastereomer of racemate, prepared using rac-BINAP:

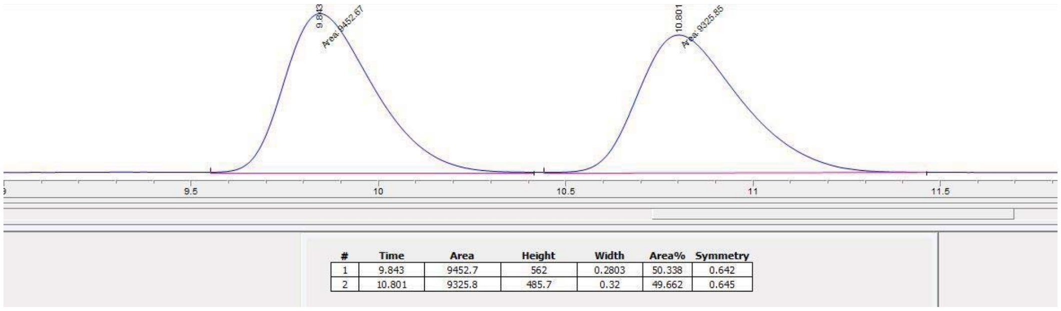

#	Time	Area	Height	Width	Area%	Symmetry
1	9.843	9452.7	562	0.2803	50.338	0.642
2	10.801	9325.8	485.7	0.32	49.662	0.645

SFC analysis of the major diastereomer of enantioenriched material, prepared using (R)-DTBM-SEGPHOS:

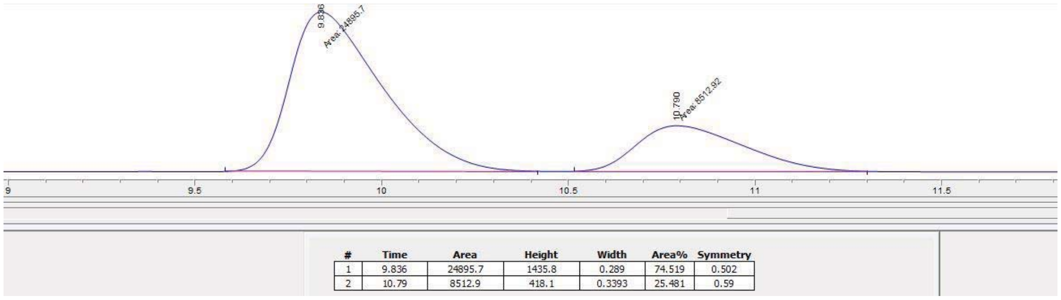

#	Time	Area	Height	Width	Area%	Symmetry
1	9.836	24895.7	1435.8	0.289	74.519	0.502
2	10.79	8512.9	418.1	0.3393	25.481	0.59

(*2S,3S*)-2-((4-Hydroxyphenyl)amino)-*N,N*-dimethyl-3-phenylbutanamide (3pa):

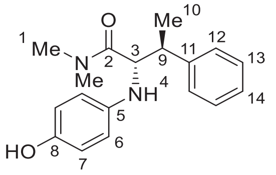

General procedure C: The reaction was carried out with substrate **1p** (19.4 mg, 0.10 mmol, 100 mol%) and styrene (23.0 μL, 0.20 mmol, 200 mol%). Purification of the residue by FCC (hexane/EtOAc 65:35) afforded the title compound (25.9 mg, 87%, >30:1 B:L, d.r. >20:1 *a:b*, e.r. = 98:2) as a pale-yellow oil. ^{1}H NMR analysis of the crude material gave >30:1 B:L and d.r. = 6:1. $[\boldsymbol{\alpha}]_{\boldsymbol{D}}^{\mathbf{25}}$ = -11.8 (c = 1.0 $CHCl_3$); **IR (thin film)** ν_{max}/cm^{-1}: 3331 (br), 3027 (s), 2968 (s), 1623 (s), 1514 (s), 1240 (s), 700 (s); **^{1}H NMR** (500 MHz, $CDCl_3$) Data for the major diastereomer *a* only: δ 7.34 – 7.23 (m, 5H, $H^{12} + H^{13} + H^{14}$), 6.64 (d, *J* = 8.0 Hz, 2H, H^7), 6.50 (d, *J* = 8.0 Hz, 2H, H^6), 4.60 – 3.61 (m, 2H, $H^3 + H^4$), 3.30 – 3.24 (m, 1H, H^9), 2.89 (s, 3H, H^1), 2.72 (s, 3H, $H^{1'}$), 1.38 (d, *J* = 7.0 Hz, 3H, H^{10}); **^{13}C NMR** (126 MHz, $CDCl_3$) Data for the major diastereomer *a* only: δ 172.7 (C^2), 149.4 (C^8), 142.6 (C^5), 140.2 (C^{11}), 128.4 (C^{13}), 127.8 (C^{12}), 126.8 (C^{14}), 116.4 (C^7), 116.2 (C^6), 60.3 (C^3), 42.5 (C^9), 37.0 (C^1), 35.8 ($C^{1'}$), 16.3 (C^{10}); **HRMS** (ESI): calculated for $C_{18}H_{22}N_2O_2Na$ $[M+Na]^+$ requires *m/z* 321.1573, found *m/z* 321.1583; **Chiral SFC:** DAICEL CHIRALCEL OD-H column (25 cm), CO_2:*i*-PrOH 85:15, 2.0 mL/min, 160 bar, 40 °C. Retention times: 10.2 mins (major), 11.2 mins (minor), e.r. = 98:2. To facilitate analysis by SFC, the major diastereoisomer was separated from the minor diastereoisomer by FCC.

SFC analysis of the major diastereomer of racemate, prepared using rac-BINAP:

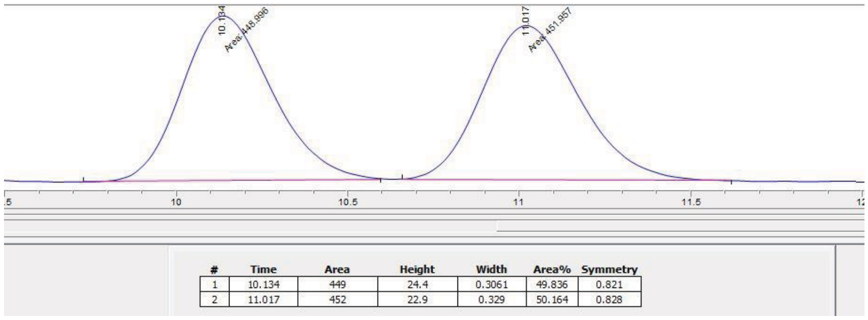

#	Time	Area	Height	Width	Area%	Symmetry
1	10.134	449	24.4	0.3061	49.836	0.821
2	11.017	452	22.9	0.329	50.164	0.828

SFC analysis of the major diastereomer of enantioenriched material, prepared using (R)-SEGPHOS:

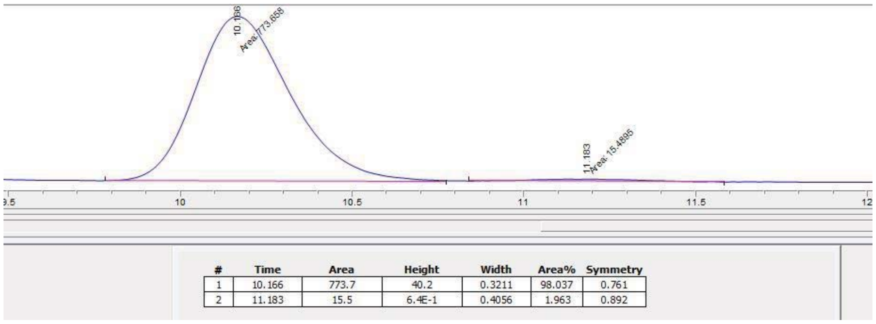

#	Time	Area	Height	Width	Area%	Symmetry
1	10.166	773.7	40.2	0.3211	98.037	0.761
2	11.183	15.5	6.4E-1	0.4056	1.963	0.892

(*2S,3S*)-2-((4-Methoxyphenyl)amino)-*N,N*-dimethyl-3-phenylbutanamide (3qa):

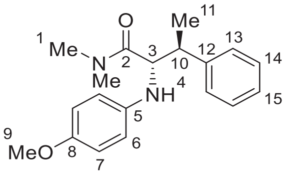

General procedure C: The reaction was carried out with substrate **1q** (20.8 mg, 0.10 mmol, 100 mol%) and styrene (23.0 µL, 0.20 mmol, 200 mol%). Purification of the residue by FCC (hexane/EtOAc 65:35) afforded the title compound (17.2 mg, 55%, >30:1 B:L, d.r. >20:1 *a:b*, e.r. = 90.5:9.5) as a pale-yellow oil. ^{1}H NMR analysis of the crude material gave >30:1 B:L and d.r. = 9:1. $[\boldsymbol{\alpha}]_{\boldsymbol{D}}^{\mathbf{25}}$ = -5.8 (c = 1.0 $CHCl_3$); **IR (thin film)** ν_{max}/cm^{-1}: 3334 (br), 2931 (s), 1637 (s), 1510 (s), 1236 (s), 701 (s); **^{1}H NMR** (500 MHz, $CDCl_3$) Data for the major diastereomer *a* only: δ 7.35 – 7.24 (m, 5H, H^{13} + H^{14} + H^{15}), 6.77 (d, *J* = 9.0 Hz, 2H, H^7), 6.62 (d, *J* = 9.0 Hz, 2H, H^6), 4.61 – 3.89 (m, 2H, H^3 + H^4), 3.75 (s, 3H, H^9), 3.34 – 3.28 (m, 1H, H^{10}), 2.91 (s, 3H, H^1), 2.68 (s, 3H, $H^{1'}$), 1.39 (d, *J* = 7.0 Hz, 3H, H^{11}); **^{13}C NMR** (126 MHz, $CDCl_3$) Data for the major diastereomer *a* only: δ 171.9 (C^2), 152.6 (C^8), 142.7 (C^5), 141.1 (C^{12}), 128.4 (C^{14}), 127.8 (C^{13}), 126.8 (C^{15}), 115.9 (C^7), 114.8 (C^6), 59.8 (C^3), 55.7 (C^9), 42.4 (C^{10}), 36.8 (C^1), 35.6 ($C^{1'}$), 16.0 (C^{11}); **HRMS** (ESI): calculated for $C_{19}H_{24}N_2O_2Na$ $[M+Na]^+$ requires *m/z* 335.1730, found *m/z* 335.1739; **Chiral SFC:** DAICEL CHIRALCEL OD-H column (25 cm), CO_2:*i*-PrOH 90:10, 2.0 mL/min, 140 bar, 40 °C. Retention times: 6.9 mins (minor), 8.3 mins (major), e.r. = 90.5:9.5. To facilitate analysis by SFC, the major diastereoisomer was separated from the minor diastereoisomer by FCC.

SFC analysis of the major diastereomer of racemate, prepared using rac-BINAP:

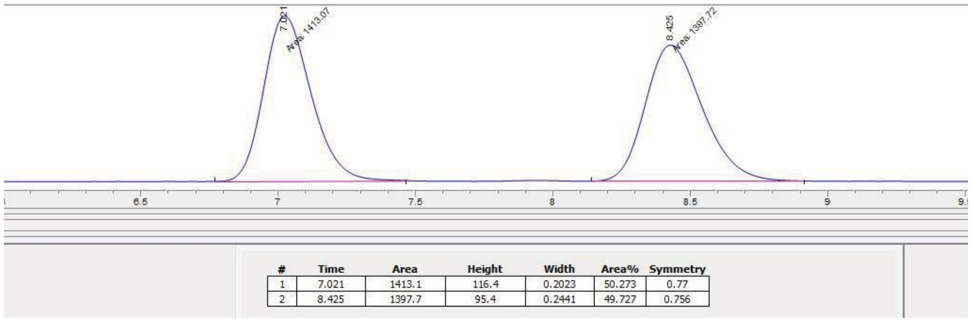

#	Time	Area	Height	Width	Area%	Symmetry
1	7.021	1413.1	116.4	0.2023	50.273	0.77
2	8.425	1397.7	95.4	0.2441	49.727	0.756

SFC analysis of the major diastereomer of enantioenriched material, prepared using (R)-SEGPHOS:

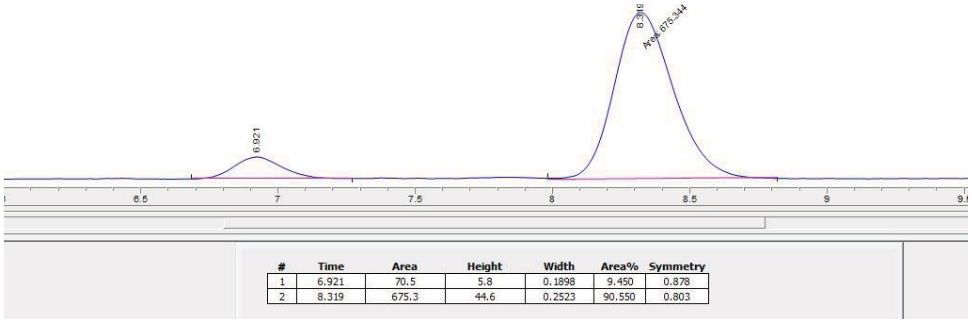

#	Time	Area	Height	Width	Area%	Symmetry
1	6.921	70.5	5.8	0.1898	9.450	0.878
2	8.319	675.3	44.6	0.2523	90.550	0.803

(*2S,3S*)-*N,N*-Dimethyl-3-phenyl-2-(*p*-tolylamino)butanamide (3ra):

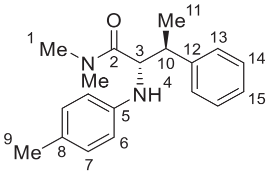

General procedure C: The reaction was carried out with substrate **1r** (19.2 mg, 0.10 mmol, 100 mol%) and styrene (23.0 μL, 0.20 mmol, 200 mol%). Purification of the residue by FCC (hexane/EtOAc 65:35) afforded the title compound (26.4 mg, 89%, >30:1 B:L, d.r. >20:1 *a:b*, e.r. = 97:3) as a pale-yellow solid. ^{1}H NMR analysis of the crude material gave >30:1 B:L and d.r. = 8:1. **m.p.** = 103 – 105 °C (hexane/EtOAc); $[\alpha]_D^{25}$ = -10.9 (c = 1.0 $CHCl_3$); **IR (thin film)** ν_{max}/cm^{-1}: 3333 (br), 2923 (s), 1639 (s), 1520 (s), 1396 (s), 1055 (s), 701 (s); **^{1}H NMR** (500 MHz, $CDCl_3$) Data for the major diastereomer *a* only: δ 7.35 – 7.24 (m, 5H, H^{13} + H^{14} + H^{15}), 6.99 (d, *J* = 8.5 Hz, 2H, H^{7}), 6.59 (d, *J* = 8.5 Hz, 2H, H^{6}), 4.71 – 4.31 (m, 2H, H^{3} + H^{4}), 3.38 – 3.32 (m, 1H, H^{10}), 2.91 (s, 3H, H^{1}), 2.66 (s, 3H, $H^{1'}$), 2.25 (s, 3H, H^{9}), 1.40 (d, *J* = 7.0 Hz, 3H, H^{11}); **^{13}C NMR** (126 MHz, $CDCl_3$) Data for the major diastereomer *a* only: δ 171.6 (C^{2}), 144.6 (C^{5}), 142.6 (C^{12}), 129.7 (C^{7}), 128.4 (C^{14}), 127.8 (C^{13}), 127.2 (C^{15}), 126.8 (C^{8}), 114.2 (C^{6}), 58.6 (C^{3}), 42.2 (C^{10}), 36.8 (C^{1}), 35.5 ($C^{1'}$), 20.3 (C^{9}), 15.8 (C^{11}); **HRMS** (ESI): calculated for

$C_{19}H_{24}N_2ONa$ $[M+Na]^+$ requires *m/z* 319.1781, found *m/z* 319.1795; **Chiral SFC:** DAICEL CHIRALCEL OD-H column (25 cm), CO_2:*i*-PrOH 90:10, 2.0 mL/min, 140 bar, 40 °C. Retention times: 5.8 mins (minor), 7.1 mins (major), e.r. = 97:3. To facilitate analysis by SFC, the major diastereoisomer was separated from the minor diastereoisomer by FCC.

SFC analysis of the major diastereomer of racemate, prepared using rac-BINAP:

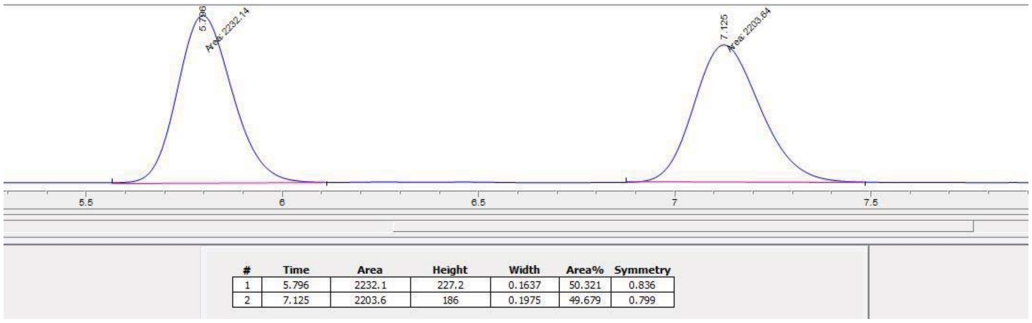

#	Time	Area	Height	Width	Area%	Symmetry
1	5.796	2232.1	227.2	0.1637	50.321	0.836
2	7.125	2203.6	186	0.1975	49.679	0.799

SFC analysis of the major diastereomer of enantioenriched material, prepared using (R)-SEGPHOS:

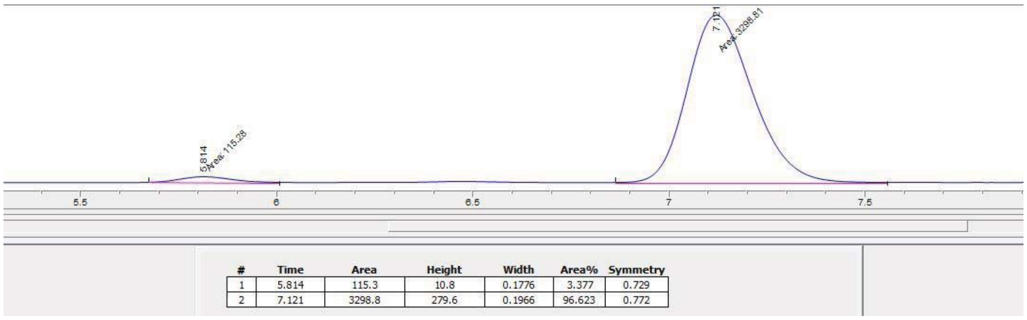

#	Time	Area	Height	Width	Area%	Symmetry
1	5.814	115.3	10.8	0.1776	3.377	0.729
2	7.121	3298.8	279.6	0.1966	96.623	0.772

(*2S,3S*)-2-((4-Fluorophenyl)amino)-*N,N*-dimethyl-3-phenylbutanamide (3sa):

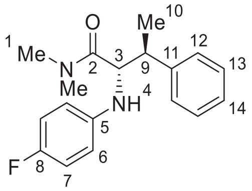

General procedure C: The reaction was carried out with substrate **1s** (19.6 mg, 0.10 mmol, 100 mol%) and styrene (23.0 μL, 0.20 mmol, 200 mol%). Purification of the residue by FCC (hexane/EtOAc 65:35) afforded the title compound (25.8 mg, 86%, >30:1 B:L, d.r. >20:1 *a:b*, e.r. = 97.5:2.5) as a pale-yellow oil. ^{1}H NMR analysis of the crude material gave >30:1 B:L and d.r. = 8:1. $[\alpha]_D^{25}$ = -2.6 (c = 1.0 $CHCl_3$); **IR (thin film)** ν_{max}/cm^{-1}: 3331 (br), 2932 (s), 1637 (s), 1509 (s), 1395 (s), 1216 (s), 823 (s), 701 (s); **^{1}H NMR** (500 MHz, $CDCl_3$) Data for the

major diastereomer *a* only: δ 7.33 – 7.22 (m, 5H, H^{12} + H^{13} + H^{14}), 6.86 – 6.83 (m, 2H, H^{7}), 6.56 – 6.53 (m, 2H, H^{6}), 4.46 (d, J = 6.5 Hz, 1H, H^{3}), 4.35 (br s, 1H, H^{4}), 3.31 – 3.25 (m, 1H, H^{9}), 2.90 (s, 3H, H^{1}), 2.69 (s, 3H, $H^{1'}$), 1.37 (d, J = 7.0 Hz, 3H, H^{10}); **^{13}C NMR** (126 MHz, $CDCl_3$) Data for the major diastereomer *a* only: δ 171.6 (C^{2}), 156.1 (d, J = 236.9 Hz, C^{8}), 143.4 (d, J = 1.3 Hz, C^{5}), 142.4 (C^{11}), 128.4 (C^{13}), 127.8 (C^{12}), 126.9 (C^{14}), 115.6 (d, J = 21.4 Hz, C^{7}), 115.1 (d, J = 7.6 Hz, C^{6}), 59.3 (C^{3}), 42.4 (C^{9}), 36.8 (C^{1}), 35.6 ($C^{1'}$), 16.1 (C^{10}); ^{19}F NMR (471 MHz, $CDCl_3$) δ -126.9; **HRMS** (ESI): calculated for $C_{18}H_{21}FN_2ONa$ $[M+Na]^+$ requires *m/z* 323.1530, found *m/z* 323.1548; **Chiral SFC:** DAICEL CHIRALCEL OD-H column (25 cm), CO_2:*i*-PrOH 95:5, 2.0 mL/min, 140 bar, 40 °C. Retention times: 8.7 mins (minor), 10.4 mins (major), e.r. = 97.5:2.5. To facilitate analysis by SFC, the major diastereoisomer was separated from the minor diastereoisomer by FCC.

SFC analysis of the major diastereomer of racemate, prepared using rac-BINAP:

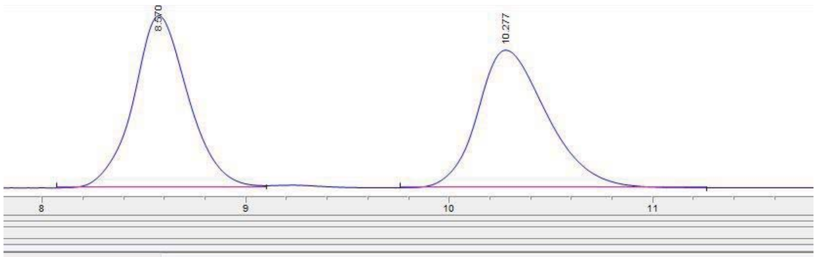

#	Time	Area	Height	Width	Area%	Symmetry
1	8.57	4248.9	224.7	0.2826	49.712	0.841
2	10.277	4298	179.6	0.3652	50.288	0.661

SFC analysis of the major diastereomer of enantioenriched material, prepared using (R)-SEGPHOS:

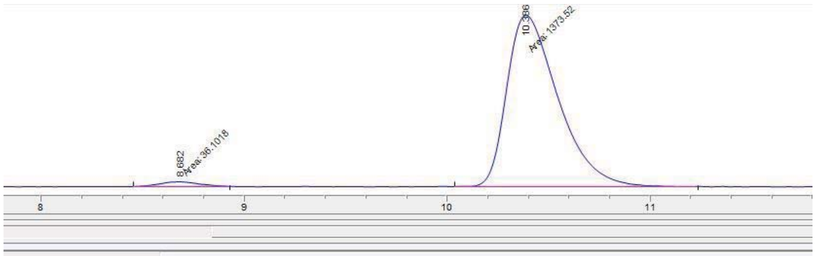

#	Time	Area	Height	Width	Area%	Symmetry
1	8.682	36.1	2.3	0.2564	2.561	0.876
2	10.386	1373.5	76.8	0.2979	97.439	0.573

(*2S,3S*)-2-((4-Chlorophenyl)amino)-*N,N*-dimethyl-3-phenylbutanamide (3ta):

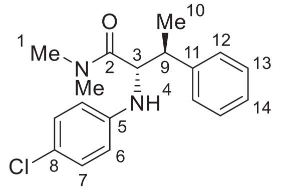

General procedure C: The reaction was carried out with substrate **1t** (21.2 mg, 0.10 mmol, 100 mol%) and styrene (23.0 µL, 0.20 mmol, 200 mol%). Purification of the residue by FCC (hexane/EtOAc 65:35) afforded the title compound (27.8 mg, 88%, >30:1 B:L, d.r. >20:1 *a:b*, e.r. = 97:3) as a pale-yellow solid. ^{1}H NMR analysis of the crude material gave >30:1 B:L and d.r. = 8:1. **m.p.** = 98 – 100 °C (hexane/EtOAc); $[\alpha]_D^{25}$ = -32.0 (c = 1.0 $CHCl_3$); **IR (thin film)** ν_{max}/cm^{-1}: 3327 (br), 2929 (s), 1635 (s), 1492 (s), 1394 (s), 816 (s), 699 (s); **^{1}H NMR** (500 MHz, $CDCl_3$) Data for the major diastereomer *a* only: δ 7.32 – 7.22 (m, 5H, H^{12} + H^{13} + H^{14}), 7.08 (d, *J* = 9.0 Hz, 2H, H^7), 6.53 (d, *J* = 9.0 Hz, 2H, H^6), 4.69 – 4.26 (m, 2H, H^3 + H^4), 3.32 – 3.27 (m, 1H, H^9), 2.90 (s, 3H, H^1), 2.67 (s, 3H, $H^{1'}$), 1.36 (d, *J* = 7.0 Hz, 3H, H^{10}); **^{13}C NMR** (126 MHz, $CDCl_3$) Data for the major diastereomer *a* only: δ 171.2 (C^2), 145.5 (C^5), 142.2 (C^{11}), 129.1 (C^7), 128.4 (C^{13}), 127.8 (C^{12}), 127.0 (C^{14}), 122.4 (C^8), 114.9 (C^6), 58.3 (C^3), 42.3 (C^9), 36.8 (C^1), 35.6 ($C^{1'}$), 15.9 (C^{10}); **HRMS** (ESI): calculated for $C_{18}H_{21}ClN_2ONa$ $[M+Na]^+$ requires *m/z* 339.1235, found *m/z* 339.1248; **Chiral SFC:** DAICEL CHIRALCEL OD-H column (25 cm), CO_2:*i*-PrOH 95:5, 2.0 mL/min, 140 bar, 40 °C. Retention times: 6.3 mins (minor), 7.5 mins (major), e.r. = 97:3. To facilitate analysis by SFC, the major diastereoisomer was separated from the minor diastereoisomer by FCC.

SFC analysis of the major diastereomer of racemate, prepared using rac-BINAP:

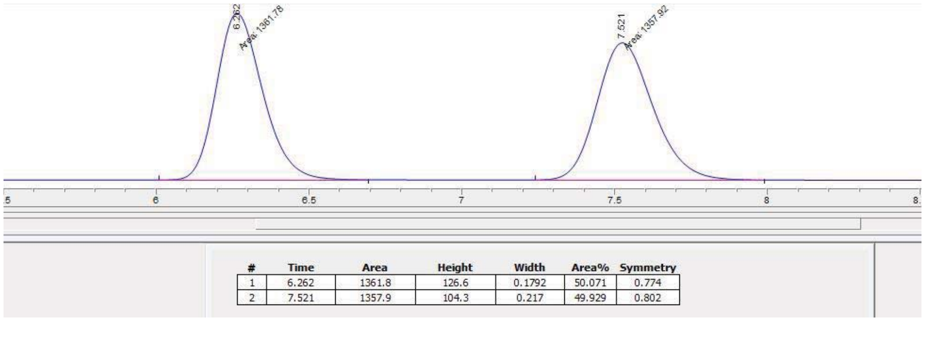

#	Time	Area	Height	Width	Area%	Symmetry
1	6.262	1361.8	126.6	0.1792	50.071	0.774
2	7.521	1357.9	104.3	0.217	49.929	0.802

SFC analysis of the major diastereomer of enantioenriched material, prepared using (R)-SEGPHOS:

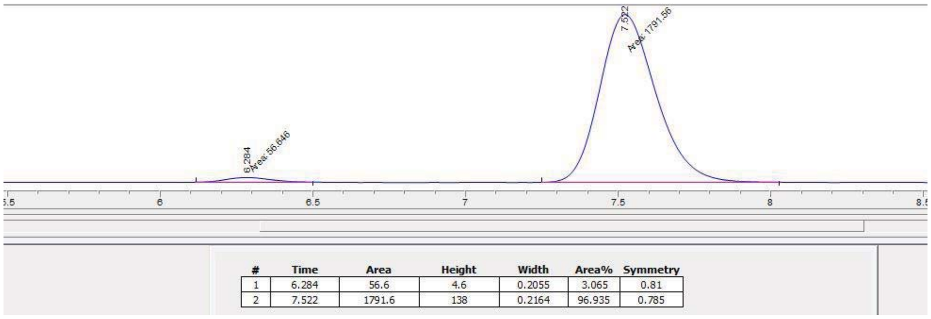

#	Time	Area	Height	Width	Area%	Symmetry
1	6.284	56.6	4.6	0.2055	3.065	0.81
2	7.522	1791.6	138	0.2164	96.935	0.785

(*2S,3S*)-*N,N*-Dimethyl-3-phenyl-2-((4-(trifluoromethyl)phenyl)amino)butanamide (3ua):

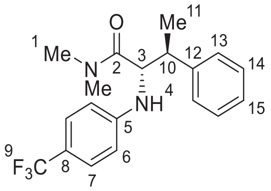

General procedure C: The reaction was carried out with substrate **1u** (24.6 mg, 0.10 mmol, 100 mol%) and styrene (23.0 µL, 0.20 mmol, 200 mol%). Purification of the residue by FCC (hexane/EtOAc 65:35) afforded the title compound (26.3 mg, 75%, >30:1 B:L, d.r. >20:1 *a:b*, e.r. = 98:2) as a pale-yellow solid. ^{1}H NMR analysis of the crude material gave >30:1 B:L and d.r. = 10:1. **m.p.** = 88 – 90 °C (hexane/EtOAc); $[\alpha]_D^{25}$ = -8.0 (c = 1.0 $CHCl_3$); **IR (thin film)** ν_{max}/cm^{-1}: 3324 (br), 2935 (s), 1639 (s), 1616 (s), 1324 (s), 1106 (s), 1065 (s), 701 (s); **^{1}H NMR** (500 MHz, $CDCl_3$) Data for the major diastereomer *a* only: δ 7.39 (d, *J* = 8.5 Hz, 2H, H^7), δ 7.36 – 7.25 (m, 5H, H^{13} + H^{14} + H^{15}), 6.63 (d, *J* = 8.5 Hz, 2H, H^6), 4.92 (br s, 1H, H^4), 4.62 (d, *J* = 6.0 Hz, 1H, H^3), 3.38 – 3.33 (m, 1H, H^{10}), 2.94 (s, 3H, H^1), 2.69 (s, 3H, $H^{1'}$), 1.40 (d, *J* = 7.0 Hz, 3H, H^{11}); **^{13}C NMR** (126 MHz, $CDCl_3$) Data for the major diastereomer *a* only: δ 170.8 (C^2), 149.4 (C^5), 141.9 (C^{12}), 128.5 (C^{14}), 128.0 (C^{13}), 127.8 (C^{15}), 126.6 (q, *J* = 3.8 Hz, C^7), 124.8 (q, *J* = 270.9 Hz, C^9), 119.1 (q, *J* = 32.8 Hz, C^8), 112.6 (C^6), 57.4 (C^3), 42.3 (C^{10}), 36.9 (C^1), 35.6 ($C^{1'}$), 15.7 (C^{11}); ^{19}F NMR (471 MHz, $CDCl_3$) δ -61.1; **HRMS** (ESI): calculated for $C_{19}H_{21}F_3N_2ONa$ $[M+Na]^+$ requires *m/z* 373.1498, found *m/z* 373.1509; **Chiral SFC:** DAICEL CHIRALCEL OD-H column (25 cm), CO_2:*i*-PrOH 95:5, 2.0 mL/min, 140 bar, 40 °C. Retention times: 6.9 mins (minor), 8.8 mins (major), e.r. = 98:2. To facilitate analysis by SFC, the major diastereoisomer was separated from the minor diastereoisomer by FCC.

SFC analysis of the major diastereomer of racemate, prepared using rac-BINAP:

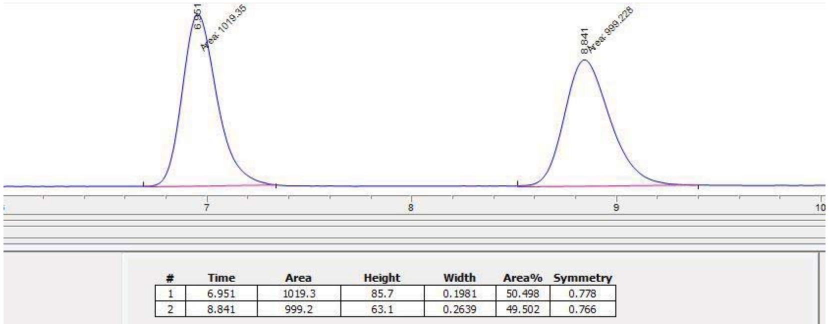

#	Time	Area	Height	Width	Area%	Symmetry
1	6.951	1019.3	85.7	0.1981	50.498	0.778
2	8.841	999.2	63.1	0.2639	49.502	0.766

SFC analysis of the major diastereomer of enantioenriched material, prepared using (R)-SEGPHOS:

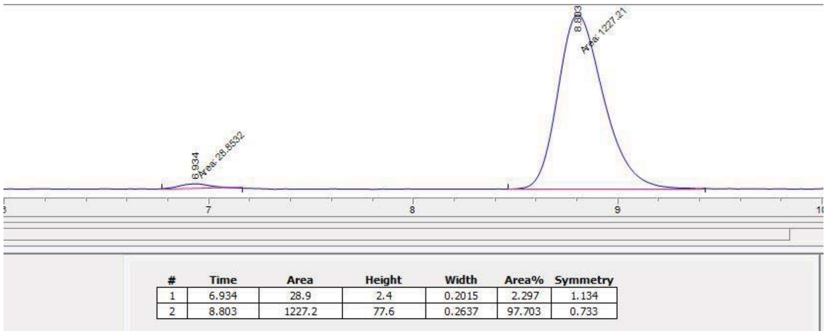

#	Time	Area	Height	Width	Area%	Symmetry
1	6.934	28.9	2.4	0.2015	2.297	1.134
2	8.803	1227.2	77.6	0.2637	97.703	0.733

(*2S,3S*)-2-((4-Fluoro-3-methoxyphenyl)amino)-*N,N*-dimethyl-3-phenylbutanamide (3va):

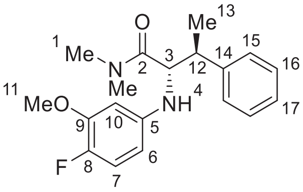

General procedure C: The reaction was carried out with substrate **1v** (22.6 mg, 0.10 mmol, 100 mol%) and styrene (23.0 µL, 0.20 mmol, 200 mol%). Purification of the residue by FCC (hexane/EtOAc 65:35) afforded the title compound (27.4 mg, 83%, >30:1 B:L, d.r. >20:1 *a:b*, e.r. = 96.5:3.5) as a pale-yellow oil. ^{1}H NMR analysis of the crude material gave >30:1 B:L and d.r. = 8:1. $[\alpha]_D^{25}$ = +10.1 (c = 1.0 $CHCl_3$); **IR (thin film)** ν_{max}/cm^{-1}: 3328 (br), 2934 (s), 1636 (s), 1519 (s), 1221 (s), 1121 (s), 701 (s); **^{1}H NMR** (500 MHz, $CDCl_3$) Data for the major diastereomer *a* only: δ 7.32 – 7.21 (m, 5H, H^{15} + H^{16} + H^{17}), 6.85 – 6.81 (m, 1H, H^7), 6.22 – 6.20 (m, 1H, H^6), 6.08 – 6.05 (m, 1H, H^{10}), 4.45 – 4.21 (m, 2H, H^3 + H^4), 3.79 (s, 3H, H^{11}), 3.30 – 3.25 (m, 1H, H^{12}), 2.90 (s, 3H, H^1), 2.72 (s, 3H, $H^{1'}$), 1.37 (d, *J* = 7.0 Hz, 3H, H^{13}); **^{13}C NMR** (126 MHz, $CDCl_3$) Data for the major diastereomer *a* only: δ 171.6 (C^2), 147.9 (d, *J* = 11.3 Hz, C^9), 145.8 (d, *J* = 235.6 Hz, C^8), 144.0 (d, *J* = 2.5 Hz, C^5), 142.3 (C^{14}), 128.4 (C^{16}),

127.8 (C^{15}), 126.9 (C^{17}), 116.0 (d, J = 18.9 Hz, C^7), 114.5 (d, J = 6.3 Hz, C^6), 110.9 (C^{10}), 59.2 (C^3), 56.0 (C^{11}), 42.5 (C^{12}), 36.8 (C^1), 35.5 ($C^{1'}$), 16.2 (C^{13}); ^{19}F NMR (471 MHz, $CDCl_3$) δ -148.7; **HRMS** (ESI): calculated for $C_{19}H_{23}FN_2O_2Na$ $[M+Na]^+$ requires *m/z* 353.1636, found *m/z* 353.1664; **Chiral SFC:** DAICEL CHIRALCEL OD-H column (25 cm), CO_2:*i*-PrOH 90:10, 2.0 mL/min, 140 bar, 40 °C. Retention times: 6.6 mins (minor), 8.3 mins (major), e.r. = 96.5:3.5. To facilitate analysis by SFC, the major diastereoisomer was separated from the minor diastereoisomer by FCC.

SFC analysis of the major diastereomer of racemate, prepared using rac-BINAP:

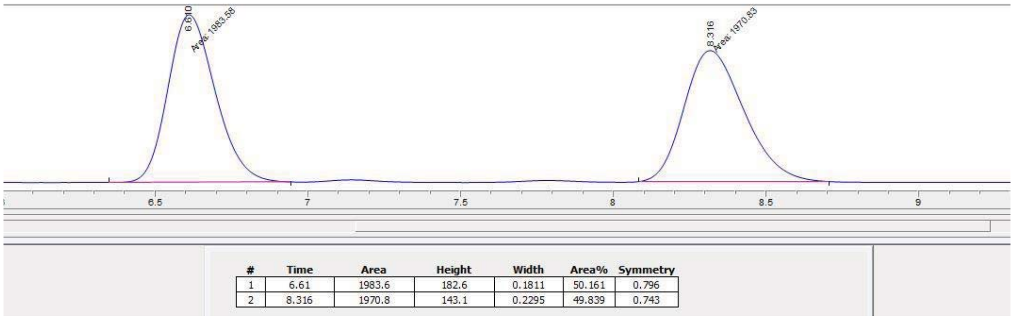

#	Time	Area	Height	Width	Area%	Symmetry
1	6.61	1983.6	182.6	0.1811	50.161	0.796
2	8.316	1970.8	143.1	0.2295	49.839	0.743

SFC analysis of the major diastereomer of enantioenriched material, prepared using (R)-SEGPHOS:

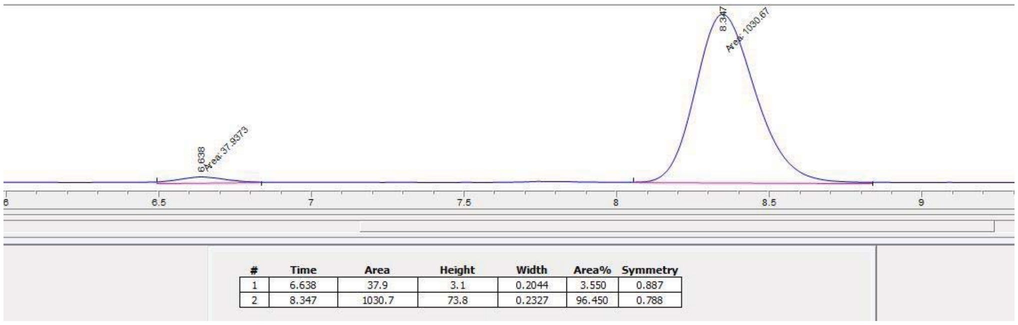

#	Time	Area	Height	Width	Area%	Symmetry
1	6.638	37.9	3.1	0.2044	3.550	0.887
2	8.347	1030.7	73.8	0.2327	96.450	0.788

(*2S,3S*)-2-((4-Hydroxyphenyl)amino)-1-morpholino-3-phenylbutan-1-one (3wa):

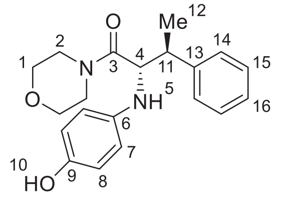

General procedure C: The reaction was carried out with substrate **1w** (23.6 mg, 0.10 mmol, 100 mol%) and styrene (23.0 μL, 0.20 mmol, 200 mol%). Purification of the residue by FCC (hexane/EtOAc 50:50) afforded the title compound (27.6 mg, 81%, >30:1 B:L, d.r. = 15:1 *a*:*b*,

e.r. = 96.5:3.5) as a pale-yellow oil. ^{1}H NMR analysis of the crude material gave >30:1 B:L and d.r. = 4:1. $[\boldsymbol{\alpha}]_D^{25}$ = -12.0 (c = 1.0 $CHCl_3$); **IR (thin film)** ν_{max}/cm^{-1}: 3337 (br), 2969 (s), 1619 (s), 1515 (s), 1452 (s), 1224 (s), 1114 (s), 701 (s); **^{1}H NMR** (500 MHz, $CDCl_3$) Data for the major diastereomer *a*: δ 7.36 – 7.26 (m, 5H, $H^{14} + H^{15} + H^{16}$), 6.67 (d, *J* = 8.5 Hz, 2H, H^8), 6.55 (d, *J* = 8.5 Hz, 2H, H^7), 5.10 (br s, 1H, H^{10}), 4.42 (d, *J* = 6.5 Hz, 1H, H^4), 4.13 (br s, 1H, H^5), 3.67 – 3.52 (m, 4H, H^1), 3.41 – 3.24 (m, 4H, $H^2 + H^{11}$), 3.13 – 3.08 (m, 1H, H^2), 1.40 (d, *J* = 7.0 Hz, 3H, H^{12}). Characteristic signal for the minor diastereomer *b*: 1.52 (d, *J* = 7.0 Hz, 2H, H^{12}); **^{13}C NMR** (126 MHz, $CDCl_3$) Data for the major diastereomer *a* only: δ 170.9 (C^3), 148.8 (C^9), 142.4 (C^6), 140.7 (C^{13}), 128.6 (C^{15}), 127.9 (C^{14}), 127.1 (C^{16}), 116.4 (C^8), 116.2 (C^7), 66.8 (C^1), 66.2 ($C^{1'}$), 66.1 (C^4), 46.0 (C^2), 42.4 (C^{11}), 42.6 ($C^{2'}$), 16.2 (C^{12}); **HRMS** (ESI): calculated for $C_{20}H_{24}N_2O_3Na$ $[M+Na]^+$ requires *m/z* 363.1679, found *m/z* 363.1684; **Chiral SFC:** DAICEL CHIRALCEL OD-H column (25 cm), CO_2:*i*-PrOH 90:10, 2.0 mL/min, 140 bar, 40 °C. Retention times: 32.3 mins (minor), 35.2 mins (major), e.r. = 96.5:3.5. To facilitate analysis by SFC, the major diastereoisomer was separated from the minor diastereoisomer by FCC.

SFC analysis of the major diastereomer of racemate, prepared using rac-BINAP:

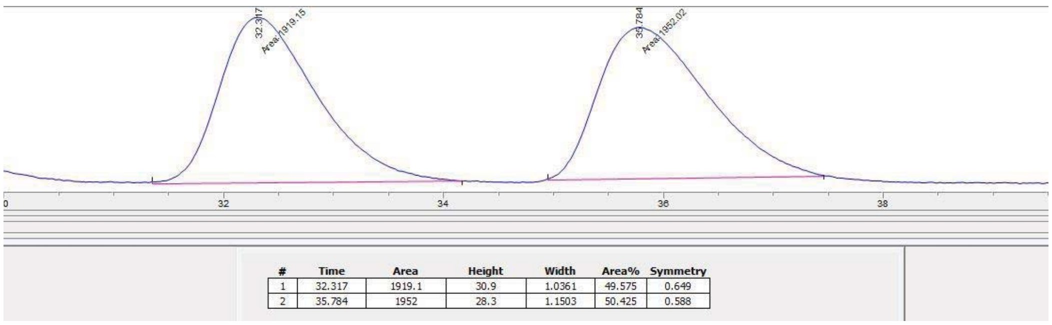

#	Time	Area	Height	Width	Area%	Symmetry
1	32.317	1919.1	30.9	1.0361	49.575	0.649
2	35.784	1952	28.3	1.1503	50.425	0.588

SFC analysis of the major diastereomer of enantioenriched material, prepared using (R)-SEGPHOS:

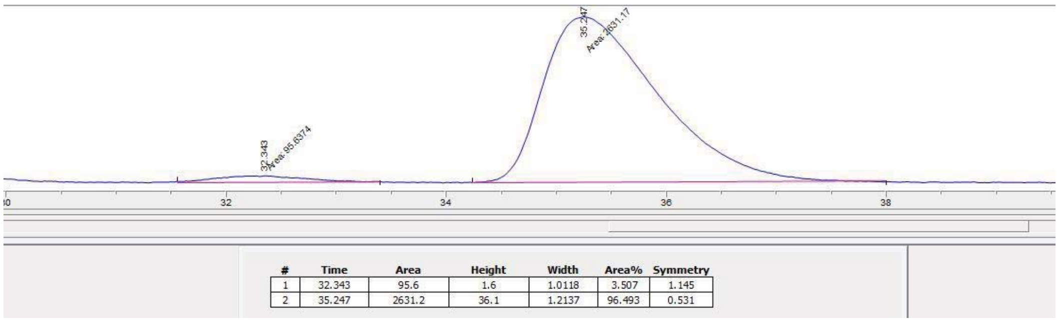

#	Time	Area	Height	Width	Area%	Symmetry
1	32.343	95.6	1.6	1.0118	3.507	1.145
2	35.247	2631.2	36.1	1.2137	96.493	0.531

(*2S,3S*)-*N,N*-Dimethyl-2-(phenylamino)-3-(*p*-tolyl)butanamide (3fb):

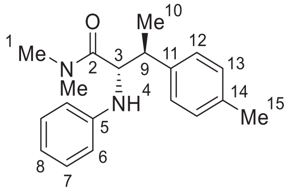

General procedure C: The reaction was carried out with substrate **1f** (17.8 mg, 0.10 mmol, 100 mol%) and 1-methyl-4-vinylbenzene **2b** (26.0 µL, 0.20 mmol, 200 mol%). Purification of the residue by FCC (hexane/EtOAc 70:30) afforded the title compound (19.8 mg, 67%, >30:1 B:L, d.r. = 8:1 *a:b*, e.r. = 96.5:3.5) as a pale-yellow solid. ^{1}H NMR analysis of the crude material gave >30:1 B:L and d.r. = 8:1. **m.p.** = 121 – 123 °C (hexane/EtOAc); $[\boldsymbol{\alpha}]_{\boldsymbol{D}}^{\boldsymbol{23}}$ = +15.1 (c = 0.14, $CHCl_3$); **IR (thin film)** ν_{max}/cm^{-1}: 3379 (br), 3009 (s), 2931 (s), 2855 (s), 1611 (s); **^{1}H NMR** (500 MHz, $CDCl_3$) Data for the major diastereomer *a*: δ 7.17 – 7.09 (m, 6H, H^7 + H^{12} + H^{13}), 6.72 – 6.69 (m, 1H, H^8), 6.63 (d, *J* = 8.0 Hz, 2H, H^6), 4.69 – 4.31 (m, 2H, H^3 + H^4), 3.32 – 3.27 (m, 1H, H^9), 2.90 (s, 3H, H^1), 2.67 (s, 3H, $H^{1'}$), 2.32 (s, 3H, H^{15}), 1.35 (d, *J* = 7.0 Hz, 3H, H^{10}). Characteristic signals for the minor diastereomer *b*: 6.55 (d, *J* = 8.0 Hz, 2H, H^6), 2.75 (s, 3H, H^1), 2.62 (s, 3H, $H^{1'}$), 1.44 (d, *J* = 7.0 Hz, 3H, H^{10}); **^{13}C NMR** (126 MHz, $CDCl_3$) Data for the major diastereomer *a* only: δ 171.7 (C^2), 147.1 (C^5), 139.5 (C^{11}), 136.6 (C^{14}), 129.4 (C^7), 129.3 (C^{13}), 127.9 (C^{12}), 118.1 (C^8), 114.1 (C^6), 58.5 (C^3), 42.0 (C^9), 37.1 (C^1), 35.8 ($C^{1'}$), 21.2 (C^{15}), 16.2 (C^9); **HRMS** (ESI): calculated for $C_{19}H_{25}N_2O$ $[M+H]^+$ requires *m/z* 296.1961, found *m/z* 297.1962; **Chiral SFC:** DAICEL CHIRALCEL OD-H column (25 cm), CO_2:*i*-PrOH 95:5, 2.5 mL/min, 140 bar, 40 °C. Retention times: 11.1 mins (major), 11.6 mins (minor), e.r. = 96.5:3.5. To facilitate analysis by SFC, the major diastereoisomer was separated from the minor diastereoisomer by FCC.

SFC analysis of the major diastereomer of racemate, prepared using rac-BINAP:

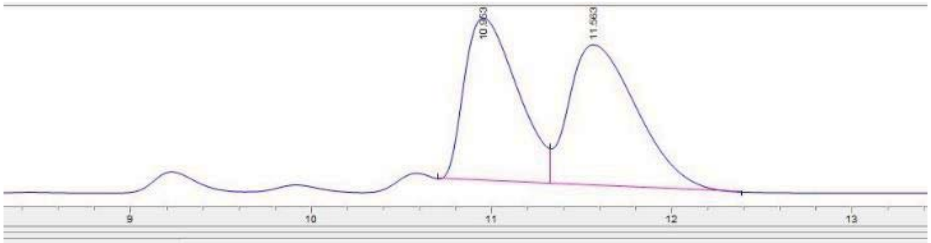

#	Time	Area	Height	Width	Area%	Symmetry
1	10.953	5411.8	263.5	0.3237	47.516	0.529
2	11.563	5977.7	227.9	0.4101	52.484	0.532

SFC analysis of the major diastereomer of enantioenriched material, prepared using (R)-SEGPHOS:

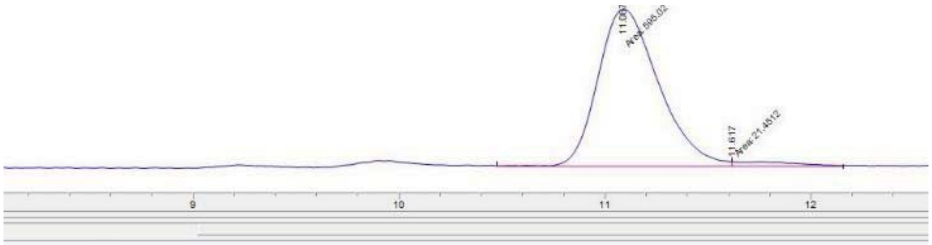

#	Time	Area	Height	Width	Area%	Symmetry
1	11.087	595	28.7	0.346	96.520	0.727
2	11.617	21.5	9.7E-1	0.2639	3.480	0

(*2S,3S*)-3-(4-Methoxyphenyl)-*N,N*-dimethyl-2-(phenylamino)butanamide (3fc):

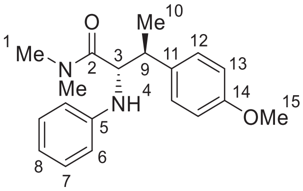

General procedure C: The reaction was carried out with substrate **1f** (17.8 mg, 0.10 mmol, 100 mol%) and 1-methoxy-4-vinylbenzene **2c** (27.0 μL, 0.20 mmol, 200 mol%). Purification of the residue by FCC (hexane/EtOAc 70:30) afforded the title compound (18.7 mg, 60%, >30:1 B:L, d.r. >20:1 *a:b*, e.r. = 98:2) as a pale-yellow oil. ^{1}H NMR analysis of the crude material gave >30:1 B:L and d.r. = 9:1. $[\boldsymbol{\alpha}]_{\boldsymbol{D}}^{\mathbf{25}}$ = +2.9 (c = 0.26, $CHCl_3$); **IR (thin film)** ν_{max}/cm^{-1}: 3711 (s), 3681 (s), 3344 (br), 2966 (s), 2936 (s), 2873 (s), 2835 (s), 1636 (s), 1602 (s), 1583 (s), 1510 (s); **^{1}H NMR** (500 MHz, $CDCl_3$) Data for the major diastereomer *a* only: δ 7.17 – 7.14 (m, 4H, $H^7 + H^{12}$), 6.85 (d, *J* = 8.5 Hz, 2H, H^{13}), 6.72 – 6.69 (m, 1H, H^8), 6.64 (d, *J* = 7.5 Hz, 2H, H^6), 4.67 – 4.29 (m, 2H, $H^3 + H^4$), 3.79 (s, 3H, H^{15}), 3.32 – 3.26 (m, 1H, H^9), 2.90 (s, 3H, H^1), 2.68 (s, 3H, $H^{1'}$), 1.35 (d, *J* = 7.0 Hz, 3H, H^{10}); **^{13}C NMR** (126 MHz, $CDCl_3$) Data for the major diastereomer *a* only: δ 171.5 (C^2), 158.5 (C^{14}), 146.9 (C^5), 134.4 (C^{11}), 129.3 (C^7), 128.8 (C^{12}), 118.0 (C^8), 113.9 (C^{13}), 113.8 (C^6), 58.4 (C^3), 55.2 (C^{15}), 41.4 (C^9), 36.9 (C^1), 35.6 ($C^{1'}$), 16.1 (C^{10}); **HRMS** (ESI): calculated for $C_{19}H_{25}N_2O_2$ $[M+H]^+$ requires *m/z* 313.1911, found *m/z* 313.1912; **Chiral SFC:** YMC Chiral ART Cellulose-SC column (25 cm), CO_2:*i*-PrOH 85:15, 3.0 mL/min, 140 bar, 40 °C. Retention times: 5.6 mins (minor), 6.9 mins (major), e.r. = 98:2. To facilitate analysis by SFC, the major diastereoisomer was separated from the minor diastereoisomer by FCC.

SFC analysis of the major diastereomer of racemate, prepared using rac-BINAP:

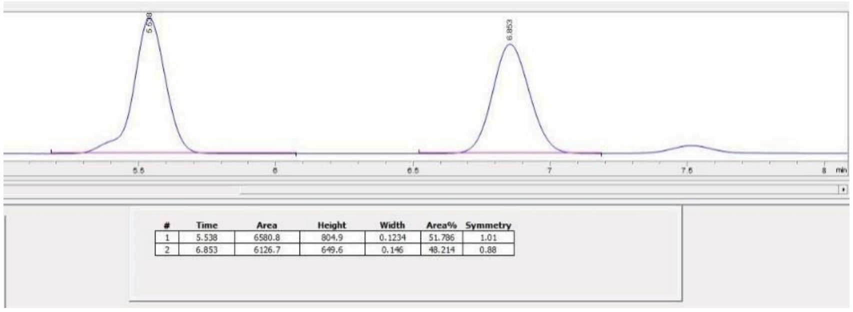

#	Time	Area	Height	Width	Area%	Symmetry
1	5.538	6580.8	804.9	0.1234	51.786	1.01
2	6.853	6126.7	649.6	0.146	48.214	0.88

SFC analysis of the major diastereomer of enantioenriched material, prepared using (R)-SEGPHOS:

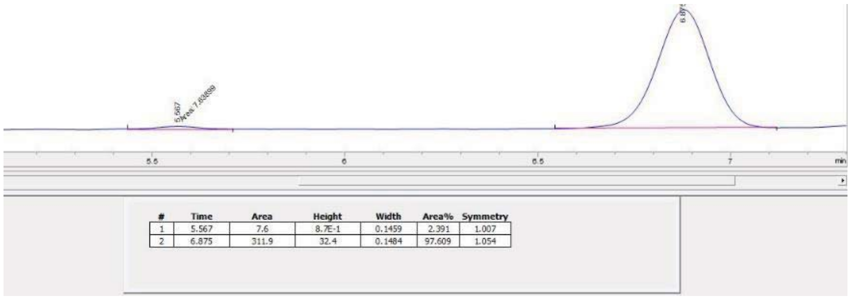

#	Time	Area	Height	Width	Area%	Symmetry
1	5.567	7.6	8.7E-1	0.1459	2.391	1.007
2	6.875	311.9	32.4	0.1484	97.609	1.054

(*2S,3S*)-3-(4-(*N,N*-Diethylsulfamoyl)phenyl)-*N,N*-dimethyl-2-(phenylamino)butanamide (3fd):

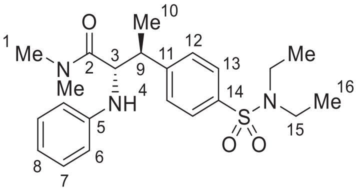

General procedure C: The reaction was carried out with substrate **1f** (17.8 mg, 0.10 mmol, 100 mol%) and *N,N*-diethyl-4-vinylbenzenesulfonamide **2d** (47.8 mg, 0.20 mmol, 200 mol%). Purification of the residue by FCC (hexane/EtOAc 60:30) afforded the title compound (29.8 mg, 71%, >30:1 B:L, d.r. = 6:1 *a:b*, e.r. = 97:3) as a pale-yellow oil. ^{1}H NMR analysis of the crude material gave >30:1 B:L and d.r. = 6:1; $[\boldsymbol{\alpha}]_{\boldsymbol{D}}^{\mathbf{27}}$ = -2.8 (c = 1.0, $CHCl_3$); **IR (thin film)** ν_{max}/cm^{-1}: 3329 (br), 2974 (s), 1638 (s), 1601 (s), 1497 (s), 1329 (s), 1154 (s), 1013 (s), 696 (s); **^{1}H NMR** (500 MHz, $CDCl_3$) Data for the major diastereomer *a* only: δ 7.74 (d, *J* = 8.5 Hz, 2H, H^{13}), 7.40 (d, *J* = 8.5 Hz, 2H, H^{12}), 7.15 – 7.12 (m, 2H, H^7), 6.73 – 6.70 (m, 1H, H^8), 6.59 (d, *J* = 7.5 Hz, 2H, H^6), 4.71 – 4.18 (m, 2H, H^3 + H^4), 3.43 – 3.35 (m, 1H, H^9), 3.22 (q, *J* = 7.0 Hz, 4H, H^{15}), 2.93 (s, 3H, H^1), 2.82 (s, 3H, H$^{1'}$), 1.40 (d, *J* = 7.0 Hz, 3H, H^{10}), 1.13 (t, *J* = 7.0 Hz, 6H, H^{16}); **^{13}C NMR** (126 MHz, $CDCl_3$) Data for the major diastereomer *a* only: δ 171.4

(C^2), 147.5 (C^5), 146.6 (C^{11}), 138.8 (C^{14}), 129.3 (C^7), 128.7 (C^{13}), 127.0 (C^{12}), 118.5 (C^8), 114.1 (C^6), 58.2 (C^3), 42.7 (C^9), 42.1 (C^{15}), 37.2 (C^1), 35.7 ($C^{1'}$), 16.3 (C^{10}), 14.2 (C^{16}); **HRMS** (ESI): calculated for $C_{22}H_{32}N_3O_3S$ $[M+H]^+$ requires *m/z* 418.2159, found *m/z* 418.2162; **Chiral SFC:** DAICEL CHIRALCEL SC column (25 cm), CO_2:*i*-PrOH 70:30, 3.0 mL/min, 231 bar, 40 °C. Retention times: 5.7 mins (minor), 7.4 mins (major), e.r. = 97:3. To facilitate analysis by SFC, the major diastereoisomer was separated from the minor diastereoisomer by FCC.

SFC analysis of the major diastereomer of racemate, prepared using rac-BINAP:

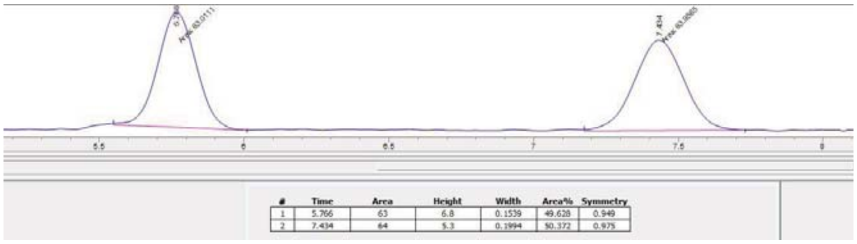

SFC analysis of the major diastereomer of enantioenriched material, prepared using (R)-SEGPHOS:

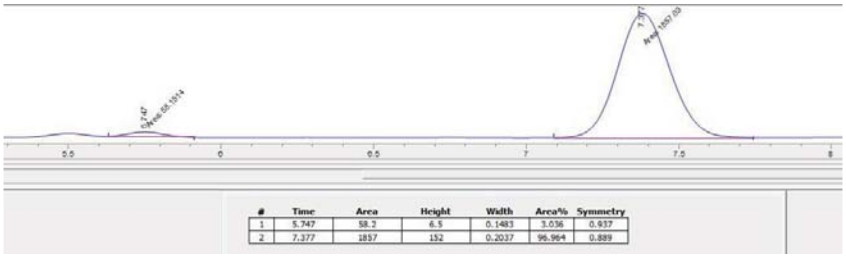

(*2S,3S*)-*N,N*-Dimethyl-3-(naphthalen-2-yl)-2-(phenylamino)butanamide (3fe):

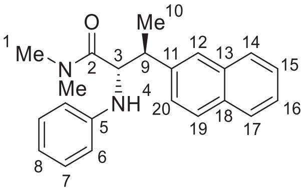

General procedure C: The reaction was carried out with substrate **1f** (17.8 mg, 0.10 mmol, 100 mol%) and 2-vinylnaphthalene **2e** (30.8 mg, 0.20 mmol, 200 mol%). Purification of the residue by FCC (hexane/EtOAc 70:30) afforded the title compound (21.9 mg, 66%, >30:1 B:L, d.r. = 7:1 *a:b*, e.r. = 98:2) as a pale-yellow solid. ^{1}H NMR analysis of the crude material gave >30:1 B:L and d.r. = 7:1. **m.p.** = 148 – 151 °C (hexane/EtOAc); $[\alpha]_D^{20}$ = -9.6 (c = 0.17, $CHCl_3$); **IR (thin film)** ν_{max}/cm^{-1}: 3334 (br), 3052 (s), 3017 (s), 2968 (s), 2932 (s), 2874 (s), 1636 (s),

1601 (s), 1504 (s); **^{1}H NMR** (500 MHz, $CDCl_3$) Data for the major diastereomer *a*: δ 7.82 – 7.79 (m, 3H, ArH), 7.70 (s, 1H, H^{12}), 7.50 – 7.43 (m, 2H, ArH), 7.40 (d, J = 8.5 Hz, 1H, ArH), 7.17 – 7.14 (m, 2H, H^{7}), 6.72 – 6.69 (m, 1H, H^{8}), 6.66 (d, J = 8.0 Hz, 2H, H^{6}), 4.82 – 4.45 (m, 2H, H^{3} + H^{4}), 3.53 – 3.48 (m, 1H, H^{9}), 2.89 (s, 3H, H^{1}), 2.62 (s, 3H, $H^{1'}$), 1.47 (d, J = 7.0 Hz, 3H, H^{10}). Characteristic signals for the minor diastereomer *b*: 7.13 – 7.10 (m, 2H, H^{7}), 6.59 (d, J = 8.0 Hz, 2H, H^{6}), 2.69 (s, 3H, H^{1}), 2.55 (s, 3H, $H^{1'}$), 1.56 (d, J = 7.0 Hz, 3H, H^{10}); **^{13}C NMR** (126 MHz, $CDCl_3$) Data for the major diastereomer *a* only: δ 171.7 (C^{2}), 147.1 (C^{5}), 140.2 (C^{11}), 133.6 (ArC), 132.7 (ArC), 129.5 (C^{7}), 128.2 (ArC), 127.81 (ArC), 127.78 (ArC), 126.6 (ArC), 126.30 (ArC), 126.27 (ArC), 125.8 (ArC), 118.2 (C^{8}), 114.1 (C^{6}), 58.3 (C^{3}), 42.6 (C^{9}), 37.2 (C^{1}), 35.8 ($C^{1'}$), 16.2 (C^{10}); **HRMS** (ESl): calculated for $C_{22}H_{25}N_2O$ $[M+H]^+$ requires *m/z* 333.1961, found *m/z* 333.1962; **Chiral SFC:** DAICEL CHIRALCEL OD-H column (25 cm), CO_2:*i*-PrOH 85:15, 2.0 mL/min, 160 bar, 40 °C. Retention times: 7.9 mins (major), 9.5 mins (minor), e.r. = 98:2. To facilitate analysis by SFC, the major diastereoisomer was separated from the minor diastereoisomer by FCC.

SFC analysis of the major diastereomer of racemate, prepared using rac-BINAP:

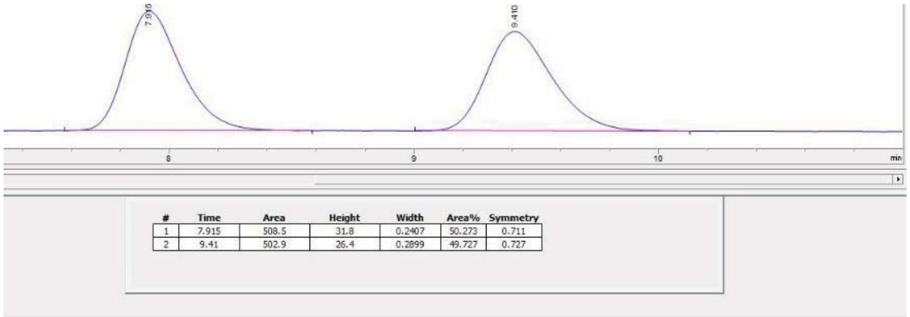

SFC analysis of the major diastereomer of enantioenriched material, prepared using (R)-SEGPHOS:

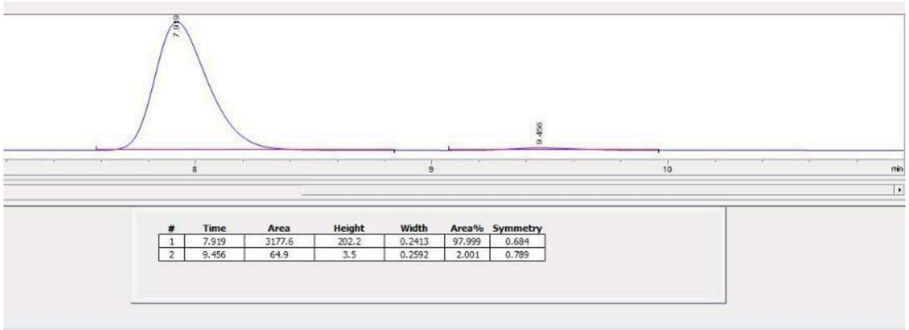

(*2S,3S*)-*N,N*-Dimethyl-3-(1-(methyl-(λ¹-oxidaneyl)-(*p*-tolyl)sulfinyl)-1*H*-indol-3-yl)-2-(phenylamino)butanamide (3ff):

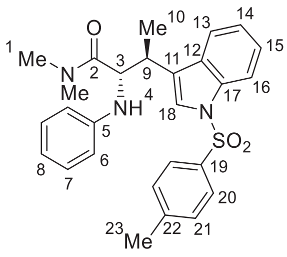

General procedure C: The reaction was carried out with substrate **1f** (17.8 mg, 0.10 mmol, 100 mol%) and 1-(methyl-(λ¹-oxidaneyl)-(*p*-tolyl)sulfinyl)-3-vinyl-1*H*-indole **2f** (62.4 mg, 0.20 mmol, 200 mol%). Purification of the residue by FCC (hexane/EtOAc 60:40) afforded the title compound (40.4 mg, 85%, >30:1 B:L, d.r. >20:1 *a:b*, e.r. = 98:2) as a pale-yellow oil. ^{1}H NMR analysis of the crude material gave >30:1 B:L and d.r. = 5:1. $[\boldsymbol{\alpha}]_D^{25}$ = -4.5 (c = 1.0 $CHCl_3$); **IR (thin film)** ν_{max}/cm^{-1}: 3330 (br), 3053 (s), 1637 (s), 1602 (s), 1497 (s), 1173 (s), 747 (s), 671 (s); **^{1}H NMR** (500 MHz, $CDCl_3$) Data for the major diastereomer *a* only: δ 8.02 (d, *J* = 8.0 Hz, 1H, ArH), 7.70 (d, *J* = 8.0 Hz, 2H, H^{20}), 7.59 (d, *J* = 8.0 Hz, 1H, ArH), 7.43 (s, 1H, H^{18}), 7.36 – 7.27 (m, 2H, ArH), 7.20 – 7.16 (m, 4H, ArH), 6.76 – 6.74 (m, 1H, H^8), 6.66 (d, *J* = 8.0 Hz, 2H, H^6), 5.12 – 4.39 (m, 2H, $H^3 + H^4$), 3.61 – 3.56 (m, 1H, H^9), 2.85 (s, 3H, H^1), 2.63 (s, 3H, $H^{1'}$), 2.34 (s, 3H, H^{23}), 1.45 (d, *J* = 7.0 Hz, 3H, H^{10}); **^{13}C NMR** (126 MHz, $CDCl_3$) Data for the major diastereomer *a* only: δ 171.4 (C^2), 146.7 (C^5), 144.8 (ArC), 135.2 (ArC), 135.1 (ArC), 130.5 (ArC), 129.8 (ArC), 129.3 (ArC), 126.6 (C^{20}), 124.8 (ArC), 124.1 (ArC), 123.4 (ArC), 123.2 (ArC), 119.3 (ArC), 118.2 (C^8), 113.93 (C^6), 113.89 (ArC), 56.6 (C^3), 37.1 (C^1), 35.6 ($C^{1'}$), 33.8 (C^9), 21.5 (C^{23}), 16.1 (C^{10}); **HRMS** (ESl): calculated for $C_{27}H_{29}N_3O_3SNa$ $[M+Na]^+$ requires *m/z* 498.1822, found *m/z* 498.1826; **Chiral SFC:** DAICEL CHIRALCEL IE column (25 cm), CO_2:*i*-PrOH 70:30, 3.0 mL/min, 180 bar, 40 °C. Retention times: 7.7 mins (minor), 8.8 mins (major), e.r. = 98:2. To facilitate analysis by SFC, the major diastereoisomer was separated from the minor diastereoisomer by FCC.

SFC analysis of the major diastereomer of racemate, prepared using rac-BINAP:

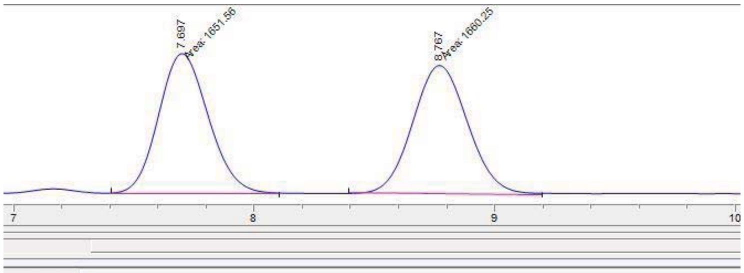

#	Time	Area	Height	Width	Area%	Symmetry
1	7.697	1651.6	113.5	0.2426	49.869	0.858
2	8.767	1660.3	103.8	0.2666	50.131	0.909

SFC analysis of the major diastereomer of enantioenriched material, prepared using (R)-SEGPHOS:

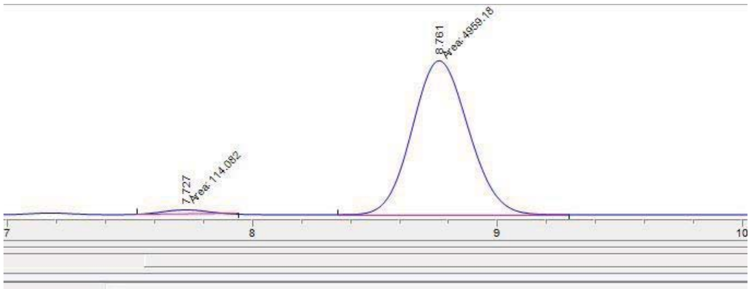

#	Time	Area	Height	Width	Area%	Symmetry
1	7.727	114.1	8.8	0.2161	2.249	1.157
2	8.761	4959.2	301.1	0.2745	97.751	0.895

Compound (3fg):

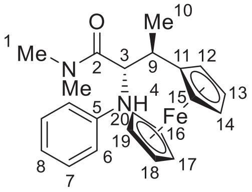

General procedure C: The reaction was carried out with substrate **1f** (17.8 mg, 0.10 mmol, 100 mol%) and vinylferrocene **2g** (42.4 mg, 0.20 mmol, 200 mol%). Purification of the residue by FCC (hexane/EtOAc 70:30) afforded the title compound (38.2 mg, 98%, >30:1 B:L, d.r. >20:1 *a*:*b*, e.r. = 98:2) as a yellow oil. ^{1}H NMR analysis of the crude material gave >30:1 B:L and d.r. = 5:1. $[\boldsymbol{\alpha}]_{\boldsymbol{D}}^{\mathbf{25}}$ = +126.0 (c = 1.0 $CHCl_3$); **IR (thin film)** ν_{max}/cm^{-1}: 3335 (br), 3054 (s), 1638 (s), 1602 (s), 1505 (s), 1106 (s), 749 (s); **^{1}H NMR** (500 MHz, $CDCl_3$) Data for the major diastereomer *a* only: δ 7.18 – 7.15 (m, 2H, H^7), 6.71 – 6.68 (m, 1H, H^8), 6.59 (d, *J* = 8.0 Hz, 2H, H^6), 4.77 (br s, 1H, H^4), 4.36 (d, *J* = 4.0 Hz, 1H, H^3), 4.18 – 4.09 (m, 9H, H^{12} + H^{13} + H^{14} + H^{15} + H^{16} + H^{17} + H^{18} + H^{19} + H^{20}), 3.07 – 3.02 (m, 1H, H^9), 2.86 (s, 3H, H^1), 2.60 (s, 3H, $H^{1'}$), 1.37 (d, *J* = 6.5 Hz, 3H, H^{10}); **^{13}C NMR** (126 MHz, $CDCl_3$) Data for the major diastereomer *a* only: δ 171.2 (C^2), 146.8 (C^5), 129.3 (C^7), 117.6 (C^8), 113.5 (C^6), 69.1 – 66.4

(m, ArC), 58.3 (C[3]), 37.5 (C[1]), 36.4 (C[9]), 35.6 (C[1']), 15.2 (C[10]); **HRMS** (ESI): calculated for $C_{22}H_{26}FeN_2ONa$ $[M+Na]^+$ requires *m/z* 413.1287, found *m/z* 413.1291; **Chiral SFC:** YMC Chiral ART Cellulose-SC column (25 cm), CO_2:*i*-PrOH 80:20, 3.0 mL/min, 170 bar, 40 °C. Retention times: 7.7 mins (minor), 9.3 mins (major), e.r. = 98:2. To facilitate analysis by SFC, the major diastereoisomer was separated from the minor diastereoisomer by FCC.

SFC analysis of the major diastereomer of racemate, prepared using rac-BINAP:

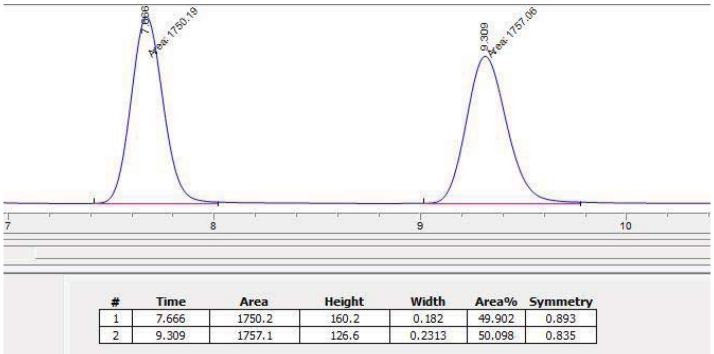

#	Time	Area	Height	Width	Area%	Symmetry
1	7.666	1750.2	160.2	0.182	49.902	0.893
2	9.309	1757.1	126.6	0.2313	50.098	0.835

SFC analysis of the major diastereomer of enantioenriched material, prepared using (R)-SEGPHOS:

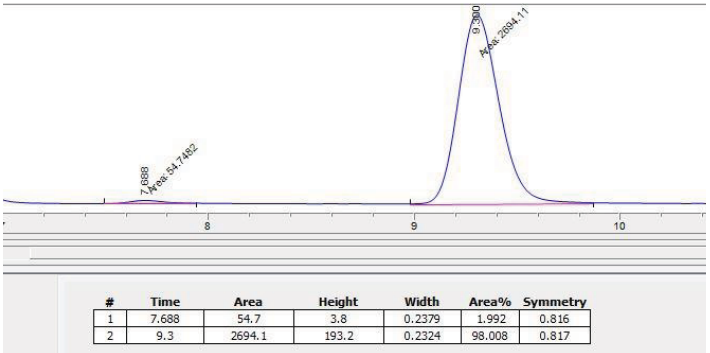

#	Time	Area	Height	Width	Area%	Symmetry
1	7.688	54.7	3.8	0.2379	1.992	0.816
2	9.3	2694.1	193.2	0.2324	98.008	0.817

(*2S,3S*)-*N,N*-Dimethyl-2-(phenylamino)-3-(4-(4,4,5,5-tetramethyl-1,3,2-dioxaborolan-2-yl)phenyl)butanamide (3fh):

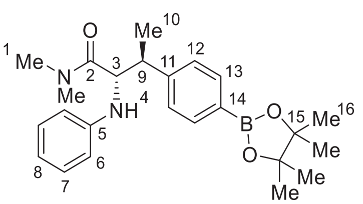

General procedure C: The reaction was carried out with substrate **1f** (17.8 mg, 0.10 mmol, 100 mol%) and 4,4,5,5-tetramethyl-2-(4-vinylphenyl)-1,3,2-dioxaborolane **2h** (47.0 µL, 0.20 mmol, 200 mol%). Purification of the residue by FCC (hexane/EtOAc 70:30) afforded the title

compound (31.3 mg, 75%, >30:1 B:L, d.r. = 8:1 *a:b*, e.r. = 96:4) as a pale-yellow solid. ^{1}H NMR analysis of the crude material gave >30:1 B:L and d.r. = 8:1. **m.p.** = 165 – 167 (hexane/EtOAc); $[\boldsymbol{\alpha}]_{\boldsymbol{D}}^{\boldsymbol{27}}$ = **-**9.1 (c = 1.0, $CHCl_3$); **IR (thin film)** ν_{max}/cm^{-1}: 3336 (br), 2977 (s), 1642 (s), 1603 (s), 1501 (s), 1361 (s), 1144 (s), 1091 (s); **^{1}H NMR** (500 MHz, $CDCl_3$) Data for the major diastereomer *a* only: δ 7.78 (d, *J* = 8.0 Hz, 2H, H^{13}), 7.28 (d, *J* = 8.0 Hz, 2H, H^{12}), 7.20 – 7.17 (m, 2H, H^{7}), 6.76 – 6.73 (m, 1H, H^{8}), 6.68 (d, *J* = 8.0 Hz, 2H, H^{6}), 5.30 – 4.37 (m, 2H, H^{3} + H^{4}), 3.42 – 3.34 (m, 1H, H^{9}), 2.90 (s, 3H, H^{1}), 2.63 (s, 3H, $H^{1'}$), 1.40 – 1.37 (m, 15H, H^{10} + H^{16}); **^{13}C NMR** (126 MHz, $CDCl_3$) Data for the major diastereomer *a* only: δ 171.3 (C^{2}), 146.6 (C^{5}), 145.8 (C^{11}), 135.0 (C^{13}), 129.4 (C^{7}), 127.4 (C^{12}), 118.3 (C^{8}), 114.2 (C^{6}), 83.8 (C^{15}), 58.2 (C^{3}), 42.4 (C^{9}), 37.0 (C^{1}), 35.7 ($C^{1'}$), 24.94 (C^{16}), 24.85 ($C^{16'}$), 15.6 (C^{10}); **HRMS** (ESI): calculated for $C_{24}H_{34}BN_2O_3$ $[M+H]^+$ requires *m/z* 409.2657, found *m/z* 409.2662; **Chiral SFC:** DAICEL CHIRALCEL SC column (25 cm), CO_2:*i*-PrOH 80:20, 3.0 mL/min, 227 bar, 40 °C. Retention times: 2.8 mins (minor), 3.5 mins (major), e.r. = 96:4. To facilitate analysis by SFC, the major diastereoisomer was separated from the minor diastereoisomer by FCC.

SFC analysis of the major diastereomer of racemate, prepared using rac-BINAP:

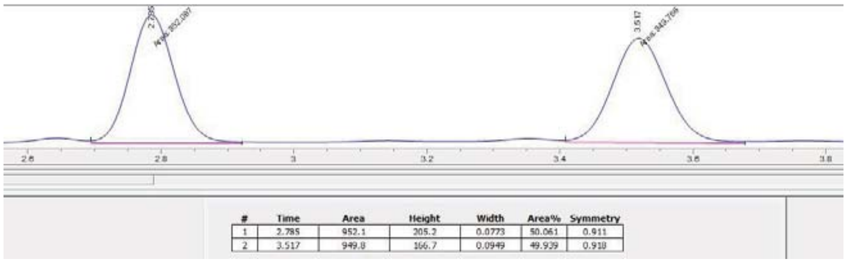

SFC analysis of the major diastereomer of enantioenriched material, prepared using (R)-SEGPHOS:

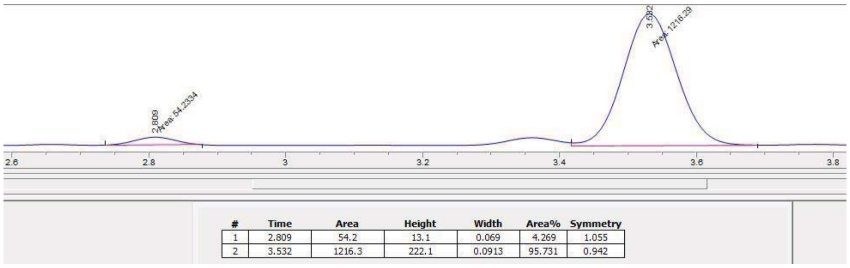

(*2S,3S*)-3-(4-Bromophenyl)-*N,N*-dimethyl-2-(phenylamino)butanamide (3fi):

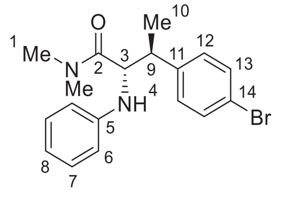

General procedure C: The reaction was carried out with substrate **1f** (17.8 mg, 0.10 mmol, 100 mol%), and 1-bromo-4-vinylbenzene **2i** (26.0 μL, 0.20 mmol, 200 mol%) at 140 ºC. Purification of the residue by FCC (hexane/EtOAc 70:30) afforded the title compound (18.0 mg, 50%, >30:1 B:L, d.r. >20:1 *a:b*, e.r. = 98:2) as a pale-yellow solid. ^{1}H NMR analysis of the crude material gave >30:1 B:L, d.r. = 8:1 and 35% substrate **1f**. **m.p.** = 102 – 104 °C (hexane/EtOAc); $[\boldsymbol{\alpha}]_{\boldsymbol{D}}^{\mathbf{25}}$ = -6.7 (c = 1.0 $CHCl_3$); **IR (thin film)** ν_{max}/cm^{-1}: 3328 (br), 2924 (s), 2852 (s), 1637 (s), 1602 (s), 1490 (s), 1398 (s), 750 (s), 693 (s); **^{1}H NMR** (500 MHz, $CDCl_3$) Data for the major diastereomer *a* only: δ 7.42 (d, *J* = 8.5 Hz, 2H, H^{13}), 7.16 – 7.11 (m, 4H, H^7 + H^{12}), 6.73 – 6.70 (m, 1H, H^8), 6.62 (d, *J* = 8.5 Hz, 2H, H^6), 4.69 – 4.10 (m, 2H, H^3 + H^4), 3.30 – 3.24 (m, 1H, H^9), 2.91 (s, 3H, H^1), 2.77 (s, 3H, $H^{1'}$), 1.35 (d, *J* = 7.0 Hz, 3H, H^{10}); **^{13}C NMR** (126 MHz, $CDCl_3$) Data for the major diastereomer *a* only: δ 171.4 (C^2), 146.8 (C^5), 141.4 (C^{11}), 131.4 (C^{13}), 129.7 (C^{12}), 129.3 (C^7), 120.7 (C^{14}), 118.4 (C^8), 114.1 (C^6), 58.2 (C^3), 42.0 (C^9), 37.1 ($C^{1'}$), 35.7 (C^1), 16.3 (C^{10}); **HRMS** (ESl): calculated for $C_{18}H_{21}BrN_2ONa$ $[M+Na]^+$ requires *m/z* 383.0729, found *m/z* 383.0733; **Chiral SFC:** DAICEL CHIRALCEL OD-H column (25 cm), CO_2:*i*-PrOH 90:10, 2.0 mL/min, 140 bar, 40 °C. Retention times: 9.2 mins (minor), 9.8 mins (major), e.r. = 98:2. To facilitate analysis by SFC, the major diastereoisomer was separated from the minor diastereoisomer by FCC.

SFC analysis of the major diastereomer of racemate, prepared using rac-BINAP:

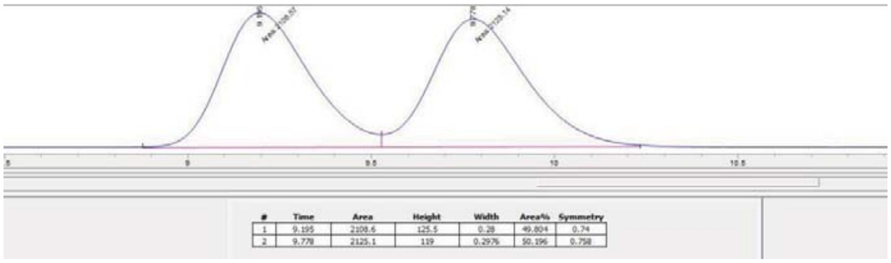

#	Time	Area	Height	Width	Area%	Symmetry
1	9.195	2108.6	125.5	0.28	49.804	0.74
2	9.778	2125.1	119	0.2976	50.196	0.758

SFC analysis of the major diastereomer of enantioenriched material, prepared using (R)-SEGPHOS:

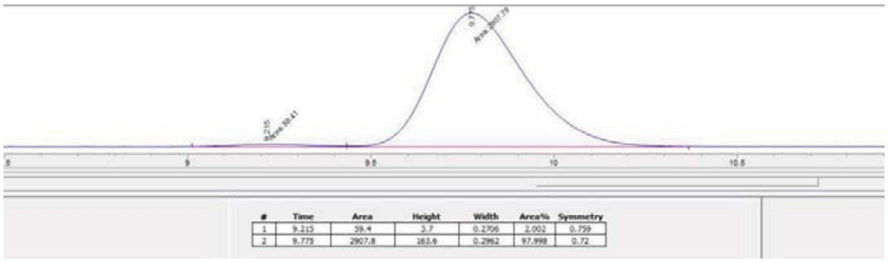

#	Time	Area	Height	Width	Area%	Symmetry
1	9.215	59.4	3.7	0.2706	2.002	0.759
2	9.775	2907.8	163.6	0.2962	97.998	0.72

(*2S,3S*)-3-([1,1'-Biphenyl]-4-yl)-*N,N*-dimethyl-2-(phenylamino)butanamide (3fj):

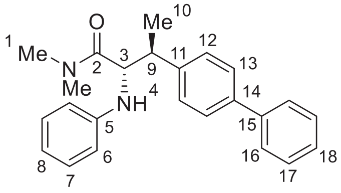

General procedure C: The reaction was carried out with substrate **1f** (17.8 mg, 0.10 mmol, 100 mol%) and 4-vinyl-1,1'-biphenyl **2j** (36.0 mg, 0.20 mmol, 200 mol%). Purification of the residue by FCC (hexane/EtOAc 70:30) afforded the title compound (34.4 mg, 96%, >30:1 B:L, d.r. >20:1 *a:b*, e.r. = 97:3) as a pale-yellow solid. ^{1}H NMR analysis of the crude material gave >30:1 B:L and d.r. = 4:1. **m.p.** = 103 – 105 °C (hexane/EtOAc); $[\boldsymbol{\alpha}]_{D}^{25}$ = -11.6 (c = 1.0 $CHCl_3$); **IR (thin film)** ν_{max}/cm^{-1}: 3330 (br), 3028 (s), 1638 (s), 1602 (s), 1499 (s), 1398 (s), 750 (s), 695 (s); **^{1}H NMR** (500 MHz, $CDCl_3$) Data for the major diastereomer *a* only: δ 7.60 – 7.54 (m, 4H, ArH), 7.46 – 7.43 (m, 2H, H^{17}), 7.36 – 7.32 (m, 3H, ArH), 7.18 – 7.15 (m, 2H, H^{7}), 6.73 – 6.70 (m, 1H, H^{8}), 6.66 (d, *J* = 7.5 Hz, 2H, H^{6}), 5.30 – 4.37 (m, 2H, H^{3} + H^{4}), 3.41 – 3.36 (m, 1H, H^{9}), 2.92 (s, 3H, H^{1}), 2.71 (s, 3H, H$^{1'}$), 1.41 (d, *J* = 7.0 Hz, 3H, H^{10}); **^{13}C NMR** (126 MHz, $CDCl_3$) Data for the major diastereomer *a* only: δ 171.5 (C^{2}), 146.8 (C^{5}), 141.6 (C^{11}), 140.7 (C^{15}), 139.8 (C^{14}), 129.3 (C^{7}), 128.7 (C^{17}), 128.3 (C^{12}), 127.2 (C^{18}), 127.1 (C^{16}), 127.0 (C^{13}), 118.2 (C^{8}), 114.1 (C^{6}), 58.3 (C^{3}), 42.0 (C^{9}), 37.0 (C^{1}), 35.7 (C$^{1'}$), 16.0 (C^{10}); **HRMS** (ESl): calculated for $C_{24}H_{26}N_2ONa$ $[M+Na]^+$ requires *m/z* 381.1937, found *m/z* 381.1948; **Chiral SFC:** DAICEL CHIRALCEL OD-H column (25 cm), CO_2:*i*-PrOH 90:10, 2.0 mL/min, 140 bar, 40 °C. Retention times: 19.7 mins (minor), 21.7 mins (major), e.r. = 97:3. To facilitate analysis by SFC, the major diastereoisomer was separated from the minor diastereoisomer by FCC.

SFC analysis of the major diastereomer of racemate, prepared using rac-BINAP:

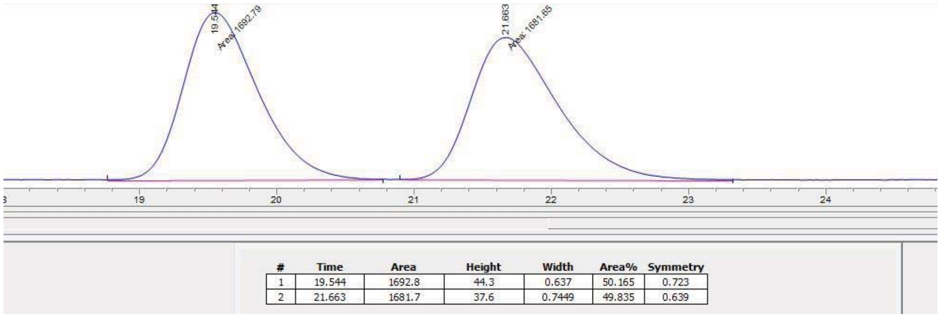

#	Time	Area	Height	Width	Area%	Symmetry
1	19.544	1692.8	44.3	0.637	50.165	0.723
2	21.663	1681.7	37.6	0.7449	49.835	0.639

SFC analysis of the major diastereomer of enantioenriched material, prepared using (R)-SEGPHOS:

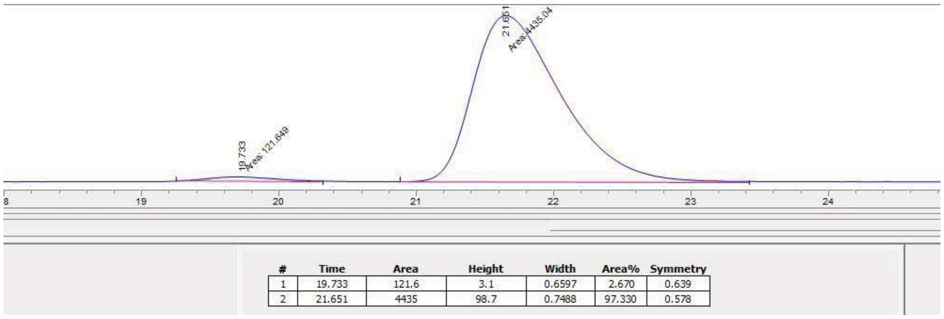

#	Time	Area	Height	Width	Area%	Symmetry
1	19.733	121.6	3.1	0.6597	2.670	0.639
2	21.651	4435	98.7	0.7488	97.330	0.578

(*2S,3S*)-*N,N*-Dimethyl-2-(phenylamino)-3-(4-(trimethylsilyl)phenyl)butanamide (3fk):

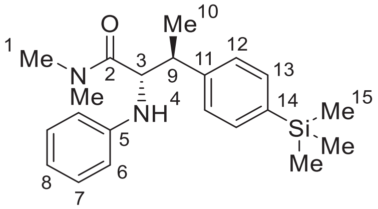

General procedure C: The reaction was carried out with substrate **1f** (17.8 mg, 0.10 mmol, 100 mol%) and trimethyl(4-vinylphenyl)silane **2k** (39.0 μL, 0.20 mmol, 200 mol%). Purification of the residue by FCC (hexane/EtOAc 70:30) afforded the title compound (27.7 mg, 78%, >30:1 B:L, d.r. = 7:1 *a:b*, e.r. = 95:5) as a pale-yellow solid. ^{1}H NMR analysis of the crude material gave >30:1 B:L and d.r. = 7:1. **m.p.** = 113 – 115 (hexane/EtOAc); $[\boldsymbol{\alpha}]_{\boldsymbol{D}}^{\mathbf{23}}$ = **-**3.0 (c = 1.0, $CHCl_3$); **IR (thin film)** ν_{max}/cm^{-1}: 3339 (br), 2955 (s), 1638 (s), 1641 (s), 1602 (s), 1501 (s), 1248 (s), 1112 (s), 838 (s); **^{1}H NMR** (500 MHz, $CDCl_3$) Data for the major diastereomer *a* only: δ 7.48 (d, *J* = 8.0 Hz, 2H, H^{13}), 7.26 (d, *J* = 8.0 Hz, 2H, H^{12}), 7.19 – 7.16 (m, 2H, H^7), 6.75 – 6.72 (m, 1H, H^8), 6.66 (d, *J* = 7.0 Hz, 2H, H^6), 4.60 – 4.59 (m, 2H, H^3 + H^4), 3.37 – 3.31 (m, 1H, H^9), 2.93 (s, 3H, H^1), 2.69 (s, 3H, $H^{1'}$), 1.40 (d, *J* = 7.0 Hz, 3H, H^{10}), 0.28 (s, 9H, H^{15}); **^{13}C NMR** (126 MHz, $CDCl_3$) Data for the major diastereomer *a* only: δ 171.6

(C^2), 146.8 (C^5), 143.0 (C^{11}), 138.9 (C^{14}), 133.5 (C^{13}), 129.3 (C^7), 127.4 (C^{12}), 118.2 (C^8), 114.1 (C^6), 58.3 (C^3), 42.4 (C^9), 36.9 (C^1), 35.7 ($C^{1'}$), 16.0 (C^{10}), -1.1 (C^{10}); **HRMS** (ESI): calculated for $C_{21}H_{31}N_2OSi$ $[M+H]^+$ requires *m/z* 355.2200, found *m/z* 355.2205; **Chiral SFC:** DAICEL CHIRALCEL SC column (25 cm), CO_2:*i*-PrOH 90:10, 2.0 mL/min, 170 bar, 40 °C. Retention times: 8.1 mins (minor), 10.8 mins (major), e.r. = 95:5. To facilitate analysis by SFC, the major diastereoisomer was separated from the minor diastereoisomer by FCC.

SFC analysis of the major diastereomer of racemate, prepared using rac-BINAP:

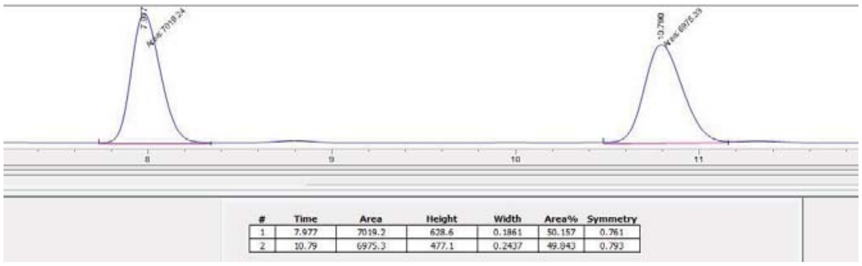

#	Time	Area	Height	Width	Area%	Symmetry
1	7.977	7019.2	628.6	0.1861	50.157	0.761
2	10.79	6975.3	477.1	0.2437	49.843	0.793

SFC analysis of the major diastereomer of enantioenriched material, prepared using (R)-SEGPHOS:

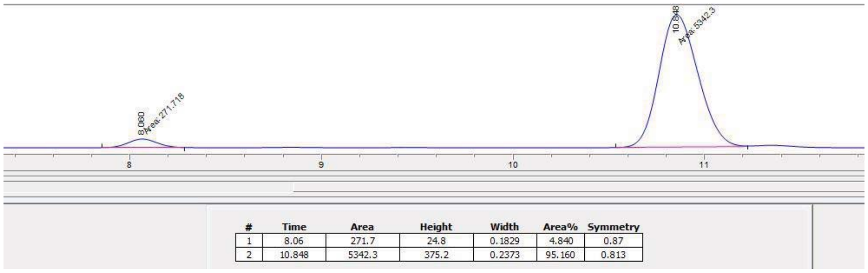

#	Time	Area	Height	Width	Area%	Symmetry
1	8.06	271.7	24.8	0.1829	4.840	0.87
2	10.848	5342.3	375.2	0.2373	95.160	0.813

(*2S,3S*)-*N,N*-Dimethyl-2-(phenylamino)-3-(*o*-tolyl)butanamide (3fl):

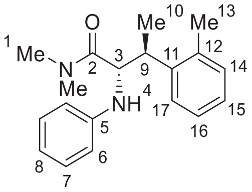

General procedure C: The reaction was carried out with substrate **1f** (17.8 mg, 0.10 mmol, 100 mol%) and 1-methyl-2-vinylbenzene **2l** (26.0 μL, 0.20 mmol, 200 mol%). Purification of the residue by FCC (hexane/EtOAc 60:40) afforded the title compound (21.9 mg, 74%, >30:1 B:L, d.r. = 13:1 *a:b*, e.r. = 98:2) as a pale-yellow solid. ^{1}H NMR analysis of the crude material gave >30:1 B:L and d.r. = 8:1. **m.p.** = 135 – 137 °C (hexane/EtOAc); $[\alpha]_D^{24}$ = +19.4 (c = 0.25, $CHCl_3$); **IR (thin film)** ν_{max}/cm^{-1}: 3681 (s), 3329 (br), 3052 (s), 3017 (s), 2967 (s), 2937 (s),

2873 (s), 2845 (s), 1635 (s), 1602 (s); **^{1}H NMR** (500 MHz, $CDCl_3$) Data for the major diastereomer *a*: δ 7.21 – 7.10 (m, 6H, $H^7 + H^{14} + H^{15} + H^{16} + H^{17}$), 6.73 – 6.70 (m, 1H, H^8), 6.65 (d, *J* = 7.5 Hz, 2H, H^6), 4.76 – 4.38 (m, 2H, $H^3 + H^4$), 3.66 – 3.61 (m, 1H, H^9), 2.86 (s, 3H, H^1), 2.56 (s, 3H, $H^{1'}$), 2.44 (s, 3H, H^{13}), 1.34 (d, *J* = 7.0 Hz, 3H, H^{10}). Characteristic signals for the minor diastereomer *b*: 2.71 (s, 2H, H^1), 2.62 (s, 2H, $H^{1'}$), 2.35 (s, 2H, H^{13}), 1.41 (d, *J* = 7.0 Hz, 2H, H^{10}); **^{13}C NMR** (126 MHz, $CDCl_3$) Data for the major diastereomer *a* only: δ 171.8 (C^2), 147.0 (C^5), 140.9 (C^{11}), 136.1 (C^{12}), 130.5 (C^{14}), 129.5 (C^7), 126.7 (ArC), 126.6 (ArC), 126.2 (ArC), 118.3 (C^8), 114.3 (C^6), 56.5 (C^3), 37.4 (C^9), 36.8 (C^1), 35.7 ($C^{1'}$), 19.9 (C^{13}), 15.9 (C^{10}); **HRMS** (ESI): calculated for $C_{19}H_{25}N_2O$ $[M+H]^+$ requires *m/z* 297.1961, found *m/z* 297.1964; **Chiral SFC:** DAICEL CHIRALCEL OD-H column (25 cm), CO_2:*i*-PrOH 92:8, 2.0 mL/min, 140 bar, 40 °C. Retention times: 8.6 mins (major), 9.7 mins (minor), e.r. = 98:2. To facilitate analysis by SFC, the major diastereoisomer was separated from the minor diastereoisomer by FCC.

SFC analysis of the racemates, prepared using rac-BINAP:

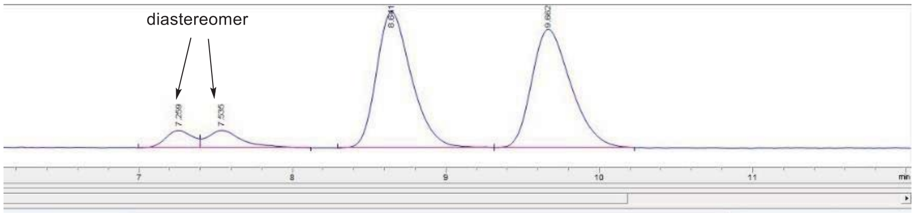

#	Time	Area	Height	Width	Area%	Symmetry
1	7.259	45.4	3.7	0.1821	4.497	0.952
2	7.535	58.2	3.7	0.2294	5.764	0.604
3	8.641	458.2	28.9	0.2449	45.404	0.71
4	9.662	447.4	25.1	0.2716	44.335	0.691

SFC analysis of the major diastereomer of enantioenriched material, prepared using (R)-SEGPHOS:

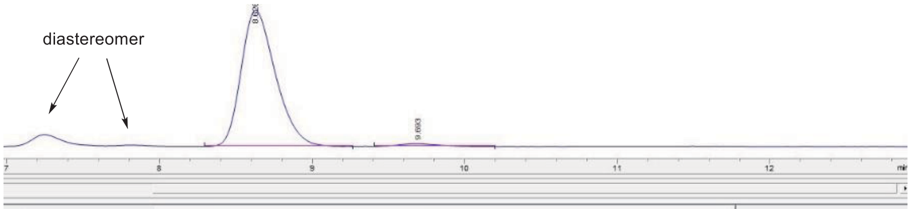

#	Time	Area	Height	Width	Area%	Symmetry
1	8.626	1236.1	78.6	0.2414	97.834	0.694
2	9.693	27.4	1.5	0.2241	2.166	0.923

(*2S,3S*)-3-(Benzofuran-5-yl)-*N,N*-dimethyl-2-(phenylamino)butanamide (3fm):

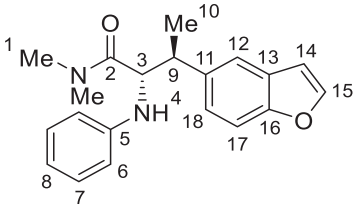

General procedure C: The reaction was carried out with substrate **1f** (17.8 mg, 0.10 mmol, 100 mol%) and 5-vinylbenzofuran **2m** (28.8 mg, 0.20 mmol, 200 mol%). Purification of the residue by FCC (hexane/EtOAc 70:30) afforded the title compound (25.5 mg, 79%, >30:1 B:L, d.r. = 8:1 *a:b*, e.r. = 92:8) as a pale-yellow oil. ^{1}H NMR analysis of the crude material gave >30:1 B:L and d.r. = 8:1; $[\boldsymbol{\alpha}]_{\boldsymbol{D}}^{\mathbf{23}}$ = -7.8 (c = 1.0, $CHCl_3$); **IR (thin film)** ν_{max}/cm^{-1}: 3334 (br), 2931 (s), 1637 (s), 1602 (s), 1499 (s), 1397 (s), 1113 (s), 745 (s); **^{1}H NMR** (500 MHz, $CDCl_3$) Data for the major diastereomer *a* only: δ 7.63 (d, *J* = 2.0 Hz, 1H, H^{15}), 7.51 (d, *J* = 2.0 Hz, 1H, H^{12}), 7.47 (d, *J* = 8.5 Hz, 1H, H^{17}), 7.22 – 7.16 (m, 3H, H^7 + H^{18}), 6.76 (d, *J* = 2.0 Hz, 1H, H^{14}), 6.75 – 6.72 (m, 1H, H^8), 6.67 (d, *J* = 7.5 Hz, 2H, H^6), 4.97 – 4.40 (m, 2H, H^3 + H^4), 3.49 – 3.44 (m, 1H, H^9), 2.92 (s, 3H, H^1), 2.66 (s, 3H, $H^{1'}$), 1.45 (d, *J* = 7.0 Hz, 3H, H^{10}); **^{13}C NMR** (126 MHz, $CDCl_3$) Data for the major diastereomer *a* only: δ 171.6 (C^2), 154.1 (C^{16}), 146.9 (C^5), 145.4 (C^{11}), 137.0 (C^{15}), 129.3 (C^7), 127.6 (C^{13}), 124.2 (C^{18}), 120.2 (C^{12}), 118.2 (C^8), 114.1 (C^6), 111.2 (C^{17}), 106.5 (C^{14}), 58.7 (C^3), 42.2 (C^9), 37.0 (C^1), 35.7 ($C^{1'}$), 16.6 (C^{10}); **HRMS** (ESI): calculated for $C_{20}H_{23}N_2O_2$ $[M+H]^+$ requires *m/z* 323.1754, found *m/z* 323.1757; **Chiral SFC:** DAICEL CHIRALCEL SC column (25 cm), CO_2:*i*-PrOH 80:20, 2.0 mL/min, 180 bar, 40 °C. Retention times: 6.4 mins (minor), 7.4 mins (major), e.r. = 92:8. To facilitate analysis by SFC, the major diastereoisomer was separated from the minor diastereoisomer by FCC.

SFC analysis of the major diastereomer of racemate, prepared using rac-BINAP:

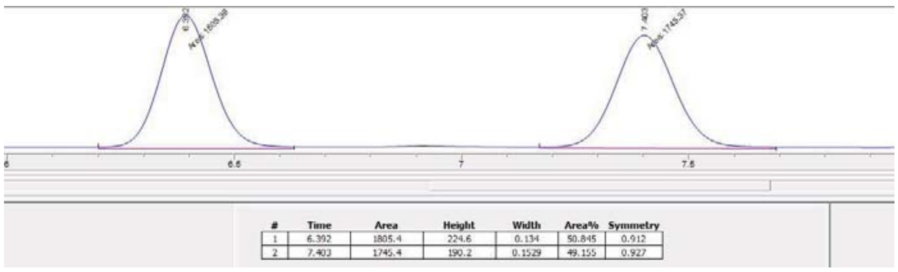

#	Time	Area	Height	Width	Area%	Symmetry
1	6.392	1805.4	224.6	0.134	50.845	0.912
2	7.403	1745.4	190.2	0.1529	49.155	0.927

SFC analysis of the major diastereomer of enantioenriched material, prepared using (R)-SEGPHOS:

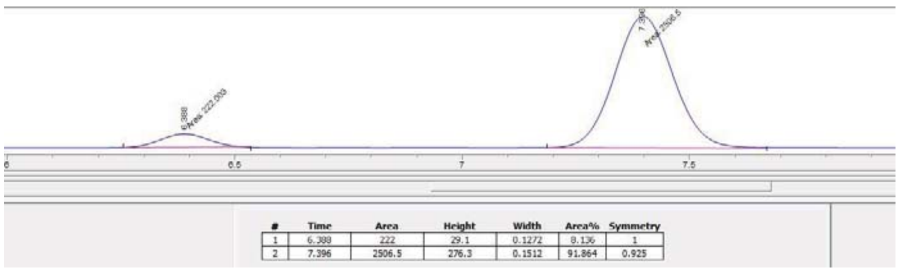

#	Time	Area	Height	Width	Area%	Symmetry
1	6.388	222	29.1	0.1272	8.136	1
2	7.396	2506.5	276.3	0.1512	91.864	0.925

(*2S,3S*)-3-(2-Chlorophenyl)-*N,N*-dimethyl-2-(phenylamino)butanamide (3fn):

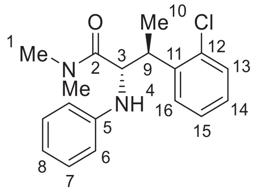

General procedure C: The reaction was carried out with substrate **1f** (17.8 mg, 0.10 mmol, 100 mol%) and 1-chloro-2-vinylbenzene **2n** (25.4 µL, 0.20 mmol, 200 mol%). Purification of the residue by FCC (hexane/EtOAc 70:30) afforded the title compound (11.7 mg, 37%, >30:1 B:L, d.r. >20:1 *a:b*, e.r. = 98:2) as a pale-yellow oil. ^{1}H NMR analysis of the crude material gave >30:1 B:L and d.r. = 7:1. $[\boldsymbol{\alpha}]_{\boldsymbol{D}}^{\mathbf{25}}$ = +19.7 (c = 1.0 $CHCl_3$); **IR (thin film)** ν_{max}/cm^{-1}: 3344 (br), 2933 (s), 1639 (s), 1603 (s), 1504 (s), 752 (s), 693 (s); **^{1}H NMR** (500 MHz, $CDCl_3$) Data for the major diastereomer *a* only: δ 7.36 (d, *J* = 9.5 Hz, 1H, H^{13}), 7.29 – 7.27 (m, 1H, H^{16}), 7.24 – 7.15 (m, 4H, $H^7 + H^{14} + H^{15}$), 6.77 (d, *J* = 8.0 Hz, 2H, H^6), 6.75 – 6.72 (m, 1H, H^8), 5.24 – 4.50 (m, 2H, $H^3 + H^4$), 3.99 – 3.94 (m, 1H, H^9), 2.80 (s, 3H, H^1), 2.55 (s, 3H, $H^{1'}$), 1.39 (d, *J* = 7.0 Hz, 3H, H^{10}); **^{13}C NMR** (126 MHz, $CDCl_3$) Data for the major diastereomer *a* only: δ 170.8 (C^2), 146.5 (C^5), 139.6 (C^{11}), 134.2 (C^{12}), 129.4 (ArC), 129.3 (C^7), 128.8 (ArC), 128.0 (ArC), 126.8 (ArC), 118.2 (C^8), 114.2 (C^6), 55.2 (C^3), 37.5 (C^9), 36.6 (C^1), 35.4 ($C^{1'}$), 14.7 (C^{10}); **HRMS** (ESl): calculated for $C_{18}H_{21}ClN_2ONa$ $[M+Na]^+$ requires *m/z* 339.1235, found *m/z* 339.1250; **Chiral SFC:** YMC Chiral ART Cellulose-SB column (25 cm), CO_2:*i*-PrOH 90:10, 2.0 mL/min, 140 bar, 40 °C. Retention times: 7.1 mins (major), 7.9 mins (minor), e.r. = 98:2. To facilitate analysis by SFC, the major diastereoisomer was separated from the minor diastereoisomer by FCC.

SFC analysis of the major diastereomer of racemate, prepared using rac-BINAP:

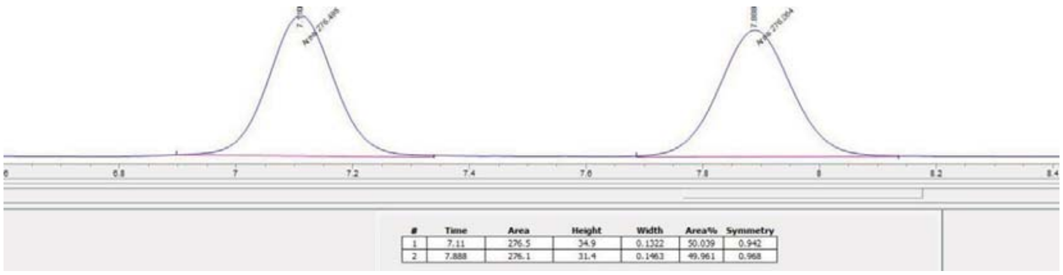

#	Time	Area	Height	Width	Area%	Symmetry
1	7.11	276.5	34.9	0.1322	50.039	0.942
2	7.888	276.1	31.4	0.1463	49.961	0.968

SFC analysis of the major diastereomer of enantioenriched material, prepared using (R)-SEGPHOS:

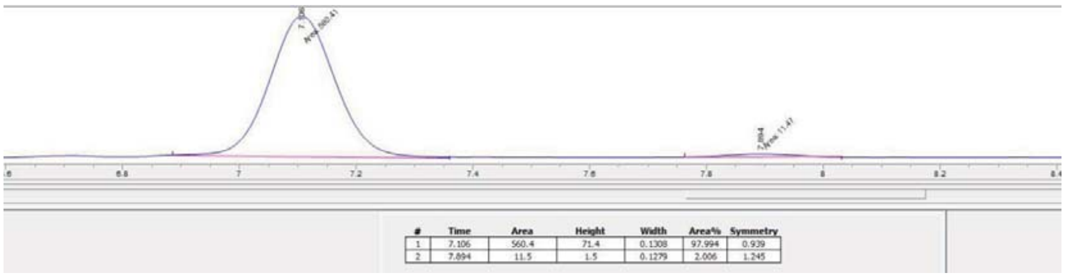

#	Time	Area	Height	Width	Area%	Symmetry
1	7.106	560.4	71.4	0.1308	97.994	0.939
2	7.894	11.5	1.5	0.1279	2.006	1.245

(*2S,3S*)-*N,N*-Dimethyl-3-(perfluorophenyl)-2-(phenylamino)butanamide (3fo):

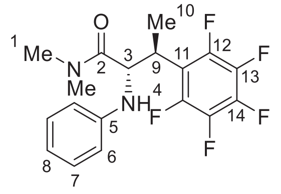

General procedure C: The reaction was carried out with substrate **1f** (17.8 mg, 0.10 mmol, 100 mol%) and 1,2,3,4,5-pentafluoro-6-vinylbenzene **2o** (27.0 µL, 0.20 mmol, 200 mol%). Purification of the residue by FCC (hexane/EtOAc 70:30) afforded the title compound (27.5 mg, 74%, >30:1 B:L, d.r. = 2:1 *a:b*, e.r. = 98.5:1.5) as a pale-yellow oil. ^{1}H NMR analysis of the crude material gave >30:1 B:L and d.r. = 2:1. $[\boldsymbol{\alpha}]_{\boldsymbol{D}}^{\mathbf{21}}$ = -95.3 (c = 0.13, $CHCl_3$); **IR (thin film)** ν_{max}/cm^{-1}: 3323 (br), 3058 (s), 3031 (s), 2979 (s), 2940 (s), 2887 (s), 1643 (s), 1602 (s), 1521 (s); **^{1}H NMR** (500 MHz, $CDCl_3$) Data for diastereomers *a* and *b*: δ 7.18 – 7.15 (m, 0.8H, H^{7b}), δ 7.10 – 7.07 (m, 1.2H, H^{7a}), 6.75 – 6.72 (m, 0.4H, H^{8b}), 6.71 – 6.68 (m, 0.6H, H^{8a}), 6.65 (d, *J* = 8.0 Hz, 0.7H, H^{6b}), 6.54 (d, *J* = 8.0 Hz, 1.2H, H^{6a}), 4.83 – 4.79 (m, 1H, H^3), 3.72 – 3.63 (m, 1H, H^9), 3.18 (s, 1.8H, H^{1a}), 2.99 (s, 1.8H, $H^{1a'}$), 2.96 (s, 1H, H^{1b}), 2.80 (s, 1H, $H^{1b'}$), 1.46 (d, *J* = 7.0 Hz, 1.2H, H^{10b}), 1.34 (d, *J* = 7.0 Hz, 1.9H, H^{10a}); **^{13}C NMR** (126 MHz, $CDCl_3$) Data for diastereomers *a* and *b*: δ 172.6 (C^{2a}), 171.7 (C^{2b}), 147.2 (C^5), 146.9 (C^{11}), 129.6 (C^{7b}), 129.5 (C^{7a}), 119.3 (C^{8a}), 118.7 (C^{8b}), 114.6 (C^{6a}), 113.7 (C^{6b}), 56.4 (C^3), 37.7 (C^{1a}), 37.3 (C^{1b}), 36.0 ($C^{1a'}$), 35.9 ($C^{1b'}$), 35.1 (C^{9a}), 34.9 (C^{9b}), 15.8 (C^{10b}), 15.7 (C^{10a}); **^{19}F NMR** (471 MHz, $CDCl_3$)

Data for diastereomers *a* and *b*: δ -141.34 (dd, *J* = 22.5, 7.5 Hz, C^{12}-F^b), -142.52 (dd, *J* = 22.5, 7.5 Hz, C^{12}-F^b), -156.28 (t, *J* = 21.0 Hz, C^{14}-F^b), -156.61 (t, *J* = 21.0 Hz, C^{14}-F^a), -161.85 (td, *J* = 22.5, 7.5 Hz, C^{13}-F^b), -162.45 (td, *J* = 22.5, 7.5 Hz, C^{13}-F^a); **HRMS** (ESI): calculated for $C_{18}H_{18}F_5N_2O$ $[M+H]^+$ requires *m/z* 373.1334, found *m/z* 373.1333; **Chiral SFC:** DAICEL CHIRALPAK IE column (25 cm), CO_2:*i*-PrOH 98:2, 3.5 mL/min, 140 bar, 40 °C. Retention times: 15.0 mins (minor), 15.8 mins (major), e.r. = 98.5:1.5. To facilitate analysis by SFC, the major diastereoisomer was separated from the minor diastereoisomer by FCC.

SFC analysis of the major diastereomer of racemate, prepared using rac-BINAP:

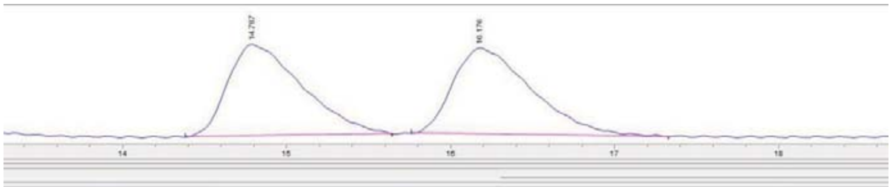

#	Time	Area	Height	Width	Area%	Symmetry
1	14.787	433.5	13.6	0.3966	50.210	0.465
2	16.176	429.9	12.8	0.4226	49.790	0.493

SFC analysis of the major diastereomer of enantioenriched material, prepared using (R)-SEGPHOS:

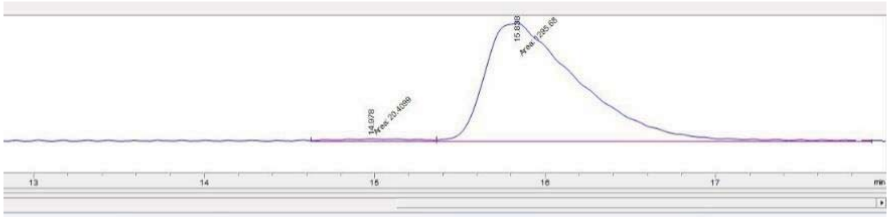

#	Time	Area	Height	Width	Area%	Symmetry
1	14.978	20.4	6.9E-1	0.4957	1.551	0
2	15.838	1295.7	33.5	0.6445	98.449	0.5

((*2S,3S*)-2-((4-Hydroxyphenyl)amino)-*N,N*-dimethyl-3-(1-(methyl-(λ¹-oxidaneyl)-(*p*-tolyl)sulfinyl)-1*H*-indol-3-yl)butanamide (3pf):

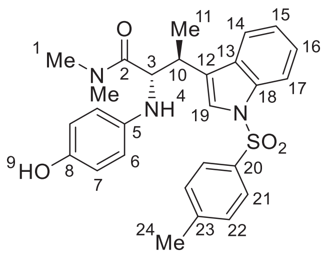

General procedure C: The reaction was carried out with substrate **1p** (19.4 mg, 0.10 mmol, 100 mol%) and 1-(methyl-(λ¹-oxidaneyl)-(*p*-tolyl)sulfinyl)-3-vinyl-1*H*-indole **2f** (62.4 mg,

0.20 mmol, 200 mol%). Purification of the residue by FCC (hexane/EtOAc 35:65) afforded the title compound (46.2 mg, 94%, >30:1 B:L, d.r. >20:1 *a:b*, e.r. = 95.5:4.5) as a pale-yellow solid. ^{1}H NMR analysis of the crude material gave >30:1 B:L and d.r. = 5:1. **m.p.** = 96 – 98 °C (hexane/EtOAc); $[\alpha]_D^{25}$ = +2.8 (c = 1.0 $CHCl_3$); **IR (thin film)** ν_{max}/cm^{-1}: 3348 (br), 3053 (s), 1628 (s), 1515 (s), 1447 (s), 1173 (s), 1125 (s), 747 (s), 671 (s); **^{1}H NMR** (500 MHz, $CDCl_3$) Data for the major diastereomer *a* only: δ 7.98 (d, *J* = 8.0 Hz, 1H, ArH), 7.68 (d, *J* = 8.0 Hz, 2H, H^{21}), 7.52 (d, *J* = 8.0 Hz, 1H, ArH), 7.40 (s, 1H, H^{19}), 7.32 – 7.28 (m, 1H, ArH), 7.25 – 7.22 (m, 1H, ArH), 7.14 (d, *J* = 8.0 Hz, 2H, H^{22}), 6.60 (d, *J* = 8.0 Hz, 2H, H^{7}), 6.47 (d, *J* = 8.0 Hz, 2H, H^{6}), 6.00 (br s, 1H, H^{9}), 4.52 (d, *J* = 6.0 Hz, 1H, H^{3}), 4.15 (br s, 1H, H^{4}), 3.51 – 3.46 (m, 1H, H^{10}), 2.81 (s, 3H, H^{1}), 2.63 (s, 3H, $H^{1'}$), 2.29 (s, 3H, H^{24}), 1.41 (d, *J* = 7.0 Hz, 3H, H^{11}); **^{13}C NMR** (126 MHz, $CDCl_3$) Data for the major diastereomer *a* only: δ 172.3 (C^{2}), 149.1 (C^{8}), 144.9 (C^{5}), 140.5 (ArC), 135.17 (ArC), 135.16 (ArC), 130.5 (ArC), 129.8 (C^{22}), 126.6 (C^{21}), 124.8 (ArC), 124.3 (ArC), 123.4 (ArC), 123.2 (ArC), 119.4 (ArC), 116.4 (C^{7}), 116.2 (C^{6}), 113.9 (C^{12}), 58.8 (C^{3}), 37.1 (C^{1}), 35.8 ($C^{1'}$), 33.9 (C^{10}), 21.5 (C^{24}), 16.3 (C^{11}); **HRMS** (ESl): calculated for $C_{27}H_{30}N_3O_4S$ $[M+H]^+$ requires *m/z* 492.1952, found *m/z* 492.1966; **Chiral SFC:** DAICEL CHIRALCEL OD-H column (25 cm), CO_2:*i*-PrOH 80:20, 2.0 mL/min, 170 bar, 40 °C. Retention times: 13.9 mins (major), 16.3 mins (minor), e.r. = 95.5:4.5. To facilitate analysis by SFC, the major diastereoisomer was separated from the minor diastereoisomer by FCC.

SFC analysis of the major diastereomer of racemate, prepared using rac-BINAP:

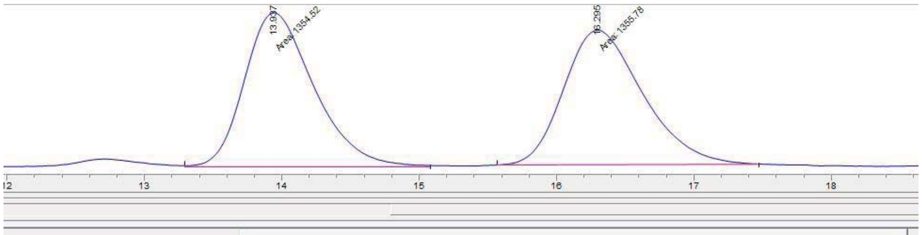

#	Time	Area	Height	Width	Area%	Symmetry
1	13.937	1354.5	38.2	0.5903	49.977	0.721
2	16.295	1355.8	33.5	0.6736	50.023	0.747

SFC analysis of the major diastereomer of enantioenriched material, prepared using (R)-SEGPHOS:

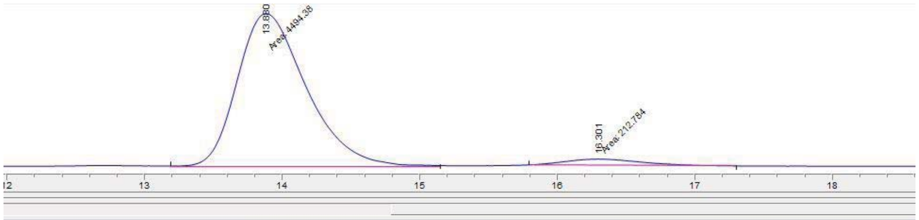

#	Time	Area	Height	Width	Area%	Symmetry
1	13.88	4494.4	127.1	0.5893	95.480	0.688
2	16.301	212.8	5.3	0.6686	4.520	0.681

(*2S,3S*)-3-(3-Chlorophenyl)-2-((4-hydroxyphenyl)amino)-1-morpholinobutan-1-one (3wk):

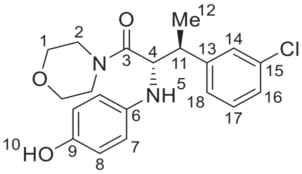

General procedure C: The reaction was carried out with substrate **1w** (23.6 mg, 0.10 mmol, 100 mol%) and 1-chloro-3-vinylbenzene **2k** (25.0 µL, 0.20 mmol, 200 mol%). Purification of the residue by FCC (hexane/EtOAc 35:65) afforded the title compound (29.9 mg, 80%, >30:1 B:L, d.r. >20:1 *a:b*, e.r. = 97:3) as a pale-yellow oil. ^{1}H NMR analysis of the crude material gave >30:1 B:L and d.r. = 5:1. $[\alpha]_D^{25}$ = -9.8 (c = 1.0 $CHCl_3$); **IR (thin film)** ν_{max}/cm^{-1}: 3327 (br), 2968 (s), 1624 (s), 1515 (s), 1432 (s), 1224 (s), 1114 (s), 1033 (s), 827 (s), 783 (s); **^{1}H NMR** (500 MHz, $CDCl_3$) Data for the major diastereomer *a* only: δ 7.24 – 7.21 (m, 3H, H^{14} + H^{16} + H^{17}), 7.14 – 7.12 (m, 1H, H^{18}), 6.63 (d, *J* = 8.0 Hz, 2H, H^8), 6.53 (d, *J* = 8.0 Hz, 2H, H^7), 5.93 (br s, 1H, H^{10}), 4.68 – 3.99 (m, 2H, H^4 + H^5), 3.63 – 3.52 (m, 4H, H^1 + H^2), 3.40 – 3.13 (m, 5H, $H^{1'}$ + $H^{2'}$ + H^{11}), 1.35 (d, *J* = 7.0 Hz, 3H, H^{12}); **^{13}C NMR** (126 MHz, $CDCl_3$) Data for the major diastereomer *a* only: δ 170.7 (C^3), 149.6 (C^9), 144.5 (C^6), 139.7 (C^{13}), 134.4 (C^{15}), 129.8 (ArC), 127.9 (ArC), 127.2 (ArC), 126.2 (C^{18}), 117.0 (C^8), 116.3 (C^7), 66.7 (C^1), 66.2 ($C^{1'}$), 60.2 (C^4), 46.2 (C^2), 42.5 ($C^{2'}$), 42.1 (C^{11}), 16.5 (C^{12}); **HRMS** (ESl): calculated for $C_{20}H_{24}ClN_2O_3$ $[M+H]^+$ requires *m/z* 375.1470, found *m/z* 375.1479; **Chiral SFC:** YMC Chiral ART Cellulose-SC column (25 cm), CO_2:*i*-PrOH 80:20, 2.0 mL/min, 170 bar, 40 °C. Retention times: 7.5 mins (minor), 7.9 mins (major), e.r. = 97:3. To facilitate analysis by SFC, the major diastereoisomer was separated from the minor diastereoisomer by FCC.

SFC analysis of the major diastereomer of racemate, prepared using rac-BINAP:

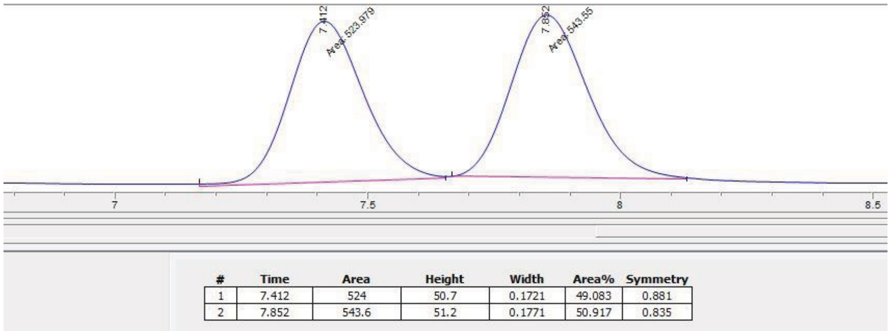

#	Time	Area	Height	Width	Area%	Symmetry
1	7.412	524	50.7	0.1721	49.083	0.881
2	7.852	543.6	51.2	0.1771	50.917	0.835

SFC analysis of the major diastereomer of enantioenriched material, prepared using (R)-SEGPHOS:

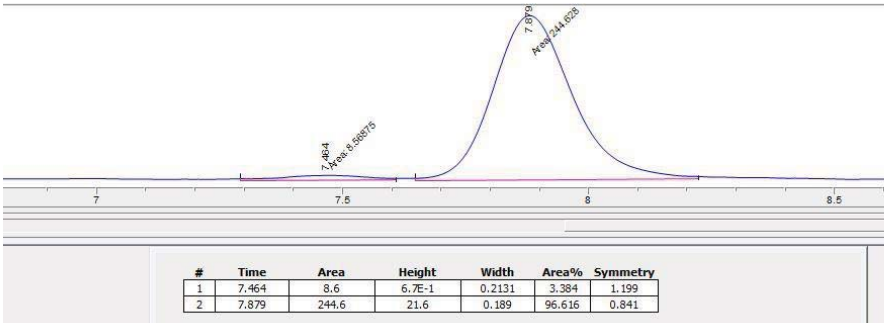

#	Time	Area	Height	Width	Area%	Symmetry
1	7.464	8.6	6.7E-1	0.2131	3.384	1.199
2	7.879	244.6	21.6	0.189	96.616	0.841

(*2R,3R*)-*N,N*-Dimethyl-3-phenyl-2-(phenylamino)butanamide (*ent*-3fa):

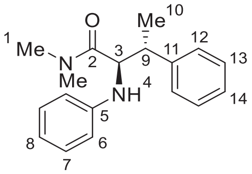

General procedure C: The reaction was carried out with substrate **1f** (17.8 mg, 0.10 mmol, 100 mol%), styrene (23.0 µL, 0.20 mmol, 200 mol%) and (*S*)-SEGPHOS (3.05 mg, 5.00 µmol, 5 mol%). Purification of the residue by FCC (hexane/EtOAc 70:30) afforded the title compound (25.4 mg, 90%, >30:1 B:L, d.r. >20:1 *a:b*, e.r. = 2.5:97.5) as a pale-yellow solid. ^{1}H NMR analysis of the crude material gave >30:1 B:L and d.r. = 9:1. **m.p.** = 101 – 103 °C (hexane/EtOAc); $[\alpha]_D^{25}$ = +8.8 (c = 1.0 $CHCl_3$); **Chiral SFC:** YMC Chiral ART Cellulose-SC column (25 cm), CO_2:*i*-PrOH 80:20, 2.0 mL/min, 170 bar, 40 °C. Retention times: 4.9 mins (major), 5.9 mins (minor), e.r. = 2.5:97.5. To facilitate analysis by SFC, the major diastereoisomer was separated from the minor diastereoisomer by FCC. All other analytical data was identical to that listed earlier for **3fa**.

SFC analysis of the major diastereomer of racemate, prepared using rac-BINAP:

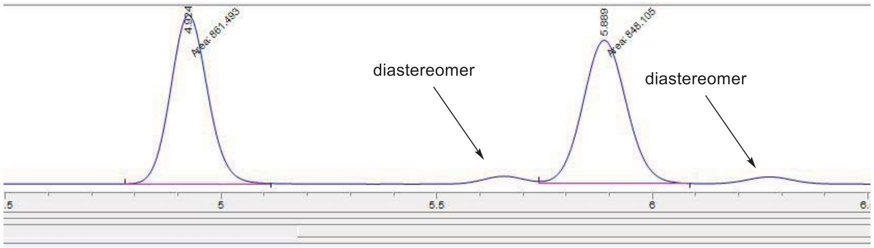

#	Time	Area	Height	Width	Area%	Symmetry
1	4.924	861.5	141.1	0.1018	50.392	0.912
2	5.889	848.1	119	0.1188	49.608	0.947

SFC analysis of the major diastereomer of enantioenriched material, prepared using (S)-SEGPHOS:

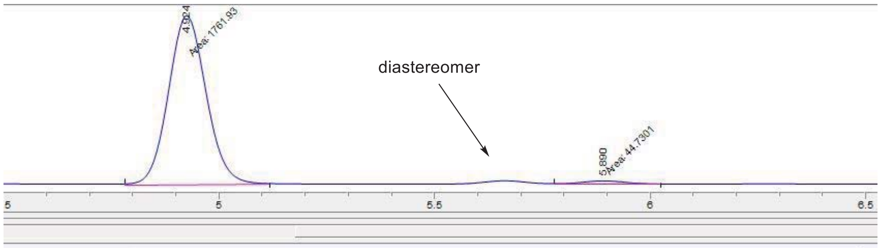

#	Time	Area	Height	Width	Area%	Symmetry
1	4.924	1761.9	285.1	0.103	97.524	0.911
2	5.89	44.7	5.8	0.1285	2.476	0.897

(*4S,5S*)-2,2,5-Trimethyl-4-(phenylamino)nonan-3-one (3op):

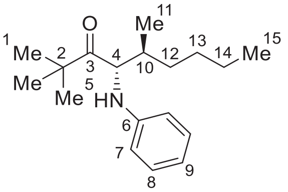

General procedure C: The reaction was carried out with substrate **1o** (19.1 mg, 0.10 mmol, 100 mol%), hex-1-ene **2p** (125 μL, 1.00 mmol, 1000 mol%), Ir(cod)$_2$BARF (12.7 mg, 0.01 mmol, 10 mol%) and (*R*)-DM-SEGPHOS (7.23 mg, 0.01 mmol, 10 mol%) and was run in mesitylene (0.2 mL) at 110 °C. Purification of the residue by FCC (hexane/EtOAc 90:10) afforded the title compound (20.6 mg, 75%, >30:1 B:L, d.r. >20:1 *a:b*, e.r. = 94:6) as a pale-yellow oil. ^{1}H NMR analysis of the crude material gave >30:1 B:L and d.r. = 5:1. $[\boldsymbol{\alpha}]_{\boldsymbol{D}}^{\boldsymbol{25}}$ = +9.9 (c = 1.0 $CHCl_3$); **IR (thin film)** ν_{max}/cm^{-1}: 3381 (br), 2960 (s), 2929 (s), 1699 (s), 1601 (s), 1513 (s), 1497 (s), 745 (s), 690 (s); **^{1}H NMR** (500 MHz, $CDCl_3$) Data for the major diastereomer *a* only: δ 7.16 – 7.12 (m, 2H, H^8), 6.71 – 6.68 (s, 1H, H^9), 6.63 (d, *J* = 7.5 Hz, 2H, H^7), 4.53 – 3.93 (m, 2H, H^4 + H^5), 1.96 – 1.88 (m, 1H, H^{10}), 1.42 – 1.26 (m, 6H, H^{12} + H^{13} + H^{14}), 1.14 (s, 9H, H^1), 0.91 (t, *J* = 7.0 Hz, 3H, H^{15}), 0.86 (d, *J* = 7.0 Hz, 3H, H^{11}); **^{13}C NMR**

(126 MHz, $CDCl_3$) Data for the major diastereomer *a* only: δ 216.2 (C^3), 148.0 (C^6), 129.3 (C^8), 118.0 (C^9), 114.2 (C^7), 60.9 (C^4), 43.4 (C^2), 35.4 (C^{10}), 34.4 (C^{12}), 29.7 (C^{13}), 26.8 (C^1), 22.8 (C^{14}), 14.1 (C^{11}), 13.9 (C^{15}); **HRMS** (ESl): calculated for $C_{18}H_{30}NO$ $[M+H]^+$ requires m/z 276.2322, found m/z 276.2334; **Chiral SFC:** YMC Chiral ART Cellulose-SC column (25 cm), CO_2:*i*-PrOH 97:3, 1.0 mL/min, 140 bar, 40 °C. Retention times: 6.1 mins (major), 6.5 mins (minor), e.r. = 94:6. To facilitate analysis by SFC, the major diastereoisomer was separated from the minor diastereoisomer by FCC.

SFC analysis of the major diastereomer of racemate, prepared using rac-BINAP:

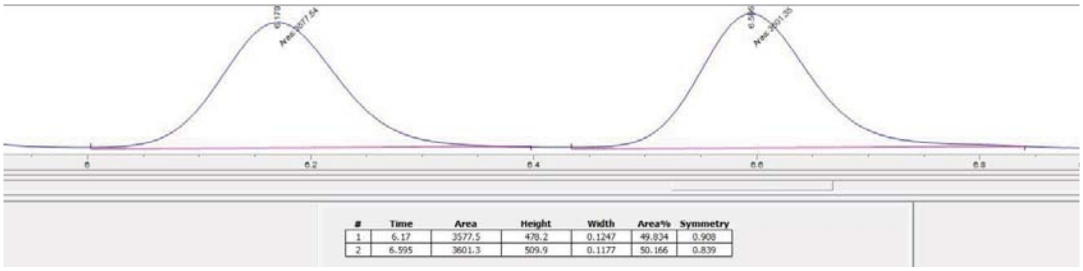

#	Time	Area	Height	Width	Area%	Symmetry
1	6.17	3577.5	478.2	0.1247	49.834	0.908
2	6.595	3601.3	509.9	0.1177	50.166	0.839

SFC analysis of the major diastereomer of enantioenriched material, prepared using (R)-DM-SEGPHOS:

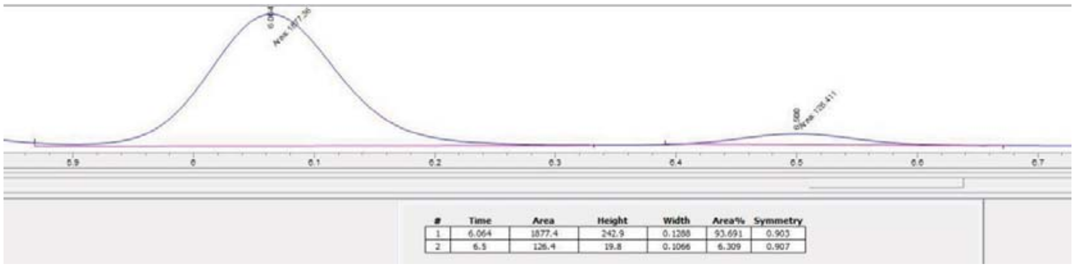

#	Time	Area	Height	Width	Area%	Symmetry
1	6.064	1877.4	242.9	0.1288	93.691	0.903
2	6.5	126.4	19.8	0.1066	6.309	0.907

(*4S,5S*)-2,2,5,7-Tetramethyl-4-(phenylamino)octan-3-one (3oq):

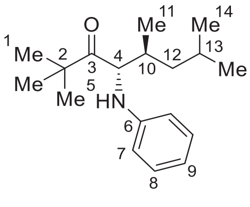

General procedure C: The reaction was carried out with substrate **1o** (19.1 mg, 0.10 mmol, 100 mol%), 4-methylpent-1-ene **2q** (127 μL, 1.00 mmol, 1000 mol%), $Ir(cod)_2BARF$ (12.7 mg, 0.01 mmol, 10 mol%) and (*R*)-DM-SEGPHOS (7.23 mg, 0.01 mmol, 10 mol%) and was run in mesitylene (0.2 mL) at 110 °C. Purification of the residue by FCC (hexane/EtOAc 90:10) afforded the title compound (18.7 mg, 68%, >30:1 B:L, d.r. = 2:1 *a:b*, e.r. = 90:10 as a pale-

yellow oil. ^{1}H NMR analysis of the crude material gave >30:1 B:L and d.r. = 2:1. $[\alpha]_D^{25}$ = +1.5 (c = 1.0 $CHCl_3$); **IR (thin film)** ν_{max}/cm^{-1}: 3380 (br), 2958 (s), 2971 (s), 1699 (s), 1601 (s), 1498 (s), 747 (s), 691 (s); **^{1}H NMR** (500 MHz, $CDCl_3$) Data for diastereomers *a* and *b*: δ 7.16 – 7.12 (m, 2H, H^8), 6.71 – 6.68 (m, 1H, H^9), 6.62 (d, *J* = 7.5 Hz, 2H, H^7), 4.42 – 4.30 (m, 1H, H^4), 4.18 – 4.07 (m, 1H, H^5), 2.05 – 1.97 (m, 1H, H^{10}), 1.75 – 1.66 (m, 1H, H^{13}), 1.28 – 1.25 (m, 2H, H^{12}), 1.14 – 1.13 (m, 9H, H^1), 0.99 (d, *J* = 6.5 Hz, 1H, H^{14b}), 0.94 (d, *J* = 6.5 Hz, 2H, H^{14a}), 0.89 (d, *J* = 6.5 Hz, 3H, H^{11}), 0.84 (d, *J* = 6.5 Hz, 2H, $H^{14a'}$), 0.80 (d, *J* = 6.5 Hz, 1H, $H^{14b'}$); **^{13}C NMR** (126 MHz, $CDCl_3$) Data for diastereomers *a* and *b*: δ 216.2 (C^{3b}), 216.1 (C^{3a}), 148.1 (C^{6a}), 147.9 (C^{6b}), 129.3 (C^8), 118.0 (C^{9a}), 117.8 (C^{9b}), 114.1 (C^{7a}), 113.8 (C^{7b}), 62.3 (C^{4b}), 61.4 (C^{4a}), 44.3 (C^{2a}), 43.3 (C^{2b}), 39.8 (C^{10}), 33.6 (C^{13b}), 33.0 (C^{13a}), 26.73 (C^{1a}), 26.66 (C^{1b}), 25.24 (C^{12a}), 25.17 (C^{12b}), 24.3 (C^{14b}), 23.5 (C^{14a}), 21.9 ($C^{14a'}$), 21.1 ($C^{14b'}$), 17.5 (C^{11b}), 13.7 (C^{11a}); **HRMS** (ESI): calculated for $C_{18}H_{30}NO$ $[M+H]^+$ requires m/z 276.2322, found m/z 276.2331; **Chiral SFC:** YMC Chiral ART Cellulose-SC column (25 cm), CO_2:*i*-PrOH 97:3, 1.0 mL/min, 140 bar, 40 °C. Retention times: 5.8 mins (major), 6.1 mins (minor), e.r. = 90:10. To facilitate analysis by SFC, the major diastereoisomer was separated from the minor diastereoisomer by FCC.

SFC analysis of the major diastereomer of racemate, prepared using rac-BINAP:

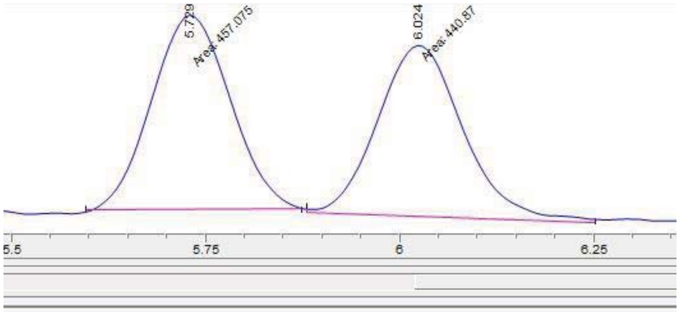

#	Time	Area	Height	Width	Area%	Symmetry
1	5.729	457.1	65.1	0.117	50.902	0.937
2	6.024	440.9	57.4	0.128	49.098	0.892

SFC analysis of the major diastereomer of enantioenriched material, prepared using (R)-DM-SEGPHOS:

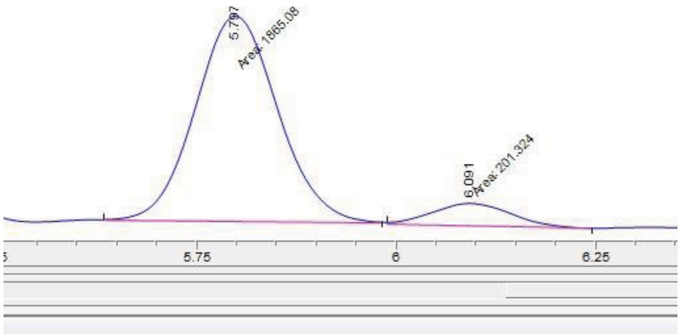

#	Time	Area	Height	Width	Area%	Symmetry
1	5.797	1865.1	258.6	0.1202	90.257	0.925
2	6.091	201.3	28.7	0.117	9.743	0.845

(*4S,5S*)-2,2,5,6-Tetramethyl-4-(phenylamino)heptan-3-one (3or):

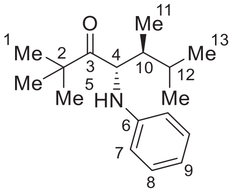

General procedure C: The reaction was carried out with substrate **1o** (19.1 mg, 0.10 mmol, 100 mol%), 3-methylbut-1-ene **2r** (129 µL, 1.00 mmol, 1000 mol%), Ir(cod)$_2$BARF (12.7 mg, 0.01 mmol, 10 mol%) and (*R*)-DM-SEGPHOS (7.23 mg, 0.01 mmol, 10 mol%) and was run in mesitylene (0.2 mL) at 110 °C. Purification of the residue by FCC (hexane/EtOAc 90:10) afforded the title compound (11.8 mg, 45%, >30:1 B:L, d.r. >20:1 *a:b*, e.r. = 71:29) as a pale-yellow oil. ^{1}H NMR analysis of the crude material gave >30:1 B:L and d.r. = 16:1. $[\boldsymbol{\alpha}]_D^{25}$ = +20.6 (c = 1.0 CHCl$_3$); **IR (thin film)** ν_{max}/cm^{-1}: 3374 (br), 2958 (s), 1699 (s), 1602 (s), 1498 (s), 749 (s), 566 (s); **^{1}H NMR** (500 MHz, CDCl$_3$) Data for the major diastereomer *a* only: δ 7.16 – 7.12 (m, 2H, H^8), 6.71 – 6.68 (m, 1H, H^9), 6.62 (d, *J* = 7.5 Hz, 2H, H^7), 4.66 (d, *J* = 3.0 Hz, 1H, H^4), 4.09 (br s, 1H, H^5), 1.75 – 1.64 (m, 2H, H^{10} + H^{12}), 1.15 (s, 9H, H^1), 1.07 (d, *J* = 6.0 Hz, 3H, H^{13}), 0.95 (d, *J* = 6.0 Hz, 3H, H$^{13'}$), 0.84 (d, *J* = 6.5 Hz, 3H, H^{11}); **^{13}C NMR** (126 MHz, CDCl$_3$) Data for the major diastereomer *a* only: δ 216.4 (C^3), 147.9 (C^6), 129.3 (C^8), 118.1 (C^9), 114.1 (C^7), 59.3 (C^4), 43.4 (C^2), 41.0 (C^{10}), 30.7 (C^{12}), 26.9 (C^1), 21.7 (C^{13}), 19.5 (C$^{13'}$), 10.2 (C^{11}); **HRMS** (ESl): calculated for $C_{17}H_{28}NO$ $[M+H]^+$ requires m/z 262.2165, found m/z 262.2181; **Chiral SFC:** DAICEL CHIRALCEL IE column (25 cm), CO$_2$:*i*-PrOH 97:3, 1.0 mL/min, 140 bar, 40 °C. Retention times: 9.5 mins (major), 10.3 mins (minor), e.r. = 71:29. To facilitate analysis by SFC, the major diastereoisomer was separated from the minor diastereoisomer by FCC.

SFC analysis of the major diastereomer of racemate, prepared using rac-BINAP:

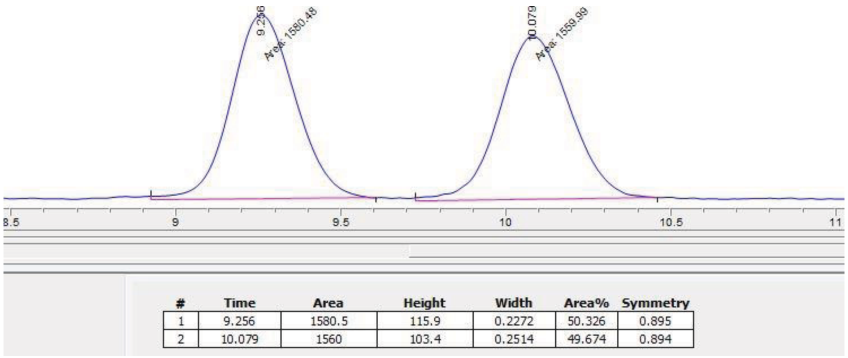

#	Time	Area	Height	Width	Area%	Symmetry
1	9.256	1580.5	115.9	0.2272	50.326	0.895
2	10.079	1560	103.4	0.2514	49.674	0.894

SFC analysis of the major diastereomer of enantioenriched material, prepared using (R)-DM-SEGPHOS:

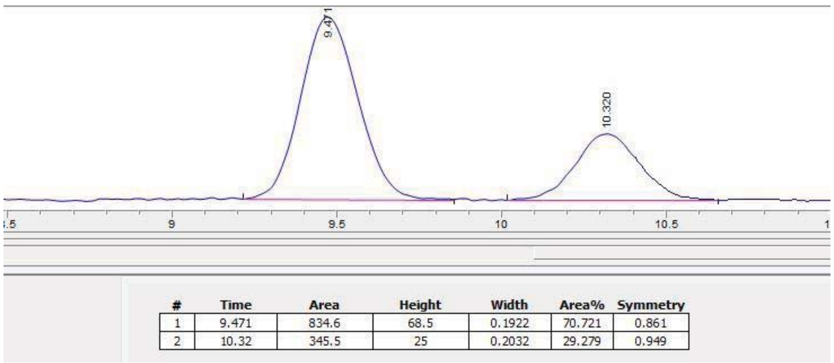

#	Time	Area	Height	Width	Area%	Symmetry
1	9.471	834.6	68.5	0.1922	70.721	0.861
2	10.32	345.5	25	0.2032	29.279	0.949

(*4S,5S*)-9-((*Tert*-butyldimethylsilyl)oxy)-2,2,5-trimethyl-4-(phenylamino)nonan-3-one (3os):

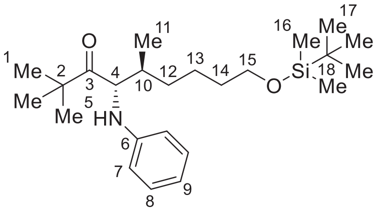

General procedure C: The reaction was carried out with substrate **1o** (19.1 mg, 0.10 mmol, 100 mol%), tert-butyl(hex-5-en-1-yloxy)dimethylsilane **2s** (42.8 mg, 1.00 mmol, 1000 mol%), Ir(cod)$_2$BARF (12.7 mg, 0.01 mmol, 10 mol%) and (*R*)-DM-SEGPHOS (7.23 mg, 0.01 mmol, 10 mol%) and was run in mesitylene (0.2 mL) at 110 °C. Purification of the residue by FCC (hexane/EtOAc 97:3) afforded the title compound (16.2 mg, 40%, >30:1 B:L, d.r. = 5:1 *a:b*, e.r. = 96:4) as a pale-yellow oil. ^{1}H NMR analysis of the crude material gave >30:1 B:L, d.r. = 5:1 and 37% substrate **1o**. $[\alpha]_D^{25}$ = +19.7 (c = 1.0 CHCl$_3$); **IR (thin film)** ν_{max}/cm^{-1}: 3381 (br),

2930 (s), 2857 (s), 1701 (s), 1601 (s), 1498 (s), 1251 (s), 1097 (s), 835 (s), 775 (s), 747 (s); **^{1}H NMR** (500 MHz, $CDCl_3$) Data for diastereomers *a* and *b*: δ 7.15 – 7.12 (m, 2H, H^8), 6.71 – 6.67 (m, 1H, H^9), 6.64 – 6.61 (m, 2H, H^7), 4.60 – 4.12 (m, 2H, $H^4 + H^5$), 3.63 – 3.55 (m, 2H, H^{15}), 1.94 – 1.88 (m, 1H, H^{10}), 1.56 – 1.25 (m, 6H, $H^{12} + H^{13} + H^{14}$), 1.14 – 1.12 (m, 9H, H^1), 0.90 – 0.86 (m, 12H, $H^{11} + H^{17}$), 0.05 – 0.03 (m, 6H, H^{16}); **^{13}C NMR** (126 MHz, $CDCl_3$) Data for the major diastereomer *a* only: δ 216.1 (C^3), 148.0 (C^6), 129.3 (C^8), 118.1 (C^9), 114.2 (C^7), 63.1 (C^{15}), 60.9 (C^4), 43.4 (C^2), 35.5 (C^{10}), 34.5 (C^{14}), 32.9 (C^{12}), 26.8 (C^{17}), 26.0 (C^1), 23.9 (C^{13}), 18.4 (C^{18}), 13.9 (C^{11}), -5.3 (C^{16}); **HRMS** (ESI): calculated for $C_{24}H_{43}NO_2SiNa$ $[M+Na]^+$ requires m/z 428.2955, found m/z 428.2947; **Chiral SFC:** YMC Chiral ART Cellulose-SC column (25 cm), CO_2:*i*-PrOH 97:3, 0.5 mL/min, 140 bar, 40 °C. Retention times: 13.6 mins (major), 14.4 mins (minor), e.r. = 96:4. To facilitate analysis by SFC, the major diastereoisomer was separated from the minor diastereoisomer by FCC.

SFC analysis of the major diastereomer of racemate, prepared using rac-BINAP:

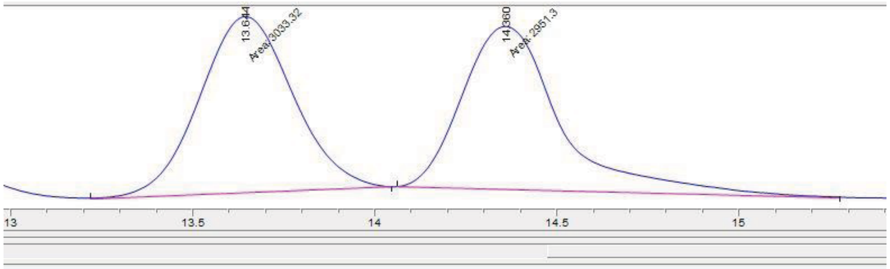

#	Time	Area	Height	Width	Area%	Symmetry
1	13.644	3033.3	169.4	0.2984	50.685	0.953
2	14.36	2951.3	156.3	0.3148	49.315	0.752

SFC analysis of the major diastereomer of enantioenriched material, prepared using (R)-DM-SEGPHOS:

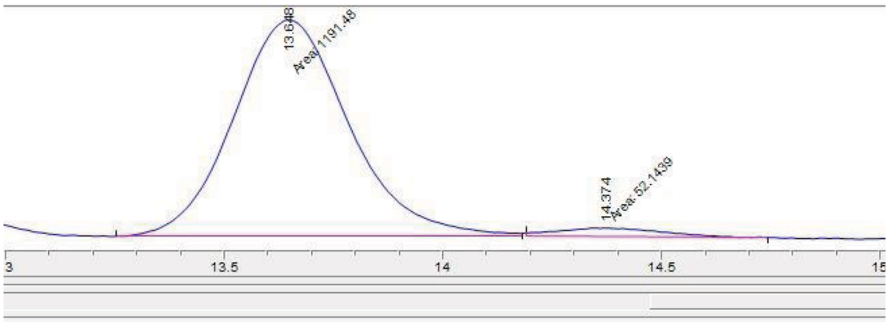

#	Time	Area	Height	Width	Area%	Symmetry
1	13.648	1191.5	64.4	0.3082	95.807	0.885
2	14.374	52.1	2.8	0.3065	4.193	0.512

4-((*2S,3S*)-4-(Dimethylamino)-4-oxo-3-(phenylamino)butan-2-yl)benzyl 2-(1-(4-chlorobenzoyl)-5-methoxy-2-methyl-1*H*-indol-3-yl)acetate (3ft):

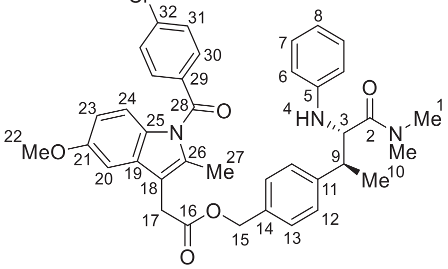

General procedure C: The reaction was carried out with substrate **1f** (17.8 mg, 0.10 mmol, 100 mol%) and 4-vinylbenzyl 2-(1-(4-chlorobenzoyl)-5-methoxy-2-methyl-1*H*-indol-3-yl)acetate **2t** (94.6 mg, 0.20 mmol, 200 mol%). Purification of the residue by FCC (hexane/EtOAc 60:40) afforded the title compound (41.0 mg, 63%, >30:1 B:L, d.r. = 20:1 *a:b*, e.r. = 98:2) as a pale-yellow oil. ^{1}H NMR analysis of the crude material gave >30:1 B:L and d.r. = 10:1. $[\alpha]_D^{25}$ = -4.0 (c = 1.0 $CHCl_3$); **IR (thin film)** ν_{max}/cm^{-1}: 3342 (br), 2931 (s), 1736 (s), 1681 (s), 1640 (s), 1602 (s), 1478 (s), 1316 (s), 1262 (s), 1160 (s), 754 (s), 735 (s); **^{1}H NMR** (500 MHz, $CDCl_3$) Data for the major diastereomer *a* only: δ 7.68 (d, *J* = 8.5 Hz, 2H, H^{30}), 7.49 (d, *J* = 8.5 Hz, 2H, H^{31}), 7.30 – 7.23 (m, 4H, ArH), 7.18 – 7.15 (m, 2H, H^7), 6.96 (d, *J* = 2.5 Hz, 1H, H^{20}), 6.90 (d, *J* = 9.0 Hz, 1H, ArH), 6.75 – 6.63 (m, 4H, ArH), 5.14 (s, 2H, H^{15}), 4.70 – 4.34 (m, 2H, H^3 + H^4), 3.80 (s, 3H, H^{22}), 3.73 (s, 2H, H^{17}), 3.36 – 3.30 (m, 1H, H^9), 2.91 (s, 3H, H^1), 2.73 (s, 3H, H$^{1'}$), 2.40 (s, 3H, H^{27}), 1.39 (d, *J* = 7.0 Hz, 3H, H^{10}); **^{13}C NMR** (126 MHz, $CDCl_3$) Data for the major diastereomer *a* only: δ 171.5 (C^2), 170.6 (C^{16}), 168.3 (C^{28}), 156.0 (C^{21}), 146.9 (C^5), 142.7 (C^{11}), 139.3 (ArC), 136.0 (ArC), 134.3 (ArC), 133.8 (ArC), 131.2 (C^{30}), 130.8 (C^{20}), 130.6 (ArC), 129.3 (C^7), 129.1 (C^{31}), 128.2 (C^{13}), 128.1 (C^{12}), 118.3 (C^8), 114.9 (ArC), 114.1 (C^6), 112.4 (ArC), 111.7 (ArC), 101.3 (C^{20}), 66.5 (C^{15}), 58.3 (C^3), 55.6 (C^{22}), 42.2 (C^9), 36.9 (C^1), 35.6 (C$^{1'}$), 30.4 (C^{17}), 16.3 (C^{10}), 13.4 (C^{27}); **HRMS** (ESI): calculated for $C_{38}H_{39}ClN_3O_5$ $[M+H]^+$ requires m/z 652.2573, found m/z 652.2592; **Chiral SFC:** DAICEL CHIRALCEL IE column (25 cm), CO_2:*i*-PrOH 70:30, 4.0 mL/min, 180 bar, 40 °C. Retention times: 21.5 mins (minor), 23.5 mins (major), e.r. = 98:2. To facilitate analysis by SFC, the major diastereoisomer was separated from the minor diastereoisomer by FCC.

SFC analysis of the major diastereomer of racemate, prepared using rac-BINAP:

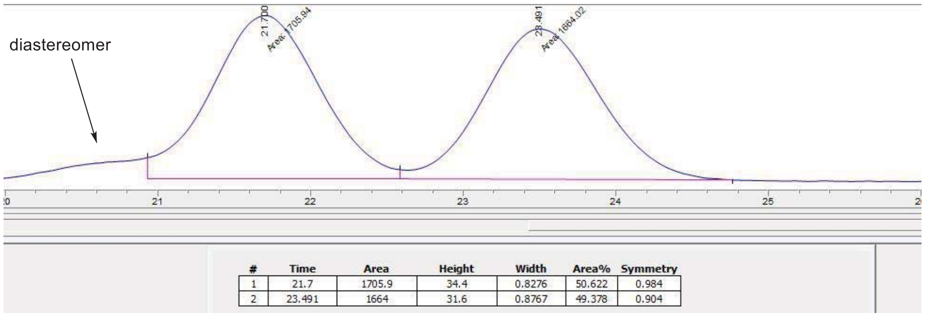

SFC analysis of the major diastereomer of enantioenriched material, prepared using (R)-SEGPHOS:

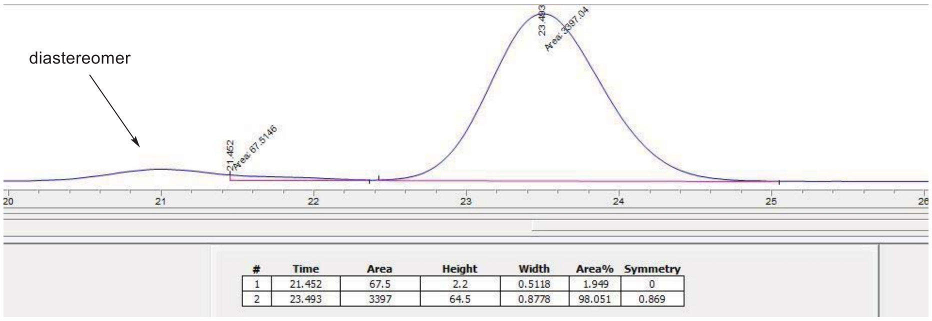

(*2R,3R*)-*N,N*-Dimethyl-3-phenyl-2-((phenylamino)methyl)butanamide (16):

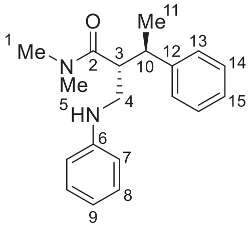

General procedure C: The reaction was carried out with substrate **15** (19.2 mg, 0.10 mmol, 100 mol%) and styrene (35.4 µL, 0.30 mmol, 300 mol%), $Ir(cod)_2BARF$ (12.7 mg, 0.01 mmol, 10 mol%) and (*R*)-BINAP (6.23 mg, 0.01 mmol, 10 mol%) and was run for 96 hours at 140 ^oC. Purification of the residue by FCC (hexane/EtOAc 50:50) afforded the title compound (16.3 mg, 55%, >30:1 B:L, d.r. >20:1 *a:b*, e.r. = 71:29) as a pale-yellow oil. ^1H NMR analysis of the crude material gave >30:1 B:L and d.r. = 2:1. $[\alpha]_D^{25}$ = -16.3 (c = 1.0 $CHCl_3$); **IR (thin film)** ν_{max}/cm^{-1}: 3348 (br), 2923 (s), 2854 (s), 1628 (s), 1602 (s), 1496 (s), 750 (s), 670 (s); **^{1}H NMR** (500 MHz, $CDCl_3$) Data for the major diastereomer *a* only: δ 7.37 – 7.33 (m, 2H, H^{14}), 7.27 – 7.25 (m, 3H, $H^{13} + H^{15}$), 7.10 – 7.06 (m, 2H, H^8), 6.64 – 6.61 (m, 1H H^9), 6.33 (d, *J* = 8.0 Hz, 2H, H^7), 3.97 (br s, 1H, H^5), 3.34 – 3.30 (m, 1H, H^3), 3.26 – 3.22 (m, 1H, H^4), 3.13 – 3.07 (m, 1H, H^{10}), 3.00 – 2.96 (m, 4H, $H^1 + H^{4'}$), 2.86 (s, 3H, $H^{1'}$), 1.22 (d, *J* = 7.0 Hz, 3H, H^{11}); **^{13}C**

NMR (126 MHz, $CDCl_3$) Data for the major diastereomer *a* only: δ 174.5 (C^2), 147.7 (C^6), 144.5 (C^{12}), 129.2 (C^8), 128.7 (C^{14}), 127.4 (C^{13}), 126.8 (C^{15}), 117.2 (C^9), 112.7 (C^7), 46.7 (C^3), 45.3 (C^4), 41.5 (C^{10}), 37.7 (C^1), 35.8 ($C^{1'}$), 20.2 (C^{11}); **HRMS** (ESI): calculated for $C_{19}H_{25}N_2O$ $[M+H]^+$ requires m/z 297.1961, found m/z 297.1968; **Chiral SFC:** DAICEL CHIRALCEL OD-H column (25 cm), CO_2:*i*-PrOH 80:20, 2.0 mL/min, 170 bar, 40 °C. Retention times: 3.6 mins (minor), 4.9 mins (major), e.r. = 71:29. To facilitate analysis by SFC, the major diastereoisomer was separated from the minor diastereoisomer by FCC.

SFC analysis of the major diastereomer of racemate, prepared using rac-BINAP:

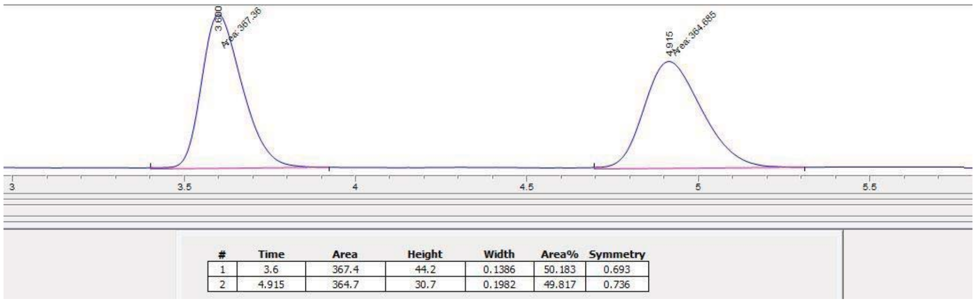

#	Time	Area	Height	Width	Area%	Symmetry
1	3.6	367.4	44.2	0.1386	50.183	0.693
2	4.915	364.7	30.7	0.1982	49.817	0.736

SFC analysis of the major diastereomer of enantioenriched material, prepared using (R)-DM-SEGPHOS:

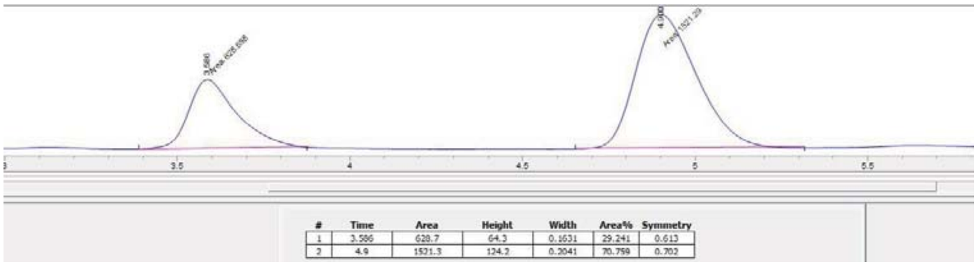

#	Time	Area	Height	Width	Area%	Symmetry
1	3.586	628.7	64.3	0.1631	29.241	0.613
2	4.9	1521.3	124.2	0.2041	70.759	0.702

Supplementary Figure 1

Supplementary Figure 1a: Other styrenes, including 1-(*tert*-butyl)-4-vinylbenzene, 1-fluoro-4-vinylbenzene, 1-chloro-3-vinylbenzene and 1-fluoro-2-vinylbenzene, have been evaluated and led to the formation of the corresponding products **3fu-3fx** in 42-77 yields, >30:1 branched:linear selectivity, 6:1-9:1 d.r. and 97:3- 98.5:1.5 e.r. Supplementary Figure 1b: When primary amide **1x** (R^1 = NH_2) was subjected to this reaction, the corresponding product **3xa** was isolated in 29% yield, 97:3 e.r., >30:1 branched to linear selectivity and 3:1 d.r.

Supplementary Figure 1. Further scope of the process.

a.

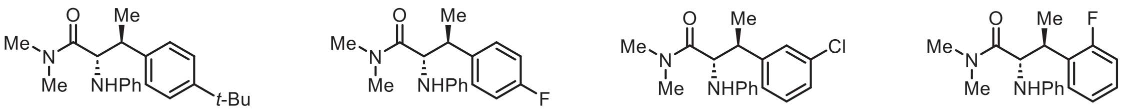

3fu, 58% Yield, 97:3 e.r. >30:1 B:L, 8:1 d.r.

3fv, 62% Yield, 98.5:1.5 e.r. >30:1 B:L, 8:1 d.r.

3fw, 42% Yield, 97:3 e.r. >30:1 B:L, 9:1 d.r.

3fx, 77% Yield, 98:2 e.r. >30:1 B:L, 6:1 d.r.

b.

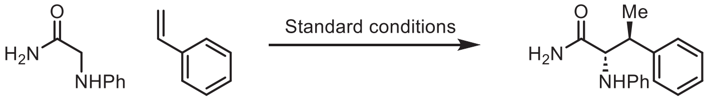

1x

3xa, 29% Yield, 97:3 e.r. >30:1 B:L, 3:1 d.r.

(*2S,3S*)-3-Phenyl-2-(phenylamino)butanamide (3xa):

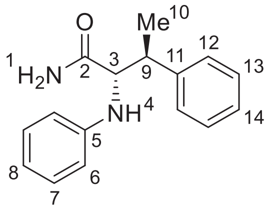

General procedure C: The reaction was carried out with substrate **1x** (15.0 mg, 0.10 mmol, 100 mol%) and styrene (23.0 μL, 0.20 mmol, 200 mol%) at 140 °C. Purification of the residue by FCC (hexane/EtOAc 40:60) afforded the title compound (7.3 mg, 29%, >30:1 B:L, d.r. = 3:1 *a*:*b*, e.r. = 97:3) as a pale-yellow oil. ^{1}H NMR analysis of the crude material gave >30:1 B:L, d.r. = 3:1 and 50% substrate **1x**. $[\alpha]_D^{25}$ = +60.0 (c = 0.5, $CHCl_3$); **IR (thin film)** ν_{max}/cm^{-1}: 3304 (br), 2930 (s), 1664 (s), 1601 (s), 1500 (s), 1312 (s), 736 (s); **^{1}H NMR** (500 MHz, $CDCl_3$) Data for diastereomers *a* and *b*: δ 7.38 – 7.22 (m, 5H, H^{12} + H^{13} + H^{14}), 7.18 – 7.12 (m, 2H, H^7), 6.80 – 6.75 (m, 1H, H^8), 6.58 – 6.53 (m, 2H, H^6), 6.50 (br s, 0.21H, H^{1b}), 6.42 (br s, 0.77H, H^{1a}), 5.62 (br s, 0.22H, H^{1b}), 5.50 (br s, 0.74H, H^{1a}), 4.10 – 3.70 (m, 2H, H^3 + H^4), 3.62 – 3.57 (m, 0.25H, H^{9b}), 3.46 – 3.41 (m, 0.78H, H^{9a}), 1.47 – 1.44 (m, 3H, H^{10}); **^{13}C NMR** (126 MHz, $CDCl_3$) Data for diastereomers *a* and *b*: δ 175.7 (C^{2a}), 175.0 (C^{2b}), 146.9 (C^{5b}), 146.7 (C^{5a}), 141.9 (C^{11b}), 141.3 (C^{11a}), 129.4 (C^{7a}), 129.3 (C^{7b}), 129.0 (C^{13a}), 128.8 (C^{13b}), 127.7 (C^{12a}), 127.44 (C^{14a}), 127.37 (C^{12b}), 127.1 (C^{14b}), 119.23 (C^{8b}), 119.17 (C^{8a}), 113.9 (C^{6b}), 113.8 (C^{6a}), 64.5 (C^{3a}), 64.4 (C^{3b}), 42.2 (C^{9a}), 41.2 (C^{9b}), 18.6 (C^{10a}), 14.7 (C^{10b}); **HRMS** (ESI): calculated for $C_{16}H_{18}N_2ONa$ $[M+Na]^+$ requires *m/z* 277.1311, found *m/z* 277.1303; YMC Chiral ART Cellulose-SC column (25 cm), CO_2:*i*-PrOH 90:10, 2.0 mL/min, 165 bar, 40 °C. Retention

times: 17.4 mins (major), 17.8 mins (minor), e.r. = 97:3. To facilitate analysis by SFC, the major diastereoisomer was separated from the minor diastereoisomer by FCC.

SFC analysis of the major diastereomer of the racemate, prepared using rac-BINAP:

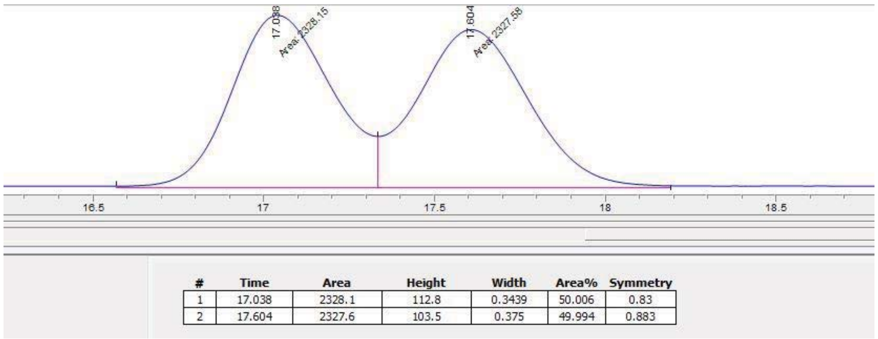

#	Time	Area	Height	Width	Area%	Symmetry
1	17.038	2328.1	112.8	0.3439	50.006	0.83
2	17.604	2327.6	103.5	0.375	49.994	0.883

SFC analysis of the major diastereomer of enantioenriched material, prepared using (R)-SEGPHOS:

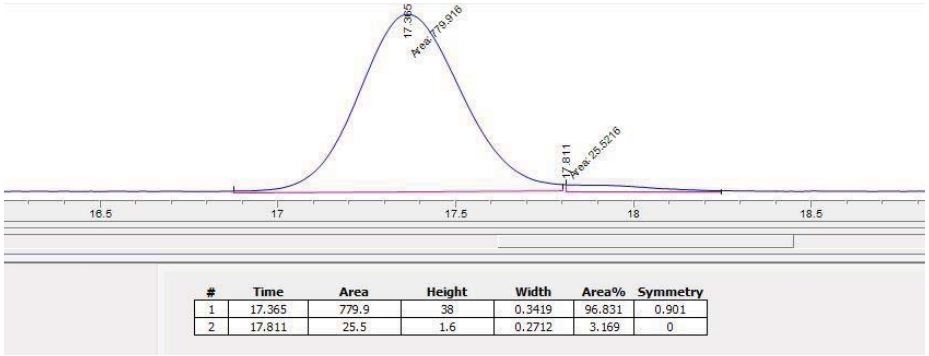

#	Time	Area	Height	Width	Area%	Symmetry
1	17.365	779.9	38	0.3419	96.831	0.901
2	17.811	25.5	1.6	0.2712	3.169	0

(*2S,3S*)-3-(4-(*Tert*-butyl)phenyl)-*N,N*-dimethyl-2-(phenylamino)butanamide (3fu):

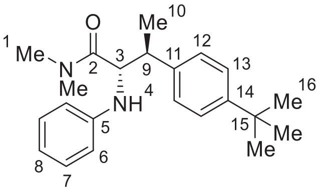

General procedure C: The reaction was carried out with substrate **1f** (17.8 mg, 0.10 mmol, 100 mol%) and 1-(*tert*-butyl)-4-vinylbenzene **2u** (37.0 µL, 0.20 mmol, 200 mol%). Purification of the residue by FCC (hexane/EtOAc 70:30) afforded the title compound (19.6 mg, 58%, >30:1 B:L, d.r. = 8:1 *a:b*, e.r. = 97:3) as a pale-yellow solid. ^{1}H NMR analysis of the crude material gave >30:1 B:L and d.r. = 8:1. **m.p.** = 121 – 123 (hexane/EtOAc); $[\alpha]_D^{23}$ = -2.5 (c = 0.16, $CHCl_3$); **IR (thin film)** ν_{max}/cm^{-1}: 3681 (s), 3350 (br), 2960 (s), 2925 (s), 2868 (s), 1638 (s), 1632 (s), 1502 (s); **^{1}H NMR** (500 MHz, $CDCl_3$) Data for the major diastereomer *a*: δ 7.33

– 7.31 (m, 2H, H^{13}), 7.18 – 7.13 (m, 4H, H^{7} + H^{12}), 6.71 – 6.68 (m, 1H, H^{8}), 6.62 (d, *J* = 7.5 Hz, 2H, H^{6}), 4.80 – 4.29 (m, 2H, H^{3} + H^{4}), 3.34 – 3.29 (m, 1H, H^{9}), 2.89 (s, 3H, H^{1}), 2.61 – 2.60 (s, 3H, $H^{1'}$), 1.35 (d, *J* = 7.0 Hz, 3H, H^{10}), 1.30 (s, 9H, H^{16}). Characteristic signals for the minor diastereomer *b*: 6.49 (d, *J* = 7.5 Hz, 2H, H^{6}), 2.74 (s, 3H, H^{1}), 1.45 (d, *J* = 7.0 Hz, 3H, H^{10}); **^{13}C NMR** (126 MHz, $CDCl_3$) Data for the major diastereomer *a* only: δ 171.7 (C^{2}), 150.0 (C^{14}), 147.0 (C^{5}), 139.5 (C^{11}), 129.4 (C^{7}), 127.7 (C^{13}), 125.4 (C^{12}), 118.1 (C^{8}), 114.1 (C^{6}), 58.5 (C^{3}), 41.9 (C^{9}), 36.9 (C^{1}), 35.8 ($C^{1'}$), 34.6 (C^{15}), 31.5 (C^{16}), 15.9 (C^{10}); **HRMS** (ESl): calculated for $C_{22}H_{31}N_2O$ $[M+H]^+$ requires *m/z* 339.2431, found *m/z* 339.2434; **Chiral SFC:** DAICEL CHIRALCEL OD-H column (25 cm), CO_2:*i*-PrOH 95.5:4.5, 2.5 mL/min, 140 bar, 40 °C. Retention times: 11.5 mins (major), 14.1 mins (minor), e.r. = 97:3. To facilitate analysis by SFC, the major diastereoisomer was separated from the minor diastereoisomer by FCC.

SFC analysis of the major diastereomer of racemate, prepared using rac-BINAP:

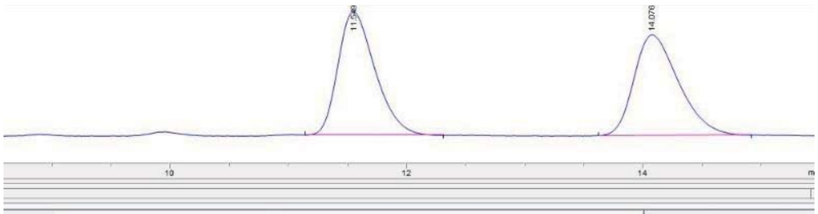

#	Time	Area	Height	Width	Area%	Symmetry
1	11.549	891.3	40.7	0.3354	50.629	0.68
2	14.076	869.1	33.2	0.3837	49.371	0.656

SFC analysis of the major diastereomer of enantioenriched material, prepared using (R)-SEGPHOS:

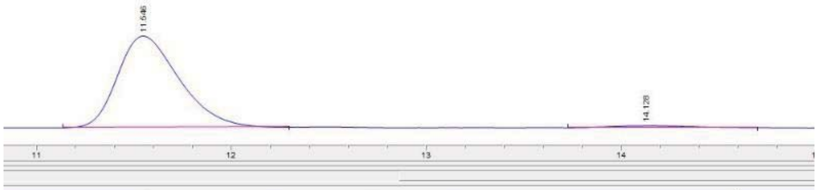

#	Time	Area	Height	Width	Area%	Symmetry
1	11.546	2131	96.9	0.3345	97.306	0.675
2	14.128	59	2.1	0.3458	2.694	0.783

(*2S,3S*)-3-(4-Fluorophenyl)-*N,N*-dimethyl-2-(phenylamino)butanamide (3fv):

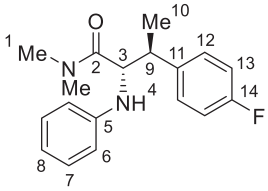

General procedure C: The reaction was carried out with substrate **1f** (17.8 mg, 0.10 mmol, 100 mol%) and 1-fluoro-4-vinylbenzene **2v** (24.0 μL, 0.20 mmol, 200 mol%). Purification of the residue by FCC (hexane/EtOAc 60:40) afforded the title compound (18.6 mg, 62%, >30:1 B:L, d.r. = 8:1 *a:b*, e.r. = 98.5:1.5) as a pale-yellow solid. ^{1}H NMR analysis of the crude material gave >30:1 B:L and d.r. = 8:1. **m.p.** = 117 – 120 °C (hexane/EtOAc); $[\boldsymbol{\alpha}]_{\boldsymbol{D}}^{\boldsymbol{24}}$ = **-**5.1 (c = 0.19, $CHCl_3$); **IR (thin film)** ν_{max}/cm^{-1}: 3709 (s), 3681 (s), 3337 (br), 2968 (s), 2937 (s), 2873 (s), 2845 (s), 1637 (s), 1602 (s), 1508 (s); **^{1}H NMR** (500 MHz, $CDCl_3$) Data for the major diastereomer *a*: δ 7.22 – 7.13 (m, 4H, H^{12} + H^{13}), 7.01 – 6.97 (m, 2H, H^7), 6.72 – 6.69 (m, 1H, H^8), 6.61 (d, *J* = 8.0 Hz, 2H, H^6), 4.73 – 4.29 (m, 2H, H^3 + H^4), 3.33 – 3.27 (m, 1H, H^9), 2.90 (s, 3H, H^1), 2.76 – 2.75 (m, 3H, $H^{1'}$), 1.36 (d, *J* = 7.0 Hz, 3H, H^{10}). Characteristic signals for the minor diastereomer *b*: 6.52 (d, *J* = 8.0 Hz, 2H, H^6), 2.68 (s, 3H, H^1), 1.43 (d, *J* = 7.0 Hz, 3H, H^{10}); **^{13}C NMR** (126 MHz, $CDCl_3$) Data for the major diastereomer *a* only: δ 171.7 (C^2), 161.9 (d, *J* = 245.0 Hz, C^{14}), 147.1 (C^5), 138.2 (d, *J* = 3.0 Hz, C^{11}), 129.5 – 129.4 (C^7 + C^{12}), 118.4 (C^8), 115.3 (d, *J* = 21.0 Hz, H^{13}), 114.2 (C^6), 58.5 (C^3), 41.9 (C^9), 37.1 (C^1), 35.8 ($C^{1'}$), 16.5 (C^{10}); ^{19}F NMR (471 MHz, $CDCl_3$) δ -115.90; **HRMS** (ESI): calculated for $C_{18}H_{22}FN_2O$ $[M+H]^+$ requires *m/z* 301.1711, found *m/z* 301.1712; **Chiral SFC:** DAICEL CHIRALCEL OD-H column (25 cm), CO_2:*i*-PrOH 97:3, 2.5 mL/min, 140 bar, 40 °C. Retention times: 15.1 mins (minor), 16.2 mins (major), e.r. = 98.5:1.5. To facilitate analysis by SFC, the major diastereoisomer was separated from the minor diastereoisomer by FCC.

SFC analysis of the racemates, prepared using rac-BINAP:

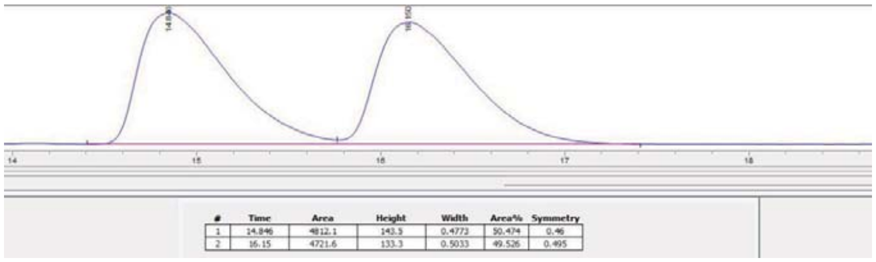

#	Time	Area	Height	Width	Area%	Symmetry
1	14.846	4812.1	143.5	0.4773	50.474	0.46
2	16.15	4721.6	133.3	0.5033	49.526	0.495

SFC analysis of the major diastereomer of enantioenriched material, prepared using (R)-SEGPHOS:

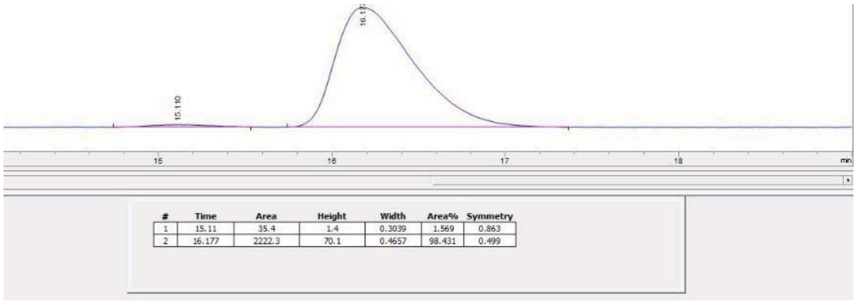

(*2S,3S*)-3-(3-Chlorophenyl)-*N,N*-dimethyl-2-(phenylamino)butanamide (3fw):

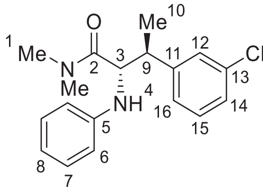

General procedure C: The reaction was carried out with substrate **1f** (17.8 mg, 0.10 mmol, 100 mol%) and 1-chloro-3-vinylbenzene **2w** (25.0 µL, 0.20 mmol, 200 mol%). Purification of the residue by FCC (hexane/EtOAc 70:30) afforded the title compound (13.3 mg, 42%, >30:1 B:L, d.r. = 9:1 *a:b*, e.r. = 97:3) as a pale-yellow oil. ^{1}H NMR analysis of the crude material gave >30:1 B:L and d.r. = 9:1. $[\alpha]_D^{25}$ = -8.8 (c = 0.29, $CHCl_3$); **IR (thin film)** ν_{max}/cm^{-1}: 3668 (s), 3329 (br), 3055 (s), 3025 (s), 2968 (s), 2936 (s), 2877 (s), 1636 (s), 1601 (s), 1572 (s); **^{1}H NMR** (500 MHz, $CDCl_3$) Data for the major diastereomer *a*: δ 7.24 – 7.10 (m, 6H, $H^7 + H^{12} + H^{14} + H^{15} + H^{16}$), 6.73 – 6.70 (m, 1H, H^8), 6.62 (d, *J* = 8.0 Hz, 2H, H^6), 4.68 – 4.32 (m, 2H, $H^3 + H^4$), 3.32 – 3.26 (m, 1H, H^9), 2.91 (s, 3H, H^1), 2.74 (s, 3H, $H^{1'}$), 1.35 (d, *J* = 7.0 Hz, 3H, H^{10}). Characteristic signals for the minor diastereomer *b*: 6.54 (d, *J* = 8.0 Hz, 2H, H^6), 2.76 (s, 3H, H^1), 2.68 (s, 3H, $H^{1'}$), 1.44 (d, *J* = 7.0 Hz, 3H, H^{10}); **^{13}C NMR** (126 MHz, $CDCl_3$) Data for the major diastereomer *a* only: δ 171.5 (C^2), 146.9 (C^5), 144.8 (C^{11}), 134.4 (C^{13}), 129.8 (ArC), 129.5 (C^7), 128.1 (ArC), 127.2 (ArC), 126.4 (ArC), 118.5 (C^8), 114.3 (C^6), 58.2 (C^3), 42.4 (C^9), 37.2 (C^1), 35.8 ($C^{1'}$), 16.2 (C^{10}); **HRMS** (ESI): calculated for $C_{18}H_{22}ClN_2O$ $[M+H]^+$ requires *m/z* 317.1415, found *m/z* 317.1416; **Chiral SFC:** YMC Chiral ART Cellulose-SC column (25 cm), CO_2:*i*-PrOH 90:10, 2.0 mL/min, 140 bar, 40 °C. Retention times: 9.4 mins (minor), 11.8 mins (major), e.r. = 97:3. To facilitate analysis by SFC, the major diastereoisomer was separated from the minor diastereoisomer by FCC.

SFC analysis of the racemates, prepared using rac-BINAP:

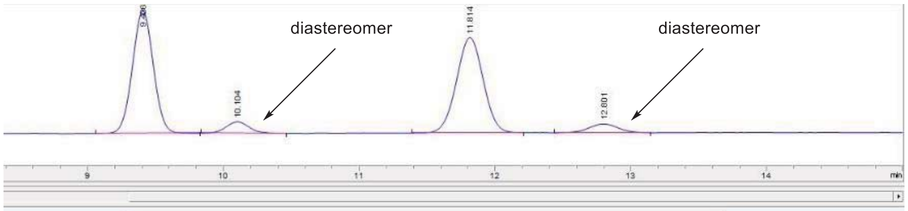

#	Time	Area	Height	Width	Area%	Symmetry
1	9.406	666.6	62.3	0.1669	45.448	0.967
2	10.104	69	5.8	0.183	4.706	0.963
3	11.814	663	48.4	0.2119	45.201	0.99
4	12.801	68.1	4.4	0.2248	4.645	1.008

SFC analysis of the major diastereomer of enantioenriched material, prepared using (R)-SEGPHOS:

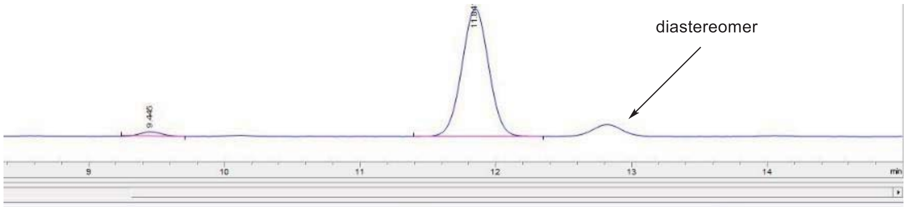

#	Time	Area	Height	Width	Area%	Symmetry
1	9.445	24.1	2.1	0.1652	2.761	0.822
2	11.841	849.9	61.9	0.2142	97.239	0.972

(*2S,3S*)-3-(2-Fluorophenyl)-*N,N*-dimethyl-2-(phenylamino)butanamide (3fx):

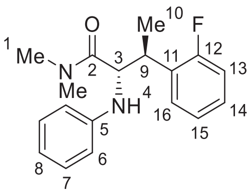

General procedure C: The reaction was carried out with substrate **1f** (17.8 mg, 0.10 mmol, 100 mol%) and 2-fluorostyrene **2x** (24.0 µL, 0.20 mmol, 200 mol%). Purification of the residue by FCC (hexane/EtOAc 70:30) afforded the title compound (23.1 mg, 77%, >30:1 B:L, d.r. = 6:1 *a:b*, e.r. = 98:2) as a pale-yellow solid. ^{1}H NMR analysis of the crude material gave >30:1 B:L and d.r. = 6:1. **m.p.** = 78 – 81 °C (hexane/EtOAc); $[\boldsymbol{\alpha}]_{\boldsymbol{D}}^{\mathbf{24}}$ = **-**2.2 (c = 0.34, $CHCl_3$); **IR (thin film)** ν_{max}/cm^{-1}: 3681 (s), 3333 (br), 2973 (s), 2938 (s), 2874 (s), 2845 (s), 1638 (s), 1602 (s), 1584 (s); **^{1}H NMR** (500 MHz, $CDCl_3$) Data for the major diastereomer *a*: δ 7.27 – 7.00 (m, 6H, H^{7} + H^{13} + H^{14} + H^{15} + H^{16}), 6.71 – 6.62 (m, 3H, H^{6} + H^{8}), 4.87 – 4.38 (m, 2H, H^{3} + H^{4}),

3.70 – 3.64 (m, 1H, H[9]), 2.88 (s, 3H, H[1]), 2.76 (s, 3H, H[1']), 1.37 (d, J = 7.0 Hz, 3H, H[10]). Characteristic signals for the minor diastereomer *b*: 6.37 (d, J = 7.0 Hz, 3H, H[6]), 3.01 (s, 4H, H[1]), 2.86 (s, 4H, H[1']); **^{13}C NMR** (126 MHz, $CDCl_3$) Data for the major diastereomer *a* only: δ 171.7 (C[2]), 161.3 (d, J = 244.0 Hz, C[12]), 147.1 (C[5]), 129.5 – 129.2 (m, ArC), 128.5 (d, J = 8.5 Hz, ArC), 124.3 (d, J = 3.5 Hz, C[15]), 118.2 (C[8]), 115.3 (d, J = 22.7 Hz, C[13]), 114.1 (C[6]), 56.6 (C[3]), 37.0 (C[1]), 35.7 (C[1']), 35.6 (C[9]), 15.2 (C[10]); **^{19}F NMR** (471 MHz, $CDCl_3$) δ -117.92 (dt, J = 12.0, 6.5 Hz), -118.48 (dt, J = 12.0, 6.5 Hz); **HRMS** (ESl): calculated for $C_{18}H_{22}FN_2O$ $[M+H]^+$ requires *m/z* 301.1711, found *m/z* 301.1713; **Chiral SFC:** YMC Chiral ART Cellulose-SC column (25 cm), CO_2:*i*-PrOH 90:10, 2.0 mL/min, 140 bar, 40 °C. Retention times: 7.7 mins (minor), 9.6 mins (major), e.r. = 98:2. To facilitate analysis by SFC, the major diastereoisomer was separated from the minor diastereoisomer by FCC.

SFC analysis of the racemates, prepared using rac-BINAP:

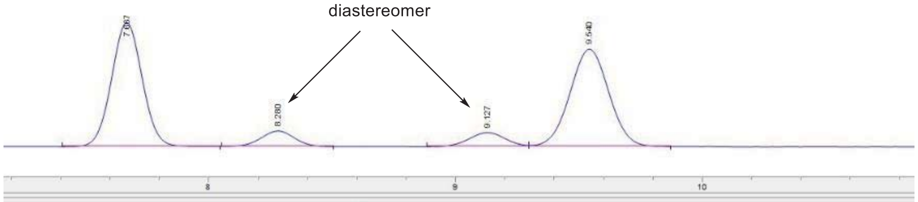

#	Time	Area	Height	Width	Area%	Symmetry
1	7.667	537.5	62.5	0.1343	44.009	0.952
2	8.28	73	7.8	0.1472	5.977	1.023
3	9.127	72.5	7.1	0.1578	5.940	1.005
4	9.54	538.3	49	0.17	44.074	0.975

SFC analysis of the major diastereomer of enantioenriched material, prepared using (R)-SEGPHOS:

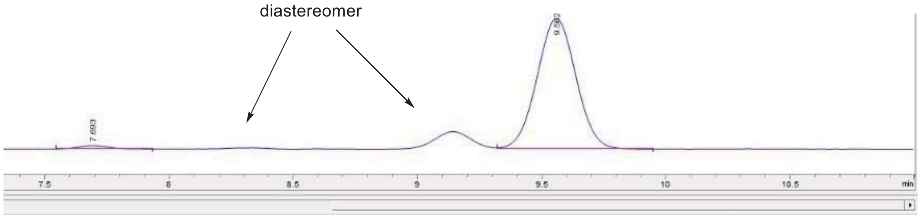

#	Time	Area	Height	Width	Area%	Symmetry
1	7.693	15.6	1.8	0.1285	1.937	0.835
2	9.562	789.2	72.1	0.1698	98.063	0.969

Supplementary Figure 2

Supplementary Figure 2a: Other alkenes, including 4-vinylpyridine, 6-vinylquinoline, 4-vinylbenzonitrile, 4,4,5,5-tetramethyl-2-vinyl-1,3,2-dioxaborolane, hex-1-ene, allylbenzene, but-3-en-1-yn-1-ylbenzene, triethyl(vinyl)silane, ethene-1,1-diyldibenzene, (*E*)-prop-1-en-1-ylbenzene, cyclohexene and ethene, have been evaluated and only led to the formation of the corresponding products in very low yields as yet. Supplementary Figure 2b: Use of substrate **1y** with an *ortho*-substituted N-aryl unit led to the formation of the corresponding product in only 15% NMR yield and 9:1 d.r..

Supplementary Figure 2. Limitations of the process.

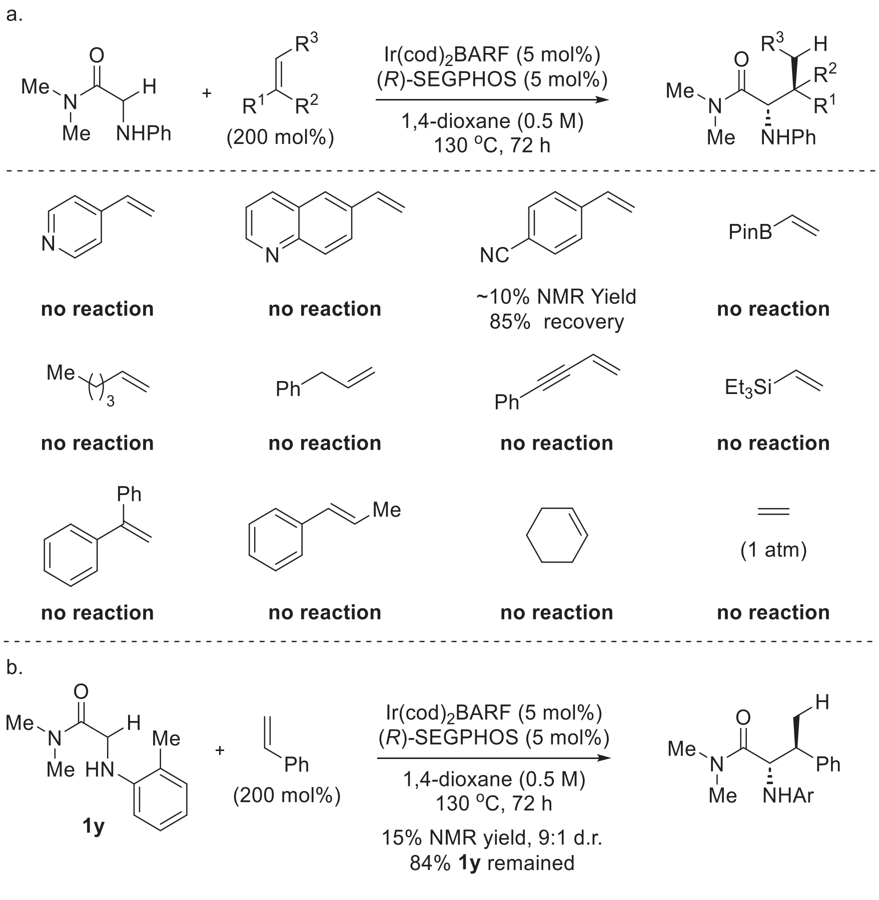

Applications and derivatizations

Synthesis of **3fa** on 1.00 mmol scale:

A Schlenk tube was charged with amide **1f** (178 mg, 1.00 mmol, 100 mol%), [Ir(cod)$_2$]BARF (63.6 mg, 0.05 mmol, 5 mol%) and (*R*)-SEGPHOS (30.5 mg, 0.05 mmol, 5 mol%). The

Schlenk tube was evacuated and refilled with N_2 (three cycles), then styrene (229 μL, 2.00 mmol, 200 mol%) was added followed by 1,4-dioxane (2.0 mL). The tube was sealed and heated at 130 °C for 72 hours. After cooling to r.t., the solvent was removed under reduced pressure and the crude reaction mixture was purified by FCC (hexane/EtOAc 65:35) to afford the desired product **3fa** (265 mg, 94%, >30:1 B:L, d.r. = 10:1, e.r. = 98:2) as a pale-yellow solid. ^{1}H NMR analysis of the crude material gave >30:1 B:L and d.r. = 10:1.

(*2S,3S*)-3-Phenyl-2-(phenylamino)butan-1-ol (4):

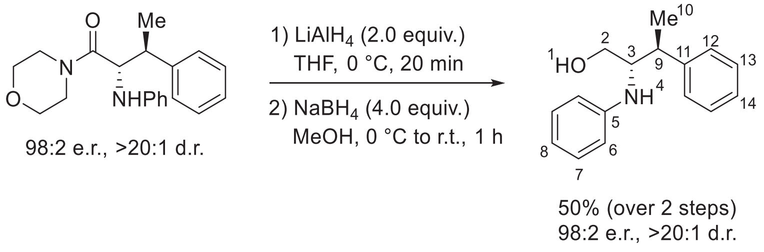

$LiAlH_4$ (7.60 mg, 0.20 mmol, 200 mol%) was slowly added, portion-wise, to a solution of **3ka** (32.4 mg, 0.10 mmol, 100 mol%) in anhydrous THF (1.0 mL) at 0 °C in a sealed tube. After the addition was complete, the reaction mixture was stirred for 20 minutes at 0 °C and the progress of the reaction was monitored by TLC. Upon completion, water (1 drop) was added to the reaction mixture, followed by the addition of aq. 4M NaOH solution (1 drop) and water (2 drops). The resulting suspension was then warmed to r.t. and stirred for 15 minutes before anhydrous Na_2SO_4 was added. The mixture was stirred for an additional 15 minutes before filtration. The filtrate was collected, and the solvent was removed *in vacuo* to provide the amino aldehyde intermediate which could be used in the next step without purification.

A solution of the above synthesized amino aldehyde intermediate in MeOH (1.0 mL) was stirred at 0 °C in a sealed tube. $NaBH_4$ (15.1 mg, 0.40 mmol, 400 mol%) was then added to the stirring solution. Then the solution was stirred at ambient temperature for 1 hour. Upon completion, the reaction mixture was transferred to a separatory funnel and water (approx. 5.0 mL) was added. The aqueous phase was extracted with EtOAc (approx. 3 × 10.0 mL). The combined organic phases were dried over anhydrous $MgSO_4$. The concentration of the filtrate *in vacuo* was followed by FCC (hexane/EtOAc 25:75) to afford the desired product **4** (12.1 mg, 50%, over 2 steps, d.r. >20:1, e.r. = 99:1) as a colorless oil. $[\boldsymbol{\alpha}]_{\boldsymbol{D}}^{\mathbf{25}}$ = +26.4 (c = 1.0 $CHCl_3$); **IR (thin film)** ν_{max}/cm^{-1}: 3399 (br), 2964 (s), 2928 (s), 1600 (s), 1495 (s), 1317 (s), 1261 (s), 747 (s), 691 (s); **^{1}H NMR** (500 MHz, $CDCl_3$) δ 7.33 – 7.21 (m, 5H, $H^{12} + H^{13} + H^{14}$), 7.16 – 7.13 (m, 2H, H^7), 6.73 – 6.70 (m, 1H, H^8), 6.62 (d, *J* = 7.5 Hz, 2H, H^6), 3.76 – 3.68 (m, 2H,

H^{2}), 3.61 – 3.26 (m, 2H, H^{3} + H^{4}), 3.19 – 3.14 (m, 1H, H^{9}), 1.91 (br s, 1H, H^{1}), 1.35 (d, J = 7.0 Hz, 3H, H^{10}); **^{13}C NMR** (126 MHz, $CDCl_3$) δ 147.9 (C^{5}), 142.7 (C^{11}), 129.3 (C^{7}), 128.6 (C^{13}), 127.9 (C^{12}), 126.8 (C^{14}), 118.0 (C^{8}), 113.9 (C^{6}), 62.5 (C^{2}), 60.2 (C^{3}), 41.0 (C^{9}), 18.1 (C^{10}); **HRMS** (ESI): calculated for $C_{16}H_{20}NO$ $[M+H]^+$ requires m/z 242.1539, found m/z 242.1540; **Chiral SFC:** YMC Chiral ART Cellulose-SC column (25 cm), CO_2:*i*-PrOH 92:8, 2.0 mL/min, 140 bar, 40 °C. Retention times: 6.4 mins (minor), 7.1 mins (major), e.r. = 99:1.

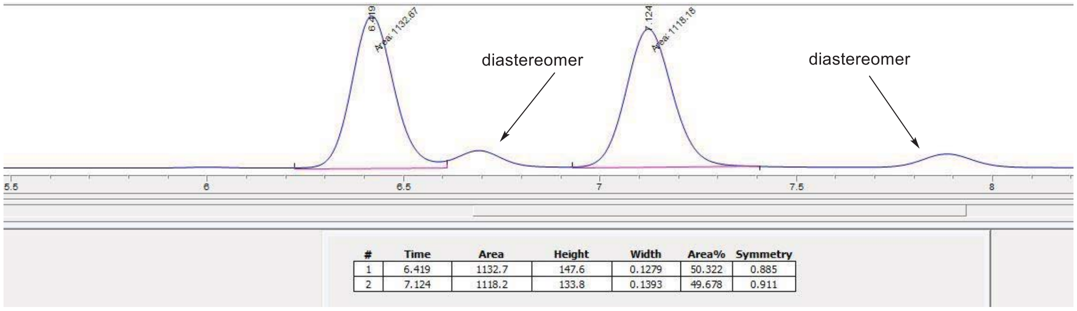

#	Time	Area	Height	Width	Area%	Symmetry
1	6.419	1132.7	147.6	0.1279	50.322	0.885
2	7.124	1118.2	133.8	0.1393	49.678	0.911

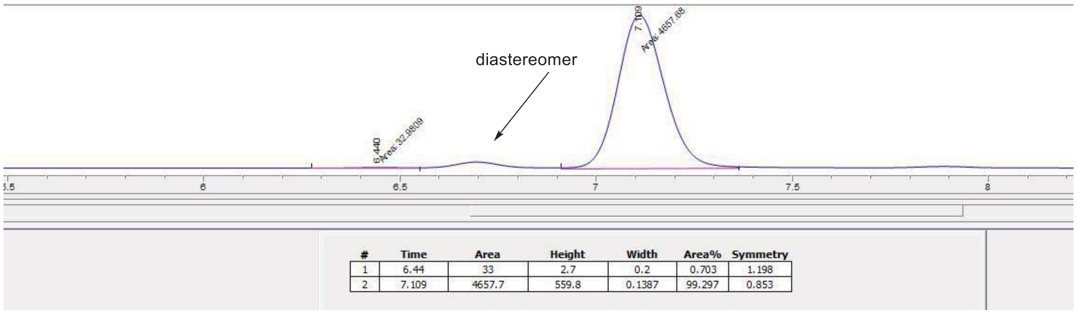

#	Time	Area	Height	Width	Area%	Symmetry
1	6.44	33	2.7	0.2	0.703	1.198
2	7.109	4657.7	559.8	0.1387	99.297	0.853

(*2S,3S*)-3-Phenyl-2-(phenylamino)butanal (5):

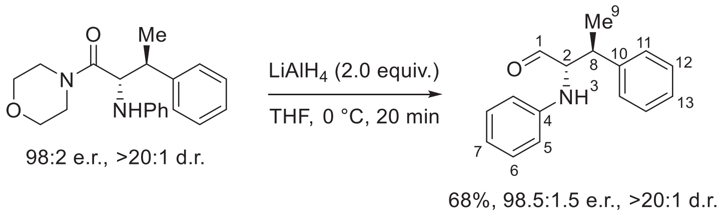

$LiAlH_4$ (7.60 mg, 0.20 mmol, 200 mol%) was slowly added, portion-wise, to a solution of **3ka** (32.4 mg, 0.10 mmol, 100 mol%) in anhydrous THF (1.0 mL) at 0 °C in a sealed tube. After the addition was complete, the reaction mixture was stirred for 20 minutes at 0 °C and the progress of the reaction was monitored by TLC. Upon completion, water (1 drop) was added to the reaction mixture, followed by the addition of aq. 4M NaOH solution (1 drop) and water (2 drops). The resulting suspension was then warmed to r.t. and stirred for 15 minutes before anhydrous Na_2SO_4 was added. The mixture was stirred for an additional 15 minutes before

filtration. The concentration of the filtrate *in vacuo* was followed by FCC (hexane/EtOAc 10:90) to afford the desired product **5** (16.3 mg, 68%, d.r. >20:1, e.r. = 98.5:1.5) as a colorless oil. $[\boldsymbol{\alpha}]_{\boldsymbol{D}}^{\mathbf{25}}$ = +17.7 (c = 1.0 $CHCl_3$); **IR (thin film)** ν_{max}/cm^{-1}: 3404 (br), 3027 (s), 2970 (s), 2931 (s), 1726 (s), 1599 (s), 1494 (s), 1310 (s), 1266 (s), 748 (s), 698 (s); **^{1}H NMR** (500 MHz, $CDCl_3$) δ 9.53 (d, *J* = 2.5 Hz, 1H, H^1), 7.39 – 7.28 (m, 5H, H^{11} + H^{12} + H^{13}), 7.19 – 7.15 (m, 2H, H^6), 6.76 – 6.73 (m, 1H, H^7), 6.60 (d, *J* = 7.5 Hz, 2H, H^5), 4.21 – 3.95 (m, 2H, H^2 + H^3), 3.43 – 3.38 (m, 1H, H^8), 1.38 (d, *J* = 7.0 Hz, 3H, H^9); **^{13}C NMR** (126 MHz, $CDCl_3$) δ 202.3 (C^1), 146.6 (C^4), 140.8 (C^{10}), 129.4 (C^6), 129.0 (C^{12}), 127.8 (C^{11}), 127.4 (C^{13}), 118.4 (C^7), 113.3 (C^5), 67.3 (C^2), 39.7 (C^8), 17.1 (C^9); **HRMS** (ESI): calculated for $C_{16}H_{18}NO$ $[M+H]^+$ requires m/z 240.1383, found m/z 240.1389; **Chiral SFC:** DAICEL CHIRALCEL OD-H column (25 cm), CO_2:*i*-PrOH 95:5, 1.0 mL/min, 140 bar, 40 °C. Retention times: 15.3 mins (major), 16.4 mins (minor), e.r. = 98.5:1.5.

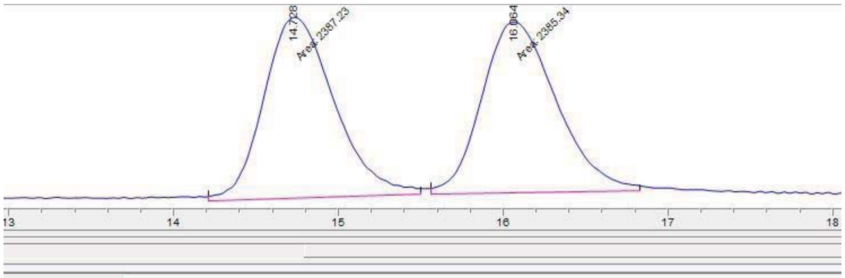

#	Time	Area	Height	Width	Area%	Symmetry
1	14.728	2387.2	79.6	0.4997	50.020	0.765
2	16.064	2385.3	75.3	0.5283	49.980	0.767

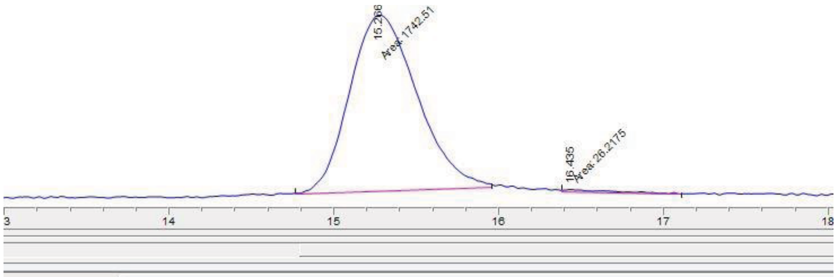

#	Time	Area	Height	Width	Area%	Symmetry
1	15.266	1742.5	60.5	0.4804	98.518	0.747
2	16.435	26.2	1.1	0.3803	1.482	0.123

***N*-((*2S,3S*)-1-Morpholino-3-phenylbutan-2-yl)aniline (6):**

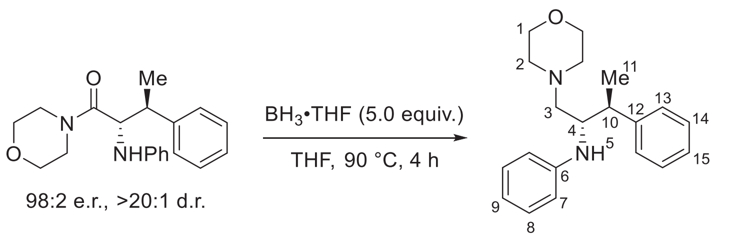

To a solution of **3ka** (32.4 mg, 0.10 mmol, 100 mol%) in anhydrous THF (1.0 mL), was added a solution of BH_3•THF in THF (1.0 M, 0.5 mL, 0.50 mmol, 500 mol%) under N_2 at r.t. Then the mixture was heated to 90 °C for 4 hours and the progress of the reaction was monitored by TLC. Upon completion, the mixture was cooled to 0 °C and water (5.0 mL) was slowly added. The mixture was extracted with EtOAc (approx. 3 × 10.0 mL). The combined organic phases were dried over anhydrous $MgSO_4$. The concentration of the filtrate *in vacuo* was followed by FCC (hexane/EtOAc 35:65) to afford the desired product **6** (21.4 mg, 69%, d.r. >20:1, e.r. = 99:1) as a colorless oil. $[\alpha]_D^{25}$ = -7.9 (c = 1.0 $CHCl_3$); **IR (thin film)** ν_{max}/cm^{-1}: 3366 (br), 2961 (s), 2852 (s), 1601 (s), 1504 (s), 1117 (s), 748 (s), 693 (s); **^{1}H NMR** (500 MHz, $CDCl_3$) δ 7.40 – 7.26 (m, 5H, $H^{13} + H^{14} + H^{15}$), 7.22 – 7.19 (m, 2H, H^8), 6.73 – 6.68 (m, 3H, $H^7 + H^9$), 3.78 – 3.61 (m, 6H, $H^1 + H^4 + H^5$), 3.49 – 3.44 (m, 1H, H^{10}), 2.56 – 2.17 (m, 6H, $H^2 + H^3$), 1.38 (d, *J* = 7.0 Hz, 3H, H^{11}); **^{13}C NMR** (126 MHz, $CDCl_3$) δ 148.2 (C^6), 142.4 (C^{12}), 129.3 (C^8), 128.4 (C^{14}), 128.3 (C^{13}), 126.5 (C^{15}), 117.2 (C^9), 113.2 (C^7), 66.9 (C^1), 59.3 (C^3), 54.5 (C^4), 53.6 (C^2), 39.9 (C^{10}), 16.2 (C^{11}); **HRMS** (ESI): calculated for $C_{20}H_{27}N_2O$ $[M+H]^+$ requires m/z 311.2118, found m/z 311.2133; **Chiral SFC:** DAICEL CHIRALCEL OD-H column (25 cm), CO_2:*i*-PrOH 90:10, 2.0 mL/min, 140 bar, 40 °C. Retention times: 5.8 mins (minor), 7.3 mins (major), e.r. = 99:1.

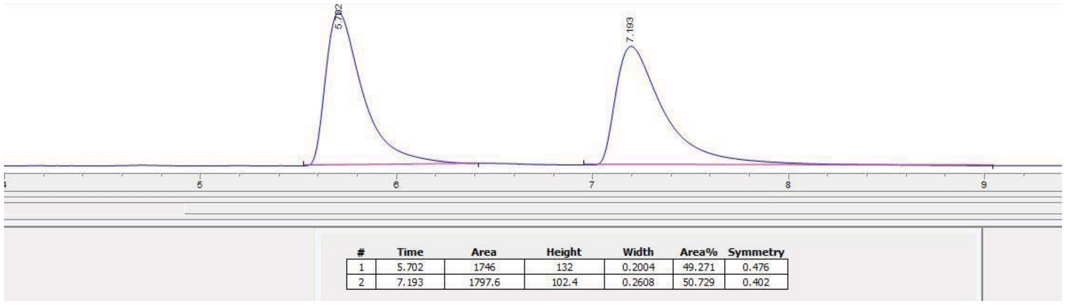

#	Time	Area	Height	Width	Area%	Symmetry
1	5.702	1746	132	0.2004	49.271	0.476
2	7.193	1797.6	102.4	0.2608	50.729	0.402

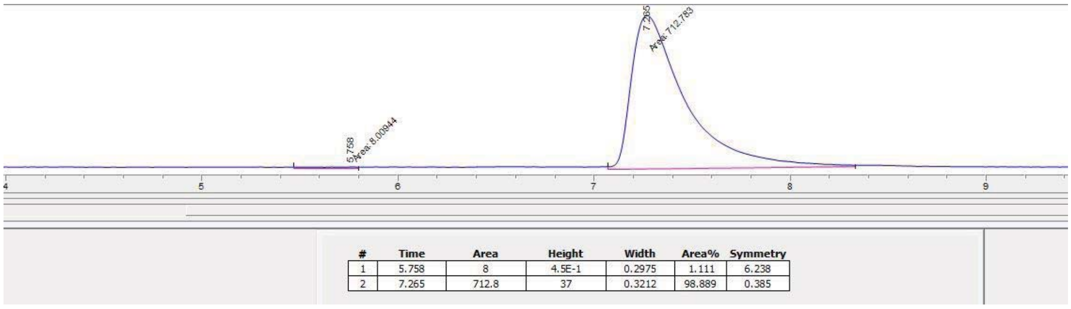

#	Time	Area	Height	Width	Area%	Symmetry
1	5.758	8	4.5E-1	0.2975	1.111	6.238
2	7.265	712.8	37	0.3212	98.889	0.385

(*2S,3S*)-1,3-Diphenyl-2-(phenylamino)butan-1-one (7):

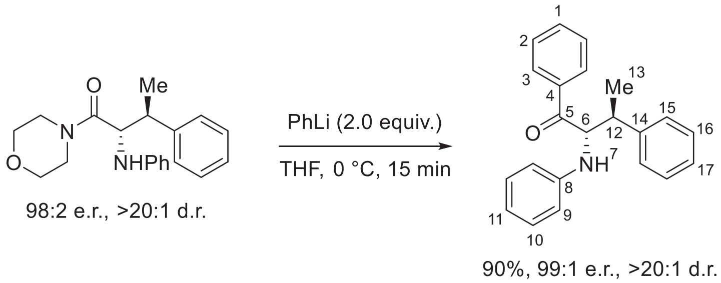

To a solution of **3ka** (32.4 mg, 0.10 mmol, 100 mol%) in anhydrous THF (1.0 mL), a solution of PhLi in Et_2O (1.9 M, 105 µL, 0.20 mmol, 200 mol%) was then added dropwise over 3 minutes under N_2 at 0 °C. Then the mixture was stirred at 0 °C for 15 minutes and the progress of the reaction was monitored by TLC. Upon completion, water (5.0 mL) was slowly added to the mixture to quench excess PhLi. The mixture was extracted with EtOAc (approx. 3 × 10.0 mL). The combined organic phases were dried over anhydrous $MgSO_4$. The concentration of the filtrate *in vacuo* was followed by FCC (EtOAc/hexane 10:90) to afford the desired product **7** (28.4 mg, 90%, d.r. >20:1, e.r. = 99:1) as a colorless oil. $[\alpha]_D^{25}$ = +66.4 (c = 1.0 $CHCl_3$); **IR (thin film)** ν_{max}/cm^{-1}: 3379 (br), 3027 (s), 1682 (s), 1601 (s), 1506 (s), 748 (s), 693 (s); **^{1}H NMR** (500 MHz, CD_2Cl_2) δ 7.93 (d, *J* = 7.5 Hz, 2H, H^3), 7.61 – 7.58 (m, 1H, H^1), 7.49 – 7.46 (m, 2H, H^2), 7.26 – 7.18 (m, 3H, H^{16} + H^{17}), 7.12 – 7.08 (m, 4H, H^{10} + H^{15}), 6.68 – 6.65 (m, 3H, H^9 + H^{11}), 5.19 (d, *J* = 5.0 Hz, 1H, H^6), 4.37 (br s, 1H, H^7), 3.45 – 3.40 (m, 1H, H^{12}), 1.44 (d, *J* = 7.0 Hz, 3H, H^{13}); **^{13}C NMR** (126 MHz, CD_2Cl_2) δ 200.4 (C^5), 147.8 (C^8), 141.4 (C^{14}),

136.3 (C^4), 133.5 (C^1), 129.3 (C^{10}), 128.9 (C^3), 128.4 (C^{16}), 128.3 (C^2), 128.2 (C^{15}), 127.0 (C^{17}), 118.2 (C^{11}), 114.0 (C^9), 63.5 (C^6), 42.9 (C^{12}), 18.2 (C^{13}); **HRMS** (ESI): calculated for $C_{22}H_{21}NONa$ $[M+Na]^+$ requires *m/z* 338.1515, found *m/z* 338.1524; **Chiral SFC:** DAICEL CHIRALCEL IE column (25 cm), CO_2:*i*-PrOH 95:5, 1.0 mL/min, 140 bar, 40 °C. Retention times: 41.4 mins (minor), 51.6 mins (major), e.r. = 99:1.

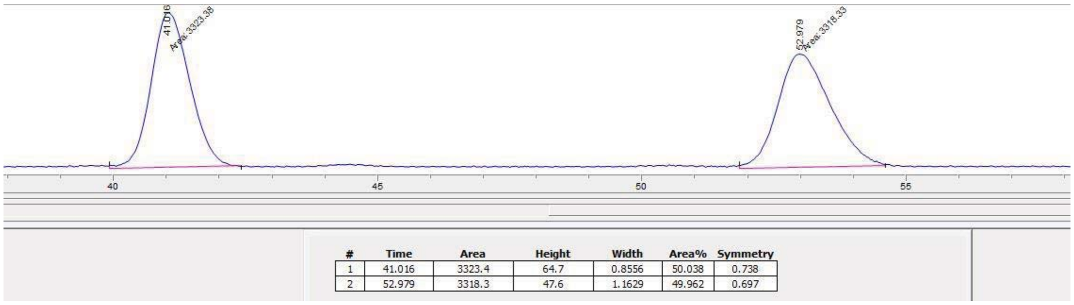

#	Time	Area	Height	Width	Area%	Symmetry
1	41.016	3323.4	64.7	0.8556	50.038	0.738
2	52.979	3318.3	47.6	1.1629	49.962	0.697

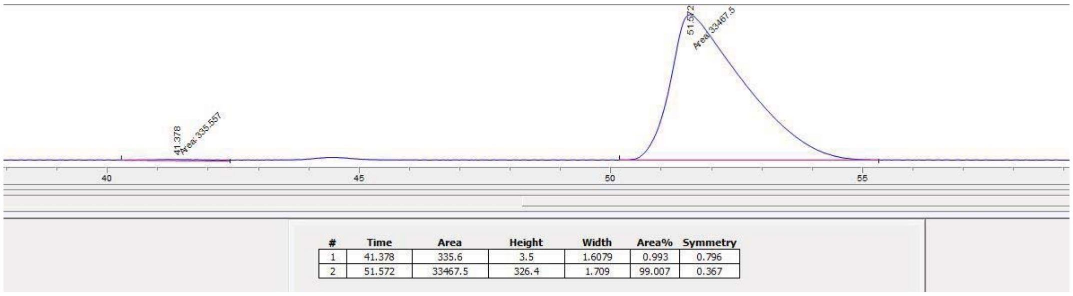

#	Time	Area	Height	Width	Area%	Symmetry
1	41.378	335.6	3.5	1.6079	0.993	0.796
2	51.572	33467.5	326.4	1.709	99.007	0.367

(*S*)-*N*-(2-Phenylpropyl)aniline (8):

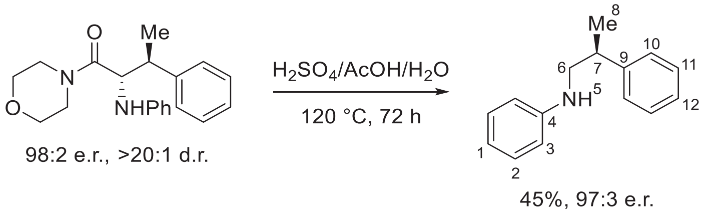

To a solution of **3ka** (32.4 mg, 0.10 mmol, 100 mol%) in AcOH (0.1 mL) and water (0.1 mL), concentrated sulfuric acid (0.3 mL) was then added dropwise over 2 minutes at r.t. The mixture was then stirred at 120 °C for 72 hours and the progress of the reaction was monitored by TLC. Upon completion, the mixture was cooled down and diluted with water (5.0 mL). This was followed by the addition of aq. 2M NaOH solution until a pH value of 9–10 was achieved. The mixture was extracted with EtOAc (approx. 3 × 10.0 mL). The combined organic phases were dried over anhydrous $MgSO_4$. The concentration of the filtrate *in vacuo* was followed by FCC (EtOAc/hexane 2:98) to afford the desired product **8** (9.5 mg, 45%, e.r. = 97:3) as a colorless oil. $[\alpha]_D^{25}$ = -24.8 (c = 1.0 $CHCl_3$); **IR (thin film)** ν_{max}/cm^{-1}: 3415 (br), 3026 (s), 2961 (s), 1602

(s), 1506 (s), 1320 (s), 748 (s), 693 (s); **^{1}H NMR** (500 MHz, CD_2Cl_2) δ 7.35 – 7.23 (m, 5H, H^{10} + H^{11} + H^{12}), 7.18 – 7.15 (m, 2H, H^2), 6.72 – 6.69 (m, 1H, H^1), 6.60 (d, *J* = 7.5 Hz, 2H, H^3), 4.09 (br s, 1H, H^5), 3.37 – 3.23 (m, 2H, H^6), 3.11 – 3.04 (m, 1H, H^7), 1.35 (d, *J* = 7.0 Hz, 3H, H^8); **HRMS** (ESI): calculated for $C_{15}H_{18}N$ $[M+H]^+$ requires m/z 212.1434, found m/z 212.1433; **Chiral SFC:** DAICEL CHIRALCEL IE column (25 cm), CO_2:*i*-PrOH 90:10, 2.0 mL/min, 140 bar, 40 °C. Retention times: 3.0 mins (major), 3.3 mins (minor), e.r. = 97:3.

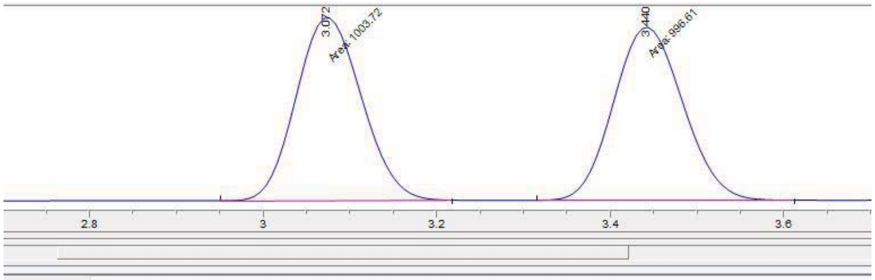

#	Time	Area	Height	Width	Area%	Symmetry
1	3.072	1003.7	188.8	0.0886	50.178	0.88
2	3.44	996.6	178	0.0933	49.822	0.891

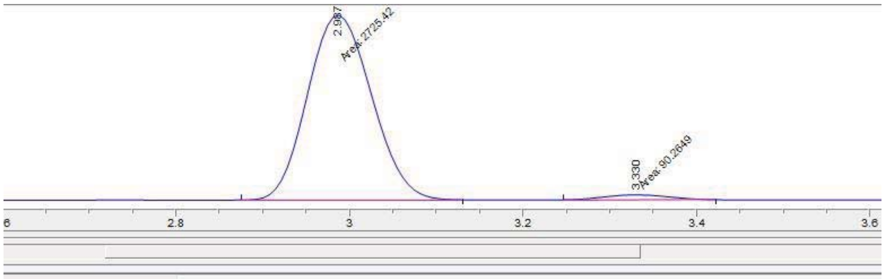

#	Time	Area	Height	Width	Area%	Symmetry
1	2.987	2725.4	522.2	0.087	96.794	0.873
2	3.33	90.3	15.8	0.0952	3.206	1.004

The spectroscopic properties were consistent with the data available in the literature.[21]

(*2S,3S*)-2-Amino-*N,N*-dimethyl-3-phenylbutanamide (9):

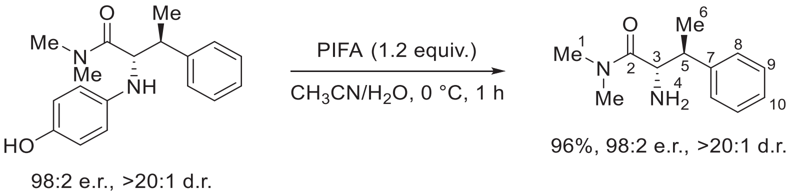

To a solution of **3pa** (29.8 mg, 0.10 mmol, 100 mol%) in CH_3CN (1.0 mL) and water (0.5 mL), (bis(trifluoroacetoxy)iodo)benzene (51.6 mg, 0.12 mmol, 120 mol%) was added portion-wise at 0 °C. The mixture was then stirred at 0 °C for 1 hour and the progress of the reaction was monitored by TLC. Upon completion, the mixture was cooled to 0 °C and a saturated aq. $Na_2S_2O_3$ solution (approx. 0.2 mL) was slowly added to quench excess PIFA. The mixture was

extracted with CH_2Cl_2 (approx. 5 × 5.0 mL). The combined organic phases were dried over anhydrous $MgSO_4$. The concentration of the filtrate *in vacuo* was followed by FCC (basic Al_2O_3, CH_2Cl_2/MeOH 90:10) to afford the desired product **9** (19.8 mg, 96%, d.r. >20:1, e.r. = 98:2) as a pale-yellow oil. $[\boldsymbol{\alpha}]_{\boldsymbol{D}}^{\boldsymbol{25}}$ = +10.1 (c = 1.0 $CHCl_3$); **^{1}H NMR** (500 MHz, CD_2Cl_2) δ 7.34 – 7.20 (m, 5H, $H^8 + H^9 + H^{10}$), 3.82 (d, *J* = 8.5 Hz, 1H, H^3), 3.05 – 3.00 (m, 4H, $H^1 + H^5$), 2.99 (s, 3H, $H^{1'}$), 1.58 (br s, 2H, H^4), 1.24 (d, *J* = 7.0 Hz, 3H, H^6); **HRMS** (ESI): calculated for $C_{12}H_{18}N_2ONa$ $[M+Na]^+$ requires m/z 229.1311, found m/z 229.1317; **Chiral SFC:** DAICEL CHIRALCEL IE column (25 cm), CO_2:MeOH (with 0.5% TEA) 80:20, 2.0 mL/min, 170 bar, 40 °C. Retention times: 6.5 mins (minor), 7.8 mins (major), e.r. = 98:2.

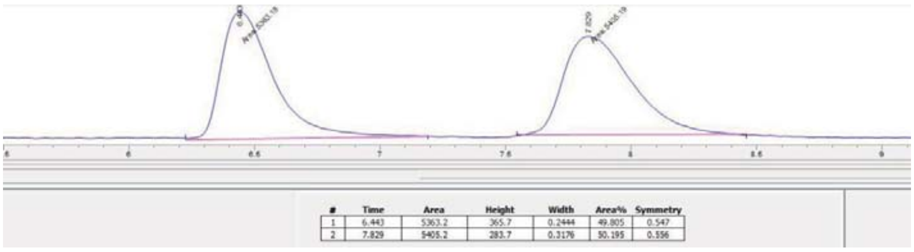

#	Time	Area	Height	Width	Area%	Symmetry
1	6.443	5363.2	365.7	0.2444	49.805	0.547
2	7.829	5405.2	283.7	0.3176	50.195	0.556

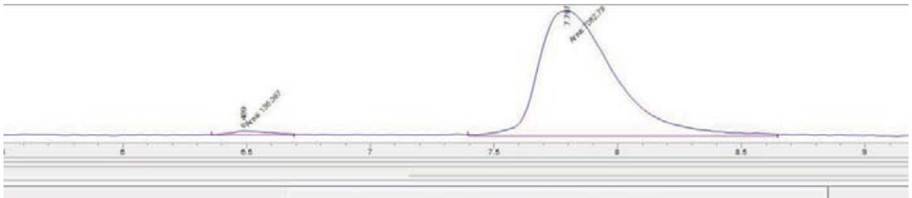

#	Time	Area	Height	Width	Area%	Symmetry
1	6.489	136.4	11.2	0.2032	1.889	0.487
2	7.797	7082.8	328.9	0.3589	98.111	0.59

The spectroscopic properties were consistent with the data available in the literature.[22]

Synthesis of (*2S*,*3S*)-2-Amino-3-phenyl-butyric acid hydrochloride 11, (*2S*,*3S*)-2-amino-3-phenylbutan-1-ol 11-1 and (*2S*,*3S*)-3-phenyl-2-(phenylamino)butan-1-ol 11-2:

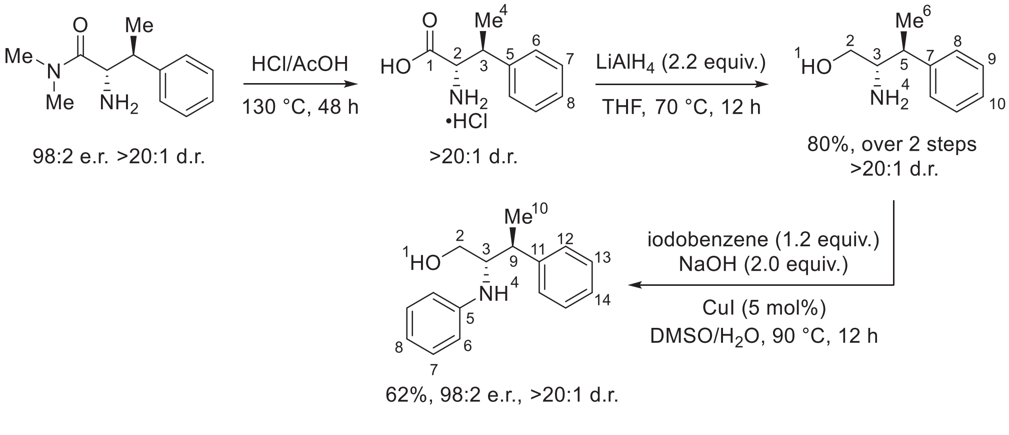

To a solution of **9** (103 mg, 0.50 mmol, 100 mol%) in AcOH (2.5 mL), concentrated hydrochloric acid (2.5 mL) was then added dropwise over 2 minutes at r.t. The mixture was then stirred at 130 °C for 48 hours and the progress of the reaction was monitored by TLC. After cooling to r.t., the solvents were removed *in vacuo*, the solid was then washed with EtOAc and dried under vacuum to afford **11** as a brown solid. The solid was used in the next step without further purification. **IR (thin film)** ν_{max}/cm^{-1}: 3386 (br), 2973 (s), 1732 (s), 1646 (s), 1497 (s), 1468 (s), 1228 (s), 703 (s); **^{1}H NMR** (500 MHz, D_2O) δ 7.48 – 7.37 (m, 5H, $H^6 + H^7 + H^8$), 4.12 (d, *J* = 7.5 Hz, 1H, H^2), 3.46 – 3.40 (m, 1H, H^3), 1.46 (d, *J* = 7.0 Hz, 3H, H^4); **^{13}C NMR** (126 MHz, D_2O) δ 171.8 (C^1), 139.3 (C^5), 129.3 (C^7), 128.2 (C^8), 127.8 (C^6), 59.2 (C^2), 34.5 (C^3), 17.0 (C^4). The enantioselectivity was confirmed by the subsequent arylation.

To the above solid, a solution of $LiAlH_4$ in THF (1.0 M, 1.1 mL, 1.10 mmol, 220 mol%) was then added dropwise over 2 minutes under N_2 at r.t. The mixture was then stirred at 70 °C for 12 hours and the progress of the reaction was monitored by TLC. Upon completion, the mixture was cooled to 0 °C and 5 drops of water were slowly added. This was followed by addition of aq. 4M NaOH solution (5 drops) and water (5 drops). The resulting suspension was then warmed to r.t. and stirred for 15 minutes before anhydrous Na_2SO_4 was added. The mixture was stirred for additional 15 minutes before filtration. The concentration of the filtrate *in vacuo* to afford the desired product **11-1** (66.0 mg, 80%, over 2 steps, d.r. >20:1) as a colorless oil. $[\alpha]_D^{25}$ = -17.7 (c = 1.0 $CHCl_3$); **IR (thin film)** ν_{max}/cm^{-1}: 3356 (br), 2963 (s), 2929 (s), 1582 (s), 1494 (s), 1453 (s), 1040 (s), 763 (s), 702 (s); **^{1}H NMR** (500 MHz, $CDCl_3$) δ 7.33 – 7.19 (m, 5H, $H^8 + H^9 + H^{10}$), 3.78 – 3.75 (m, 1H, H^2), 3.44 – 3.40 (m, 1H, $H^{2'}$), 2.96 – 2.92 (m, 1H, H^3), 2.71 – 2.64 (m, 1H, H^5), 1.99 (br s, 3H, $H^1 + H^4$), 1.26 (d, *J* = 7.0 Hz, 3H, H^6); **^{13}C NMR** (126 MHz, $CDCl_3$) δ 144.2 (C^7), 128.7 (C^9), 127.8 (C^8), 126.6 (C^{10}), 64.4 (C^2), 58.0 (C^3), 43.7 (C^5), 18.7 (C^6); **HRMS** (ESl): calculated for $C_{10}H_{16}NO$ $[M+H]^+$ requires m/z 166.1226, found m/z 166.1221. The enantioselectivity was confirmed by the subsequent arylation.

To a solution of **11-1** (21.5 mg, 0.13 mmol, 100 mol%) in DMSO (0.3 mL) and water (0.15 mL), iodobenzene (17.5 µL, 0.16 mmol, 120 mol%), NaOH (10.4 mg, 0.26 mmol, 200 mol%) and CuI (1.2 mg, 6.5 µmol, 5 mol%) were sequentially added under N_2 at r.t. The mixture was then stirred at 90 °C for 12 hours and the progress of the reaction was monitored by TLC. After cooling to r.t., the mixture was extracted with EtOAc (approx. 3 × 5.0 mL). The combined organic phases were dried over anhydrous $MgSO_4$. The concentration of the filtrate *in vacuo* was followed by FCC (EtOAc/hexane 25:75) to afford the desired product **11-2** (19.4 mg, 62%, d.r. >20:1, e.r. = 98:2) as a colorless oil. $[\alpha]_D^{25}$ = +27.4 (c = 1.0 $CHCl_3$); **IR (thin film)**

ν_{max}/cm^{-1}: 3399 (br), 2964 (s), 2928 (s), 1600 (s), 1495 (s), 1317 (s), 1261 (s), 747 (s), 691 (s); **^{1}H NMR** (500 MHz, $CDCl_3$) δ 7.33 – 7.21 (m, 5H, H^{12} + H^{13} + H^{14}), 7.16 – 7.13 (m, 2H, H^7), 6.73 – 6.70 (m, 1H, H^8), 6.62 (d, J = 7.5 Hz, 2H, H^6), 3.76 – 3.68 (m, 2H, H^2), 3.61 – 3.26 (m, 2H, H^3 + H^4), 3.19 – 3.14 (m, 1H, H^9), 1.91 (br s, 1H, H^1), 1.35 (d, J = 7.0 Hz, 3H, H^{10}); **^{13}C NMR** (126 MHz, $CDCl_3$) δ 147.9 (C^5), 142.7 (C^{11}), 129.3 (C^7), 128.6 (C^{13}), 127.9 (C^{12}), 126.8 (C^{14}), 118.0 (C^8), 113.9 (C^6), 62.5 (C^2), 60.2 (C^3), 41.0 (C^9), 18.1 (C^{10}); **HRMS** (ESI): calculated for $C_{16}H_{20}NO$ $[M+H]^+$ requires m/z 242.1539, found m/z 242.1543; **Chiral SFC:** YMC Chiral ART Cellulose-SC column (25 cm), CO_2:*i*-PrOH 92:8, 2.0 mL/min, 140 bar, 40 °C. Retention times: 6.5 mins (minor), 7.2 mins (major), e.r. = 98:2.

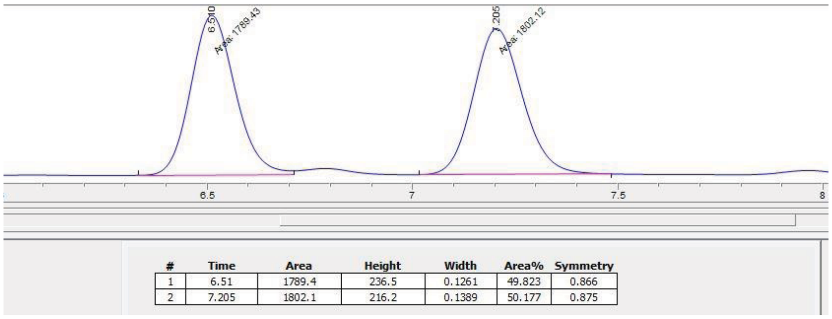

#	Time	Area	Height	Width	Area%	Symmetry
1	6.51	1789.4	236.5	0.1261	49.823	0.866
2	7.205	1802.1	216.2	0.1389	50.177	0.875

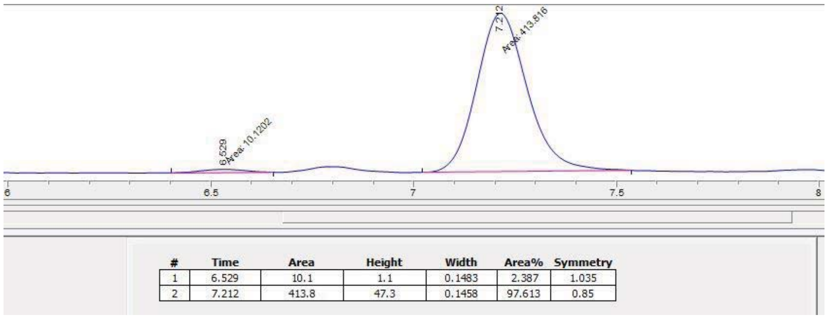

#	Time	Area	Height	Width	Area%	Symmetry
1	6.529	10.1	1.1	0.1483	2.387	1.035
2	7.212	413.8	47.3	0.1458	97.613	0.85

4-Bromo-*N*-((*2S,3S*)-1-(dimethylamino)-1-oxo-3-phenylbutan-2-yl)benzamide (10):

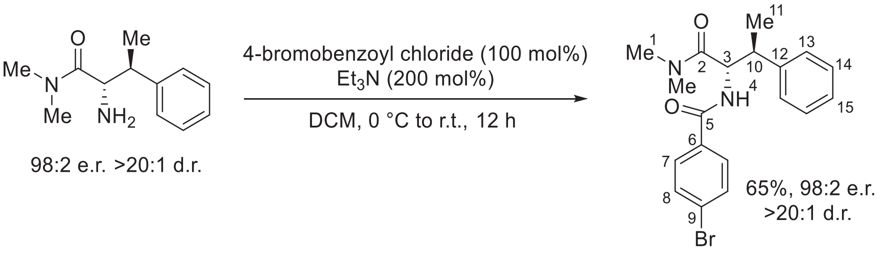

4-Bromobenzoyl chloride (21.8 mg, 0.10 mmol, 100 mol%) was slowly added, portion-wise, to a solution of **9** (20.6 mg, 0.10 mmol, 100 mol%), Et_3N (28.0 μL, 0.20 mmol, 200 mol%) and CH_2Cl_2 (1.0 mL) at 0 °C. The reaction was stirred at ambient temperature for 12 hours. Upon

completion, the reaction mixture was diluted with CH_2Cl_2 (5.0 mL) and transferred to a separatory funnel. The mixture was washed with aq. 1N HCl (10.0 mL). The combined organic phases were dried over anhydrous $MgSO_4$. The concentration of the filtrate *in vacuo* was followed by FCC (EtOAc/hexane 40:60) to afford the desired product **10** (25.2 mg, 65%, d.r. >20:1, e.r. = 98:2) as a colorless solid. **m.p.** = 154 – 156 °C (hexane/EtOAc); $[\boldsymbol{\alpha}]_{\boldsymbol{D}}^{\mathbf{25}}$ = +52.8 (c = 1.0 $CHCl_3$); **IR (thin film)** ν_{max}/cm^{-1}: 3310 (s), 2926 (s), 1621 (s), 1538 (s), 1484 (s), 1335 (s), 698 (s), 648 (s); **^{1}H NMR** (500 MHz, $CDCl_3$) δ 7.51 (s, 4H, $H^7 + H^8$), 7.34 – 7.23 (m, 5H, $H^{13} + H^{14} + H^{15}$), 6.96 (d, J = 8.5 Hz, 1H, H^4), 5.35 (t, J = 8.0 Hz, 1H, H^3), 3.46 – 3.40 (m, 1H, H^{10}), 2.97 – 2.96 (m, 6H, H^1), 1.39 (d, J = 7.0 Hz, 3H, H^{11}); **^{13}C NMR** (126 MHz, $CDCl_3$) δ 170.7 (C^2), 165.7 (C^5), 141.6 (C^{12}), 132.8 (C^6), 131.6 (C^8), 128.6 (C^7), 128.5 (C^{14}), 127.8 (C^{13}), 127.1 (C^{15}), 126.1 (C^9), 53.7 (C^3), 42.9 (C^{10}), 37.3 (C^1), 35.7 ($C^{1'}$), 16.9 (C^{11}); **HRMS** (ESI): calculated for $C_{19}H_{21}BrN_2O_2Na$ $[M+Na]^+$ requires m/z 411.0679, found m/z 411.0682; **Chiral SFC:** DAICEL CHIRALCEL IE column (25 cm), CO_2:*i*-PrOH 80:20, 2.0 mL/min, 170 bar, 40 °C. Retention times: 7.2 mins (minor), 9.3 mins (major), e.r. = 98:2.

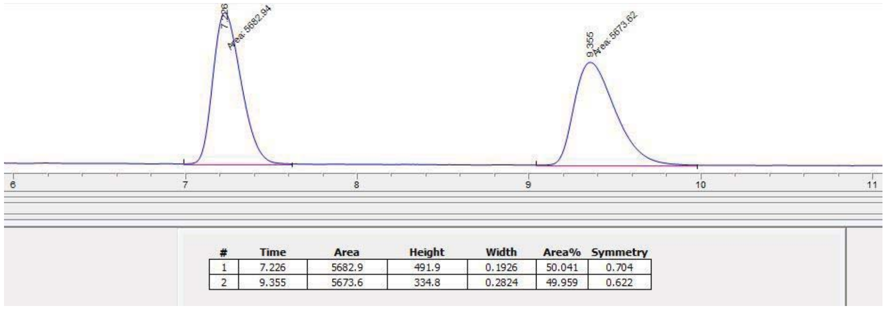

#	Time	Area	Height	Width	Area%	Symmetry
1	7.226	5682.9	491.9	0.1926	50.041	0.704
2	9.355	5673.6	334.8	0.2824	49.959	0.622

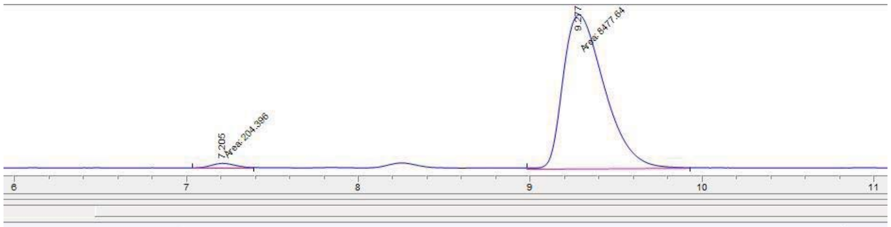

#	Time	Area	Height	Width	Area%	Symmetry
1	7.205	204.4	17.6	0.1937	2.354	0.831
2	9.277	8477.6	503.8	0.2805	97.646	0.591

The structure of this compound was determined by single crystal X-ray diffraction of crystals grown from EtOAc/hexane. See CCDC **2245009**.

(*2S,3S*)-2-Amino-*N*,3-diphenylbutanamide (12):

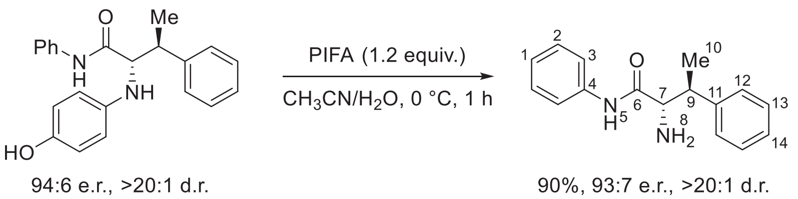

To a solution of **3la** (34.6 mg, 0.10 mmol, 100 mol%) in CH_3CN (1.0 mL) and water (0.5 mL), (bis(trifluoroacetoxy)iodo)benzene (51.6 mg, 0.12 mmol, 120 mol%) was slowly added, portion-wise, at 0 °C. The mixture was then stirred at 0 °C for 1 hour and the progress of the reaction was monitored by TLC. Upon completion, saturated aq. $Na_2S_2O_3$ solution (0.2 mL) was slowly added to the mixture to quench excess PIFA at 0 °C. The mixture was extracted with CH_2Cl_2 (approx. 5 × 5.0 mL). The combined organic phases were dried over anhydrous $MgSO_4$. The concentration of the filtrate *in vacuo* was followed by FCC (basic Al_2O_3, EtOAc/hexane 65:35) to afford the desired product **12** (22.9 mg, 90%, d.r. >20:1, e.r. = 93:7) as a pale-yellow oil. $[\boldsymbol{\alpha}]_{\boldsymbol{D}}^{\mathbf{25}}$ = -84.1 (c = 1.0 $CHCl_3$); **IR (thin film)** ν_{max}/cm^{-1}: 3301 (br), 3060 (s), 2928 (s), 1666 (s), 1600 (s), 1523 (s), 1443 (s), 755 (s), 701 (s); **^{1}H NMR** (500 MHz, $CDCl_3$) δ 9.15 (s, 1H, H^5), 7.54 (d, J = 8.0 Hz, 2H, H^3), 7.35 – 7.22 (m, 7H, $H^2 + H^{12} + H^{13} + H^{14}$), 7.11 – 7.08 (m, 1H, H^1), 3.61 (d, J = 6.0 Hz, 1H, H^7), 3.47 – 3.41 (m, 1H, H^9), 1.93 (br s, 2H, H^8), 1.42 (d, J = 7.0 Hz, 3H, H^{10}); **^{13}C NMR** (126 MHz, $CDCl_3$) δ 172.0 (C^6), 141.5 (C^{11}), 137.6 (C^4), 128.9 (C^2), 128.7 (C^{13}), 128.0 (C^{12}), 127.1 (C^{14}), 124.1 (C^1), 119.6 (C^3), 61.3 (C^7), 42.6 (C^9), 18.4 (C^{10}); **HRMS** (ESl): calculated for $C_{16}H_{19}N_2O$ $[M+H]^+$ requires m/z 255.1492, found m/z 255.1494; **Chiral SFC:** DAICEL CHIRALCEL IE column (25 cm), CO_2:MeOH (with 0.5% TEA) 90:10, 2.0 mL/min, 140 bar, 40 °C. Retention times: 23.9 mins (minor), 33.9 mins (major), e.r. = 93:7.

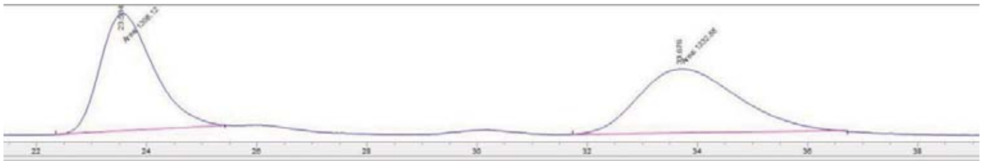

#	Time	Area	Height	Width	Area%	Symmetry
1	23.534	1308.1	19.3	1.1316	49.531	0.651
2	33.676	1332.9	10.6	2.098	50.469	0.7

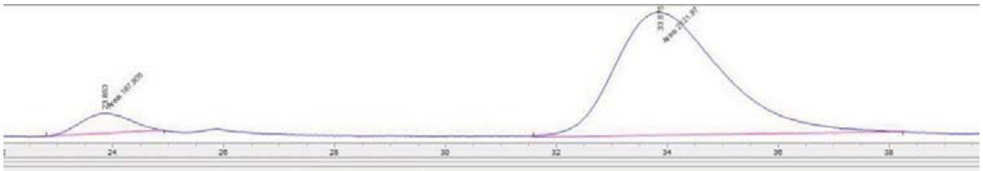

#	Time	Area	Height	Width	Area%	Symmetry
1	23.853	167.9	2.6	1.0579	7.333	0.973
2	33.875	2122	15.7	2.2471	92.667	0.711

(*2S,3S*)-2-Amino-3-(1*H*-indol-3-yl)-*N,N*-dimethylbutanamide (13):

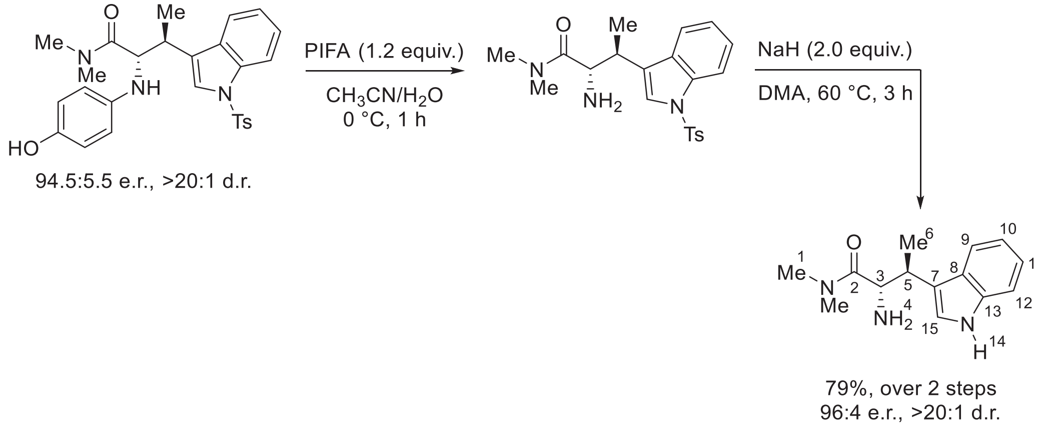

To a solution of **3pf** (49.1 mg, 0.10 mmol, 100 mol%) in CH_3CN (1.0 mL) and water (0.5 mL), (bis(trifluoroacetoxy)iodo)benzene (51.6 mg, 0.12 mmol, 120 mol%) was slowly added, portion-wise, at 0 °C. The mixture was then stirred at 0 °C for 1 hour and the progress of the reaction was monitored by TLC. Upon completion, saturated aq. $Na_2S_2O_3$ solution (0.2 mL) was slowly added to the mixture to quench excess PIFA at 0 °C. The mixture was extracted with CH_2Cl_2 (approx. 5 × 5.0 mL). The combined organic phases were dried over anhydrous $MgSO_4$. The concentration of the filtrate *in vacuo* was followed by FCC (basic Al_2O_3, CH_2Cl_2/MeOH 90:10) to afford the amide intermediate (38.3 mg, 96%, d.r. >20:1) as a pale-yellow oil.

The above amide intermediate (39.9 mg, 0.10 mmol, 100 mol%) in DMA (0.50 mL) was slowly added to a solution of NaH (8.00 mg, 0.20 mmol, 200 mol%) in DMA (0.25 mL) at r.t. The mixture was then stirred at 60 °C for 3 hours and the progress of the reaction was monitored

by TLC. After cooling to 0 °C, water (approx. 5.0 mL) was added to the reaction mixture and then extracted with EtOAc (approx. 5 × 10.0 mL). The combined organic phases were dried over anhydrous $MgSO_4$. The concentration of the filtrate *in vacuo* was followed by FCC (basic Al_2O_3, CH_2Cl_2/MeOH 80:20) to afford the desired product **13** (20.1 mg, 82%, d.r. >20:1, e.r. = 96:4) as a pale-yellow solid. **m.p.** = 92 – 94 °C (hexane/EtOAc); $[\boldsymbol{\alpha}]_{\boldsymbol{D}}^{\mathbf{25}}$ = +42.7 (c = 1.0 $CHCl_3$); **IR (thin film)** ν_{max}/cm^{-1}: 3261 (br), 2925 (s), 1631 (s), 1458 (s), 1343 (s), 1110 (s), 742 (s); **^{1}H NMR** (500 MHz, $CDCl_3$) δ 8.44 (s, 1H, H^{14}), 7.60 (d, J = 8.0 Hz, 1H, H^9), 7.36 (d, J = 8.0 Hz, 1H, H^{12}), 7.19 – 7.16 (m, 1H, H^{11}), 7.11 – 7.08 (m, 1H, H^{10}), 7.05 (s, 1H, H^{15}), 4.03 (d, J = 8.0 Hz, 1H, H^3), 3.40 – 3.35 (m, 1H, H^5), 3.01 (s, 3H, H^1), 2.96 (s, 3H, $H^{1'}$), 2.17 (br s, 2H, H^4), 1.36 (d, J = 7.0 Hz, 3H, H^6); **^{13}C NMR** (126 MHz, $CDCl_3$) δ 173.9 (C^2), 136.5 (C^{13}), 126.9 (C^8), 122.1 (C^{11} + C^{15}), 119.4 (C^{10}), 119.0 (C^9), 117.2 (C^7), 111.5 (C^{12}), 55.7 (C^3), 37.4 (C^1 + C^5), 35.8 ($C^{1'}$), 17.9 (C^6); **HRMS** (ESI): calculated for $C_{14}H_{20}N_3O$ $[M+H]^+$ requires m/z 246.1601, found m/z 246.1605; **Chiral SFC:** DAICEL CHIRALCEL IE column (25 cm), CO_2:MeOH (with 0.5% TEA) 80:20, 3.0 mL/min, 170 bar, 40 °C. Retention times: 7.7 mins (minor), 9.4 mins (major), e.r. = 96:4.

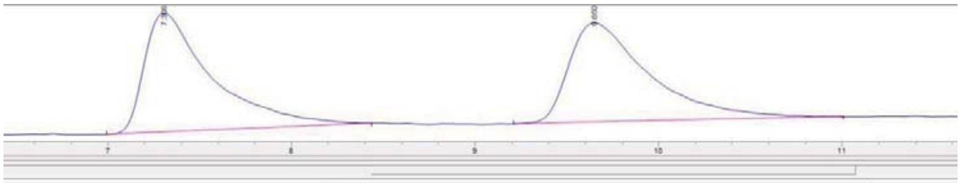

#	Time	Area	Height	Width	Area%	Symmetry
1	7.306	334.5	13.1	0.3665	49.977	0.374
2	9.65	334.8	10.9	0.4459	50.023	0.424

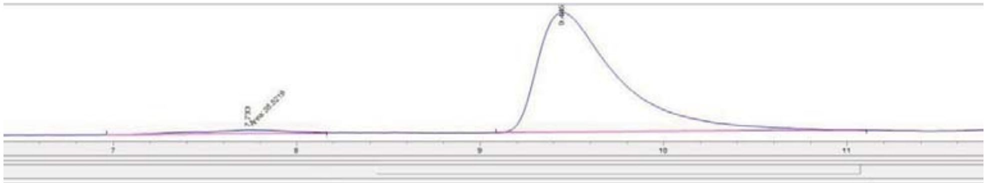

#	Time	Area	Height	Width	Area%	Symmetry
1	7.733	38.5	1E0	0.6429	3.939	1.307
2	9.445	939.3	30.9	0.4425	96.061	0.4

Formal synthesis of (*S*)-Lorcaserin:

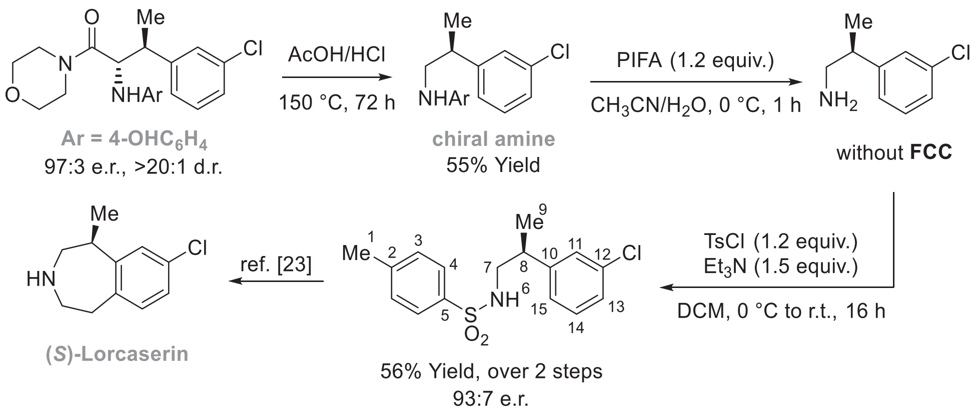

To a solution of **3wk** (187 mg, 0.50 mmol, 100 mol%) in AcOH (0.4 mL), concentrated hydrochloric acid (2.0 mL) was added dropwise over 2 minutes at r.t. The mixture was then stirred at 150 °C for 72 hours and the progress of the reaction was monitored by TLC. Upon completion, the mixture was cooled to r.t. and diluted with water (5.0 mL). This was followed by addition of aq. 2M NaOH solution until a pH value of 9–10 was achieved. The mixture was extracted with EtOAc (approx. 3 × 10.0 mL). The combined organic phases were dried over anhydrous $MgSO_4$. The concentration of the filtrate *in vacuo* was followed by FCC (EtOAc/hexane 25:75) to afford the amine intermediate **14** (71.8 mg, 55%) as a colorless oil.

To a solution of **14** (39.2 mg, 0.15 mmol, 100 mol%) in CH_3CN (1.5 mL) and water (0.75 mL), (bis(trifluoroacetoxy)iodo)benzene (77.4 mg, 0.18 mmol, 120 mol%) was added portion-wise, at 0 °C. The mixture was then stirred at 0 °C for 1 hour and the progress of the reaction was monitored by TLC. Upon completion, the mixture was cooled to 0 °C and a saturated aq. $Na_2S_2O_3$ solution (0.2 mL) was slowly added to quench excess PIFA. This was followed by the addition of aq. 2M NaOH solution until a pH value of 10–11 was achieved. The mixture was extracted with CH_2Cl_2 (approx. 3 × 5.0 mL). The combined organic phases were dried over anhydrous $MgSO_4$. The filtrate was concentrated *in vacuo* to afford the free amine intermediate **14-1** as a pale-yellow oil. The oil was used in the next step without further purification.

To a solution of the above free amine intermediate, Et_3N (32.0 μL, 0.23 mmol, 150 mol%) in CH_2Cl_2 (1.5 mL), and 4-toluenesulfonyl chloride (34.3 mg, 0.18 mmol, 120 mol%) were added portion-wise at 0 °C. The reaction was stirred at r.t. for 16 hours. Upon completion, the reaction mixture was diluted with CH_2Cl_2 (5.0 mL) and transferred to a separatory funnel and washed with aq. 1N HCl (10.0 mL). The combined organic phases were dried over anhydrous $MgSO_4$.

The concentration of the filtrate *in vacuo* was followed by FCC (EtOAc/hexane 25:75) to afford the Ts-protected amine **14-2** (26.9 mg, 56%, over 2 steps, e.r. = 93:7) as a pale-yellow oil. $[\boldsymbol{\alpha}]_D^{25}$ = -10.7 (c = 1.0 $CHCl_3$); **^{1}H NMR** (500 MHz, $CDCl_3$) δ 7.65 (d, *J* = 8.0 Hz, 2H, H^4), 7.29 (d, *J* = 8.0 Hz, 2H, H^3), 7.22 – 7.18 (m, 2H, ArH), 6.99 – 6.96 (m, 2H, ArH), 4.30 – 4.28 (m, 1H, H^6), 3.21 – 3.16 (m, 1H, H^7), 3.01 – 2.96 (m, 1H, $H^{7'}$), 2.88 – 2.82 (m, 1H, H^8), 2.43 (s, 3H, H^1), 1.21 (d, *J* = 6.9 Hz, 3H, H^9); **Chiral SFC:** YMC Chiral ART Cellulose-SC column (25 cm), CO_2:*i*-PrOH 80:20, 2.0 mL/min, 165 bar, 40 °C. Retention times: 4.9 mins (minor), 5.2 mins (major), e.r. = 93:7.

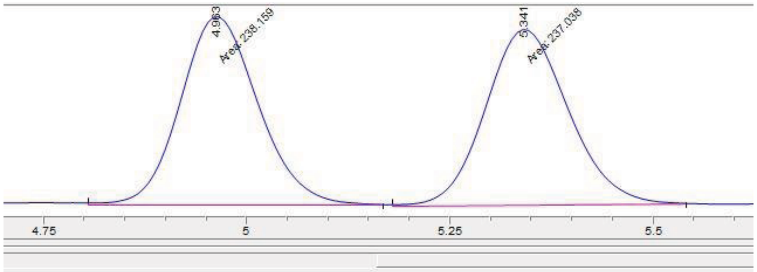

#	Time	Area	Height	Width	Area%	Symmetry
1	4.963	238.2	35.6	0.1116	50.118	0.862
2	5.341	237	33.2	0.1191	49.882	0.874

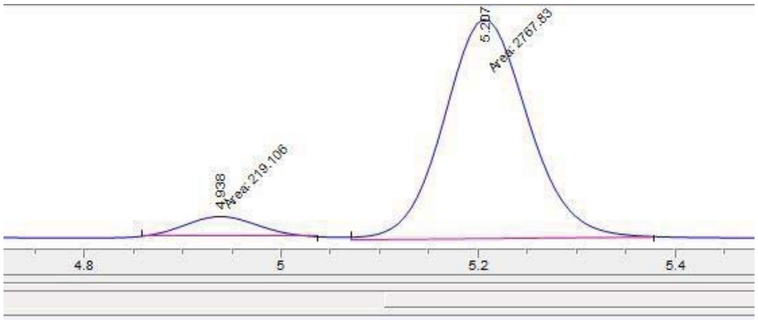

#	Time	Area	Height	Width	Area%	Symmetry
1	4.938	219.1	42.5	0.0858	7.335	0.906
2	5.207	2767.8	463.2	0.0996	92.665	0.919

The spectroscopic properties were consistent with the data available in the literature.[23]

Evaluation of other directing groups

Different directing groups, including carbamate (**1a**), sulfonamide (**1b**), amide (**1c**), N-benzhydryl (**1d**) and the free amine (**1e**), have been evaluated and all were less efficient than the NHPh group.

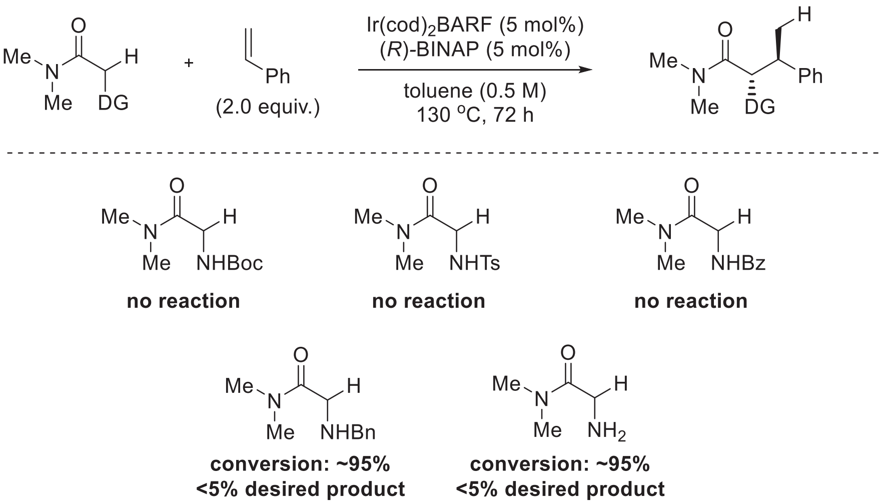

Mechanistic studies

Other possible reaction mechanisms were considered. One option starts with reversible carbonyl-directed N-H oxidative addition to generate **A/A'**. From **A/A'**, reversible dehydrogenation forms imine **B** and an iridium-hydride species. The alkyl iridium species **C** (or **C'**), which is reversibly generated from the migratory insertion of the alkene into the iridium-hydride species, undergoes the addition to imine **B** to afford **D**. Finally, N-H bond-forming reductive elimination delivers the desired product **3fa** and regenerates the Ir(I) catalyst. An alternate oxidative coupling pathway could also provide the product from imine **B**.

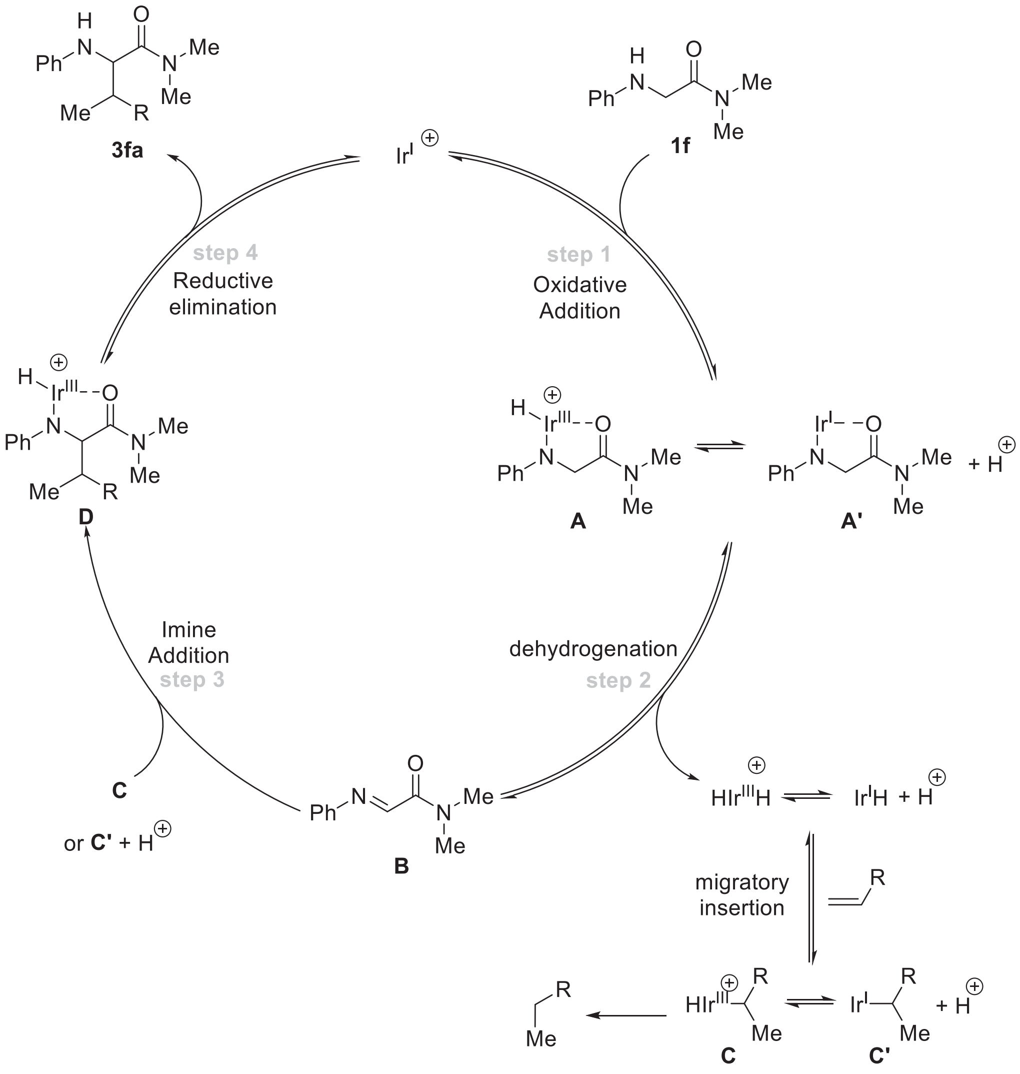

Alternate oxidative coupling pathway:

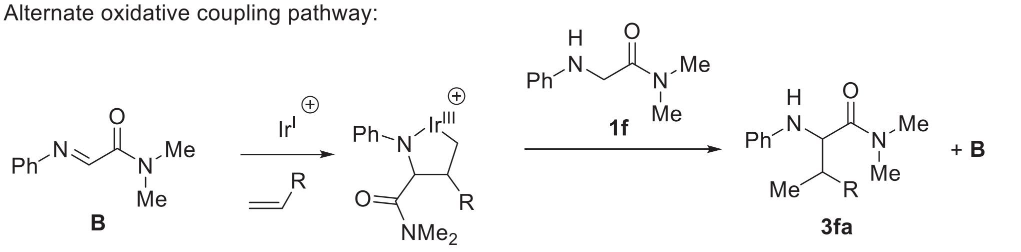

To probe the possibility of the above mechanisms, a control experiment was conducted in the presence of 1,2-diphenylethyne: Substrate **1f** (17.8 mg, 0.10 mmol, 100 mol%), [Ir(cod)$_2$]BARF (6.36 mg, 5.00 μmol, 5 mol%) and *rac*-BINAP (3.11 mg, 5.00 μmol, 5 mol%) were placed in an oven-dried resealable tube equipped with a magnetic stirrer bar and capped with a rubber septum. The tube was evacuated and refilled with N_2 (this operation was repeated three times) and then 1,2-diphenylethyne (31.4 μL, 0.20 mmol, 200 mol%), styrene (23.0 μL, 0.20 mmol, 200 mol%) and toluene (0.2 mL) were added sequentially. The tube was sealed

with a screw cap and heated to 130 °C for 24 hours with vigorous stirring. Upon completion, the reaction mixture was cooled to r.t. and then the internal standard (1,3,5-trimethoxybenzene, 16.8 mg) was added. The mixture was concentrated *in vacuo*. The yield of 1,2-diphenylethene was calculated by integration of the ^{1}H NMR spectrum. *The control experiment indicates that an iridium-dihydride species is not involved in the formation of* ***3fa****, and is also inconsistent with an alternate oxidative coupling pathway, because products from oxidative coupling of the alkyne were not observed.*

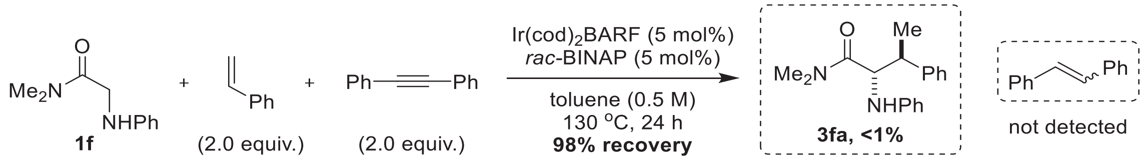

Another control experiment was conducted in the presence of an enone: Substrate **1f** (17.8 mg, 0.10 mmol, 100 mol%), (*E*)-4-phenylbut-3-en-2-one (29.2 mg, 0.20 mmol, 200 mol%), [Ir(cod)$_2$]BARF (6.36 mg, 5.00 µmol, 5 mol%) and *rac*-BINAP (3.11 mg, 5.00 µmol, 5 mol%) were placed in an oven-dried resealable tube equipped with a magnetic stirrer bar and capped with a rubber septum. The tube was evacuated and refilled with N_2 (this operation was repeated three times) and then styrene (23.0 µL, 0.20 mmol, 200 mol%) and toluene (0.2 mL) were added sequentially. The tube was sealed with a screw cap and heated to 130 °C for 24 hours with vigorous stirring. Upon completion, the reaction mixture was cooled to r.t. and then the internal standard (1,3,5-trimethoxybenzene, 16.8 mg) was added. The mixture was concentrated *in vacuo*. The yield of 4-phenylbutan-2-one was calculated by integration of the ^{1}H NMR spectrum. *The control experiment indicates that an iridium-dihydride species is not involved in the formation of* ***3fa****, and is also inconsistent with an alternate oxidative coupling pathway, because product from oxidative coupling of the enone were not observed.*

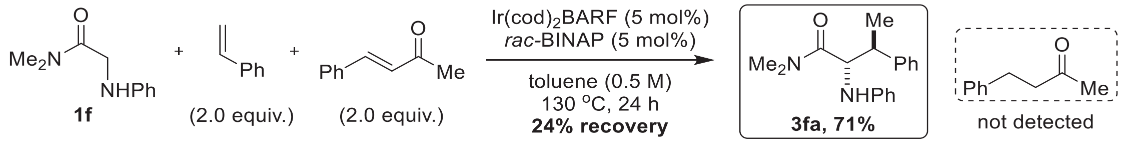

A cross over experiment involving the addition of an imine was also undertaken: Substrate **1f** (17.8 mg, 0.10 mmol, 100 mol%), 1-phenyl-2-(*p*-tolylimino)ethan-1-one (22.3 mg, 0.10 mmol, 100 mol%), [Ir(cod)$_2$]BARF (6.36 mg, 5.00 µmol, 5 mol%) and *rac*-BINAP (3.11 mg, 5.00 µmol, 5 mol%) were placed in an oven-dried resealable tube equipped with a magnetic stirrer bar and capped with a rubber septum. The tube was evacuated and refilled with N_2 (this

operation was repeated three times) and then styrene (23.0 μL, 0.20 mmol, 200 mol%) and toluene (0.2 mL) were added sequentially. The tube was sealed with a screw cap and heated to 90 °C for 24 hours with vigorous stirring. Upon completion, the reaction mixture was cooled to r.t. and then the internal standard (1,3,5-trimethoxybenzene, 16.8 mg) was added. The mixture was concentrated *in vacuo*. *The control experiment indicates that an iridium-dihydride species is not involved in the productive pathway, and is also inconsistent with an alternate oxidative coupling pathway.*

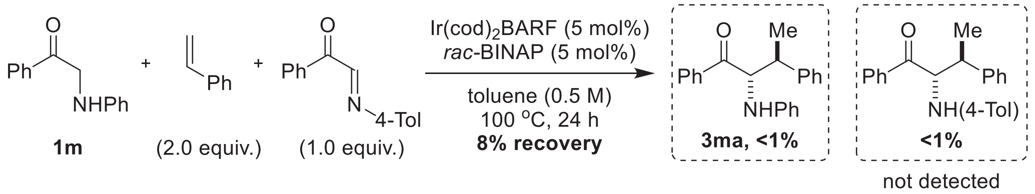

Supplementary Figure 3: Control experiments

A series of control experiments have been undertaken to elucidate key requirements for the alkylation process.

Supplementary Figure 3a: Substrate **1f** (17.8 mg, 0.10 mmol, 100 mol%) and *rac*-BINAP (3.11 mg, 5.00 μmol, 5 mol%) were placed in an oven-dried resealable tube equipped with a magnetic stirrer bar and capped with a rubber septum. The tube was evacuated and refilled with N_2 (this operation was repeated three times) and then styrene (23.0 μL, 0.20 mmol, 200 mol%) and toluene (0.2 mL) were added sequentially. The tube was sealed with a screw cap and heated to 130 °C for 72 hours with vigorous stirring. Upon completion, the solvent was removed under reduced pressure and the crude mixture was purified by FCC (hexane/EtOAc 60:40) to recover amide **1f**. *The control experiment shows that the process requires Ir(cod)₂BARF.*

Supplementary Figure 3b: [Ir(cod)$_2$]BARF (6.36 mg, 5.00 μmol, 5 mol%) and *rac*-BINAP (3.11 mg, 5.00 μmol, 5 mol%) were placed in an oven-dried resealable tube equipped with a magnetic stirrer bar and capped with a rubber septum. The tube was evacuated and refilled with N_2 (this operation was repeated three times) and then N^1,N^1-dimethyl-N^2-phenylethane-1,2-diamine (16.5 μL, 0.10 mmol, 100 mol%), styrene (23.0 μL, 0.20 mmol, 200 mol%) and toluene (0.2 mL) were added sequentially. The tube was sealed with a screw cap and heated to 130 °C for 72 hours with vigorous stirring. Upon completion, the solvent was removed under reduced pressure and the crude mixture was purified by FCC (hexane/EtOAc 90:10) to recover aniline **S3-1**. *The control experiment shows that the process requires a carbonyl unit.*

Supplementary Figure 3c: $[Ir(cod)_2]BARF$ (6.36 mg, 5.00 μmol, 5 mol%) and *rac*-BINAP (3.11 mg, 5.00 μmol, 5 mol%) were placed in an oven-dried resealable tube equipped with a magnetic stirrer bar and capped with a rubber septum. The tube was evacuated and refilled with N_2 (this operation was repeated three times) and then *N*-propylaniline (14.5 μL, 0.10 mmol, 100 mol%), styrene (23.0 μL, 0.20 mmol, 200 mol%) and toluene (0.2 mL) were added sequentially. The tube was sealed with a screw cap and heated to 130 °C for 72 hours with vigorous stirring. Upon completion, the solvent was removed under reduced pressure and the crude mixture was purified by FCC (hexane/EtOAc 90:10) to recover aniline **S3-3**. *The control experiment shows that the process requires a carbonyl unit.*

Supplementary Figure 3d: $[Ir(cod)_2]BARF$ (6.36 mg, 5.00 μmol, 5 mol%) and *rac*-BINAP (3.11 mg, 5.00 μmol, 5 mol%) were placed in an oven-dried resealable tube equipped with a magnetic stirrer bar and capped with a rubber septum. The tube was evacuated and refilled with N_2 (this operation was repeated three times) and then DMA (9.3 μL, 0.10 mmol, 100 mol%), styrene (23.0 μL, 0.20 mmol, 200 mol%) and toluene (0.2 mL) were added sequentially. The tube was sealed with a screw cap and heated to 130 °C for 72 hours with vigorous stirring. Upon completion, the solvent was removed under reduced pressure and the presence of DMA in the crude mixture was confirmed by ^1H NMR spectroscopy using 1,3,5-trimethoxybenzene as the internal standard. *The control experiment shows that the process requires an NHAr unit.*

Supplementary Figure 3e: 2-(Diphenylamino)-*N,N*-dimethylacetamide (25.4 mg, 0.10 mmol, 100 mol%), $[Ir(cod)_2]BARF$ (6.36 mg, 5.00 μmol, 5 mol%) and *rac*-BINAP (3.11 mg, 5.00 μmol, 5 mol%) were placed in an oven-dried resealable tube equipped with a magnetic stirrer bar and capped with a rubber septum. The tube was evacuated and refilled with N_2 (this operation was repeated three times) and then styrene (23.0 μL, 0.20 mmol, 200 mol%) and toluene (0.2 mL) were added sequentially. The tube was sealed with a screw cap and heated to 130 °C for 72 hours with vigorous stirring. Upon completion, the solvent was removed under reduced pressure and the crude mixture was purified by FCC (hexane/EtOAc 75:25) to recover amide **S3-5**. *The control experiment shows that the process requires an NH unit.*

Supplementary Figure 3f: *N,N*-dimethyl-2-(methyl(phenyl)amino)acetamide (19.2 mg, 0.10 mmol, 100 mol%), $[Ir(cod)_2]BARF$ (6.36 mg, 5.00 μmol, 5 mol%) and *rac*-BINAP (3.11 mg, 5.00 μmol, 5 mol%) were placed in an oven-dried resealable tube equipped with a magnetic stirrer bar and capped with a rubber septum. The tube was evacuated and refilled with N_2 (this operation was repeated three times) and then styrene (23.0 μL, 0.20 mmol, 200 mol%) and

toluene (0.2 mL) were added sequentially. The tube was sealed with a screw cap and heated to 130 °C for 72 hours with vigorous stirring. Upon completion, the solvent was removed under reduced pressure and the crude mixture was purified by FCC (hexane/EtOAc 75:25) to recover amide **S3-7** and obtain product ***rac*-3fa**. *The control experiment shows that the process requires an NH unit. The formation of **3fa** is attributed to an Ir-catalyzed demethylation pathway.*

Supplementary Figure 3. Control experiments.

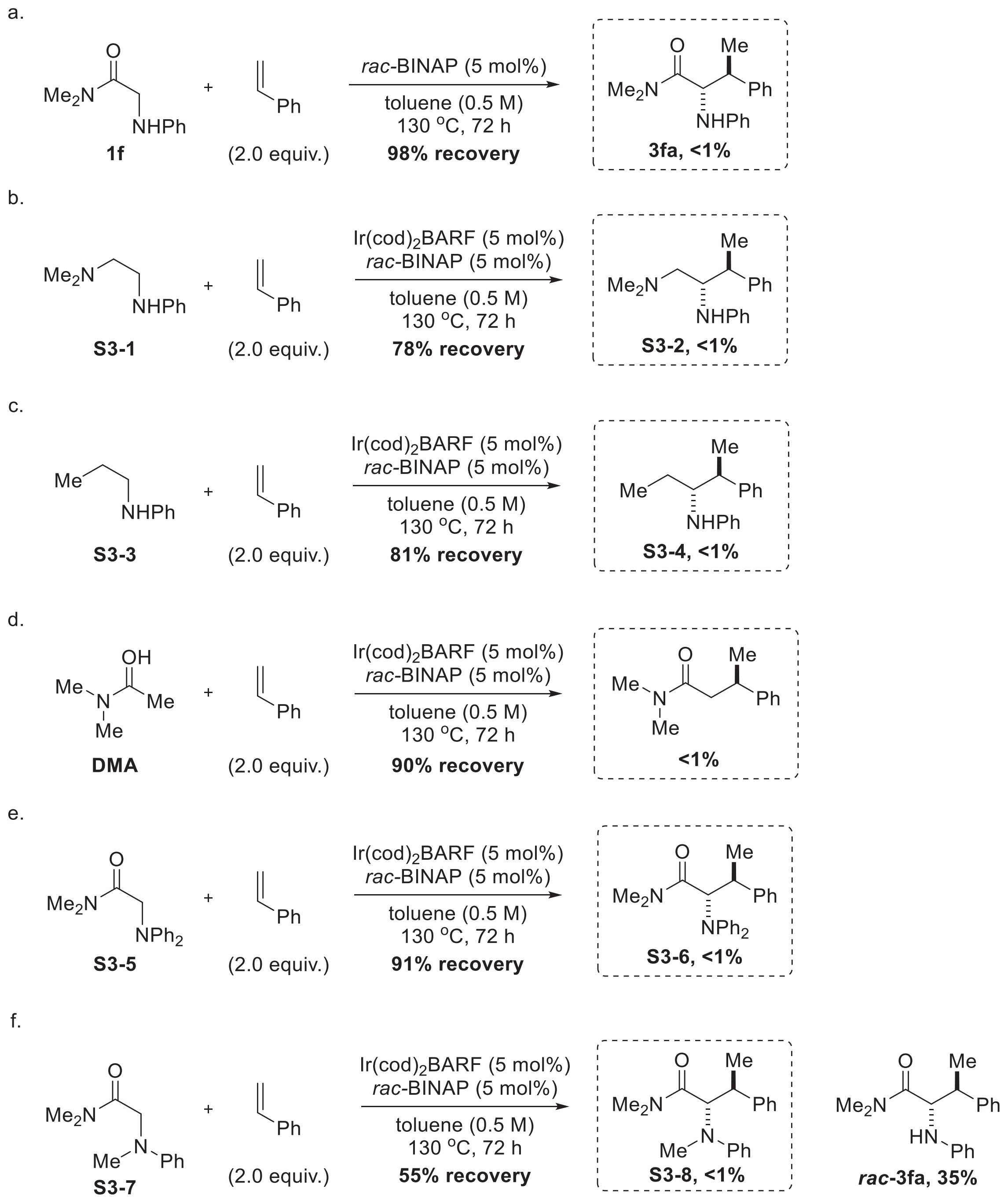

Supplementary Figure 4: Deuterium exchange and labelling experiments

Supplementary Figure 4a: Substrate **1f** (17.8 mg, 0.10 mmol, 100 mol%) and *rac*-BINAP (3.11 mg, 5.00 μmol, 5 mol%) were placed in an oven-dried resealable tube equipped with a magnetic stirrer bar and capped with a rubber septum. The tube was evacuated and refilled with N_2 (this operation was repeated three times) and then styrene (23.0 μL, 0.20 mmol, 200 mol%), toluene (0.2 mL) and D_2O (18.0 μL, 1.00 mmol, 1000 mol%) were added sequentially. The tube was sealed with a screw cap and heated to 130 °C for 72 hours with vigorous stirring. Upon completion, the solvent was removed under reduced pressure and the crude mixture was purified by FCC (hexane/EtOAc 60:40) to recover amide **1f**. Deuterium incorporation was calculated by integration of the ^{1}H NMR spectrum.

Supplementary Figure 4b: Substrate **1f** (17.8 mg, 0.10 mmol, 100 mol%), [Ir(cod)$_2$]BARF (6.36 mg, 5.00 μmol, 5 mol%) and *rac*-BINAP (3.11 mg, 5.00 μmol, 5 mol%) were placed in an oven-dried resealable tube equipped with a magnetic stirrer bar and capped with a rubber septum. The tube was evacuated and refilled with N_2 (this operation was repeated three times) and then toluene (0.2 mL) and D_2O (18.0 μL, 1.00 mmol, 1000 mol%) were added sequentially. The tube was sealed with a screw cap and heated to 130 °C for 24 hours with vigorous stirring. Upon completion, the solvent was removed under reduced pressure and the crude mixture was purified by FCC (hexane/EtOAc 60:40) to recover amide ***deuterio*-1f'**. Deuterium incorporation was calculated by integration of the ^{1}H NMR spectrum.

Supplementary Figure 4c: Substrate **1f** (17.8 mg, 0.10 mmol, 100 mol%), [Ir(cod)$_2$]BARF (6.36 mg, 5.00 μmol, 5 mol%) and *rac*-BINAP (3.11 mg, 5.00 μmol, 5 mol%) were placed in an oven-dried resealable tube equipped with a magnetic stirrer bar and capped with a rubber septum. The tube was evacuated and refilled with N_2 (this operation was repeated three times) and then styrene (23.0 μL, 0.20 mmol, 200 mol%), toluene (0.2 mL) and D_2O (18.0 μL, 1.00 mmol, 1000 mol%) were added sequentially. The tube was sealed with a screw cap and heated to 130 °C for 24 hours with vigorous stirring. Upon completion, the solvent was removed under reduced pressure and the crude mixture was purified by FCC (hexane/EtOAc 70:30) to recover amide ***deuterio*-1f'** and obtain product ***deuterio*-3fa'**. Deuterium incorporation was calculated by integration of the ^{1}H NMR spectrum.

Supplementary Figure 4d: (*2S,3S*)-*N,N*-Dimethyl-3-phenyl-2-(phenylamino)butanamide (28.2 mg, 0.10 mmol, 100 mol%), [Ir(cod)$_2$]BARF (6.36 mg, 5.00 μmol, 5 mol%) and *rac*-BINAP (3.11 mg, 5.00 μmol, 5 mol%) were placed in an oven-dried resealable tube equipped with a

magnetic stirrer bar and capped with a rubber septum. The tube was evacuated and refilled with N_2 (this operation was repeated three times) and then toluene (0.2 mL) and D_2O (18.0 µL, 1.00 mmol, 1000 mol%) were added sequentially. The tube was sealed with a screw cap and heated to 130 °C for 24 hours with vigorous stirring. Upon completion, the solvent was removed under reduced pressure and the crude mixture was purified by FCC (hexane/EtOAc 65:35) to recover amide ***deuterio*-3fa'**. Deuterium incorporation was calculated by integration of the ^{1}H NMR spectrum.

Supplementary Figure 4e: Substrate **1f** (17.8 mg, 0.10 mmol, 100 mol%), [Ir(cod)$_2$]BARF (6.36 mg, 5.00 µmol, 5 mol%), *rac*-BINAP (3.11 mg, 5.00 µmol, 5 mol%) and ***deuterio*-2j**[24] (36.4 mg, 0.20 mmol, 200 mol%) were placed in an oven-dried resealable tube equipped with a magnetic stirrer bar and capped with a rubber septum. The tube was evacuated and refilled with N_2 (this operation was repeated three times) and then toluene (0.2 mL) was added. The tube was sealed with a screw cap and heated to 130 °C for 24 hours with vigorous stirring. Upon completion, the solvent was removed under reduced pressure and the crude mixture was purified by FCC (hexane/EtOAc 70:30) to obtain two fractions.

- F_1 (Rf = 0.9) contained impure recovered ***deuterio*-2j'.**
- F_2 (Rf = 0.3) contained pure product ***deuterio*-3fj'** (26.5 mg, 74%).

F_1 was then purified a second time by FCC (using pentane/toluene 80:20) to afford pure recovered ***deuterio*-2j'** (17.1 mg).

Deuterium incorporation was calculated by integration of both ^{1}H and ^{2}D NMR spectra.

Use of C2 deuterated alkene ***deuterio*-2j** resulted in scrambling of the labels in both the product **3fj'** and recovered alkene ***deuterio*-2j'**. Additionally, deuterium exchange was observed at the -NMe_2 unit. This indicates that reversible oxidative addition of the C-H bond of the NMe_2 unit of **1f** or **3fj'** occurs. This would then allow scrambling of the deuterium labels via reversible hydrometallation of the alkene. An iridium-hydride species may also form by other pathways, as described in the experiment involving **1n**, shown below.

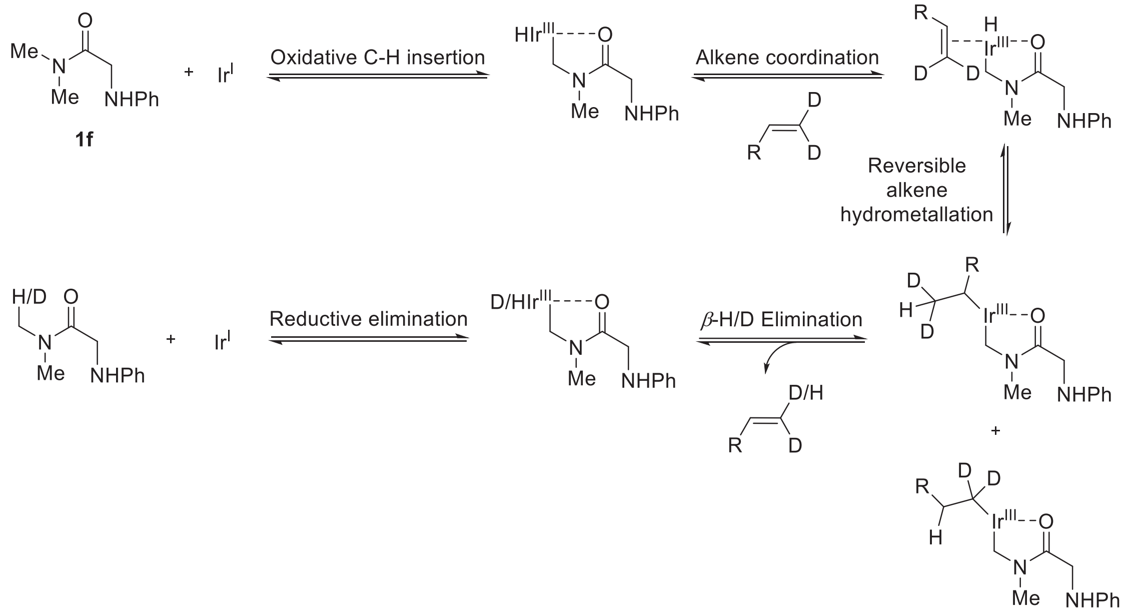

Supplementary Figure 4f: Substrate **1n** (14.9 mg, 0.10 mmol, 100 mol%), [Ir(cod)$_2$]BARF (6.36 mg, 5.00 µmol, 5 mol%), *rac*-BINAP (3.11 mg, 5.00 µmol, 5 mol%) and ***deuterio*-2j** (36.4 mg, 0.20 mmol, 200 mol%) were placed in an oven-dried resealable tube equipped with a magnetic stirrer bar and capped with a rubber septum. The tube was evacuated and refilled with N_2 (this operation was repeated three times) and then toluene (0.2 mL) was added. The tube was sealed with a screw cap and heated to 100 °C for 24 hours with vigorous stirring. Upon completion, the solvent was removed under reduced pressure and the crude mixture was purified by FCC (hexane/EtOAc 95:5) to obtain two fractions.

- F_1 (Rf = 0.8) contained impure recovered ***deuterio*-2j'**.
- F_2 (Rf = 0.2) contained pure product ***deuterio*-3na'** (24.7 mg, 75%).

F_1 was then purified a second time by FCC (using pentane/toluene 80:20) to afford pure recovered ***deuterio*-2j'** (20.8 mg).

Deuterium incorporation was calculated by integration of both ^{1}H and ^{2}D NMR spectra.

This result indicates that an iridium hydride species is also formed during the reaction of **1n**. The precise nature of this species, and the pathway for its formation cannot be advanced based on available data; however, in the proposed alkylation mechanism, proton release occurs from the NHPh directing group during metallation. Protonation of an Ir(I) species would provide a Ir(III)-hydride, which could mediate reversible hyrometallation of the alkene.

Supplementary Figure 4. Deuterium exchange and labelling experiments.

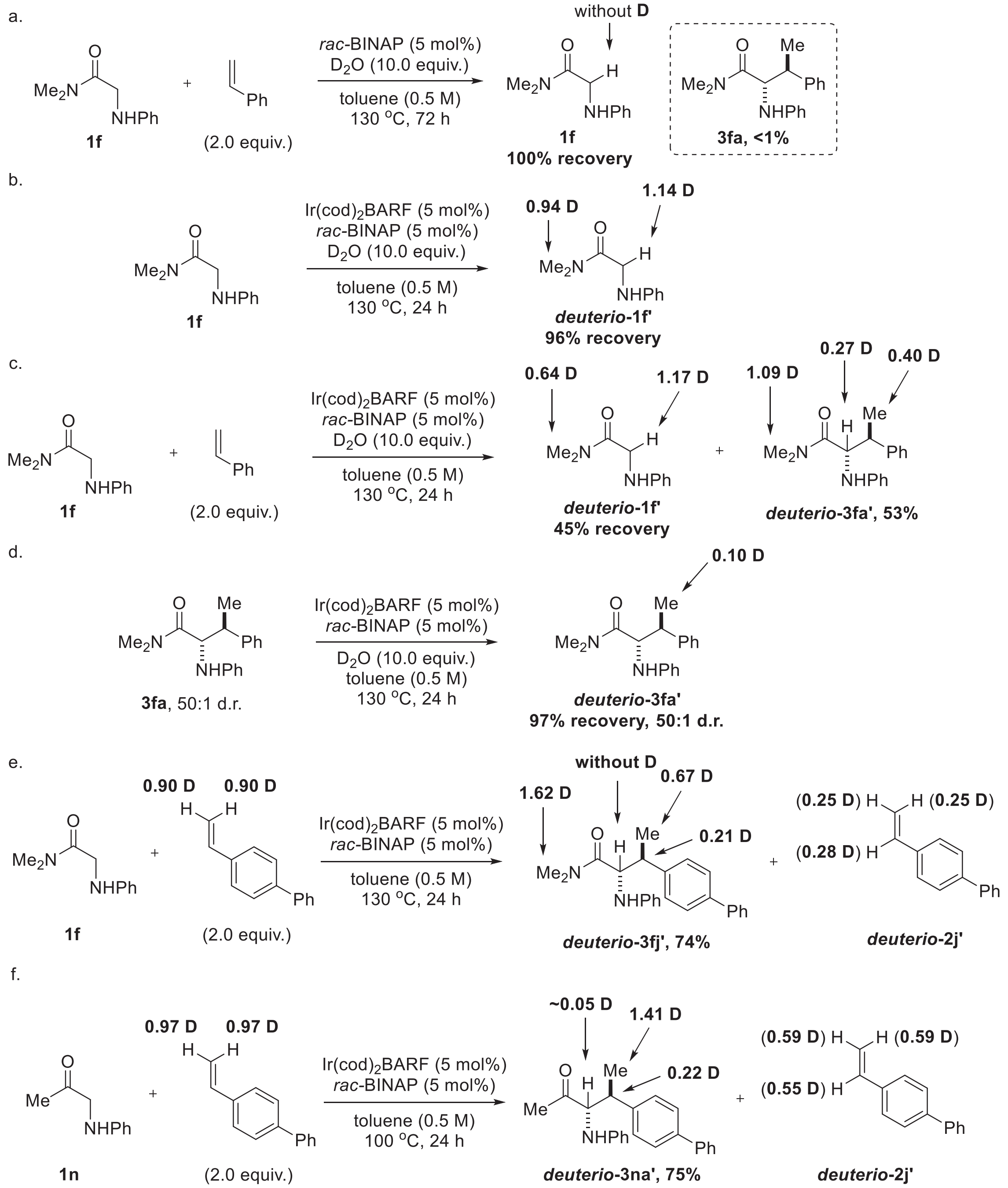

^{13}C-KIE determination experiment

Procedure (Singleton method):

An oven-dried resealable tube fitted with a magnetic stirrer bar was charged with amide **1f** (507 mg, 2.85 mmol, 143 mol%), styrene **2j** (361 mg, 2.00 mmol, 100 mol%), [Ir(cod)$_2$]BARF (127 mg, 0.10 mmol, 5 mol%) and *rac*-BINAP (62.3 mg, 0.10 mmol, 5 mol%). The tube was

evacuated and refilled with N_2 (this operation was repeated three times) and then toluene (4.0 mL) was added. The tube was sealed with a screw cap and placed into a preheated heating block at 130 °C and stirred for 11 hours. The reaction mixture was cooled to r.t. and concentrated *in vacuo*. The crude mixture was transferred into a 10.0 mL volumetric flask, which was previously charged with an internal standard (1,3,5-trimethoxybenzene, 84.1 mg) and the flask was filled with $CDCl_3$ (pre-treated by filtration through anhydrous K_2CO_3). 0.2 mL aliquots were taken from the solution and transferred into four NMR tubes. An additional 0.4 mL of base washed $CDCl_3$ was added to each sample. A ^{1}H NMR spectrum was recorded for each sample employing a 500 MHz instrument, using the following parameters: 16 scans, π/2 pulse, 6.5 s acquisition time and 40 s relaxation delay. The conversion of the alkene starting material (F) was determined by the integration of the C_1-H_{trans} signal of 4-vinylbiphenyl against the aromatic C-H signal of the internal standard.

- Conversion: 64.3%, 64.3%, 64.3%, 64.3%

The crude material was then purified by FCC (using pentane/toluene 80:20) to afford styrene **2j** contaminated by approximatively 5% of the reduced compound (4-ethylbiphenyl). The desired product **3fj** was iaolated in 60.4% yield. Two successive purifications by FCC on alumina (using pentane/toluene 80:20) afforded pure styrene **2j**.

Quantitative ^{13}C NMR analysis:

All NMR samples were prepared using 60 mg of styrene **2j** (starting material or recovered starting material) in 0.6 mL of basified $CDCl_3$. The ^{13}C NMR spectra were recorded at 126 MHz using inverse gated decoupling and employing a 500 MHz instrument equipped with a CryoProbeTM. The spectra were recorded according to the following parameters: 1024 scans, π/6 pulse, 15 s relaxation delay. The common compromise of a shorter (π/6) excitation pulse (vs π/2 in refeernce 26) was used to allow shorter relaxation delays – this effectively sacrifices signal-to-noise for more efficient data collection time. Because of the instrumentation, the latter was not limiting in terms of integration accuracy. A 15 second relaxation delay was found to be a conservative compromise that gave very reliable integrations under these excitation conditions that were in line with experiments run using longer (and shorter) relaxation delays. A total of three spectra were recorded for each sample. The resulting six FIDs (3 for SM + 3 for the recovered SM) were processed at the same time applying the same phase correction - a fifteenth order polynomial fit baseline correction and 256K zero filling. Integrations were numerically determined using a constant region for each peak corresponding to eight times of peak width at half height (± 5w1/2). The peak

belonging to C_6 of styrene **2j** was chosen as the internal standard and was set with an integration of 1000.

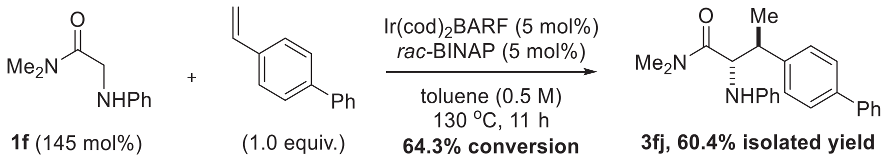

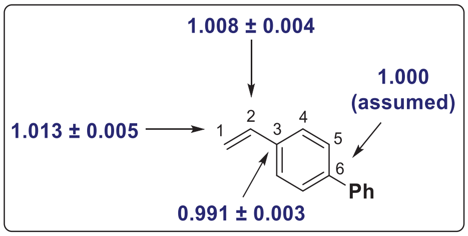

<u>Formulas applied for the determination of ^{13}C KIEs:</u>

The formulas employed in the calculations for the determination of the KIE were reported by Saunders[25] and Singleton[26] and are summarised as follows:

F = conversion of starting material.

R/R₀ = proportion of the minor isotopic component in recovered material compared to the original starting material.

Δ(R/R₀) $= R/R_0((\Delta R/R)^2 + (\Delta R_0/R_0)^2)^{1/2}$

$$\textbf{KIE} = \frac{\ln(1-F)}{\ln[(1-F)R/R_0}$$

$$\Delta\textbf{KIE}_F = \frac{\partial KIE}{\partial F}\,\Delta F = \frac{-ln(R/R_0)}{(1-F)ln^2[(1-F)R/R_0]}\,\Delta F$$

$$\Delta\textbf{KIE}_R = \frac{\partial KIE}{\partial (R/R_0)}\,\Delta(R/R_0) = \frac{-ln(1-F)}{(R/R_0)ln^2[(1-F)R/R_0]}\,\Delta R/R_0$$

ΔKIE $= KIE*((\Delta KIE_R/KIE)^2 + (\Delta KIE_F/KIE)^2)^{1/2}$

Tables used for the determination of the ^{13}C-KIE values are reported on the next pages.

Conversion (F)					
**fid1**	**fid2**	**fid3**	**fid4**	**F**	**ΔF**
64.3	64.3	64.3	64.3	64.3	**0.0**

^{13}C-NMR integration of alkene starting material (R_0)					
**ppm peaks**	**fid1**	**fid2**	**fid3**	**R_0**	**ΔR_0**
**113.9 (C1)**	918.2	909.9	911.8	**913.3**	**4.3**
**136.4 (C2)**	1076.2	1070.0	1073.7	**1073.3**	**3.1**
**136.6 (C3)**	1014.2	1008.2	1008.1	**1010.2**	**3.5**
**126.6 (C4)**	2039.7	2035.3	2037.0	**2037.3**	**2.2**
**140.7 (C6)**	1000.0	1000.0	1000.0	**1000.0**	**0.0**

^{13}C-NMR integration of alkene from 64.3% conversion reaction (R)					
**ppm peaks**	**fid1**	**fid2**	**fid3**	**R**	**ΔR**
**113.9 (C1)**	923.3	926.7	926.8	**925.6**	**2.0**
**136.4 (C2)**	1084.9	1082.3	1079.9	**1082.4**	**2.5**
**136.6 (C3)**	1001.2	999.6	1000.0	**1000.3**	**0.8**
**126.6 (C4)**	2029.5	2033.6	2033.4	**2032.2**	**2.3**
**140.7 (C6)**	1000.0	1000.0	1000.0	**1000.0**	**0.0**

Determination of ^{13}C KIEs						
**ppm peaks**	**R/R_0**	**$\Delta(R/R_0)$**	**ΔKIE_F**	**ΔKIE_R**	**KIE**	**ΔKIE**
**113.9 (C1)**	1.013468	0.005296	0.000000	0.005207	1.013159	0.005207
**136.4 (C2)**	1.008447	0.003744	0.000000	0.003664	1.008234	0.003664
**136.6 (C3)**	0.990200	0.003522	0.000000	0.003388	0.990529	0.003388
**126.6 (C4)**	0.997464	0.001571	0.000000	0.001521	0.997541	0.001521
**140.7 (C6)**	1	0	0	0	1	0

Output

**C**	**KIE**
C1	**1.013 ± 0.005**
C2	**1.008 ± 0.004**
C3	**0.991 ± 0.003**
C4	**0.998 ± 0.002**

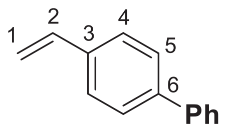

Supplementary Figure 5: Kinetic experiments

Supplementary Figure 5a (using BINAP as ligand): Two oven-dried resealable tubes were charged with amide **1f** (89.1 mg, 0.50 mmol, 100 mol%), $[Ir(cod)_2]BARF$ (19.1 mg, 15.0 μmol, 3 mol% and 31.8 mg, 25.0 μmol, 5 mol%) and *rac*-BINAP (9.34 mg, 15.0 μmol, 3 mol% and 15.6 mg, 25.0 μmol, 5 mol%), respectively. These tubes were evacuated and refilled with N_2 (this operation was repeated three times) and then styrene (115 μL, 1.00 mmol, 200 mol%) and toluene (1.0 mL) were added, respectively. A portion of each stock solution was transferred to five Schlenk tubes (220 μL in each) under nitrogen. These tubes were sealed with a screw cap, placed into a preheated heating block at 130 °C and stirred for the indicated time. The reaction mixture was cooled to r.t. and then an internal standard (1,3,5-trimethoxybenzene, 16.8 mg) was added. The mixture was concentrated *in vacuo*. The concentration of substrate **1f** was calculated by integration of the ^1H NMR spectrum.

Method: The order in catalyst has been determined applying the Variable Time Normalization Graphical Analysis (VTNGA).[27] Different concentrations of catalyst (3 mol% and 5 mol%, respectively) were used and the concentration of substrate **1f** was plotted against $t[Ir]^n$ where n is the partial order in iridium catalyst, and t is the reaction time. The order in iridium catalyst is that value of n that causes the curves to overlay. The overlap between the temporal reaction profiles with catalyst loadings of 3 mol% and 5 mol% indicates that the order in iridium catalyst is 2.1 using *rac*-BINAP as a ligand.

Supplementary Figure 5b (using SEGPHOS as ligand): Two oven-dried resealable tubes were charged with amide **1f** (107 mg, 0.60 mmol, 100 mol%), $[Ir(cod)_2]BARF$ (22.9 mg, 18.0 μmol, 3 mol% and 38.2 mg, 30.0 μmol, 5 mol%) and *R*-SEGPHOS (11.0 mg, 18.0 μmol, 3 mol% and 18.3 mg, 30.0 μmol, 5 mol%), respectively. These tubes were evacuated and refilled with N_2 (this operation was repeated three times) and then styrene (138 μL, 1.20 mmol, 200 mol%) and 1,4-dioxane (1.2 mL) were added, respectively. A portion of each stock solution was transferred to six Schlenk tubes (240 μL in each) under nitrogen. These tubes were sealed with a screw cap, placed into a preheated heating block at 130 °C and stirred for the indicated time. The reaction mixture was cooled to r.t. and then an internal standard (1,3,5-trimethoxybenzene, 16.8 mg) was added. The mixture was concentrated *in vacuo*. The concentration of substrate **1f** was calculated by integration of the ^1H NMR spectrum.

Method: The order in catalyst has been determined applying the Variable Time Normalization Graphical Analysis (VTNGA).[27] Different concentrations of catalyst (3 mol% and 5 mol%, respectively) were used and the concentration of substrate **1f** was plotted against $t[Ir]^n$ where n

is the partial order in iridium catalyst, and t is the reaction time. The order in iridium catalyst is that value of n that causes the curves to overlay. The overlap between the temporal reaction profiles with catalyst loadings of 3 mol% and 5 mol% indicates that the order in iridium catalyst is 1.95 using *R*-SEGPHOS as a ligand.

Supplementary Figure 5a. Kinetic experiment using BINAP as ligand (2 runs).

Run 1

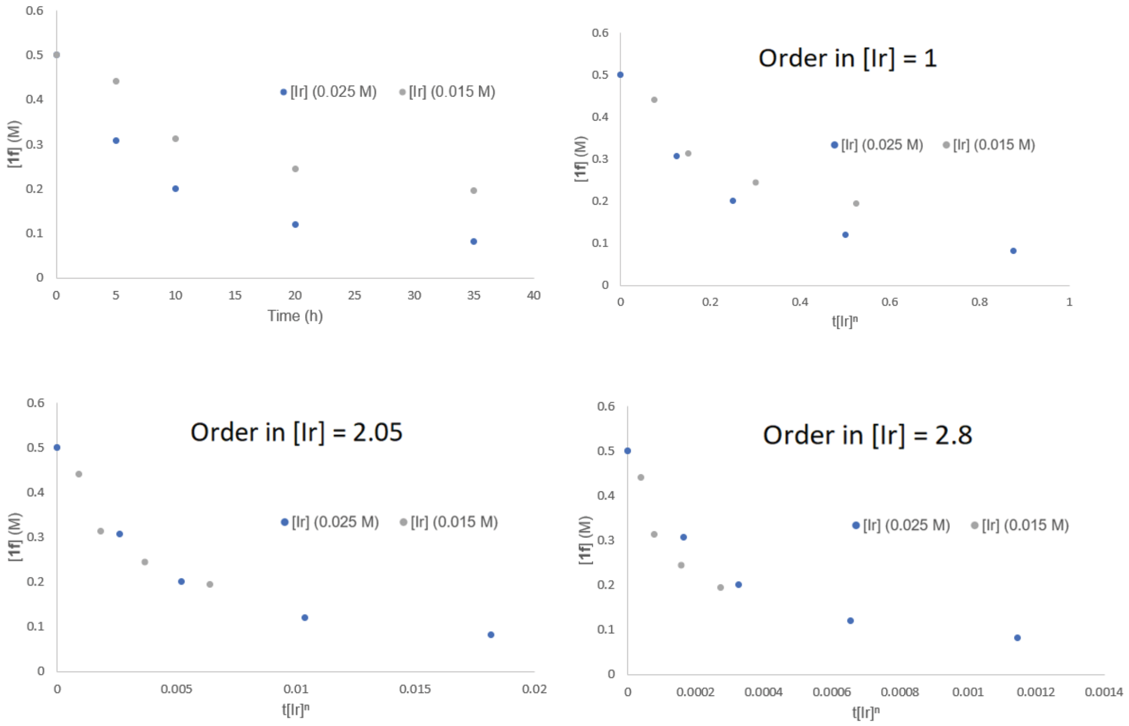

Run 2

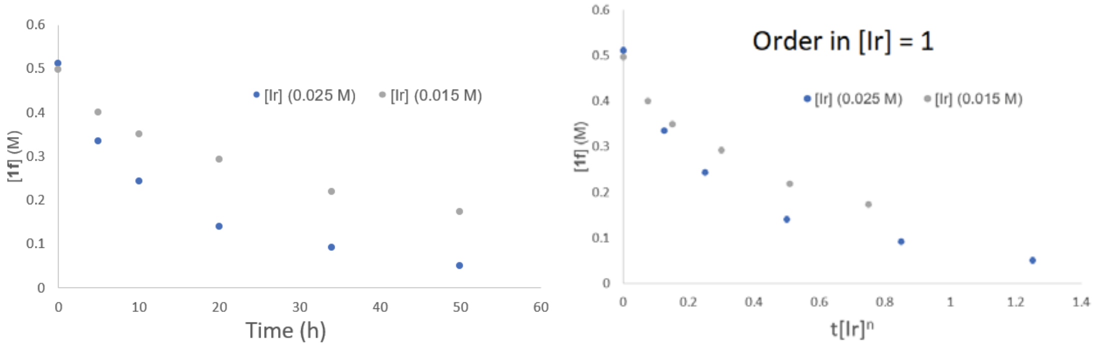

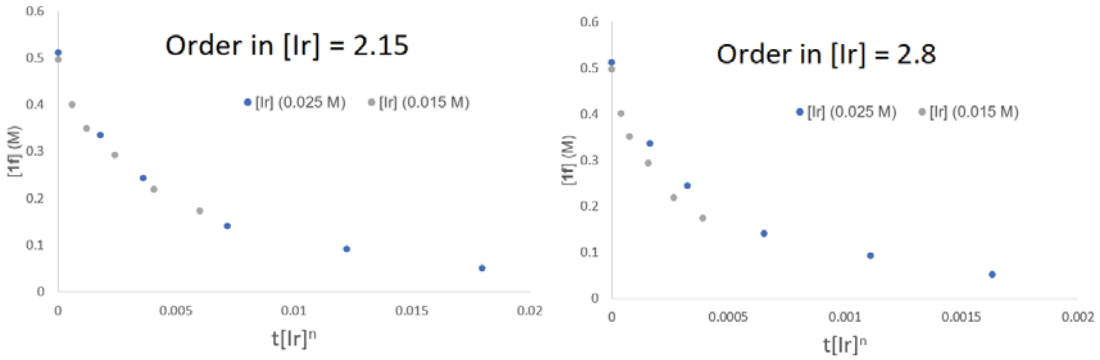

Supplementary Figure 5b. Kinetic experiment using SEGPHOS as ligand (1 run).

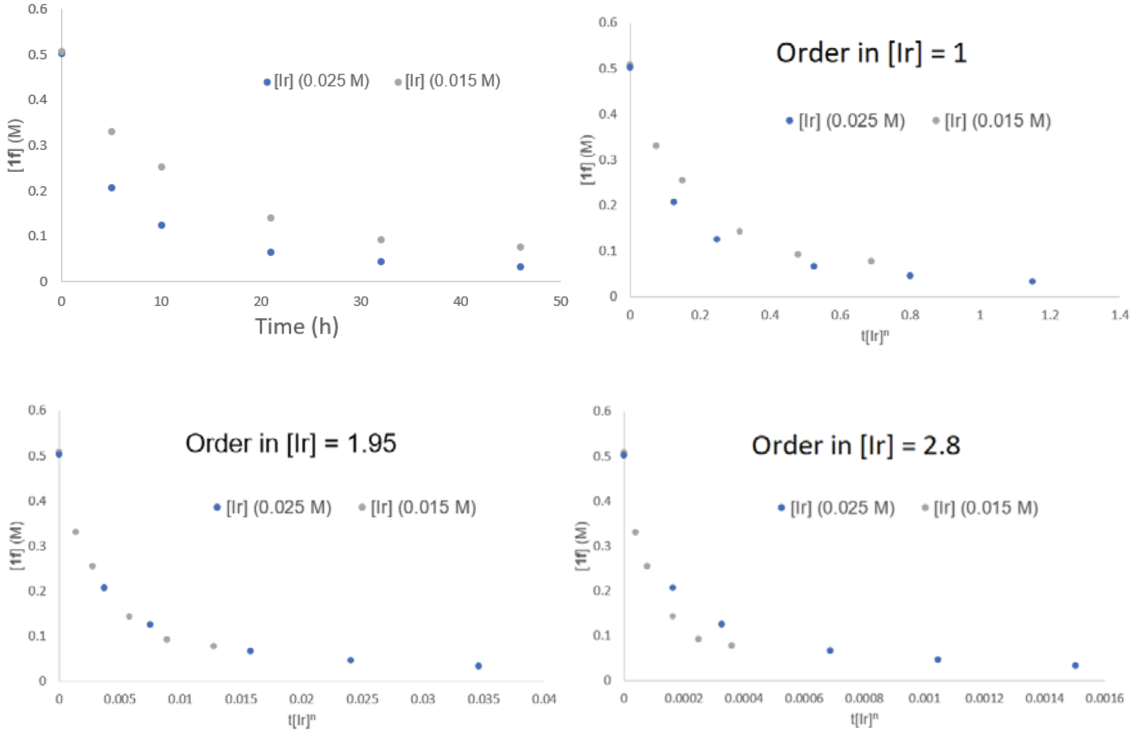

X-Ray crystallography data

The structure of compound **3fa** was determined by single crystal X-ray diffraction of crystals grown from EtOAc/hexane. See CCDC 2246104.

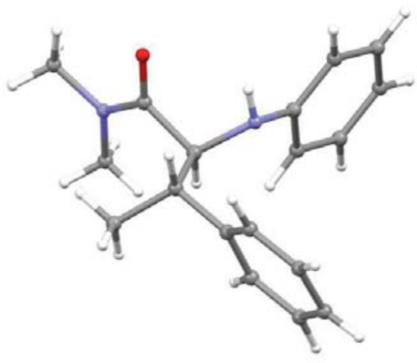

The structure of compound **10** was determined by single crystal X-ray diffraction of crystals grown from EtOAc/hexane. See CCDC 2245009.

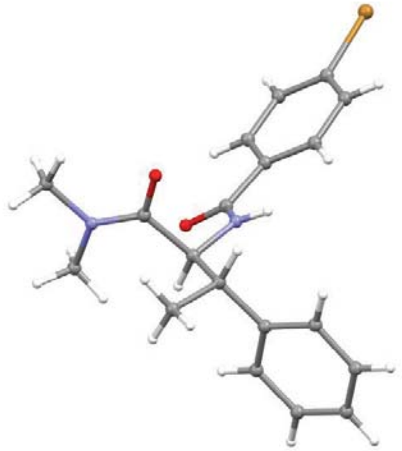

References

1. Tsuchikama, K., Kasagawa, M., Endo, K., Shibata, T. Cationic Ir(I)-Catalyzed sp^3 C−H Bond Alkenylation of Amides with Alkynes. *Org. Lett.* **11**, 1821−1823 (2009).
2. Dervisi, A., Carcedo, C., Ooi, L.-L. Chiral Diphosphine Ddppm-Iridium Complexes: Effective Asymmetric Imine Hydrogenations at Ambient Pressures. *Adv. Synth. Catal.* **348**, 175−183 (2006).
3. Coste, A., Karthikeyan, G., Couty, F., Evano, G. Copper-Mediated Coupling of 1,1-Dibromo-1-alkenes with Nitrogen Nucleophiles: A General Method for the Synthesis of Ynamides. *Angew. Chem. Int. Ed.* **48**, 4381−4385 (2009).
4. Lee, K., Silverio, D. L., Torker, S., Robbins, D. W., Haeffner, F., van der Mei, F. W., Hoveyda, A. H. Catalytic Enantioselective Addition of Organoboron Reagents to Fluoroketones Controlled by Electrostatic Interactions. *Nat. Chem.* **8**, 768−777 (2016).
5. Crapster, J. A., Guzei, I. A., Blackwell, H. E. A Peptoid Ribbon Secondary Structure. *Angew. Chem. Int. Ed.* **52**, 5079−5084 (2013).
6. Tian, H., Xu, W., Liu, Y., Wang, Q. Unnatural α-Amino Acid Synthesized through α-Alkylation of Glycine Derivatives by Diacyl Peroxides. *Org. Lett.* **22**, 5005−5008 (2020).
7. Cai, C., Chen, T.-T., Copper Triflate Catalyzed Oxidative α-Allylation of Glycine Derivatives. *Synlett* **28**, 1368−1372 (2017).
8. Wang, Z. J., Peck, N. E., Renata, H., Arnold, F. H. Cytochrome P450-Catalyzed Insertion of Carbenoids into N−H Bonds. *Chem. Sci.* **5**, 598−601 (2014).
9. Iso, Y., Shindo, H., Hamana, H. Efficient Synthesis of Resin-Bond α-TMSdiazoketones and Their use in Solid-Phase Organic Synthesis. *Tetrahedron*, **56**, 5353−5361 (2000).
10. Xu, Q., Li, B., Ma, Y., Sun, F., Gao Y., Ye, N. $K_2S_2O_8$-HFIP Synergistically Promoted *para*-Selective sp^3 C−H Bond Diarylation of Glycine Esters. *Org. Biomol. Chem.* **18**, 666−670 (2020).
11. Chen, C., Zhu, M., Jiang, L., Zeng, Z., Yi, N., Xiang, J. Copper-Catalyzed Oxidative Cross-Coupling of α-Aminocarbonyl Compounds with Primary Amines toward 2-oxo-Acetamidines. *Org. Biomol. Chem.* **15**, 8134−8139 (2017).

12. Dai, Z., Tian, Q., Li, Y., Shang, S., Luo, W., Wang, X., Li, D., Zhang, Y., Li, Z., Yuan, J. Michael Addition Reaction Catalyzed by Imidazolium Chloride to Protect Amino Groups and Construct Medium Ring Hetereocycles. *Molecules* **24**, 4224−4241 (2019).

13. Chen, Z., Yan, Q., Liu, Z., Xu, Y., Zhang, Y. Copper-Mediated Synthesis of 1,2,3-Triazoles from *N*-Tosylhydrazones and Anilines. *Angew. Chem. Int. Ed.* **52**, 13324−13328 (2013).

14. Hattori, G., Sakata, K., Matsuzawa, H., Tanabe, Y., Miyake, Y., Nishibayashi, Y. Copper-Catalyzed Enantioselective Propargylic Amination of Propargylic Esters with Amines: Copper-Allenylidene Complexes as Key Intermediates. *J. Am. Chem. Soc.* **132**, 10592−10608 (2010).

15. Wang, L., Qin, R.-Q., Yan, H.-Y., Ding, M.-W. New Efficient Synthesis of 1,4-Benzodiazepin-5-ones by Catalytic Aza-Wittig Reaction. *Synthesis* **47**, 3522−3528 (2015).

16. Yu, Z., Liu, Q., Li, Q., Huang, Z., Yang, Y., You, J. Remote Editing of Stacked Aromatic Assemblies for Heteroannular C−H Functionalization by a Palladium Switch between Aromatic Rings. *Angew. Chem. Int. Ed.* 10.1002/anie. e202212079 (2022).

17. Yan, X.-B., Li, L., Wu, W.-Q., Xu, L., Li, Ke., Liu, Y.-C., Shi, H. Ni-Catalyzed Hydroalkylation of Olifins with *N*-Sulfonyl Amines. *Nat. Commun.* **12**, 5881 **(2021)**.

18. Zhou, Y., Bandar, J. S., Buchwald, S. L. Enantioselective CuH-Catalyzed Hydroacylation Employing Unsaturated Carboxylic Acids as Aldehyde Surrogates. *J. Am. Chem. Soc.* **139**, 8126−8129 (2017).

19. Myers, A. G., Lanman, B. A. A Solid-Supported, Enantioselective Synthesis Suitable for the Rapid Preparation of Large Numbers of Diverse Structural Analogues of (-)-Saframycin A. *J. Am. Chem. Soc.* **124**, 12969−12971 (2002).

20. Hong, F.-L., Chen, Y.-B., Ye, S.-H., Zhu, G.-Y., Zhu, X.-Q., Lu, X., Liu, R.-S., Ye, L.-W. Copper-Catalyzed Asymmetric Reaction of Alkenyl Diynes with Styrenes by Formal [3+2] Cycloaddition via Cu-Containing All-Carbon 1,3-Dipoles: Access to Chiral Pyrrole-Fused Bridged [2.2.1] Skeletons. *J. Am. Chem. Soc.* **142**, 7618−7626 (2020).

21. Hoffmann, S., Nicoletti, M., List, B. Catalytic Asymmetric Reductive Amination of Aldehydes via Dynamic Kinetic Resolution. *J. Am. Chem. Soc.* **128**, 13074−13075 (2006).

22. Hu, L., Wang, Y.-Z., Xu, L., Yin, Q., Zhang, X. Highly Enantioselective Synthesis of *N*-Unprotected Unnatural α-Amino Acid Derivatives by Ruthenium-Catalyzed Direct Asymmetric Reductive Amination. *Angew. Chem. Int. Ed.* 10.1002/anie.202202552 (2022).

23. Cabré, A., Verdaguer, X., Riera, A. Enantioselective Synthesis of β-Methyl Amines via Iridium-Catalyzed Asymmetric Hydrogenation of *N*-Sulfonyl Allyl Amines. *Adv. Synth. Catal.* **361**, 4196−4200 (2019).

24. Zhao, Z., Racicot, L., Murphy, G. K. Fluorinative Rearrangements of Substituted Phenylallenes Mediated by (Difluoroiodo)toluene: Synthesis of α-(Difluoromethyl)styrenes. *Angew. Chem. Int. Ed.* **56**, 11620−11623 (2017).

25. Saunders, W. H., Melander, L. In Reaction Rates of Isotopic Molecules, Wiley, New York (1981).

26. Singleton, D. A., Thomas, A. A. High-Precision Simultaneous Determination of Multiple Small Kinetic Isotope Effects at Natural Abundance *J. Am. Chem. Soc.* **117**, 9357−9358 (1995).

27. Burés, J. A Simple Graphical Method to Determine the Order in Catalyst. *Angew. Chem. Int. Ed.* **55**, 2028−2031 (2016).

NMR Spectra

N,*N*-Dimethyl-2-((4-methylphenyl)sulfonamido)acetamide (1b)

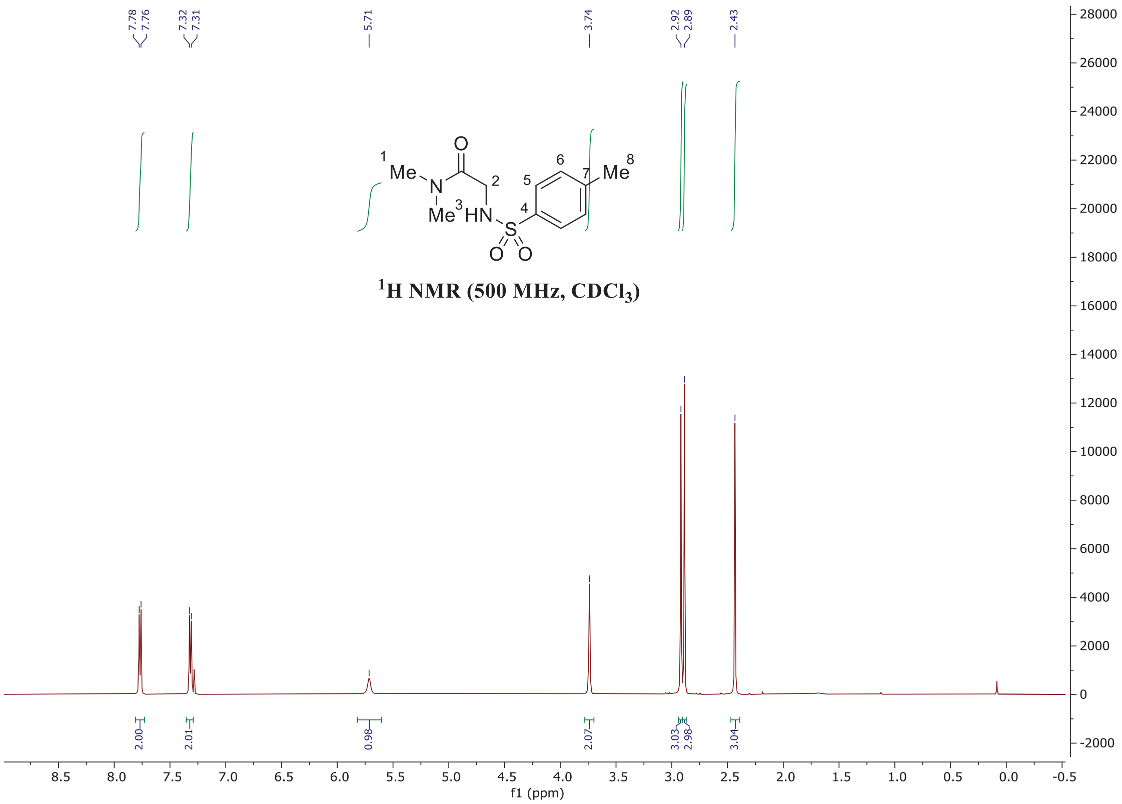

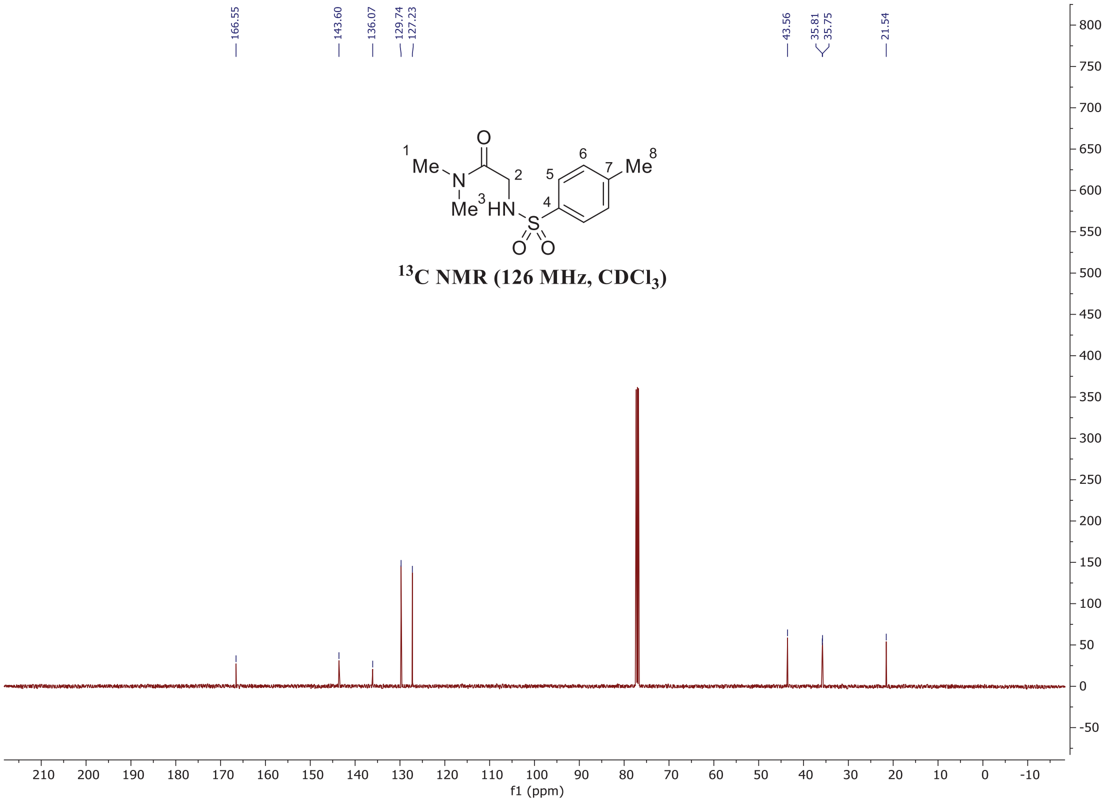

2-(Benzylamino)-*N*,*N*-dimethylacetamide (1d)

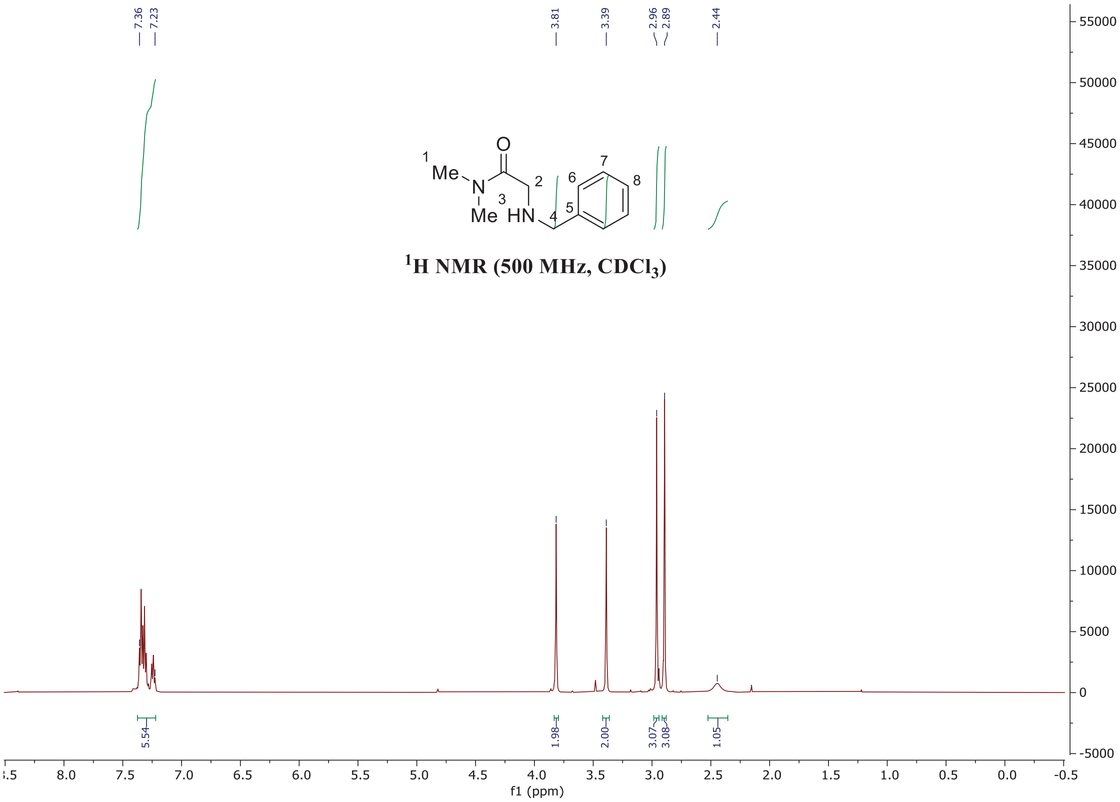

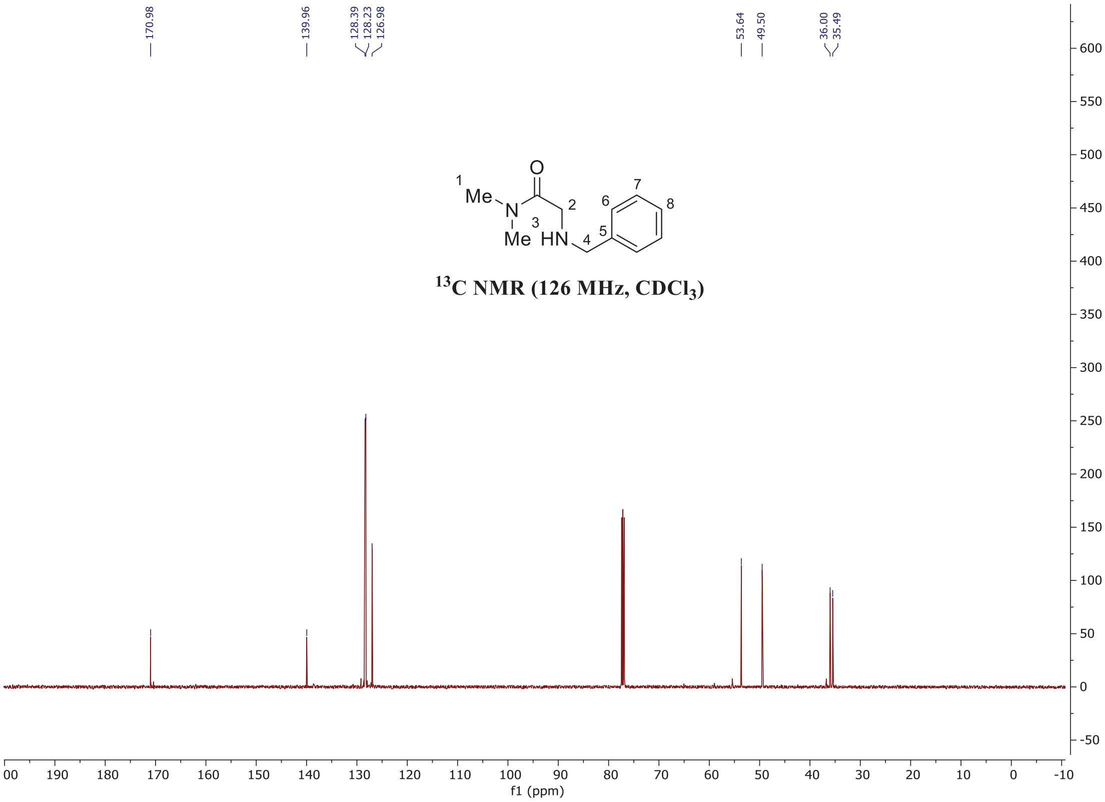

N,N-Dimethyl-2-(phenylamino)acetamide (1f)

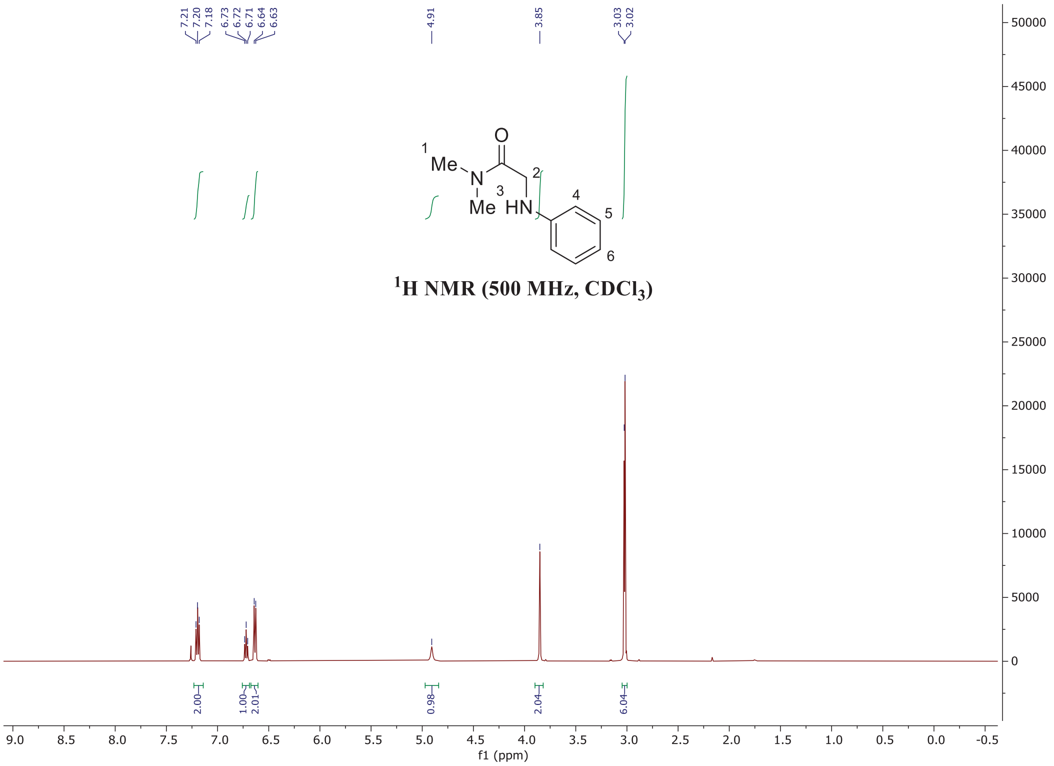

N-Methyl-2-(phenylamino)acetamide (1g)

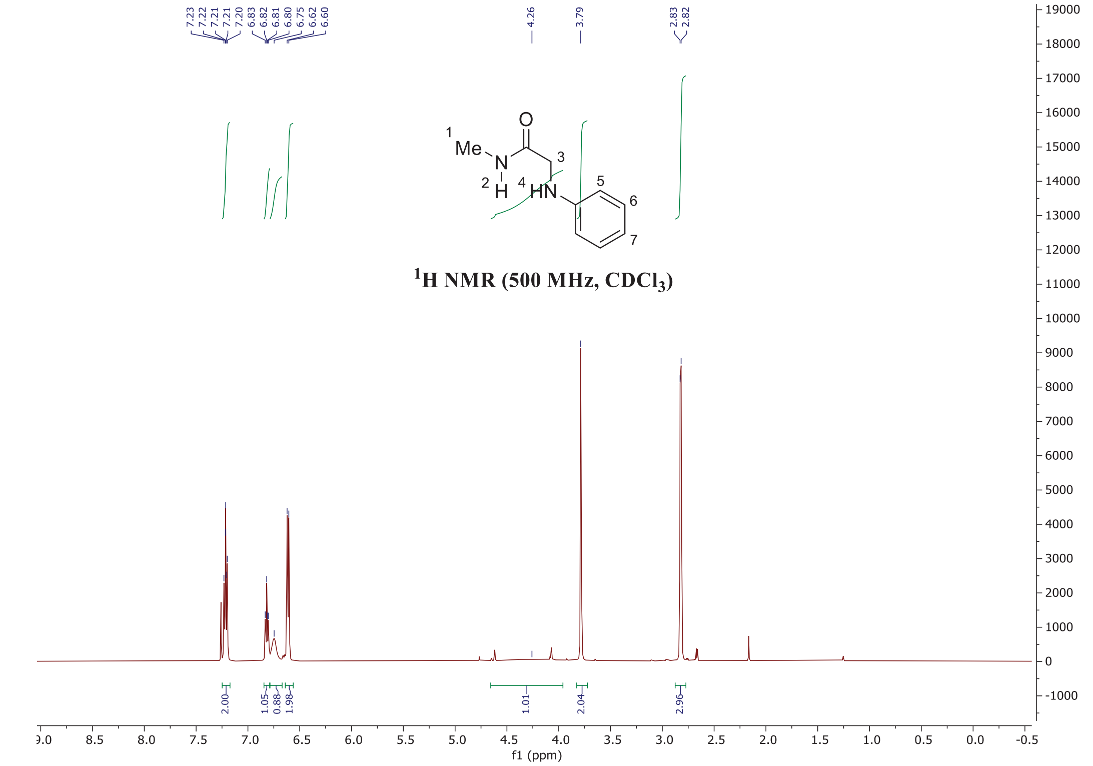

N,*N*-Diethyl-2-(phenylamino)acetamide (1h)

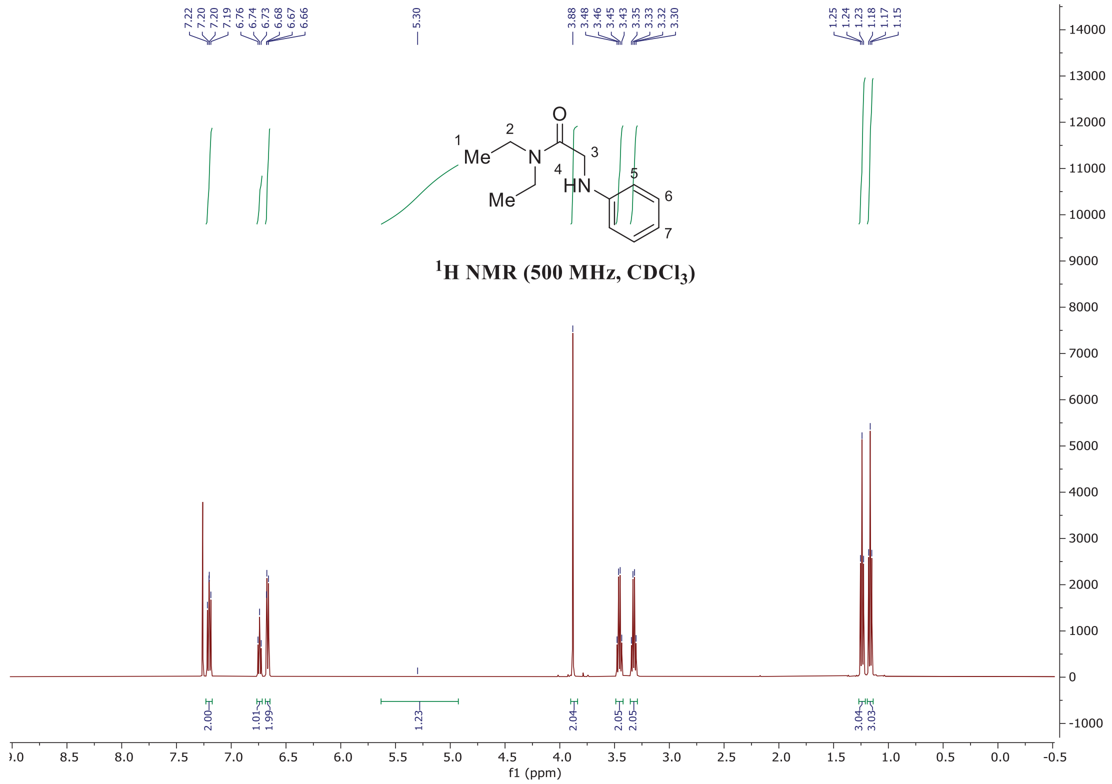

2-(Phenylamino)-1-(pyrrolidin-1-yl)ethan-1-one (1i)

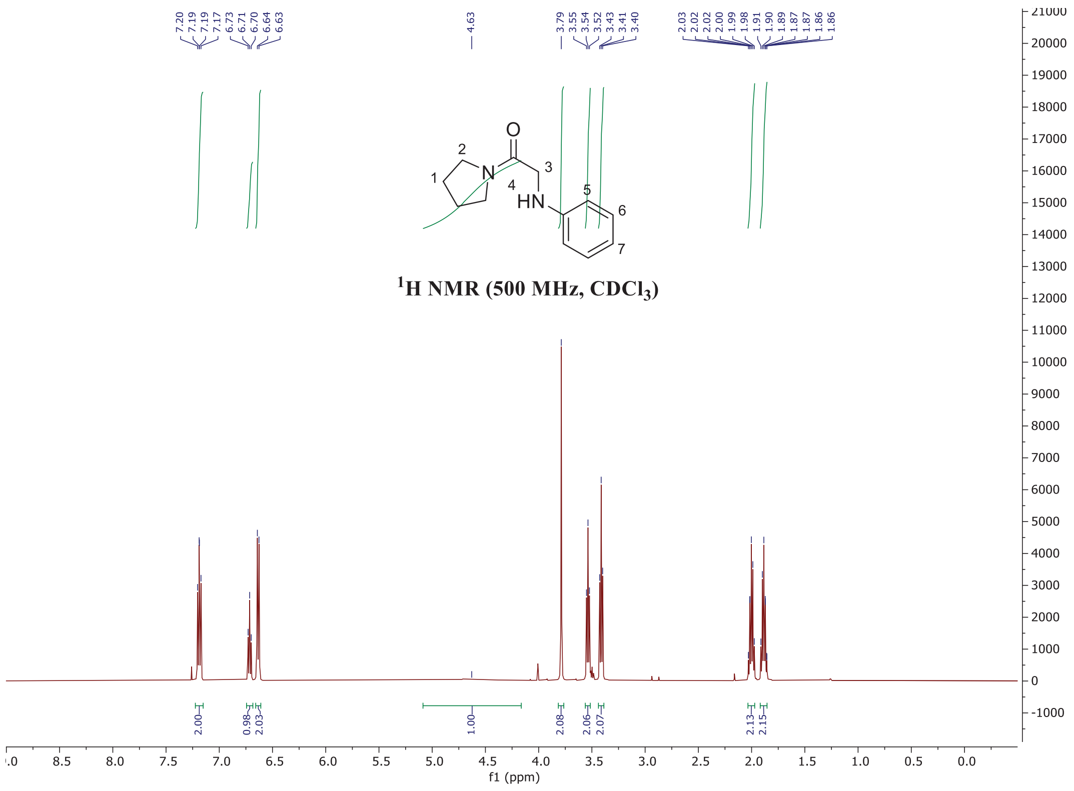

2-(Phenylamino)-1-(piperidin-1-yl)ethan-1-one (1j)

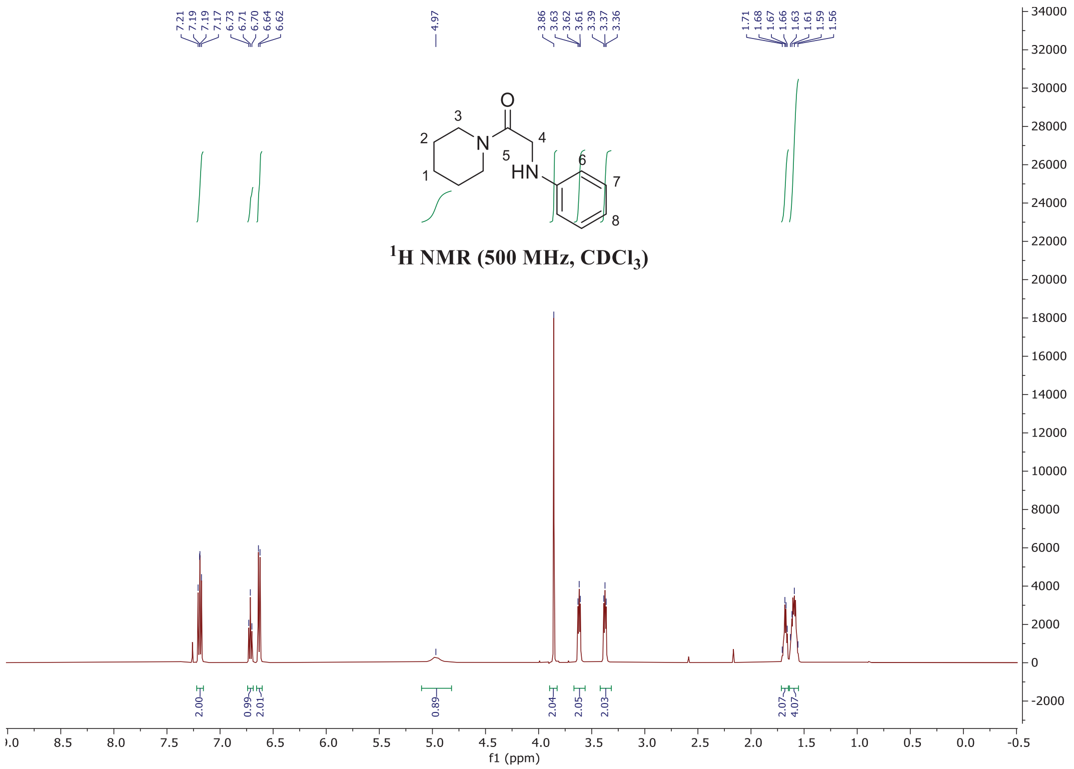

1-Morpholino-2-(phenylamino)ethan-1-one (1k)

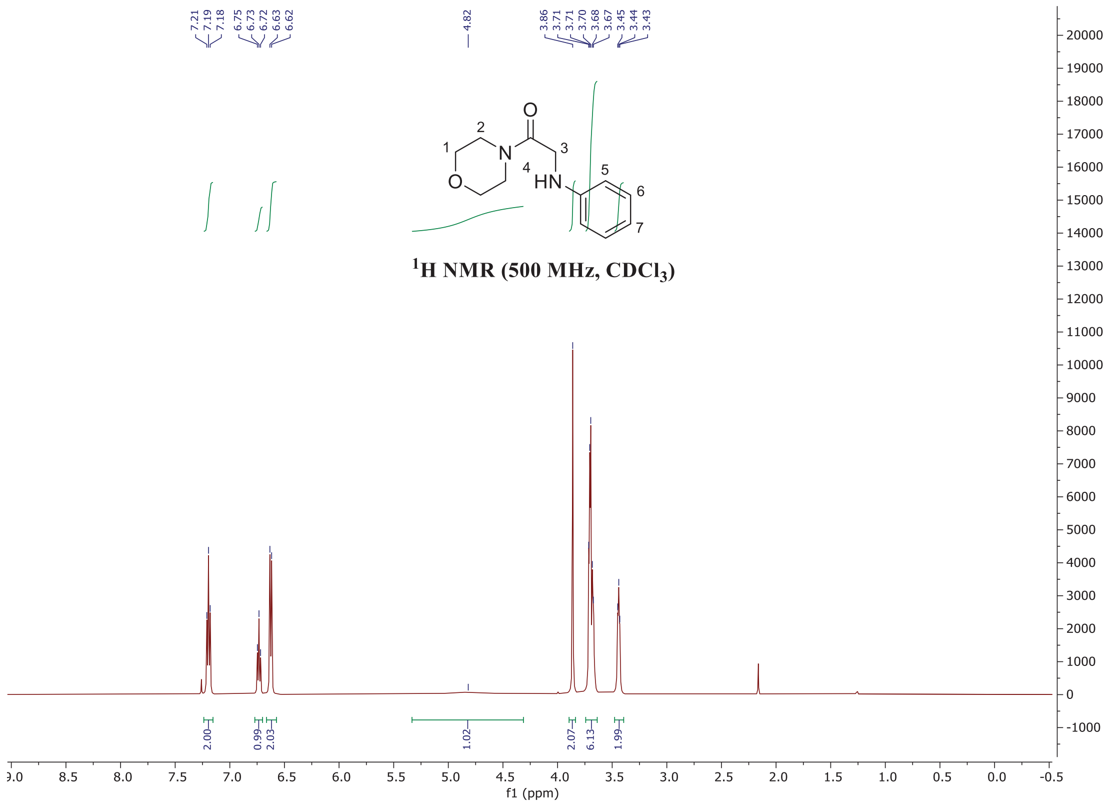

2-((4-Hydroxyphenyl)amino)-*N*-phenylacetamide (1l)

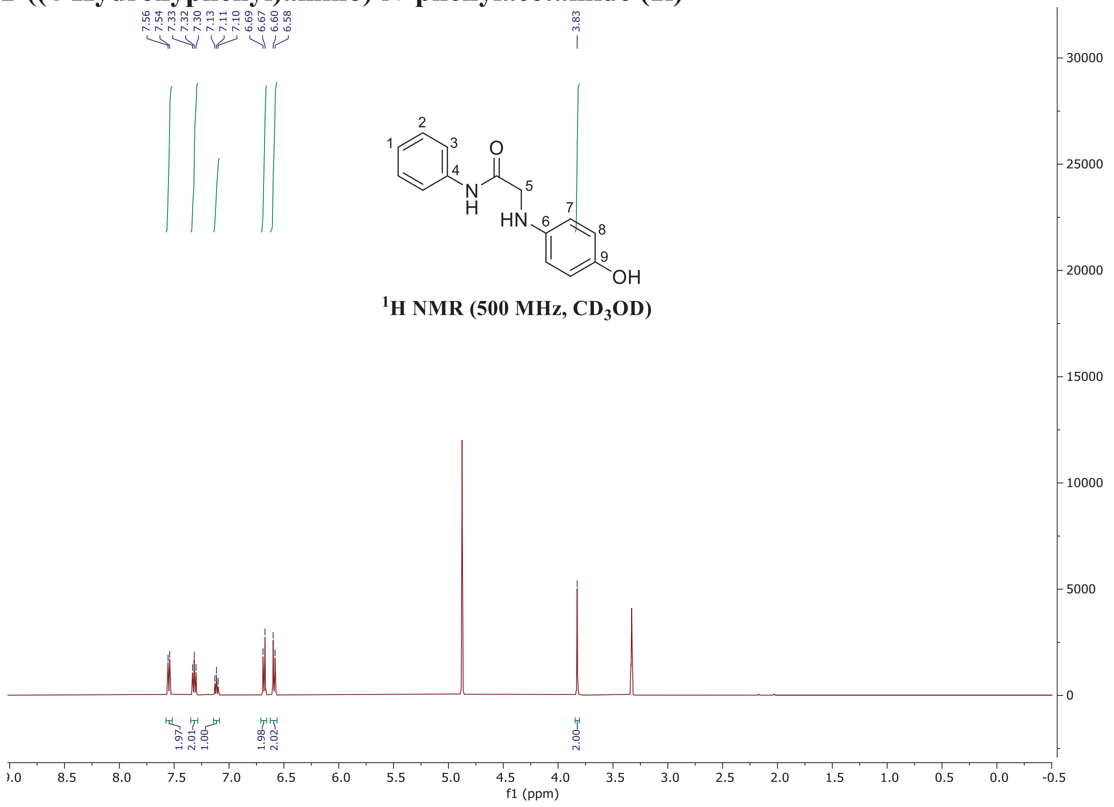

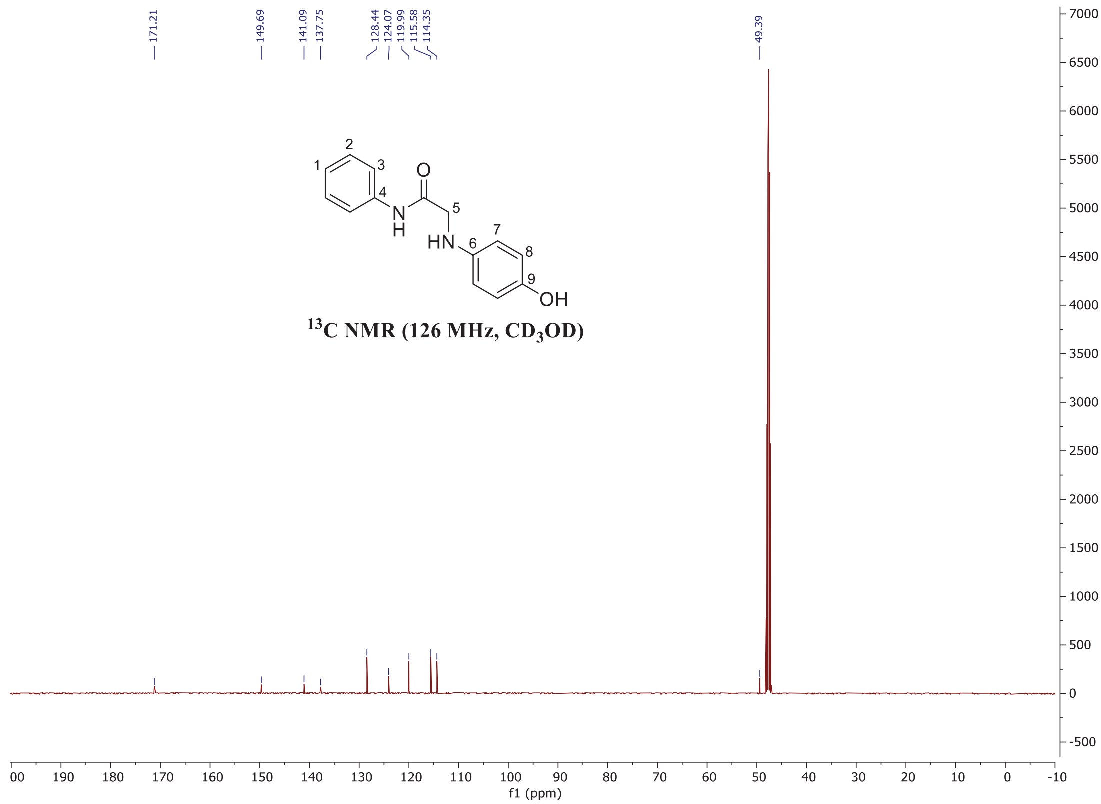

1-Phenyl-2-(phenylamino)ethan-1-one (1m)

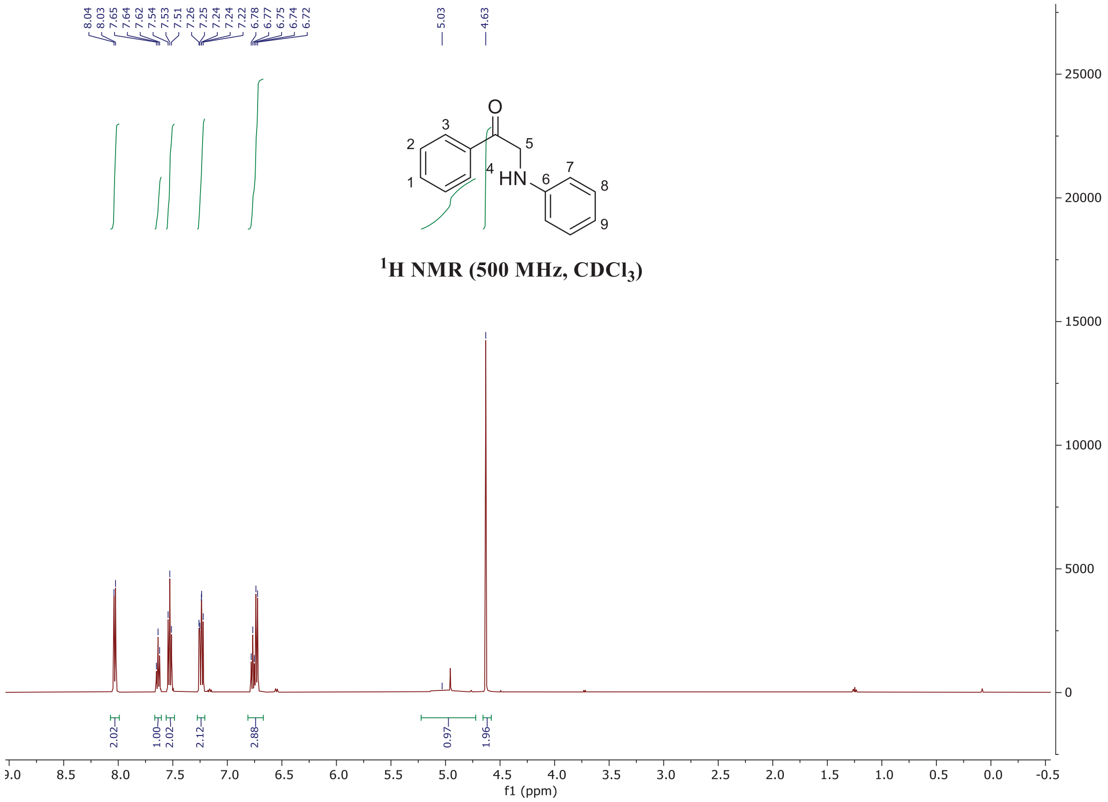

1-(Phenylamino)propan-2-one (1n)

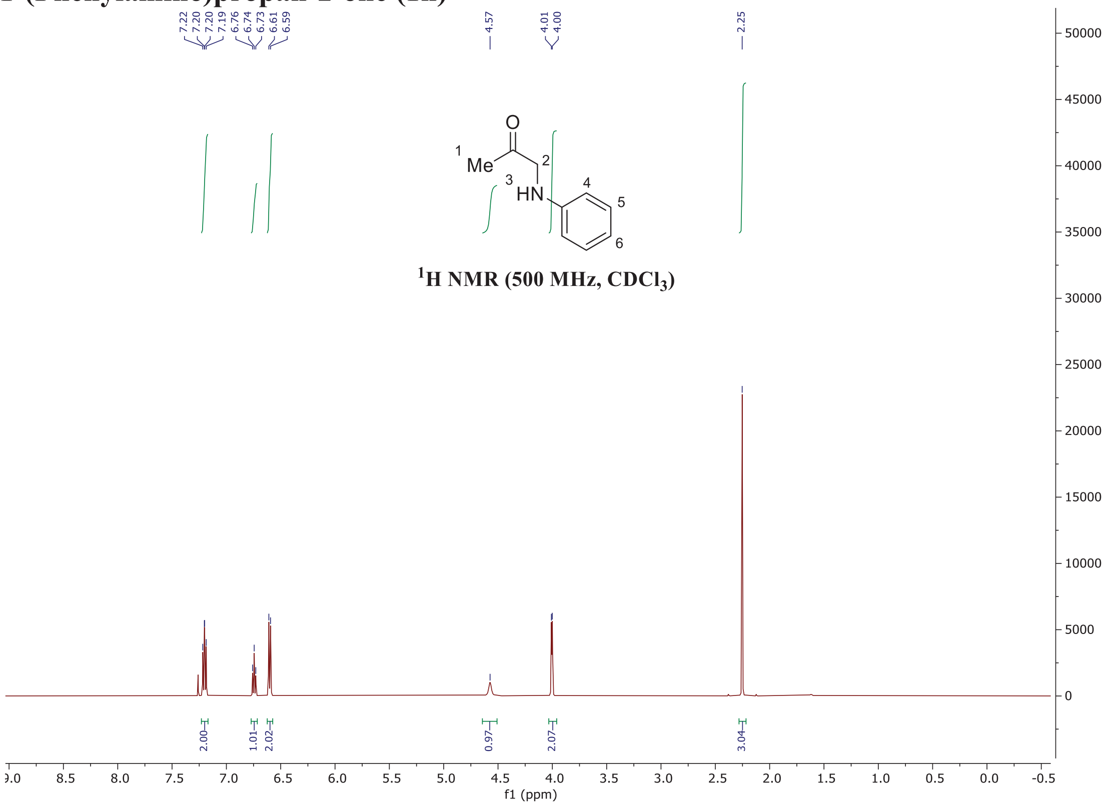

3,3-Dimethyl-1-(phenylamino)butan-2-one (1o)

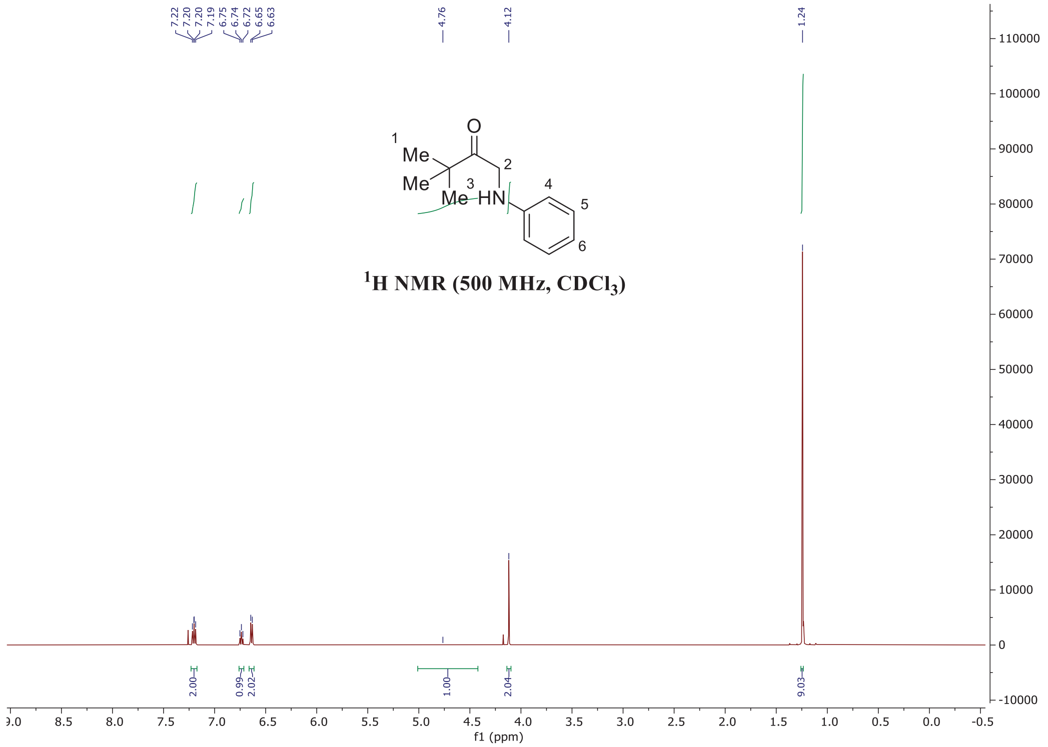

2-((4-Hydroxyphenyl)amino)-*N*,*N*-dimethylacetamide (1p)

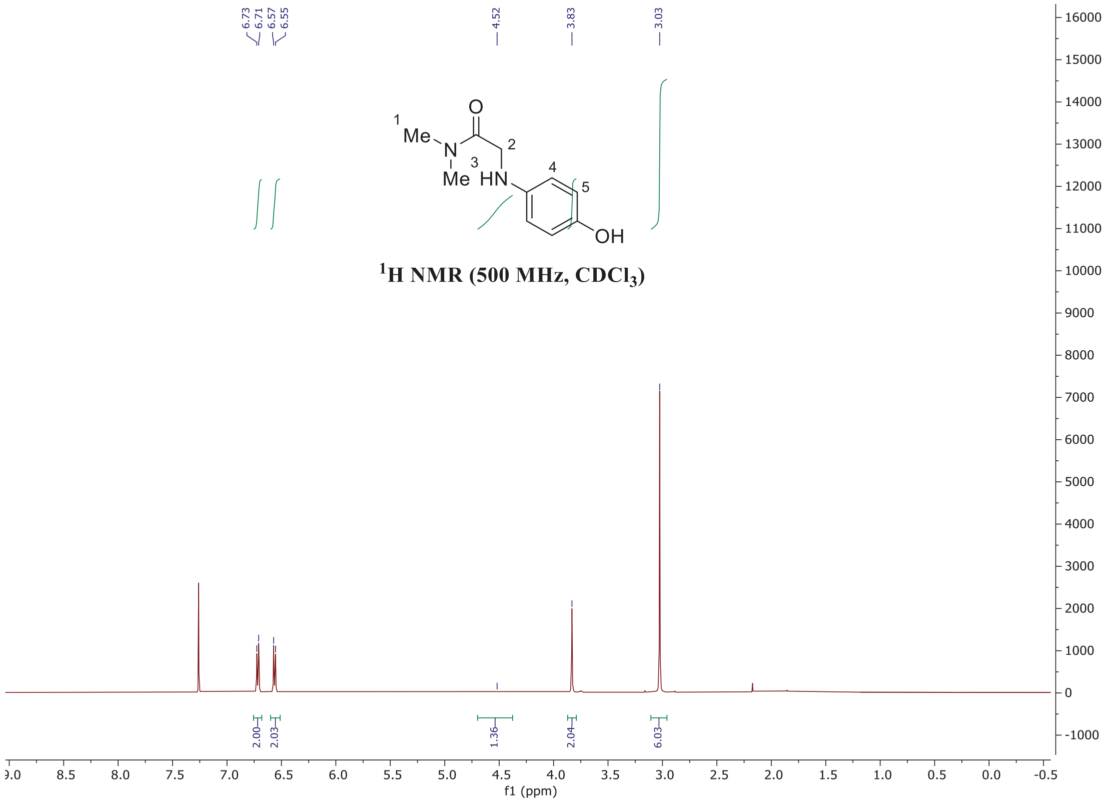

2-((4-Methoxyphenyl)amino)-*N*,*N*-dimethylacetamide (1q)

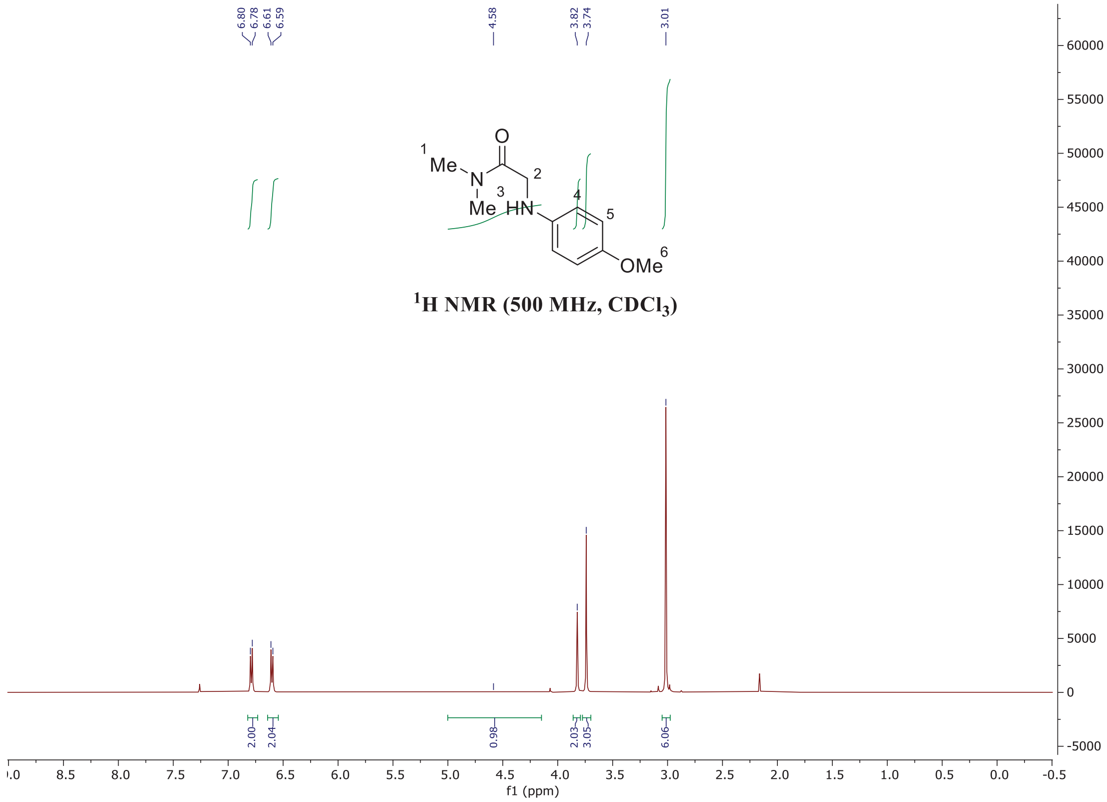

N,*N*-Dimethyl-2-(*p*-tolylamino)acetamide (1r)

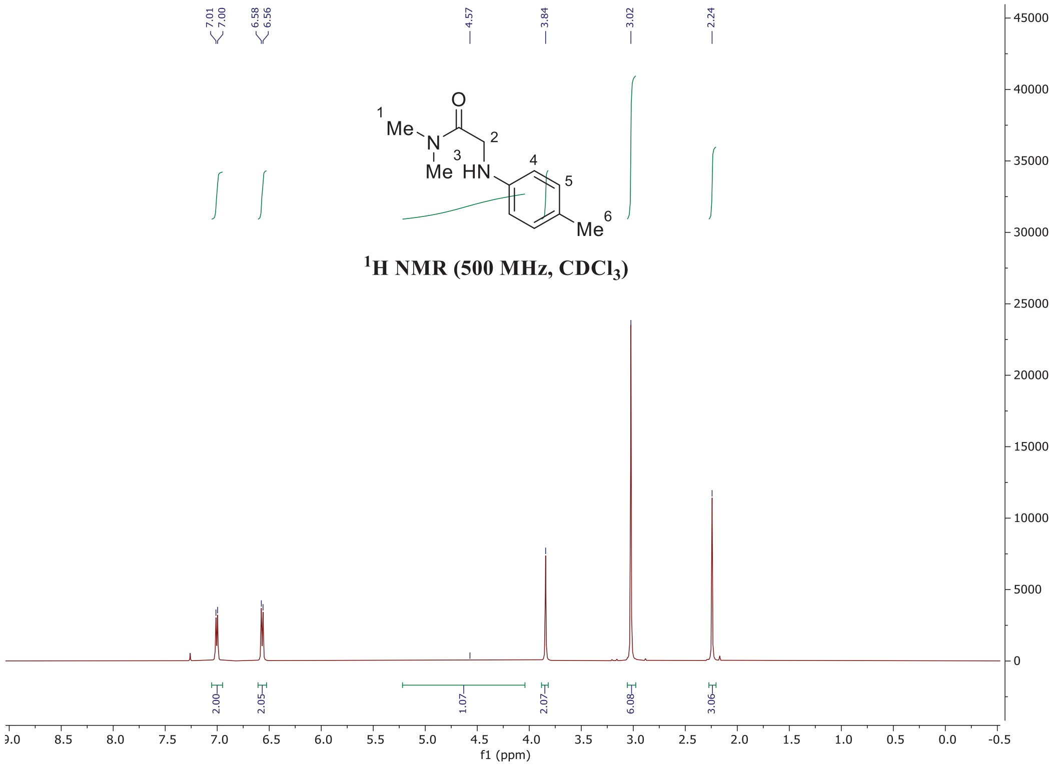

2-((4-Fluorophenyl)amino)-*N*,*N*-dimethylacetamide (1s)

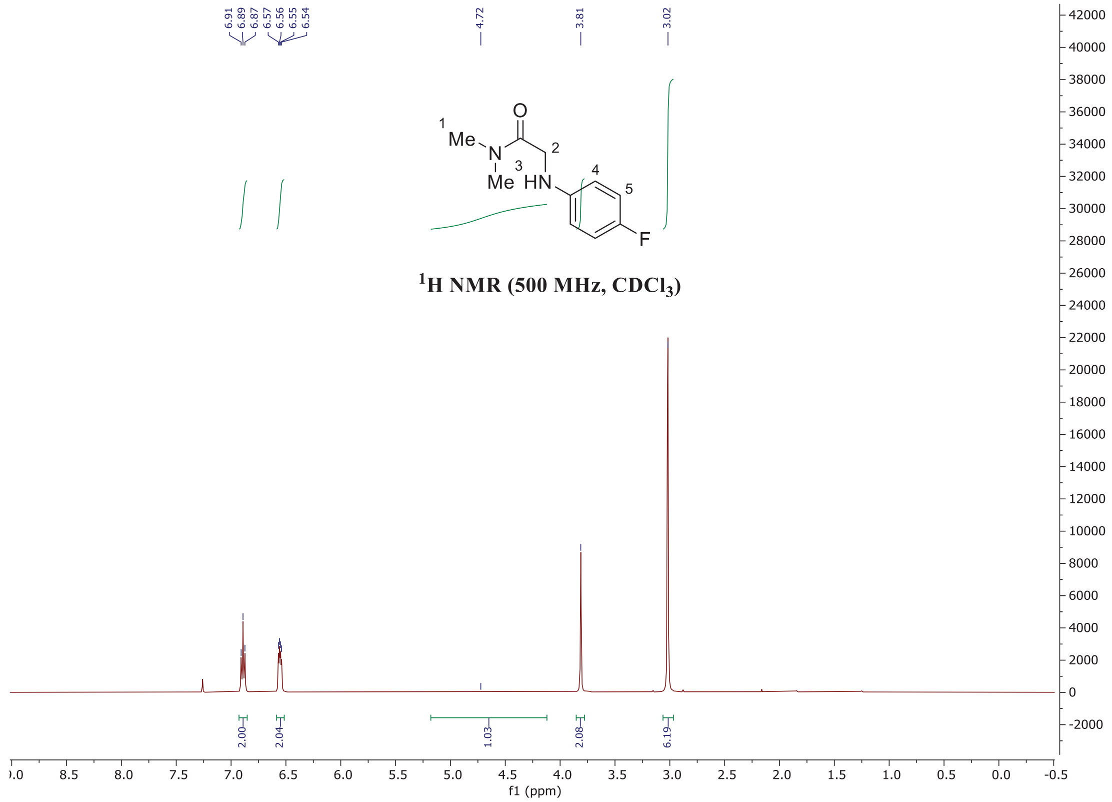

2-((4-Chlorophenyl)amino)-*N,N*-dimethylacetamide (1t)

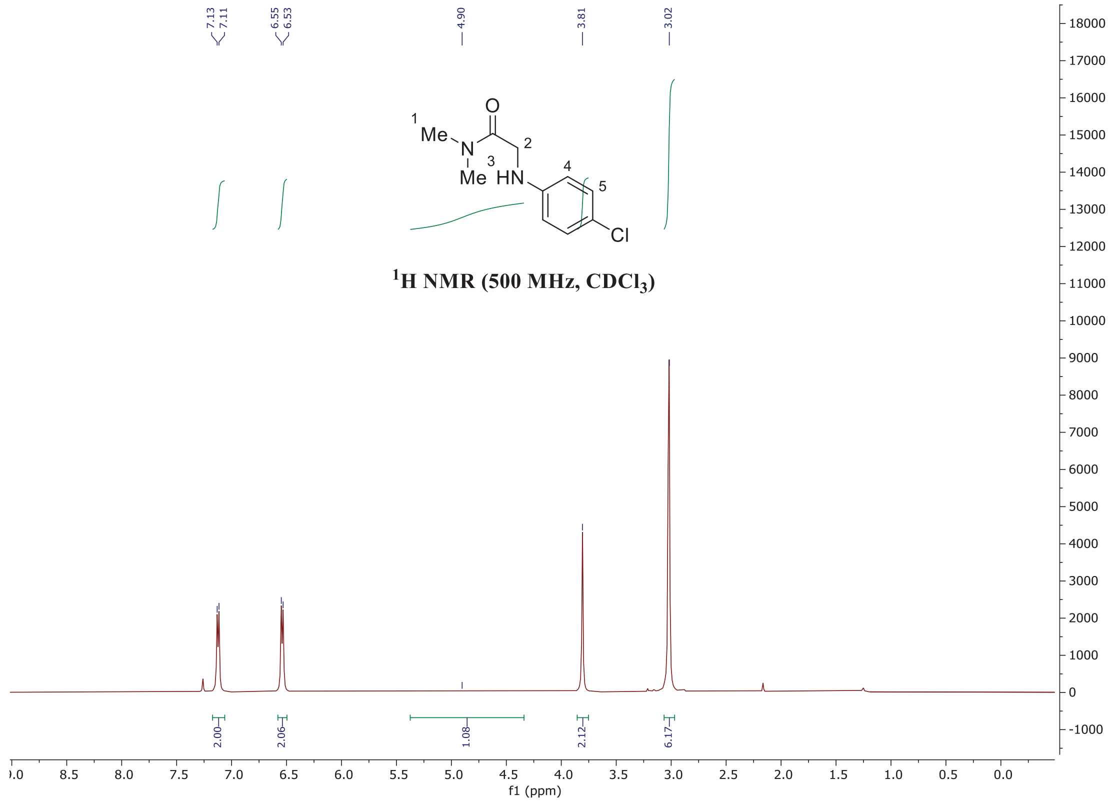

N,N-Dimethyl-2-((4-(trifluoromethyl)phenyl)amino)acetamide (1u)

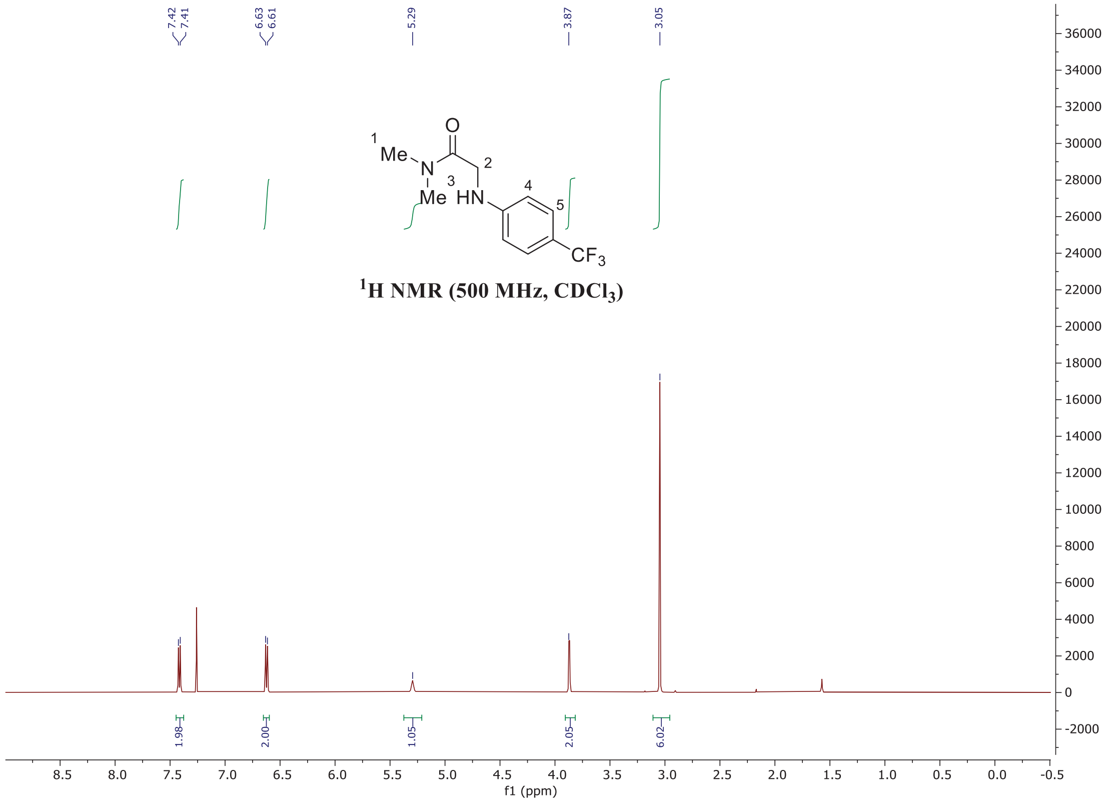

2-((4-Fluoro-3-methoxyphenyl)amino)-*N*,*N*-dimethylacetamide (1v)

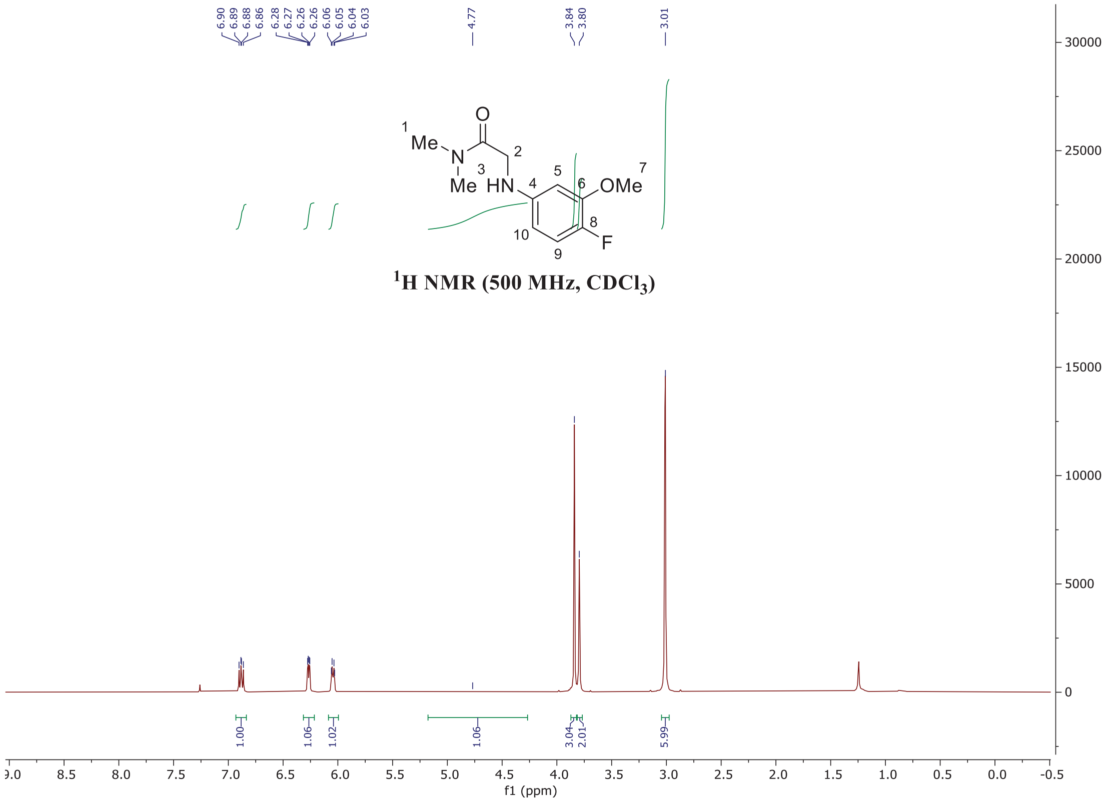

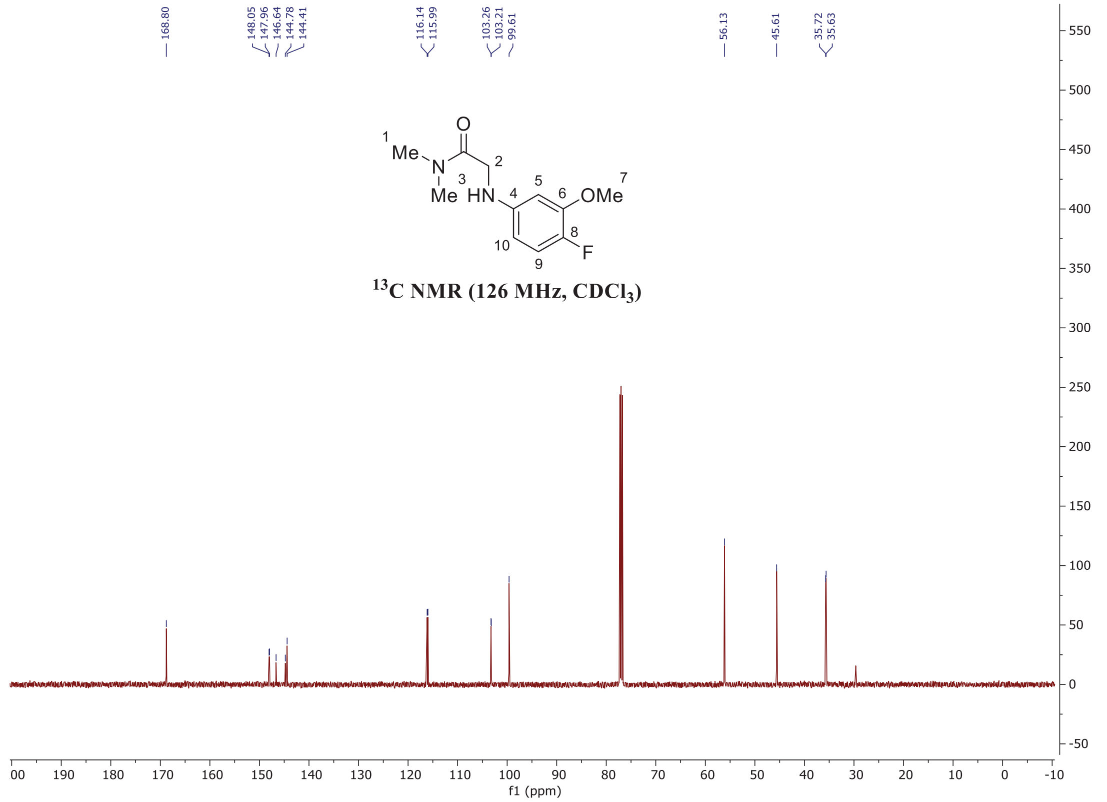
168.80
148.05
147.96
146.64
144.78
144.41
116.14
115.99
103.26
103.21
99.61
56.13
45.61
35.72
35.63
O
1
Me
N
2
3
Me
HN
4
5
6
OMe
7
8
10
9
F
13C NMR (126 MHz, CDCl3)
f1 (ppm)

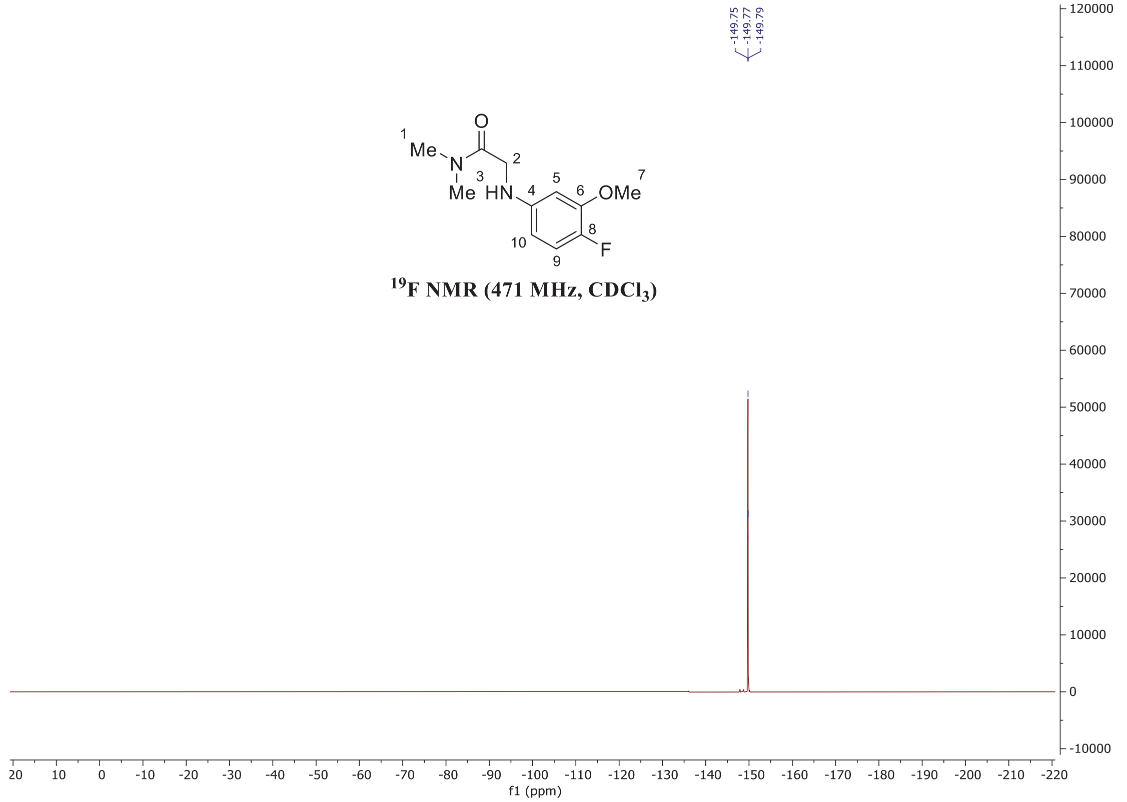
-149.75
-149.77
-149.79
O
1
Me
N
2
3
Me
HN
4
5
6
OMe
7
8
10
9
F
19F NMR (471 MHz, CDCl3)
f1 (ppm)

2-((4-Hydroxyphenyl)amino)-1-morpholinoethan-1-one (1w)

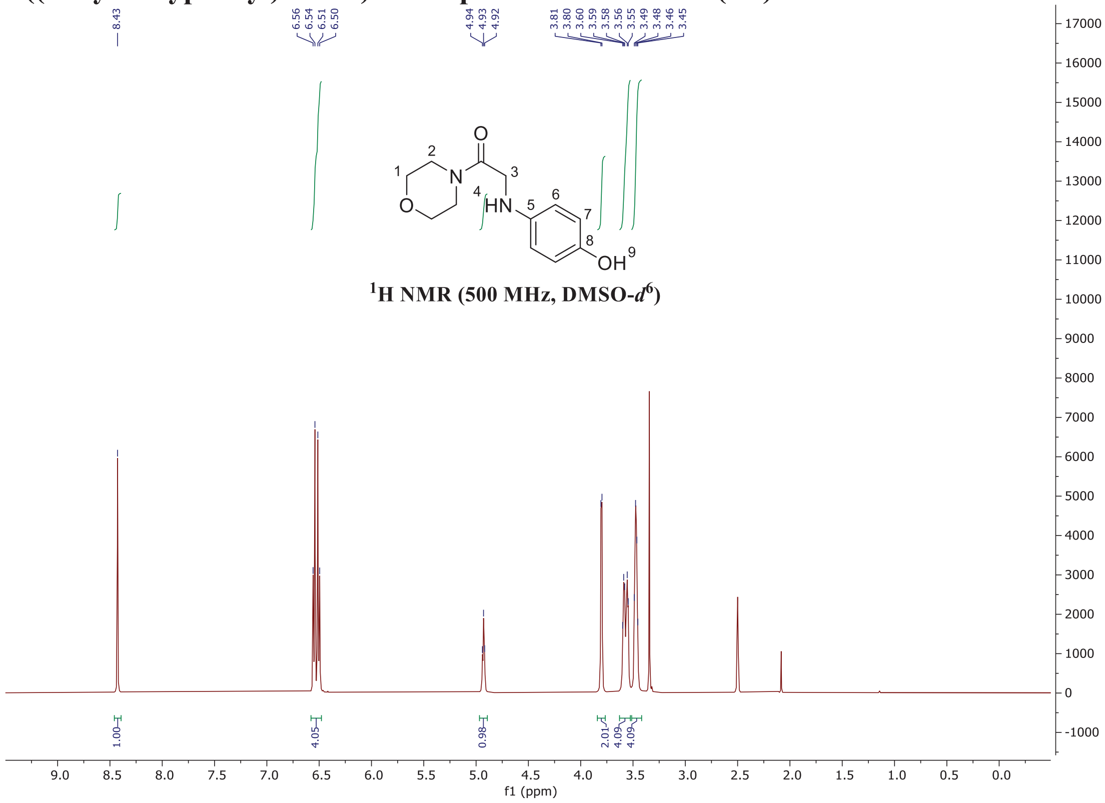

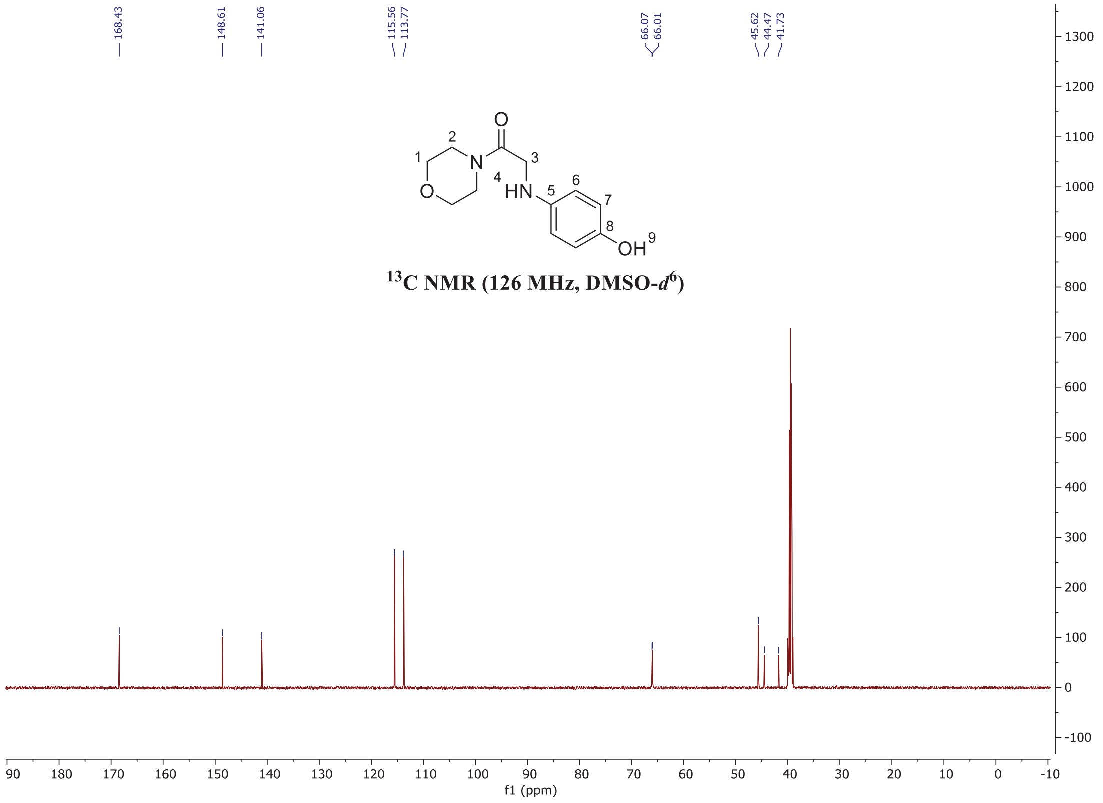

N,*N*-Dimethyl-2-(*o*-tolylamino)acetamide (1y)

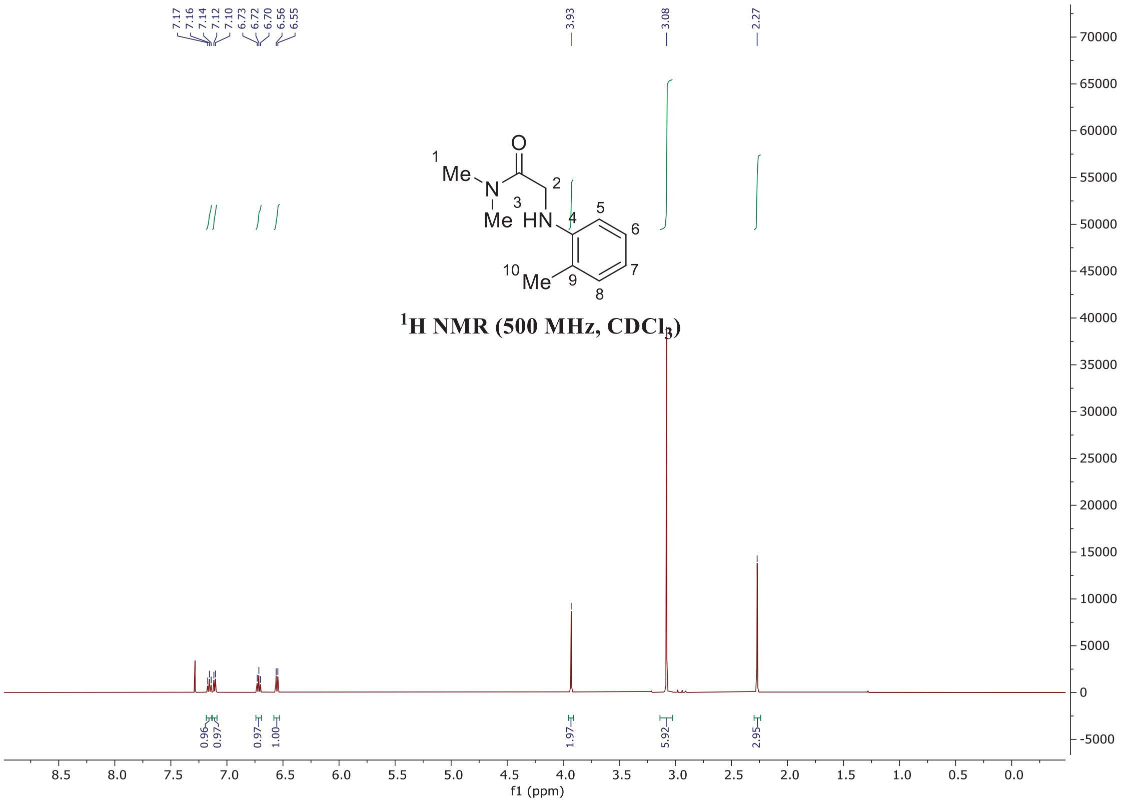

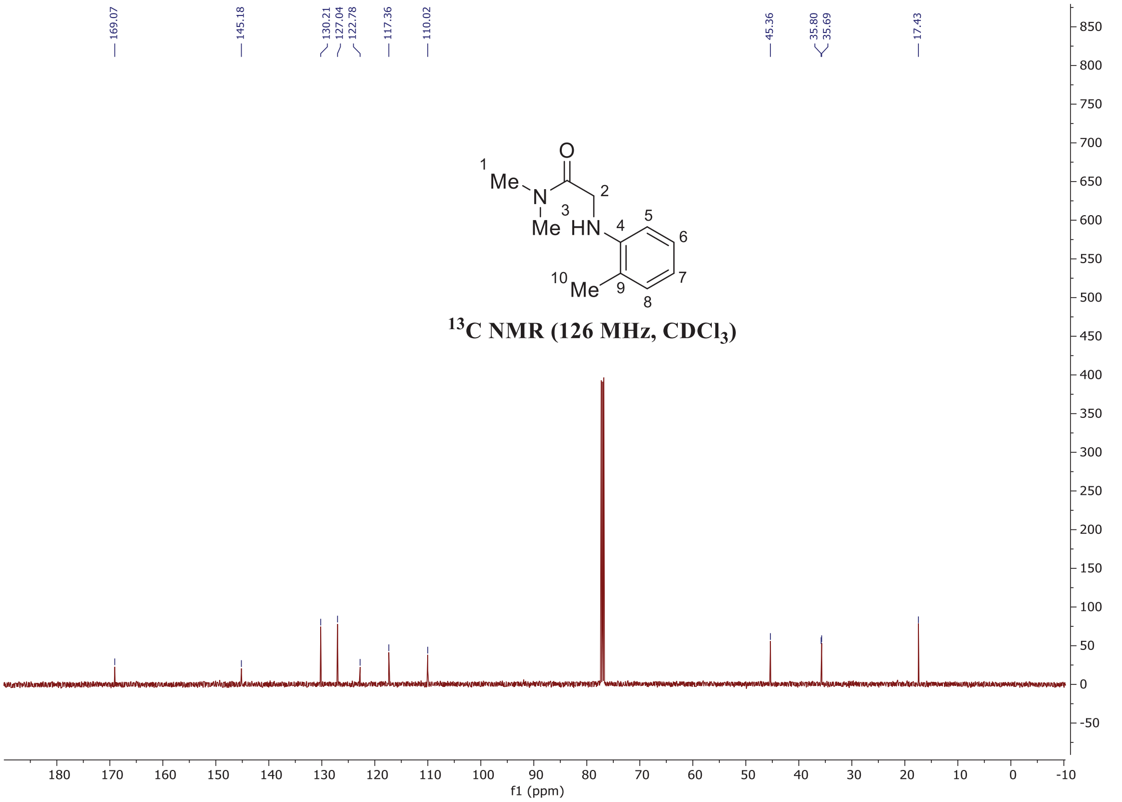

N,N-Dimethyl-3-(phenylamino)propanamide (15)

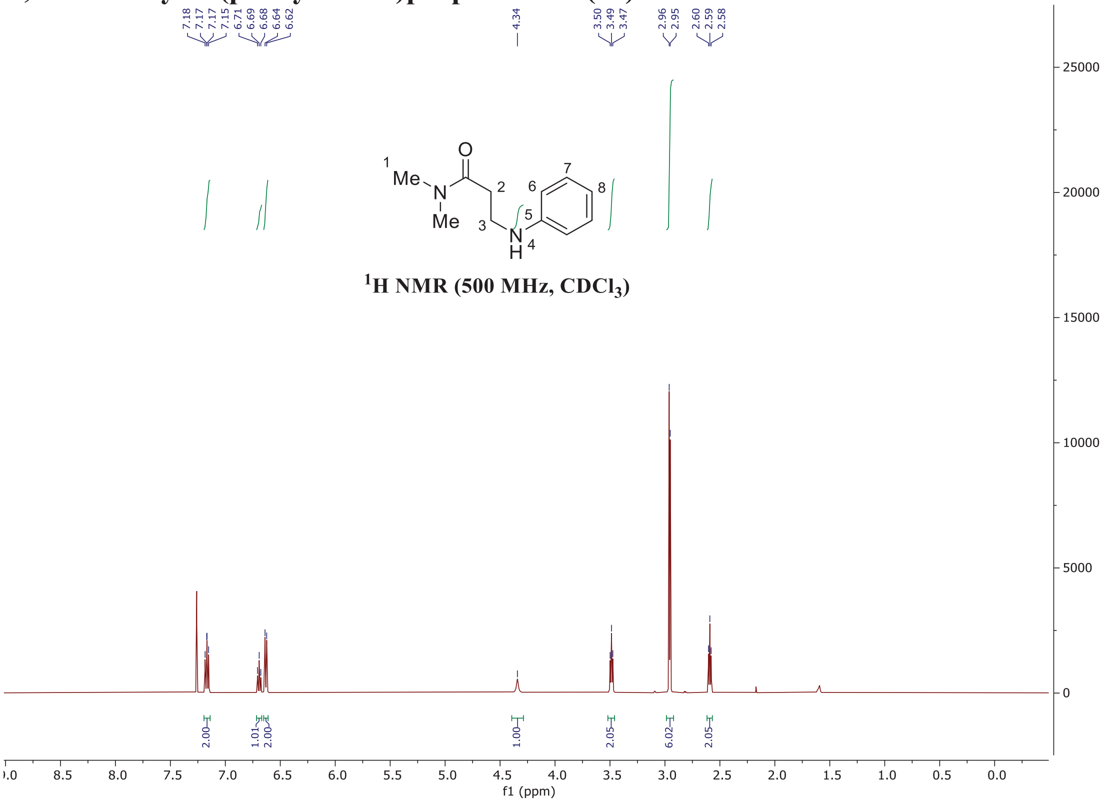

N,N-Diethyl-4-vinylbenzenesulfonamide (2d)

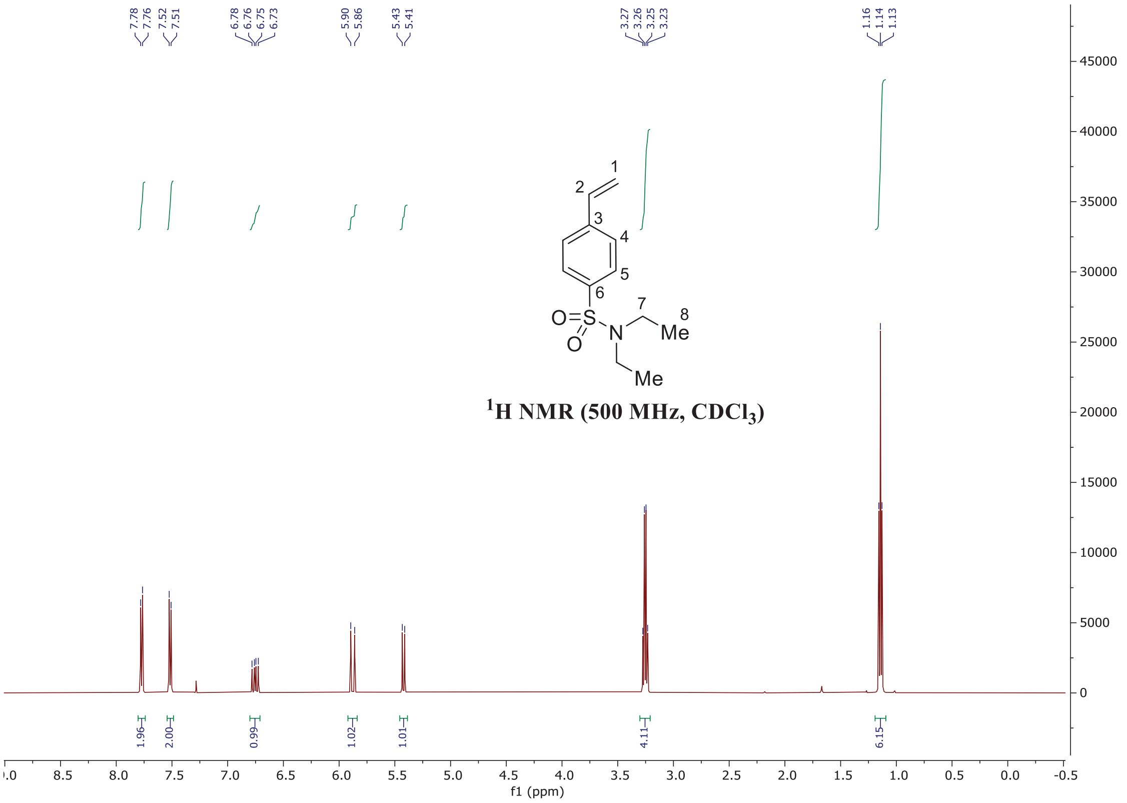

1-Tosyl-3-vinyl-1*H*-indole (2f)

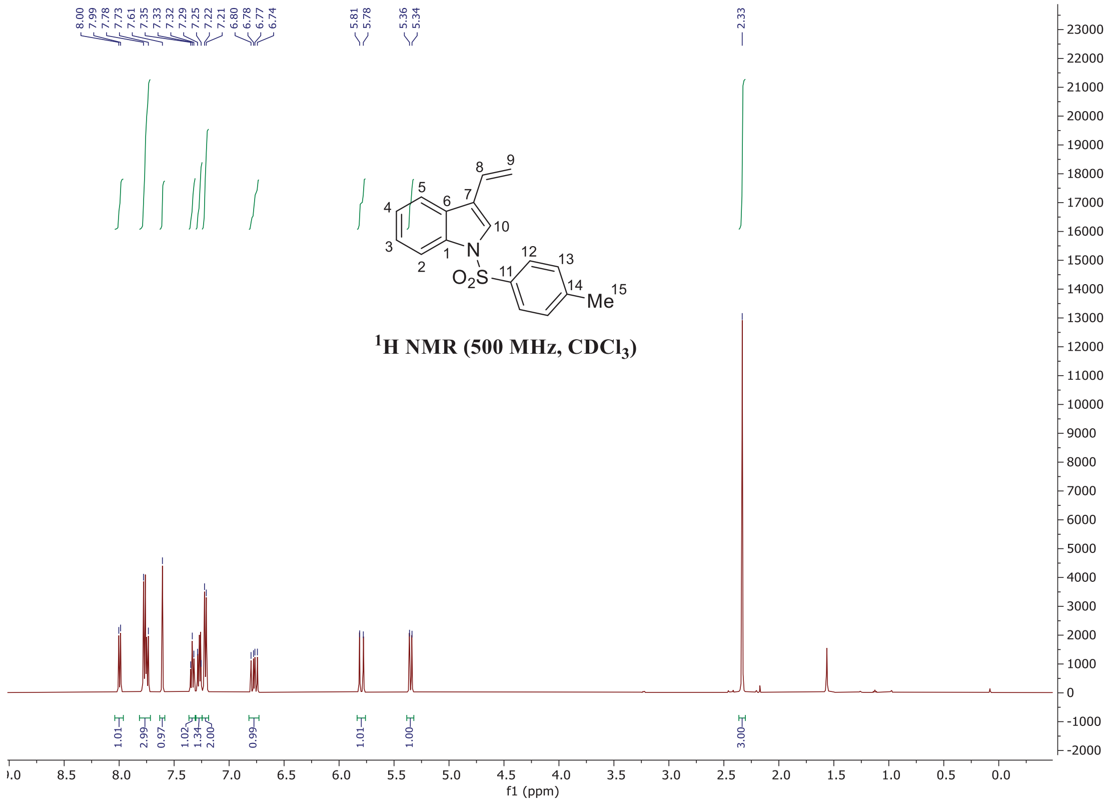

5-Vinylbenzofuran (2m):

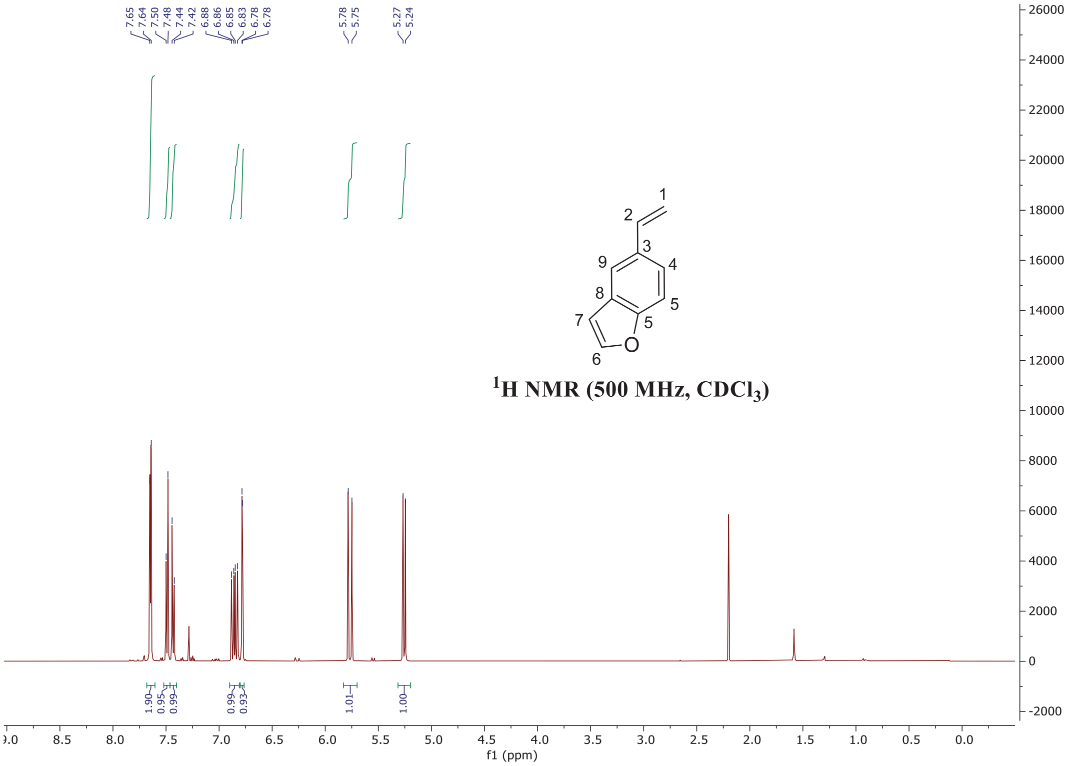

Tert-butyl(hex-5-en-1-yloxy)dimethylsilane (2s)

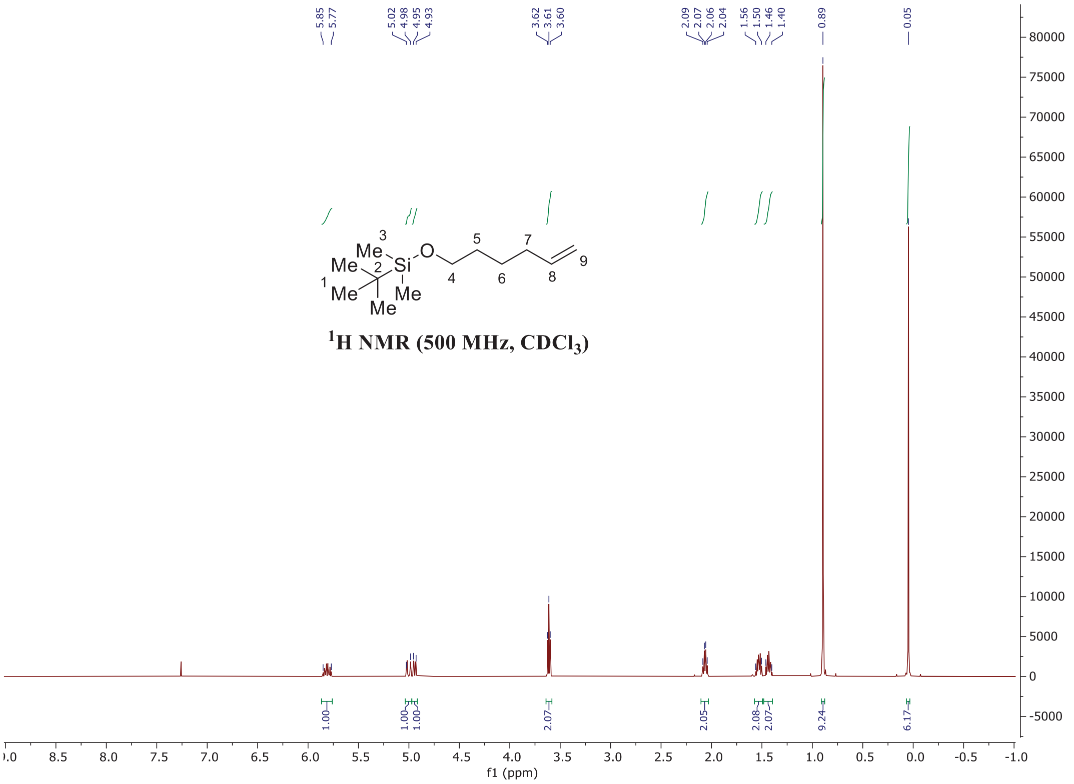

4-Vinylbenzyl 2-(1-(4-chlorobenzoyl)-5-methoxy-2-methyl-1*H*-indol-3-yl)acetate (2t)

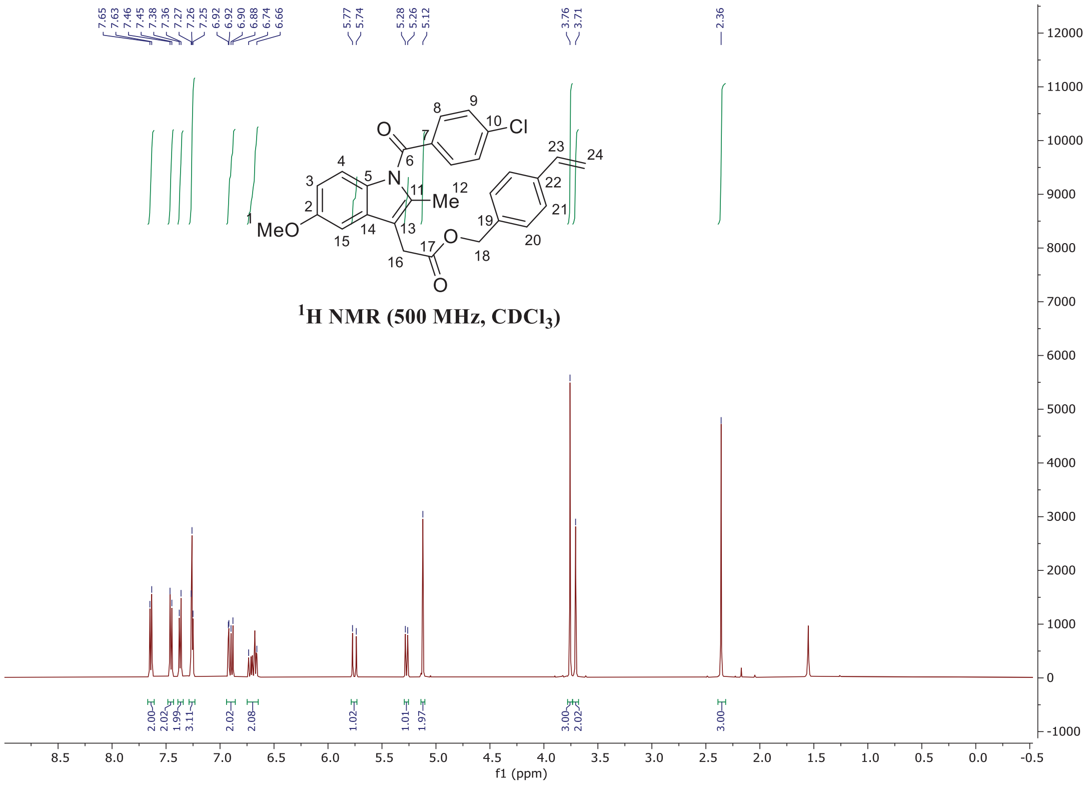

(*2S,3S*)-*N,N*-Dimethyl-3-phenyl-2-(phenylamino)butanamide (3fa)

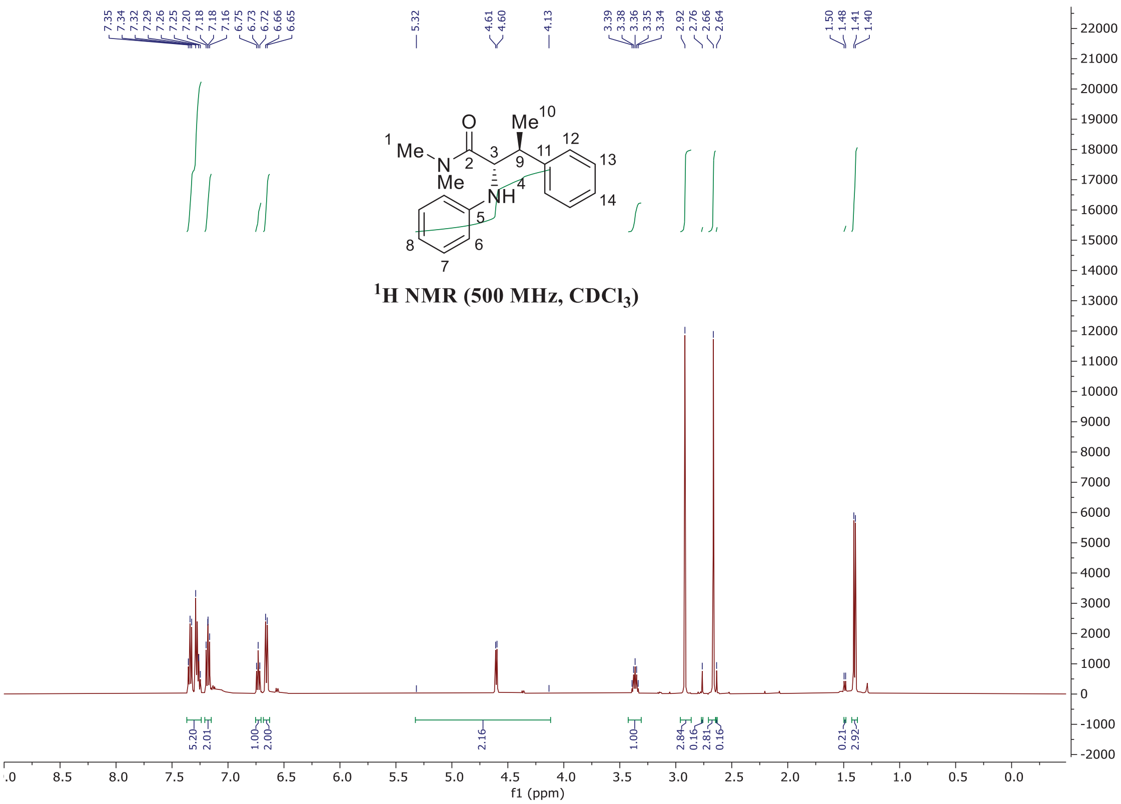

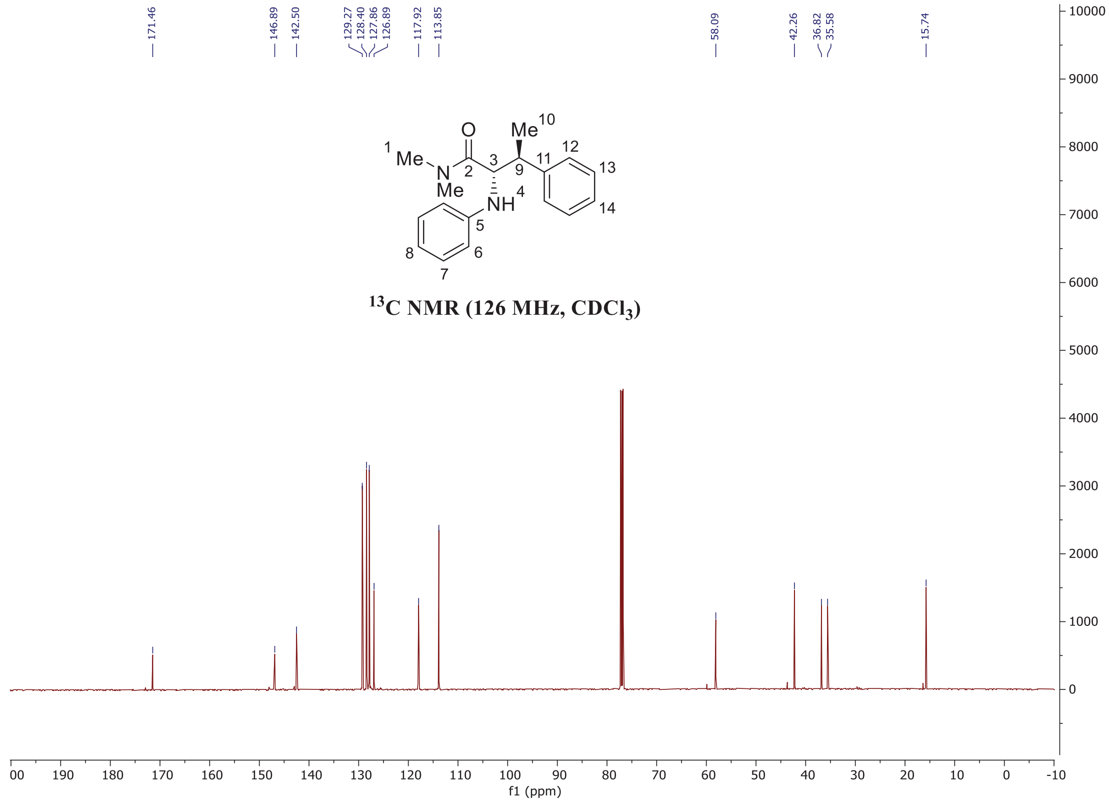

(*2S,3S*)-*N*-Methyl-3-phenyl-2-(phenylamino)butanamide (3ga)

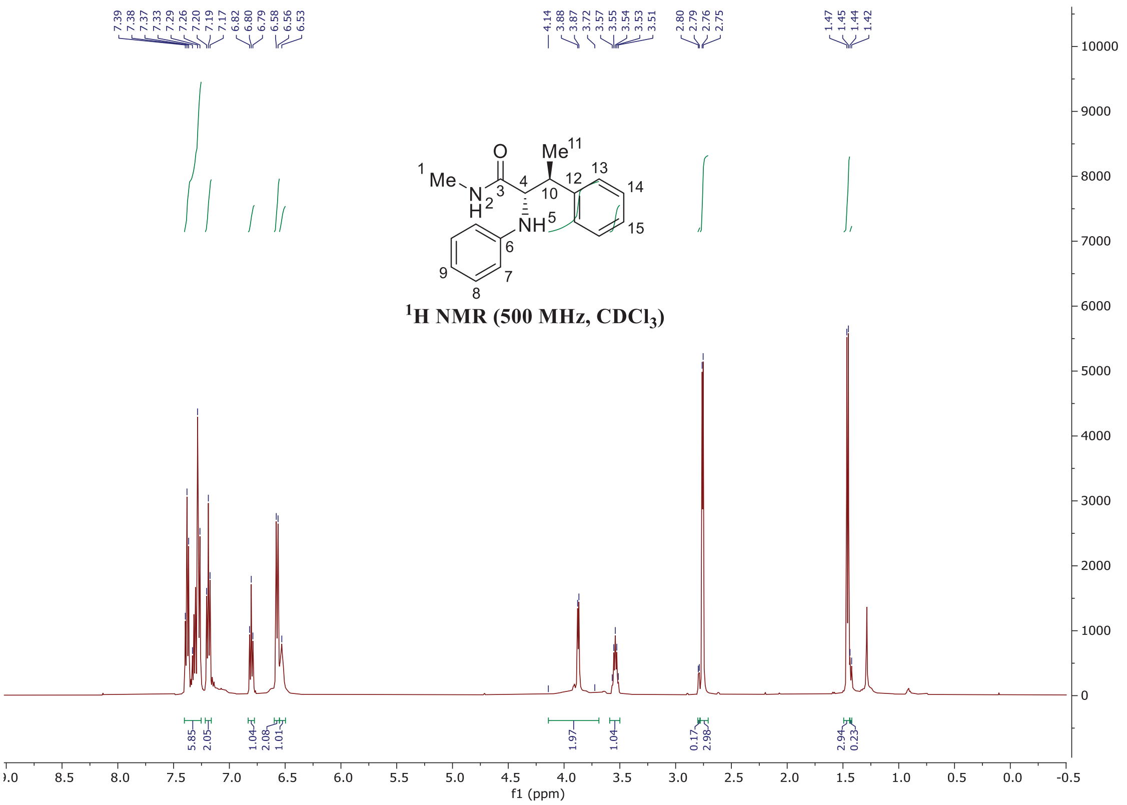

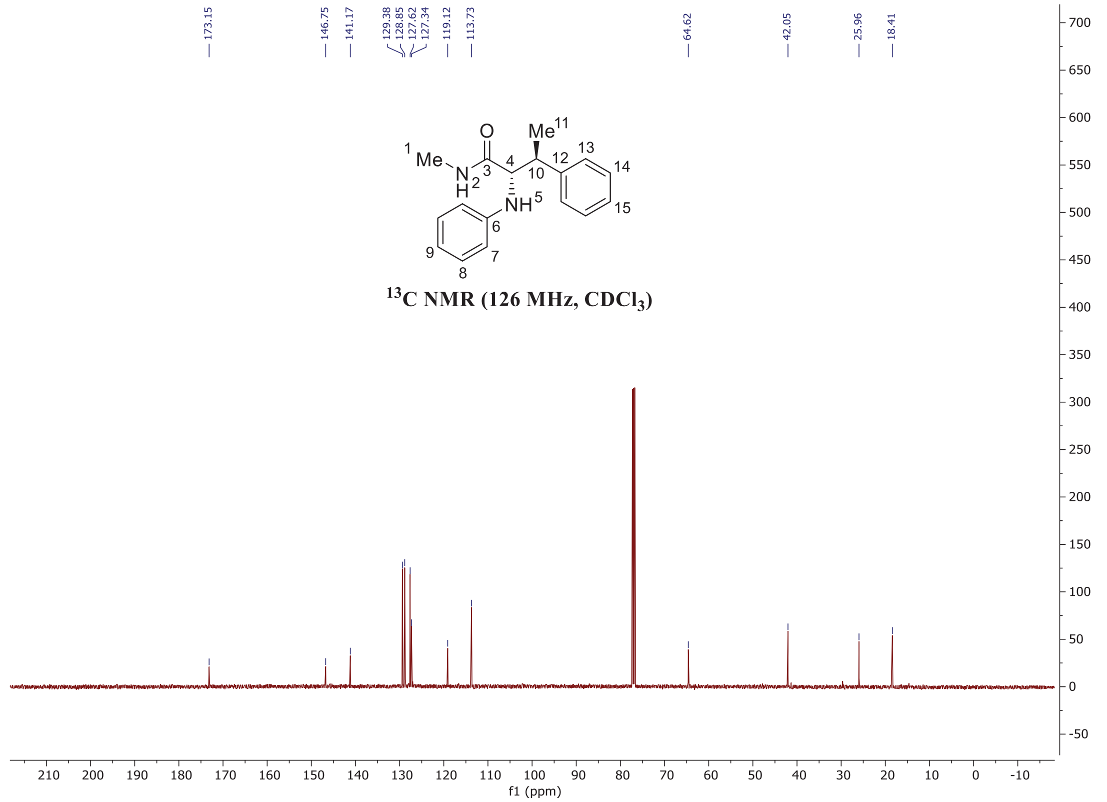

(*2S,3S*)-*N,N*-Diethyl-3-phenyl-2-(phenylamino)butanamide (3ha)

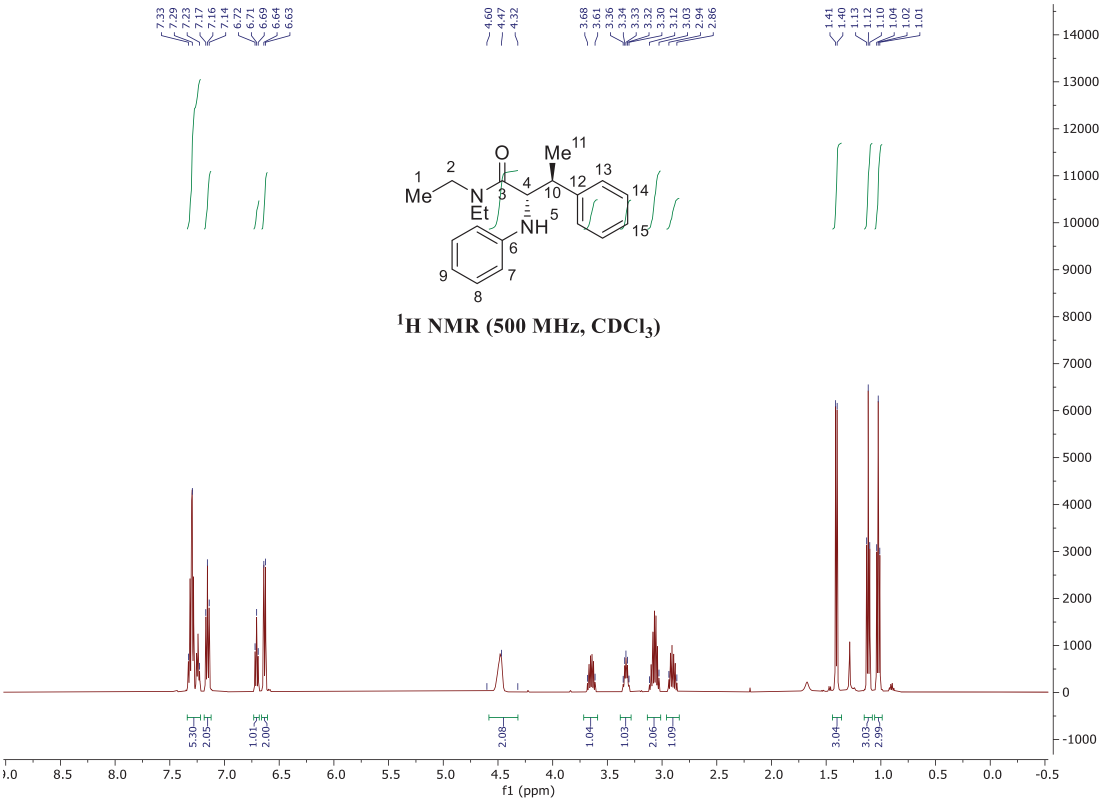

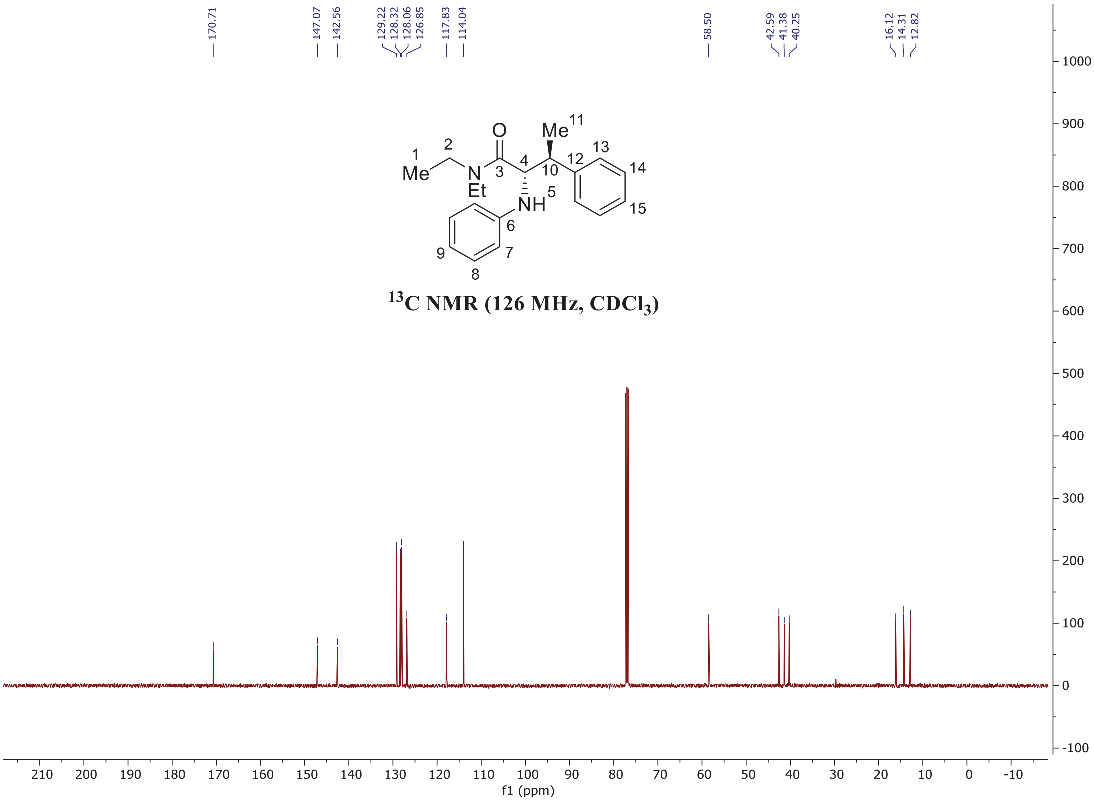

(*2S,3S*)-3-Phenyl-2-(phenylamino)-1-(pyrrolidin-1-yl)butan-1-one (3ia)

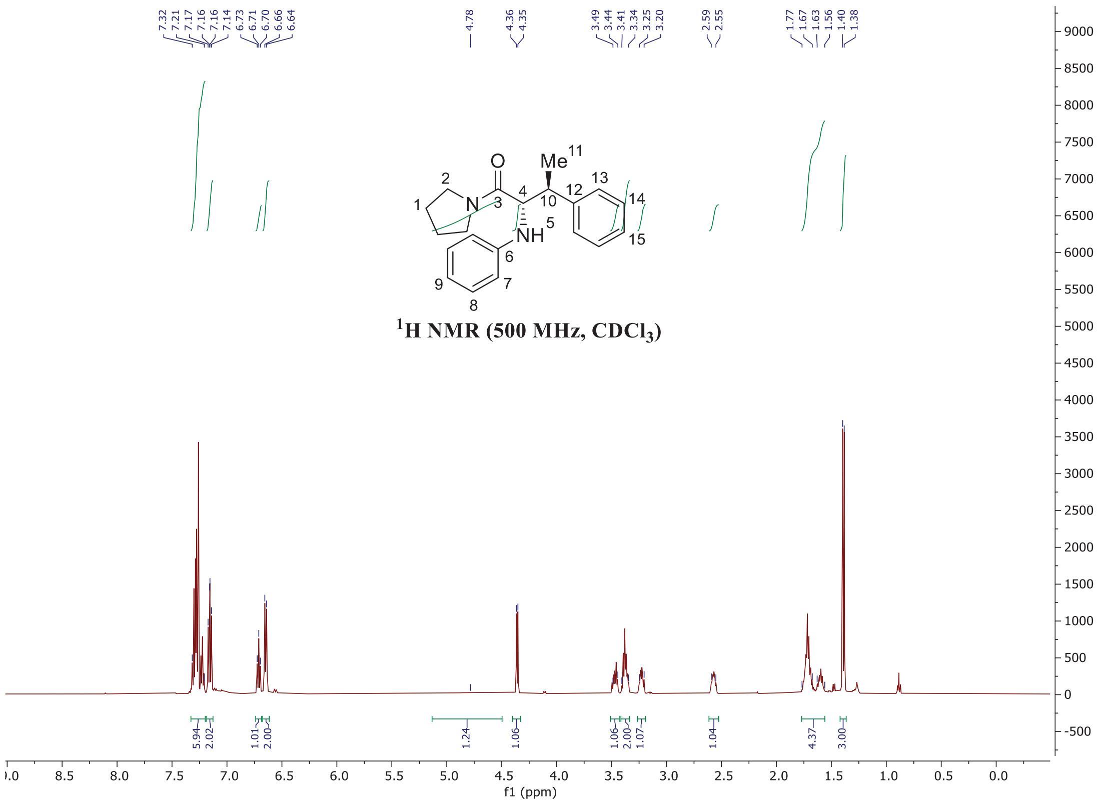

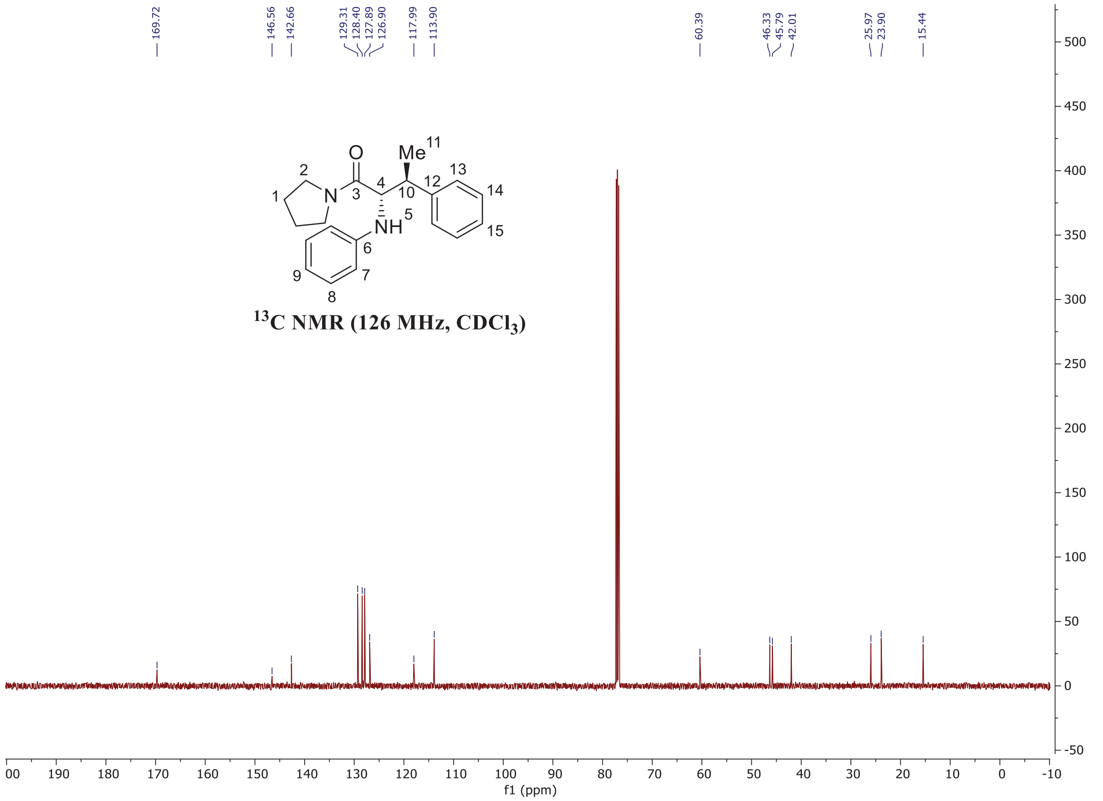

(*2S*,*3S*)-3-Phenyl-2-(phenylamino)-1-(piperidin-1-yl)butan-1-one (3ja)

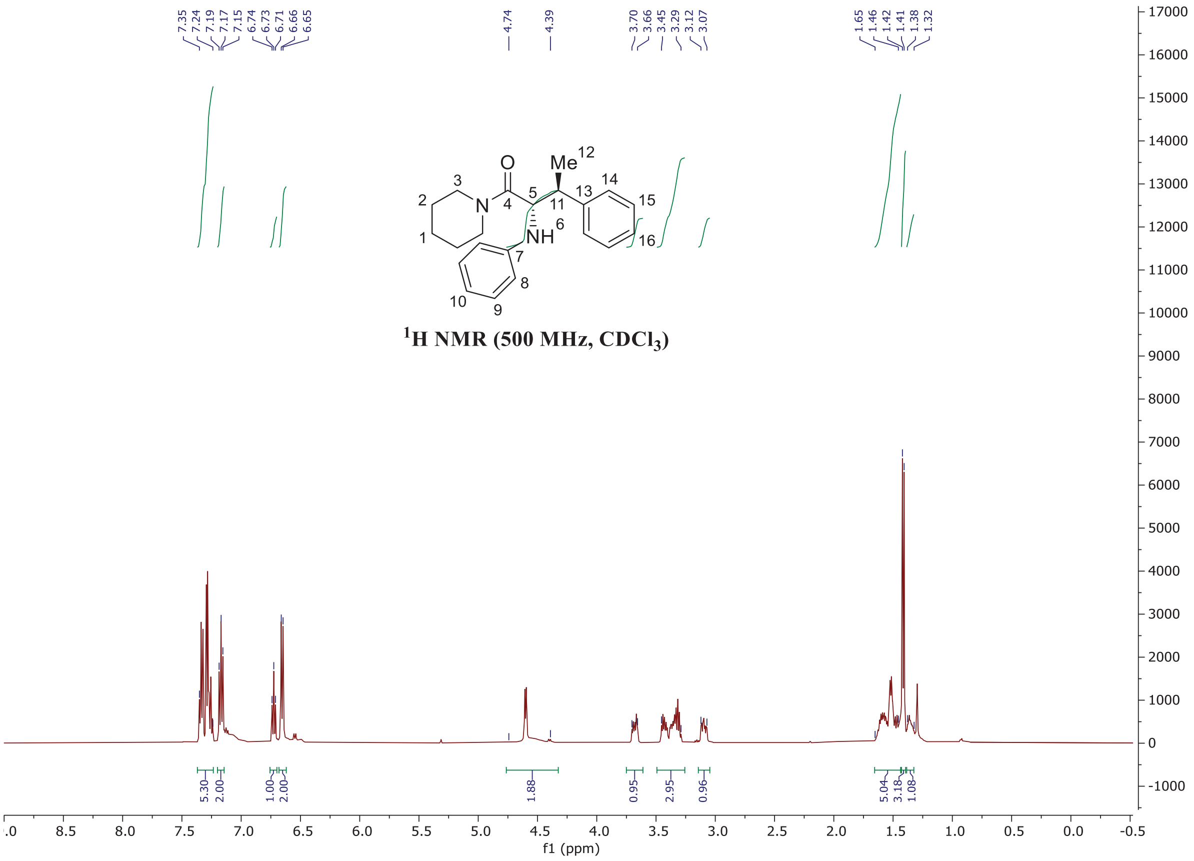

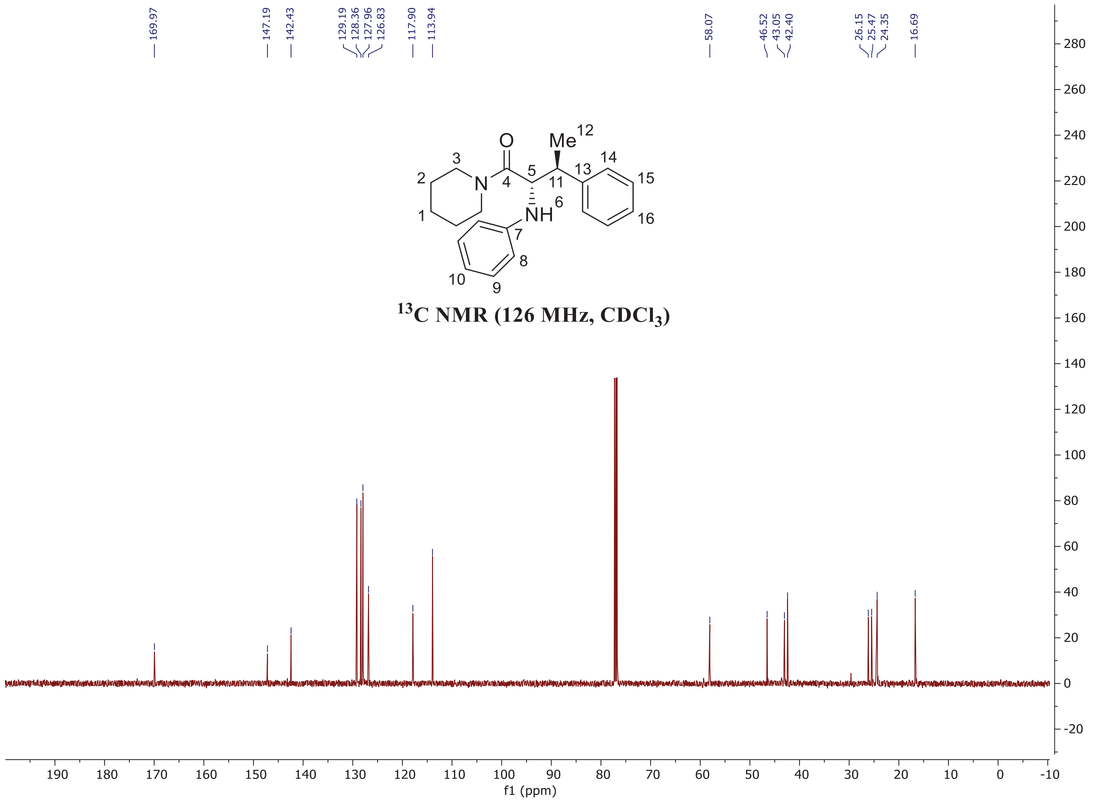

(2S,3S)-1-Morpholino-3-phenyl-2-(phenylamino)butan-1-one (3ka)

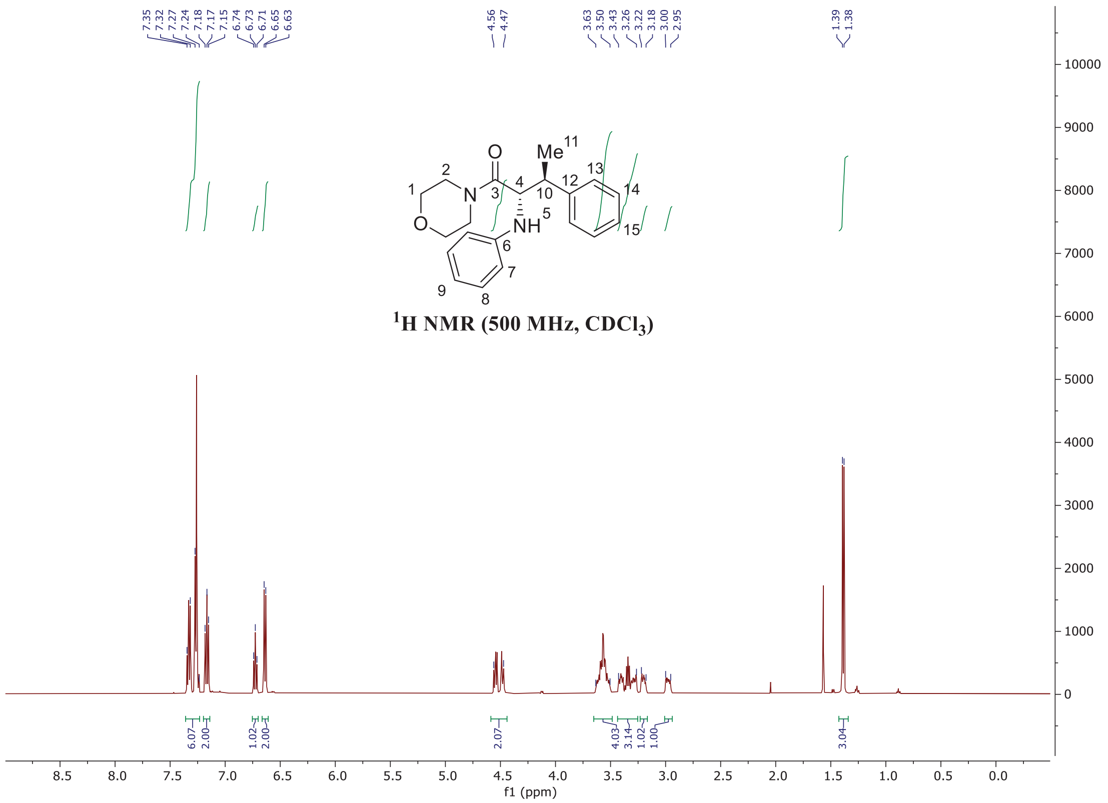

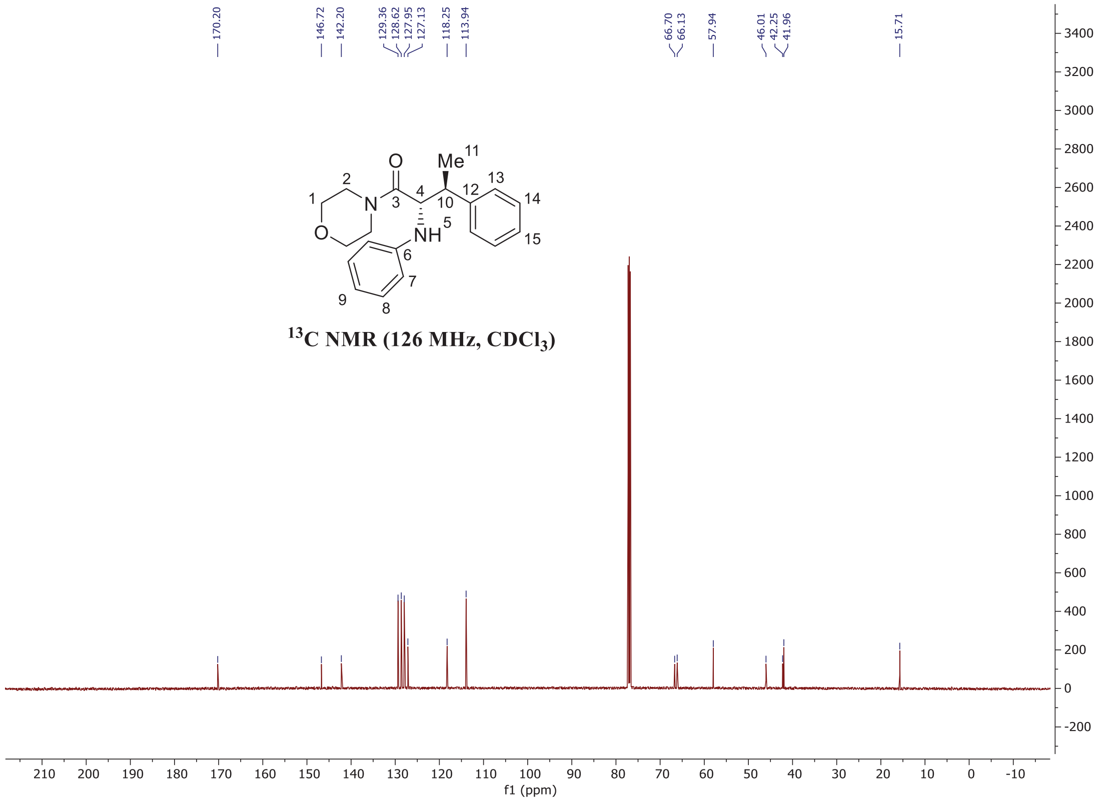

(*2S,3S*)-2-((4-Hydroxyphenyl)amino)-*N*,3-diphenylbutanamide (3la)

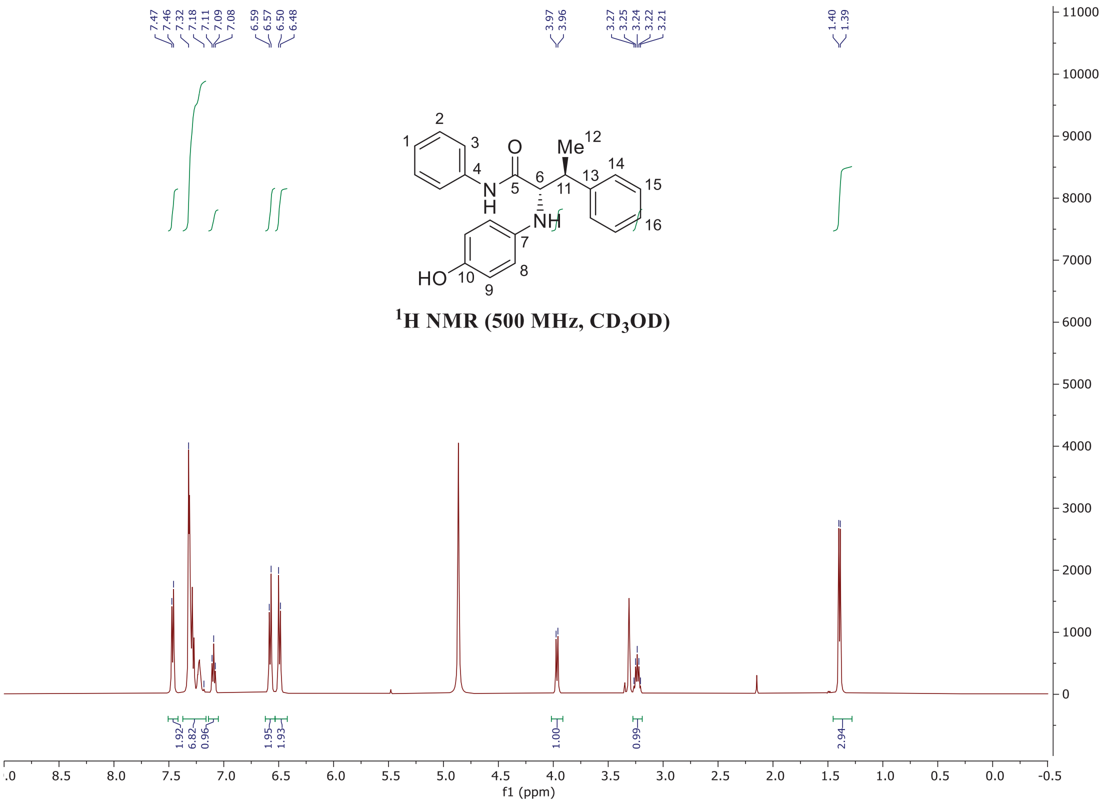

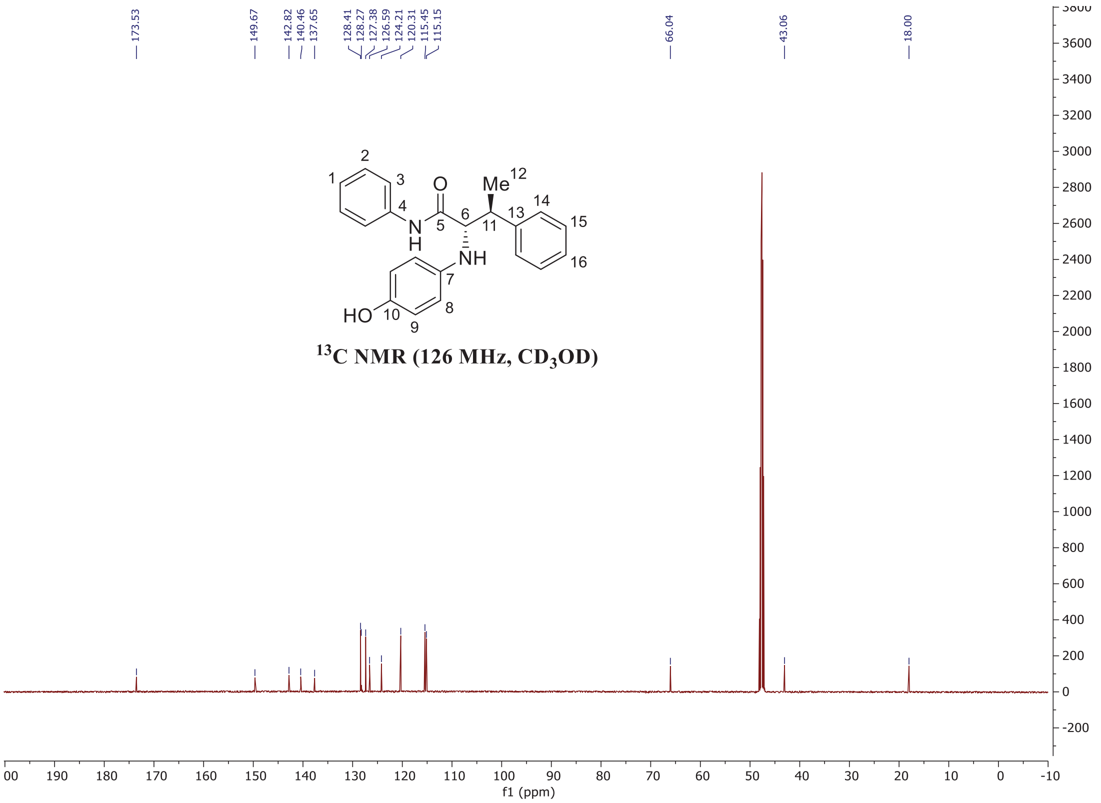

(2S,3S)-3-Phenyl-2-(phenylamino)butanamide (3xa)

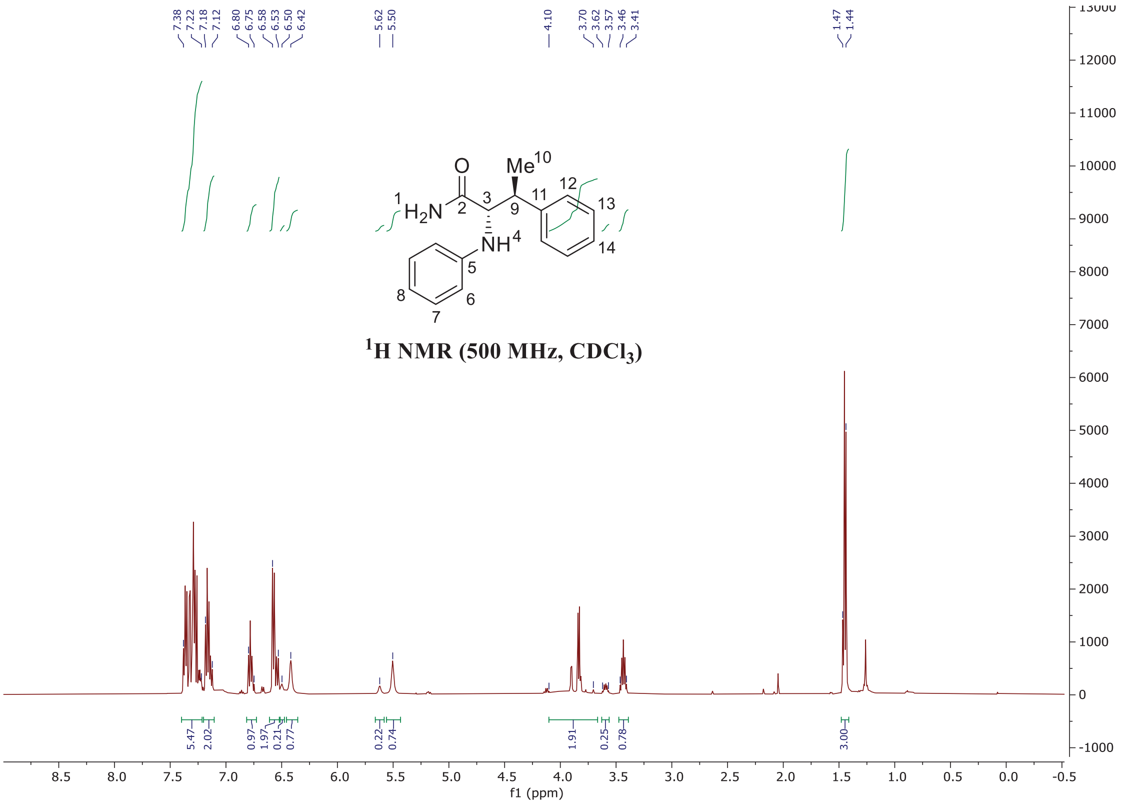

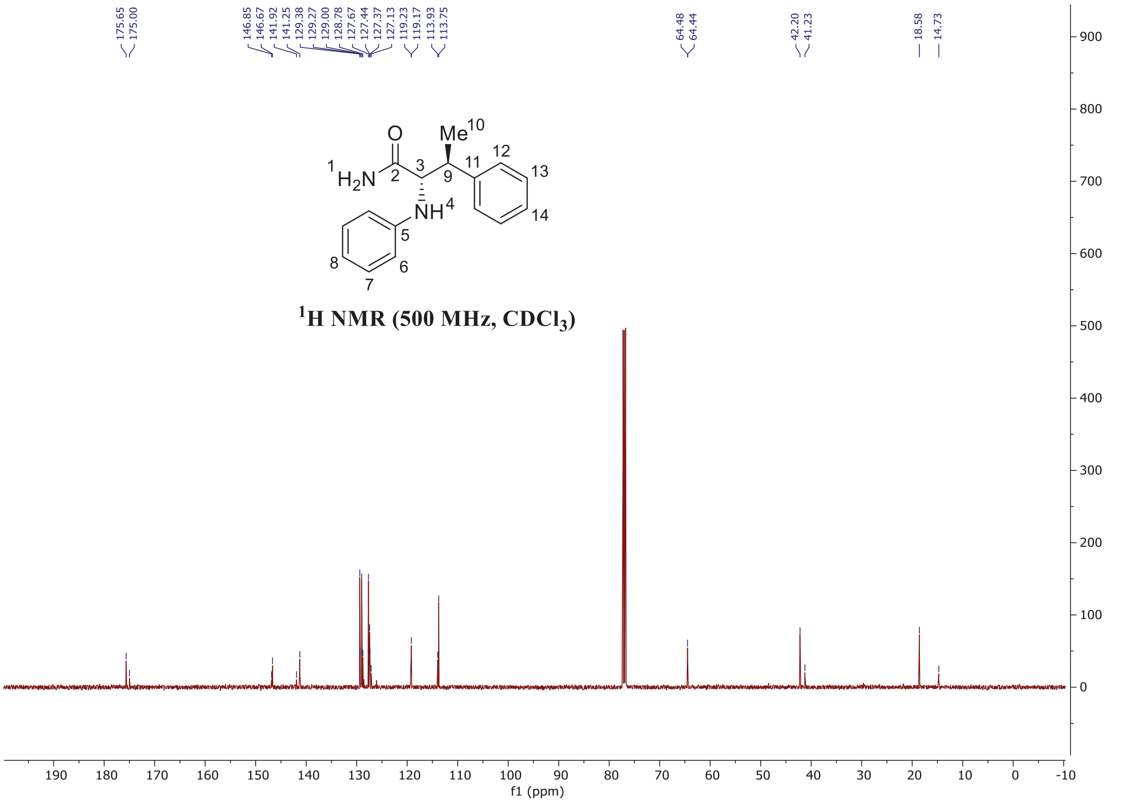

(*2S,3S*)-1,3-Diphenyl-2-(phenylamino)butan-1-one (3ma)

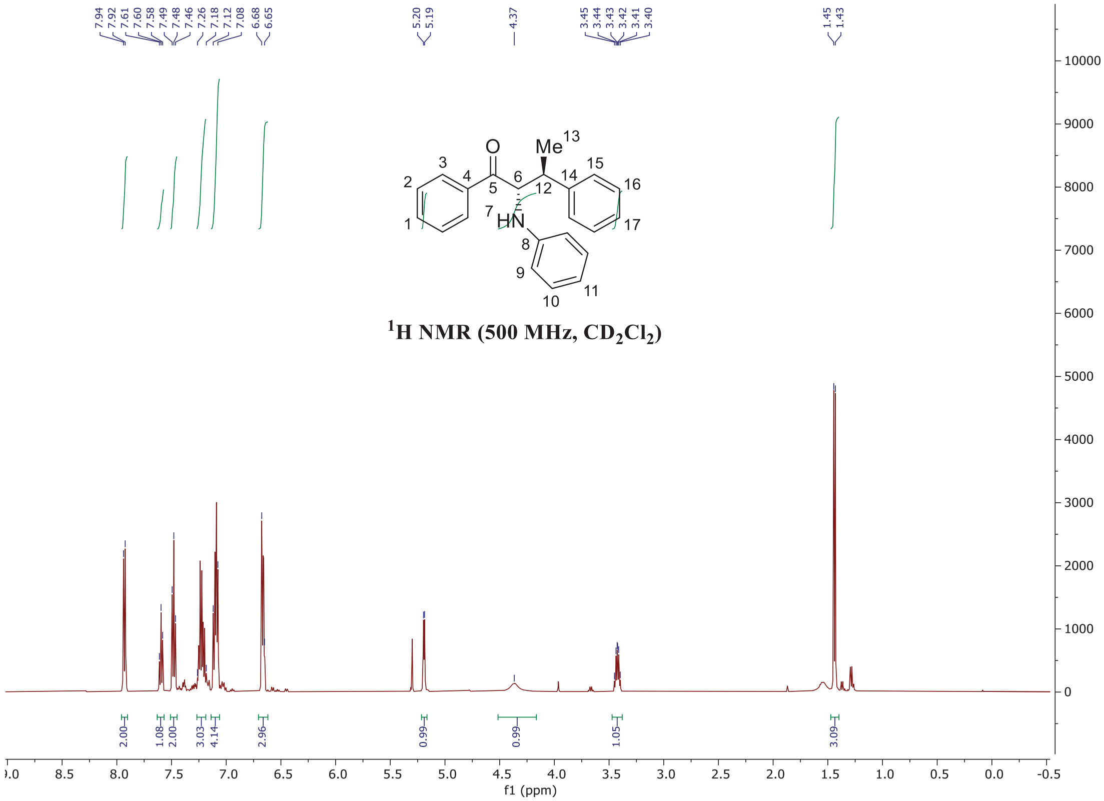

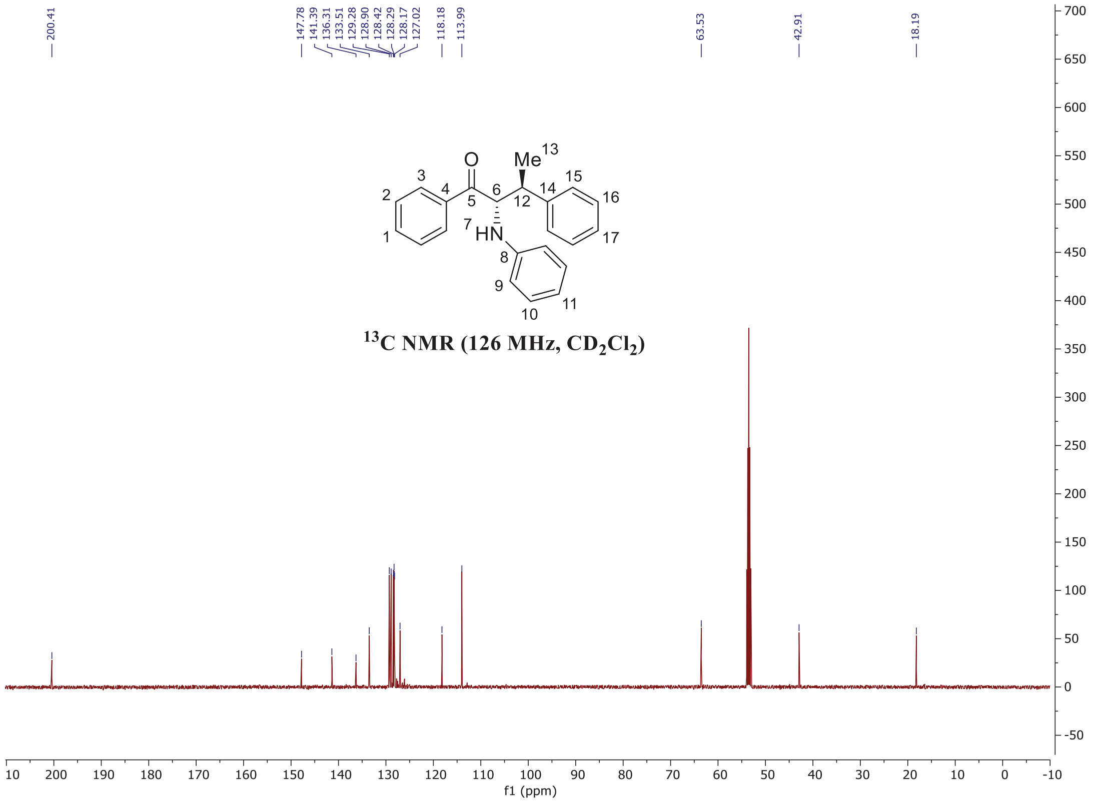

(*3S,4S*)-4-Phenyl-3-(phenylamino)pentan-2-one (3na)

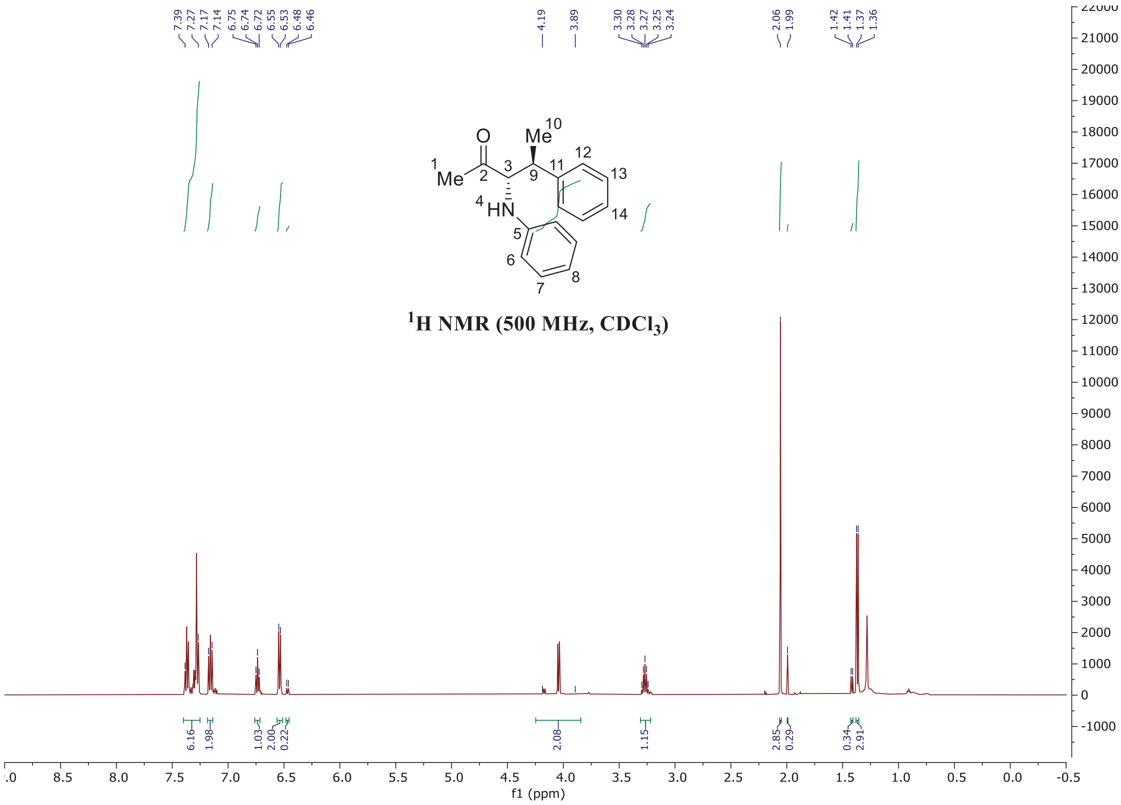

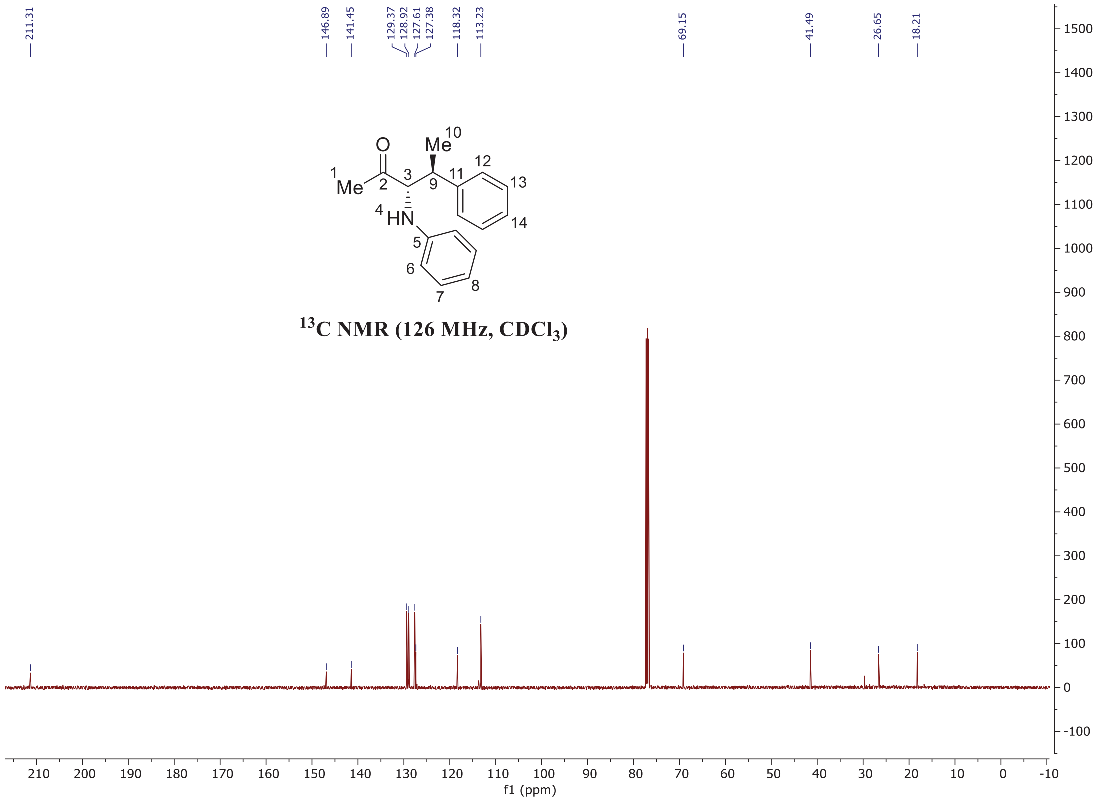

(*4S,5S*)-2,2-Dimethyl-5-phenyl-4-(phenylamino)hexan-3-one (3oa)

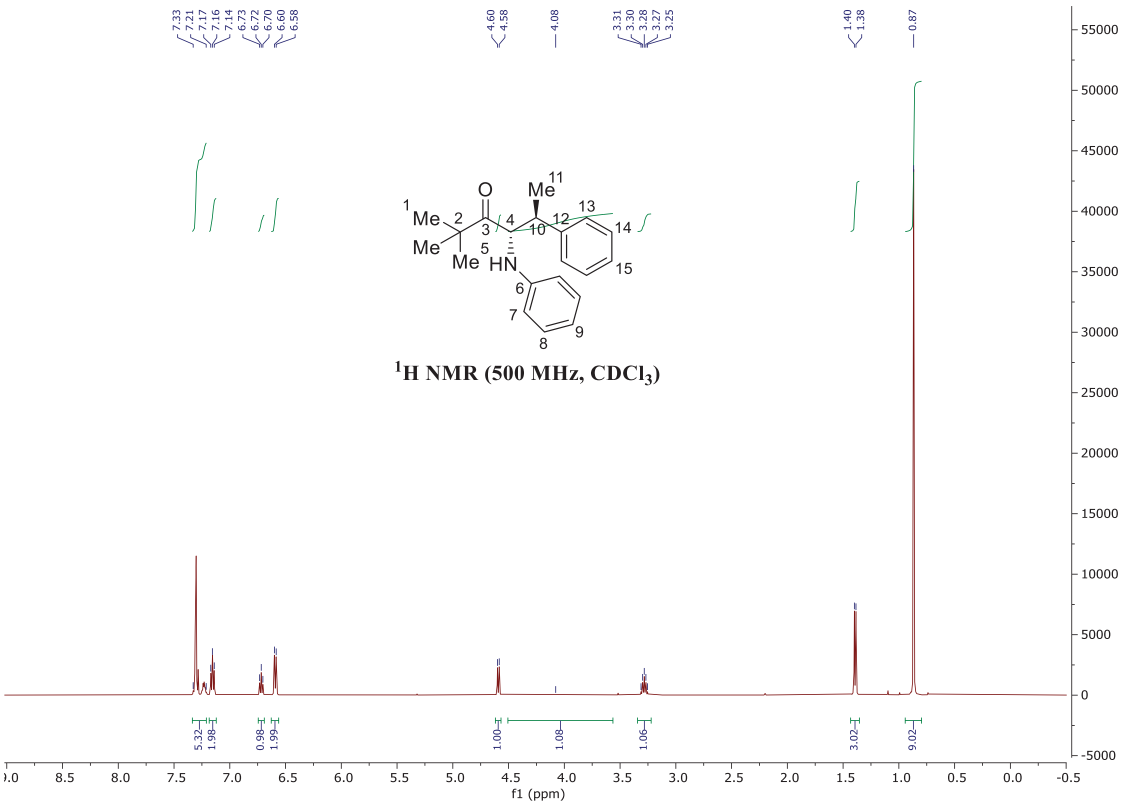

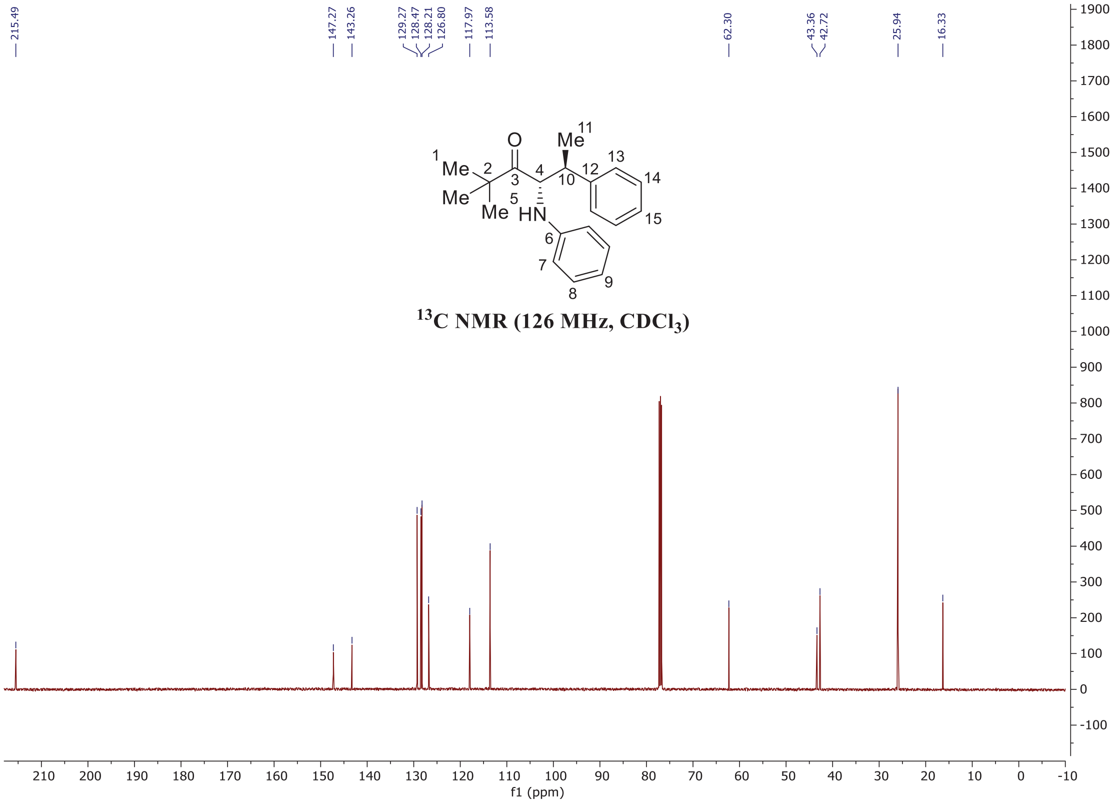

(2*S*,3*S*)-2-((4-Hydroxyphenyl)amino)-*N*,*N*-dimethyl-3-phenylbutanamide (3pa)

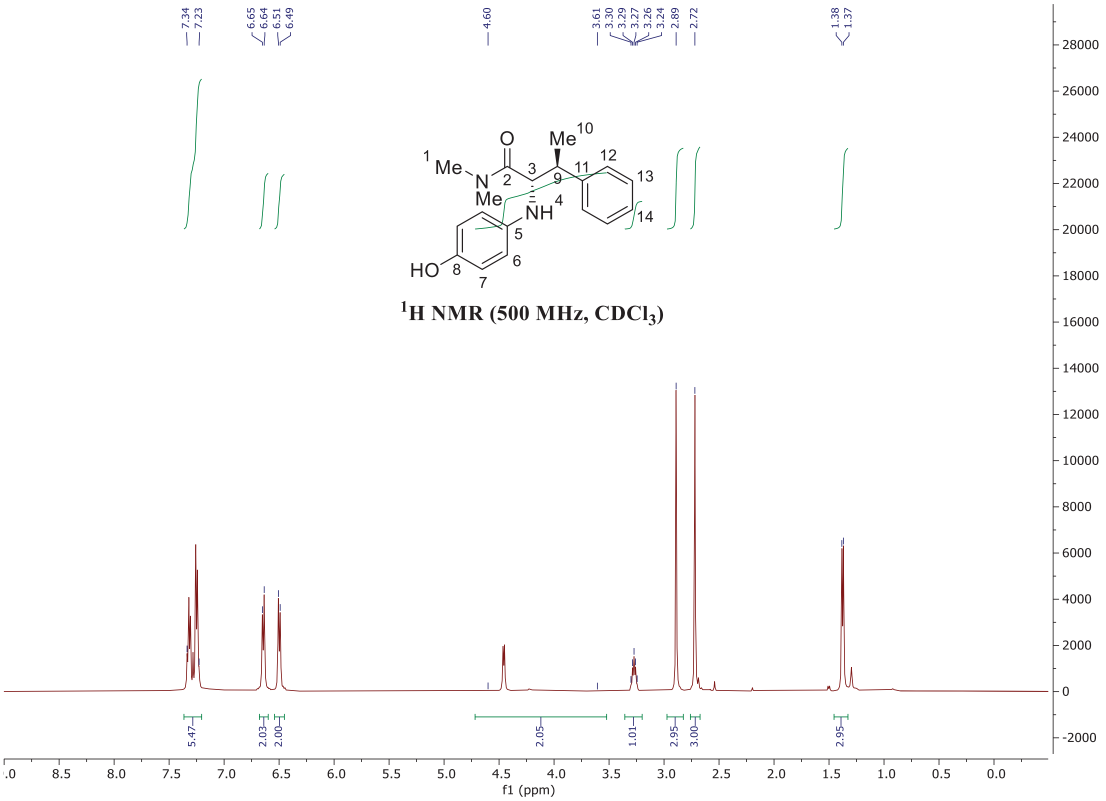

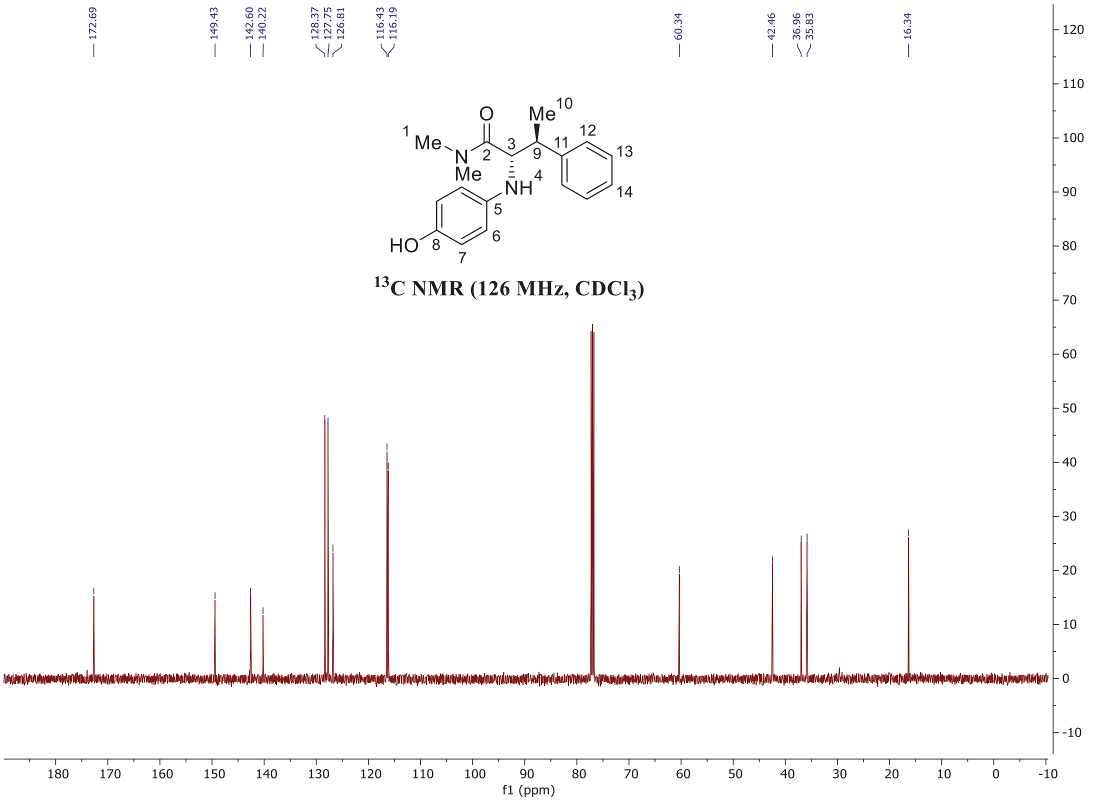

(*2S,3S*)-2-((4-Methoxyphenyl)amino)-*N*,*N*-dimethyl-3-phenylbutanamide (3qa)

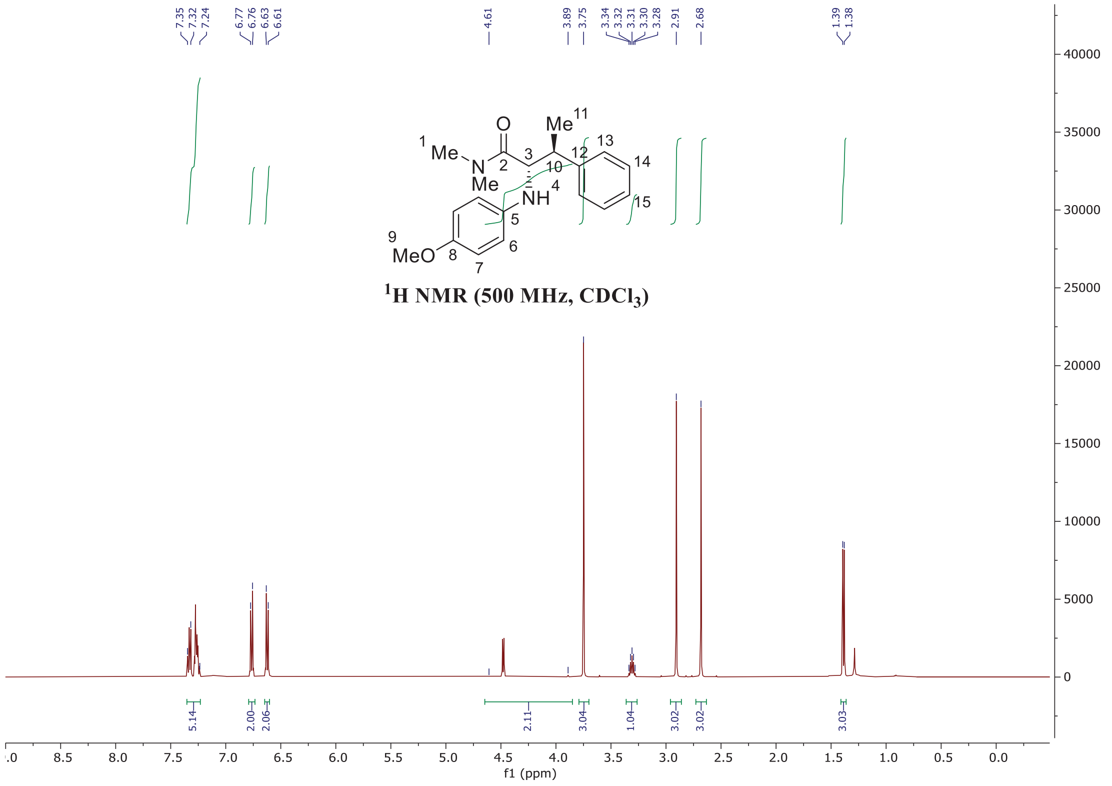

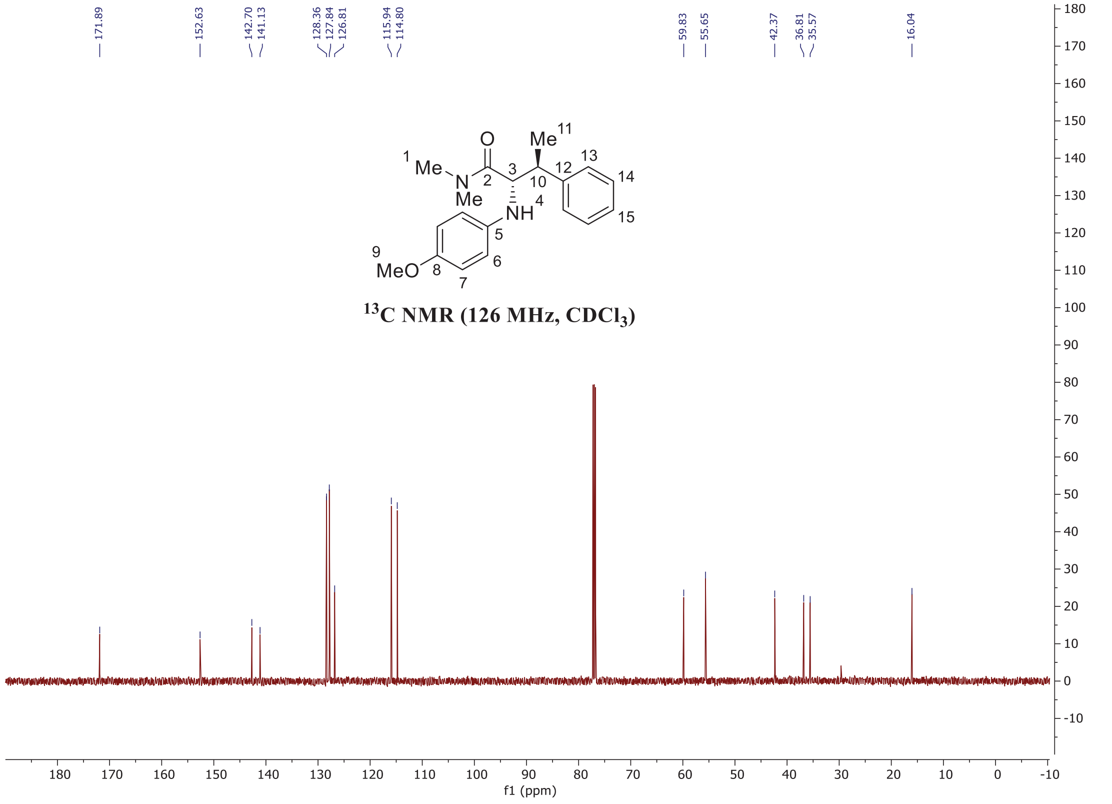

(2S,3S)-N,N-Dimethyl-3-phenyl-2-(p-tolylamino)butanamide (3ra)

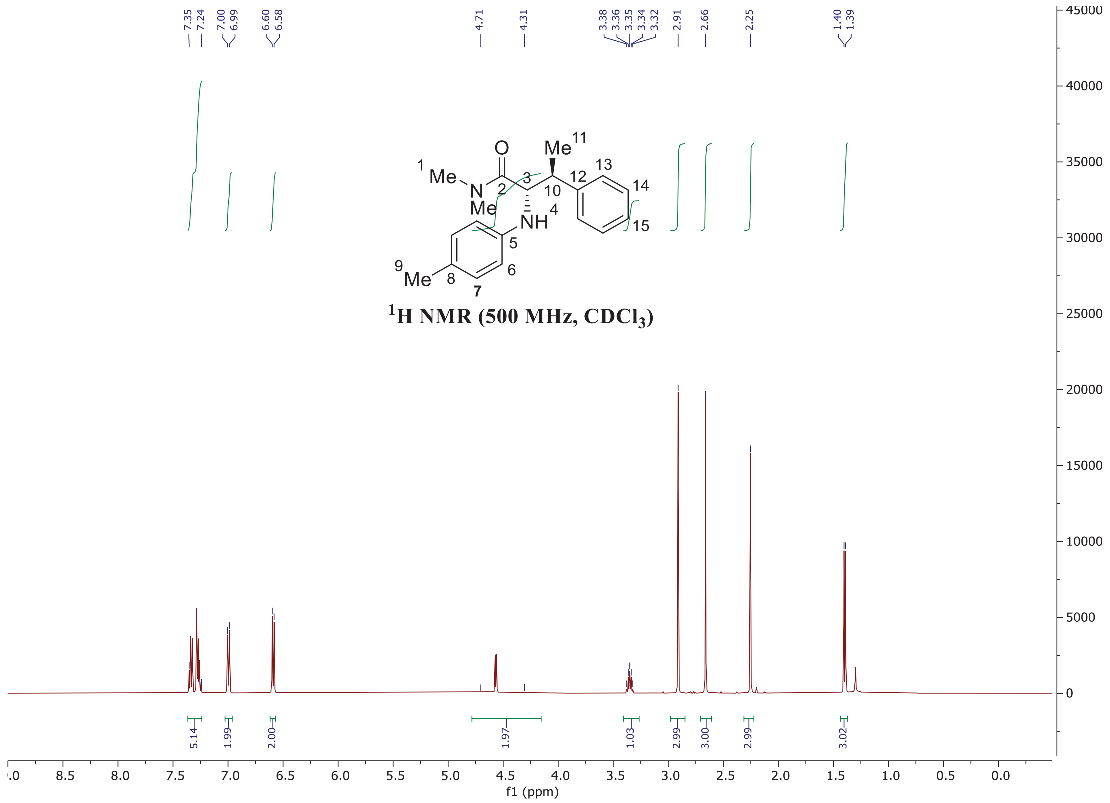

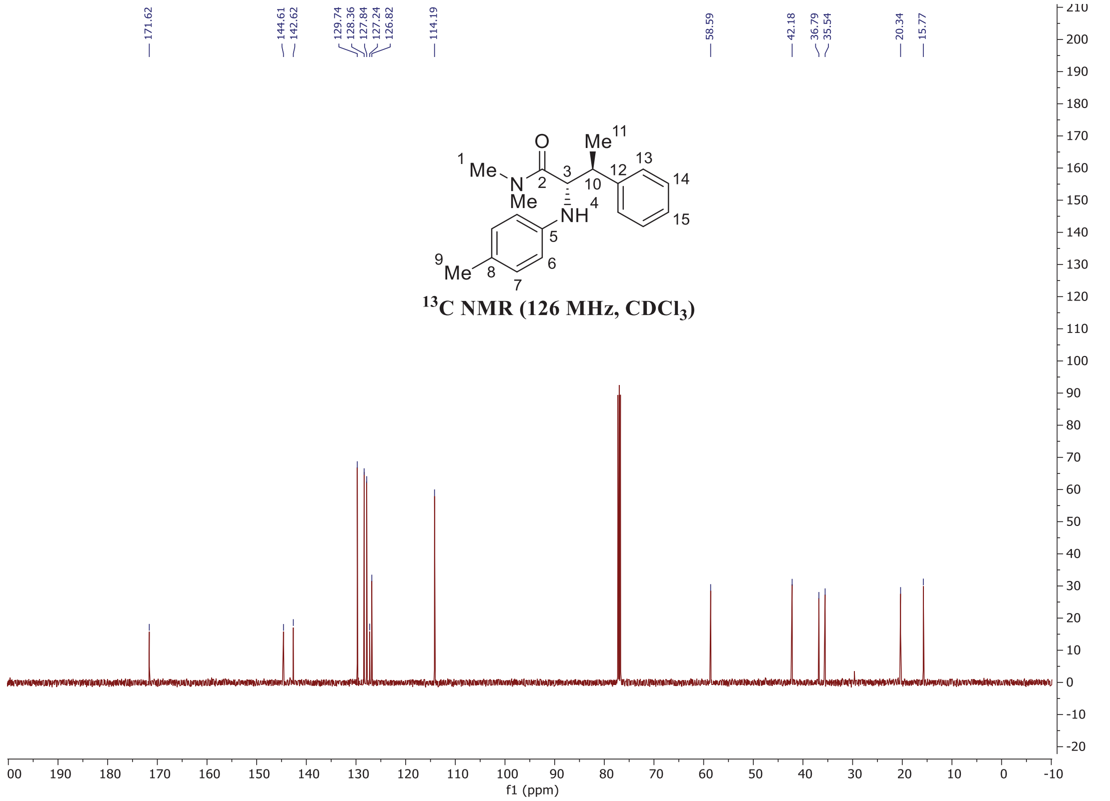

(2S,3S)-2-((4-Fluorophenyl)amino)-N,N-dimethyl-3-phenylbutanamide (3sa)

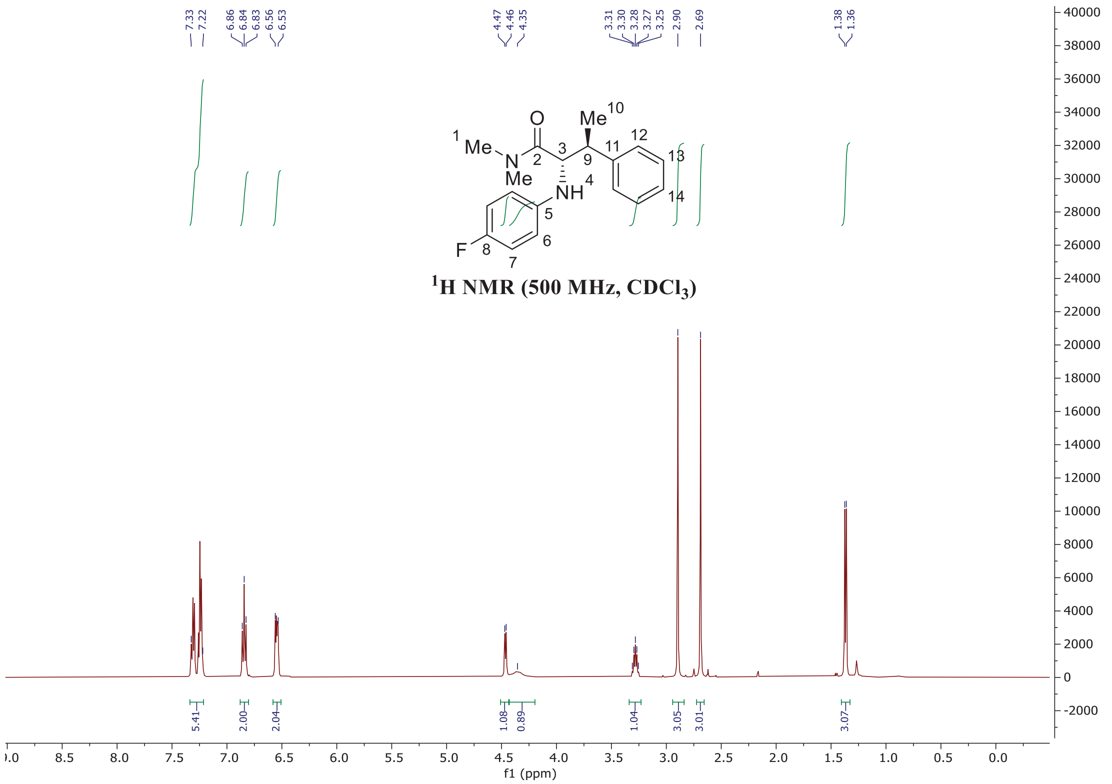

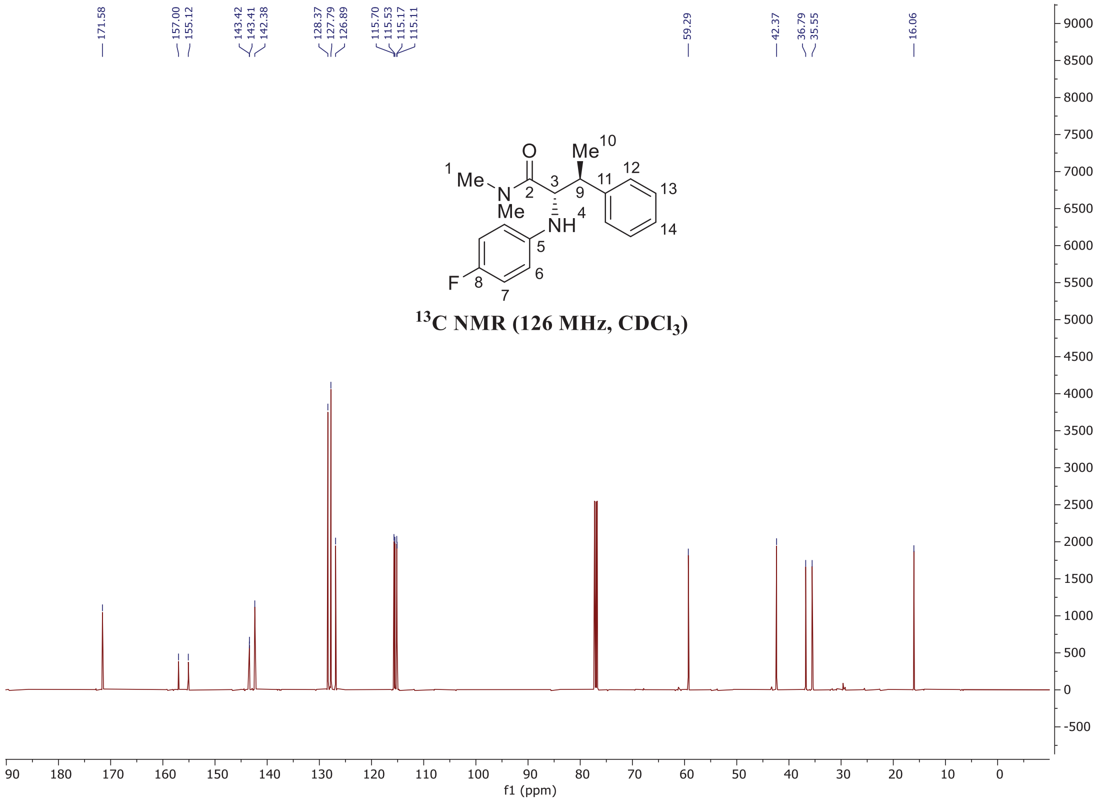

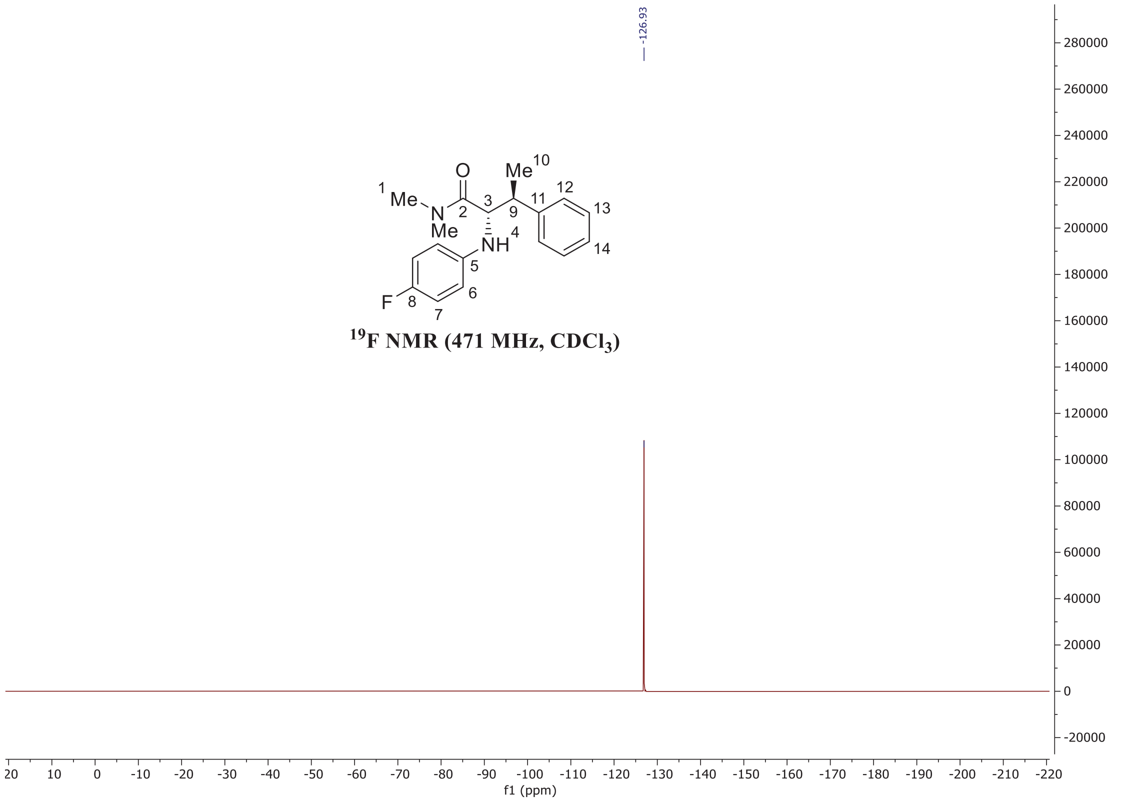

(*2S,3S*)-2-((4-Chlorophenyl)amino)-*N,N*-dimethyl-3-phenylbutanamide (3ta):

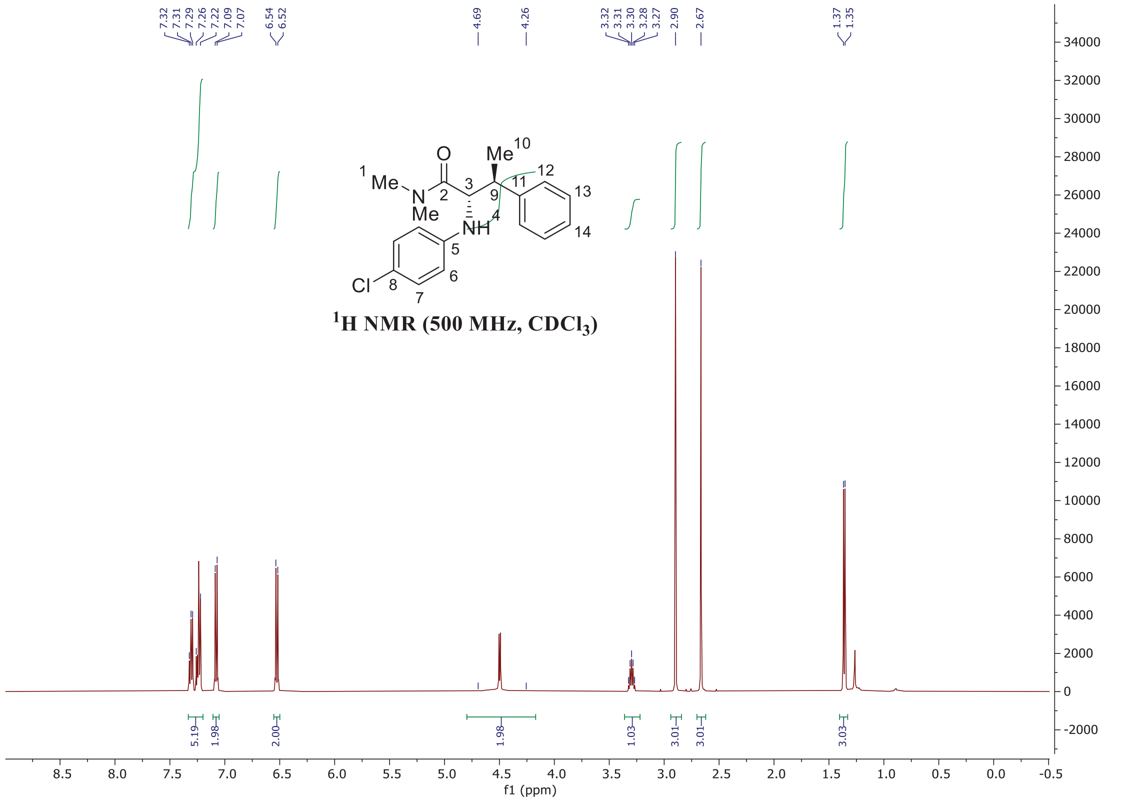

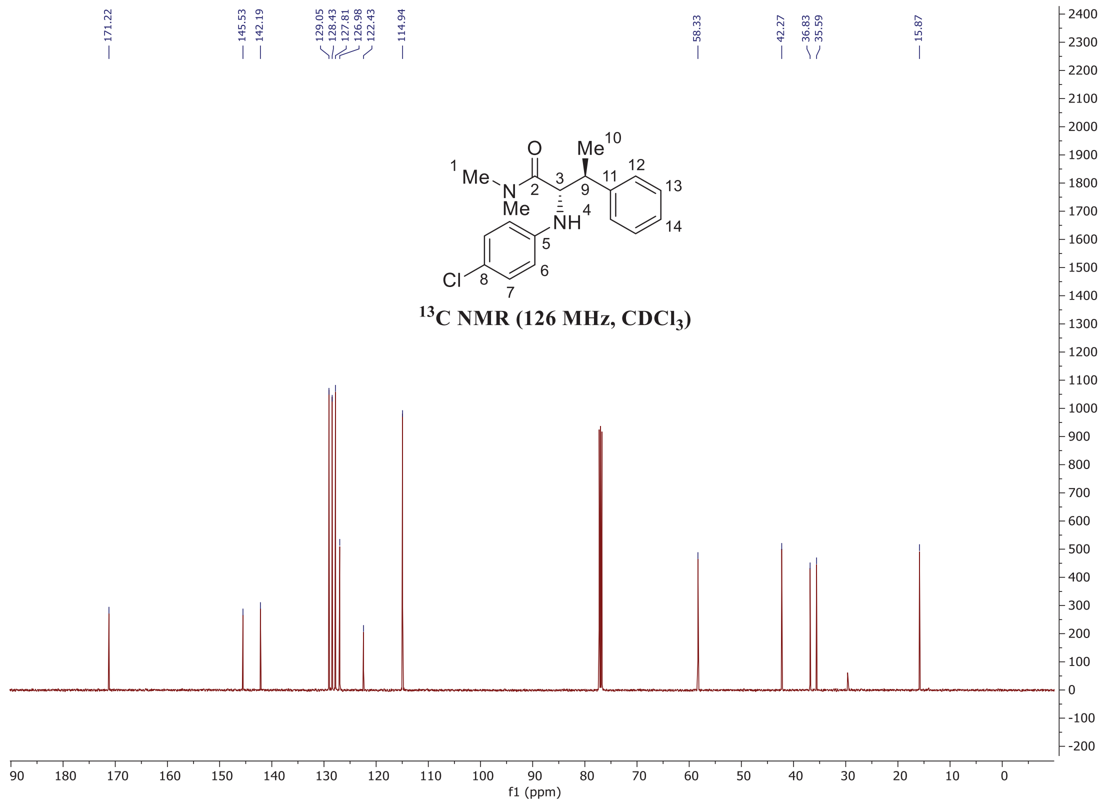

(*2S,3S*)-*N,N*-Dimethyl-3-phenyl-2-((4-(trifluoromethyl)phenyl)amino)butanamide (3ua)

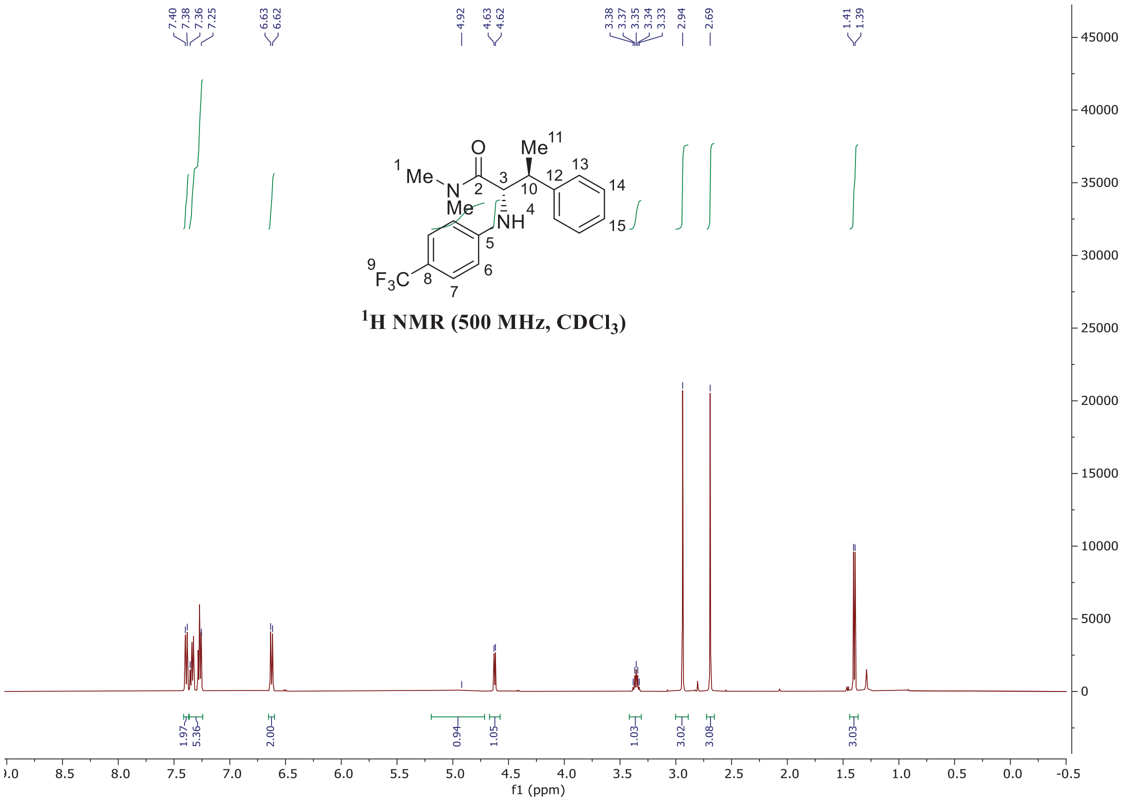

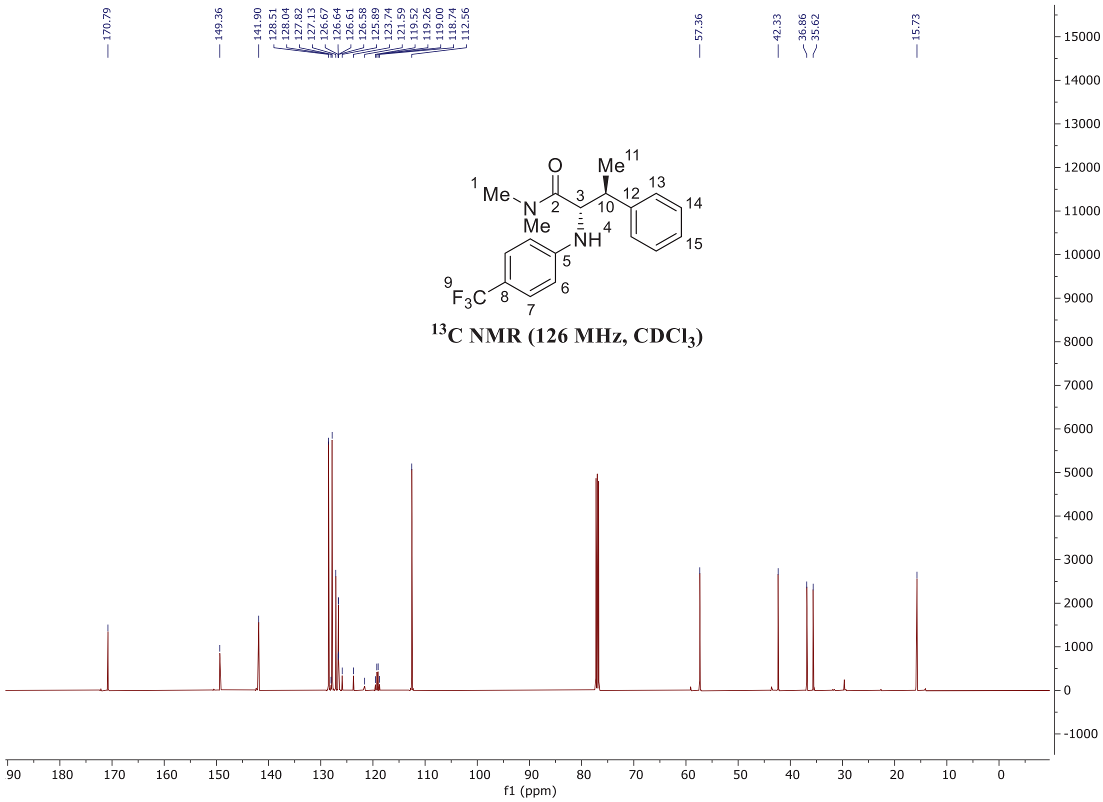
170.79
149.36
141.90
128.51
128.04
127.82
127.13
126.67
126.64
126.61
126.58
125.89
123.74
121.59
119.52
119.26
119.00
118.74
112.56
57.36
42.33
36.86
35.62
15.73
13C NMR (126 MHz, CDCl3)
f1 (ppm)

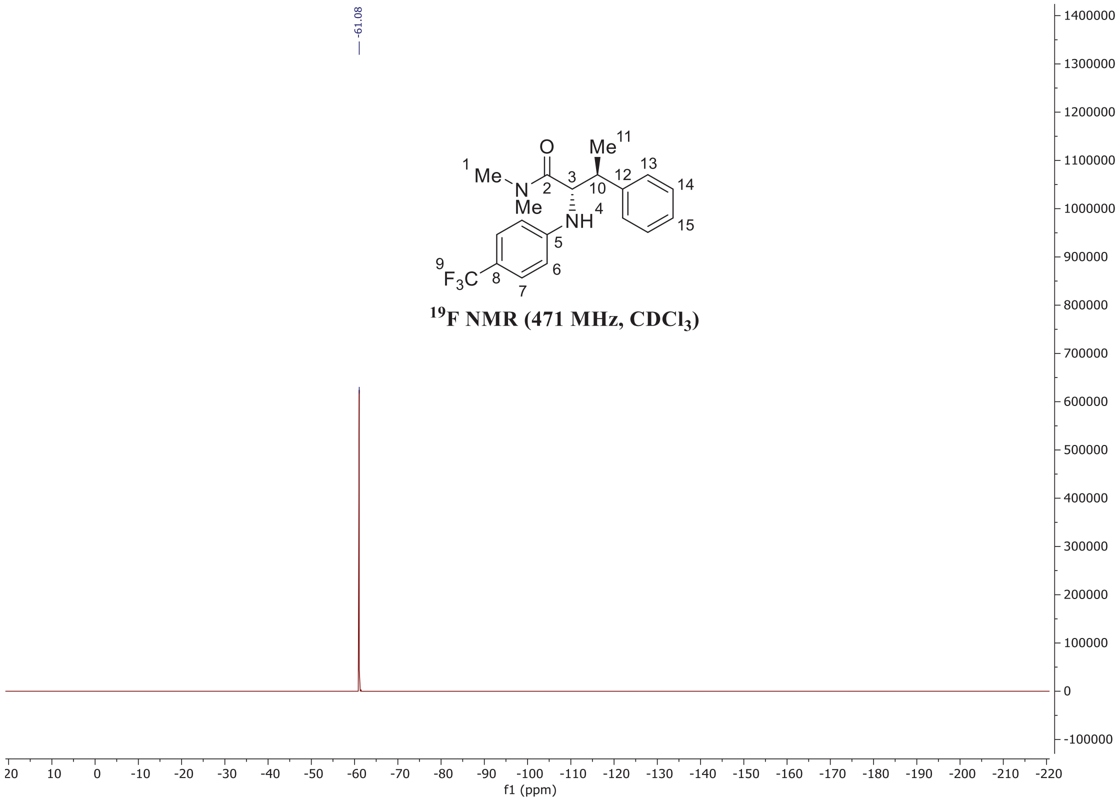
-61.08
19F NMR (471 MHz, CDCl3)
f1 (ppm)

(*2S,3S*)-2-((4-Fluoro-3-methoxyphenyl)amino)-*N,N*-dimethyl-3-phenylbutanamide (3va)

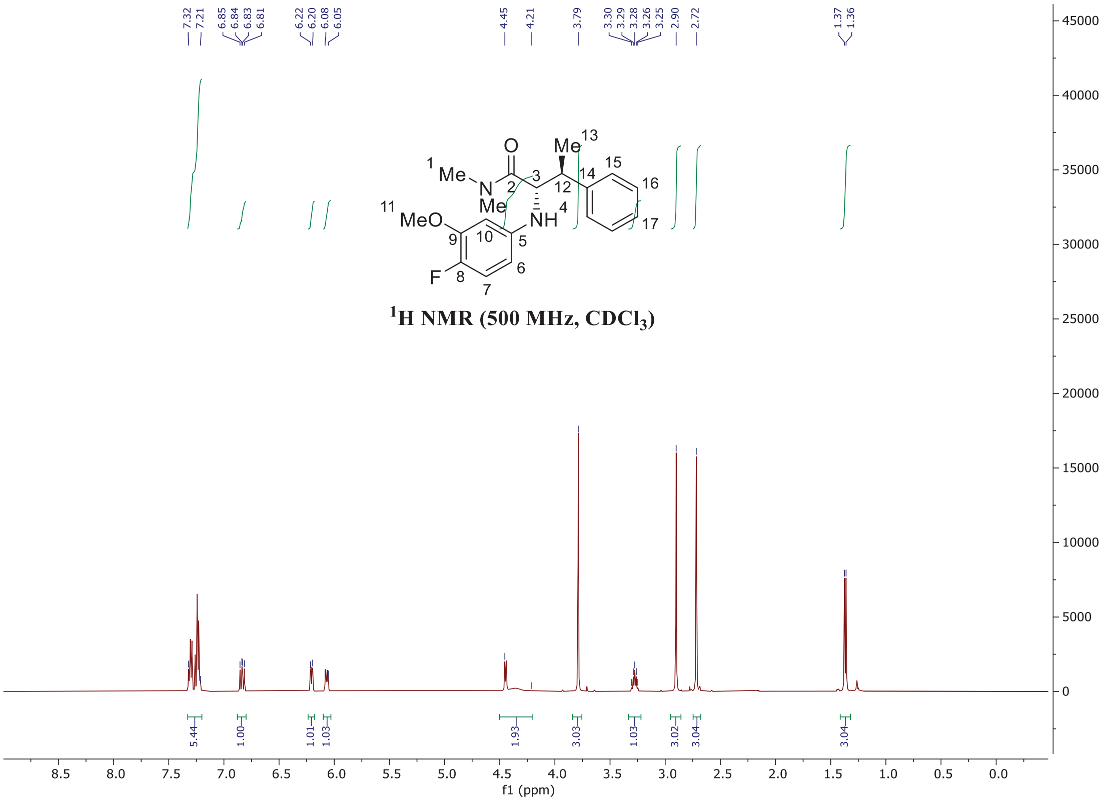

^{1}H NMR (500 MHz, $CDCl_3$)

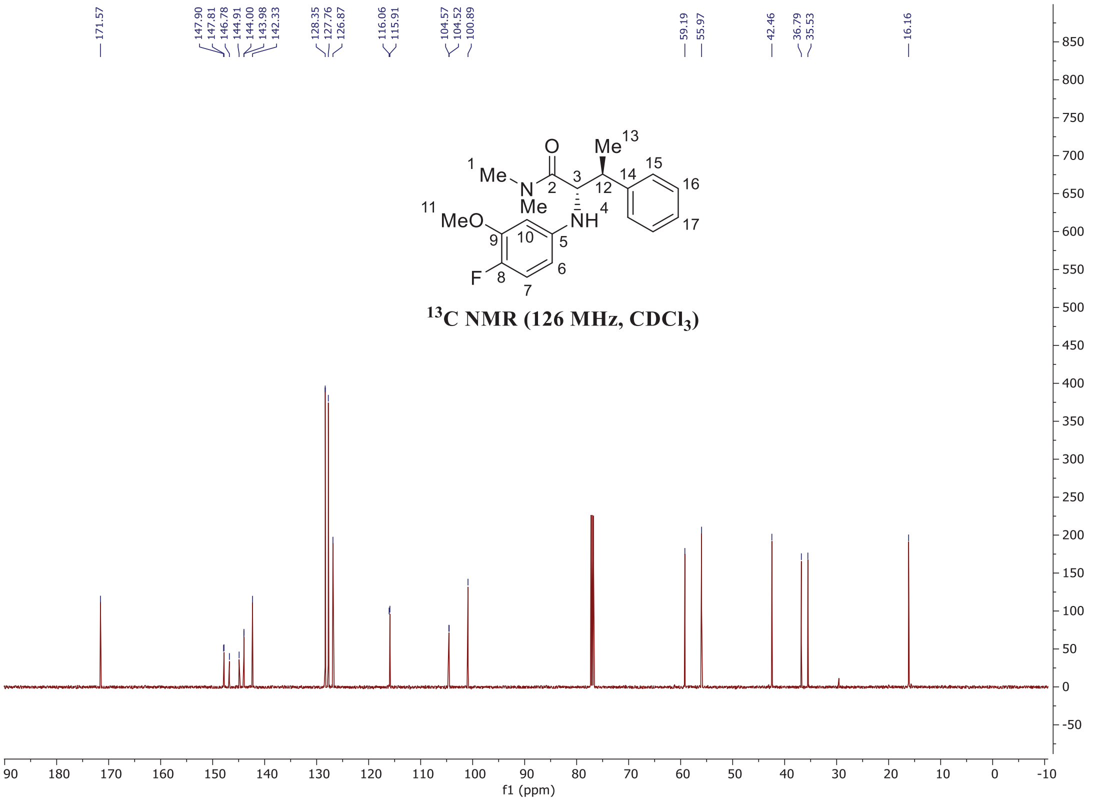

^{13}C NMR (126 MHz, $CDCl_3$)

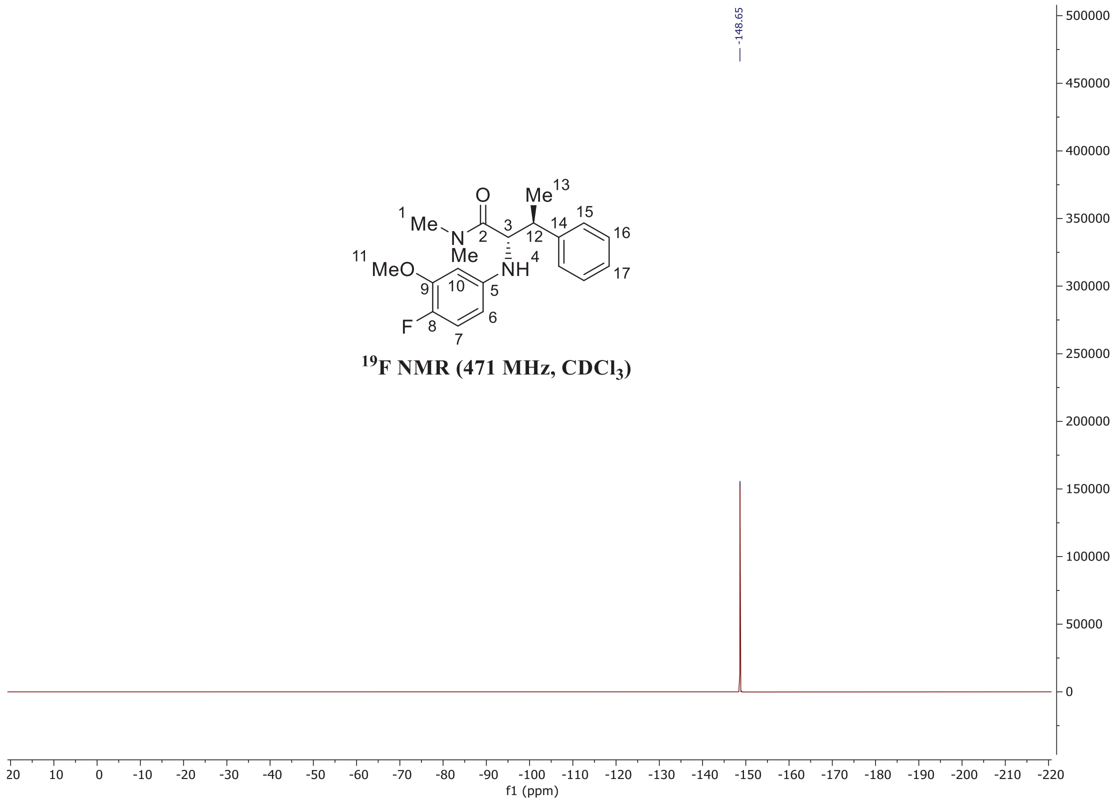

(*2S,3S*)-2-((4-Hydroxyphenyl)amino)-1-morpholino-3-phenylbutan-1-one (3wa)

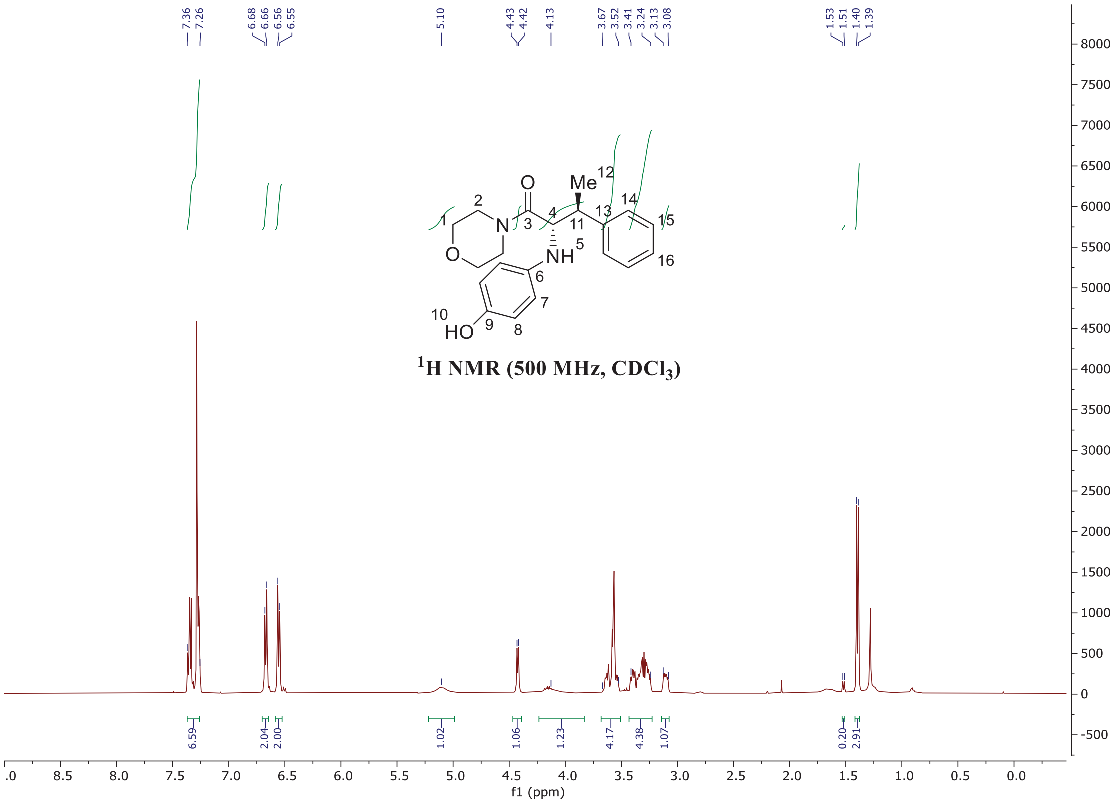

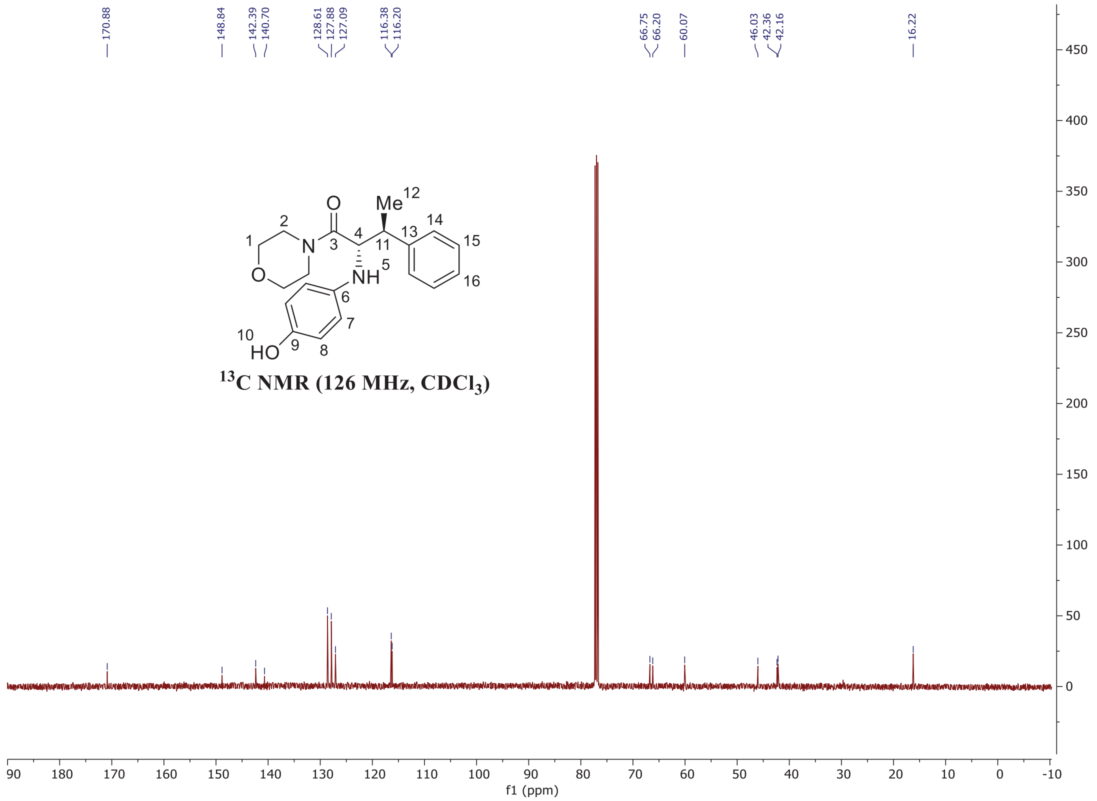

(*2S,3S*)-*N,N*-Dimethyl-2-(phenylamino)-3-(*p*-tolyl)butanamide (3fb)

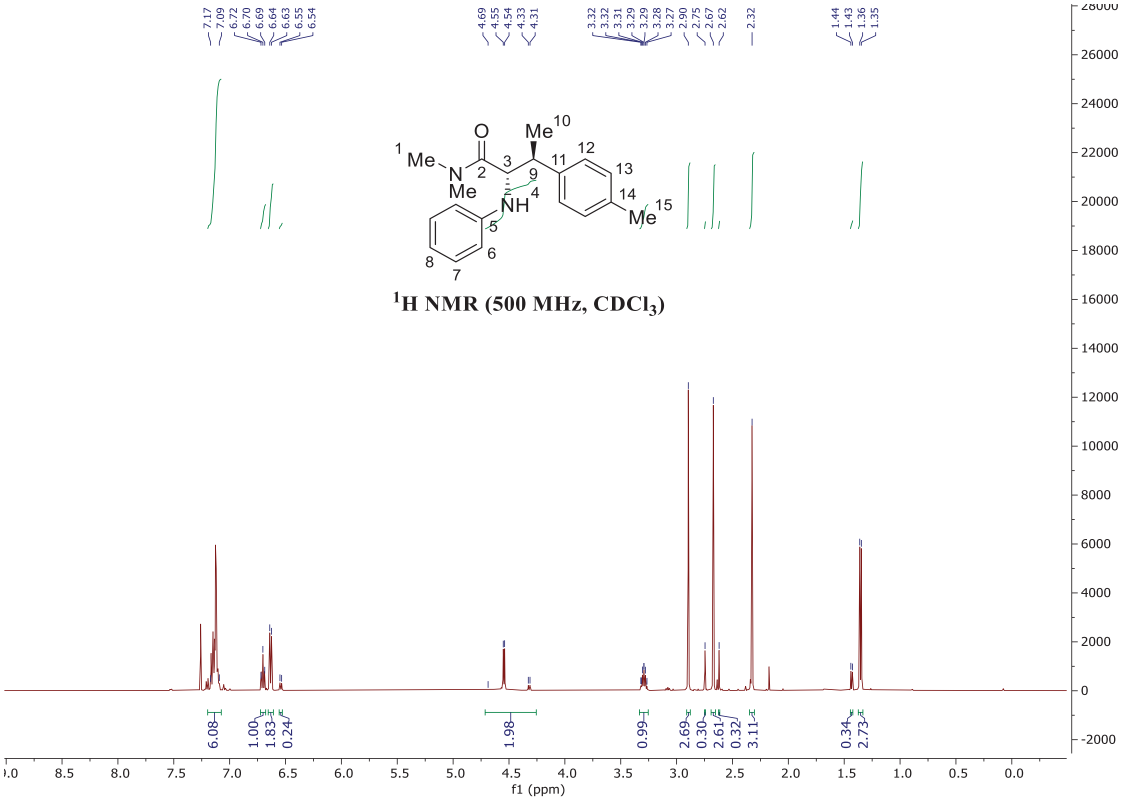

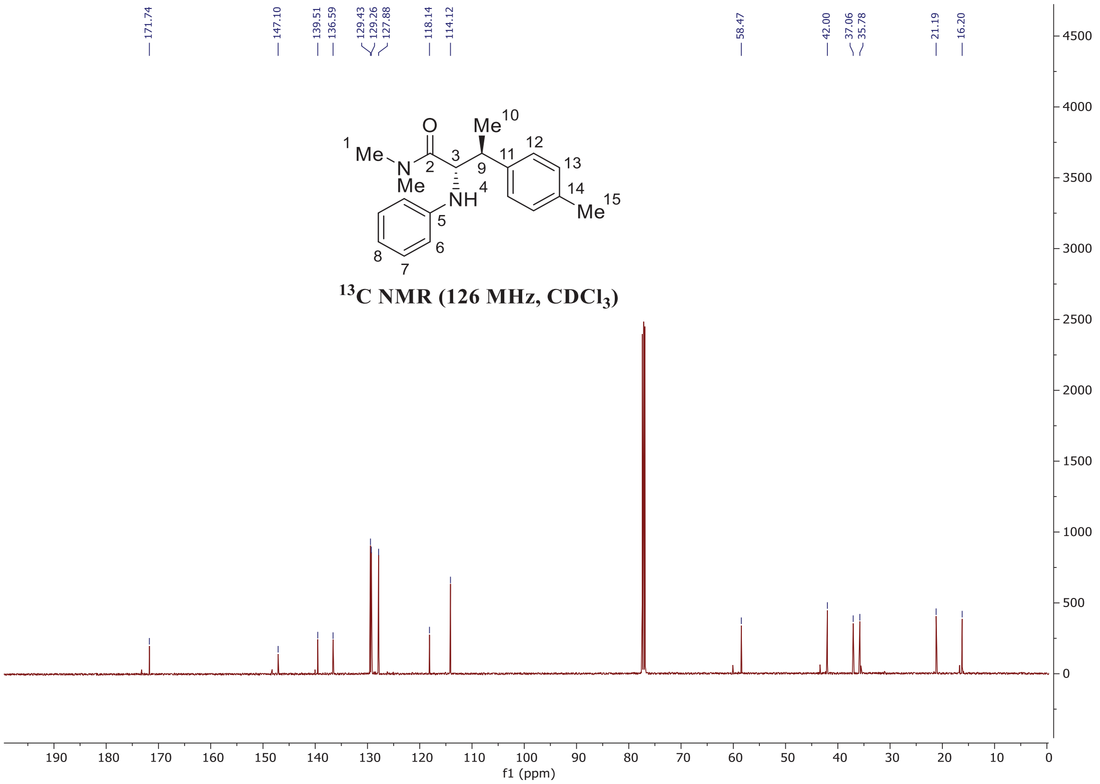

(2S,3S)-3-(4-Methoxyphenyl)-N,N-dimethyl-2-(phenylamino)butanamide (3fc)

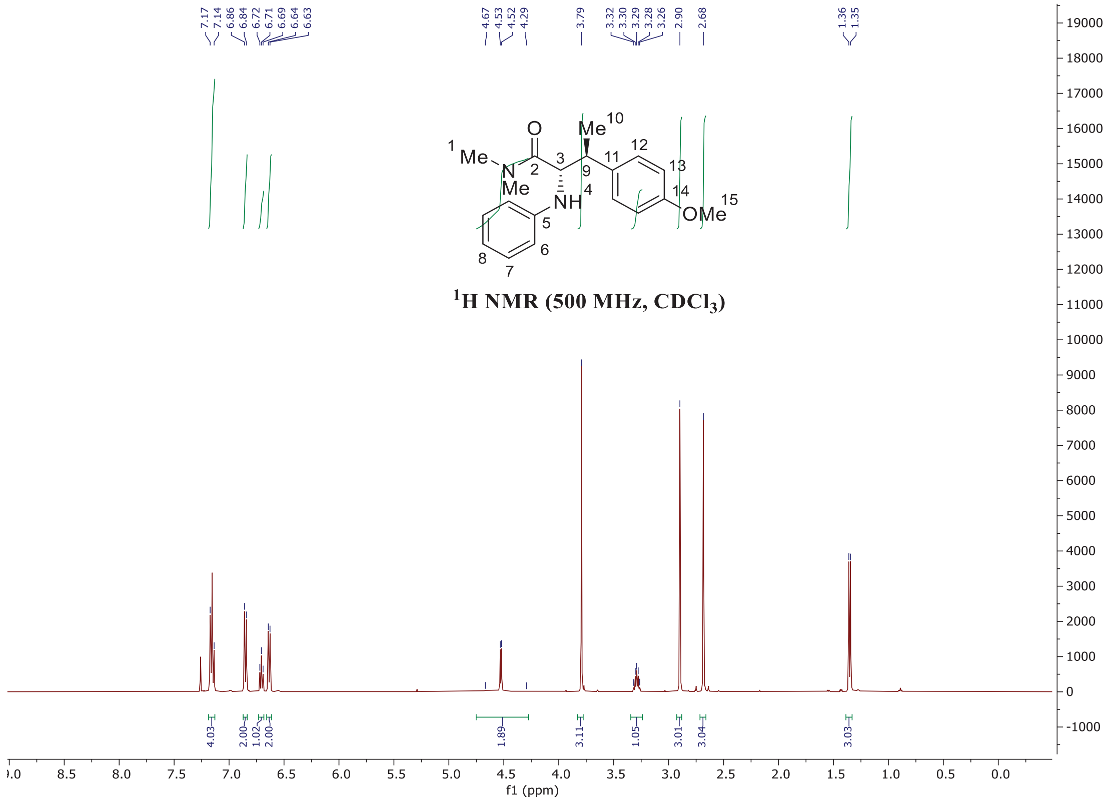

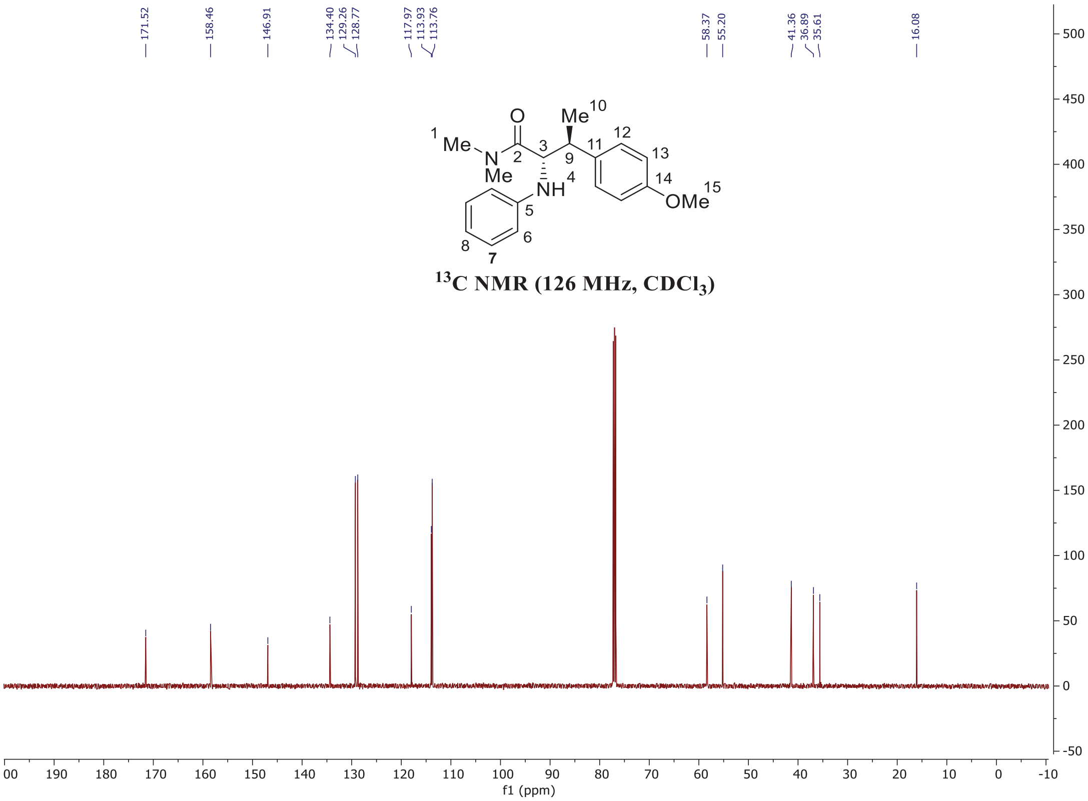

(*2S,3S*)-3-(4-(*N,N*-Diethylsulfamoyl)phenyl)-*N,N*-dimethyl-2-(phenylamino)butanamide (3fd):

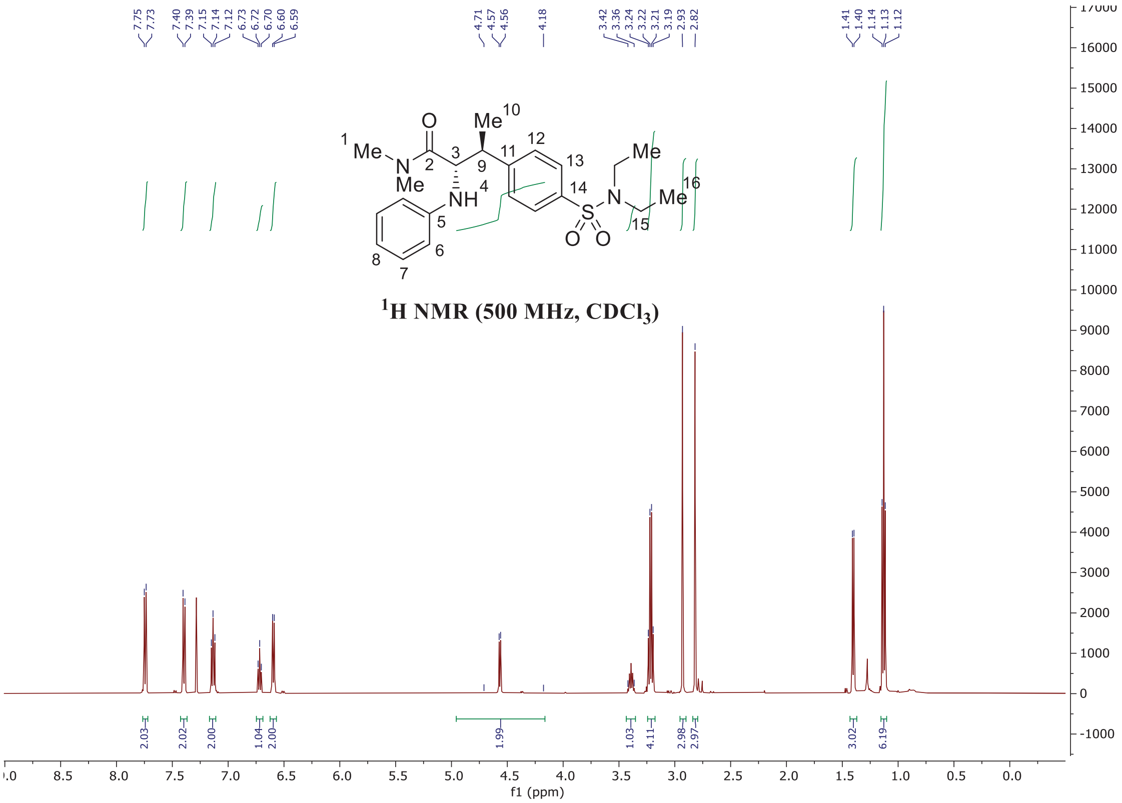

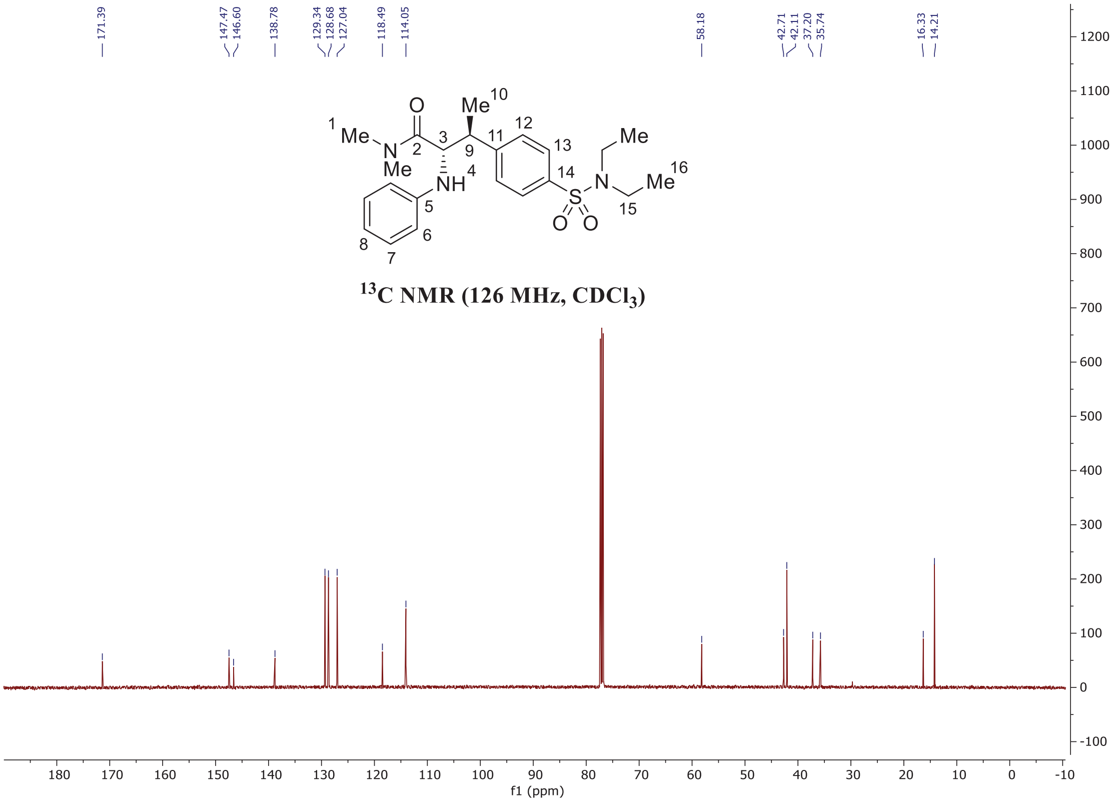

(2S,3S)-N,N-Dimethyl-3-(naphthalen-2-yl)-2-(phenylamino)butanamide (3fe)

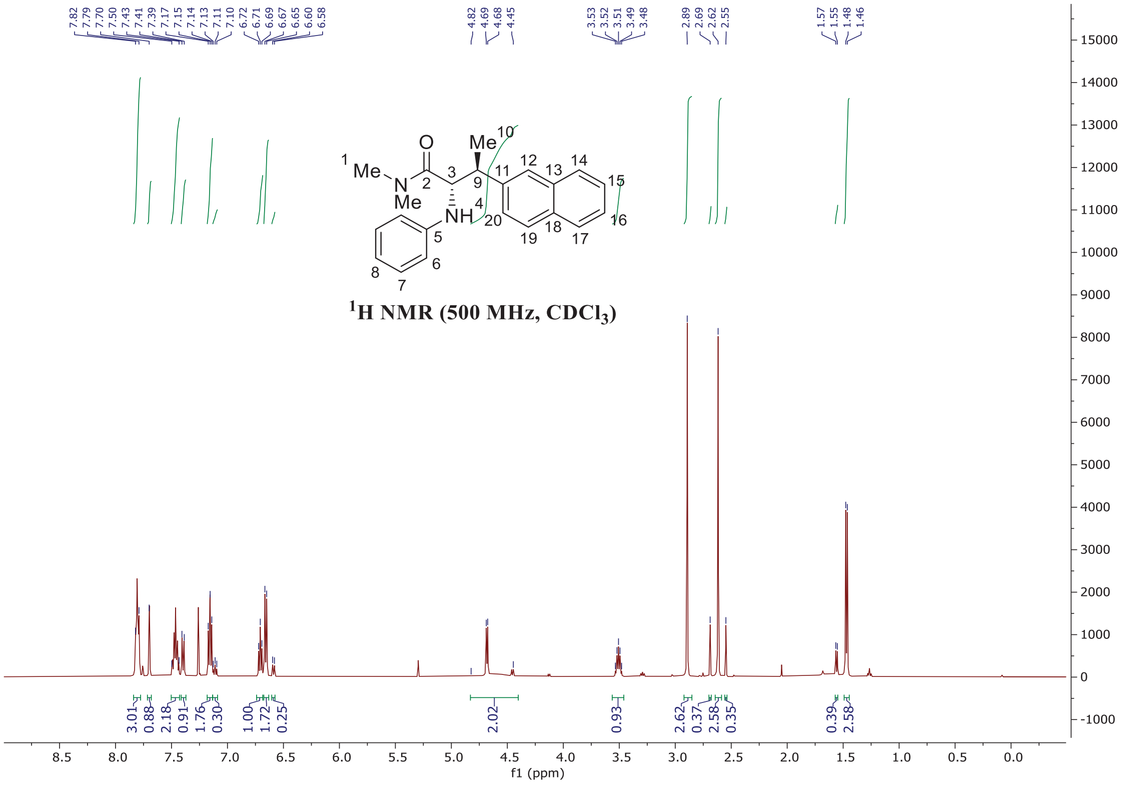

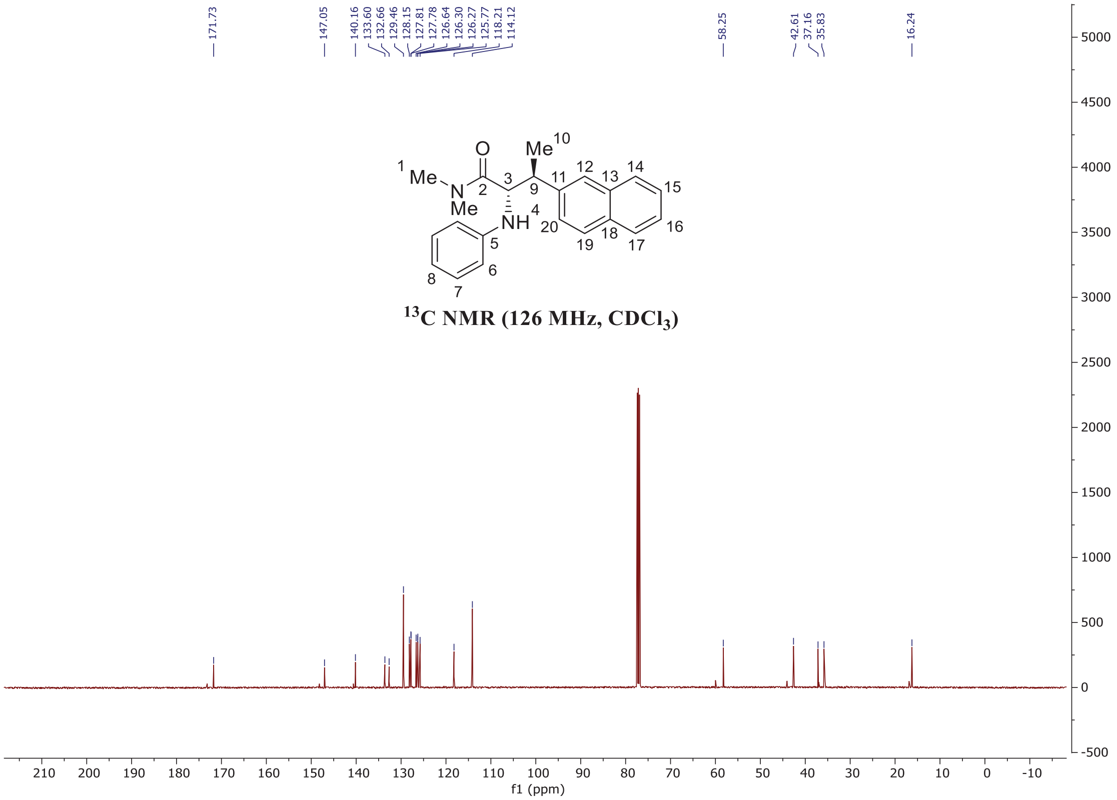

(*2S,3S*)-*N,N*-Dimethyl-3-(1-(methyl-(λ[1]-oxidaneyl)-(*p*-tolyl)sulfinyl)-1*H*-indol-3-yl)-2-(phenylamino)butanamide (3ff)

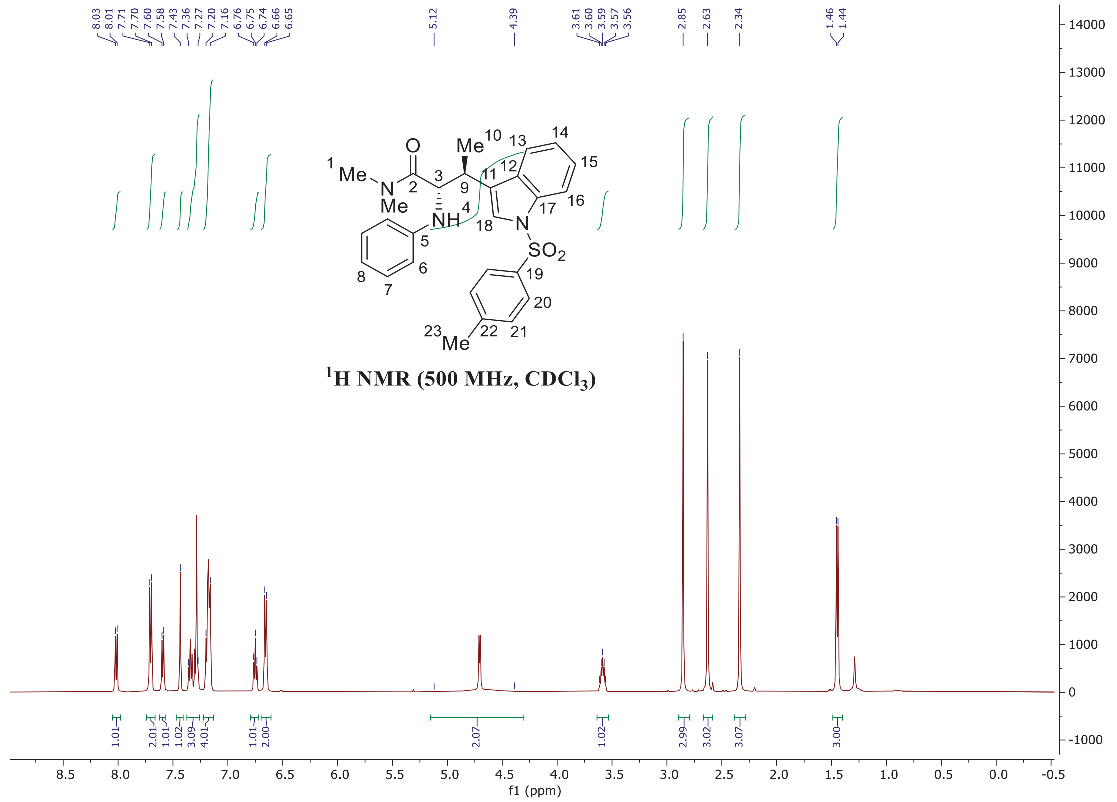

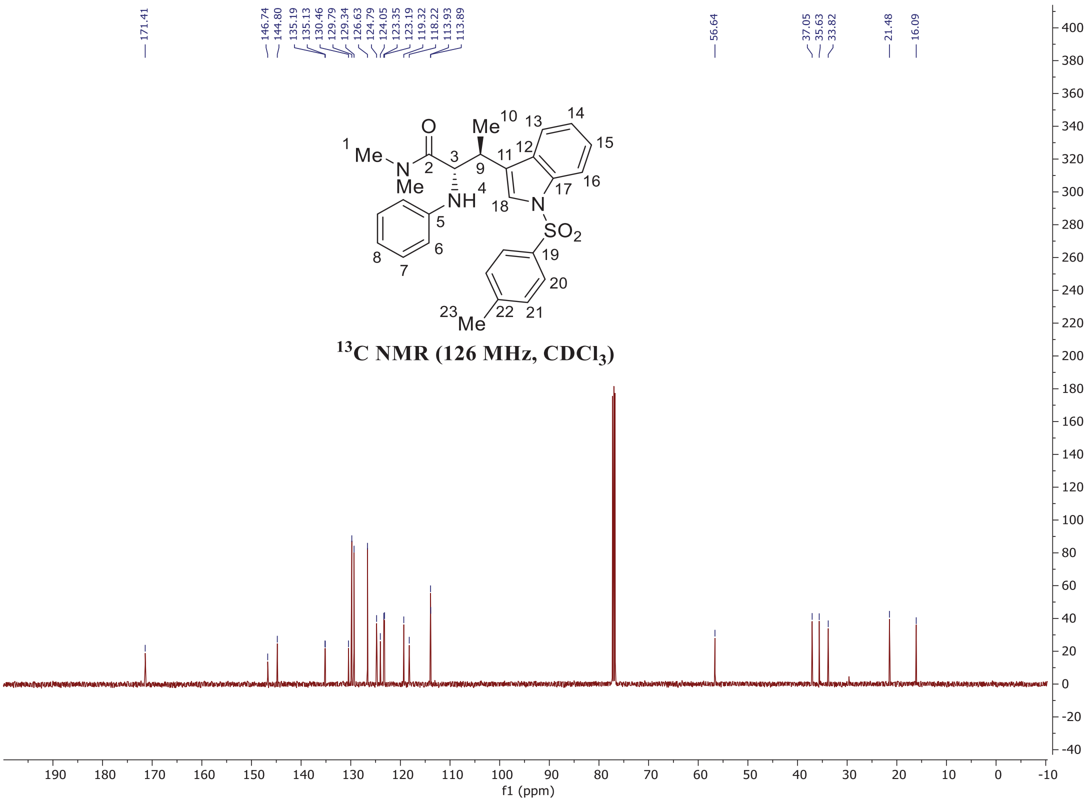

Compound (3fg)

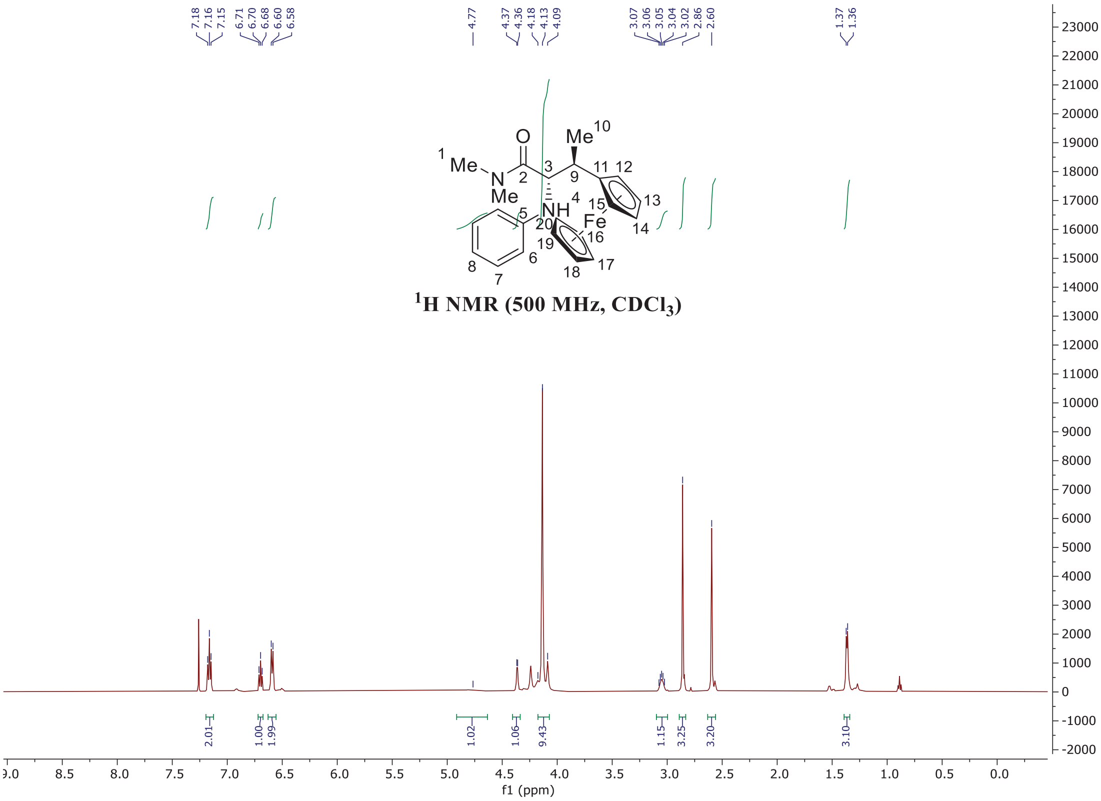

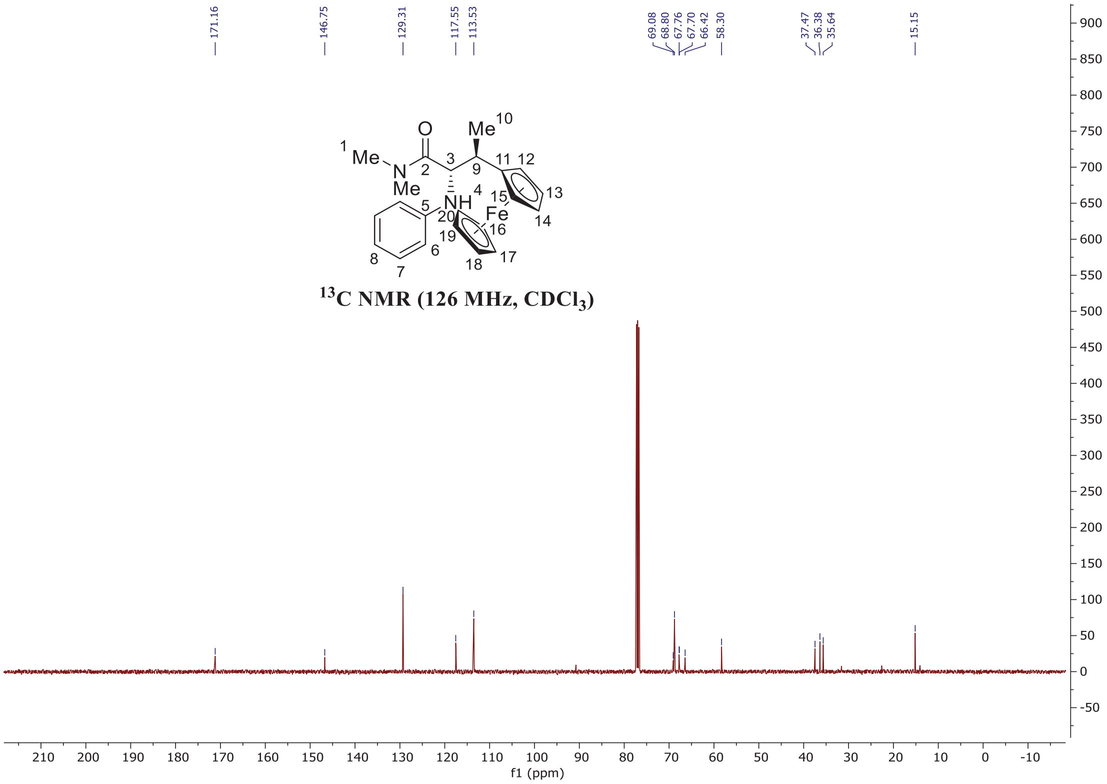

(*2S,3S*)-*N,N*-Dimethyl-2-(phenylamino)-3-(4-(4,4,5,5-tetramethyl-1,3,2-dioxaborolan-2-yl)phenyl)butanamide (3fh):

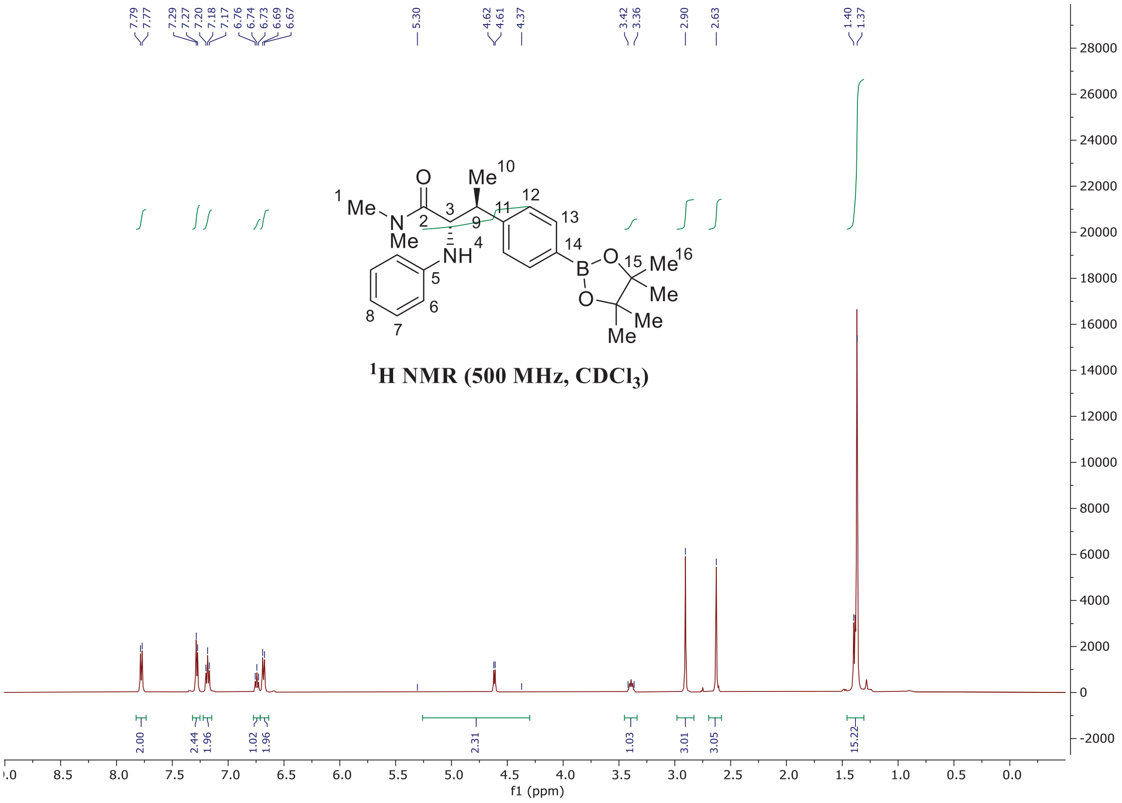

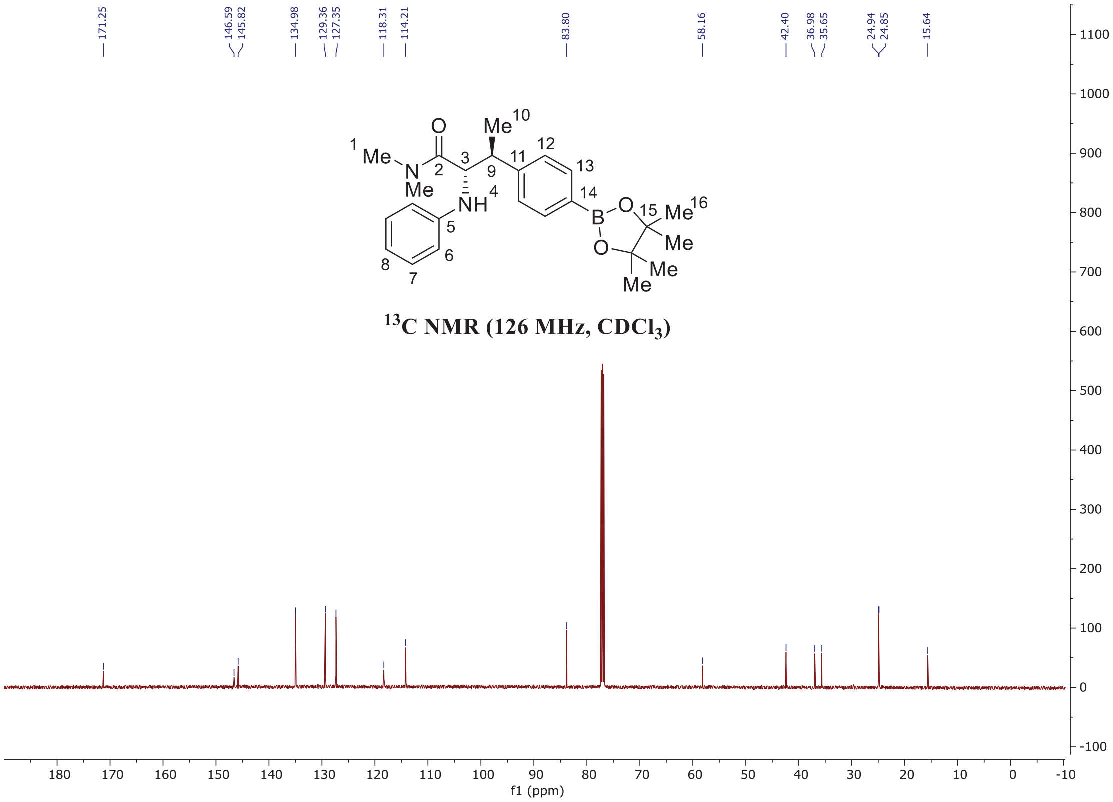

(2*S*,3*S*)-3-(4-Bromophenyl)-*N*,*N*-dimethyl-2-(phenylamino)butanamide (3fi)

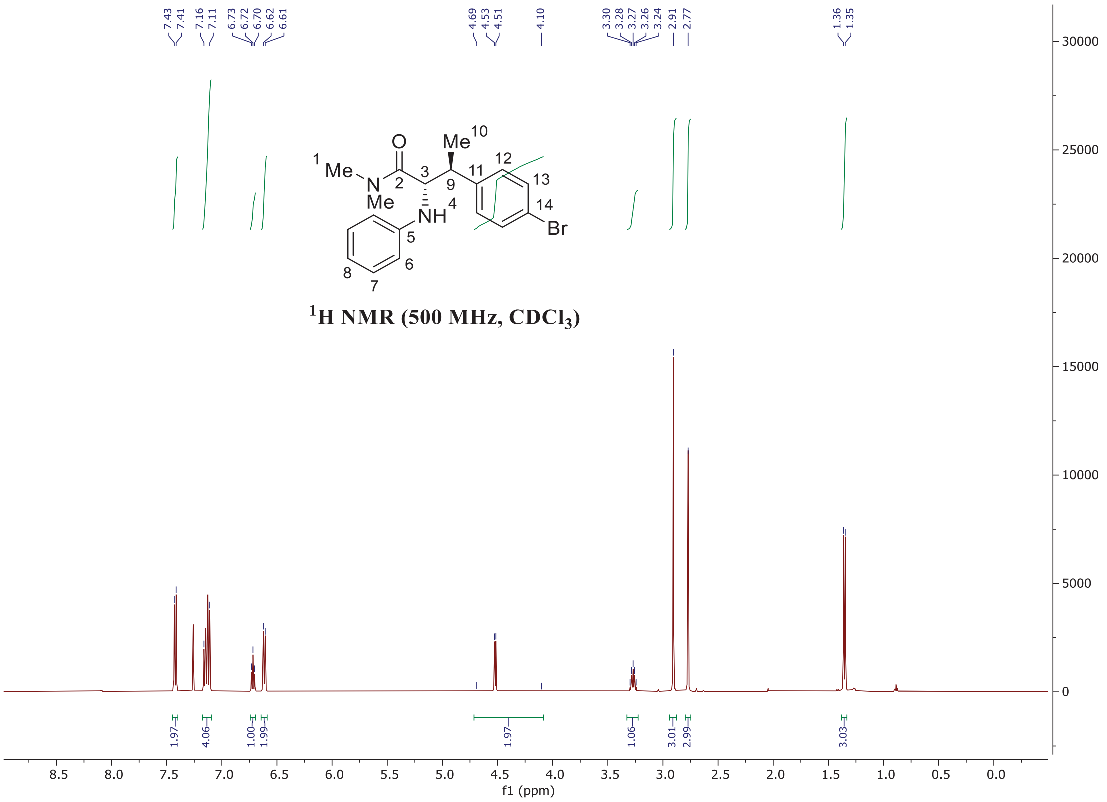

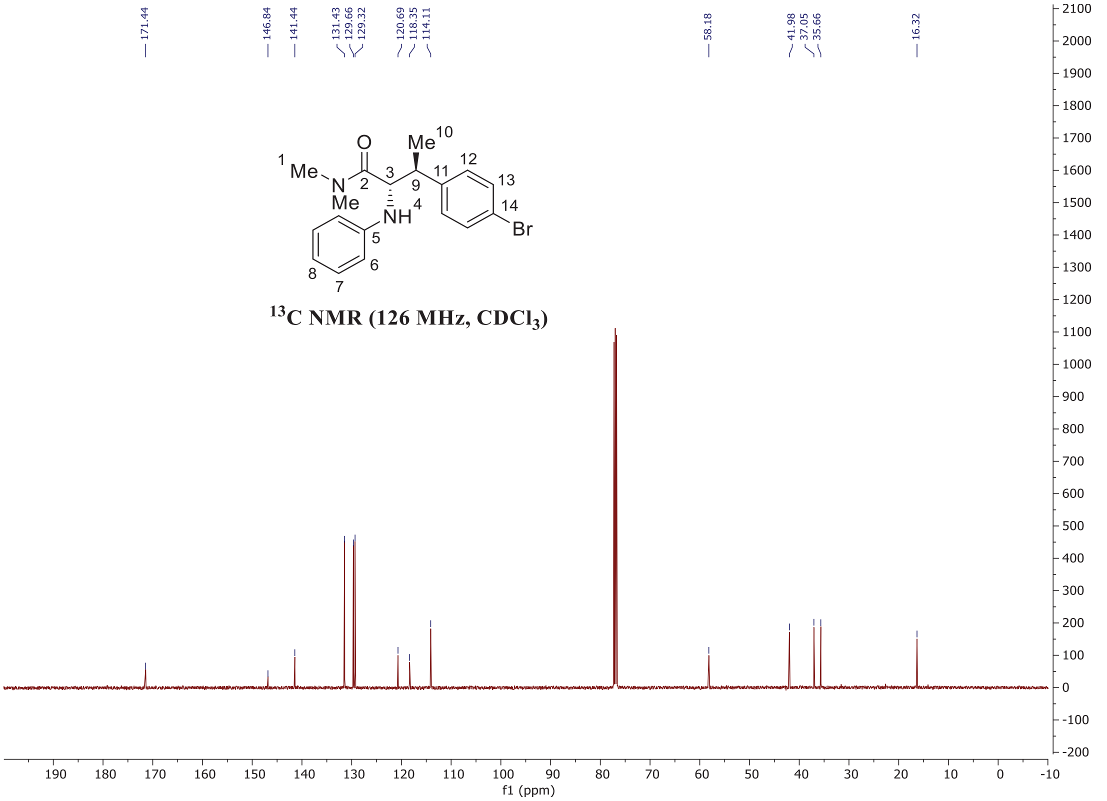

(2S,3S)-3-([1,1'-Biphenyl]-4-yl)-N,N-dimethyl-2-(phenylamino)butanamide (3fj)

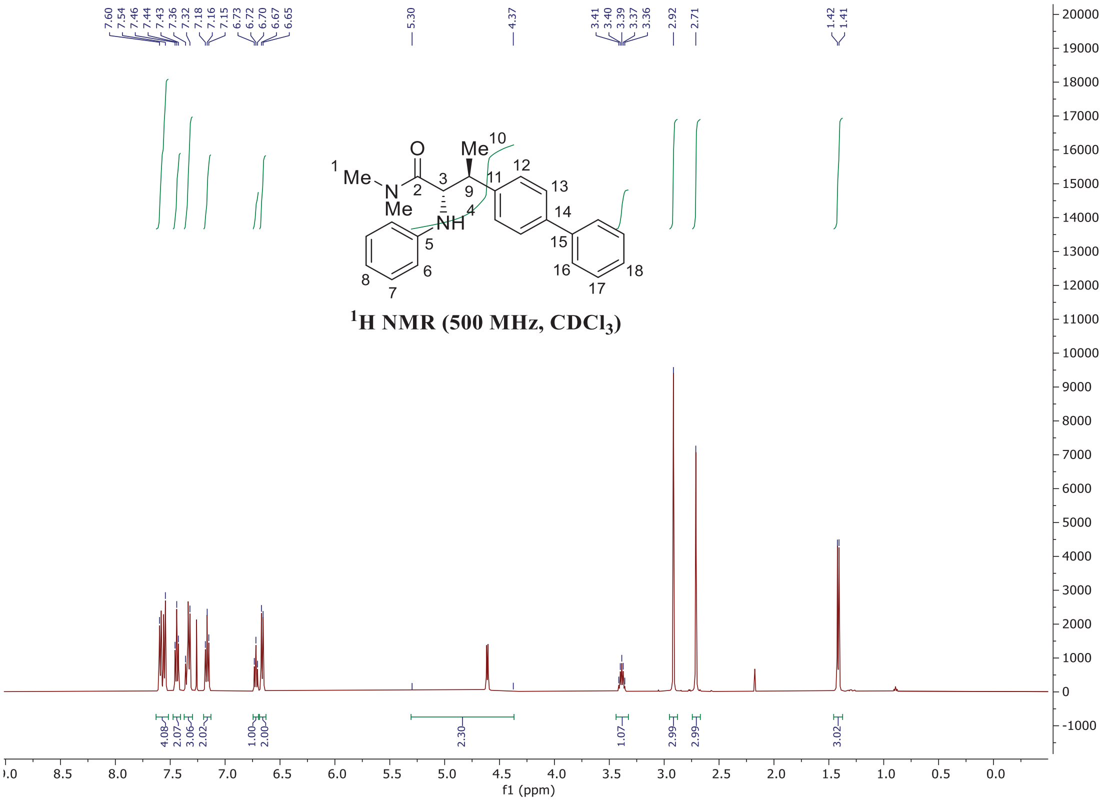

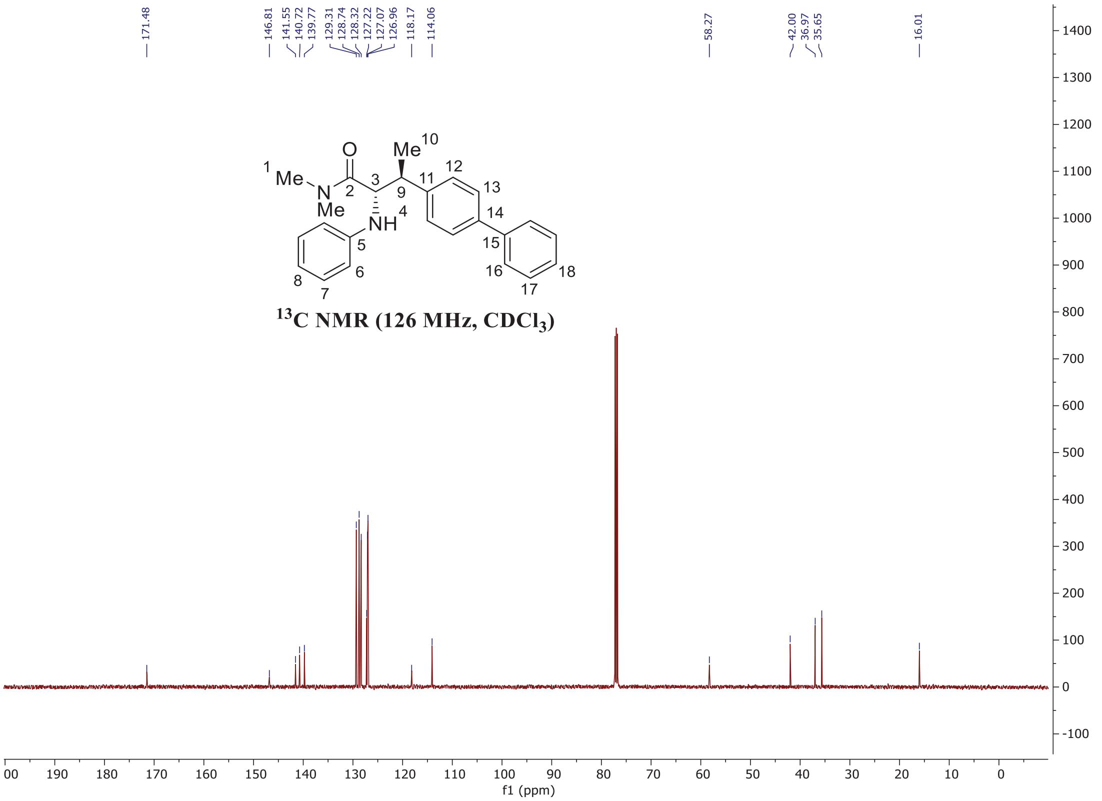

(*2S,3S*)-*N,N*-Dimethyl-2-(phenylamino)-3-(4-(trimethylsilyl)phenyl)butanamide (3fk):

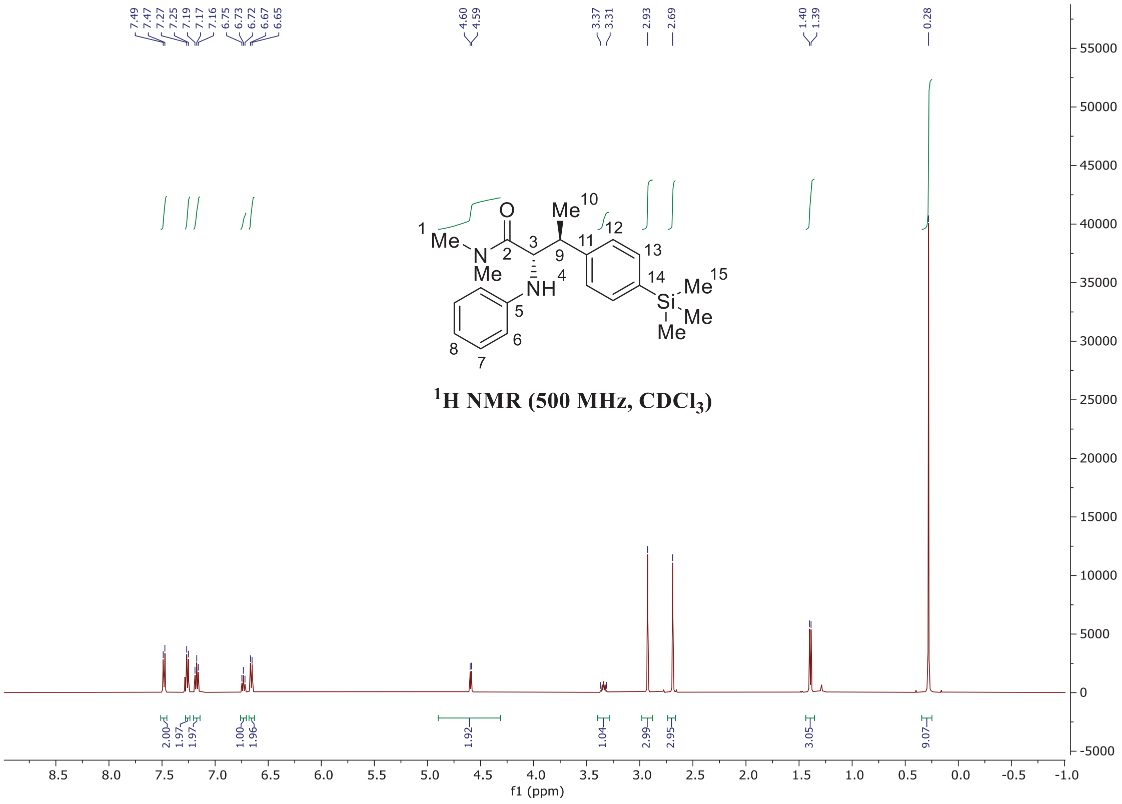

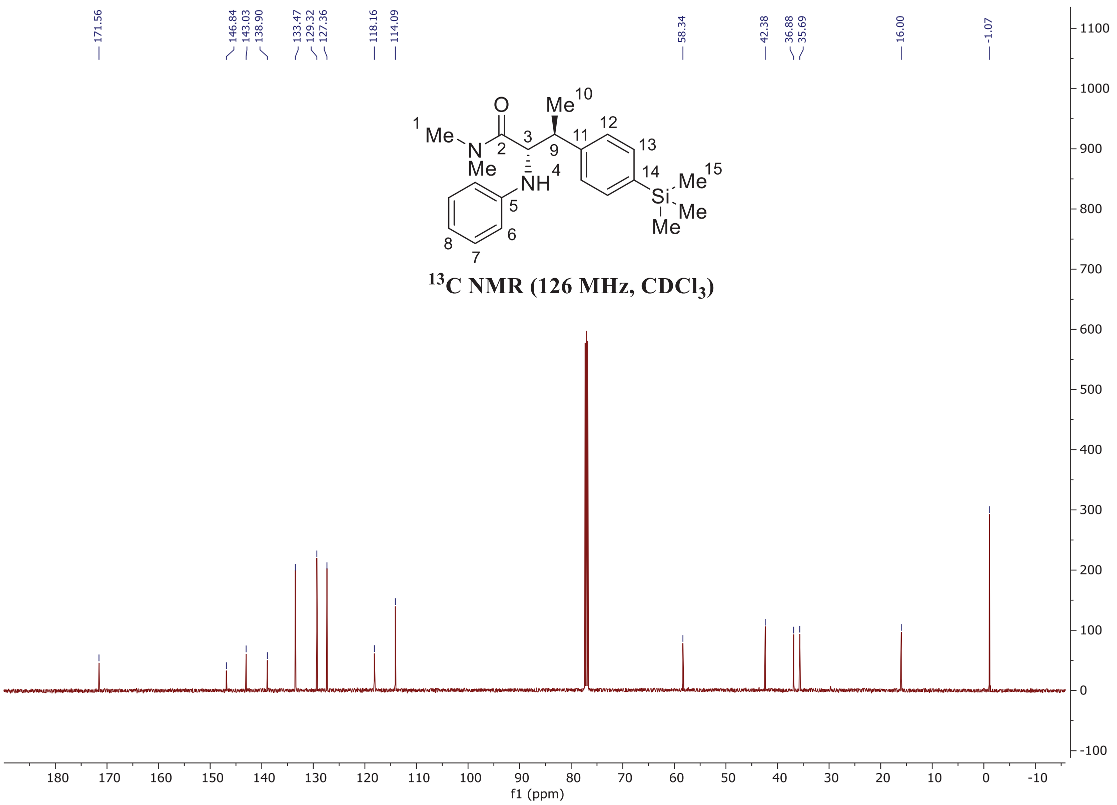

(*2S,3S*)-*N,N*-Dimethyl-2-(phenylamino)-3-(*o*-tolyl)butanamide (3fl)

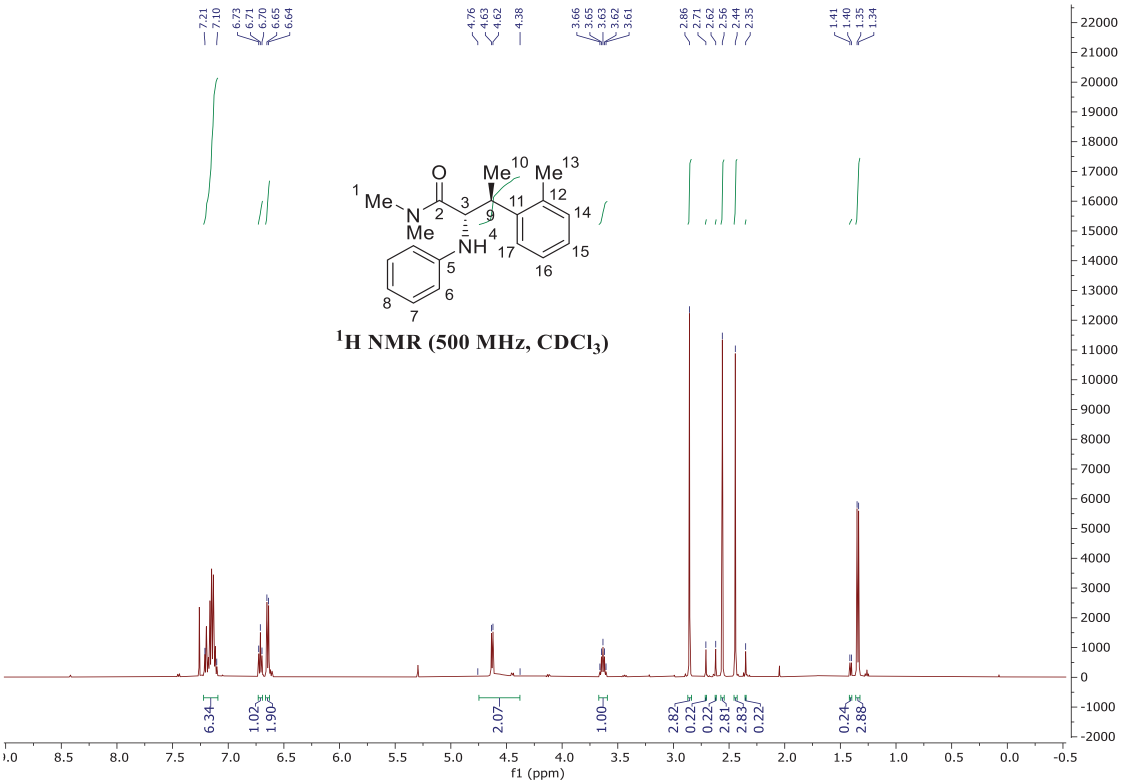

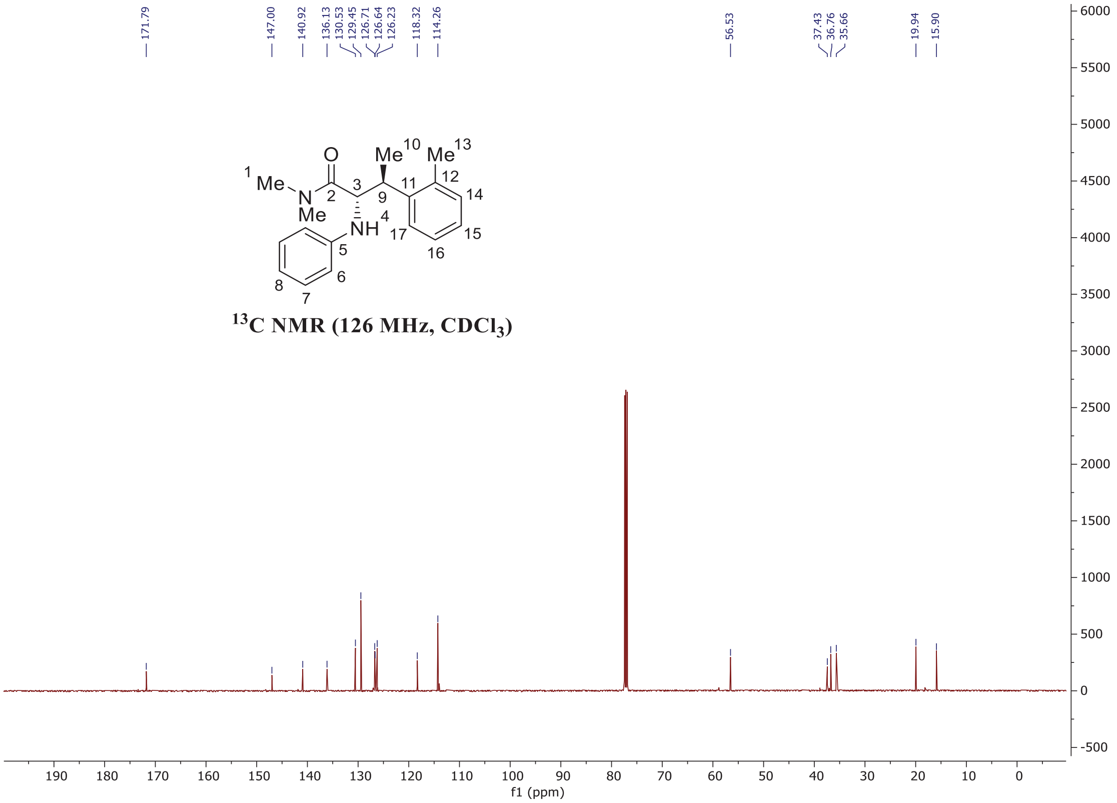

(*2S,3S*)-3-(Benzofuran-5-yl)-*N,N*-dimethyl-2-(phenylamino)butanamide (3fm):

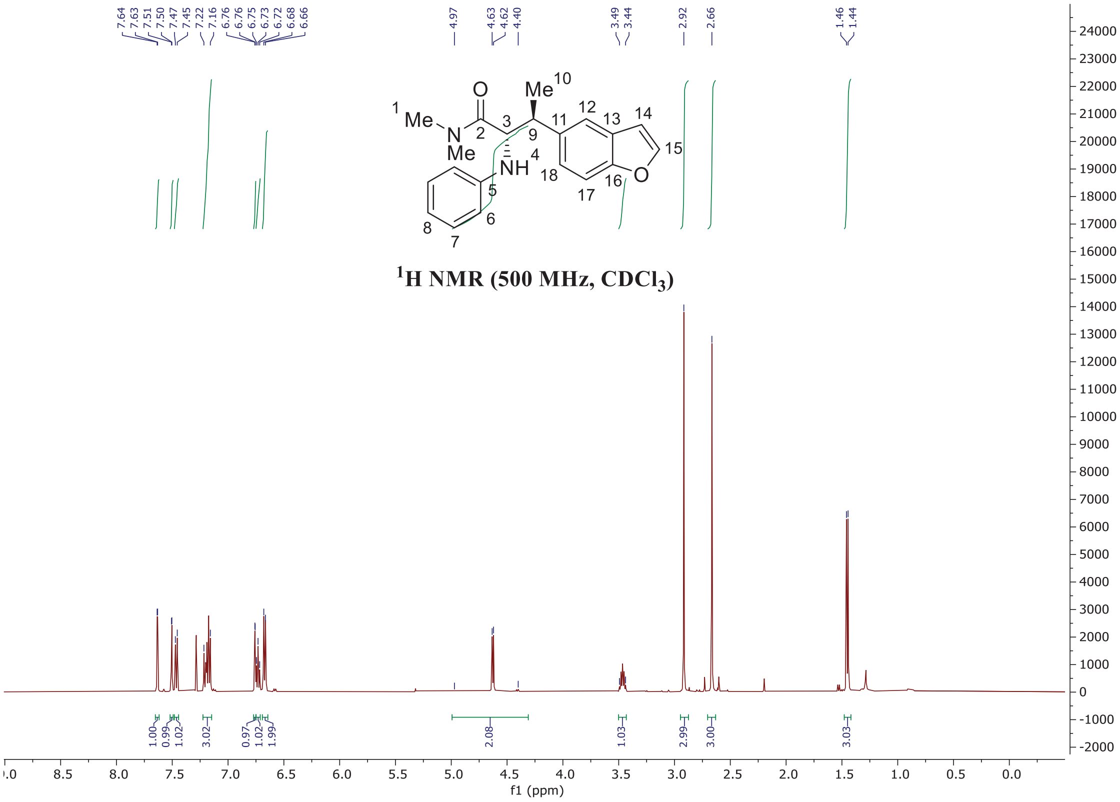

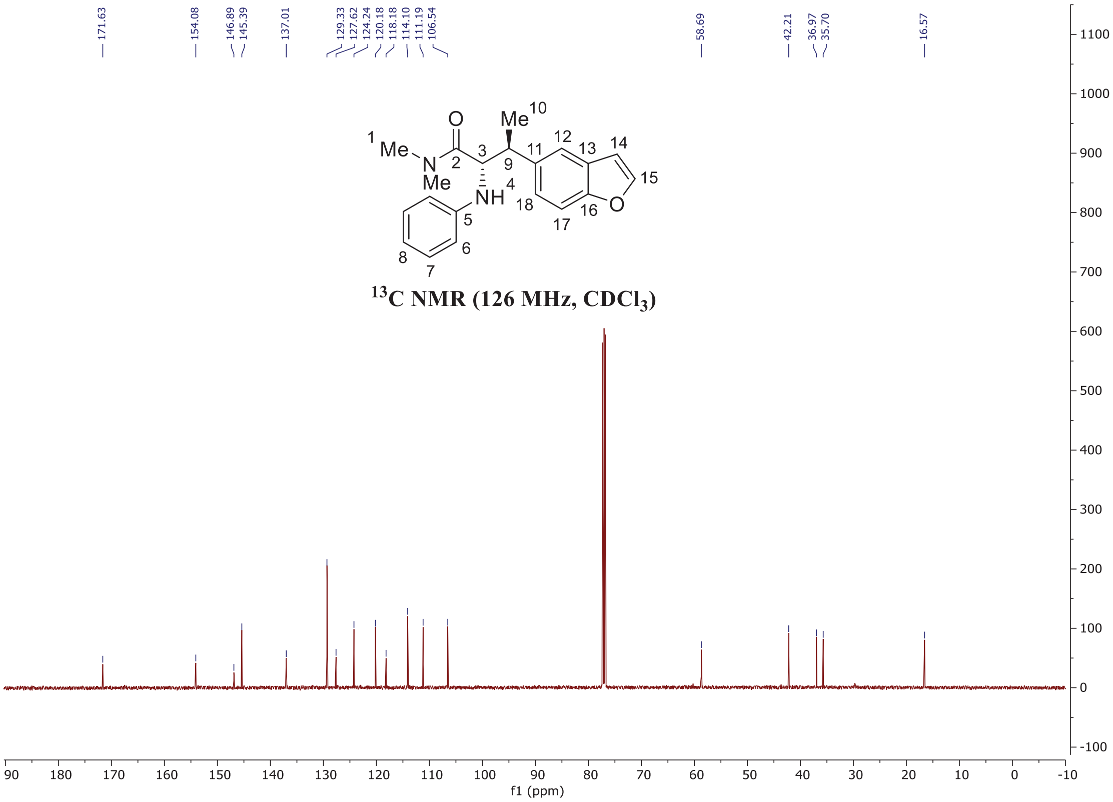

(*2S,3S*)-3-(2-Chlorophenyl)-*N,N*-dimethyl-2-(phenylamino)butanamide (3fn)

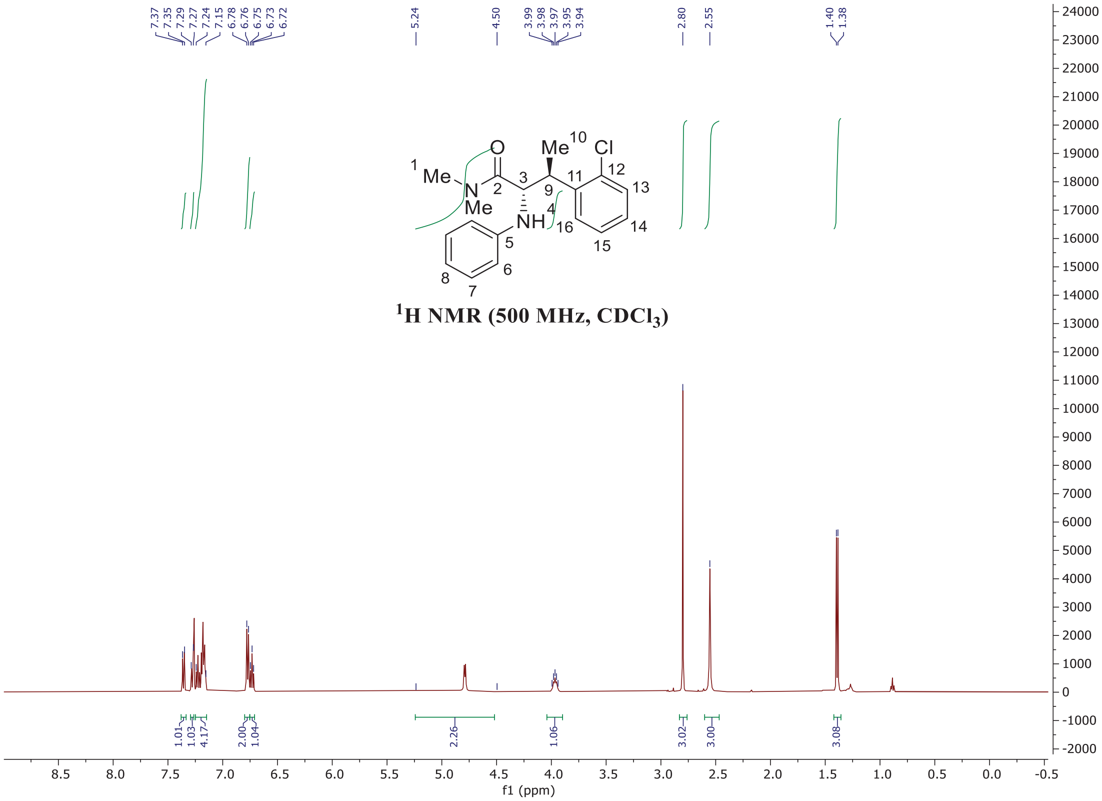

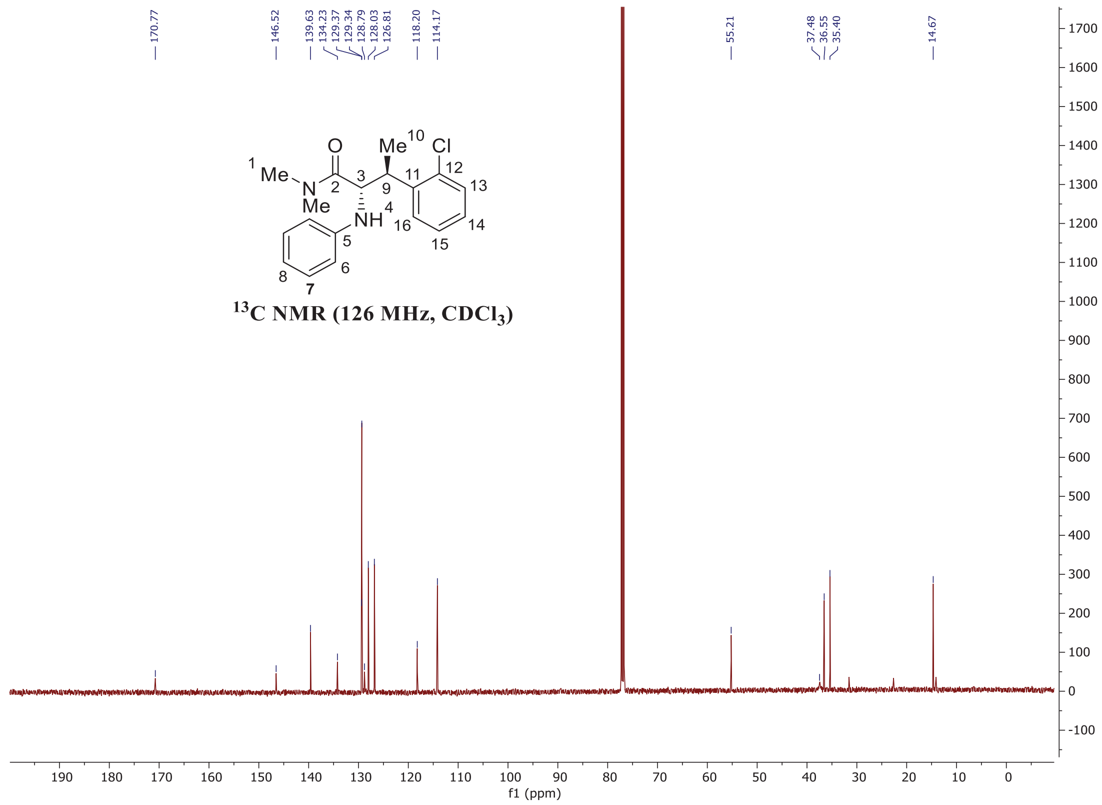

(*2S,3S*)-*N,N*-Dimethyl-3-(perfluorophenyl)-2-(phenylamino)butanamide (3fo)

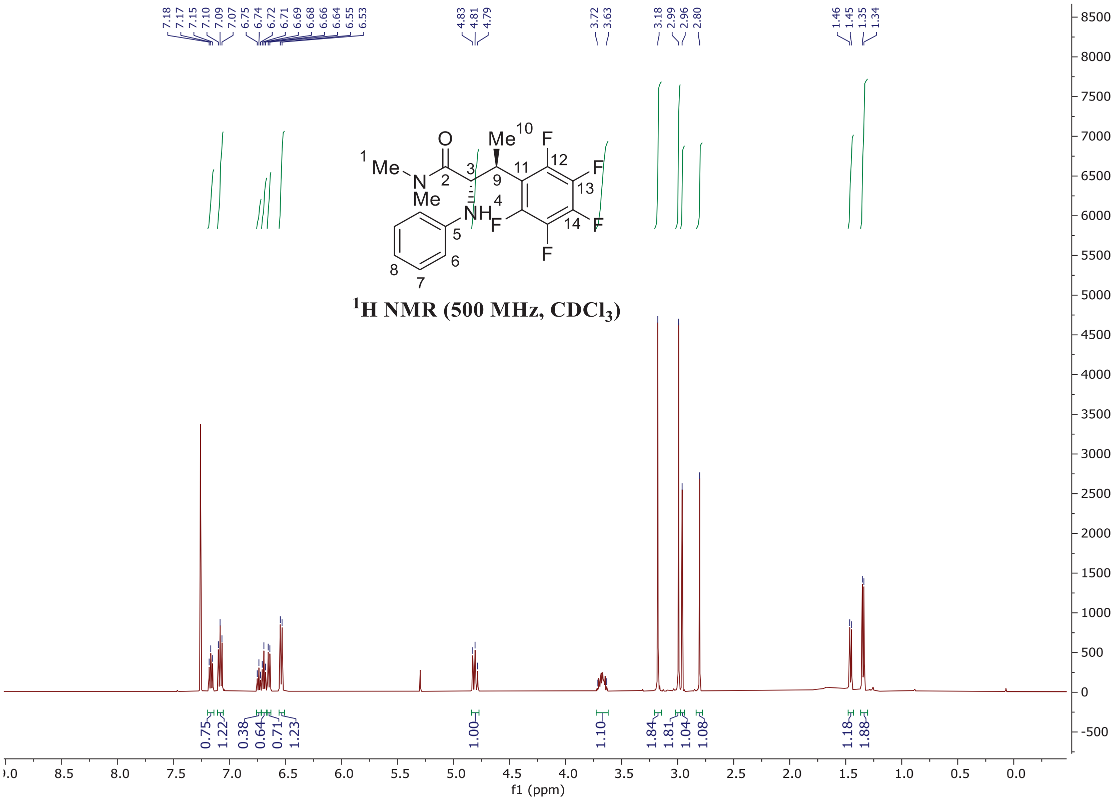

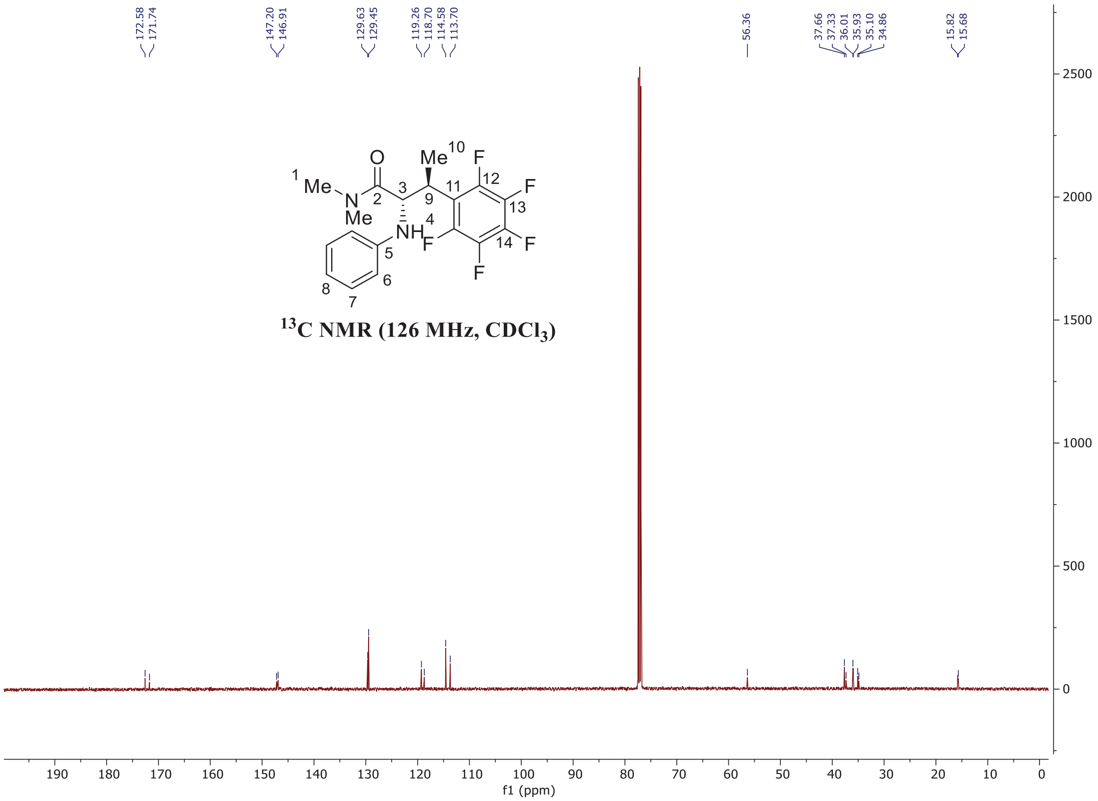
172.58
171.74
147.20
146.91
129.63
129.45
119.26
118.70
114.58
113.70
56.36
37.66
37.33
36.01
35.93
35.10
34.86
15.82
15.68
13C NMR (126 MHz, CDCl3)
f1 (ppm)

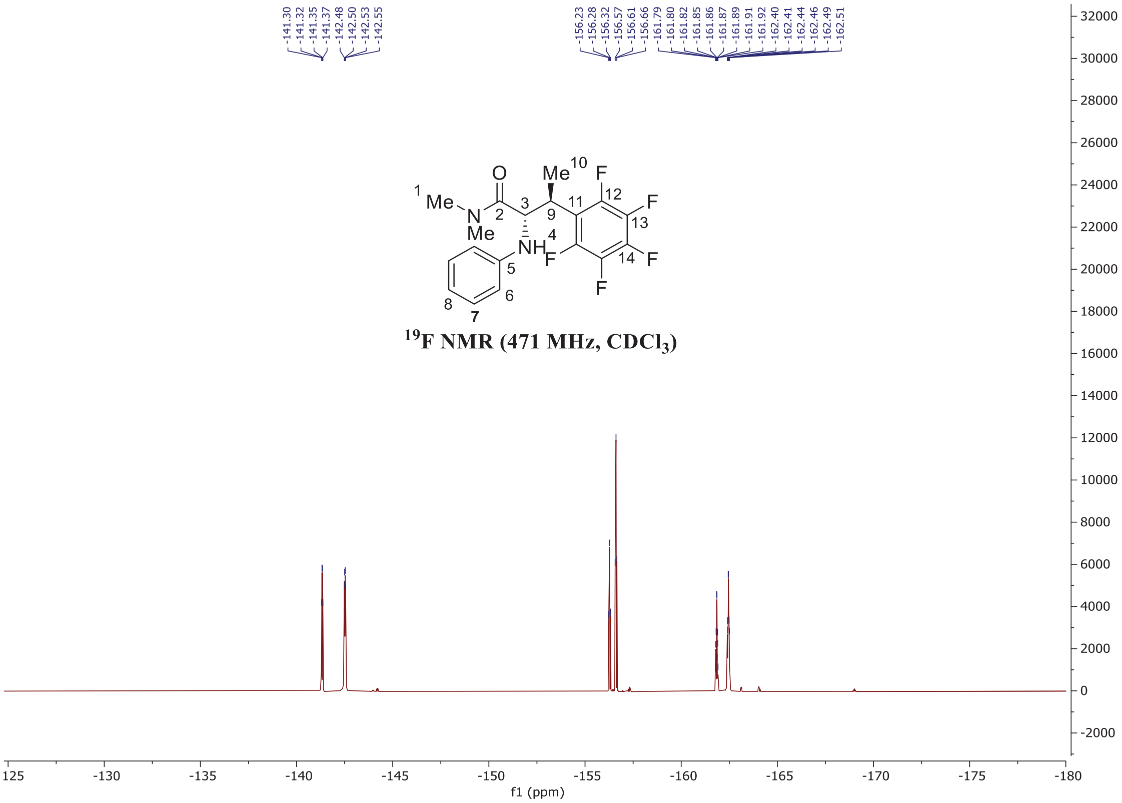
-141.30
-141.32
-141.35
-141.37
-142.48
-142.50
-142.53
-142.55
-156.23
-156.28
-156.32
-156.57
-156.61
-156.66
-161.79
-161.80
-161.82
-161.85
-161.86
-161.87
-161.89
-161.91
-161.92
-162.40
-162.41
-162.44
-162.46
-162.49
-162.51
19F NMR (471 MHz, CDCl3)
f1 (ppm)

((*2S,3S*)-2-((4-Hydroxyphenyl)amino)-*N,N*-dimethyl-3-(1-(methyl-(λ[1]-oxidaneyl)-(*p*-tolyl)sulfinyl)-1*H*-indol-3-yl)butanamide (3pf)

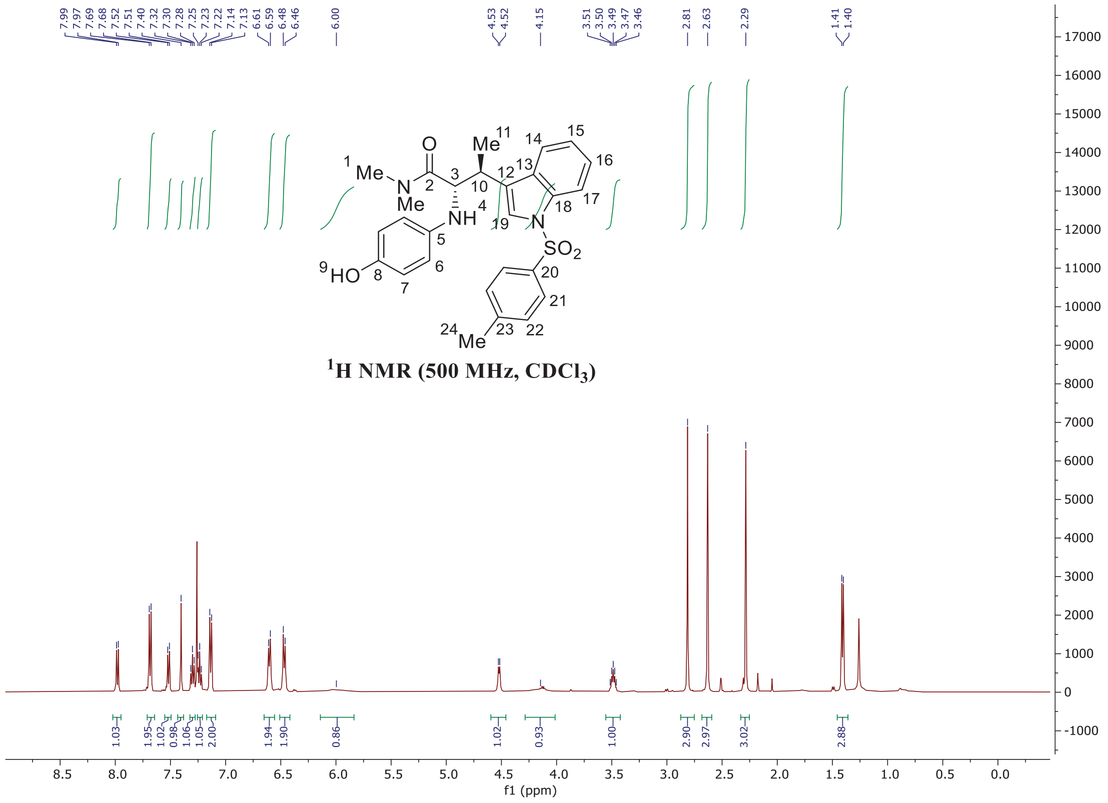

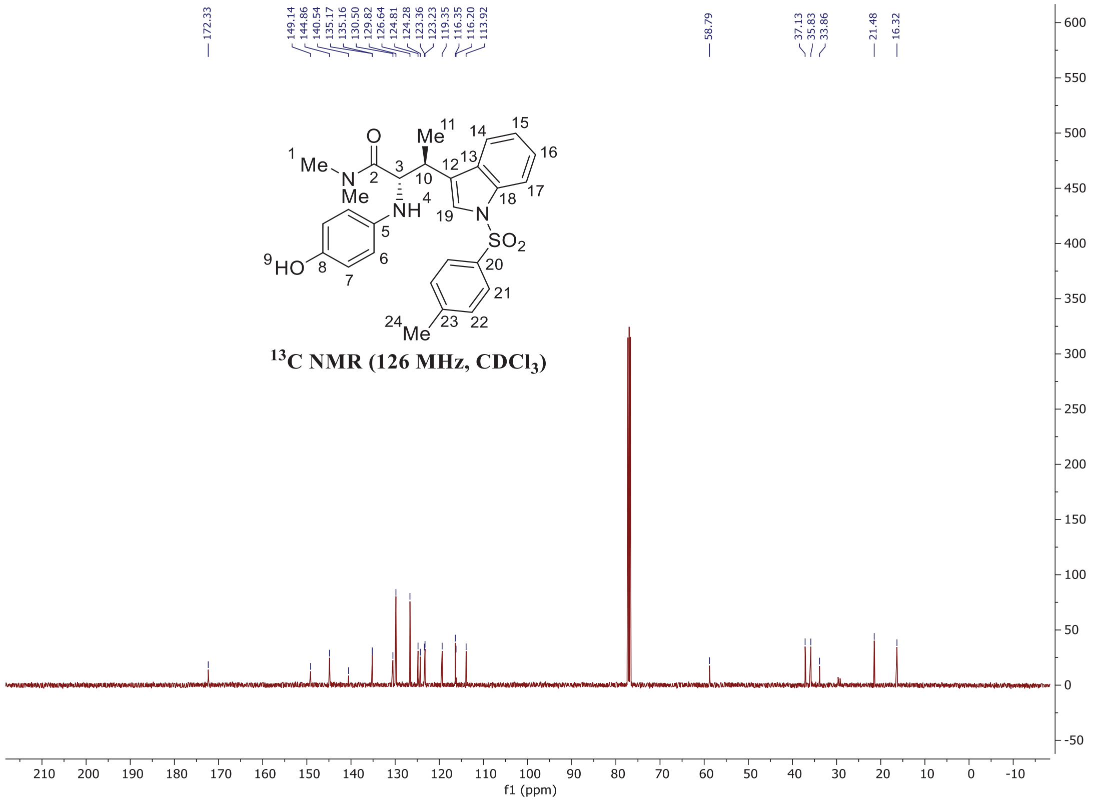

(*2S,3S*)-3-(3-Chlorophenyl)-2-((4-hydroxyphenyl)amino)-1-morpholinobutan-1-one (3wk)

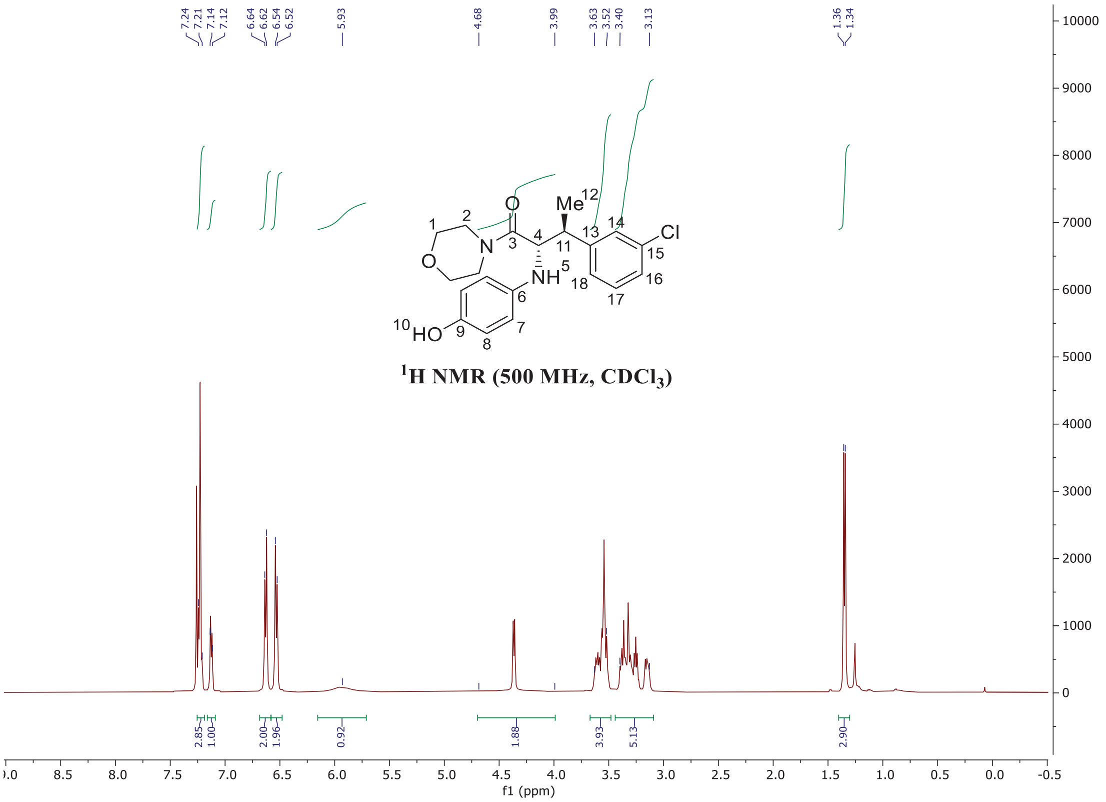

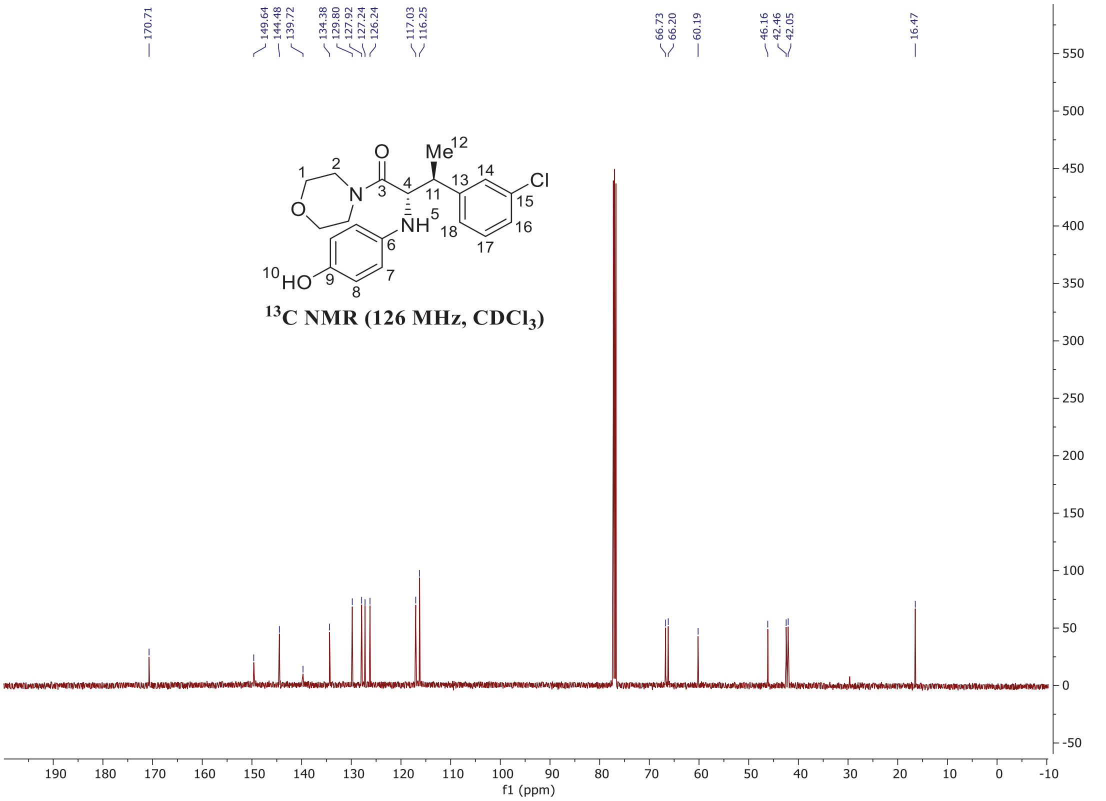

(*4S,5S*)-2,2,5-Trimethyl-4-(phenylamino)nonan-3-one (3op)

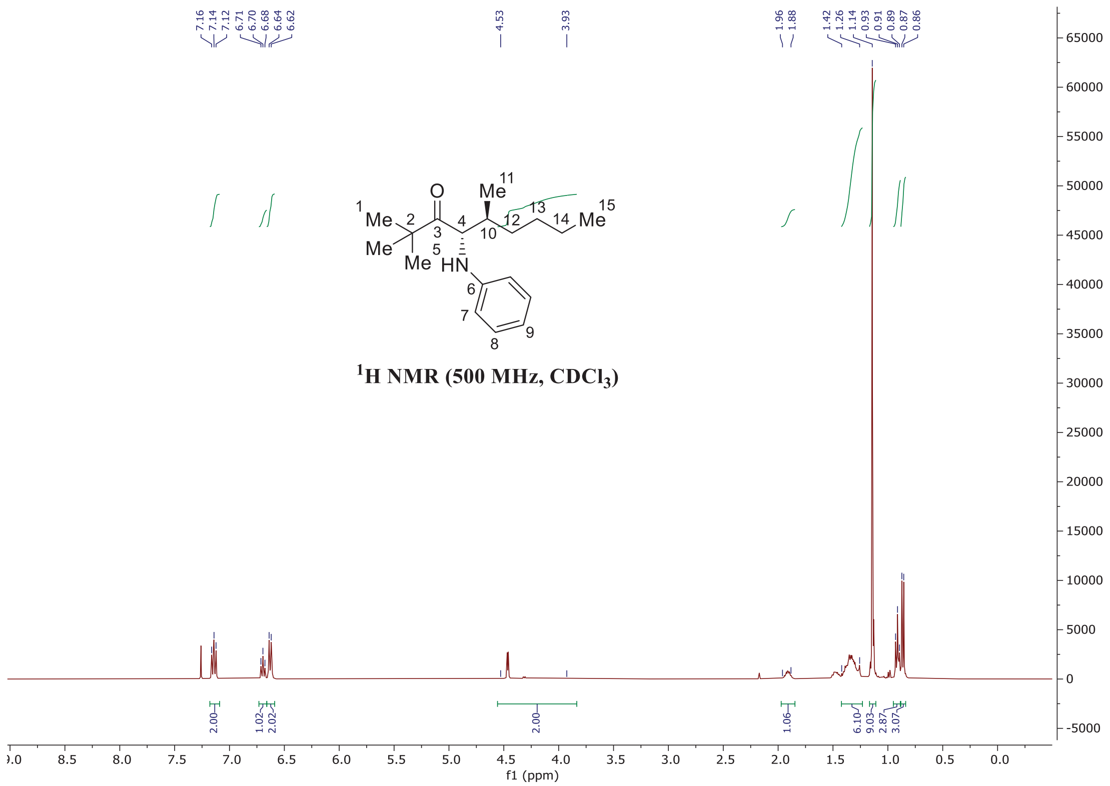

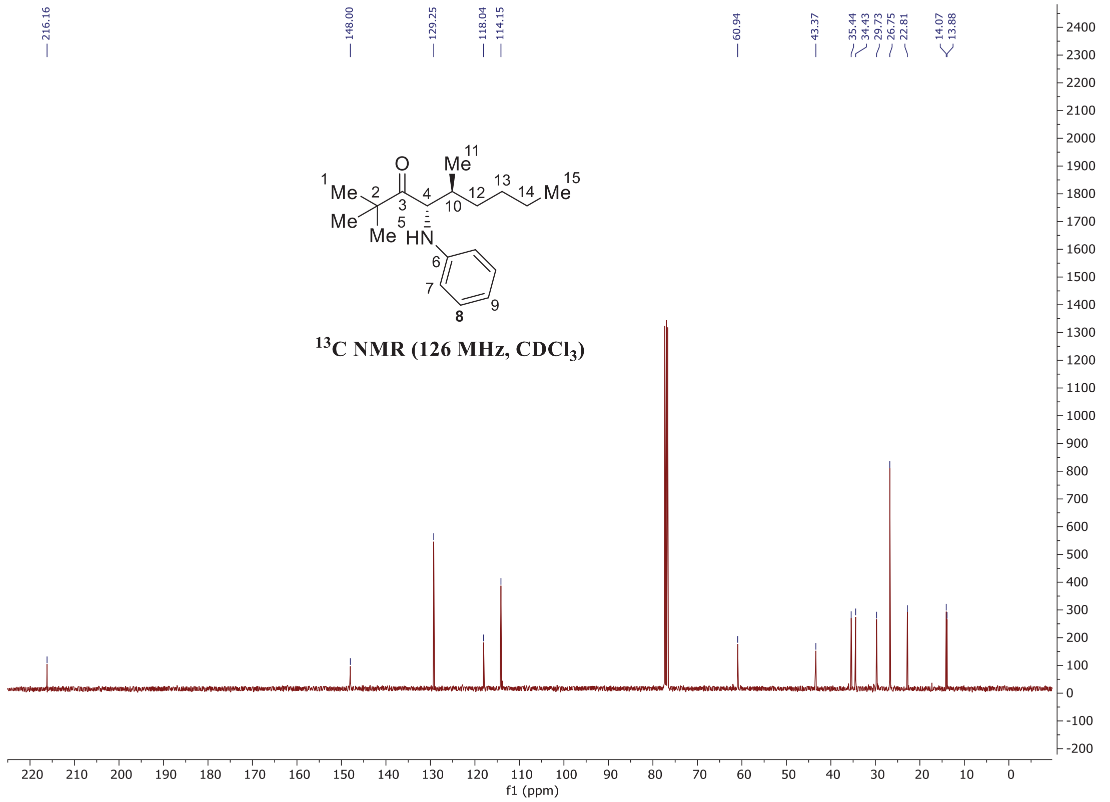

(*4S,5S*)-2,2,5,7-Tetramethyl-4-(phenylamino)octan-3-one (3oq)

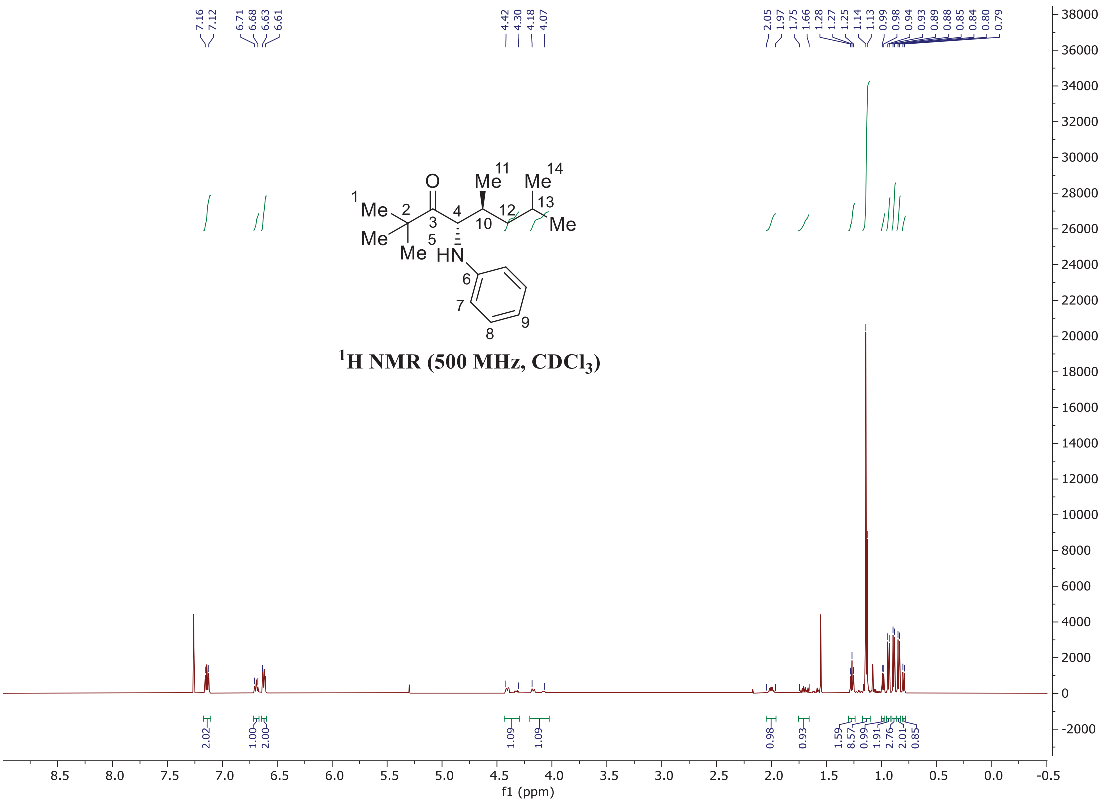

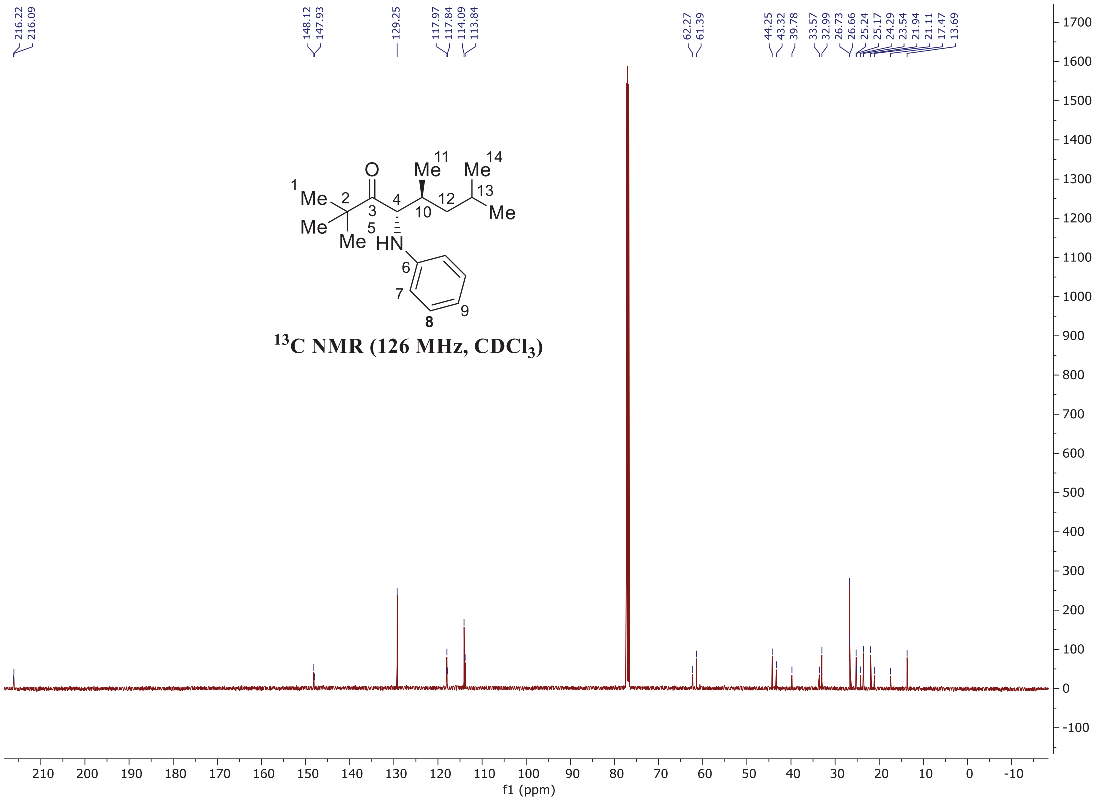

(4S,5S)-2,2,5,6-Tetramethyl-4-(phenylamino)heptan-3-one (3or)

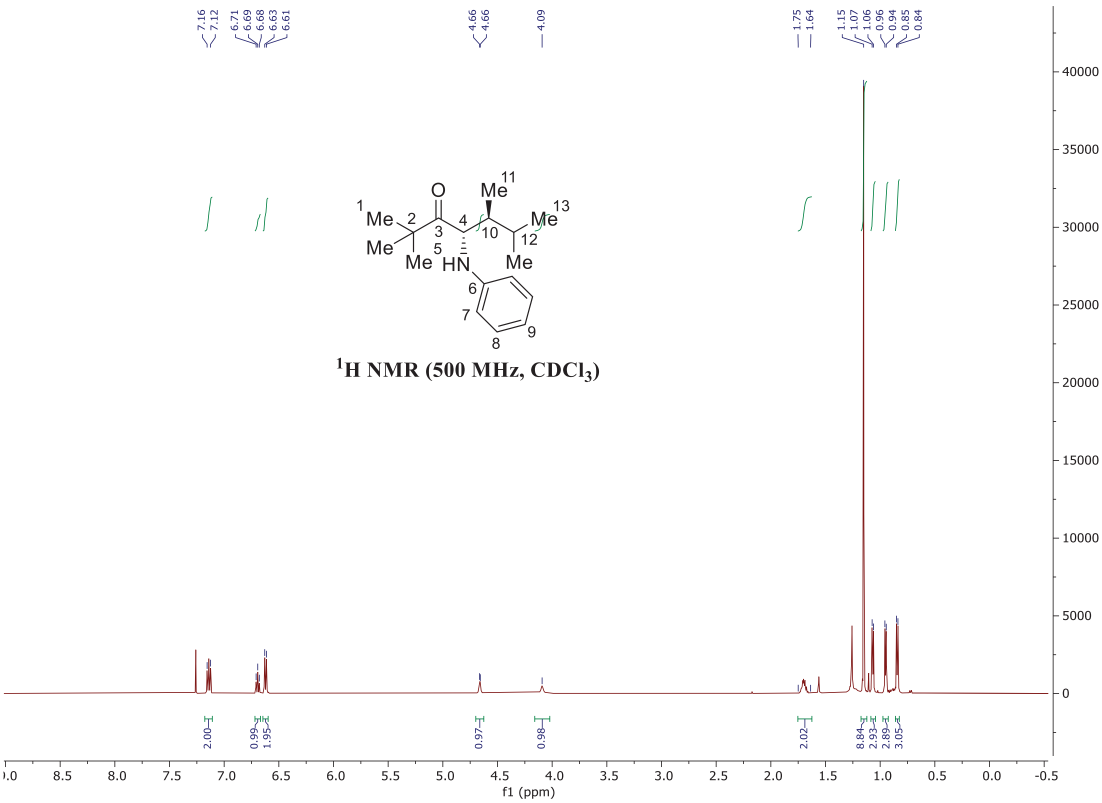

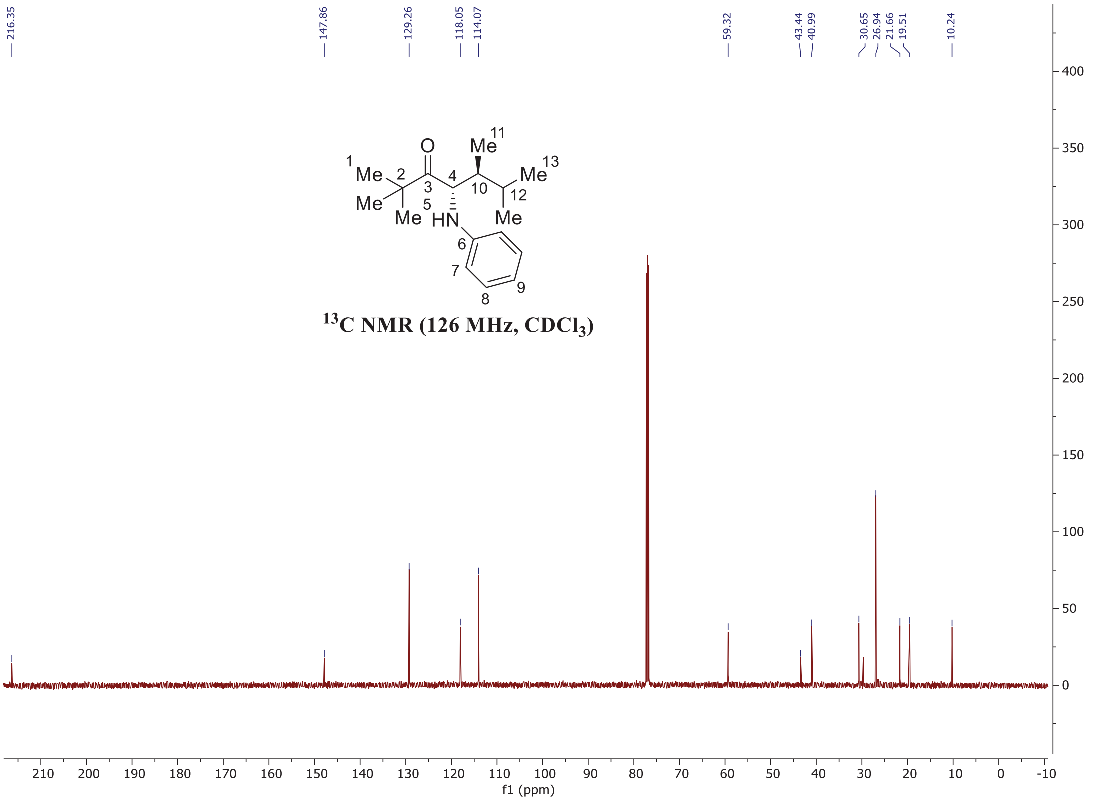

(*4S,5S*)-9-((*Tert*-butyldimethylsilyl)oxy)-2,2,5-trimethyl-4-(phenylamino)nonan-3-one (3os)

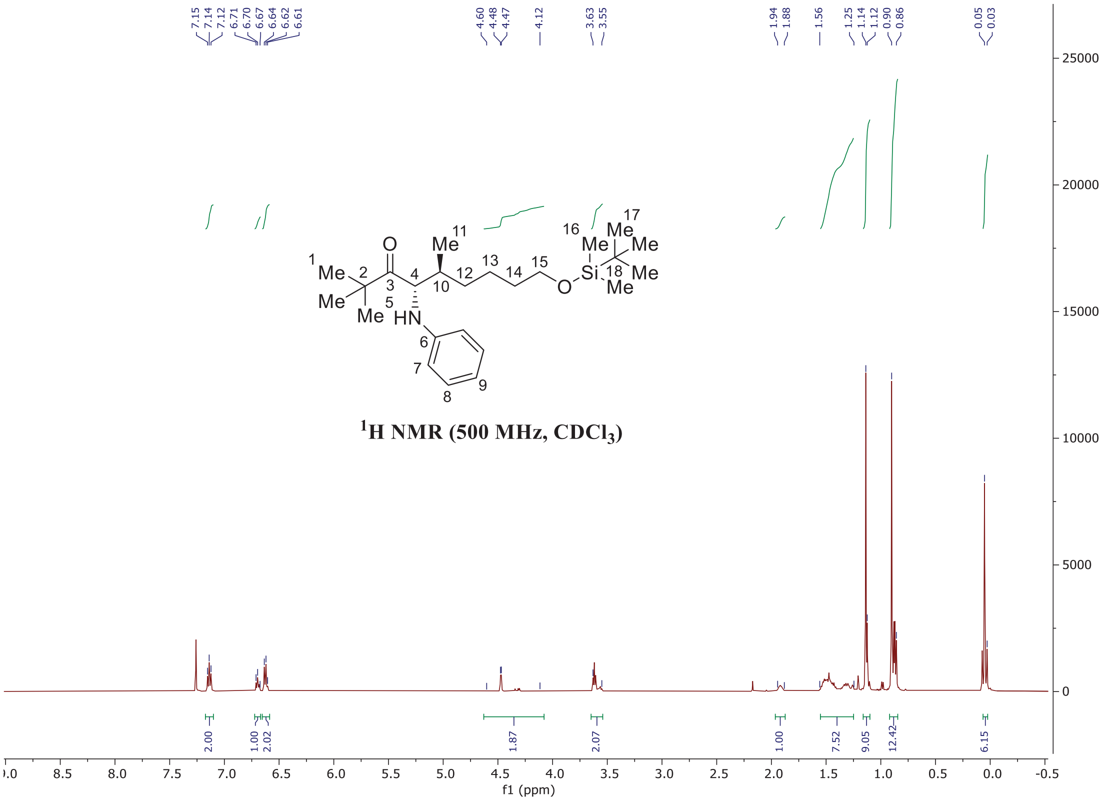

^{1}H NMR (500 MHz, CDCl$_3$)

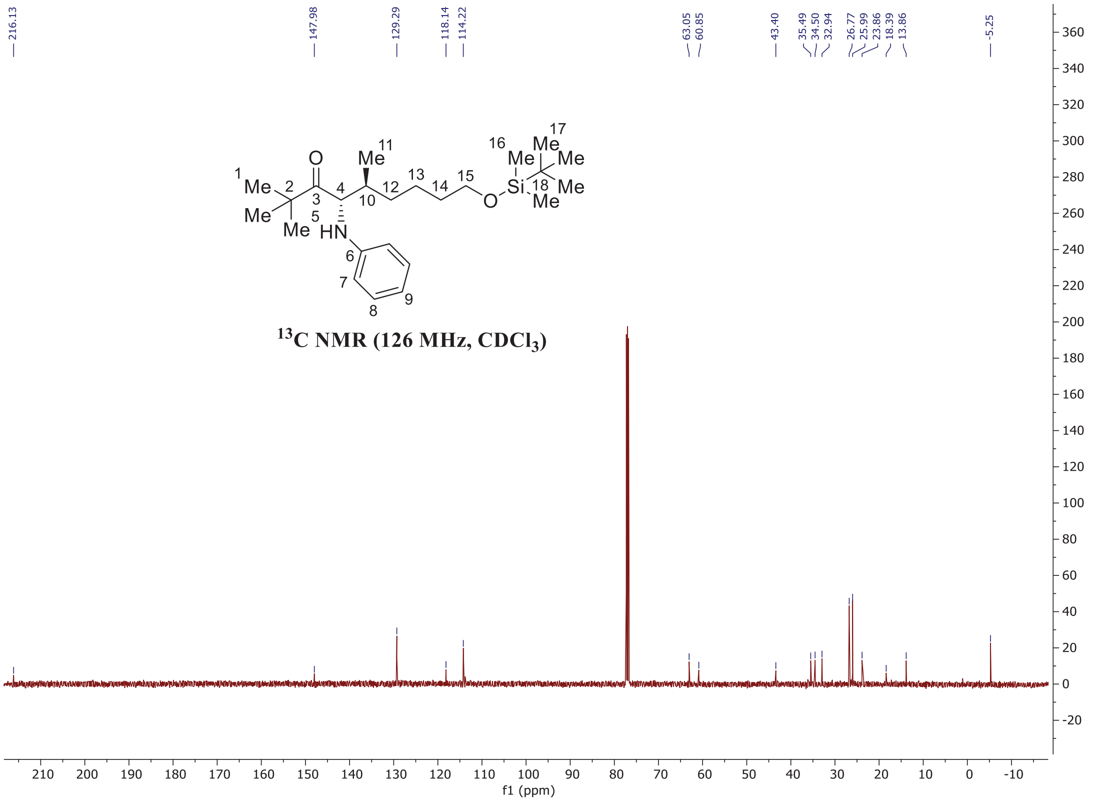

^{13}C NMR (126 MHz, CDCl$_3$)

4-((*2S,3S*)-4-(Dimethylamino)-4-oxo-3-(phenylamino)butan-2-yl)benzyl 2-(1-(4-chlorobenzoyl)-5-methoxy-2-methyl-1*H*-indol-3-yl)acetate (3ft)

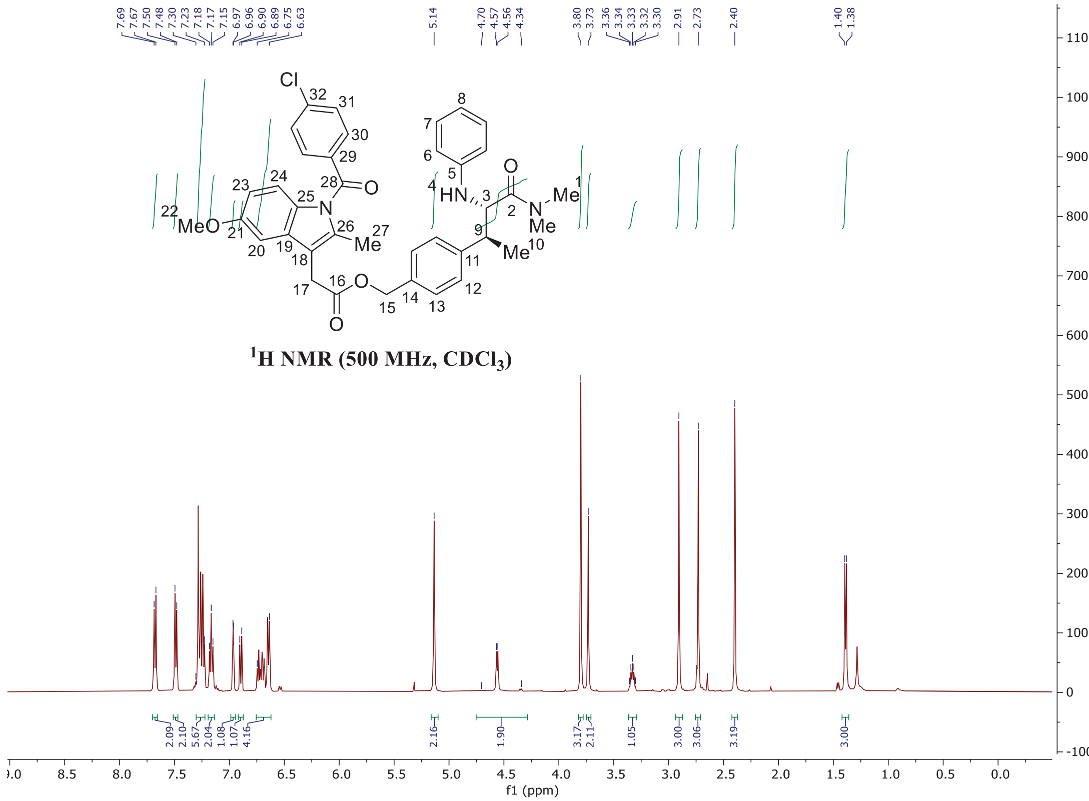

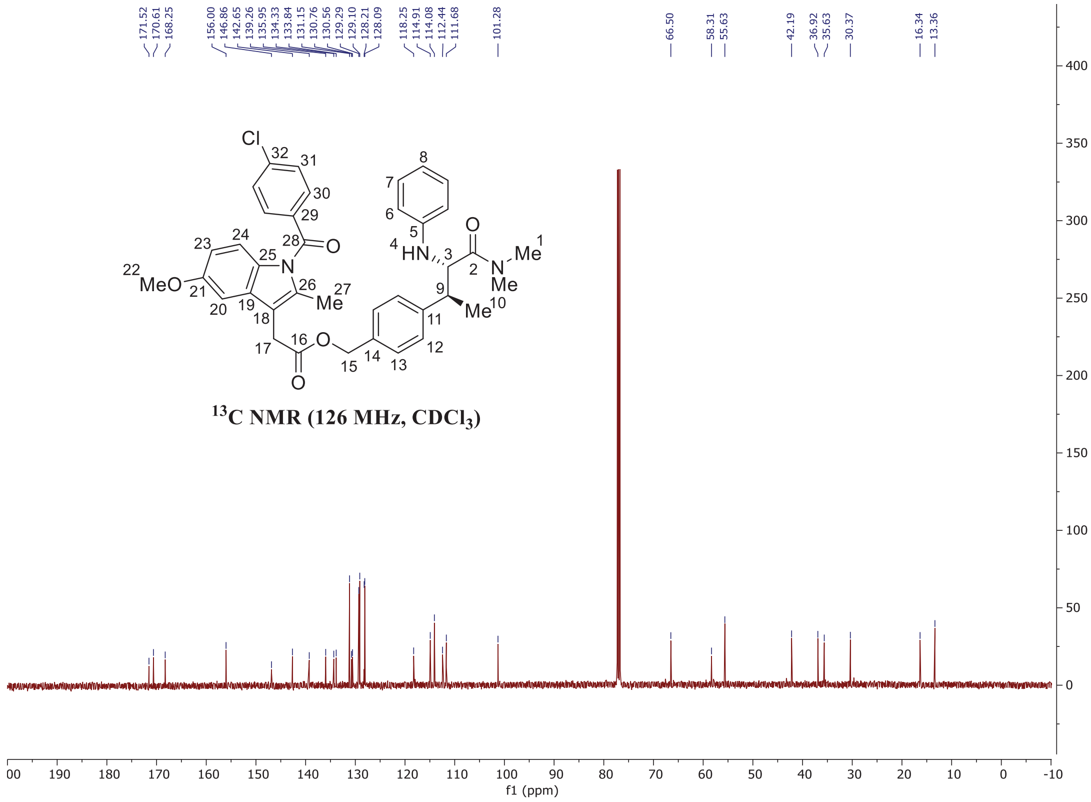

(*2S,3S*)-3-(4-(*Tert*-butyl)phenyl)-*N*,*N*-dimethyl-2-(phenylamino)butanamide (3fu)

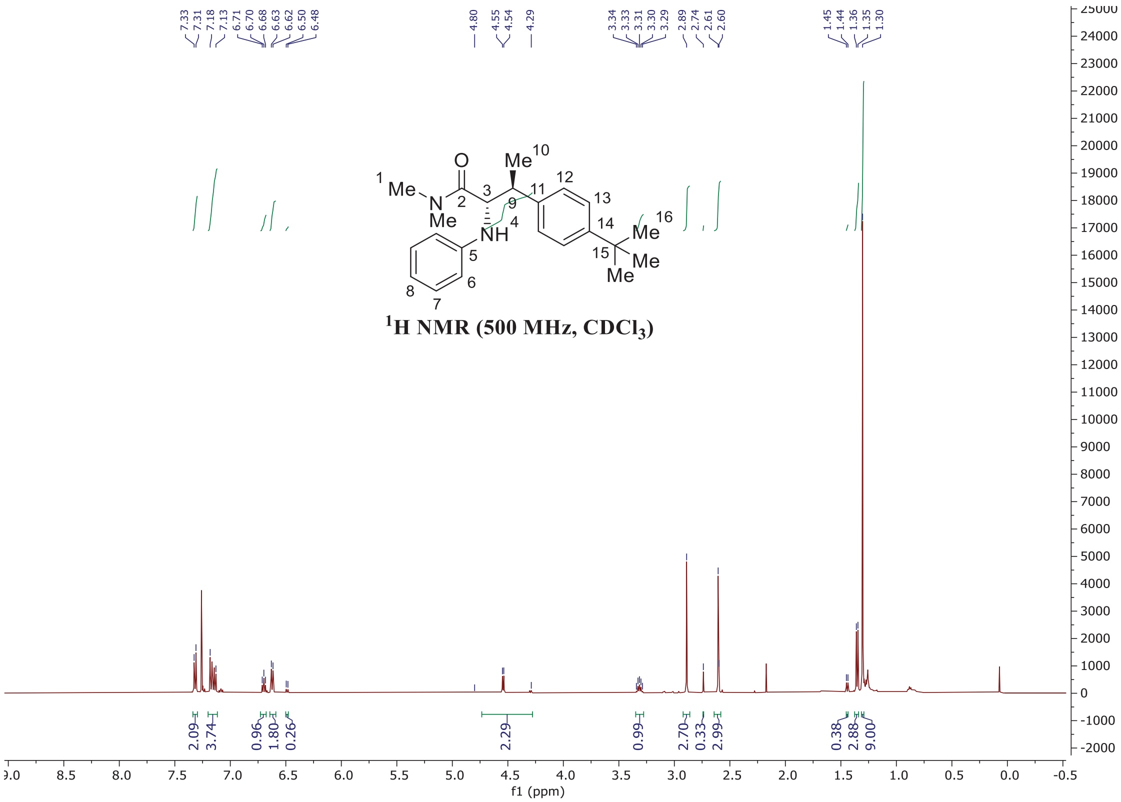

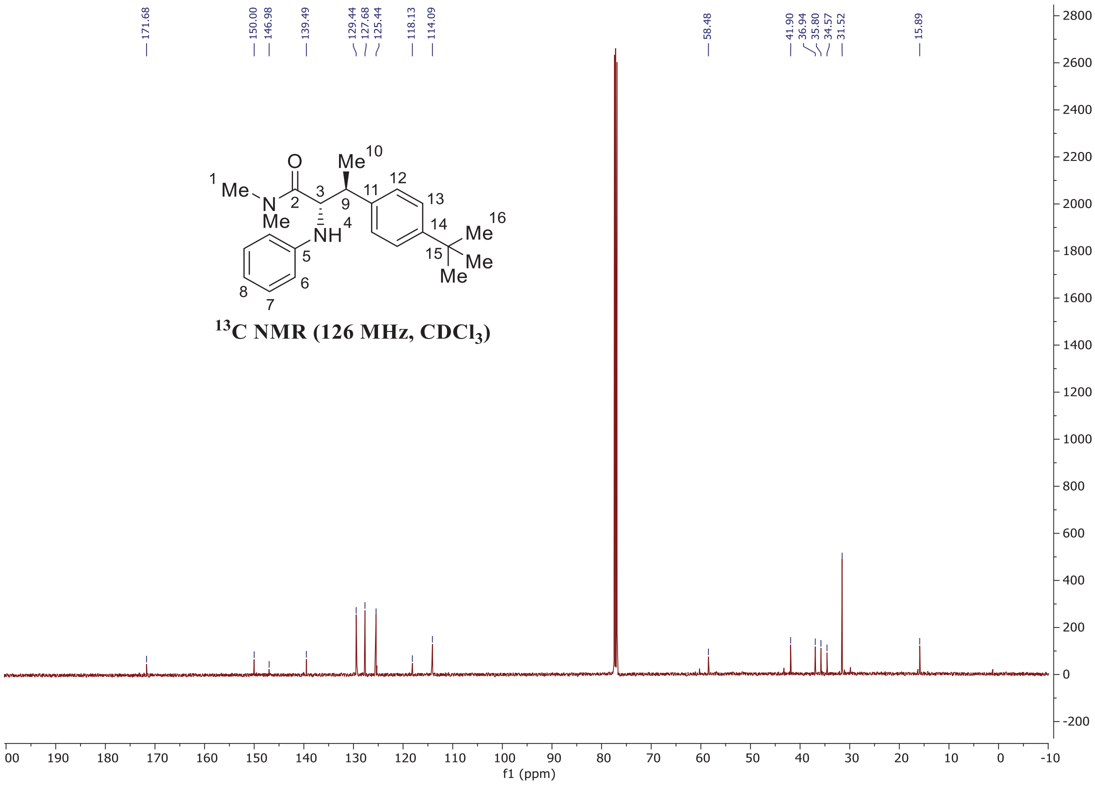

(*2S,3S*)-3-(4-Fluorophenyl)-*N,N*-dimethyl-2-(phenylamino)butanamide (3fv)

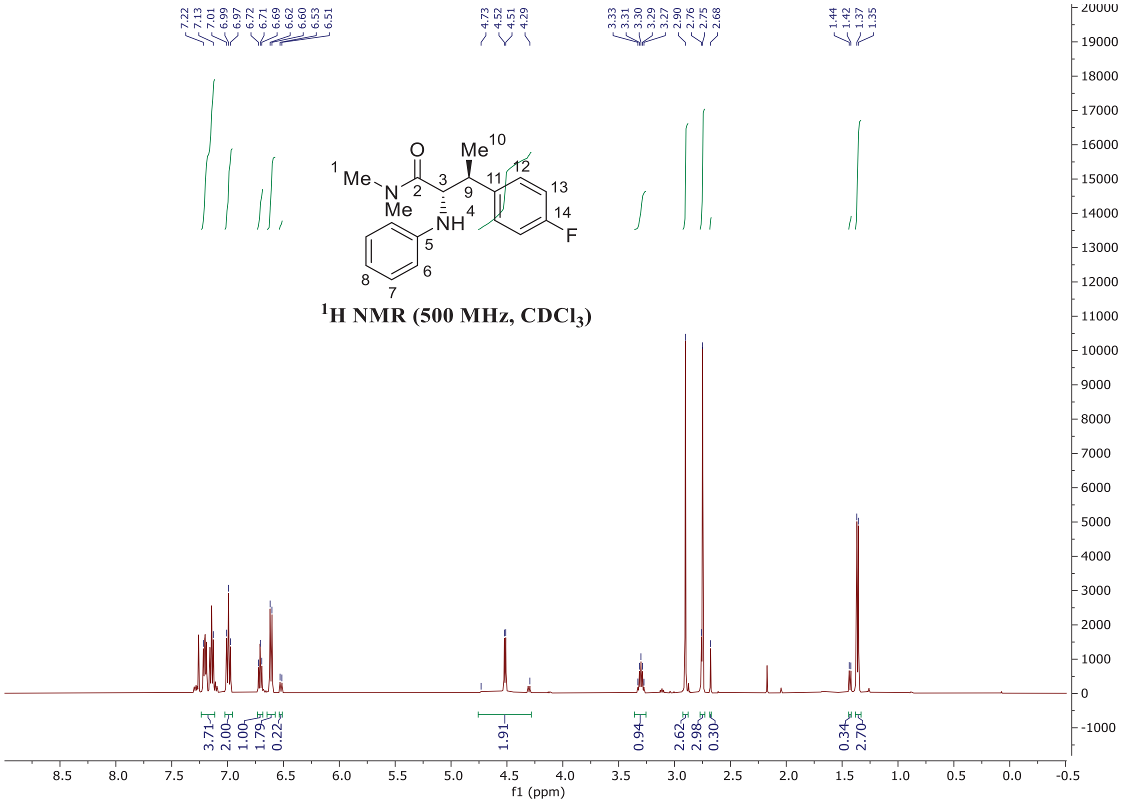

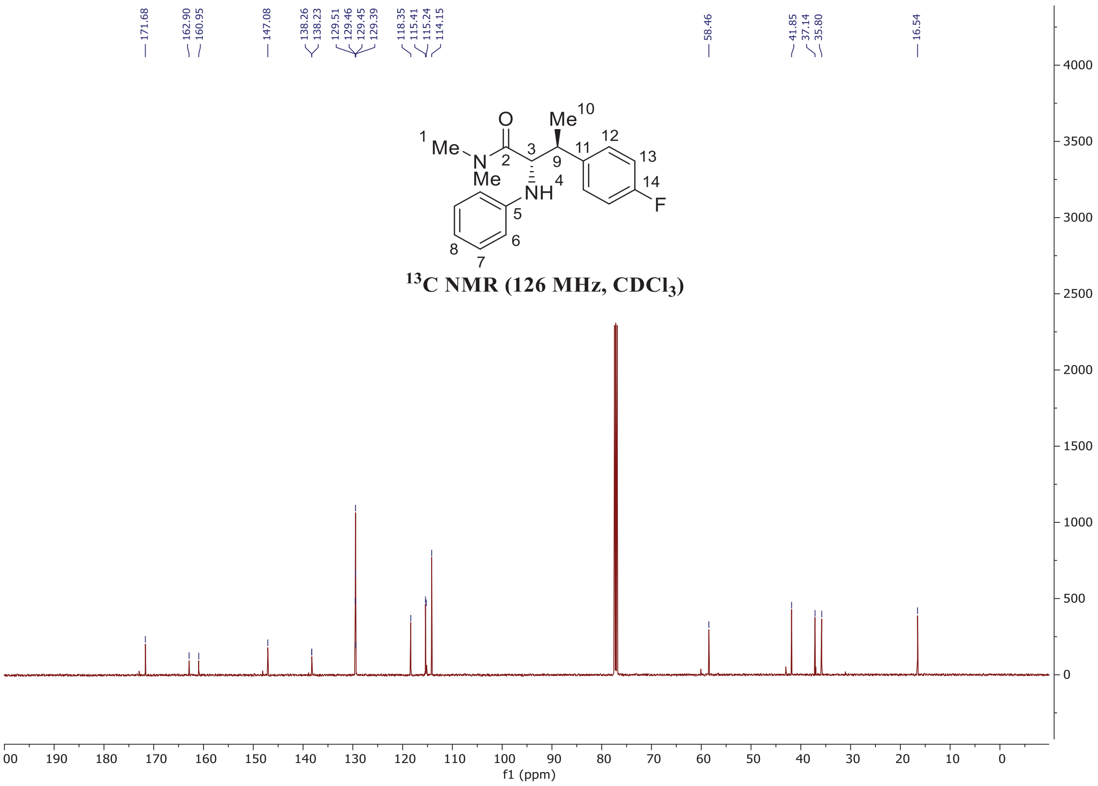

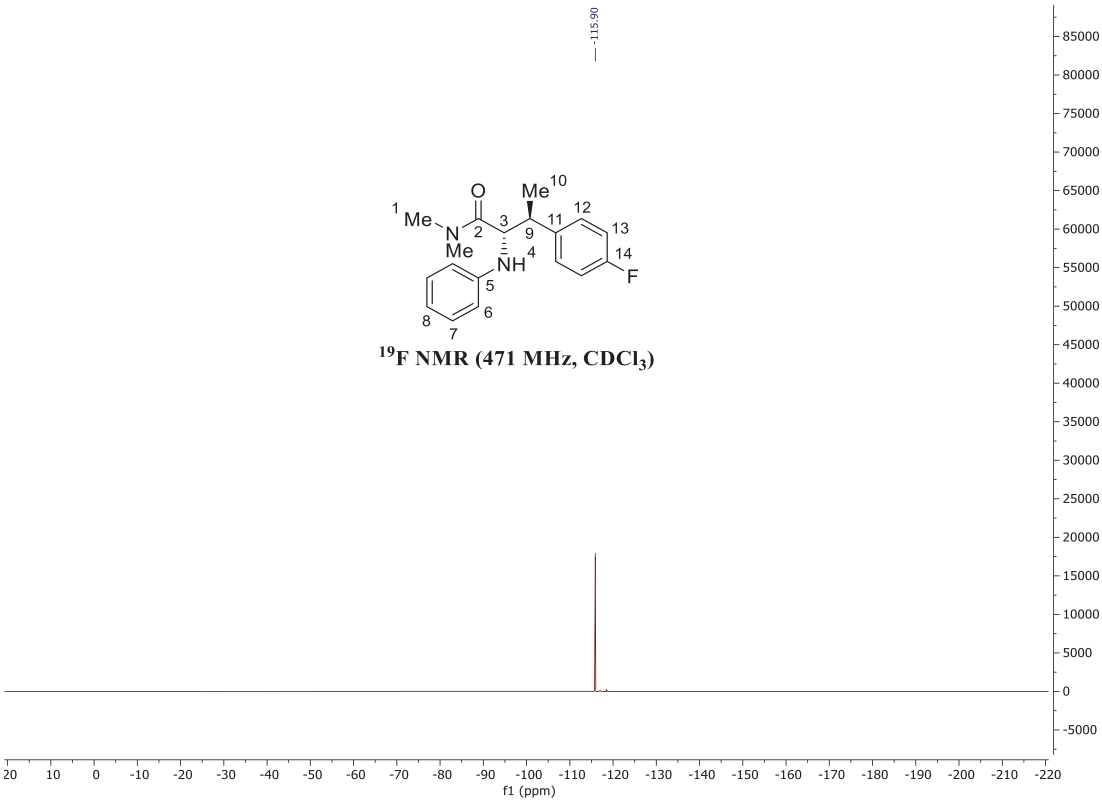

(*2S,3S*)-3-(3-Chlorophenyl)-*N,N*-dimethyl-2-(phenylamino)butanamide (3fw)

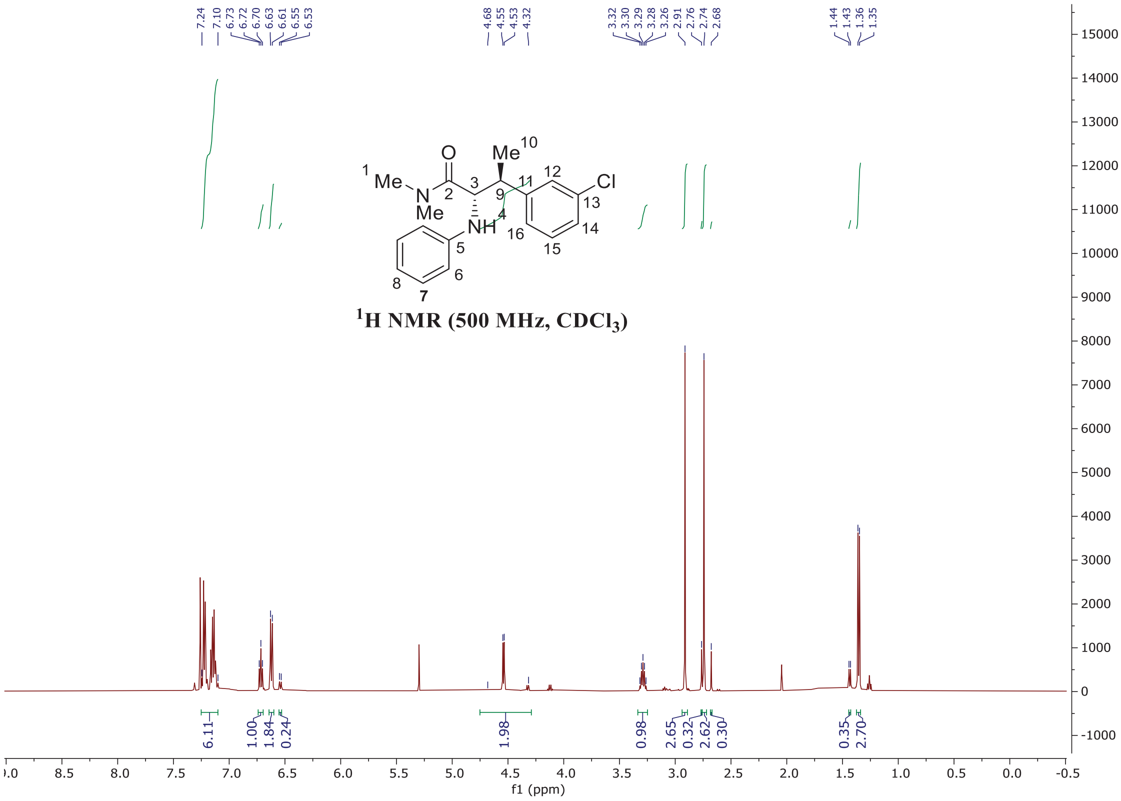

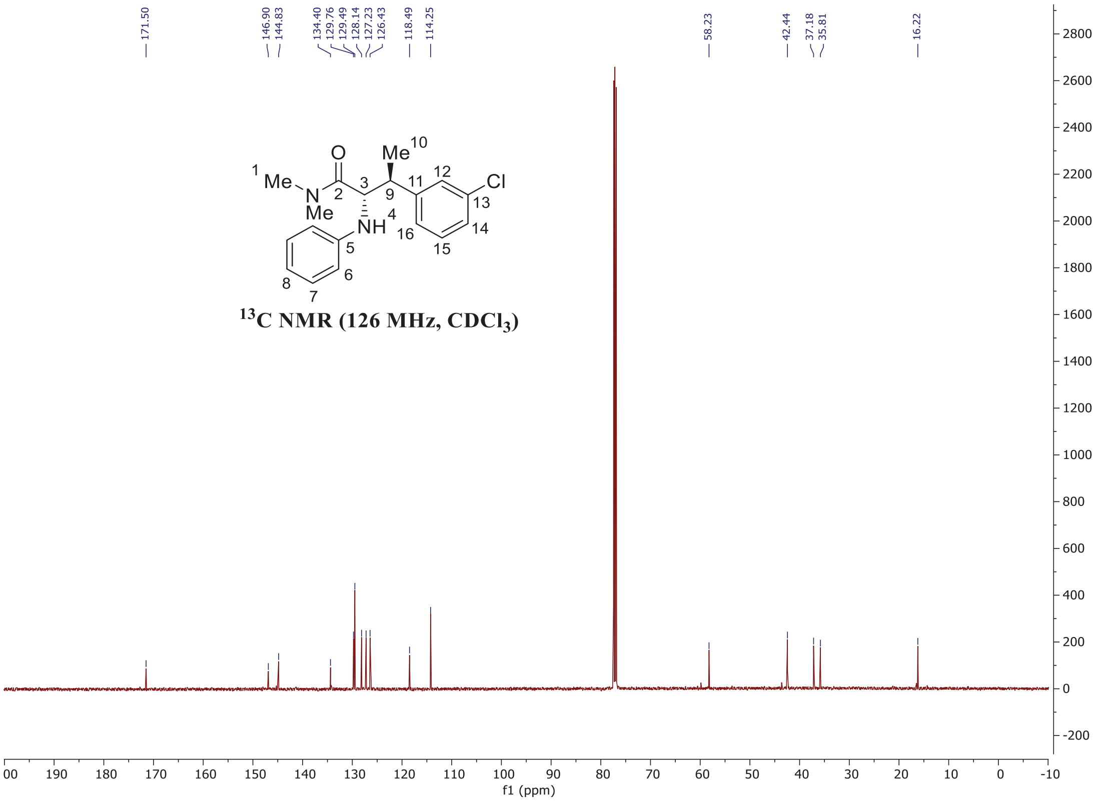

(*2S,3S*)-3-(2-Fluorophenyl)-*N*,*N*-dimethyl-2-(phenylamino)butanamide (3fx)

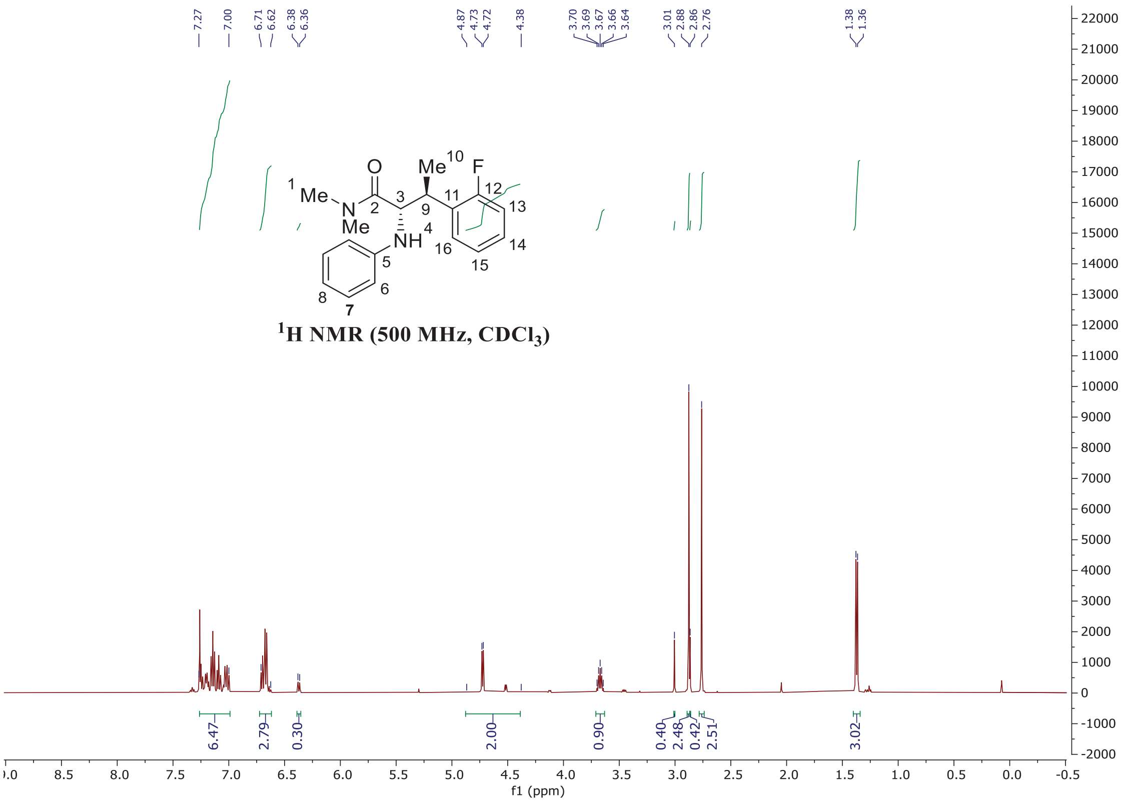

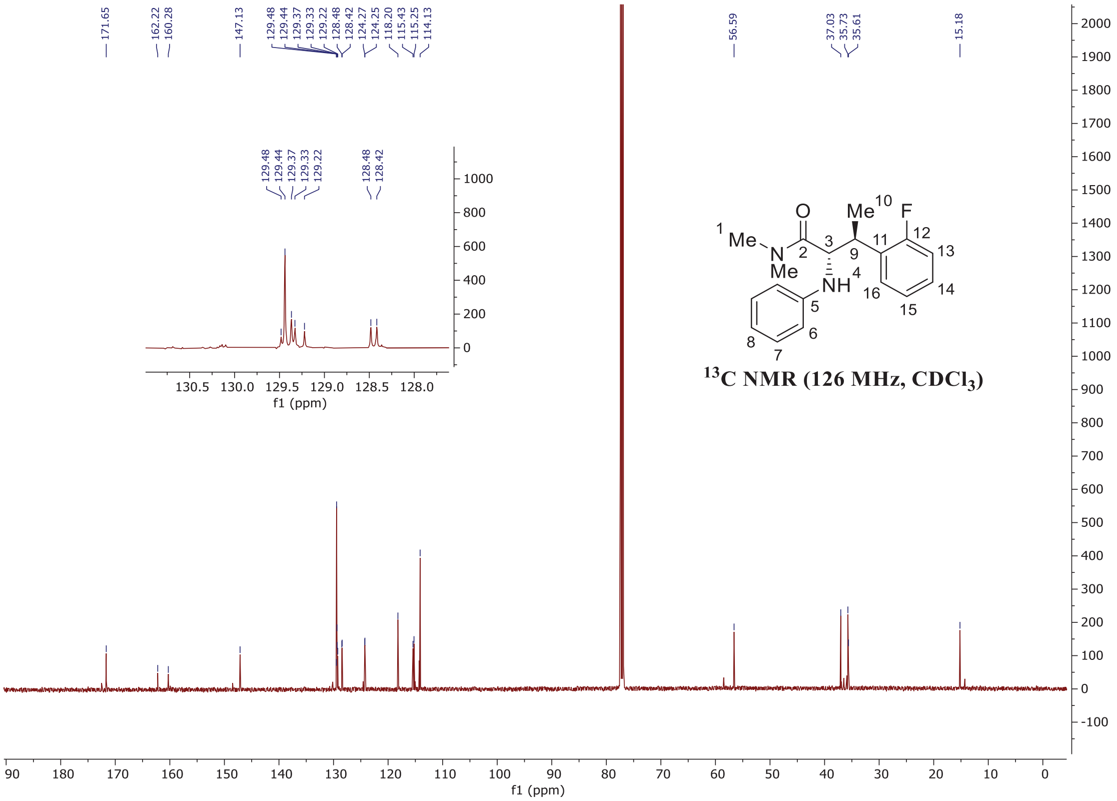
171.65
162.22
160.28
147.13
129.48
129.44
129.37
129.33
129.22
128.48
128.42
124.27
124.25
118.20
115.43
115.25
114.13
56.59
37.03
35.73
35.61
15.18
f1 (ppm)
^{13}C NMR (126 MHz, CDCl$_3$)
Me
N
Me
O
NH
Me
F

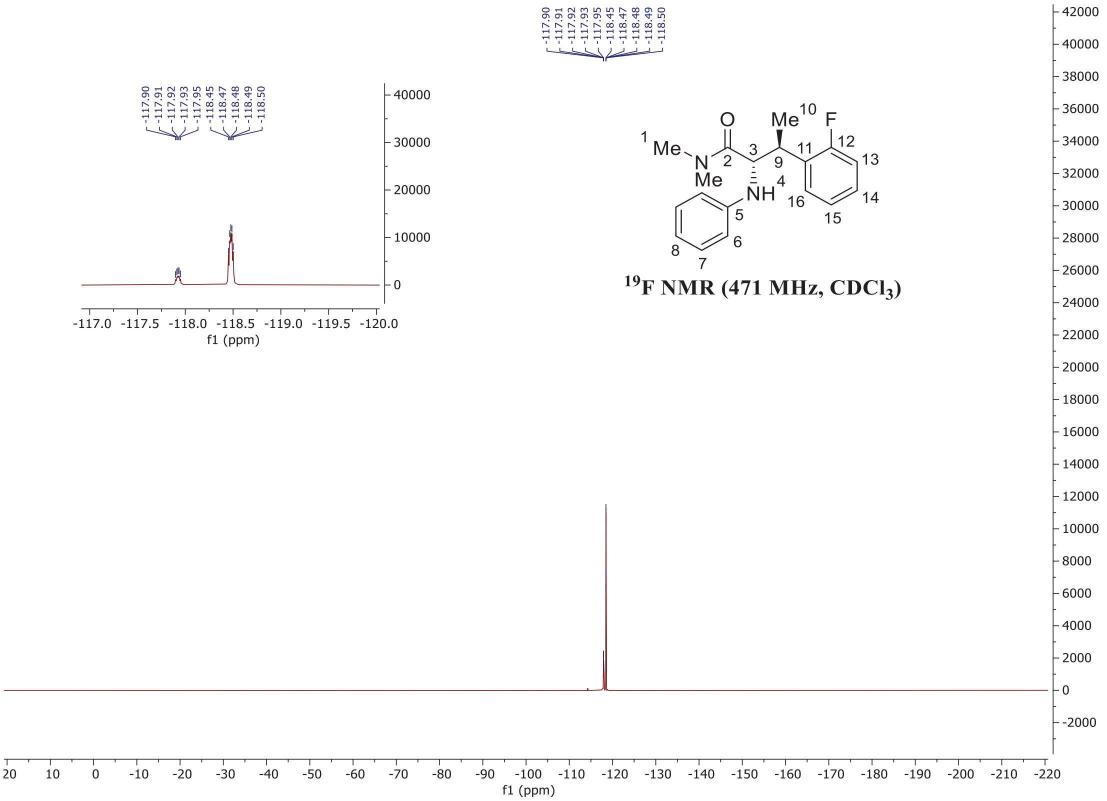
-117.90
-117.91
-117.92
-117.93
-117.95
-118.45
-118.47
-118.48
-118.49
-118.50
f1 (ppm)
^{19}F NMR (471 MHz, CDCl$_3$)
Me
N
Me
O
NH
Me
F

(2S,3S)-3-Phenyl-2-(phenylamino)butan-1-ol (4)

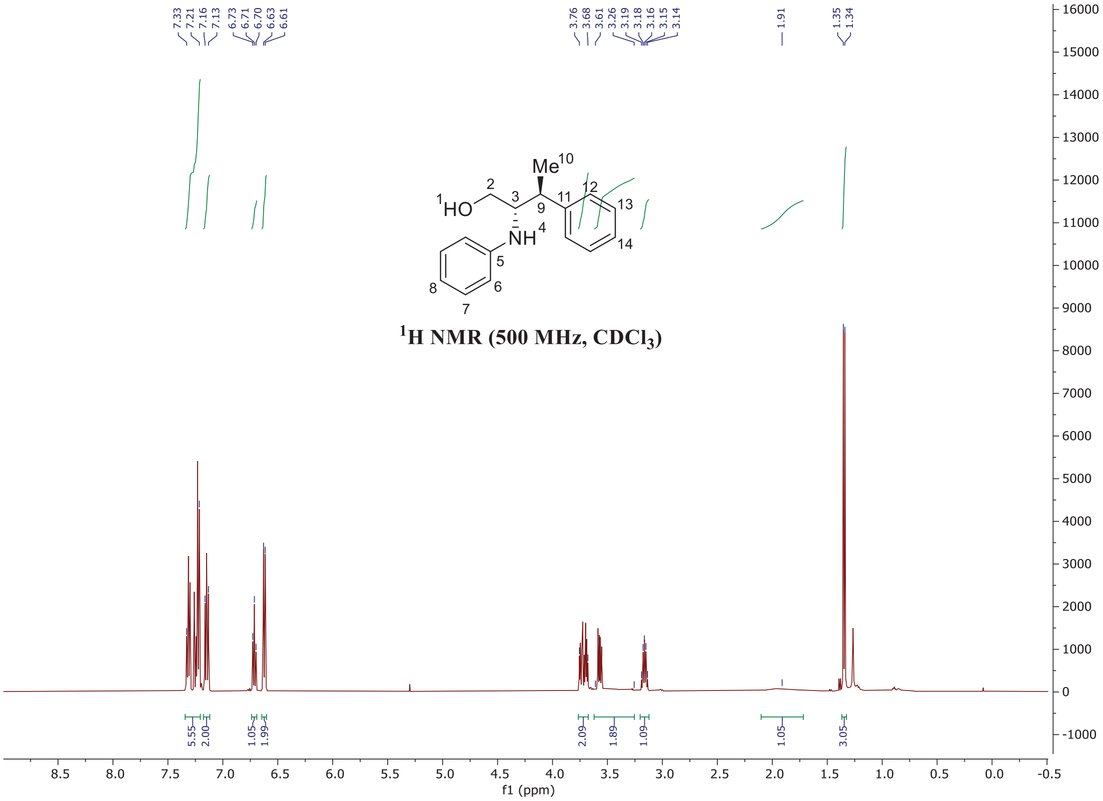

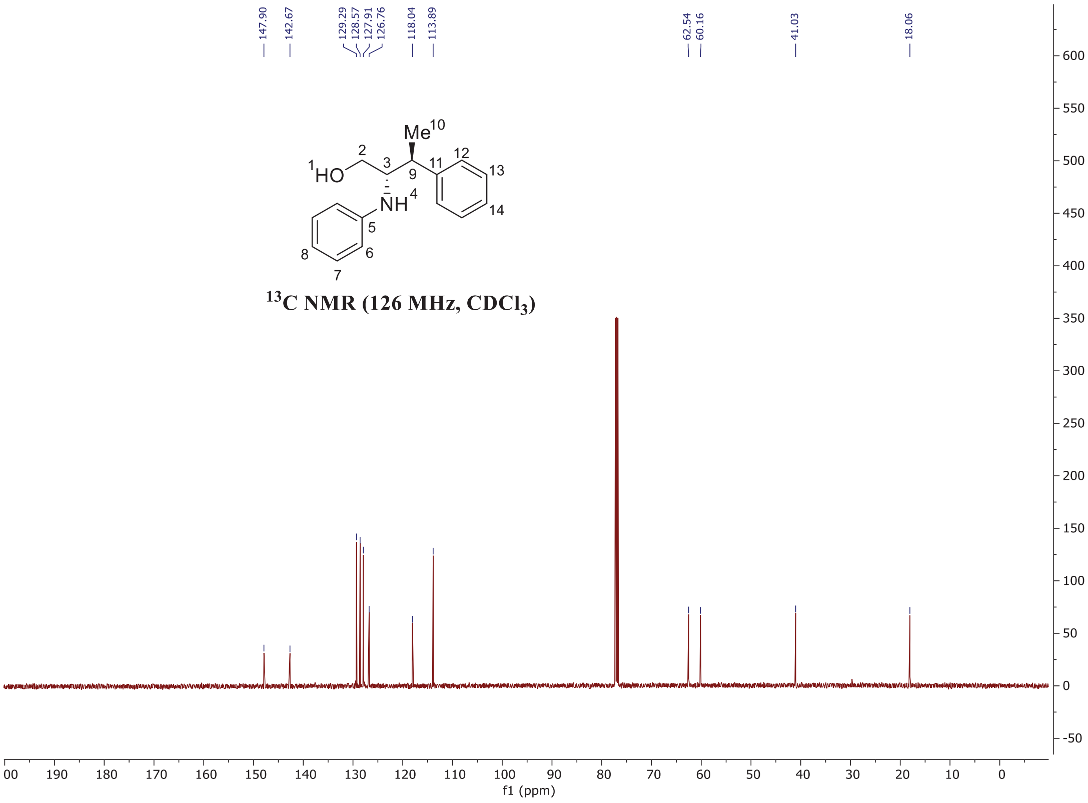

(*2S,3S*)-3-Phenyl-2-(phenylamino)butanal (5)

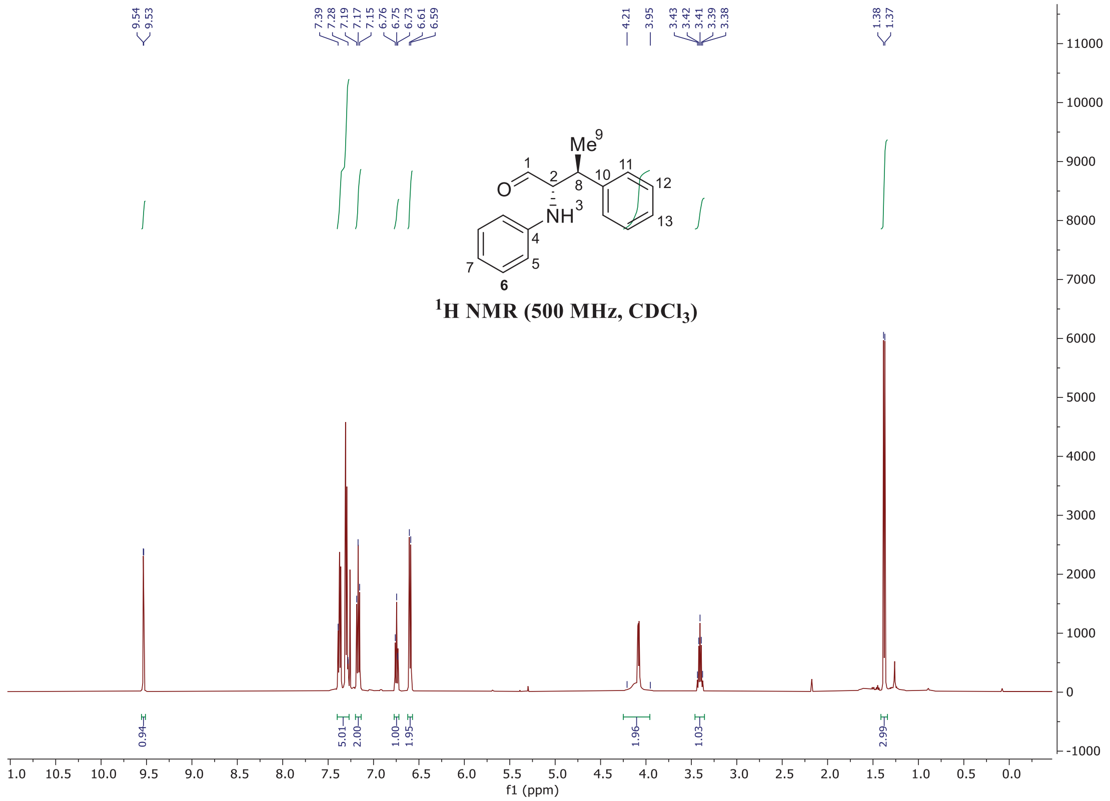

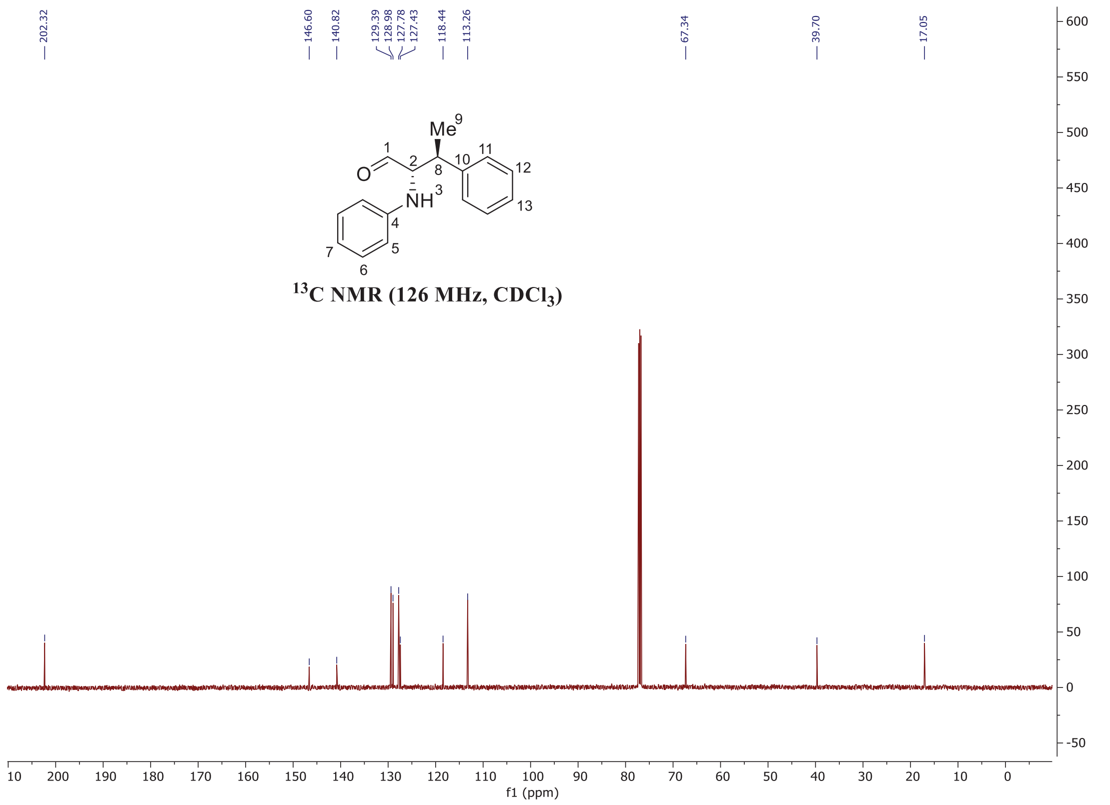

N-((*2S*,*3S*)-1-Morpholino-3-phenylbutan-2-yl)aniline (6)

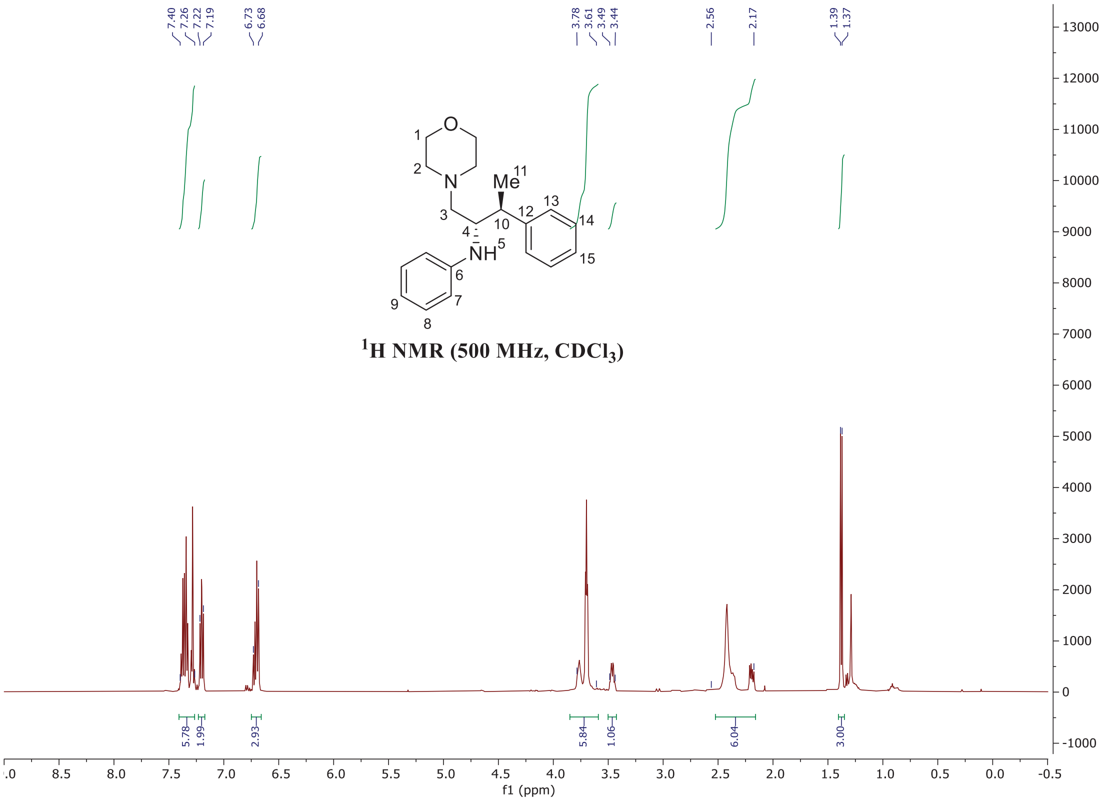

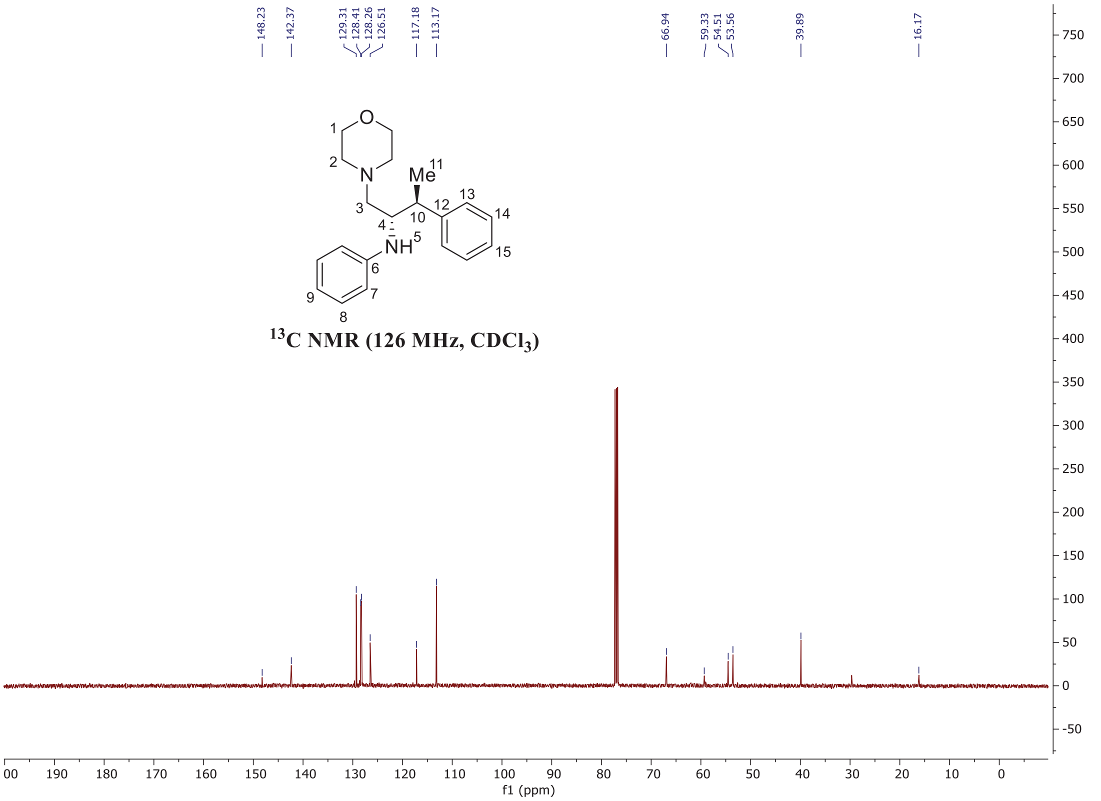

(*S*)-*N*-(2-Phenylpropyl)aniline (8)

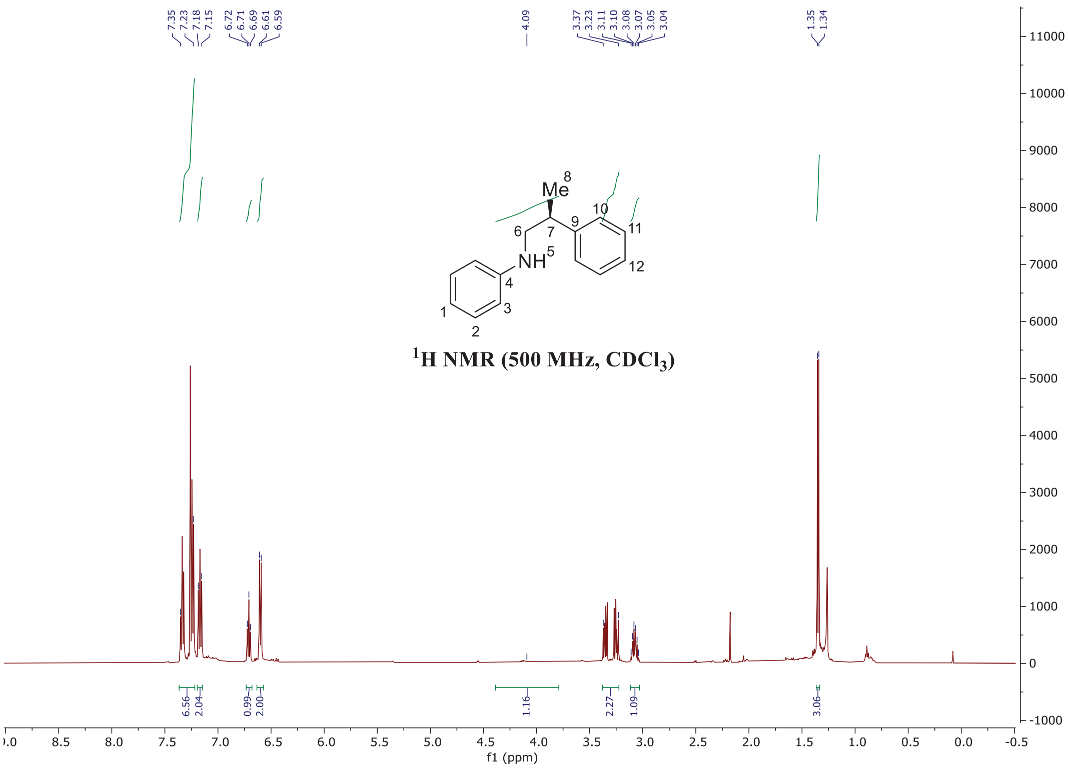

(*2S,3S*)-2-Amino-*N,N*-dimethyl-3-phenylbutanamide (9)

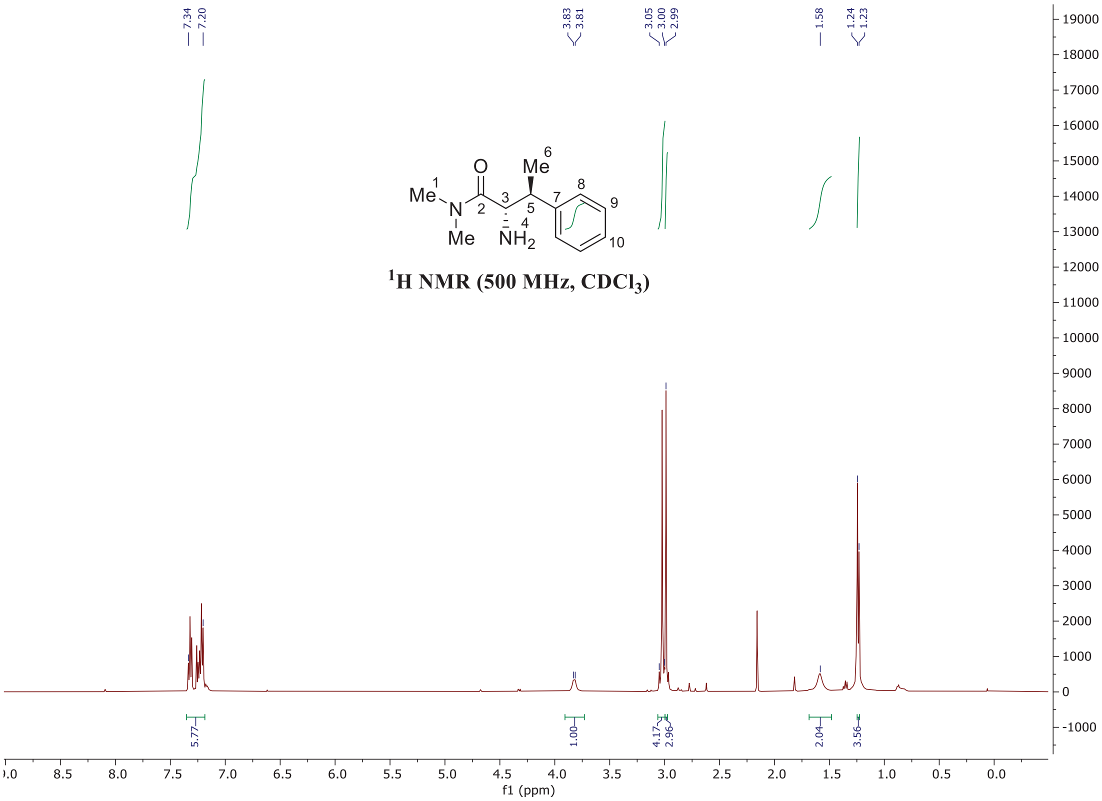

4-Bromo-*N*-((*2S*,*3S*)-1-(dimethylamino)-1-oxo-3-phenylbutan-2-yl)benzamide (10)

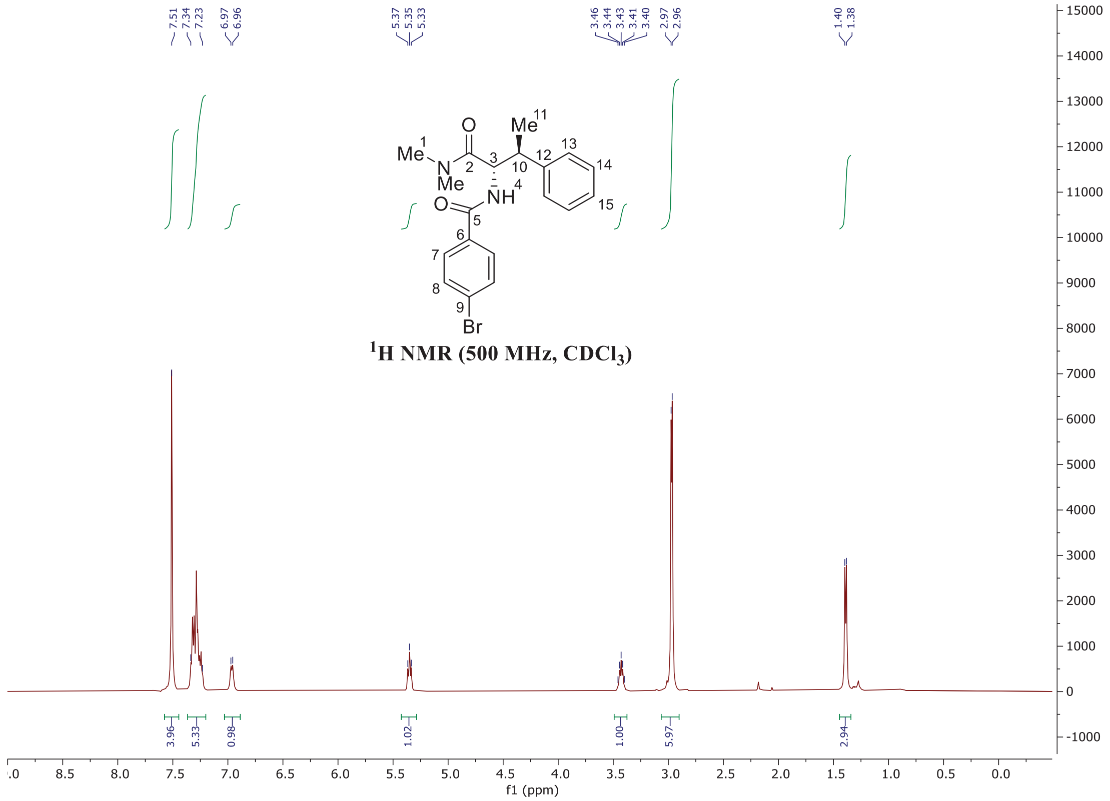

^{1}H NMR (500 MHz, $CDCl_3$)

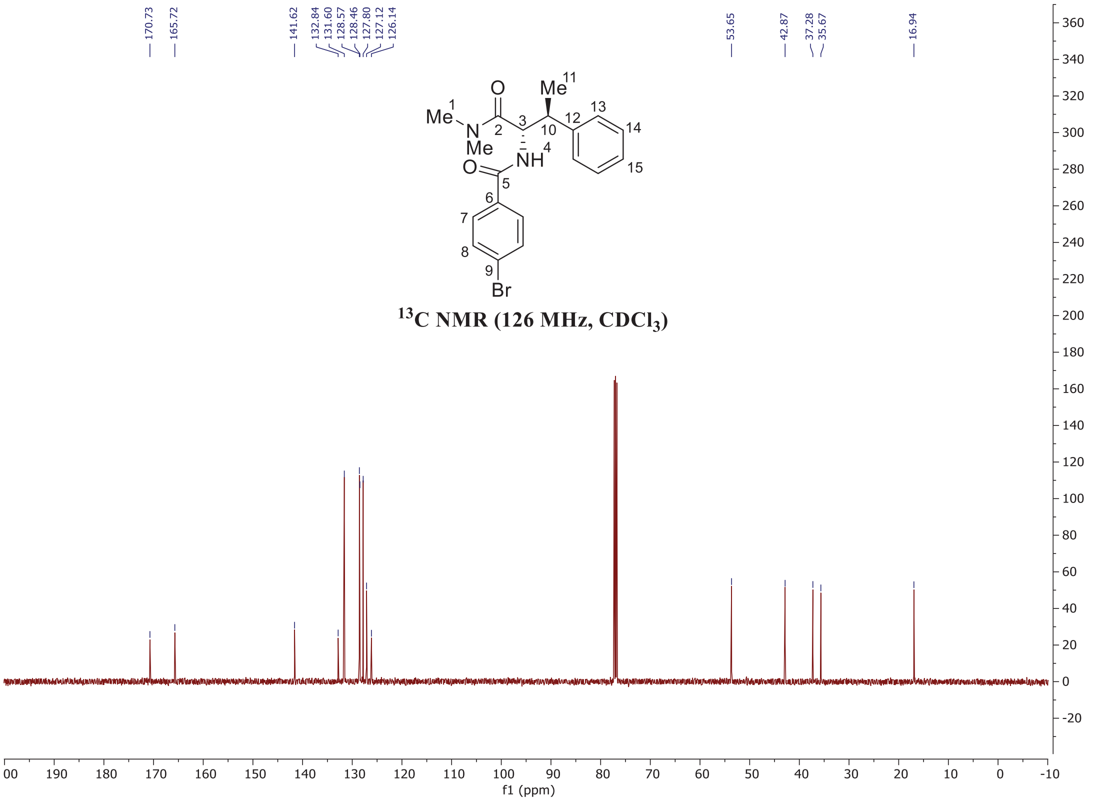

^{13}C NMR (126 MHz, $CDCl_3$)

(*2S*,*3S*)-2-Amino-3-phenylbutanoic acid (11)

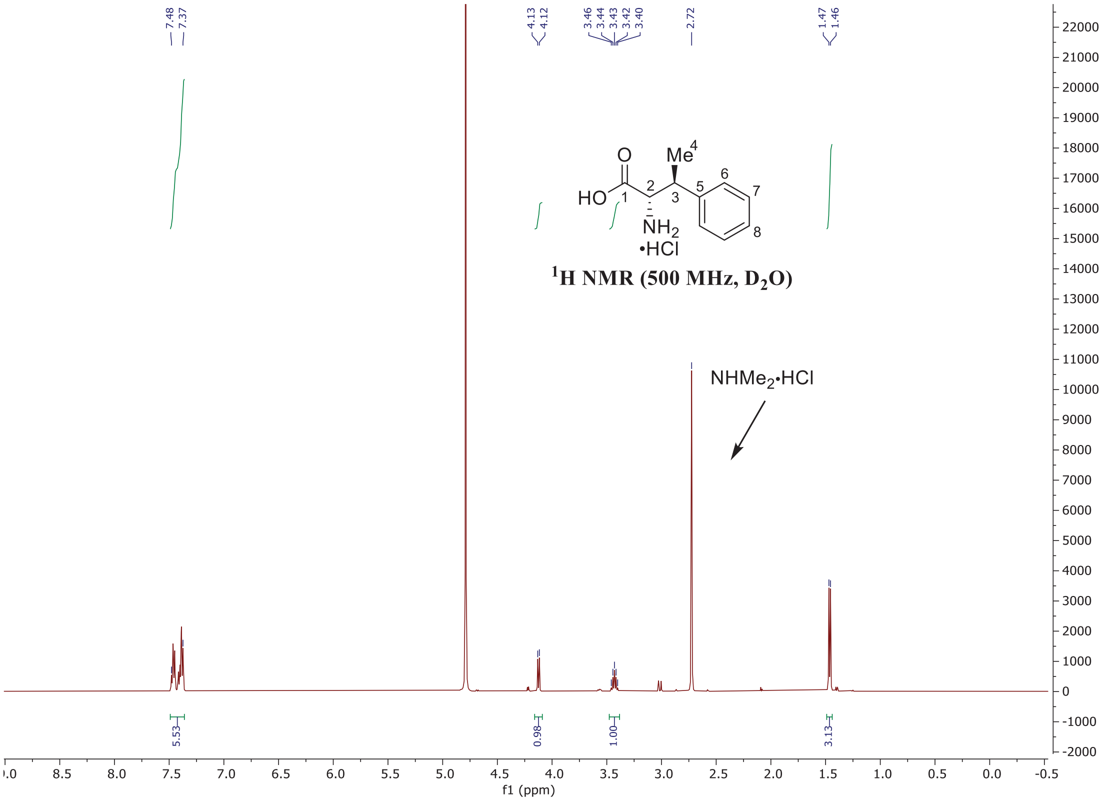

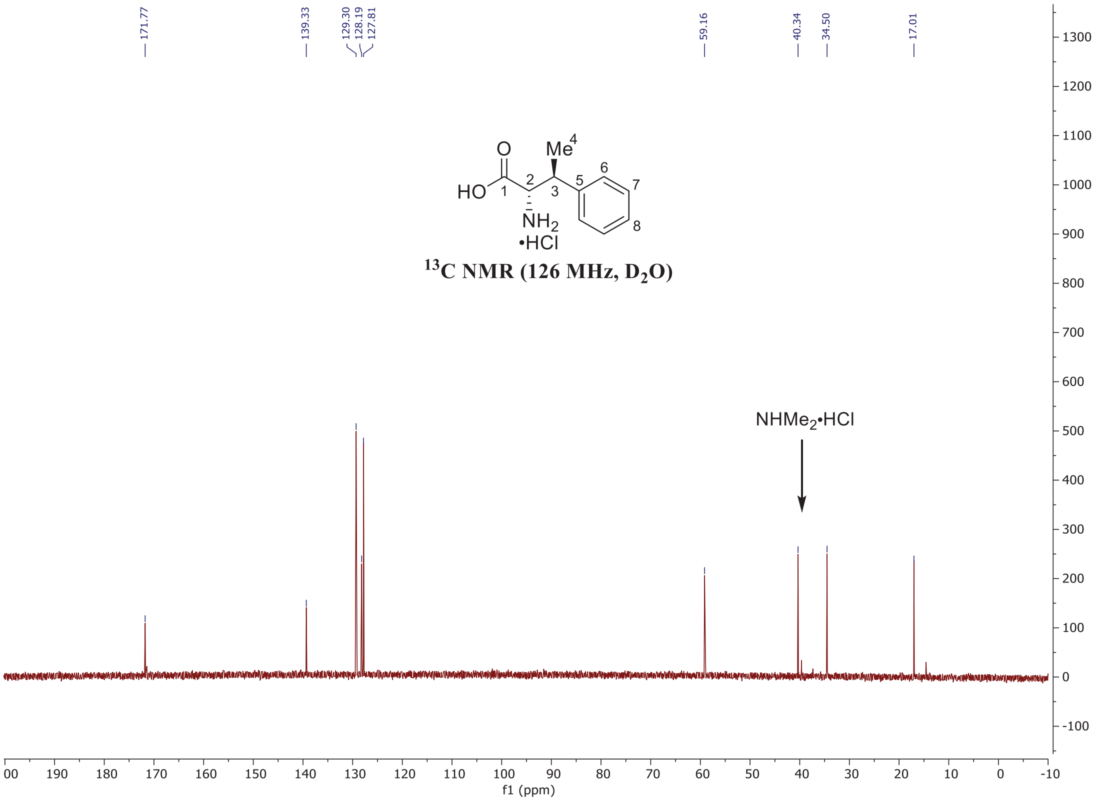

(*2S*,*3S*)-2-Amino-3-phenylbutan-1-ol (11-1)

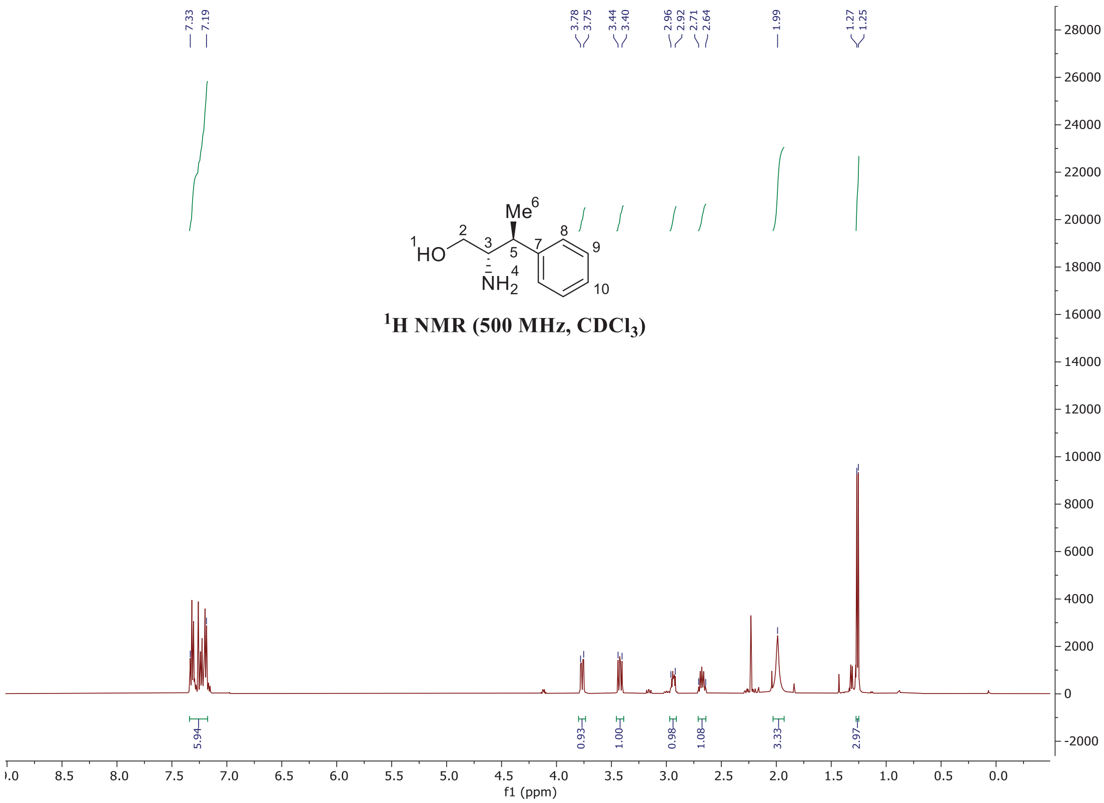

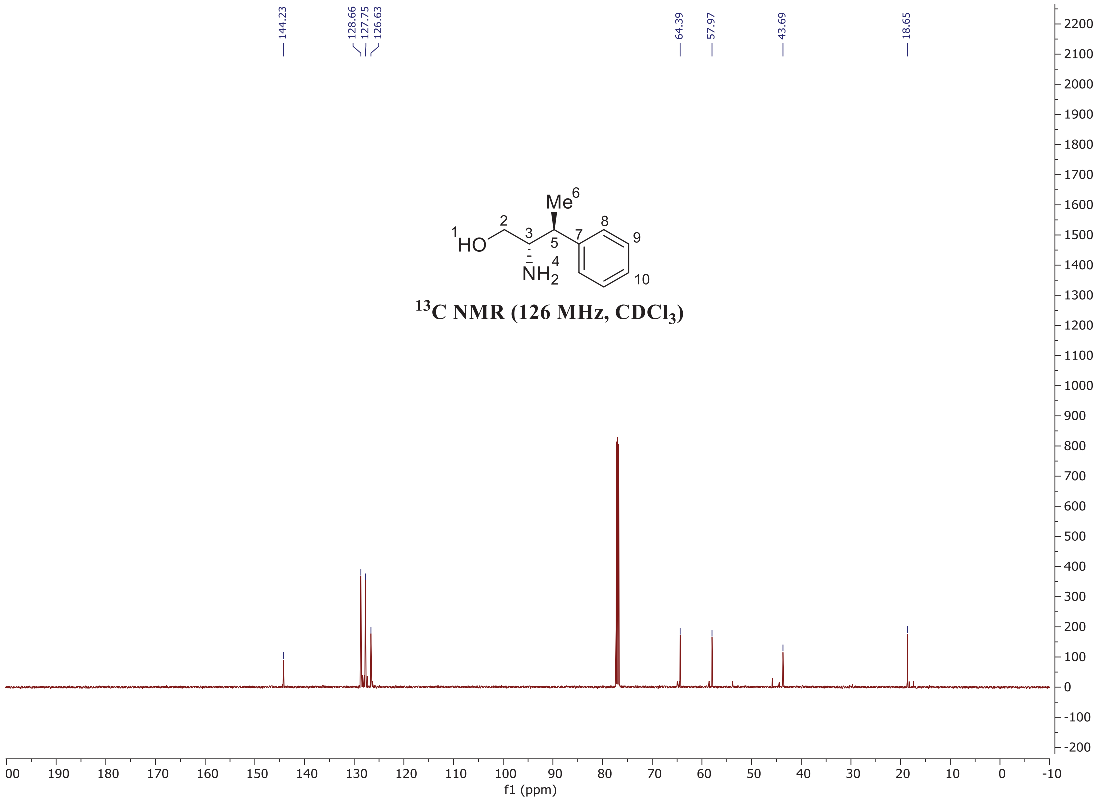

(*2S,3S*)-2-Amino-*N*,3-diphenylbutanamide (12)

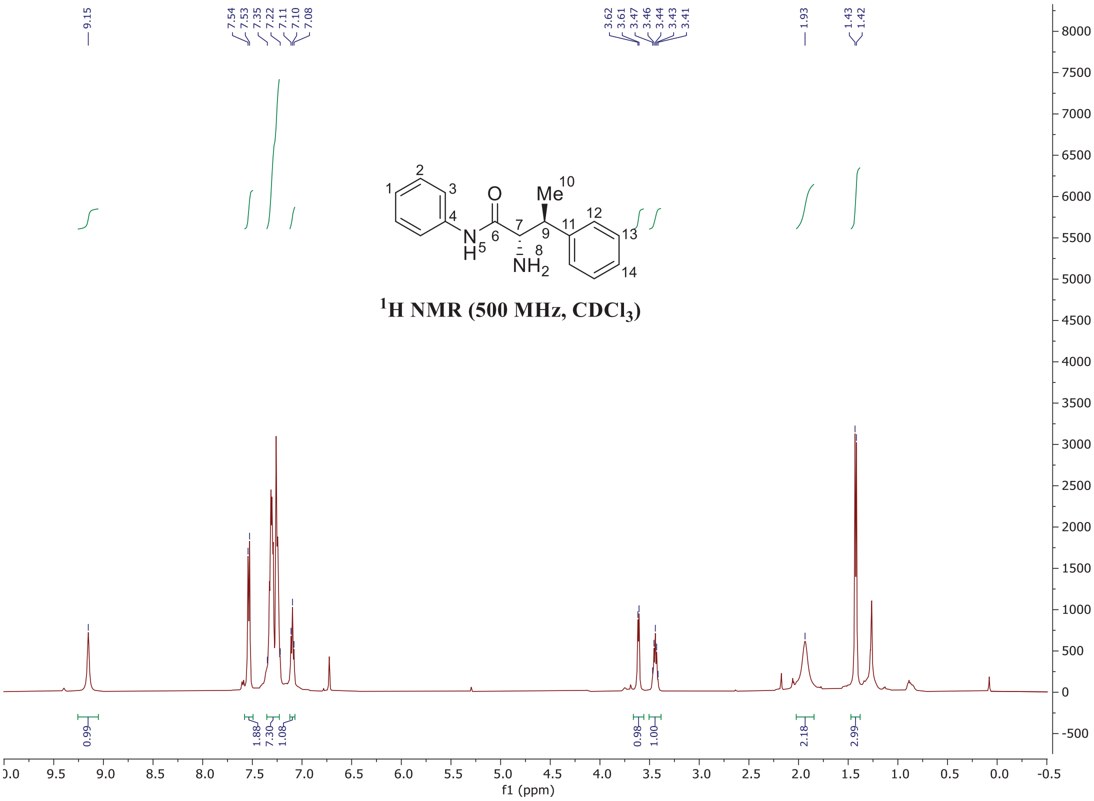

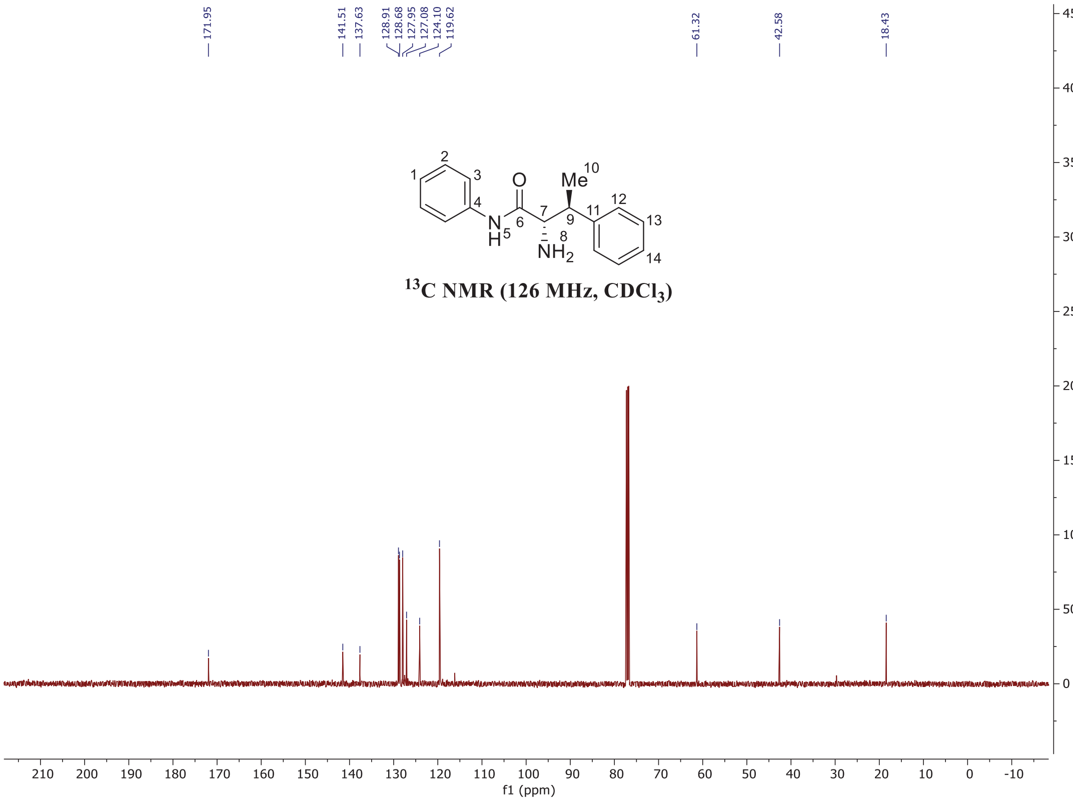

(2S,3S)-2-Amino-3-(1H-indol-3-yl)-N,N-dimethylbutanamide (13)

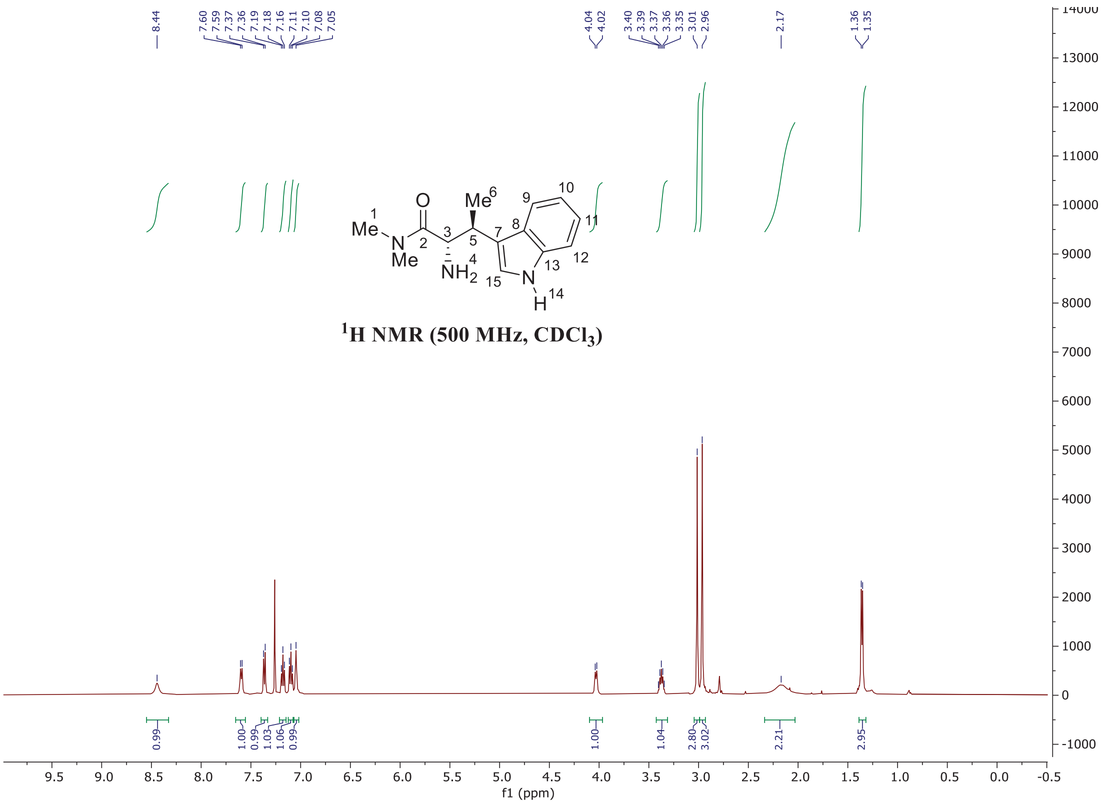

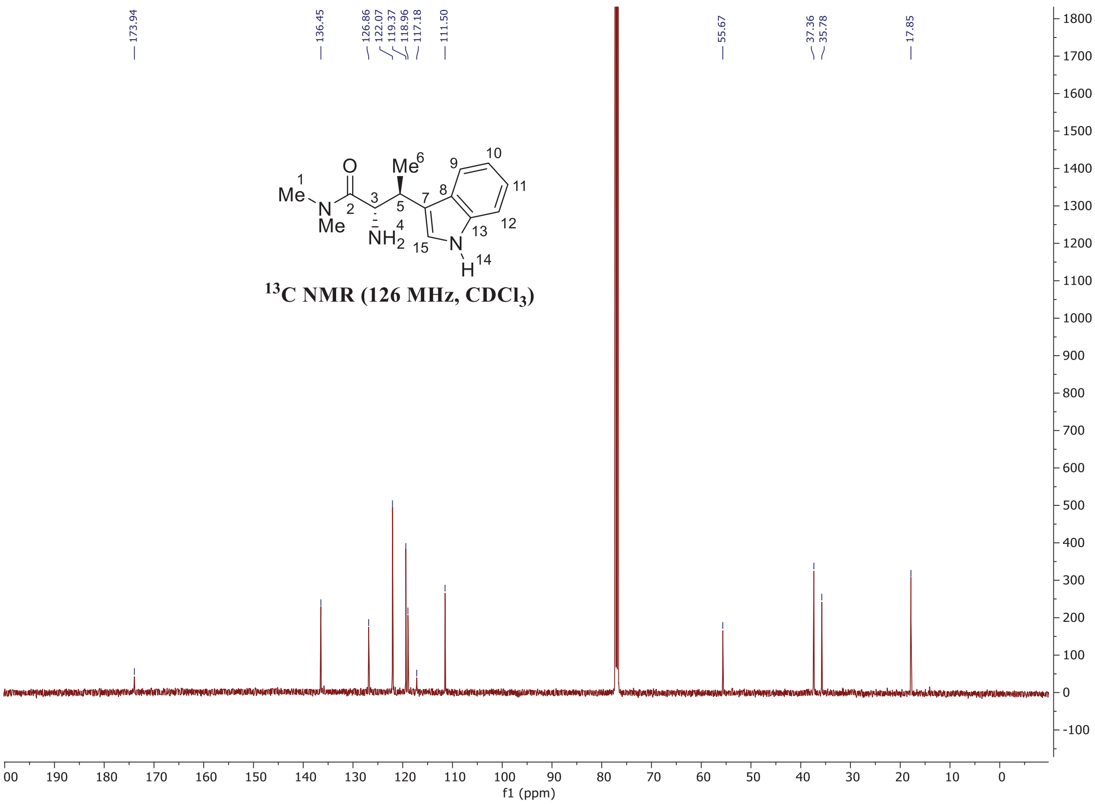

(*S*)-*N*-(2-(3-Chlorophenyl)propyl)-4-methylbenzenesulfonamide (14-2)

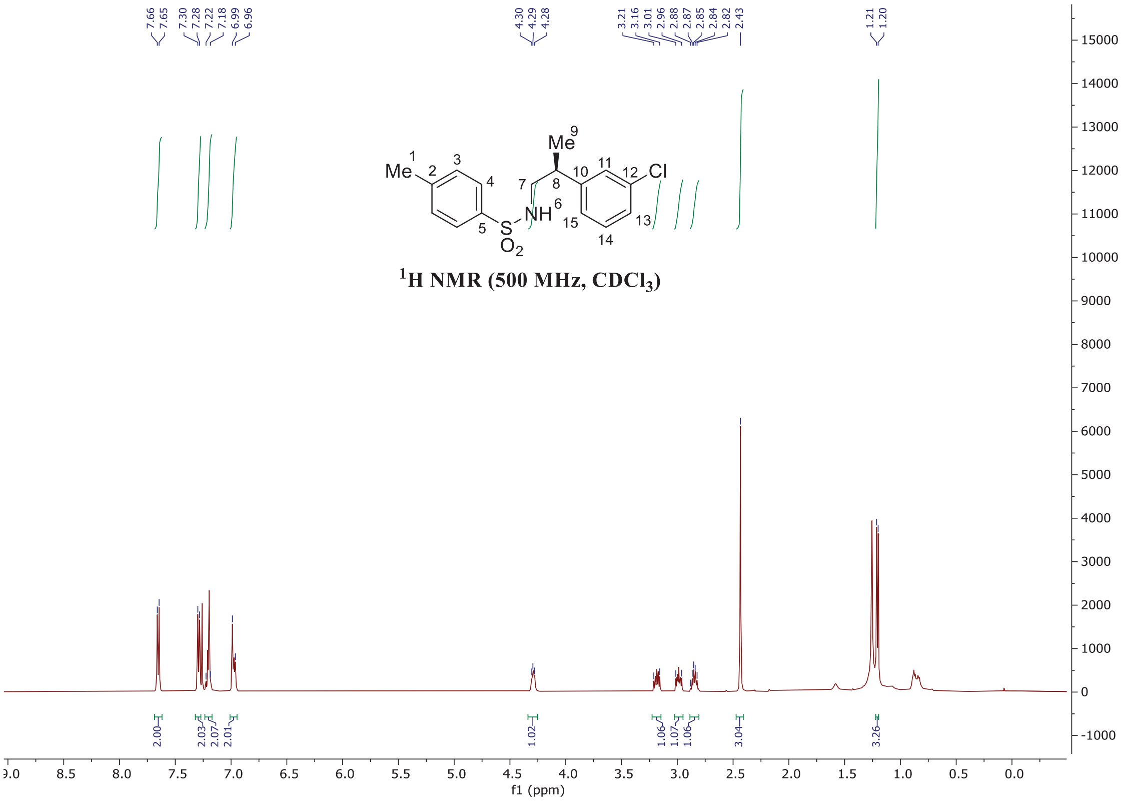

(*2R*,*3R*)-*N*,*N*-Dimethyl-3-phenyl-2-((phenylamino)methyl)butanamide (16)

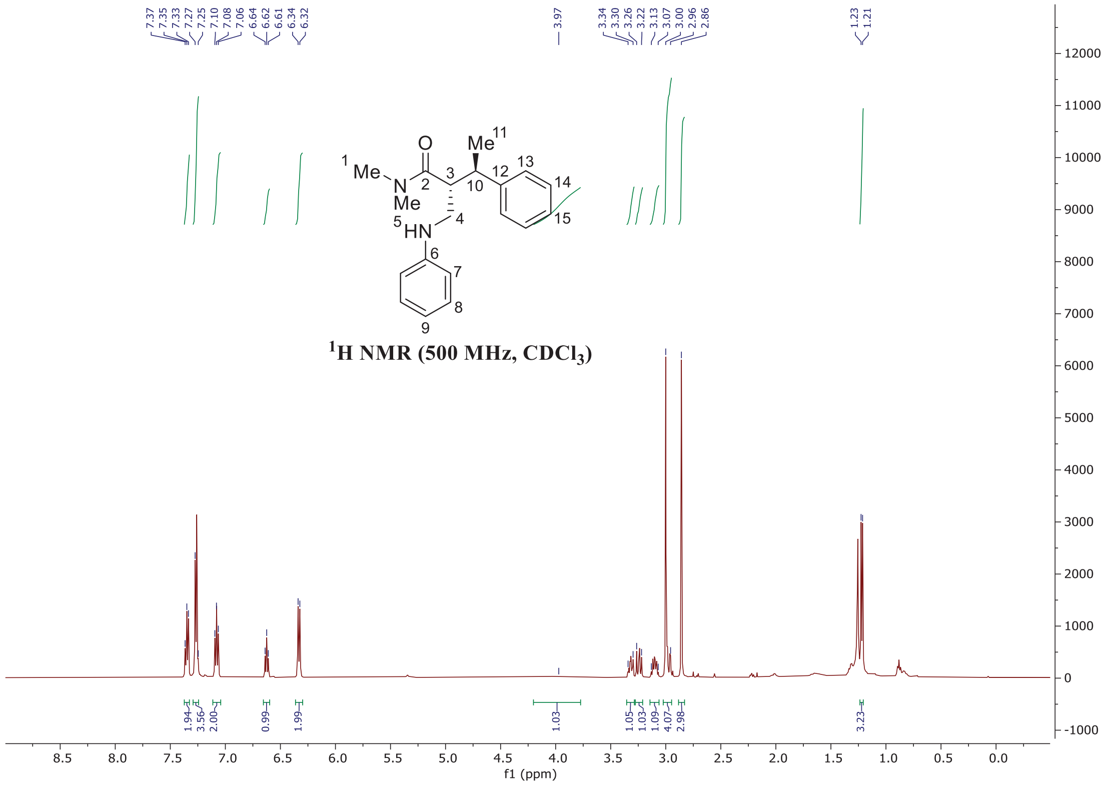

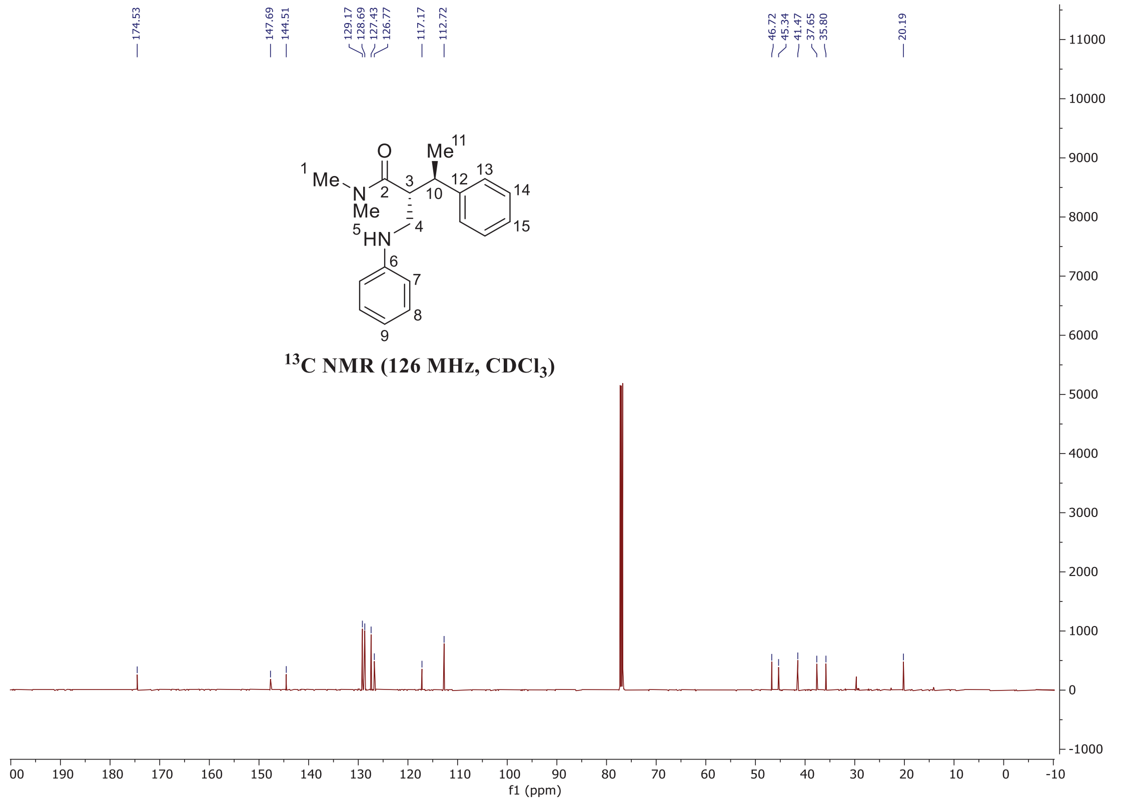
174.53
147.69
144.51
129.17
128.69
127.43
126.77
117.17
112.72
46.72
45.34
41.47
37.65
35.80
20.19
^{13}C NMR (126 MHz, CDCl$_3$)
f1 (ppm)

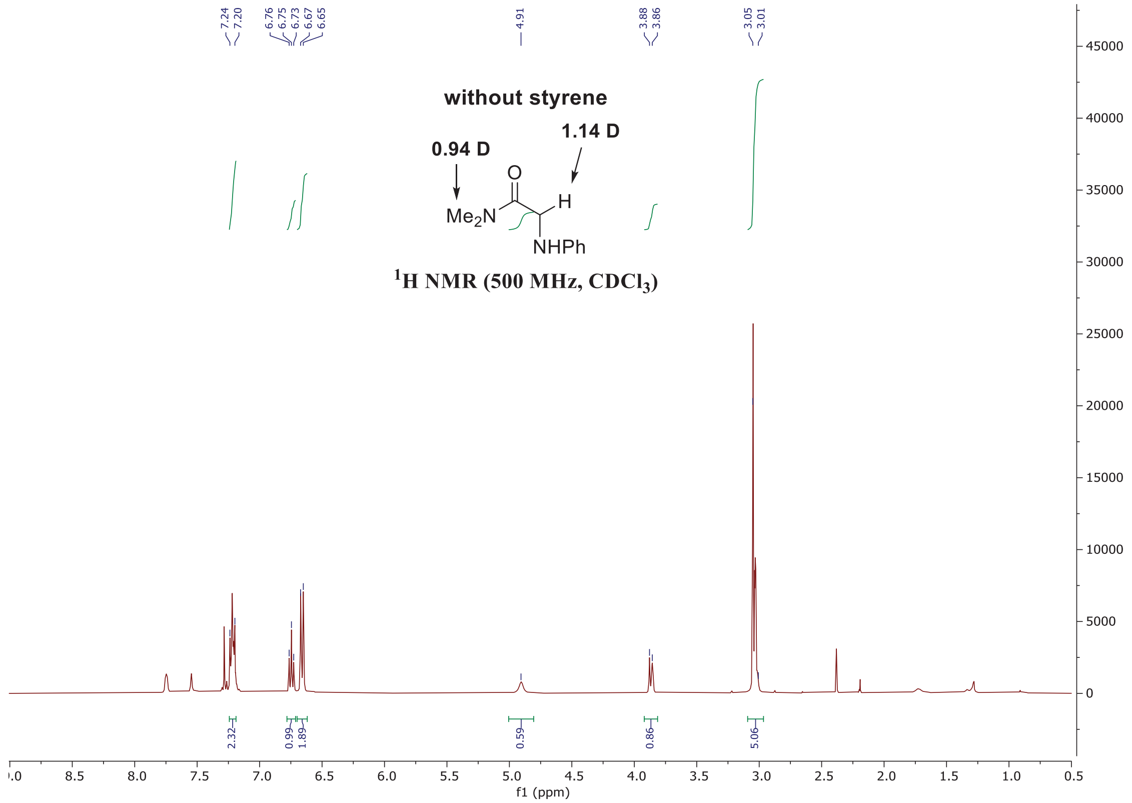
7.24
7.20
6.76
6.75
6.73
6.67
6.65
4.91
3.88
3.86
3.05
3.01
without styrene
0.94 D
1.14 D
Me$_2$N
H
NHPh
^{1}H NMR (500 MHz, CDCl$_3$)
2.32
0.99
1.89
0.59
0.86
5.06
f1 (ppm)

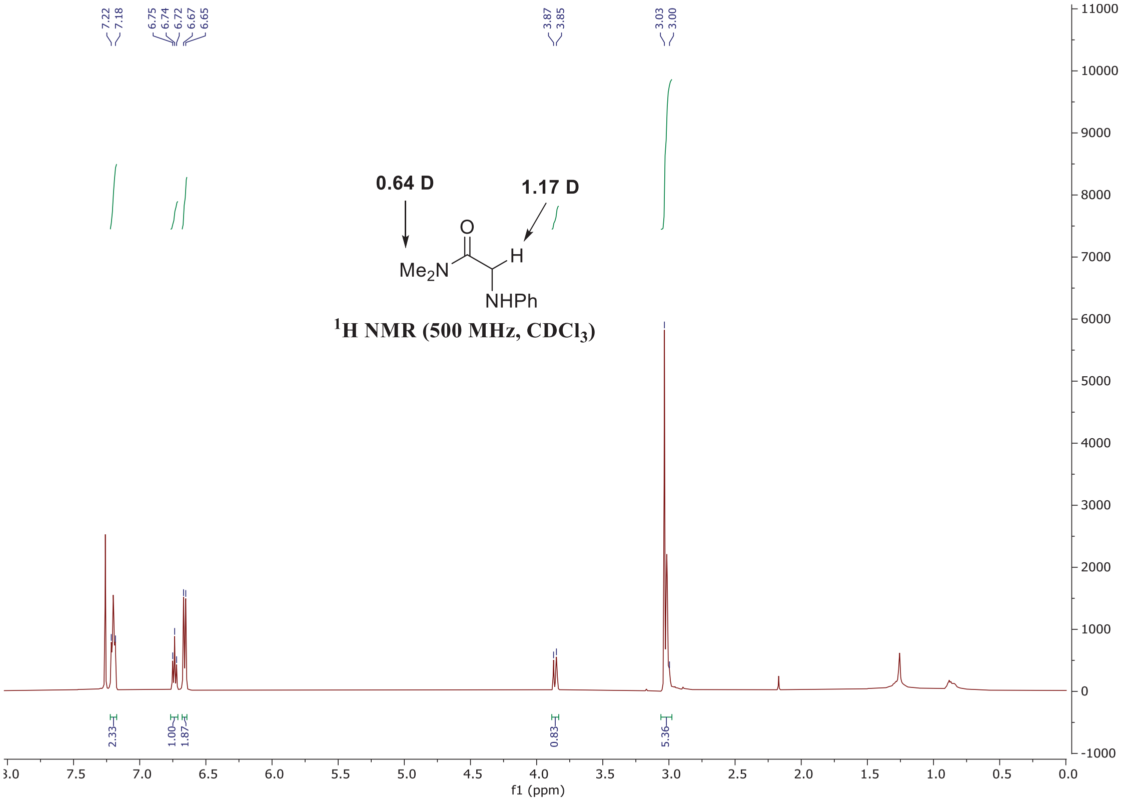
0.64 D
1.17 D
O
H
Me2N
NHPh
1H NMR (500 MHz, CDCl3)
f1 (ppm)

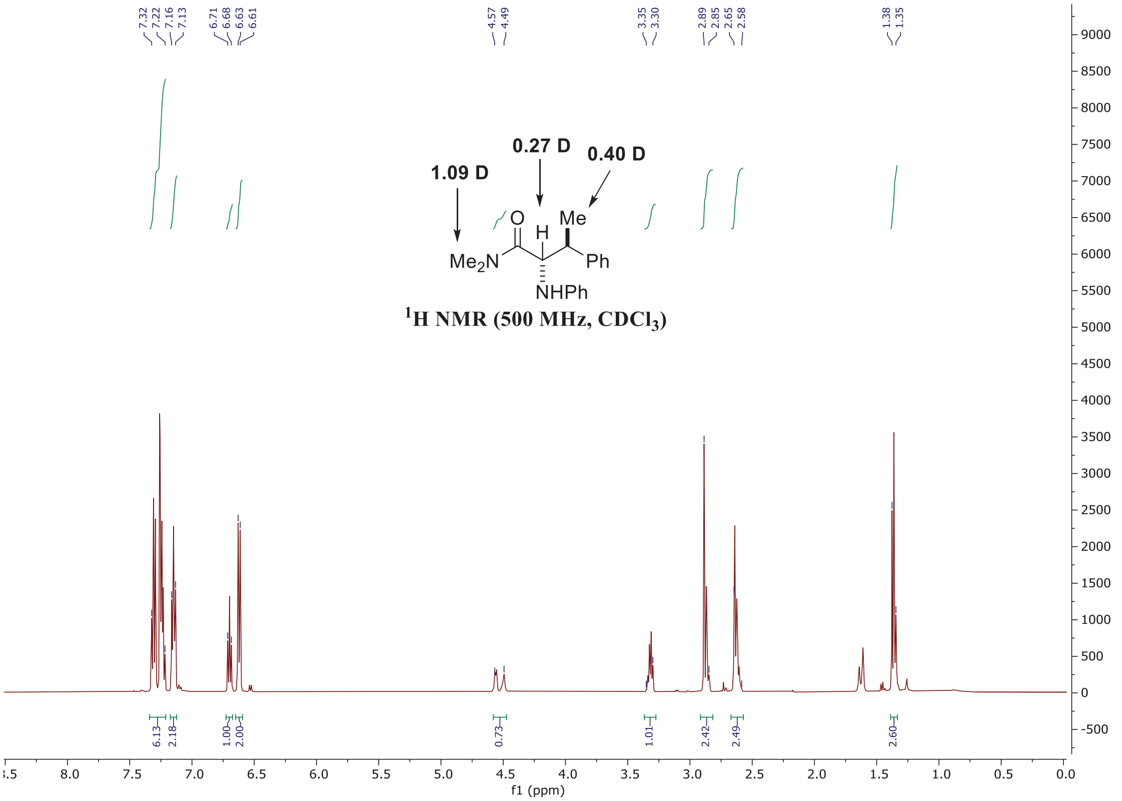
1.09 D
0.27 D
0.40 D
O
H
Me
Me2N
Ph
NHPh
1H NMR (500 MHz, CDCl3)
f1 (ppm)

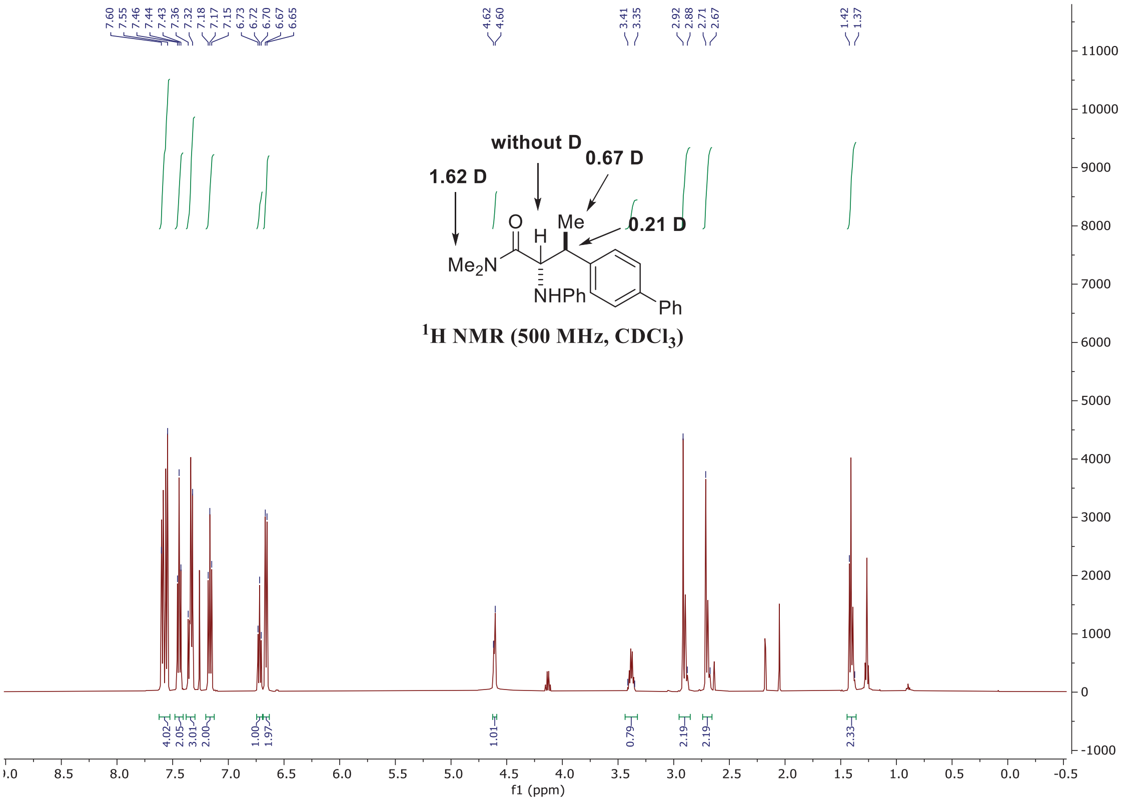
without D
0.67 D
1.62 D
0.21 D
Me
H
O
Me2N
NHPh
Ph
1H NMR (500 MHz, CDCl3)
f1 (ppm)

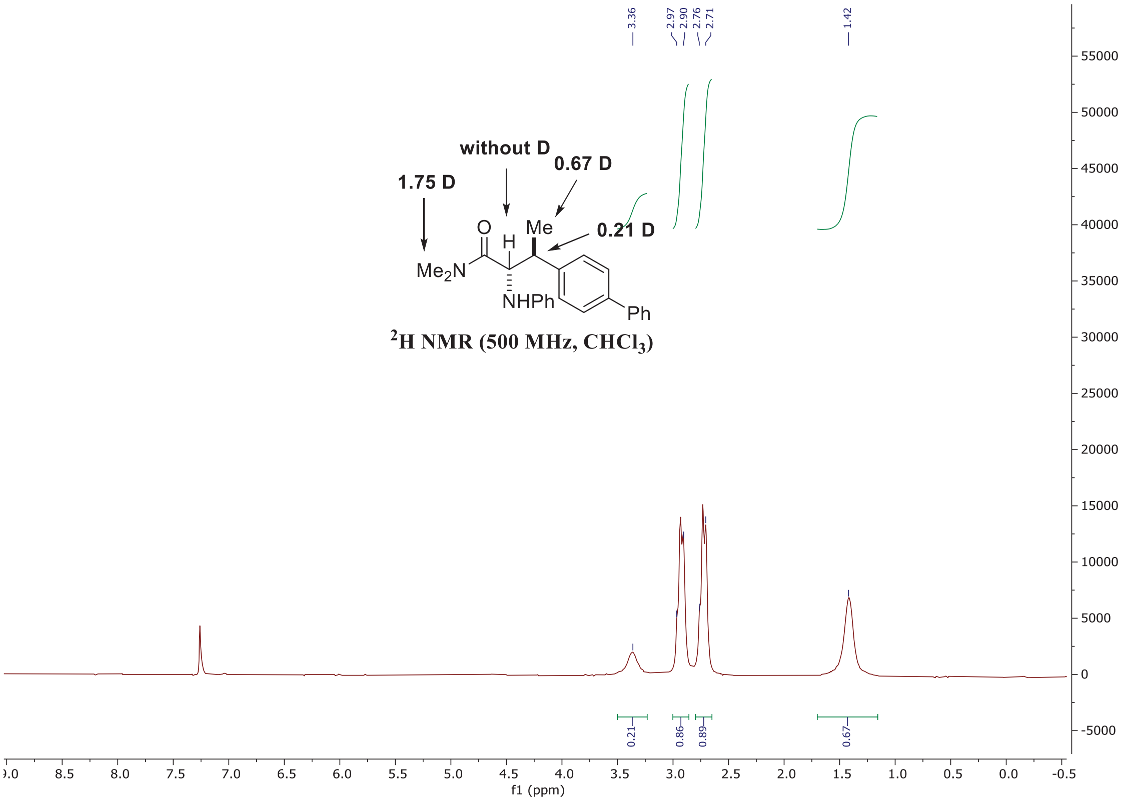
without D
0.67 D
1.75 D
0.21 D
Me
H
O
Me2N
NHPh
Ph
2H NMR (500 MHz, CHCl3)
f1 (ppm)

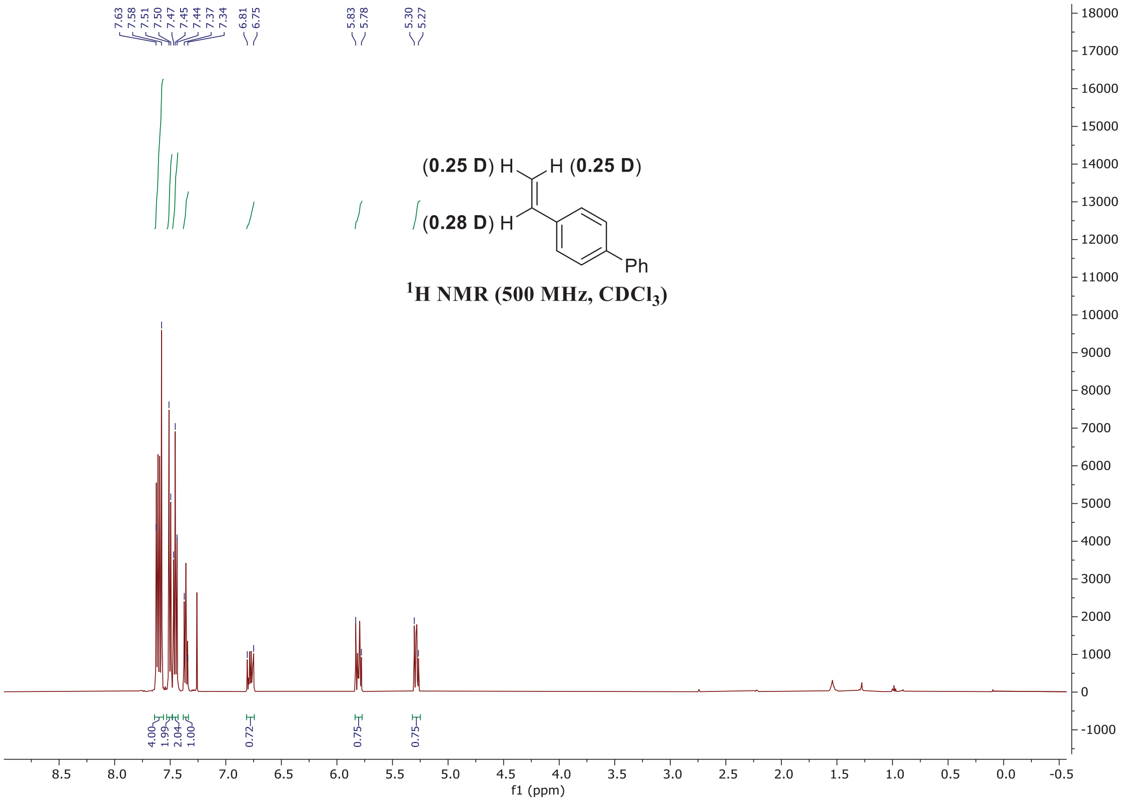
(0.25 D) H
H (0.25 D)
(0.28 D) H
Ph
1H NMR (500 MHz, CDCl3)
f1 (ppm)

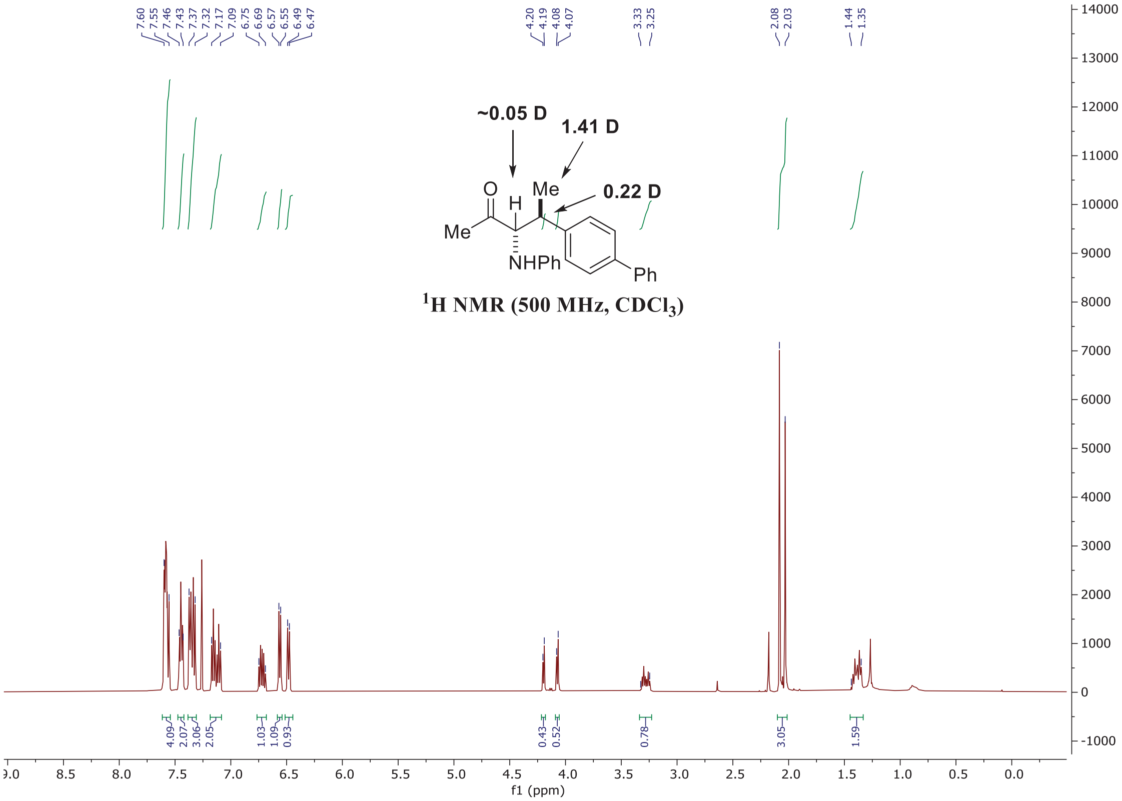
~0.05 D
1.41 D
0.22 D
O
H
Me
Me
NHPh
Ph
1H NMR (500 MHz, CDCl3)
f1 (ppm)

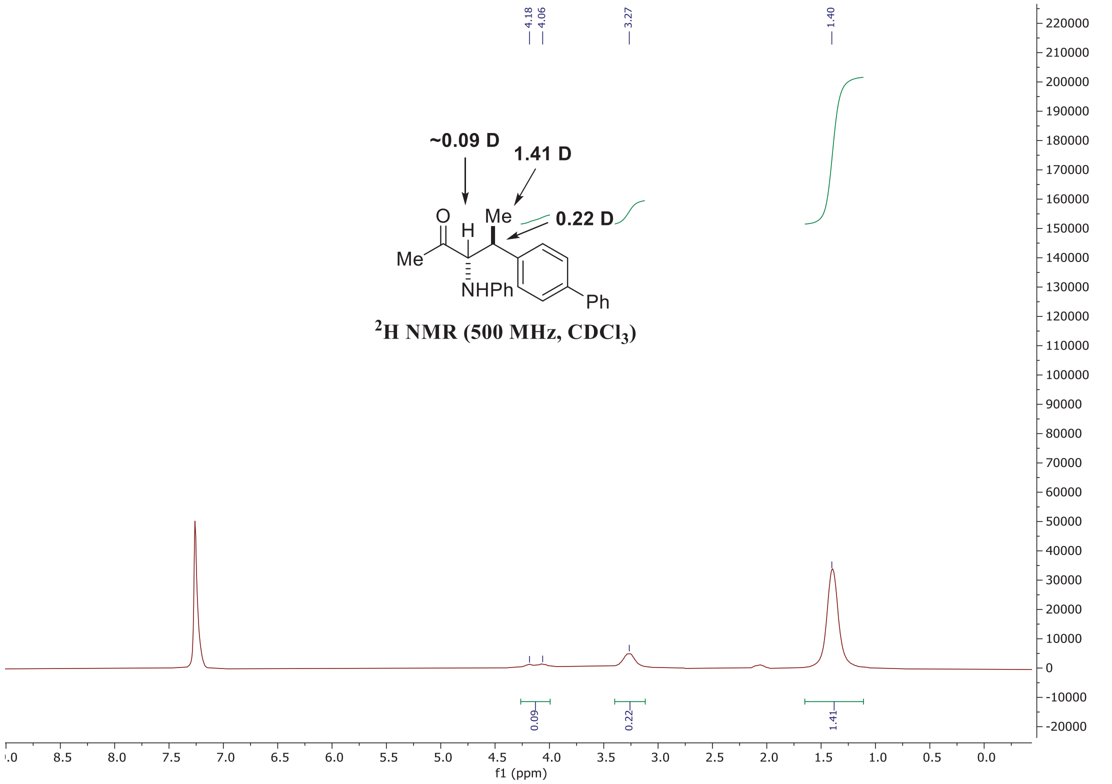

4.18
4.06
3.27
1.40
~0.09 D
1.41 D
0.22 D
O
H
Me
Me
NHPh
Ph
²H NMR (500 MHz, CDCl3)
0.09
0.22
1.41
f1 (ppm)

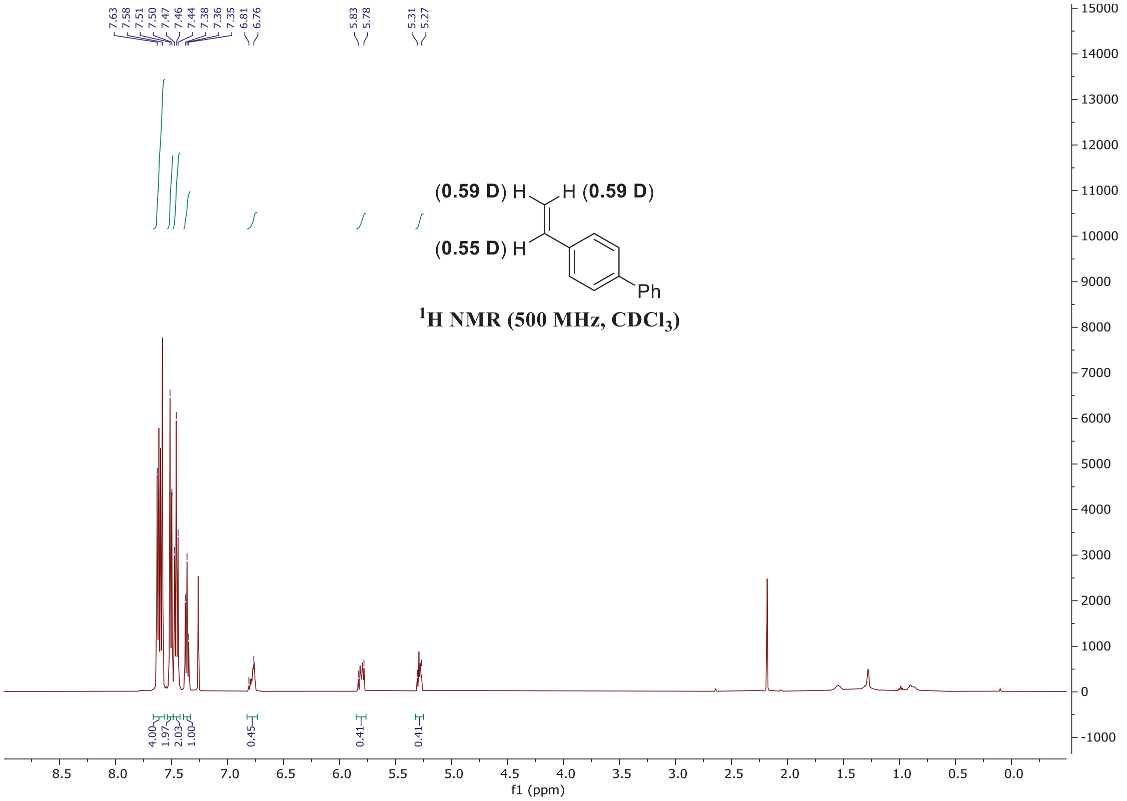

7.63
7.58
7.51
7.50
7.47
7.46
7.44
7.38
7.36
7.35
6.81
6.76
5.83
5.78
5.31
5.27
(0.59 D) H
H (0.59 D)
(0.55 D) H
Ph
¹H NMR (500 MHz, CDCl3)
4.00
1.97
2.03
1.00
0.45
0.41
0.41
f1 (ppm)
